# Adaptive randomization methods for sequential multiple assignment randomized trials (smarts) via thompson sampling

**DOI:** 10.1093/biomtc/ujae152

**Published:** 2024-12-16

**Authors:** Peter Norwood, Marie Davidian, Eric Laber

**Affiliations:** Quantum Leap Healthcare Collaborative, 499 Illinois Ave, Suite 200, San Francisco, CA 94158, United States; Department of Statistics, North Carolina State University, 2311 Stinson Drive, Campus Box 8203, Raleigh, NC 27695-8203, United States; Department of Statistical Science, Duke University, 214 Old Chemistry, Box 90251, Durham, NC 27708-0251, United States

**Keywords:** inverse probability weighted estimator, precision medicine, response-adaptive randomization, treatment regime

## Abstract

Response-adaptive randomization (RAR) has been studied extensively in conventional, single-stage clinical trials, where it has been shown to yield ethical and statistical benefits, especially in trials with many treatment arms. However, RAR and its potential benefits are understudied in sequential multiple assignment randomized trials (SMARTs), which are the gold-standard trial design for evaluation of multi-stage treatment regimes. We propose a suite of RAR algorithms for SMARTs based on Thompson Sampling (TS), a widely used RAR method in single-stage trials in which treatment randomization probabilities are aligned with the estimated probability that the treatment is optimal. We focus on two common objectives in SMARTs: (1) comparison of the regimes embedded in the trial and (2) estimation of an optimal embedded regime. We develop valid post-study inferential procedures for treatment regimes under the proposed algorithms. This is nontrivial, as even in single-stage settings standard estimators of an average treatment effect can have nonnormal asymptotic behavior under RAR. Our algorithms are the first for RAR in multi-stage trials that account for non-standard limiting behavior due to RAR. Empirical studies based on real-world SMARTs show that TS can improve in-trial subject outcomes without sacrificing efficiency for post-trial comparisons.

## 1 INTRODUCTION

A treatment regime is a sequence of decision rules, one for each key decision point in a patient’s disease progression, that maps accumulated information on a patient to a recommended treatment (Tsiatis et al., 2020). Sequential multiple assignment randomized trials (SMARTs) are the gold standard for the study of treatment regimes (Murphy, 2005) and have been applied in a range of areas, including cancer, addiction, and HIV/prevention (eg, Bigirumurame et al., 2022; Kidwell, 2014; Lorenzoni et al., 2023). A SMART involves multiple stages of randomization, each stage corresponding to a decision point, where the sets of possible treatments may depend on baseline and interim information.

**FIGURE 1:**
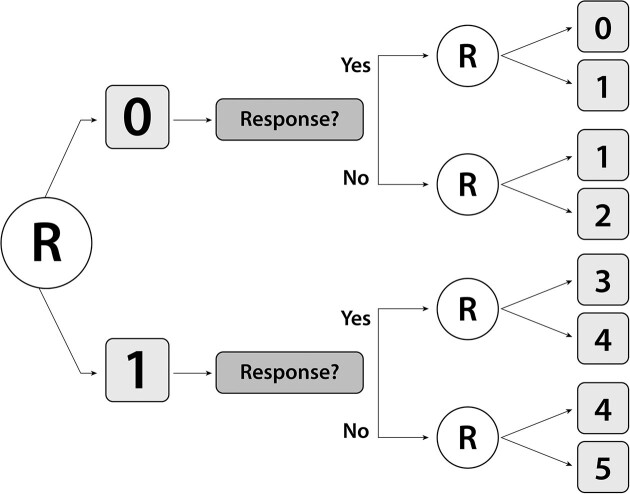
Schematic depicting the design of the SMART for evaluation of behavioral interventions for cancer pain management. The 8 embedded regimes implied by the design are of the form “Give *a* initially; if response, give *b*, otherwise if nonresponse give *c*,” where regimes 1,..., 8 correspond to $(a,b,c) =$ (0,0,1), (0,0,2), (0,1,2), (0,1,1), (1,3,4), (1,3,5), (1,4,5), and (1,4,4), respectively. At stage 2, 0 = no further intervention with PCST-Brief, 1 = PCST-Brief Maintenance, 2 = PCST-Full, 3 = no further intervention with PCST-Full, 4 = PCST-Full Maintenance, and 5 = PCST-Plus.

Figure 1 depicts a two-stage SMART to evaluate behavioral intervention strategies for cancer pain management (Somers et al., 2023). Subjects were randomized at baseline with equal probability to one of two first-stage interventions: Pain Coping Skills Training (PCST) with 5 sessions (PCST-Full, coded as 1) or one session (PCST-Brief, coded as 0). At the end of stage 1, subjects were classified as responders if they achieved a 30% reduction in pain score from baseline and as nonresponders otherwise. Subjects were then assigned with equal probability to one of two second-stage interventions depending on their first-stage intervention and response status. This design, like any fixed SMART design, can be represented as a single-stage trial in which subjects are randomized at baseline among a set of regimes known as the SMART’s embedded regimes (Tsiatis et al., 2020, Chapter 9). The cancer pain SMART has 8 embedded regimes determined by the stage 1 treatment and stage 2 treatments for responders and nonresponders, for example, one of these assigns PCST-Full initially followed by no further treatment if the subject responds and PCST-Plus otherwise. A key goal was to evaluate the embedded regimes based on mean percent reduction in pain from baseline at the end of stage 2 and to identify a regime yielding the greatest reduction.

As in this example, most SMARTs use fixed randomization probabilities at each stage. Although fixed and balanced randomization in SMARTs yields high power for comparing treatments and treatment regimes (Murphy, 2005), updating randomization probabilities based on accruing information can improve outcomes for trial subjects, increase enrollment, and decrease dropout (Food and Drug Administration, 2019). Response-adaptive randomization (RAR) uses accumulating information to skew randomization probabilities toward promising treatments and has long been used in single-stage randomized clinical trials (RCTs, Berry, 2015; Kim et al., 2011; Wang and Yee, 2019); the large literature on RAR is reviewed for single-stage RCTs by Hu and Rosenberger (2006), Berry et al. (2010), and Atkinson and Biswas (2013). RAR procedures are typically formalized as a multi-arm bandit in which each treatment option is a bandit “arm” (Berry and Fristedt, 1985; Lattimore and Szepesvári, 2020; Villar et al., 2015a). The advantages of RAR are most pronounced in single-stage RCTs with a large number of treatments, as RAR algorithms oversample more favorable treatments and undersample less favorable ones (Berry, 2011). RAR also presents challenges, for example, early evidence can lead the randomization to become “stuck,” favoring suboptimal treatments (Thall et al., 2015 ), and there have been spirited debates about its potential pitfalls (Proschan and Evans, 2020; Viele et al., 2020; Villar et al., 2021).

As the number of regimes evaluated in a SMART can be large, the use of RAR in SMARTs could have great benefits, but development is limited. Cheung et al. (2014) propose SMART-AR, an RAR method based on Q-learning (eg, Tsiatis et al., 2020, Section 5.7.1) that adapts randomization probabilities to favor treatments with large estimated Q-functions. However, the approach does not account for uncertainty in the estimated Q-functions nor the potential information gain associated with each treatment, so may be inefficient. Wang et al. (2022) propose RA-SMART, an RAR method for two-stage SMARTs with the same treatments at each stage. The method does not incorporate delayed effects, that is, it does not account for treatment effects at stage 2 that depend on stage 1 treatment. Because interactions among treatments at different stages are key to development of treatment regimes, RAR schemes that acknowledge delayed effects are desirable.

We propose RAR approaches for SMARTs based on Thompson sampling (TS, Thompson, 1933), a bandit algorithm also known as probability matching (Russo et al., 2018) that is a popular basis for RAR in single-stage RCTs (Villar et al., 2015b; Wang, 2021; Williamson and Villar, 2020). The premise of TS is that a treatment’s randomization probability should be based on confidence that the treatment is optimal. Classical TS measures confidence with a posterior probability; aligned with standard SMART methodology, we adopt a frequentist perspective in which confidence is assessed using a confidence distribution (Xie and Singh, 2013). The methods are applicable to SMARTs to evaluate a fixed set of treatment regimes and/or identify an optimal regime and account for delayed effects.

In Section 2, we present the statistical framework and a variant of TS for SMARTs, and we use the latter in Section 3 to construct the proposed RAR approaches. One class of methods randomizes subjects up-front to entire embedded regimes, while the other sequentially randomizes subjects at each decision point. We propose estimators for the marginal mean outcome under a regime in Section 3 and argue in Section 4 that they are consistent and asymptotically normal; this is nontrivial, as adaptive randomization can lead to nonnormal limits for plug-in estimators (Hadad et al., 2021; Zhang et al., 2021b). Simulations demonstrating performance are reported in Section 5.

## 2 BACKGROUND AND PRELIMINARIES

### 2.1 Notation and assumptions

We first review SMARTs with fixed (ie, nonadaptive) randomization. For a SMART with *K* stages, let $\mathcal {A}_k$ be the (finite) set of available treatment options at stage $k = 1,\ldots ,K$. For a given subject, let $A_k \in \mathcal {A}_k$ be the treatment assigned at stage *k*, and let $\overline{A}_k = (A_1,\ldots , A_k) \in \mathcal {A}_1 \times \cdots \times \mathcal {A}_k$ denote all treatments given through stage *k*. Let $\boldsymbol{X}_1$ be a set of baseline variables collected on the subject prior to stage 1 treatment, and let $\boldsymbol{X}_k$ comprise variables collected between stages $k-1$ and *k*, $k=2,\ldots ,K$. Define $\overline{\boldsymbol{X}}_k = (\boldsymbol{X}_1,\dots ,\boldsymbol{X}_k)$, $k=1,\ldots ,K$; and let $\boldsymbol{H}_1=\boldsymbol{X}_1$ and $\boldsymbol{H}_k = (\boldsymbol{X}_1,A_1,\boldsymbol{X}_2,A_2,\dots ,A_{k-1},\boldsymbol{X}_k) = (\overline{\boldsymbol{X}}_k,\overline{\boldsymbol{A}}_{k-1})$, $k=2,\ldots ,K$, denote the information at the time $A_k$ is assigned, with $\mathcal {H}_k$ denoting the domain of $\boldsymbol{H}_k$, $k=1,\ldots , K$. Let *Y* be the real-valued outcome of interest, which is measured or constructed from $\overline{\boldsymbol{X}}_{K}$ and information measured after stage *K* at a specified follow-up time, coded so that larger values are favorable. Elements of $\boldsymbol{X}_k$ may include current and past measures of the subject’s health status, previous measures of the outcome, and response status. For a subject with history $\boldsymbol{H}_k=\boldsymbol{h}_k$ at stage *k*, $\Psi _k(\boldsymbol{h}_k) \subseteq \mathcal {A}_k$ is the set of feasible treatment options in $\mathcal {A}_k$, where $\Psi _k$ maps $\mathcal {H}_k$ to subsets of $\mathcal {A}_k$ (Tsiatis et al., 2020, Section 6.2.2). For example, at stage $k=2$ of the cancer pain SMART, $\Psi _2(\boldsymbol{h}_2) = \lbrace 1, 2\rbrace$ for a subject who does not respond to intervention 0 given at the first stage. At stage *k*, a subject is randomized to option $a_k \in \Psi _k(\boldsymbol{h}_k)$ with probability $P(A_k = a_k| \boldsymbol{H}_k = \boldsymbol{h}_k)$; often, $P(A_k=a_k|\boldsymbol{H}_k=\boldsymbol{h}_k) = 1/|\Psi _k(\boldsymbol{h}_k)|$ for all *k*.

A decision rule $d_k: \mathcal {H}_k \rightarrow \mathcal {A}_k$ maps an individual’s history to a recommended treatment at stage *k*, where $d_k(\boldsymbol{h}_k) \in \Psi _k(\boldsymbol{h}_k)$ for all $\boldsymbol{h}_k \in \mathcal {H}_k$. A treatment regime $\mathbf {d}= \left(d_1,\dots ,d_K \right)$ is a sequence of decision rules, where $d_k$ is the rule for stage *k*, $k=1,\ldots ,K$. The mean outcome that would be achieved if the population were to receive treatment according to $\mathbf {d}$ can be characterized in terms of potential outcomes. For any $\overline{\boldsymbol{a}}_{k-1} = (a_1,\ldots , a_{k-1}) \in \mathcal {A}_{k-1}$, let $\boldsymbol{X}_k^{*}(\overline{\boldsymbol{a}}_{k-1})$ denote the potential subject information that would accrue between stages $k-1$ and *k*, and define $\overline{\boldsymbol{X}}_k^{*}(\overline{\boldsymbol{a}}_{k-1}) = \lbrace \boldsymbol{X}_1,\boldsymbol{X}_2^{*}(a_1),\ldots ,\boldsymbol{X}_k^{*}(\overline{\boldsymbol{a}}_{k-1})\rbrace$, $k=2,\ldots ,K$. The potential history at stage $k\ge 2$ is thus $\boldsymbol{H}_k^{*}(\overline{\boldsymbol{a}}_{k-1}) = \lbrace \overline{\boldsymbol{X}}_k^{*}(\overline{\boldsymbol{a}}_{k-1}), \overline{\boldsymbol{a}}_{k-1} \rbrace$, with $\boldsymbol{X}_1^{*}(\overline{\boldsymbol{a}}_0) = \boldsymbol{H}_1^{*}(\overline{\boldsymbol{a}}_0) = \boldsymbol{H}_1$. For any $\overline{\boldsymbol{a}}_K\in \overline{\mathcal {A}}_K$, let $Y^{*}(\overline{\boldsymbol{a}}_K)$ be the potential outcome that would be achieved under $\overline{\boldsymbol{a}}_K$. For $k=2,\ldots , K$, the potential accrued information between stages $k-1$ and *k* under regime $\mathbf {d}$ is then $\boldsymbol{X}^{*}_k(\mathbf {d}) = \sum _{\overline{\boldsymbol{a}}_{k-1} \in \overline{\mathcal {A}}_{k-1}} \boldsymbol{X}^{*}_k(\overline{\boldsymbol{a}}_{k-1}) \prod ^{k-1}_{v=1} I [d_v\lbrace \boldsymbol{H}_v^{*}(\overline{\boldsymbol{a}}_{v-1}) \rbrace = a_v]$, and the potential outcome under $\mathbf {d}$ is $Y^{*}(\mathbf {d}) = \sum _{\overline{\boldsymbol{a}}_K \in \overline{\mathcal {A}}_K} Y^{*}(\overline{\boldsymbol{a}}_K) \prod ^K_{k=1} I[d_k\lbrace \boldsymbol{H}_k^{*}(\overline{\boldsymbol{a}}_{k-1}) \rbrace = a_k]$, where $I(\cdot )$ is the indicator function. The mean outcome for regime $\mathbf {d}$, known as the value of $\mathbf {d}$, is $\mathcal {V}(\mathbf {d}) = E\lbrace Y^{*}(\mathbf {d})\rbrace$.

Define $\mathcal {W}^{*} = \lbrace \boldsymbol{X}_2^{*}(a_1),\ldots ,\boldsymbol{X}_K^{*}(\overline{\boldsymbol{a}}_{K-1}),Y^{*}(\overline{\boldsymbol{a}}_K)$ for all $\overline{\boldsymbol{a}}_K \in \mathcal {A}_K\rbrace$ to be the set of all potential outcomes. For a given regime $\mathbf {d}$, identification of $\mathcal {V}(\mathbf {d})$ is possible from the data $(\overline{\boldsymbol{X}}_K,\overline{\boldsymbol{A}}_K,Y)$ under the following assumptions, which are discussed extensively elsewhere (eg, Tsiatis et al., 2020, Section 6.2.4) and which we adopt: (1) consistency, $Y = Y^{*}(\overline{\boldsymbol{A}}_K)$, $\boldsymbol{H}_k = \boldsymbol{H}_k^{*}(\overline{\boldsymbol{A}}_{k-1})$, $k=1,\ldots ,K$; (2) positivity, $P(A_k = a_k| \boldsymbol{H}_k = \boldsymbol{h}_k) > 0$ for all $a_k \in \Psi _k(\boldsymbol{h}_k)$ and all $\boldsymbol{h}_k \in \mathcal {H}_k$, $k=1,\ldots ,K$; and (3) sequential ignorability, $\mathcal {W}^{*} \perp\!\!\!\perp A_k \, | \, \boldsymbol{H}_k$, $k=1,\ldots ,K$, where “$\perp\!\!\!\perp$” denotes statistical independence. We also assume that there is no interference among individuals nor multiple versions of a treatment. In a SMART with nonadaptive randomization, (1) is true by design, and (2) is guaranteed by randomization; with RAR, (3) holds if constraints are imposed on the randomization probabilities (discussed shortly) and (4) holds because randomization probabilities are known features of the history.

Denote the *m* regimes embedded in a SMART as $\mathbf {d}^1,\ldots ,\mathbf {d}^m$. A common primary analysis is the comparison of $\mathcal {V}(\mathbf {d}^j)$, $j=1,\ldots ,m$, that is, the mean outcomes that would be achieved if the population were to receive treatments according to $\mathbf {d}^1,\ldots ,\mathbf {d}^m$. Another common analysis is identification of an optimal embedded regime, $\mathbf {d}^{\mathrm{opt}}$, satisfying $\mathcal {V}(\mathbf {d}^{\mathrm{opt}}) \ge \mathcal {V}(\mathbf {d}^j)$, $j=1,\ldots ,m$.

### 2.2 Subject accrual and progression processes

Ordinarily, it is assumed that subjects enroll in a clinical trial by a completely random process over a planned accrual period. Under such a process, subjects in a SMART progress through the stages in a staggered fashion. Thus, at any point during the trial, enrolled subjects will have reached different stages, with only some having the outcome *Y* ascertained. By reaching stage *k*, we mean that a subject has completed the previous stages and that $A_k$ has been assigned. For example, in a SMART with $K=2$ stages, at any time, there may be subjects who have received only a first-stage treatment, for whom $(\boldsymbol{X}_1,A_1)$ is available; subjects who have reached stage 2 but have not completed follow-up, for whom $(\boldsymbol{X}_1,A_1,\boldsymbol{X}_2,A_2)$ is available; and subjects who have completed the trial, for whom $(\boldsymbol{X}_1,A_1,\boldsymbol{X}_2,A_2,Y)$ is available.

Ideally, randomization probabilities would be updated each time a new randomization is needed. However, it may be more feasible in practice to update the probabilities only periodically; see Web Appendix A of the Supplementary Material for discussion. For definiteness, we consider a SMART that will enroll *N* subjects over *T* weeks, with randomization probabilities updated weekly. We develop two RAR randomization schemes: the first, which we term up-front, randomizes subjects to an embedded regime at baseline; the second, termed sequential, randomizes subjects at each stage. While these two schemes are equivalent in a SMART with fixed randomization probabilities, the sequential scheme under RAR allows a subject’s probabilities to depend on information collected as they progress through the trial.

We assume that a group of subjects of random size enrolls at each week *t* and, depending on the randomization scheme, up-front or sequential, requires assignment to either an entire regime (up-front) or a stage 1 treatment (sequential) according to probabilities that are updated at *t* based on the accrued data from subjects previously enrolled at weeks $t-1, t-2,\ldots ,0$. Each of these subjects is assigned a regime (up-front) or $A_1$ (sequential) using these probabilities. At each subsequent stage $k=2,\ldots ,K$, we assume that there is a time interval, measured in weeks, between when $A_{k-1}$ is assigned and when $\boldsymbol{X}_k$ is ascertained and $A_k$ is assigned, as well as a follow-up period after $A_K$ is assigned but before *Y* is recorded. Thus, under sequential randomization, these subjects will require randomization to $A_k$, $k = 2,\ldots ,K$, at a future week $(t+s)$, say, using probabilities based on the accrued data from subjects previously enrolled at or before week $(t+s-1)$.

To represent the data available on subjects who are already enrolled at any week *t*, let $\Gamma _{t} \in \lbrace 0,1\rbrace$ be the indicator that a subject has enrolled in the SMART and been assigned to a regime or stage 1 treatment by week *t* (ie, at a week $\le t$). For such subjects, let $\tau \le t$ be the week of enrollment and assignment; $\kappa _t \in \lbrace 1,\ldots ,K\rbrace$ be the most recent stage reached by week *t*; and $\Delta _{t} \in \lbrace 0, 1\rbrace$ be the indicator that a subject has completed follow-up by week *t*, so that *Y* has been observed. The data available on a given subject at week *t* are then $D_{t} = \Gamma _{t}\lbrace 1, \tau ,\kappa _{t}, \boldsymbol{X}_1, A_1, I(\kappa _{t}> 1)\boldsymbol{X}_2, I(\kappa _{t}> 1)A_2, \ldots , I(\kappa _{t}> K-1)\boldsymbol{X}_K, I(\kappa _{t}> K-1)A_K, \Delta _{t}, \Delta _{t}Y \rbrace$; $D_t$ is null if a subject has not yet enrolled by *t*. Indexing subjects by *i*, the accrued data from all previously enrolled subjects that can inform the randomization probabilities at week *t* are then $\mathfrak {D}_{t-1} = \lbrace D_{t-1,i}, \forall i { such that } \Gamma _{t-1,i} = 1\rbrace$.

### 2.3 Thompson sampling

The central idea of TS is to map a treatment’s randomization probability to the belief that it is optimal among the available options. This belief is represented conventionally by the posterior probability that a treatment is optimal in the sense that it optimizes expected outcome (Thompson, 1933). For example, in the up front case, let $\widehat{\rho }_{t}^j \in [0,1]$ be the estimated belief that treatment regime $j=1,\ldots ,m$ is optimal at week *t* based on the accrued data $\mathfrak {D}_{t-1}$ from previously enrolled subjects (and similarly for treatment *j* in a single-stage RCT). Let $r_{t}^j$ be regime *j*’s randomization probability for subjects needing randomization at week *t*. Ordinarily, $r_{t}^j$ is taken to be a monotone function of $\widehat{\rho }_{t}^j$; a popular choice is $r_{t}^j= (\widehat{\rho }_{t}^j)^{c_t} / \sum _{v=1}^m (\widehat{\rho }_{t}^v)^{c_t},$ where $c_t \in [0,1]$ is a damping constant (Thall and Wathen, 2007). Smaller values of $c_t$ pull the probabilities toward uniform randomization; higher values pull them closer to the beliefs. The $c_t$ can be the same for all *t* or increase with *t* to impose greater adaptation as data accumulate. Because aggressive adaptation can lead to randomization probabilities approaching 0 or 1 for some regimes or treatments, limiting exploration of all options, one may impose clipping constants, that is, lower/upper bounds on the probabilities (Zhang et al., 2021a). This practice is consistent with the positivity assumption.

In a fully Bayesian formulation, beliefs $\widehat{\rho }_{t}^j$ are estimated posterior probabilities based on $\mathfrak {D}_{t-1}$. We propose a frequentist analog based on the so-called confidence distribution (Xie and Singh, 2013). Here, we provide a basic overview of the approach; details for the up-front and sequential algorithms are in subsequent sections. Let $\boldsymbol{\theta }$ be the vector of parameters that, with a subject’s current history, determines a subject’s optimal treatment; for example, under up-front randomization, $\boldsymbol{\theta }= (\theta ^1,\ldots ,\theta ^m)^T = \lbrace \mathcal {V}(\mathbf {d}^1),\ldots ,\mathcal {V}(\mathbf {d}^m)\rbrace ^T$. Let $\widehat{\mathcal {P}}_{\boldsymbol{\theta },t}$ be the estimated confidence distribution for $\boldsymbol{\theta }$ at week *t* based on $\mathfrak {D}_{t-1}$, for example, the estimated asymptotic distribution of some estimator $\widehat{\boldsymbol{\theta }}_t$ for $\boldsymbol{\theta }$; and let $\widetilde{\boldsymbol{\theta }}^b_t = (\widetilde{\theta }^{1,b}_{t},\ldots , \widetilde{\theta }^{m,b}_{t})^T$, $b=1,\ldots ,B$, be independent draws from $\widehat{\mathcal {P}}_{\boldsymbol{\theta },t}$. Then for $j=1,\ldots ,m$, $\widehat{\rho }_{t}^j = B^{-1} \sum _{b=1}^B I \lbrace \widetilde{\theta }^{j,b}_{t}=\max (\widetilde{\theta }^{1,b}_{t},\ldots , \widetilde{\theta }^{m,b}_{t}) \rbrace$.

At the onset of a SMART, a burn-in period of nonadaptive randomization may be required from which to obtain an initial estimate of $\boldsymbol{\theta }$ and associated confidence distribution to be used for the first update of randomization probabilities. The burn-in may be characterized in terms of numbers of subjects who have completed each stage or the calendar time elapsed since the start of the trial; examples are presented in Section 5. In what follows, $t^{*}$ is the calendar time at which burn-in is complete, so that adaptation starts at week $t = t^{*}+1$. See Web Appendix A for further discussion of specification of the burn-in period.

## 3 ADAPTIVE RANDOMIZATION FOR SMARTS USING THOMPSON SAMPLING

We now present the proposed up-front and sequential TS approaches to RAR for SMARTs. As above, under up-front randomization, subjects are randomized once, at enrollment, to an embedded regime, which they follow through all *K* stages. Thus, the path that a subject will take through the trial is determined by $\mathfrak {D}_{t-1}$. Up-front randomization is logistically simpler but does not use additional data that have accumulated as the subject progresses through the trial. Up-front randomization is preferred when simplicity of implementation is a priority or when enrollment is expected to be slow relative to the time to progress through the trial, so that the amount of new data accumulating before a subject completes all *K* stages is modest. Under sequential randomization, subjects are randomized at each stage, so that up-to-date information from previous subjects informs randomization probabilities as a subject progresses, but involves greater logistical complexity.

### 3.1 Up-front randomization among regimes

To randomize newly enrolled subjects at week *t* to the embedded regimes $\mathbf {d}^j$, $j=1,\ldots ,m$, we require an estimator $\widehat{\boldsymbol{\theta }}_t$ for $\boldsymbol{\theta }=(\theta ^1,\ldots ,\theta ^m)^T=\lbrace \mathcal {V}(\mathbf {d}^1),\ldots ,\mathcal {V}(\mathbf {d}^m)\rbrace ^T$ based on $\mathfrak {D}_{t-1}$ with which to construct a confidence distribution and thus randomization probabilities $r_{t}^j$. Aligned with standard methods for SMARTs, we focus on inverse probability weighted (IPW) and augmented IPW (AIPW) estimators for $\theta ^1,\ldots ,\theta ^m$ (Tsiatis et al., 2020, Sections 6.4.3-6.4.4). As we discuss in Section 4, under any form of RAR, the asymptotic distribution of these estimators based on the data at the end of the trial need not be normal; thus, we adapt the approach of Zhang et al. (2021b) and propose weighted versions of these estimators, where the weights are chosen so that the weighted estimators are asymptotically normal. For stratified sampling, the formulation applies within each stratum.

For subjects who enroll at week *t*, define $\eta _{t,1}(a_1, \boldsymbol{x}_1, \mathfrak {D}_{t-1}) = P(A_1 = a_1| \boldsymbol{X}_1 = \boldsymbol{x}_1, \mathfrak {D}_{t-1}) = P(A_1 = a_1| \boldsymbol{H}_1 = \boldsymbol{h}_1, \mathfrak {D}_{t-1})$ and, for $k = 2,\ldots , K$, $\eta _{t,k}(a_k, \overline{\boldsymbol{x}}_k,\overline{\boldsymbol{a}}_{k-1},\mathfrak {D}_{t-1}) = P(A_k = a_k| \overline{\boldsymbol{X}}_k= \overline{\boldsymbol{x}}_k,\overline{\boldsymbol{A}}_{k-1}=\overline{\boldsymbol{a}}_{k-1}, \mathfrak {D}_{t-1}) =P(A_k = a_k| \boldsymbol{H}_k=\boldsymbol{h}_k, \mathfrak {D}_{t-1})$. For regime $\mathbf {d}$, let $\overline{d}_1(\boldsymbol{x}_1) = d_1(\boldsymbol{x}_1)$, $\ldots$, $\overline{d}_k(\overline{\boldsymbol{x}}_k) = [d_1(\boldsymbol{x}_1),d_2\lbrace \overline{\boldsymbol{x}}_2,d_1(\boldsymbol{x}_1)\rbrace$, $\ldots$, $d_k\lbrace \overline{\boldsymbol{x}}_k, \overline{d}_{k-1}(\overline{\boldsymbol{x}}_{k-1})\rbrace ]$, $k=3,\ldots ,K$. For each $\mathbf {d}^j$, let $C^j = I\lbrace \overline{\boldsymbol{A}}_K = \overline{d}_K^j(\overline{\boldsymbol{X}}_K)\rbrace$ be the indicator that a subject’s experience through all *K* stages is consistent with receiving treatment using $\mathbf {d}^j$. Each enrolled subject in $\mathfrak {D}_{t-1}$ was randomized at week $\tau \le t-1$ based on $\mathfrak {D}_{\tau -1} \subseteq \mathfrak {D}_{t-1}$, thus using $r_{\tau }^j$, $j=1,\ldots ,m$. For each, define $\pi _{\tau , 1}^j(\boldsymbol{x}_1) = \eta _{\tau ,1}\lbrace d_1^j(\boldsymbol{x}_1), \boldsymbol{x}_1,\mathfrak {D}_{\tau -1}\rbrace$, $\pi _{\tau ,k}^j(\overline{\boldsymbol{x}}_k) = \eta _{\tau ,k}[d_k^j\lbrace \overline{\boldsymbol{x}}_k,\overline{d}_{k-1}^j(\overline{\boldsymbol{x}}_{k-1})\rbrace , \overline{\boldsymbol{x}}_k,\overline{d}_{k-1}^j(\overline{\boldsymbol{x}}_{k-1}),\mathfrak {D}_{\tau -1}]$, $k=2,\ldots ,K$. The weighted IPW (WIPW) estimator for $\theta ^j$ is


(1) \begin{eqnarray*}
\widehat{\theta }^{\mathrm{WIPW},j}_{t} &=& \left[\sum ^N_{i=1}\frac{ W_{\tau _i}^j\Delta _{t-1,i}C_{i}^j }{ \lbrace \prod _{k=2}^K \pi _{\tau _i,k}^j(\overline{\boldsymbol{X}}_{k,i})\rbrace \pi _{\tau _i, 1}^j(\boldsymbol{X}_{1,i}) } \right]^{-1} \\
&&\times \,\sum ^N_{i=1}\frac{W_{\tau _i}^j\Delta _{t-1,i}C_{i}^j Y_i}{ \lbrace \prod _{k=2}^K \pi _{\tau _i,k}^j(\overline{\boldsymbol{X}}_{k,i})\rbrace \pi _{\tau _i, 1}^j(\boldsymbol{X}_{1,i})},
\end{eqnarray*}

where $W_{\tau }^j$ is a weight depending on $\mathfrak {D}_{\tau -1}$ discussed further in Section 4. The denominator in each term in (1) can be interpreted as the propensity for receiving treatment consistent with $\mathbf {d}^j$ through all *K* stages and depends on $r_{\tau }^j$, $j=1,\ldots ,m$. For example, in the cancer pain SMART with $K=2$, let $X_1$ denote a patient’s baseline pain score and $X_{2,1}$ their pain score at the end of stage 1, so that $\overline{\boldsymbol{X}}_2 = (X_1,X_{2,1})^T$, and let $X_{2,2} = I(X_{2,1} \le 0.7\, X_1)$ denote their response status. For embedded regime $\mathbf {d}^1$, under which a subject will receive $A_1=0$ and then $A_2=0$ if they respond to $A_1$ and $A_2=1$ otherwise, $C^1 = I(A_1=0, X_{2,2}=1, A_2=0)+I(A_1=0,X_{2,2}=0,A_2=1)$. Because regimes 1–4 assign $A_1=0$, $\pi _{\tau , 1}^1(\boldsymbol{X}_1) = \sum ^4_{j=1} r_{\tau }^j$, and because regimes 1 and 2 assign $A_2=0$ to responders and regimes 1 and 4 assign $A_2=1$ to nonresponders, $\pi _{\tau , 2}^1(\overline{\boldsymbol{X}}_2) = \lbrace I(X_{2,2}=1)(r_{\tau }^1+r_{\tau }^2) + I(X_{2,2}=0) (r_{\tau }^1+r_{\tau }^4)\rbrace /\sum ^4_{j=1} r_{\tau }^j$.

Usual AIPW estimators incorporate baseline and interim information (eg, Tsiatis et al., 2020, Section 6.4.4) to gain efficiency over IPW estimators. Accordingly, we consider a class of weighted AIPW (WAIPW) estimators. Let $W_{\tau }^{A,j}$ be a weight depending on $\mathfrak {D}_{\tau -1}$; and $\overline{C}_{k}^j = I\lbrace \overline{\boldsymbol{A}}_k = \overline{d}_k^j(\overline{\boldsymbol{X}}_k) \rbrace$, $k=1,\ldots ,K$, with $\overline{C}_{0}^j \equiv 1$. Estimators in the class are of the form


(2) \begin{eqnarray*}
\widehat{\theta }_{t}^{\mathrm{WAIPW},j} &=& \left(\sum ^N_{i=1}W_{\tau _i}^{A,j}\Delta _{t-1,i} \right)^{-1} \sum ^N_{i=1}W_{\tau _i}^{A,j}\Delta _{t-1,i}\left(\frac{C_{i}^j Y_i}{ \left\lbrace \prod _{k=2}^K \pi _{\tau _i,k}^j(\overline{\boldsymbol{X}}_{k,i})\right\rbrace \pi _{\tau _i, 1}^j(\boldsymbol{X}_{1,i}) } \vphantom{\frac{\overline{C}_{k,i}^j}{\left\lbrace \prod _{v=2}^{k-1} \pi _{d,v,tau_i} (\overline{\boldsymbol{X}}_{v,i})\right\rbrace \pi _{d,1\tau _i}(\boldsymbol{X}_{1,i}) } }\right. \\
&&+\,\left.\sum _{k=1}^K \left[\frac{\overline{C}_{k-1,i}^j}{ \left\lbrace \prod _{v=2}^{k-1} \pi _{\tau _i,v}^j(\overline{\boldsymbol{X}}_{v,i})\right\rbrace \pi _{\tau _i,1}^j(\boldsymbol{X}_{1,i}) } - \frac{\overline{C}_{k,i}^j}{ \left\lbrace \prod _{v=2}^{k} \pi _{\tau _i,v}^j(\overline{\boldsymbol{X}}_{v,i})\right\rbrace \pi _{\tau _i,1}^j(\boldsymbol{X}_{1,i}) } \right] L^j_k(\overline{\boldsymbol{X}}_{k,i}) \right),
\end{eqnarray*}

where $L^j_k(\overline{\boldsymbol{x}}_k)$ is an arbitrary function of $\overline{\boldsymbol{x}}_k$ and $\mathbf {d}^j$, $k=1,\ldots ,K$; and $\prod _{v=1}^0 \pi _{\tau ,v}^j (\boldsymbol{x}_v) \equiv 1$.

The optimal choice is $L^j_k(\overline{\boldsymbol{x}}_k) = E\lbrace Y^{*}(\mathbf {d}^j)| \overline{\boldsymbol{X}}_k=\overline{\boldsymbol{x}}_k, \overline{\boldsymbol{A}}_{k-1} = \overline{d}_{k-1}^j(\overline{\boldsymbol{x}}_{k-1})\rbrace$, $k=1,\ldots ,K$, which can be modeled and estimated by adapting the backward iterative scheme in Tsiatis et al. (2020, Section 6.4.2). Just as the denominators of each term in (1) and (2) depend on $r_{\tau }^j$, $j=1,\ldots ,m$, based on $\mathfrak {D}_{\tau -1} \subseteq \mathfrak {D}_{t-1}$, the fitted models used to approximate the optimal $L^j_k(\overline{\boldsymbol{X}}_k)$ should be based on $\mathfrak {D}_{\tau -1}$. At stage *K*, define $Q_K(\overline{\boldsymbol{x}}_K, \overline{\boldsymbol{a}}_K) = E(Y| \overline{\boldsymbol{X}}_K=\overline{\boldsymbol{x}}_K, \overline{\boldsymbol{A}}_K=\overline{\boldsymbol{a}}_K)$ and $V_K^j(\overline{\boldsymbol{x}}_K, \overline{\boldsymbol{a}}_{K-1}) = Q_K\lbrace \overline{\boldsymbol{x}}_K, \overline{\boldsymbol{a}}_{K-1}, d_K^j(\overline{\boldsymbol{x}}_K, \overline{\boldsymbol{a}}_{K-1}) \rbrace$. Posit a model $Q_K(\overline{\boldsymbol{x}}_K, \overline{\boldsymbol{a}}_K;\boldsymbol{\beta }_K)$ for $Q_K(\overline{\boldsymbol{x}}_K, \overline{\boldsymbol{a}}_K)$ indexed by $\boldsymbol{\beta }_K$, for example, a linear or logistic model for continuous or binary *Y*, respectively. Let $\widehat{\boldsymbol{\beta }}_{K,t}$ denote an estimator for $\boldsymbol{\beta }_K$ based on subjects in $\mathfrak {D}_{t-1}$ for whom $\Delta _{t-1}=1$, for example, using least squares or maximum likelihood, and define $\widehat{Q}_{K, t}(\overline{\boldsymbol{x}}_K, \overline{\boldsymbol{a}}_K) = Q_K(\overline{\boldsymbol{x}}_K, \overline{\boldsymbol{a}}_K;\widehat{\boldsymbol{\beta }}_{K,t})$ and $\widetilde{V}_{K,t}^j(\overline{\boldsymbol{x}}_K, \overline{\boldsymbol{a}}_{K-1}) = \widehat{Q}_{K,t}\lbrace \overline{\boldsymbol{x}}_K, \overline{\boldsymbol{a}}_{K-1}, d_K^j(a_K)\rbrace$. Recursively for $k=K-1, \ldots , 1$, define $Q_k^j(\overline{\boldsymbol{x}}_k, \overline{\boldsymbol{a}}_k) = E\lbrace V_{k+1}^j(\overline{\boldsymbol{X}}_{k+1}, \overline{\boldsymbol{A}}_{k})|\overline{\boldsymbol{X}}_{k}=\overline{\boldsymbol{x}}_k, \overline{\boldsymbol{A}}_{k}=\overline{\boldsymbol{a}}_k \rbrace$ and $V_k^j(\overline{\boldsymbol{x}}_k, \overline{\boldsymbol{a}}_{k-1}) = Q_k^j\lbrace \overline{\boldsymbol{x}}_k, \overline{\boldsymbol{a}}_{k-1}, d_k^j(\overline{\boldsymbol{x}}_k, \overline{\boldsymbol{a}}_{k-1})\rbrace$. Posit a model $Q_k^j(\overline{\boldsymbol{x}}_k, \overline{\boldsymbol{a}}_k;\boldsymbol{\beta }_k^j)$ indexed by $\boldsymbol{\beta }_k^j$, and obtain estimator $\widehat{\boldsymbol{\beta }}_{k,t}^j$ based on subjects in $\mathfrak {D}_{t-1}$ for whom $\Delta _{t-1}=1$ by an appropriate regression method using as the outcome the pseudo outcomes $\widetilde{V}_{k+1,t}^j(\overline{\boldsymbol{X}}_{k+1}, \overline{\boldsymbol{A}}_{k}) = \widehat{Q}_{k+1,t}^j \lbrace \overline{\boldsymbol{X}}_{k+1}, \overline{\boldsymbol{A}}_{k}, d_{k+1}^j(\overline{\boldsymbol{X}}_{k+1}, \overline{\boldsymbol{A}}_{k}) \rbrace$, and let $\widehat{Q}_{k,t}^j(\overline{\boldsymbol{x}}_k, \overline{\boldsymbol{a}}_k)=Q_k^j(\overline{\boldsymbol{x}}_k, \overline{\boldsymbol{a}}_k;\widehat{\boldsymbol{\beta }}_{k,t}^j)$. As in Tsiatis et al. (2020, Section 6.4.2), these models may involve separate expressions for responders and nonresponders; and, if at stage *k* there is only one treatment option for a subject’s history, *Y* (if $k=K-1$) or $\widetilde{V}^j_{k+2,t}(\overline{\boldsymbol{X}}_{k+2}, \overline{\boldsymbol{A}}_{k+1})$ (if $k< K-1$) can be “carried back” in the place of $\widetilde{V}_{k+1,t}^j(\overline{\boldsymbol{X}}_{k+1}, \overline{\boldsymbol{A}}_{k})$. For each *i* with $\Delta _{t-1,i}=1$ in (2), substitute $Q_K\lbrace \overline{\boldsymbol{X}}_{K,i},\overline{d}_K^j(\overline{\boldsymbol{X}}_{K,i}); \widehat{\boldsymbol{\beta }}_{K,\tau _i}\rbrace$ and $Q^j_k\lbrace \overline{\boldsymbol{X}}_{k,i},\overline{d}_k^j(\overline{\boldsymbol{X}}_{k,i}); \widehat{\boldsymbol{\beta }}^j_{k,\tau _i}\rbrace$ for $L^j_K(\overline{\boldsymbol{X}}_{K,i})$ and $L^j_k(\overline{\boldsymbol{X}}_{k,i})$, $k=1,\ldots ,K-1$, respectively.

As the basis for RAR, we propose using (1) or (2), with or without weights, to obtain estimators $\widehat{\theta }_{t}^j$ for $\theta ^j = \mathcal {V}(\mathbf {d}^j)$, $j=1,\ldots ,m$, based on $\mathfrak {D}_{t-1}$; and take the estimated confidence distribution for $\boldsymbol{\theta }$ needed to obtain $\widehat{\rho }_{t}^j$ to form $r_{t}^j$, $j=1,\ldots ,m$, to be the asymptotic normal distribution for $\widehat{\boldsymbol{\theta }}_t=\lbrace \widehat{\theta }_{t}^1,\ldots , \widehat{\theta }_{t}^m\rbrace ^T$ following from M-estimation theory (eg, Tsiatis et al., 2020, Section 6.4.4), with the weights treated as fixed; see Web Appendix A for discussion.

Basing confidence distributions and thus randomization probabilities at week *t* on (1) or (2) uses data only on subjects in $\mathfrak {D}_{t-1}$ who have completed the trial, for whom $\Delta _{t-1}=1$. To exploit partial information on subjects still progressing through the trial at *t*, with $\Delta _{t-1}=0$, it is possible to develop a weighted version of the interim AIPW (IAIPW) estimator of Manschot et al. (2023); see Web Appendix A. Simulations in Section 5 show negligible gains in performance over (1) or (2).

### 3.2 Sequential randomization based on the optimal regime

We propose methods for obtaining randomization probabilities at week *t* based on $\mathfrak {D}_{t-1}$ to be used to assign treatments for subjects requiring randomization at *t* at any stage $k=1,\ldots ,K$. Because the set of feasible treatments $\Psi _k(\boldsymbol{h}_k)$ for a subject with history $\boldsymbol{h}_k$ at stage *k* may depend on $\boldsymbol{h}_k$, as when the sets of options for responders and nonresponders to previous treatment are different, randomization probabilities may be history dependent. The approach uses Q-learning for estimation of an optimal, individualized regime (eg, Tsiatis et al., 2020, Section 7.4.1). We present the approach when *Y* is continuous and linear models are used; extensions to other outcomes and more flexible models are possible (Moodie et al., 2014).

Define the Q-functions $Q_K(\overline{\boldsymbol{x}}_K,\overline{\boldsymbol{a}}_K) = E(Y \mid \overline{\boldsymbol{X}}_K=\overline{\boldsymbol{x}}_K, \overline{\boldsymbol{A}}_K=\overline{\boldsymbol{a}}_K)$ and, for $k=K-1,\ldots ,1$, $Q_k(\overline{\boldsymbol{x}}_k,\overline{\boldsymbol{a}}_k) = E\left\lbrace V_{k+1}(\overline{\boldsymbol{x}}_k,\boldsymbol{X}_{k+1},\overline{\boldsymbol{a}}_k) \mid \overline{\boldsymbol{X}}_k=\overline{\boldsymbol{x}}_k, \overline{\boldsymbol{A}}_k=\overline{\boldsymbol{a}}_k \right\rbrace$, where $V_k(\overline{\boldsymbol{x}}_k,\overline{\boldsymbol{a}}_{k-1}) = \max _{a_k \in \Psi _k(\overline{\boldsymbol{x}}_k,\overline{\boldsymbol{a}}_{k-1})} Q_k(\overline{\boldsymbol{x}}_k,\overline{a}_{k-1},a_k)$, $k=1,\ldots , K$. Posit models $Q_k(\overline{\boldsymbol{x}}_k,\overline{\boldsymbol{a}}_k; \boldsymbol{\beta }_k) = \boldsymbol{\phi }_k(\overline{\boldsymbol{x}}_k,\overline{\boldsymbol{a}}_k)^T \boldsymbol{\beta }_k$, where $\boldsymbol{\phi }_k(\overline{\boldsymbol{x}}_k,\overline{\boldsymbol{a}}_k)$ is a $p_k$-dimensional feature function, $k=1,\ldots ,K$. Randomization probabilities are obtained via the following backward algorithm. At stage *K*, obtain $\widehat{\boldsymbol{\beta }}_{K,t}$, the ordinary least squares (OLS) estimator based on subjects in $\mathfrak {D}_{t-1}$ for whom $\Delta _{t-1}=1$ and its estimated covariance matrix $\widehat{\boldsymbol{\Sigma }}_{K,t} = \widehat{\sigma }^2_{t,K} \big \lbrace \sum ^N_{i=1}\Delta _{t-1,i} \boldsymbol{\phi }_K(\overline{\boldsymbol{X}}_{K,i},\overline{\boldsymbol{A}}_{K,i}) \boldsymbol{\phi }_K(\overline{\boldsymbol{X}}_{K,i},\overline{\boldsymbol{A}}_{K,i})^T\big \rbrace ^{-1}$, $\widehat{\sigma }^2_{K,t} = N_t^{-1}\sum ^N_{i=1}\Delta _{t-1,i} \lbrace Y_i -\boldsymbol{\phi }_K(\overline{\boldsymbol{X}}_{K,i},\overline{\boldsymbol{A}}_{K,i})^T\widehat{\boldsymbol{\beta }}_{K,t}\rbrace ^2$, $N_t = \sum ^N_{i=1}\Delta _{t-1,i}$. Based on these results, obtain the estimated confidence distribution $\widehat{\mathcal {P}}_{\boldsymbol{\beta }_{K,t}}$ for $\boldsymbol{\beta }_K$ as described below. For $k=K-1,\ldots ,1$, define pseudo outcomes $\widetilde{V}_{k+1,t}(\boldsymbol{\beta }_{k+1}) = \max _{a_{k+1} \in \Psi (\overline{\boldsymbol{X}}_{k+1},\overline{\boldsymbol{A}}_{k})} Q_{k+1}(\overline{\boldsymbol{X}}_{k+1},\overline{\boldsymbol{A}}_{k}, a_{k+1}; \boldsymbol{\beta }_{k+1})$ for subjects in $\mathfrak {D}_{t-1}$ with $\Delta _{t-1}=1$. If a subject’s history is such that $\Psi _{k+1}(\overline{\boldsymbol{X}}_{k+1},\overline{\boldsymbol{A}}_{k})$ comprises a single treatment option, $\widetilde{V}_{k+1,t}(\boldsymbol{\beta }_{k+1})$ can be taken equal to the pseudo outcome at step $k+2$ or *Y* if $k=K-1$. Obtain an estimator for $\boldsymbol{\beta }_k$, $k = K-1,\ldots ,1$, by OLS as


(3) \begin{eqnarray*}
\widehat{\boldsymbol{\beta }}_{k,t}(\widehat{\boldsymbol{\beta }}_{k+1,t}) &=& {argmin}_{\boldsymbol{\beta }} \sum ^N_{i=1}\Delta _{t-1,i} \lbrace \widetilde{V}_{k+1,t,i}(\widehat{\boldsymbol{\beta }}_{k+1,t})\\
&& -\, \boldsymbol{\phi }_k(\overline{\boldsymbol{X}}_{k,i},\overline{\boldsymbol{A}}_{k,i})^T \boldsymbol{\beta }\rbrace ^2,
\end{eqnarray*}

and estimated covariance matrix $\widehat{\boldsymbol{\Sigma }}_{k,t}(\widehat{\boldsymbol{\beta }}_{k+1,t}) = \widehat{\sigma }^2_{k,t} \big \lbrace \sum ^N_{i=1}\Delta _{t-1,i} \boldsymbol{\phi }_k(\overline{\boldsymbol{X}}_{k,i},\overline{\boldsymbol{A}}_{k,i})\boldsymbol{\phi }_k(\overline{\boldsymbol{X}}_{k,i},\overline{\boldsymbol{A}}_{k,i})^T\big \rbrace ^{-1}$, $\widehat{\sigma }^2_{k,t}(\widehat{\boldsymbol{\beta }}_{k+1,t}) = N_{t}^{-1}\sum ^N_{i=1}\Delta _{t-1,i}\lbrace \widetilde{V}_{k+1,t,i}(\widehat{\boldsymbol{\beta }}_{k+1,t}) - \boldsymbol{\phi }_k(\overline{\boldsymbol{X}}_{k,i},\overline{\boldsymbol{A}}_{k,i})^T \widehat{\boldsymbol{\beta }}_{k,t}(\widehat{\boldsymbol{\beta }}_{k+1,t})\rbrace ^2$. Based on these results, obtain an estimated confidence distribution for $\widehat{\mathcal {P}}_{\boldsymbol{\beta }_{k,t}}$ as described next. Note that, if subjects enter the SMART by a random process, basing the fitted models on subjects in $\mathfrak {D}_{t-1}$ with $\Delta _{t-1}=1$ is reasonable, as these subjects are representative of the subject population.

Because $\widehat{\boldsymbol{\beta }}_{K,t}$ is a standard OLS estimator, it is natural to approximate the confidence distribution $\widehat{\mathcal {P}}_{\boldsymbol{\beta }_{K,t}}$ for $\boldsymbol{\beta }_K$ by $\mathcal {N}(\widehat{\boldsymbol{\beta }}_{K,t}, \widehat{\boldsymbol{\Sigma }}_{K,t})$. However, because (3) is not a standard regression problem, $\widehat{\boldsymbol{\beta }}_{k,t}(\widehat{\boldsymbol{\beta }}_{k+1,t})$, $k< K$, is a nonregular estimator (Tsiatis et al., 2020, Section 10.4.1). Thus, usual large sample theory does not apply and confidence intervals based on (unadjusted) normal or bootstrap approximations need not achieve nominal coverage. Accordingly, we obtain a confidence distribution $\widehat{\mathcal {P}}_{\boldsymbol{\beta }_{k,t}}$ for $\boldsymbol{\beta }_k$, $k=1,\ldots ,K-1$, in the spirit of a projection interval (Laber et al., 2014), which faithfully represents the uncertainty in $\widehat{\boldsymbol{\beta }}_{K-1,t},\dots ,\widehat{\boldsymbol{\beta }}_{1,t}$.

We demonstrate this approach for $K=3$. For final stage 3, draw a sample of size $B_3$, $\widetilde{\boldsymbol{\beta }}_{3,t}^1,\ldots ,\widetilde{\boldsymbol{\beta }}_{3,t}^{B_3}$, say, from $\widehat{\mathcal {P}}_{\boldsymbol{\beta }_{3,t}}$, the approximate normal sampling distribution $\mathcal {N}(\widehat{\boldsymbol{\beta }}_{3,t}, \widehat{\boldsymbol{\Sigma }}_{3,t})$ as above. At stage 2, first draw a sample $\widetilde{\boldsymbol{\beta }}_{3,t}^1,\ldots ,\widetilde{\boldsymbol{\beta }}_{3,t}^{b_3}$ of size $b_3$ from $\widehat{\mathcal {P}}_{\boldsymbol{\beta }_3,t}$. For each $\widetilde{\boldsymbol{\beta }}_{3,t}^b$, form pseudo outcomes $\widetilde{V}_{3,t,i}(\widetilde{\boldsymbol{\beta }}_{3,t}^b) = \max _{a_3 \in \Psi (\overline{\boldsymbol{X}}_{3,i},\overline{\boldsymbol{A}}_{2,i})} Q_3(\overline{\boldsymbol{X}}_{3,i},\overline{\boldsymbol{A}}_{2,i},a_3; \widetilde{\boldsymbol{\beta }}_{3,t}^b)$ and obtain $\widehat{\boldsymbol{\beta }}_{2,t}(\widetilde{\boldsymbol{\beta }}_{3,t}^b)$ by OLS analogous to (3) with $k=2$, and obtain $\widehat{\boldsymbol{\Sigma }}_{2,t}(\widetilde{\boldsymbol{\beta }}_{3,t}^b)$ similarly. Then draw a sample $\widetilde{\boldsymbol{\beta }}_{2,t}^1,\ldots ,\widetilde{\boldsymbol{\beta }}_{2,t}^{b_2}$ from $\mathcal {N}\lbrace \widehat{\boldsymbol{\beta }}_{2,t}(\widetilde{\boldsymbol{\beta }}_{3,t}^b), \widehat{\boldsymbol{\Sigma }}_{2,t}(\widetilde{\boldsymbol{\beta }}_{3,t}^b)\rbrace$. The $b_3$ samples of size $b_2$ corresponding to each $\widetilde{\boldsymbol{\beta }}_{3,t}^b$, $b=1,\ldots ,b_3$, collectively comprise a sample of size $B_2 = b_3 \times b_2$ from the confidence distribution $\widehat{\mathcal {P}}_{\boldsymbol{\beta }_{2,t}}$ for $\boldsymbol{\beta }_2$. At stage 1, again draw a sample $\widetilde{\boldsymbol{\beta }}_{3,t}^1,\ldots ,\widetilde{\boldsymbol{\beta }}_{3,t}^{b_3}$ from $\widehat{\mathcal {P}}_{\boldsymbol{\beta }_3,t}$ and obtain $\widehat{\boldsymbol{\beta }}_{2,t}(\widetilde{\boldsymbol{\beta }}_{3,t}^b)$ and $\widehat{\boldsymbol{\Sigma }}_{2,t}(\widetilde{\boldsymbol{\beta }}_{3,t}^b)$, $b=1,\ldots ,b_3$, as above. Then draw a sample $\widetilde{\boldsymbol{\beta }}_{2,t}^1,\ldots ,\widetilde{\boldsymbol{\beta }}_{2,t}^{b_2}$ from $\mathcal {N}\lbrace \widehat{\boldsymbol{\beta }}_{2,t}(\widetilde{\boldsymbol{\beta }}_{3,t}^b), \widehat{\boldsymbol{\Sigma }}_{2,t}(\widetilde{\boldsymbol{\beta }}_{3,t}^b)\rbrace$. For each of $\widetilde{\boldsymbol{\beta }}_{2,t}^b$, $b=1,\ldots ,b_2$, form pseudo outcomes $\widetilde{V}_{2,t,i}(\widetilde{\boldsymbol{\beta }}_{2,t}^b) = \max _{a_2 \in \Psi (\overline{\boldsymbol{X}}_{2,i},A_{1,i})} Q_2(\overline{\boldsymbol{X}}_{2,i},A_{1,i},a_2; \widetilde{\boldsymbol{\beta }}_{2,t}^b)$, and obtain $\widehat{\boldsymbol{\beta }}_{1,t}(\widetilde{\boldsymbol{\beta }}_{2,t}^b)$ by OLS analogous to (3) with $k=1$, and obtain $\widehat{\boldsymbol{\Sigma }}_{t,1}(\widetilde{\boldsymbol{\beta }}_{2,t}^b)$. Then draw a sample $\widetilde{\boldsymbol{\beta }}_{1,t}^1,\ldots ,\widetilde{\boldsymbol{\beta }}_{1,t}^{b_1}$ from $\mathcal {N}\lbrace \widehat{\boldsymbol{\beta }}_{1,t}(\widetilde{\boldsymbol{\beta }}_{2,t}^b), \widehat{\boldsymbol{\Sigma }}_{1,t}(\widetilde{\boldsymbol{\beta }}_{2,t}^b)\rbrace$, $b=1,\ldots ,b_2$. The $b_3 \times b_2$ samples of size $b_1$ corresponding to each combination of draws $\widetilde{\boldsymbol{\beta }}_{3,t}^b$, $\widetilde{\boldsymbol{\beta }}_{2,t}^{b^{\prime }}$, $b = 1,\ldots ,b_3$, $b^{\prime } = 1,\ldots ,b_2$, collectively comprise a sample of size $B_1 = b_3 \times b_2 \times b_1$ from the confidence distribution $\widehat{\mathcal {P}}_{\boldsymbol{\beta }_1,t}$. This procedure is embarrassingly parallel, and as it involves only draws from a multivariate normal distribution, it is not computationally burdensome.

Having generated $B_k$ draws from $\widehat{\mathcal {P}}_{\boldsymbol{\beta }_{t,k}}$, $k=1,\ldots ,K$, at week *t*, we can obtain beliefs, that is, draws from approximate posterior distributions, which can be translated into randomization probabilities for subjects requiring treatment assignments at any stage $k=1,\ldots ,K$ at week *t*. Because the *Q*-functions depend on patient history, the randomization probabilities under TS can vary across subjects at a given time even if their feasible treatments are the same. To see this, suppose that there are $s_k$ feasible sets of treatments at stage *k*, denoted by $\zeta _{k}^u = \lbrace a_{k}^{1,u},\ldots ,a_{k}^{m_{k}^u,u}\rbrace$, $u=1,\ldots , s_k$. Further, suppose that there are $\ell _{k,t}$ subjects requiring randomization at stage *k* at week *t* and that $\ell _{k,t}^u$ are eligible for the *u*th feasible set. For the *u*th feasible set, randomization probabilities can be obtained for each subject $v=1,\ldots ,\ell _{k,t}^u$ by defining the belief for $a_{k}^{j,u}$, $j=1,\ldots ,m_{k}^u$, depending on $\mathfrak {D}_{t-1}$ for subject *v* as $\widehat{\rho }_{t,v}^{j,k,u} = B_k^{-1} \sum ^{B_k}_{b=1} I\lbrace a_{k}^{j,u} = {argmax}_{a \in \zeta _{k}^u} Q_k(\overline{\boldsymbol{X}}_{k,v},\overline{\boldsymbol{A}}_{k-1,v},a; \widetilde{\boldsymbol{\beta }}_{k,t}^b)\rbrace ,$ from which randomization probabilities for subject *v* are obtained. If the Q-function model depends on the history $\boldsymbol{h}_k = (\overline{\boldsymbol{x}}_k,\overline{\boldsymbol{a}}_{k-1})$ only through the components such as previous treatment and response status that dictate the feasible set, then this approach will yield the same probabilities for all $\ell _{k,t}^u$ subjects. Otherwise, randomization probabilities will be individual-specific, depending on covariate and treatment information in addition to $\mathfrak {D}_{t-1}$, which could be logistically complex. A second approach is to consider all $(m_{k}^u)^{\ell _{k,t}^u}$ possible configurations for assigning the options in $\zeta _{k}^u$ to the $\ell _{k,t}^u$ subjects and define the beliefs and thus randomization probabilities based on the configuration that maximizes the average *k*th Q-function across subjects. Letting $\Lambda _{k}^u$ be the set of all possible configurations, writing the *j*th configuration as ${\sf a}^j = ({\sf a}_1^j,\ldots ,{\sf a}_{\ell _{k,t}^u}^j)$, define $\widehat{\rho }_{t}^{j,k,u} = B_k^{-1} \sum _{b=1}^{B_k} I\Big \lbrace {\sf a}^j = {argmax}_{{\sf a} \in \Lambda _{k}^u} (\ell _{k,t}^u)^{-1} \sum _{v=1}^{\ell _{k,t}^u} Q_k(\overline{\boldsymbol{X}}_{k,v},\overline{\boldsymbol{A}}_{k-1,v},{\sf a}; \widetilde{\boldsymbol{\beta }}_{k,t}^b)\Big \rbrace .$ Simulation experiments (not shown here) suggest that the two approaches perform similarly.

## 4 POST-TRIAL INFERENCE

Although the potential outcomes $\mathcal {W}^{*}_i$, $i=1,\ldots ,N$, are independent and identically distributed (i.i.d.), under any form of RAR, the observed data are not, as the randomization probabilities are functions of the past data $\mathfrak {D}_{t-1}$ (Zhang et al., 2021b). Thus, post-trial evaluation of $\mathcal {V}(\mathbf {d}^1),\ldots ,\mathcal {V}(\mathbf {d}^m)$ based on the usual unweighted IPW or AIPW estimators is potentially problematic, as standard asymptotic theory for these estimators, which assumes i.i.d. data, does not apply (eg, Bibaut et al., 2021; Zhang et al., 2021a). Thus, we adapt the approach of Zhang et al. (2021b) and choose the weights in (1) and (2) so that asymptotic normality for these estimators can be established via the martingale central limit theorem. We sketch the rationale here; details are given in Web Appendix B.

To emphasize the key ideas, consider a simplified setting in which a fixed number of subjects, *n*, enrolls at each week and $T \rightarrow \infty$, so that the total number of subjects $N = nT \rightarrow \infty$. To simplify notation, take $n=1$. At the end of the trial, with all data complete, reindexing subjects by $t=1,\ldots ,T$, for regime *j*, the estimators $\widehat{\theta }_{T}^{\mathrm{WIPW},j}$ and $\widehat{\theta }_{T}^{\mathrm{WAIPW},j}$, say, are solutions in $\theta ^j$ to an estimating equation  $\sum _{t=1}^T M_{t}^j (\overline{\boldsymbol{X}}_{K,t},\overline{\boldsymbol{A}}_{K,t},Y_{t}; \theta ^j) = 0$; for example, for (1),


(4) \begin{eqnarray*}
M_{t}^j (\overline{\boldsymbol{X}}_{K,t},\overline{\boldsymbol{A}}_{K,t},Y_t; \theta ^j) &=& \frac{ W_{t}^j C^j_t}{ \lbrace \prod _{k=2}^K \pi _{t,k}^j(\overline{\boldsymbol{X}}_{k,t})\rbrace \pi _{t,1}^j(\boldsymbol{X}_{1,t}) }\\
&&\times \, (Y_t - \theta ^j).
\end{eqnarray*}

Critical to the proof is that the weights $W_{t}^j$ and $W^{A,j}_{t}$ are chosen so that (1) the estimating equations remain conditionally unbiased, $E\lbrace M_{t}^j (\overline{\boldsymbol{X}}_{K,t},\overline{\boldsymbol{A}}_{K,t},Y_t; \theta ^j)| \mathfrak {D}_{t-1}\rbrace = 0$, and (2) the variance is stabilized, $E[\lbrace M_{t}^j (\overline{\boldsymbol{X}}_{K,t},\overline{\boldsymbol{A}}_{K,t},Y_t; \theta ^j)\rbrace ^2| \mathfrak {D}_{t-1}] = \sigma ^2 > 0$ for all *t*. Writing $\widehat{\theta }_{T}^j$ to denote either estimator and defining $\delta _{T}^j = T^{-1} \sum _{t=1}^T \partial /\partial \theta ^j \lbrace M_{t}^j (\overline{\boldsymbol{X}}_{K,t},\overline{\boldsymbol{A}}_{K,t},Y; \theta ^j)\rbrace _{\theta ^j=\widehat{\theta }_{T}^j}$ and $(\sigma ^{j}_T)^2 = T^{-1} \sum _{t=1}^T M_{t}^j(\overline{\boldsymbol{X}}_{K,t},\overline{\boldsymbol{A}}_{K,t},Y_t; \widehat{\theta }_{T}^j)^2$, we argue in Web Appendix B that, under standard regularity conditions, $\widehat{\theta }_{T,j} \stackrel{p}{\longrightarrow }\theta ^j$ and


(5) \begin{eqnarray*}
\delta ^j_{T} (\sigma _T^j)^{-1} T^{1/2} (\widehat{\theta }_{T}^j - \theta ^j) \stackrel{D}{\longrightarrow }\mathcal {N}(0,1).
\end{eqnarray*}

In Section 5, we use (5) to construct confidence intervals and bounds for $\theta ^j$, $j=1,\ldots ,m$.

We sketch arguments for the WIPW estimator to show that (1) holds and how to choose the weights to guarantee (2); see Web Appendix B for details and arguments for the WAIPW estimator. Define $\overline{\boldsymbol{X}}_k^{*}(\mathbf {d}^j) = \lbrace \boldsymbol{X}_1, \boldsymbol{X}_2^{*}(\mathbf {d}^j), \ldots , \boldsymbol{X}_k^{*}(\mathbf {d}^j)\rbrace$, $k=1,\ldots ,K$, and write $\pi _t^{*,j}\lbrace \overline{\boldsymbol{X}}_K^{*}(\mathbf {d}^j) \rbrace = \prod _{k=2}^K\pi _{t,k}^j\lbrace \overline{\boldsymbol{X}}_k^{*}(\mathbf {d}^j)\rbrace \pi _{t,1}^j(\boldsymbol{X}_1)$. When $C^j_t=1$, $Y_t = Y^{*}(\mathbf {d}^j)$, $\overline{\boldsymbol{X}}_{K,t} = \overline{\boldsymbol{X}}_K^{*}(\mathbf {d}^j)$, so that (1) is


\begin{eqnarray*}
E\lbrace M_{t}^j (\overline{\boldsymbol{X}}_{K,t},\overline{\boldsymbol{A}}_{K,t},Y_t; \theta ^j)| \mathfrak {D}_{t-1}\rbrace &=& W_{t}^j E \left[\left. \frac{ C^j_t}{ \pi _t^{*,j}\lbrace \overline{\boldsymbol{X}}_K^{*}(\mathbf {d}^j) \rbrace } \lbrace Y^{*}(\mathbf {d}^j) - \theta ^j\rbrace \right| \mathfrak {D}_{t-1}\right] \\
&=& W_{t}^j E \left[\left. \frac{ E(C^j_t| \mathcal {W}^{*}, \mathfrak {D}_{t-1})}{ \pi _t^{*,j}\lbrace \overline{\boldsymbol{X}}_K^{*}(\mathbf {d}^j) \rbrace } \lbrace Y^{*}(\mathbf {d}^j) - \theta ^j\rbrace \right| \mathfrak {D}_{t-1}\right] = W_{t}^j E\lbrace Y^{*}(\mathbf {d}^j) - \theta ^j \rbrace = 0,
\end{eqnarray*}

because, as in Tsiatis et al. (2020, Section 6.4.3), $E(C^j_t| \mathcal {W}^{*}, \mathfrak {D}_{t-1}) = \pi _\tau ^{*,j}\lbrace \overline{\boldsymbol{X}}_K^{*}(\mathbf {d}^j)\rbrace$, and $\mathcal {W}^{*}$ and thus $Y^{*}(\mathbf {d}^j)$ is independent of $\mathfrak {D}_{t-1}$. Using similar manipulations,


(6) \begin{eqnarray*}
E\lbrace M_{t}^j (\overline{\boldsymbol{X}}_{K,t},\overline{\boldsymbol{A}}_{K,t},Y_t; \theta ^j)^2| \mathfrak {D}_{t-1}\rbrace \\
\quad = (W_{t}^j)^2 E\left[\left. \frac{ \lbrace Y^{*}(\mathbf {d}^j) - \theta ^j \rbrace ^2 }{\pi _t^{*,j}\lbrace \overline{\boldsymbol{X}}_K^{*}(\mathbf {d}^j) \rbrace } \right| \mathfrak {D}_{t-1} \right].
\end{eqnarray*}

Thus, to ensure (2), $W_{t}^j$ should be chosen so that (6) is a constant depending only on *j*.

We demonstrate the choice of $W_{t}^j$ in practice for regime 1 of the cancer pain SMART, $K=2$, and up-front randomization. From Section 3.1, letting $X^{*}_{2,2}(\mathbf {d}^1) = I\lbrace X^{*}_{2,1}(\mathbf {d}^1) \le 0.7\, X_1\rbrace$, $\pi _{t, 1}^1(\boldsymbol{X}_1) = \pi _{t,1}^1 = \sum ^4_{j=1} r_{t}^j$, and $\pi _{t, 2}^1\lbrace \overline{\boldsymbol{X}}^{*}_2(\mathbf {d}^1)\rbrace = I\lbrace X_{2,2}^{*}(\mathbf {d}^1) = 1\rbrace \pi ^{1(1)}_{t,2} + I\lbrace X_{2,2}^{*}(\mathbf {d}^1) = 0\rbrace \pi ^{1(0)}_{t,2}$, where $\pi ^{1 (1)}_{t,2}= (r_{t}^1+r_{t}^2)/\pi _{t,1}^1$ and $\pi ^{1 (0)}_{t,2}(r_{t}^1+r_{t}^4)/\pi _{t,1}^1$. Then, using $\mathcal {W}^{*}\perp\!\!\!\perp\mathfrak {D}_{t-1}$, (6) is


(7) \begin{eqnarray*}
&&(W_{t,1})^2 \left(\frac{ \mu ^{1(1)}}{\pi ^{1 (1)}_{t,2}\pi _{t, 1}^1} + \frac{ \mu ^{(0)}_1}{\pi ^{1 (0)}_{t,2}\pi _{t, 1}^1} \right), \, \, \, \mu ^{1(s)} = E[I\lbrace X_{2,2}^{*}(\mathbf {d}^1) \\
&&\quad= s\rbrace \lbrace Y^{*}(\mathbf {d}^1) - \theta ^1\rbrace ^2], \, s = 0, 1.
\end{eqnarray*}

Then, in the original notation, estimate $\mu ^{1(s)}_1$ at week *t* by $\widehat{\mu }^{1(s)}_{t} = N^{-1}_t \sum ^N_{i=1}\big [\Delta _{t-1,i}I(X_{2,2,i}=s) C^1_i (Y_i-\widetilde{\theta }_{t}^1)^2/\lbrace \pi ^{1 (s)}_{\tau _i,2}\pi _{\tau _i, 1}^1\rbrace \big ]$, $s=0,1$, based on $\mathfrak {D}_{t-1}$, where $\widetilde{\theta }_{t}^1$ is an estimator for $\theta ^1$ using $\mathfrak {D}_{t-1}$ (we use the unweighted IPW estimator). Setting (7) equal to a constant $\Xi ^1$, say, and defining $\widehat{\Xi }_{t}^1 = \sum _{s=0}^1 \lbrace \widehat{\mu }^{1(s)}_{t}/(\pi ^{1(s)}_{t,2}\pi _{t, 1}^1) \rbrace$, estimate $\Xi ^1$ based on the burn-in data, for which $W_{t}^j \equiv 1$ and $r_{t}^j$, $j=1,\ldots ,8$, are fixed (nonadaptive) for $t \le t^{*}$, by $\widehat{\Xi }_{t^{*}}^1$. Then for $t \ge t^{*}+1$, take $W_{t}^1 = (\widehat{\Xi }^1_{t^{*}}/\widehat{\Xi }^1_{t})^{1/2}$. See Web Appendix B for considerations for sequential RAR.

## 5 SIMULATION STUDIES

### 5.1 Up-front randomization among embedded regimes

We present results of simulation studies involving 5000 Monte Carlo trials under a scenario mimicking that of the cancer pain SMART introduced in Section 1 in which subjects are randomized using various forms of up-front RAR as in Section 3.1. From Zhang et al. (2021a,b), the extent to which standard unweighted estimators fail to be asymptotically normal under RAR likely depends on whether or not some or all of the true values of the embedded regimes are equal or at least very similar, with the null situation with all values the same particularly problematic (eg, Hadad et al., 2021). Thus, this scenario involves subsets of regime values that differ negligibly, which is common in practice and qualitatively similar to the configuration found by Somers et al. (2023). Additional studies under null and close-to-null scenarios and different designs and binary outcome are in Web Appendix C.

Each trial enrolls *N* subjects at times selected uniformly over integer weeks 1-24. At enrollment, we draw baseline pain score $X_1 \sim \mathcal {N}(5,1)$ and assign stage 1 treatment $A_1 \in \mathcal {A}_1=\lbrace 0,1\rbrace$. Six weeks later, second-stage pain score is generated as $X_{2,1} = \gamma _{1,0} + \gamma _{1,1} X_1 + \gamma _{1,2} A_1 + \varepsilon _1$, $\varepsilon _1 \sim \mathcal {N}(0,1)$; and response status is $X_{2,2}=I(X_{2,1}< 0.7X_1)$, which, with $A_1$, dictates the feasible subset of $\mathcal {A}_2 = \lbrace 0,1,2,3,4,5\rbrace$ from which stage 2 treatment $A_2$ is assigned. Six weeks later, we generate outcome $Y=\gamma _{2,0} + \gamma _{2,1} X_1 + \gamma _{2,2} A_1 + \gamma _{2,3} X_{2,1} + \gamma _{2,4}I(A_2=1) + \gamma _{2,5}I(A_2=2 { or } 5) + \gamma _{2,6}I(A_2=3) + \gamma _{2,7}I(A_2=4) + \varepsilon _2$, $\varepsilon _2 \sim \mathcal {N}(0,1)$. With $\boldsymbol{\gamma }_1 = (\gamma _{1,0},\gamma _{1,1},\gamma _{1,2})^T = (0.00, 0.90, -1.50)^T$ and $\boldsymbol{\gamma }_2 = (\gamma _{2,0},\ldots ,\gamma _{2,7}) = (0.00,0.30,-0.75,0.60,-0.25,-0.75,-0.75,-0.85)^T$, for the $m=8$ embedded regimes in Figure 1, the values $\lbrace \mathcal {V}(\mathbf {d}^1),\ldots ,\mathcal {V}(\mathbf {d}^8)\rbrace = (\theta _1,\ldots ,\theta _8) = \boldsymbol{\theta}$ = (−0.126, −0.374, −0.500, −0.251, −2.408, −2.401, −2.494, $-2.501)$. As larger reductions in pain are favored, regimes 1-4, which assign treatment 0 at stage 1, are inferior to regimes 5-8, which assign treatment 1. Thus, we say that treatment 1 is the optimal stage 1 treatment. Regime 8 is optimal but negligibly different in value from regime 7, and the values of regimes 5 and 6 are trivially different; thus, practically speaking, either of regimes 7 or 8 is optimal. The burn-in period ends at the time $t^{*}$ when each of the $m=8$ regimes has at least 25 subjects who have completed the trial with experience consistent with following the regime.

We implement up-front RAR using TS with $c_t = 0.25, 0.5, 0.75$, and 1 for all *t* based on the WIPW and WAIPW estimators (1) and (2), the unweighted IAIPW estimator, and the unweighted IPW and AIPW estimators. For each, at any *t* we impose clipping constants of 0.05 and 0.95, so if $r_{t}^j< 0.05$ for any $j=1,\ldots ,8$, set $r_{t}^j=0.05$ (and likewise for 0.95), and then normalize $r_{t}^j$, $j=1,\ldots ,8$, to sum to one. In the WAIPW and IAIPW estimators, $Q_{2}(\overline{\boldsymbol{x}}_2,\overline{\boldsymbol{a}}_2;\boldsymbol{\beta }_2) = \beta _{2,0} + \beta _{2,1}x_1 + \beta _{2,2}I(a_1=1) + \beta _{2,3}x_{2,1} + \beta _{2,4}I(a_2=1) + \beta _{2,5}I(a_2=2 { or } 5) + \beta _{2,6}I(a_2=3) + \beta _{2,7}I(a_2=4)$ and $Q_{1}^j(x_1,a_1;\boldsymbol{\beta }_{1}^j) = \beta _{1,0}^j + \beta _{1,1}^j x_1 + \beta _{1,2}^ja_1 + \beta _{1,3}^j x_1a_1$.

Table 1 presents results for $N=1000$ for up-front RAR using the WIPW, WAIPW, and unweighted IAIPW estimators with $c_t = 0.5$ and 1, representing moderate and aggressive adaptation, and for simple, uniform randomization (SR) among the 8 embedded regimes; results for $N = 325$, similar to the sample size in the cancer pain SMART, are in Table A.1 of Web Appendix A. Additional results for $N = 325$ and 1000 using these estimators with $c_t = 0.25$ and 0.75 and for all $c_t$ values for RAR using the unweighted IPW and AIPW estimators are in Web Appendix C. To evaluate how up-front RAR improves in-trial outcomes, we report the Monte Carlo average outcome across subjects in the trial; average proportion of subjects assigned $A_1=1$, the optimal first-stage treatment; and average proportion of subjects in the trial who had treatment experience consistent with following the optimal regime 8 and with either of regimes 7 or 8. All are improved using RAR over SR, resulting in lower average outcome and higher rates of assigning optimal stage 1 treatment and optimal regime. More aggressive adaptation, $c_t=1$, yields more favorable results than $c_t=0.5$ using any estimator; from Web Appendix C, results using $c_t=0.25$ (0.75) are less favorable (intermediate). Results are more favorable for WAIPW- and IAIPW-based randomization, as those estimators exploit covariate information, with the gains mostly at stage 2. Using weighted vs. unweighted estimators yields modest in-trial gains; see Web Appendix C.

**TABLE 1:** Simulation results using up-front RAR based on TS for 5000 Monte Carlo (MC) replications for the scenario in Section 5.1, $N = 1000$.

	SR	WIPW(0.5)	WIPW(1)	WAIPW(0.5)	WAIPW(1)	IAIPW(0.5)	IAIPW(1)
In Trial							
Mean *Y*	−1.380 (0.001)	−1.795 (0.001)	−1.992 (0.001)	−1.794 (0.001)	**−1.996** (0.001)	−1.793 (0.001)	−1.993 (0.001)
Proportion $A_1$ Opt	0.500 (0.000)	0.691 (0.000)	**0.782** (0.000)	0.691 (0.000)	**0.782** (0.000)	0.690 (0.000)	**0.782** (0.000)
Proportion Regime Opt	0.250 (0.000)	0.390 (0.001)	0.470 (0.001)	0.401 (0.001)	**0.498** (0.002)	0.402 (0.001)	0.491 (0.001)
Estimation							
$\mathbf {d}^8$ Est Opt (IPW)	0.433 (0.007)	**0.462** (0.007)	0.444 (0.007)	0.449 (0.007)	0.409 (0.007)	0.441 (0.007)	0.430 (0.007)
$\mathbf {d}^8$ Est Opt (WIPW)	0.397 (0.007)	**0.468** (0.007)	0.460 (0.007)	0.463 (0.007)	0.436 (0.007)	0.452 (0.007)	0.460 (0.007)
$\mathbf {d}^8$ Est Opt (AIPW)	0.529 (0.007)	**0.562** (0.007)	0.534 (0.007)	0.559 (0.007)	0.523 (0.007)	0.555 (0.007)	0.534 (0.007)
$\mathbf {d}^8$ Est Opt (WAIPW)	0.516 (0.007)	0.548 (0.007)	0.540 (0.007)	**0.555** (0.007)	0.524 (0.007)	0.545 (0.007)	0.539 (0.007)
$\mathbf {d}^7$ or $\mathbf {d}^8$ Est Opt (IPW)	0.778 (0.006)	**0.812** (0.006)	0.782 (0.006)	0.797 (0.006)	0.749 (0.006)	0.793 (0.006)	0.757 (0.006)
$\mathbf {d}^7$ or $\mathbf {d}^8$ Est Opt (WIPW)	0.736 (0.006)	**0.815** (0.005)	0.812 (0.006)	0.808 (0.006)	0.796 (0.006)	0.806 (0.006)	0.803 (0.006)
$\mathbf {d}^7$ or $\mathbf {d}^8$ Est Opt (AIPW)	0.857 (0.005)	**0.885** (0.005)	0.856 (0.005)	0.887 (0.004)	0.861 (0.005)	0.876 (0.005)	0.859 (0.005)
$\mathbf {d}^7$ or $\mathbf {d}^8$ Est Opt (WAIPW)	0.854 (0.005)	0.887 (0.004)	0.882 (0.005)	**0.889** (0.004)	0.887 (0.004)	0.878 (0.005)	0.881 (0.005)
$\mathcal {V}(\mathbf {d}^8)$ MSE $\times 10^2$ (IPW)	0.814 (0.017)	0.619 (0.013)	0.735 (0.024)	**0.591** (0.013)	0.671 (0.019)	0.619 (0.013)	0.669 (0.016)
$\mathcal {V}(\mathbf {d}^8)$ MSE $\times 10^2$ (WIPW)	1.185 (0.024)	0.598 (0.013)	0.646 (0.012)	**0.560** (0.012)	0.576 (0.015)	0.578 (0.012)	0.574 (0.014)
$\mathcal {V}(\mathbf {d}^8)$ MSE $\times 10^2$ (AIPW)	0.544 (0.011)	0.419 (0.008)	0.463 (0.012)	**0.407** (0.008)	0.446 (0.011)	0.438 (0.009)	0.432 (0.009)
$\mathcal {V}(\mathbf {d}^8)$ MSE $\times 10^2$ (WAIPW)	0.552 (0.012)	0.411 (0.008)	0.419 (0.010)	**0.400** (0.008)	0.420 (0.011)	0.427 (0.009)	0.411 (0.009)
Coverage							
$\mathcal {V}(\mathbf {d}^8)$ 95% CI (IPW)	0.949 (0.003)	0.950 (0.003)	0.949 (0.003)	0.954 (0.003)	0.947 (0.003)	0.951 (0.003)	0.950 (0.003)
$\mathcal {V}(\mathbf {d}^8)$ 95% CI (WIPW)	0.943 (0.003)	0.948 (0.003)	0.945 (0.003)	0.953 (0.003)	0.944 (0.003)	0.949 (0.003)	0.948 (0.003)
$\mathcal {V}(\mathbf {d}^8)$ 95% CI (AIPW)	0.944 (0.003)	0.952 (0.003)	0.951 (0.003)	0.952 (0.003)	0.954 (0.003)	0.950 (0.003)	0.950 (0.003)
$\mathcal {V}(\mathbf {d}^8)$ 95% CI (WAIPW)	0.947 (0.003)	0.951 (0.003)	0.950 (0.003)	0.951 (0.003)	0.948 (0.003)	0.947 (0.003)	0.944 (0.003)
$\mathcal {V}(\mathbf {d}^8)$ 95% LB (IPW)	0.952 (0.003)	0.960 (0.003)	0.960 (0.003)	0.954 (0.003)	0.954 (0.003)	0.950 (0.003)	0.953 (0.003)
$\mathcal {V}(\mathbf {d}^8)$ 95% LB (WIPW)	0.946 (0.003)	0.954 (0.003)	0.948 (0.003)	0.950 (0.003)	0.947 (0.003)	0.947 (0.003)	0.945 (0.003)
$\mathcal {V}(\mathbf {d}^8)$ 95% LB (AIPW)	0.944 (0.003)	0.959 (0.003)	0.955 (0.003)	0.951 (0.003)	0.954 (0.003)	0.950 (0.003)	0.952 (0.003)
$\mathcal {V}(\mathbf {d}^8)$ 95% LB (WAIPW)	0.946 (0.003)	0.957 (0.003)	0.950 (0.003)	0.947 (0.003)	0.947 (0.003)	0.944 (0.003)	0.945 (0.003)
$\mathcal {V}(\mathbf {d}^8)$ 95% UB (IPW)	0.950 (0.003)	0.944 (0.003)	0.940 (0.003)	0.955 (0.003)	0.942 (0.003)	0.949 (0.003)	0.944 (0.003)
$\mathcal {V}(\mathbf {d}^8)$ 95% UB (WIPW)	0.946 (0.003)	0.947 (0.003)	0.944 (0.003)	0.955 (0.003)	0.946 (0.003)	0.952 (0.003)	0.947 (0.003)
$\mathcal {V}(\mathbf {d}^8)$ 95% UB (AIPW)	0.952 (0.003)	0.947 (0.003)	0.948 (0.003)	0.954 (0.003)	0.949 (0.003)	0.944 (0.003)	0.948 (0.003)
$\mathcal {V}(\mathbf {d}^8)$ 95% UB (WAIPW)	0.952 (0.003)	0.946 (0.003)	0.952 (0.003)	0.956 (0.003)	0.949 (0.003)	0.948 (0.003)	0.950 (0.003)

Columns indicate the randomization method: WAIPW(0.5) is TS based on the WAIPW estimator (2) with $c_t=0.5$ for all *t* and AIPW(1) uses $c_t=1$ for all *t*; WIPW($c_t$) and IAIPW($c_t$) are defined similarly. SR denotes simple, uniform randomization. Mean *Y* denotes the MC average mean outcome for the 1000 individuals in the trial; lower mean outcomes are more favorable. Proportion $A_1$ Opt is the MC average proportion of subjects assigned the optimal treatment at the first stage; Proportion Regime Opt is the MC average proportion of subjects who were consistent with following the optimal regime $\mathbf {d}^8$ in the trial. For estimation results, $\mathbf {d}^8$ Est Opt denotes the proportion of trials we correctly estimate $\mathbf {d}^8$ to be the optimal regime; $\mathbf {d}^7$ or $\mathbf {d}^8$ Est Opt is the proportion of trials we estimate either regime 7 or 8 to be the optimal regime; and $\mathcal {V}(\mathbf {d}^8)$ MSE is the MC mean squared error for regime 8. For the estimation results, the term in parentheses, for example, (IPW), denotes the estimator used after the trial is completed. $\mathcal {V}(\mathbf {d}^8)$ 95% CI is the MC proportion of 95% confidence intervals that cover the true value; the term in parentheses is the estimator used to construct the confidence interval. $\mathcal {V}(\mathbf {d}^8)$ 95% LB for lower confidence bounds $\mathcal {V}(\mathbf {d}^8)$ 95% UB for upper confidence bounds are defined similarly. Bold values indicate the most favorable result among the randomization methods. Standard deviations of entries are in parentheses.

**FIGURE 2:**
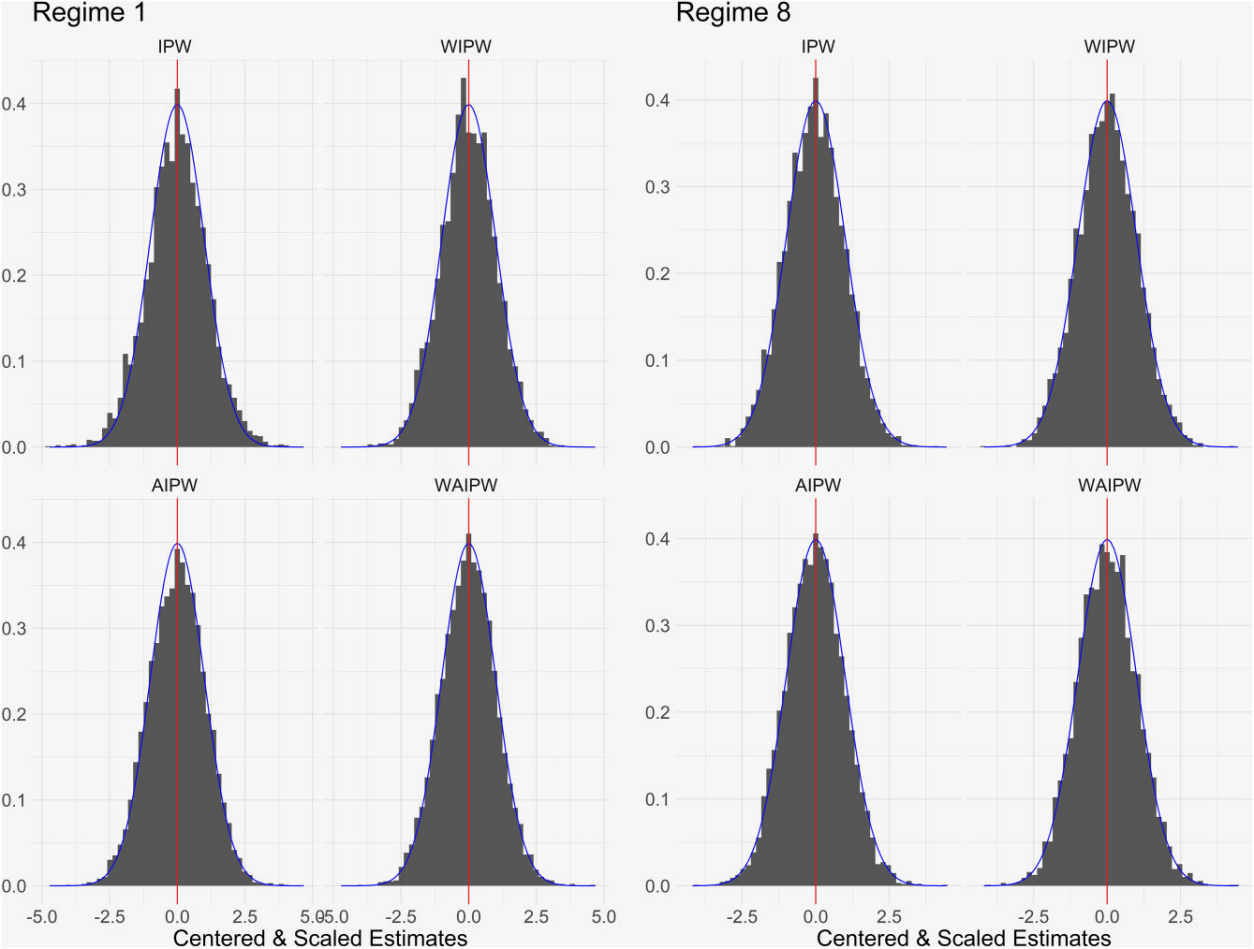
Monte Carlo distributions of centered and scaled estimates as in the theory of Section 4 for selected estimators in the simulation in Section 5.1, $N=1000$. The histograms correspond to the indicated estimator for $\mathcal {V}(\mathbf {d}^1)$ and $\mathcal {V}(\mathbf {d}^8)$ under up-front randomization using TS based on the WAIPW estimator (2) with $c_t=1$ for all *t*. The vertical line indicates mean zero, and the density of a standard normal distribution is superimposed.

Reflecting post-trial performance, Table 1 shows for $N=1000$ the proportion of trials where $\mathbf {d}^8$ alone or either of $\mathbf {d}^7$ or $\mathbf {d}^8$ is identified as optimal and the mean-squared error (MSE) for $\widehat{\theta }^8$ based on estimation of $\boldsymbol{\theta }$ using the IPW, AIPW, WIPW, and WAIPW estimators with final data at the end of the trial; analogous results for $N=325$ are in Web Appendix A. Relative to SR, using RAR based on any of the estimators generally identifies optimal regimes at higher rates. Performance of 95% confidence intervals and lower/upper confidence bounds for the true value based on the asymptotic theory in Section 4 is presented for $\mathcal {V}(\mathbf {d}^8)$; that for other regimes is similar, see Web Appendix C. Regardless of randomization scheme, the nominal level is achieved in almost every case. Figure 2 shows the distribution of the 5000 centered and scaled final value estimates $\widehat{\theta }^j$, $j=1, 8$, obtained using the IPW, AIPW, WIPW, and WAIPW estimators based on RAR using the WAIPW estimator with $c_t=1$, $N = 1000$; those for other schemes are similar. As found by Zhang et al. (2021b) and others, unweighted estimators result in mildly skewed distributions, while weighted estimators yield approximate standard normality. Similar observations hold over all simulation scenarios we have tried, with both continuous and binary outcomes; see Web Appendix C.

### 5.2 Sequential randomization at each stage based on optimal regimes

We report on simulation studies involving 5000 Monte Carlo trials under the scenario in Section 5.1 to compare the Q-learning-based sequential RAR approach using TS in Section 3.2 with $c_t = 0.25, 0.50, 0.75$ and 1 to SR and to a conservatively tuned (AR-1) and more aggressively tuned (AR-2) version of the SMART-AR method of Cheung et al. (2014); see Web Appendix A for details. Results for additional scenarios are in Web Appendix C. To implement all RAR methods, we posit linear models $Q_{2}(\overline{\boldsymbol{x}}_2,\overline{\boldsymbol{a}}_2;\boldsymbol{\beta }_2) = \beta _{2,0} + \beta _{2,1}x_1 + \beta _{2,2}I(a_1=1) + \beta _{2,3}x_{2,1} + \beta _{2,4}I(a_2=1) + \beta _{2,5}I(a_2=2 { or } 5) + \beta _{2,6}I(a_2=3) + \beta _{2,7}I(a_2=4)$ and $Q_{1}(x_1,a_1;\boldsymbol{\beta }_{1}) = \beta _{1,0} + \beta _{1,1} x_1 + \beta _{1,2}a_1$. For sequential RAR, we set $B_1 = b_2 \times b_1 = 32 \times 32 = 1024$ and $B_2 = 1000$. Clipping constants of 0.05 and 0.95 were imposed on all methods.

At each week, for each RAR method, newly enrolled subjects are assigned stage 1 treatment using the same randomization probability. Already-enrolled subjects who have reached stage 2 at this week and require stage 2 randomization are partitioned into 4 groups based on $(a_1, x_{2,2}) = (0,0), (0,1), (1,0), (1,1)$. Within each group, randomization probabilities are calculated; thus, second-stage probabilities are specific to each stage 1 treatment-response status combination. The burn-in period ends at the time $t^{*}$ when at least 25 subjects have completed the trial with experience consistent with each of the $m=8$ embedded regimes.

Table 2 presents the results for $N=1000$; those for $N=325$ are in Table A.2 of Web Appendix A. As in Section 5.1, the sequential RAR method results in improved (over SR) in-trial outcomes on average by assigning optimal treatments and regimes at higher rates. The AR methods also improve on SR but are relatively conservative. For post-trial estimation, less-aggressive RAR identifies the optimal regime at higher rates than SR. The proposed method with $c_t=1$ and 0.75 yields the best post-trial performance using any of the IPW, WIPW, AIPW, or WAIPW estimators but lower rate of identifying the optimal regime. As expected, the AIPW and WAIPW estimators are more efficient than the IPW and WIPW estimators. While the primary goal of the weighted estimators is to attain nominal coverage, an additional benefit is higher rates of identifying the optimal regime.

**TABLE 2:** Simulation results using sequential RAR based on TS for 5000 Monte Carlo replications for the scenario in Section 5.1, $N = 1000$.

	SR	TS(0.25)	TS(0.50)	TS(0.75)	TS(1)	AR-1	AR-2
In Trial							
Mean *Y*	−1.380 (0.001)	−1.976 (0.001)	−1.999 (0.001)	−2.014 (0.001)	**−2.206** (0.001)	−1.950 (0.001)	−1.957 (0.001)
Proportion $A_1$ Opt	0.500 (0.000)	0.772 (0.001)	0.780 (0.001)	0.785 (0.001)	**0.790** (0.000)	0.775 (0.000)	0.775 (0.000)
Proportion Regime Opt	0.250 (0.000)	0.445 (0.001)	0.491 (0.001)	0.517 (0.001)	**0.538** (0.002)	0.320 (0.001)	0.351 (0.001)
Estimation							
$\mathbf {d}^8$ Est Opt (IPW)	0.433 (0.007)	0.471 (0.007)	0.463 (0.007)	0.423 (0.007)	0.404 (0.007)	0.468 (0.007)	**0.484** (0.007)
$\mathbf {d}^8$ Est Opt (WIPW)	0.397 (0.007)	0.480 (0.007)	0.471 (0.007)	0.452 (0.007)	0.434 (0.007)	0.480 (0.007)	**0.488** (0.007)
$\mathbf {d}^8$ Est Opt (AIPW)	0.529 (0.007)	0.568 (0.007)	0.549 (0.007)	0.512 (0.007)	0.484 (0.007)	0.546 (0.007)	**0.569** (0.007)
$\mathbf {d}^8$ Est Opt (WAIPW)	0.516 (0.007)	0.569 (0.007)	0.548 (0.007)	0.530 (0.007)	0.519 (0.007)	0.558 (0.007)	**0.582** (0.007)
$\mathbf {d}^7$ or $\mathbf {d}^8$ Est Opt (IPW)	0.778 (0.006)	0.813 (0.006)	0.792 (0.006)	0.750 (0.006)	0.724 (0.006)	0.810 (0.006)	**0.822** (0.005)
$\mathbf {d}^7$ or $\mathbf {d}^8$ Est Opt (WIPW)	0.736 (0.006)	0.827 (0.005)	0.814 (0.006)	0.793 (0.006)	0.784 (0.006)	0.820 (0.005)	**0.830** (0.005)
$\mathbf {d}^7$ or $\mathbf {d}^8$ Est Opt (AIPW)	0.857 (0.005)	0.888 (0.005)	0.876 (0.005)	0.840 (0.005)	0.799 (0.006)	0.890 (0.004)	**0.904** (0.004)
$\mathbf {d}^7$ or $\mathbf {d}^8$ Est Opt (WAIPW)	0.854 (0.005)	0.897 (0.004)	0.889 (0.004)	0.873 (0.005)	0.860 (0.005)	0.897 (0.004)	**0.912** (0.004)
$\mathcal {V}(\mathbf {d}^8)$ MSE $\times 10^2$ (IPW)	0.814 (0.017)	0.556 (0.012)	**0.541** (0.012)	0.586 (0.015)	0.660 (0.020)	0.837 (0.018)	0.676 (0.014)
$\mathcal {V}(\mathbf {d}^8)$ MSE $\times 10^2$ (WIPW)	1.185 (0.024)	0.504 (0.011)	**0.480** (0.011)	0.515 (0.014)	0.517 (0.014)	0.767 (0.016)	0.636 (0.013)
$\mathcal {V}(\mathbf {d}^8)$ MSE $\times 10^2$ (AIPW)	0.544 (0.011)	0.396 (0.009)	**0.383** (0.008)	0.401 (0.010)	0.433 (0.013)	0.521 (0.011)	0.442 (0.009)
$\mathcal {V}(\mathbf {d}^8)$ MSE $\times 10^2$ (WAIPW)	0.552 (0.012)	0.377 (0.008)	**0.368** (0.008)	0.381 (0.010)	0.378 (0.010)	0.501 (0.010)	0.432 (0.009)
Coverage							
$\mathcal {V}(\mathbf {d}^8)$ 95% CI (IPW)	0.949 (0.003)	0.940 (0.003)	0.951 (0.003)	0.953 (0.003)	0.954 (0.003)	0.949 (0.003)	0.948 (0.003)
$\mathcal {V}(\mathbf {d}^8)$ 95% CI (WIPW)	0.943 (0.003)	0.940 (0.003)	0.951 (0.003)	0.952 (0.003)	0.954 (0.003)	0.949 (0.003)	0.947 (0.003)
$\mathcal {V}(\mathbf {d}^8)$ 95% CI (AIPW)	0.944 (0.003)	0.947 (0.003)	0.950 (0.003)	0.958 (0.003)	0.954 (0.003)	0.950 (0.003)	0.952 (0.003)
$\mathcal {V}(\mathbf {d}^8)$ 95% CI (WAIPW)	0.947 (0.003)	0.942 (0.003)	0.947 (0.003)	0.951 (0.003)	0.952 (0.003)	0.948 (0.003)	0.951 (0.003)
$\mathcal {V}(\mathbf {d}^8)$ 95% LB (IPW)	0.952 (0.003)	0.948 (0.003)	0.952 (0.003)	0.955 (0.003)	0.960 (0.003)	0.940 (0.003)	0.948 (0.003)
$\mathcal {V}(\mathbf {d}^8)$ 95% LB (WIPW)	0.946 (0.003)	0.946 (0.003)	0.948 (0.003)	0.950 (0.003)	0.954 (0.003)	0.946 (0.003)	0.949 (0.003)
$\mathcal {V}(\mathbf {d}^8)$ 95% LB (AIPW)	0.944 (0.003)	0.948 (0.003)	0.948 (0.003)	0.955 (0.003)	0.957 (0.003)	0.944 (0.003)	0.949 (0.003)
$\mathcal {V}(\mathbf {d}^8)$ 95% LB (WAIPW)	0.946 (0.003)	0.946 (0.003)	0.945 (0.003)	0.947 (0.003)	0.950 (0.003)	0.946 (0.003)	0.951 (0.003)
$\mathcal {V}(\mathbf {d}^8)$ 95% UB (IPW)	0.950 (0.003)	0.945 (0.003)	0.950 (0.003)	0.950 (0.003)	0.946 (0.003)	0.952 (0.003)	0.950 (0.003)
$\mathcal {V}(\mathbf {d}^8)$ 95% UB (WIPW)	0.946 (0.003)	0.947 (0.003)	0.951 (0.003)	0.951 (0.003)	0.952 (0.003)	0.949 (0.003)	0.948 (0.003)
$\mathcal {V}(\mathbf {d}^8)$ 95% UB (AIPW)	0.952 (0.003)	0.946 (0.003)	0.951 (0.003)	0.952 (0.003)	0.952 (0.003)	0.954 (0.003)	0.951 (0.003)
$\mathcal {V}(\mathbf {d}^8)$ 95% UB (WAIPW)	0.952 (0.003)	0.949 (0.003)	0.953 (0.003)	0.957 (0.003)	0.956 (0.003)	0.952 (0.003)	0.947 (0.003)

Columns indicate the randomization method: TS(0.25) is TS via Q-learning with $c_t=0.25$ for all *t*, TS(0.50), TS(0.75), and TS(1) are defined similarly. AR-1 is the conservatively tuned version of SMART-AR and AR-2 is the more aggressive version. SR denotes simple, uniform randomization. All entries are defined as in Table 1.

**FIGURE 3:**
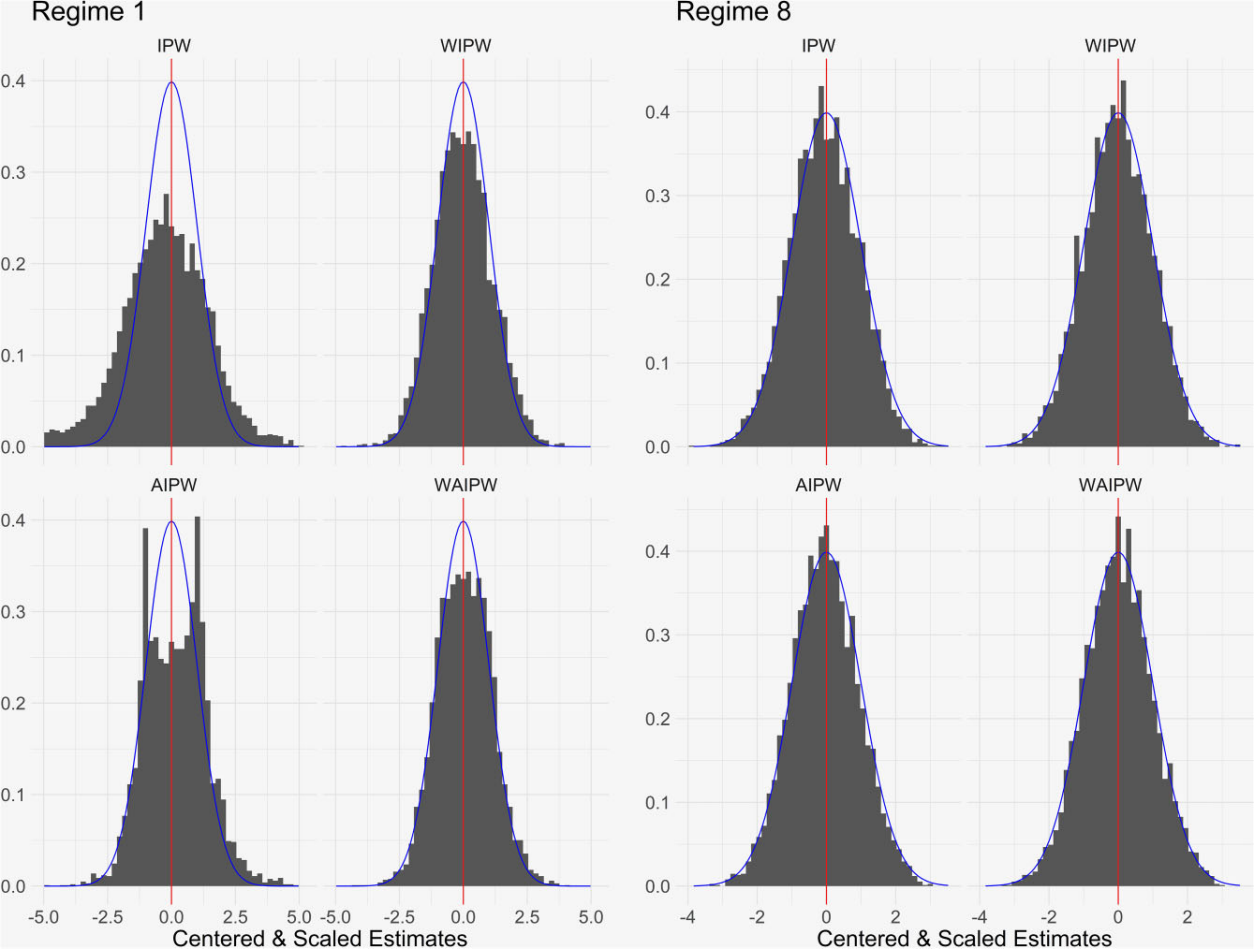
Monte Carlo distributions of centered and scaled estimates as in the theory of Section 4 for selected estimators in the simulation in Section 5.2, $N = 1000$. The histograms correspond to the indicated estimator for $\mathcal {V}(\mathbf {d}^1)$ and $\mathcal {V}(\mathbf {d}^8)$ under sequential randomization using TS with $c_t=1$ for all *t*. The vertical line indicates mean zero, and the density of a standard normal distribution is superimposed.

Figure 3 shows the distributions of the 5000 centered and scaled final value estimates $\widehat{\theta }^j$, $j=1, 8$, based on sequential RAR using TS with $c_t=1$ with $N=1000$ (plots for other methods and regimes are similar). For $\mathcal {V}(\mathbf {d}^8)$, the weighted estimators are approximately normal while the unweighted estimators are slightly left skewed; for $\mathcal {V}(\mathbf {d}^1)$, the unweighted estimators are nonnormal, and the weighted estimators are improved. We attribute this behavior to undersampling of $\mathbf {d}^1$, the least effective regime; the issue is less pronounced for less aggressive randomization. Coverage of confidence intervals and bounds for $\mathcal {V}(\mathbf {d}^8)$ is mildly improved for the weighted estimators.

## 6 DISCUSSION

We have proposed methods for RAR in SMARTs using TS, where randomization can be up-front to embedded regimes or performed sequentially at each stage. Simulation studies demonstrate the benefits over nonadaptive randomization: improved outcomes for subjects in the trial, improved ability to identify an optimal regime, and little or no effect on post-trial inference on embedded regimes. Choice of damping constant can dramatically affect the aggressiveness of TS; thus, the specific features and goals of a SMART should be considered when choosing this parameter. When randomization is up-front, basing TS on WAIPW or AIPW estimators leads to more aggressive adaptation than with WIPW or IPW estimators. SMART-AR methods yield good in- and post-trial performance; however, the tuning parameters are less intuitive and effective. For any SMART, we recommend simulating different adaptive randomization methods to see which best aligns with the trial goals.

The weighted versions of the IPW and AIPW estimators are preferred over the unweighted estimators for post-trial inference. Normalized weighted estimators have sampling distributions closer to the standard normal and yield improved coverage of confidence intervals and bounds and ability to identify optimal embedded regimes. When baseline and intermediate subject variables that are correlated with outcome are available, we recommend the WAIPW estimator for post-trial inference when using RAR of any type.

We have taken a frequentist perspective in alignment with standard SMART methodology. An alternative approach is to adopt a fully Bayesian framework and base RAR on relevant posterior distributions for the model components. With a correctly specified model for the joint distribution of all relevant subject variables across all stages and a suitable prior specification, a Bayesian approach can obviate concern over the relevance of asymptotic theory early in the trial at the cost of the need for trial-specific modeling and implementation on the part of the user. See Web Appendix A for further discussion.

## Supplementary Material

ujae152_Supplemental_Files Web Appendices A–C, referenced in Sections 3–5, and code to implement the simulations, are available with this paper at the Biometrics website on Oxford Academic.

## Data Availability

Data sharing is not applicable to this article, as no datasets are generated or analysed. The methods developed will enable design and analysis of future SMARTs.
