# Supplementary material for: Adaptive randomization methods for sequential multiple assignment randomized trials (smarts) via thompson sampling
Source: Biometrics. 2024 Dec 16;80(4):ujae152. doi: 10.1093/biomtc/ujae152 (PMC11647911; doi:10.1093/biomtc/ujae152)
Supplement: ujae152_Supplemental_Files — Web Appendices A–C, referenced in Sections 3–5, and code to implement the simulations, are available with this paper at the Biometrics website on Oxford Academic. [file ujae152_supplemental_files.zip › Supplementary_Material_SMARTS_TS_Norwood_etal_R1.pdf]

BIOMETRICS 000, 000–000 DOI: 000
000 0000

Supplementary Material for "Adaptive Randomization Methods for Sequential Multiple Assignment Randomized Trials (SMARTs) via Thompson Sampling"

Peter Norwood[1,*], Marie Davidian[2], and Eric Laber[3***]**

[1]Quantum Leap Healthcare Collaborative, San Francisco, California, U.S.A.

[2]Department of Statistics, North Carolina State University, Raleigh, North Carolina, U.S.A.

[3]Department of Statistical Science, Duke University, Durham, North Carolina, U.S.A.

*_email:_ p.norwood@quantumleaphealth.org

**_email:_ davidian@ncsu.edu

***_email:_ eric.laber@duke.edu

This paper has been submitted for consideration for publication in *Biometrics*

Web Appendix A: Practical and Methodological Considerations

Updating Randomization Probabilities

As noted in the main paper, although, ideally, randomization probabilities would be updated each time a participant in a SMART requires a new randomization, because this action requires the up-to-date accumulated data on all previous subjects, it may be more feasible in practice to update the probabilities only periodically. In the main paper, we consider the case where the randomization probabilities are updated according to a pre-set, fixed schedule, which for definiteness in describing the scheme we take to be "weekly." In many clinical trials, reporting takes place according to a fixed schedule; thus, aligning updating of the randomization probabilities with this schedule may simplify logistics. In the reconfiguration of the I-SPY 2 breast cancer platform trial as an adaptively-randomized SMART, "I-SPY 2.2" (Khoury et al., 2024; Shatsky et al., 2024) for which the first author of this article serves as the statistician responsible for implementing and maintaining the SMART's adaptive randomization strategy, randomization probabilities are updated monthly, coinciding with regular trial reporting.

An alternative strategy advocated by a referee would be to update the randomization probabilities based on sample size, e.g., after every n subjects complete stage k, similar to a convention often used in single-stage group-sequential trials. This strategy would ensure that the amount of information accrued between two updates would remain roughly the same, whereas under a fixed schedule this feature would hold only if the rate of enrollment in the SMART remains relatively constant over time.

Our proposed up-front and sequential randomization methods could in principle be used under such an updating strategy, at the expense of more complicated notation to describe the mechanics. Group-sequential and other trial designs conventionally schedule interim analyses on the basis of sample size, demonstrating the logistical feasibility. However, the interval

between updating of randomization probabilities in a SMART, particularly with the proposed sequential adaptive randomization approach, would be more frequent than that between interim analyses, which could make this strategy more complex to implement and more susceptible to possible errors. Updating randomization probabilities according to a regular schedule may better facilitate oversight of the randomization process and reduce the potential for errors of implementation.

As noted in Section 2.2 of the main paper, with fixed randomization probabilities, a trial in which randomization is "up-front" to embedded regimes and a SMART that embeds those same regimes are equivalent in that they can be designed so that the expected numbers of subjects receiving each treatment at each stage are the same. Tsiatis, Davidian, Holloway, and Laber (2020, Section 9.2.4) present a mathematical demonstration, and Almirall, Nahum-Shani, Sherwood, and Murphy (2014) discuss practical issues.

Interim AIPW Estimator

A limitation of using the WIPW or WAIPW estimators as the basis for updating randomization probabilities is that only the data on subjects in $\mathfrak{D}_{t-1}$ who have completed the trial and have Y observed are used. Thus, partial information from subjects still progressing through the trial is ignored. To make use of this information, we adopt the Interim AIPW (IAIPW) estimator of Manschot, Laber, and Davidian (2023), which is derived by viewing the partial information through the lens of monotone coarsening (Tsiatis, 2006).

We present the estimator in the case where $K = 2$ and $n_{t-1} = \sum_{i=1}^{N} \Gamma_{t-1,i}$; a general formulation is presented in Manschot et al. (2023). In this setting, the weighted IAIPW (WIAIPW) estimator for $\theta^j = \mathcal{V}(\mathbf{d}^j)$ based on $\mathfrak{D}_{t-1}$ is

$$\widehat{\theta}_t^{WIAIPW,j} = n_{t-1}^{-1} \sum_{i=1}^{N} \Gamma_{t-1,i} W^j_{\tau_i} \left[\frac{\Delta_{t-1,i} C_i^j Y_i}{\pi^j_{\tau_i,2}(\overline{\boldsymbol{X}}_{2,i}) \pi^j_{\tau_i,1}(\boldsymbol{X}_{1,i}) \widehat{\nu}_{\Delta,t-1}} + \left\{ 1 - \frac{\overline{C}^j_{1,i} I(\kappa_{t-1,i} = 2)}{\pi^j_{\tau_i,1}(\boldsymbol{X}_{1,i}) \widehat{\nu}_{2,t-1}} \right\} L_1^j(\boldsymbol{X}_{1,i}) \right.$$
$$\left. + \left\{ \frac{\overline{C}^j_{1,i} I(\kappa_{t-1,i} = 2)}{\pi^j_{\tau_i,1}(\boldsymbol{X}_{1,i}) \widehat{\nu}_{2,t-1}} - \frac{\Delta_{t-1,i} C_i^j}{\pi^j_{\tau_i,2}(\overline{\boldsymbol{X}}_{2,i}) \pi^j_{\tau_i,1}(\boldsymbol{X}_{1,i}) \widehat{\nu}_{\Delta,t-1}} \right\} L_2^j(\overline{\boldsymbol{X}}_{2,i}) \right],$$

where $\widehat{\nu}_{\Delta,t-1} = \sum_{i=1}^{N} \Delta_{t-1,i}/n_{t-1}$ and $\widehat{\nu}_{2,t-1} = \sum_{i=1}^{N} \Gamma_{t-1,i} I(\kappa_{t-1,i} = 2)/n_{t-1}$ are estimators for $P(\Delta_{t-1} = 1 | \Gamma_{t-1} = 1, \mathfrak{D}_{t-1})$ and $P(\kappa_{t-1} = 2 | \Gamma_{t-1} = 1, \mathfrak{D}_{t-1})$, $W^{j}_{\tau_i}$ is a weight, and $L^{j}_{k}(\overline{\boldsymbol{x}}_k)$, $k = 1, 2$, are arbitrary functions of $\overline{\boldsymbol{x}}_k$. The efficient choice for the functions $L^{j}_{k}(\overline{\boldsymbol{x}}_k)$, $k = 1, 2$ are derived from the Q-functions which can be modeled and estimated based on a backward iterative scheme similar to that presented for the WAIPW estimator, with modifications to the definition of the pseudo outcomes for subjects in $\mathfrak{D}_{t-1}$ who have not yet completed the trial; see Manschot et al. (2023). As for (1) and (2), the sampling distribution of $\widehat{\boldsymbol{\theta}}_t^{WIAIPW} = (\widehat{\theta}_t^{WIAIPW,1}, \ldots, \widehat{\theta}_t^{WIAIPW,m})^T$ can be approximated using large sample theory analogous to that presented in the following two sections. The general form of the weights is complex and in preliminary simulations, weighted versions of the estimators did not outperform a version where the weights are one for all τ. Due to this, the simulations in Section 5 of the main paper only use the unweighted IAIPW as a basis for randomization for simplicity. Moreover, we did not find the added precision from using partial information to have a notable impact in randomization.

Details on the AR Approach of Cheung et al.

In our simulations, we compare our proposed sequential randomization approach to two different versions of that of Cheung, Chakraborty, and Davidson (2014). We provide a brief review of the Cheung et al. (2014) approach, which as in the main paper we refer to for brevity as AR, for which the aggressiveness of adaptive randomization can be dictated by choices of tuning parameters. See Cheung et al. (2014) for full details of the approach.

The AR approach was presented in the case of two-stage SMARTs, so we focus on $K = 2$. As in the main paper, let $Q_k(\boldsymbol{h}_k, a_k; \boldsymbol{\beta}_k) = Q_k(\overline{\boldsymbol{x}}_k, \overline{\boldsymbol{a}}_k; \boldsymbol{\beta}_k)$, $k = 1, 2$, be posited parametric models for the Q-functions $Q_k(\boldsymbol{h}_k, a_k) = Q_k(\overline{\boldsymbol{x}}_k, \overline{\boldsymbol{a}}_k)$, $k = 1, 2$. Following a burn-in period, adaptation of the randomization probabilities begins. Updating of the randomization probabilities at week t proceeds as follows. Using the data on subjects in $\mathfrak{D}_{t-1}$ for whom $\Delta_{t-1} = 1$,

carry out the usual Q-learning algorithm for estimation of an optimal treatment regime based on ordinary least squares (e.g., Tsiatis et al., 2020, Sections 5.7.1, 7.4.1) to obtain the estimated Q-functions $Q_k(\boldsymbol{h}_k, a_k; \widehat{\boldsymbol{\beta}}_{k,t})$, $k = 1, 2$, where $\widehat{\boldsymbol{\beta}}_{k,t}$ are the estimators for $\boldsymbol{\beta}_k$ based on these data, and the corresponding mean squared errors $\widehat{\sigma}^2_{k,t}$, $k = 1, 2$. Then at each stage $k = 1, 2$, for the ith subject requiring randomization at that stage at week t, for feasible treatment option $a_k \in \Psi_k(\boldsymbol{H}_{k,i})$, define

$$\widehat{\rho}_t(a_k \mid \boldsymbol{H}_{k,i}) = \exp\left\{ \frac{Q_k(\boldsymbol{H}_{k,i}, a_k; \widehat{\boldsymbol{\beta}}_{k,t})}{\widehat{\sigma}^2_{k,t}} \log(b) \right\}$$

for some prespecified base $b \geqslant 1$. Letting $\widehat{a}^w_{k,i}$ be the estimated worst feasible treatment option for i in $\Psi_k(\boldsymbol{H}_{k,i})$, i.e., that with the smallest estimated Q-function when evaluated at $\boldsymbol{H}_{k,i}$, define

$$\Theta_{k,i}(a_k) = \{Q_k(\boldsymbol{H}_{k,i}, a_k; \widehat{\boldsymbol{\beta}}_{k,t}) - Q_k(\boldsymbol{H}_{k,i}, \widehat{a}^w_{k,i}; \widehat{\boldsymbol{\beta}}_{k,t})\}/\widehat{\sigma}^2_{k,t}.$$

Then the updated randomization probability for i at stage $k = 1, 2$ based on the previous data $\mathfrak{D}_{t-1}$ is

$$\widehat{\eta}_{k,t}(a_k, \boldsymbol{H}_{k,i}, \mathfrak{D}_{t-1}) = \frac{\widehat{\rho}_t(a_k \mid \boldsymbol{H}_{k,i})}{\sum_{a \in \Psi_k(\boldsymbol{H}_{k,i})} \widehat{\rho}_t(a \mid \boldsymbol{H}_{k,i})} = \frac{\exp\{\Theta_{k,i}(a_k)\log(b)\}}{\sum_{a \in \Psi_k(\boldsymbol{H}_{k,i})} \exp\{\Theta_{k,i}(a)\log(b)\}}.$$

As the actual randomization probabilities, Cheung et al. (2014) propose using a weighted average of these probabilities and the nominal fixed randomization probabilities $\eta_k(a_k, \boldsymbol{h}_k)$ used in the burn-in period; specifically, defining

$$\widetilde{\rho}_t(a_k \mid \boldsymbol{H}_{k,i}) = \exp\left[\lambda_t^{b-1} \log\{\eta_k(a_k, \boldsymbol{H}_{k,i}) + (1 - \lambda_t^{b-1})\widehat{\eta}_{k,t}(a_k, \boldsymbol{H}_{k,i}, \mathfrak{D}_{t-1})\right],$$

where $\lambda_t^{b-1} \in [0, 1]$ goes to zero as the number of subjects/weeks grows large. The randomization probability to treatment option $a_k \in \Psi_k(\boldsymbol{H}_{k,i})$ for subject i at week t needing randomization at stage k is then taken as

$$\widetilde{\eta}_{k,t}(a_k, \boldsymbol{H}_{k,i}, \mathfrak{D}_{t-1}) = \frac{\widetilde{\rho}_t(a_k \mid \boldsymbol{H}_{k,i})}{\sum_{a \in \Psi_k(\boldsymbol{H}_{k,i})} \widetilde{\rho}_t(a \mid \boldsymbol{H}_{k,i})}.$$

From the foregoing developments, to "tune" the adaptive randomization scheme, one must specify the base b and $\{\lambda_t\}$. The base b characterizes how "greedy" the scheme is;

in the context of adaptive randomization, the greedier a scheme, the more it is skewed toward using randomization probabilities based on data on previous subjects in the trial and the less toward using fixed randomization. Thus, the greedier the scheme, the more agressive the adaptive randomization. Here, when $b = 1$, the scheme reduces to using the nominal fixed randomization probabilities throughout, so no adaptive randomization, and when $b > 1$, the randomization allocation favors treatment options with larger estimated Q-functions. Thus, smaller values of b correspond to more conservative and larger values to more aggressive adaptive randomization. The authors frame the choice of λ_t in terms of a value $\tau \in [0, 1]$, where τ attenuates the greediness via the weight given to the nominal randomization probabilities, with larger values of τ leading to more attenuation. See Cheung et al. (2014) for further discussion.

In the simulations in Section 5.2 of the main paper, we compare versions of this approach to our proposed sequential randomization approach. For the conservatively-tuned AR approach, AR-1, we set the tuning parameters as $b = 10, \tau = 0.5$; for the more aggressive version, AR-2, we took $b = 100, \tau = 0.025$, and we took $\lambda_t = t^{-1}\tau^{(1-b)}$ for both. For both AR methods, we used the sample average of X_1 to calculate the components that make up the stage 2 probabilities, so that those probabilities were the same for all subjects.

Specification of Burn-In Period and Sample Size

The proposed methods involve a burn-in period during which subjects are randomized nonadaptively using fixed randomization probabilities. The purpose of the burn-in period is to accumulate enough data so that the asymptotic theory on which the relevant confidence distributions are based provides a reasonably good approximation to the true distributions once adaptive randomization commences (at the end of the burn-in). Although the final, post-trial inference on the values of the embedded regimes should ideally be based on a "large" sample size, large enough to ensure that the theory provides a reliable approximation to the

true sampling distributions of the normalized weighted IPW and AIPW value estimators, the quality of the approximation based on the sample size at the end of the burn-in period and the subsequent sample sizes of accruing past data as the trial progresses need not reach this same standard in our experience. In developing the extensive simulation studies we have conducted and report in the main paper and in Web Appendix C, we experimented with conventions for specifying the burn-in period. This exercise led us to the recommended convention we used in our simulations, namely, to continue the burn-in period until each embedded regime has at least 25 subjects who have completed the trial and whose experience in the trial is consistent with following the regime. Note that because a given subject's experience may be consistent with following more than one regime, the total number of subjects in the burn-in is considerably smaller than 25 times the number of regimes. For the up-front RAR approach, we found that with the modest sample size of 25, the asymptotic distribution approximations for the unweighted and weighted IPW and AIPW estimators on which we base updating of randomization probabilities become sufficiently close to normal to initiate adaptation without deleterious effects on in-trial and post-trial performance. Moreover, this sample size is generally sufficient to avoid computational issues in implementation. For the sequential randomization approach, we likewise found that this sample size was adequate for the large-sample normal approximations to the relevant confidence distributions to be sufficiently good to again avoid deleterious effects on in-trial and post-trial performance. We encourage investigators planning to undertake a SMART with RAR to conduct preliminary simulations under realistic assumptions to evaluate the quality of the approximations based on different numbers of burn-in subjects consistent with each regime.

Based on our experience, we remark that the use of RAR probably should be restricted to SMARTs that are relatively large, ideally involving at the least many hundreds of subjects for in-trial and post-trial benefits to be realized.

Additional Simulation Results, Main Paper Scenario

Table A.1 presents results for up-front RAR analogous to those in Table 1 of the main paper for $N = 325$. Results are qualitatively similar to those for $N = 1000$ in Table 1 of the main paper; not surprisingly, with the smaller sample size, the extent of improvement in in-trial performance using RAR over over simple, uniform randomization is less dramatic. Likewise, rates of identifying the optimal regime based on post-trial estimation for $N = 325$ are less impressive than for $N = 1000$, and the benefit of RAR is less pronounced. With $N = 325$, coverage of confidence intervals and bounds mostly achieves the nominal level of 0.95.

[Table 1 about here.]

Table A.2 presents results for sequential RAR analogous to those in Table 2 of the main paper for $N = 325$. Results are qualitatively similar to those for $N = 1000$ in Table 2 of the main paper; as for up-front RAR, with the smaller sample size, the extent of improvement in in-trial performance using RAR over over simple, uniform randomization is less dramatic. Likewise, rates of identifying the optimal regime based on post-trial estimation for $N = 325$ are less impressive than for $N = 1000$, and the benefit of RAR is less pronounced. Coverage of confidence intervals and bounds with $N = 325$ mostly achieves the nominal level of 0.95.

[Table 2 about here.]

Contrast with Fully Bayesian Approaches

An alternative approach that would be an attractive alternative in principle to those proposed in the main paper would be to adopt a fully Bayesian approach and base adaptive randomization on resulting posterior distributions. A potential challenge is the need to develop a full model framework, which would be problem-specific and involve specifying probability distributions for intermediate variables and outcomes and the like along with associated prior distributions for components of the model, which would then be used to obtain the beliefs.

The first two authors of this paper have experience with a Bayesian implementation of RAR in a SMART through their involvement in the reconfiguration of the widely-known I-SPY 2 breast cancer platform trial as an adaptively-randomized SMART, "I-SPY 2.2" (Khoury et al., 2024; Shatsky et al., 2024). We have developed a trial-specific RAR strategy, which is currently being used in this ongoing SMART; the first author is the statistician responsible for updating the randomization probabilities. Because of the specific structure of the trial, involving only binary or categorical variables, the model framework is particularly simple, depending on a series of distinct probabilities, so that the user need only specify a prior distribution for these parameters. In finalizing the approach for use in the I-SPY 2.2 SMART, we evaluated both a fully Bayesian implementation involving (Beta) priors placed on these probabilities (which induces Beta posteriors) and a frequentist analog in which the posterior distribution of probabilities is replaced by their joint asymptotic distribution. We found in extensive simulations that the two approaches performed similarly in terms of the in-trial and post-trial measures of interest. Because of the simplicity of the Bayesian framework, the Bayesian version is currently being implemented in the trial. We are in the process of preparing a manuscript for publication that describes this specific SMART and this RAR approach (which is different from and not subsumed by the approaches in this paper).

In general, the relevant model framework for a given SMART is likely to be considerably more complicated to specify. Our goal in this work is to provide "generic" approaches to RAR for SMARTs in the spirit of Thompson sampling that can be used "off the shelf," without the need for the user to specify a full, context-specific model framework and associated suitable prior distributions for the parameters involved. We feel that this practical tradeoff may be useful in motivating investigators to consider large-scale SMARTs in chronic diseases like cancer where adaptive randomization would be attractive ethically and for incentivizing

enrollment. We are working with a professional software developer to create a publicly-available software tool that will assist investigators in implementing the proposed adaptive randomization methods with only modest specifications on the part of the user required (e.g., form of Q-functions).

We note that, with either a frequentist or Bayesian approach, meaningful updating of randomization probabilities can not commence until some subjects in the trial complete all stages and have their final outcomes ascertained. In a Bayesian approach, this feature can be "automatic" in that the posterior distributions will reflect the lack of information on the outcome, although it may be prudent in practice to implement a burn-in period as a conservative check against unusual early results. In the I-SPY 2.2 SMART, updating of the randomization proabilities began after a 20 patient burn-in period.

Web Appendix B: Theory on Weighted Estimators

Asymptotic Theory

Overview

We use the theory for Martingale estimating functions (Godambe, 1985; Heyde, 1997; Godambe and Heyde, 2010, MEFs) to characterize the asymptotic behavior or our post-trial estimator under Thompson Sampling. For completeness, we state and prove the results we will use.

Suppose that we observe the first T elements of a discrete-time stochastic process, $\boldsymbol{Z}_t, \ldots, \boldsymbol{Z}_T$, taking values in $\mathcal{Z}$. In addition, suppose that the law of this stochastic process is P and our interest is in estimating $\boldsymbol{\theta}^* = \boldsymbol{\theta}(P) \in \boldsymbol{\Theta} \subseteq \mathbb{R}^p$. We assume there exists an $\mathbb{R}^p$-valued process on $\boldsymbol{\Theta}$ given by

$$\mathcal{M}_T(\boldsymbol{\theta}) = \sum_{t=1}^{T} \boldsymbol{M}_t(\boldsymbol{\theta}),$$

such that $\{\boldsymbol{M}_t(\boldsymbol{\theta}^*)\}_{t\geqslant 1}$ is a Martingale difference sequence with respect to the filtration

$\{\mathcal{F}_t\}_{t\geqslant 1}$, i.e., $E\left\{\boldsymbol{M}_t(\boldsymbol{\theta}^*)\middle|\mathcal{F}_{t-1}\right\} = 0$ with probability one for all $t \geqslant 1$. We construct an estimator $\widehat{\boldsymbol{\theta}}_T$ as a root of $\mathcal{M}_T(\boldsymbol{\theta}) = 0$; more generally, it could be that no exact root exists for finite T and thus we select $\widehat{\boldsymbol{\theta}}_T$ as a minimizer of $||\mathcal{M}_T(\boldsymbol{\theta})||_2$, where $||\cdot||_2$ denotes the Euclidean norm.

Consistency

We first consider conditions under which $\widehat{\boldsymbol{\theta}}_T \xrightarrow{p} \boldsymbol{\theta}^*$. Define $\boldsymbol{\xi}_T(\boldsymbol{\theta}) = \sum_{t=1}^T \text{var}\left\{\boldsymbol{M}_t(\boldsymbol{\theta})\middle|\mathcal{F}_{t-1}\right\}$ We will make use of the following conditions.

(A0) $\{\boldsymbol{M}_t(\boldsymbol{\theta}^*)\}_{t\geqslant 1}$ is a Martingale difference sequence with respect to $\{\mathcal{F}_t\}_{t\geqslant 1}$.

(A1) $\mathcal{M}_T(\boldsymbol{\theta})$ is continuously differentiable for all $\boldsymbol{\theta} \in \boldsymbol{\Theta}$ and $\nabla_{\boldsymbol{\theta}}\mathcal{M}_T(\boldsymbol{\theta})$ is invertible with probability one for all $\boldsymbol{\theta} \in \boldsymbol{\Theta}$ provided T is sufficiently large.

(A2) The minimum eigenvalue of $\boldsymbol{\xi}_T(\boldsymbol{\theta}^*)$ satisfies $\lambda_{\min}\{\boldsymbol{\xi}_T(\boldsymbol{\theta}^*)\} \xrightarrow{p} \infty$, as $T \to \infty$.

(A3) For any $\boldsymbol{\theta} \in \boldsymbol{\Theta}$

$$\sup_{\boldsymbol{\theta}\in\boldsymbol{\Theta}} \left|\left| \left\{\boldsymbol{\xi}_T^{-1/2}(\boldsymbol{\theta}^*)\nabla_{\boldsymbol{\theta}}\mathcal{M}_t(\boldsymbol{\theta})\boldsymbol{\xi}_T^{-1/2}(\boldsymbol{\theta}^*)\right\}^{-1} \right|\right| = O_P(1).$$

(A4) $\boldsymbol{\xi}_T^{-1/2}(\boldsymbol{\theta}^*)\mathcal{M}_T(\boldsymbol{\theta}^*) = O_P(1)$.

THEOREM 1: *Assume (A0)-(A4) and suppose that $\widehat{\boldsymbol{\theta}}_t$ satisfies $\mathcal{M}_t(\widehat{\boldsymbol{\theta}}_t) = o_P(1)$, then $\widehat{\boldsymbol{\theta}}_T \xrightarrow{p} \boldsymbol{\theta}^*$ as $T \to \infty$.*

Proof. Using a Taylor's expansion write

$$\mathcal{M}_T(\widehat{\boldsymbol{\theta}}_T) = \mathcal{M}_T(\boldsymbol{\theta}^*) + \nabla_{\boldsymbol{\theta}}\mathcal{M}_T(\overline{\boldsymbol{\theta}}_T)\left(\widehat{\boldsymbol{\theta}}_T - \boldsymbol{\theta}^*\right),$$

where $\overline{\boldsymbol{\theta}}_T$ is an intermediate point between $\widehat{\boldsymbol{\theta}}_T$ and $\boldsymbol{\theta}^*$. Using (A1), we can re-arrange the above expression to obtain

$$\begin{aligned}
\widehat{\boldsymbol{\theta}}_T &= \boldsymbol{\theta}^* + \left\{\nabla_{\boldsymbol{\theta}}\mathcal{M}_T(\overline{\boldsymbol{\theta}}_T)\right\}^{-1}\left\{\mathcal{M}_T(\widehat{\boldsymbol{\theta}}_T) - \mathcal{M}_T(\boldsymbol{\theta}^*)\right\} \\
&= \boldsymbol{\theta}^* + \left\{\boldsymbol{\xi}_T^{1/2}(\boldsymbol{\theta}^*)\boldsymbol{\xi}_T^{-1/2}(\boldsymbol{\theta}^*)\nabla_{\boldsymbol{\theta}}\mathcal{M}_T(\overline{\boldsymbol{\theta}}_T)\boldsymbol{\xi}_T^{-1/2}(\boldsymbol{\theta}^*)\boldsymbol{\xi}_T^{1/2}(\boldsymbol{\theta}^*)\right\}^{-1}\left\{\mathcal{M}_T(\widehat{\boldsymbol{\theta}}_T) - \mathcal{M}_T(\overline{\boldsymbol{\theta}}_T)\right\} \\
&= \boldsymbol{\theta}^* + \boldsymbol{\xi}_T^{-1/2}(\boldsymbol{\theta}^*)\left\{\boldsymbol{\xi}_T^{-1/2}(\boldsymbol{\theta}^*)\nabla_{\boldsymbol{\theta}}\mathcal{M}_T(\overline{\boldsymbol{\theta}}_T)\boldsymbol{\xi}_T^{-1/2}(\boldsymbol{\theta}^*)\right\}^{-1}\boldsymbol{\xi}_T^{-1/2}(\boldsymbol{\theta}^*)\left\{\mathcal{M}_T(\widehat{\boldsymbol{\theta}}_T) - \mathcal{M}_T(\boldsymbol{\theta}^*)\right\}.
\end{aligned}$$

From the minimum eigenvalue condition $||\boldsymbol{\xi}_T^{-1/2}(\boldsymbol{\theta}^*)||_F = o_P(1)$, where $||\cdot||_F$ is the Frobenius norm. Furthermore, from (A3) it follows that

$$|| \left\{ \boldsymbol{\xi}_T^{-1/2}(\boldsymbol{\theta}^*) \nabla_{\boldsymbol{\theta}} \mathcal{M}_T(\overline{\boldsymbol{\theta}}_T) \boldsymbol{\xi}_T^{-1/2}(\boldsymbol{\theta}^*) \right\}^{-1} || = O_P(1).$$

Finally, we see that $\boldsymbol{\xi}_T^{-1/2}(\boldsymbol{\theta}^*)\mathcal{M}_T(\widehat{\boldsymbol{\theta}}_T)$ is the product of $o_P(1)$ terms and thus is $o_p(1)$, and $\boldsymbol{\xi}_T^{-1/2}(\boldsymbol{\theta}^*)\mathcal{M}_T(\boldsymbol{\theta}^*) = O_P(1)$ from (A4). Thus, we have shown $\widehat{\boldsymbol{\theta}}_T = \boldsymbol{\theta}^* + o_P(1)$.

Asymptotic Normality

To establish normality we replace (A4) with

(A5) Let $\boldsymbol{I}_p$ denote the $p \times p$ identity matrix, $\boldsymbol{\xi}_T^{-1/2}(\boldsymbol{\theta}^*)\mathcal{M}_T(\boldsymbol{\theta}^*) \xrightarrow{D} \mathcal{N}(0, \boldsymbol{I}_p)$.

In addition, we assume the following conditions.

(A6) For any consistent estimator $\widetilde{\boldsymbol{\theta}}_T \xrightarrow{p} \boldsymbol{\theta}^*$

$$\left|\left| \boldsymbol{\xi}_T^{-1/2}(\boldsymbol{\theta}^*) \left\{ \nabla_{\boldsymbol{\theta}} \mathcal{M}_T(\widetilde{\boldsymbol{\theta}}_T) - \nabla_{\boldsymbol{\theta}} \mathcal{M}_T(\boldsymbol{\theta}^*) \right\} \boldsymbol{\xi}_T^{-1/2}(\boldsymbol{\theta}^*) \right|\right| \xrightarrow{p} 0,$$

as $T \to \infty$.

(A7) There exists fixed and positive definite matrix $\boldsymbol{\Sigma}$ such that

$$-\boldsymbol{\xi}_T^{-1/2}(\boldsymbol{\theta}^*) \nabla_{\boldsymbol{\theta}} \mathcal{M}_T(\boldsymbol{\theta}^*) \boldsymbol{\xi}_T^{-1/2}(\boldsymbol{\theta}^*) \xrightarrow{p} \boldsymbol{\Sigma},$$

as $T \to \infty$.

(A8) $\widehat{\boldsymbol{\theta}}_T$ satisfies $\boldsymbol{\xi}_T^{-1/2}(\boldsymbol{\theta}^*)\mathcal{M}_T(\widehat{\boldsymbol{\theta}}_T) \xrightarrow{p} 0$ as $T \to \infty$.

THEOREM 2: *Assume (A0)-(A3) and(A5)-(A8), then* $\boldsymbol{\Sigma}\boldsymbol{\xi}_T^{1/2}(\boldsymbol{\theta}^*) \left(\widehat{\boldsymbol{\theta}}_T - \boldsymbol{\theta}^* \right) \xrightarrow{D} \mathcal{N}(0, \boldsymbol{I}_p)$ *as* $T \to \infty$.

Proof. As in our proof of consistency, we begin with a Taylor series expansion

$$\mathcal{M}_T(\widehat{\boldsymbol{\theta}}_T) = \mathcal{M}_T(\boldsymbol{\theta}^*) + \nabla_{\boldsymbol{\theta}} \mathcal{M}_T(\overline{\boldsymbol{\theta}}_T) \left(\widehat{\boldsymbol{\theta}}_T - \boldsymbol{\theta}^* \right),$$

where $\overline{\boldsymbol{\theta}}_T$ is an intermediate point between $\widehat{\boldsymbol{\theta}}_T$ and $\boldsymbol{\theta}^*$. re-arranging terms yields

$$
\begin{aligned}
(\widehat{\boldsymbol{\theta}}_T - \boldsymbol{\theta}^*) &= \left\{\nabla_{\boldsymbol{\theta}} \mathcal{M}_T(\overline{\boldsymbol{\theta}}_T)\right\}^{-1} \left\{\mathcal{M}_T(\widehat{\boldsymbol{\theta}}_T) - \mathcal{M}_T(\boldsymbol{\theta}^*)\right\} \\
\Leftrightarrow \boldsymbol{\xi}_T^{1/2}(\boldsymbol{\theta}^*)(\widehat{\boldsymbol{\theta}}_T - \boldsymbol{\theta}^*) &= \boldsymbol{\xi}_T^{1/2}(\boldsymbol{\theta}^*) \left\{\boldsymbol{\xi}_T^{1/2}(\boldsymbol{\theta}^*)\boldsymbol{\xi}_T^{-1/2}(\boldsymbol{\theta}^*)\nabla_{\boldsymbol{\theta}} \mathcal{M}_T(\overline{\boldsymbol{\theta}}_T)\boldsymbol{\xi}_T^{-1/2}(\boldsymbol{\theta}^*)\boldsymbol{\xi}_T^{1/2}(\boldsymbol{\theta}^*)\right\}^{-1} \\
&\times \left\{\mathcal{M}_T(\widehat{\boldsymbol{\theta}}_T) - \mathcal{M}_T(\boldsymbol{\theta}^*)\right\} \\
&= \left\{\boldsymbol{\xi}_T^{-1/2}(\boldsymbol{\theta}^*)\nabla_{\boldsymbol{\theta}} \mathcal{M}_T(\overline{\boldsymbol{\theta}}_T)\boldsymbol{\xi}_T^{-1/2}(\boldsymbol{\theta}^*)\right\} \boldsymbol{\xi}_T^{-1/2}(\boldsymbol{\theta}^*) \left\{\mathcal{M}_T(\widehat{\boldsymbol{\theta}}_T) - \mathcal{M}_T(\boldsymbol{\theta}^*)\right\} \\
&= \left\{-\boldsymbol{\xi}_T^{-1/2}(\boldsymbol{\theta}^*)\nabla_{\boldsymbol{\theta}} \mathcal{M}_T(\overline{\boldsymbol{\theta}}_T)\boldsymbol{\xi}_T^{-1/2}(\boldsymbol{\theta}^*)\right\}^{-1} \boldsymbol{\xi}_T^{-1/2}(\boldsymbol{\theta}^*)\mathcal{M}_T(\boldsymbol{\theta}^*) \\
&\quad + \left\{\boldsymbol{\xi}_T^{-1/2}(\boldsymbol{\theta}^*)\nabla_{\boldsymbol{\theta}} \mathcal{M}_T(\overline{\boldsymbol{\theta}}_T)\boldsymbol{\xi}_T^{-1/2}(\boldsymbol{\theta}^*)\right\}^{-1} \boldsymbol{\xi}_T^{-1/2}(\boldsymbol{\theta}^*)\mathcal{M}_T(\widehat{\boldsymbol{\theta}}_T) \\
&= \boldsymbol{Z}_T + \boldsymbol{E}_T.
\end{aligned}
$$

We show that $\boldsymbol{Z}_T \xrightarrow{D} \mathcal{N}(0, \boldsymbol{\Sigma}^{-1}\boldsymbol{\Sigma}^{-T})$ and that $\boldsymbol{E}_T = o_P(1)$, where $\boldsymbol{\Sigma}^{-T}$ is the transpose of $\boldsymbol{\Sigma}^{-1}$. To obtain the limit for $\boldsymbol{Z}_T$ we write

$$
\begin{aligned}
\boldsymbol{Z}_T &= \left\{-\boldsymbol{\xi}_T^{-1/2}(\boldsymbol{\theta}^*)\nabla_{\boldsymbol{\theta}} \mathcal{M}_T(\overline{\boldsymbol{\theta}}_T)\boldsymbol{\xi}_T^{-1/2}(\boldsymbol{\theta}^*)\right\}^{-1} \boldsymbol{\xi}_T^{-1/2}(\boldsymbol{\theta}^*)\mathcal{M}_T(\boldsymbol{\theta}^*) \\
&= \left[-\boldsymbol{\xi}_T^{-1/2}(\boldsymbol{\theta}^*)\nabla_{\boldsymbol{\theta}} \mathcal{M}_T(\boldsymbol{\theta}^*)\boldsymbol{\xi}_T^{-1/2}(\boldsymbol{\theta}^*) + \boldsymbol{\xi}_T^{-1/2}(\boldsymbol{\theta}^*)\left\{\nabla_{\boldsymbol{\theta}} \mathcal{M}_T(\boldsymbol{\theta}^*) - \nabla_{\boldsymbol{\theta}} \mathcal{M}_T(\overline{\boldsymbol{\theta}}_T)\right\}\boldsymbol{\xi}_T^{-1/2}(\boldsymbol{\theta}^*)\right]^{-1} \\
&\times \boldsymbol{\xi}_T^{-1/2}(\boldsymbol{\theta}^*)\mathcal{M}_T(\boldsymbol{\theta}^*),
\end{aligned}
$$

applying (A6), it follows that the term inside the square brackets is $\boldsymbol{\Sigma}^{-1} + o_P(1)$ so that

$$
\boldsymbol{Z}_T = \boldsymbol{\Sigma}^{-1}\boldsymbol{\xi}_T^{1/2}(\boldsymbol{\theta}^*)\mathcal{M}_T(\boldsymbol{\theta}^*) + o_P(1) \xrightarrow{D} \mathcal{N}(0, \boldsymbol{\Sigma}^{-1}\boldsymbol{\Sigma}^{-T}).
$$

It remains to show that $\boldsymbol{E}_T = o_P(1)$. From (A3) $\left\{\boldsymbol{\xi}_T^{-1/2}(\boldsymbol{\theta}^*)\nabla_{\boldsymbol{\theta}} \mathcal{M}_T(\overline{\boldsymbol{\theta}}_T)\boldsymbol{\xi}_T^{-1/2}(\boldsymbol{\theta}^*)\right\}^{-1} = O_P(1)$. Furthermore, $\boldsymbol{\xi}_T^{-1/2}(\boldsymbol{\theta}^*)\mathcal{M}_T(\widehat{\boldsymbol{\theta}}_T) = o_P(1)$ from (A8). This proves the result.

Choosing the Weights

WIPW Estimator After Up-Front Randomization

Consider the WIPW estimator for the value θ^j of jth embedded regime in (1) in the main paper at the end of the trial under the conditions of Section 4 of the main paper, so that subjects are indexed by t. We require that the weights W_t^j be chosen as functions of $\mathfrak{D}_{t-1}$ so that

(i) the estimating equations are conditionally unbiased, $E\{M_t^j(\overline{\boldsymbol{X}}_{K,t}, \overline{\boldsymbol{A}}_{K,t}, Y_t; \theta^j)|\mathfrak{D}_{t-1}\} = 0$, and (ii) the variance is stabilized, $E[\{M_t^j(\overline{\boldsymbol{X}}_{K,t}, \overline{\boldsymbol{A}}_{K,t}, Y_t; \theta^j)\}^2|\mathfrak{D}_{t-1}] = \sigma > 0$ for all t, where $M_t^j(\overline{\boldsymbol{X}}_{K,t}, \overline{\boldsymbol{A}}_{K,t}, Y_t; \theta^j)$ is the estimating function (4) of the main paper given by

$$M_t^j(\overline{\boldsymbol{X}}_{K,t}, \overline{\boldsymbol{A}}_{K,t}, Y_t; \theta^j) = \frac{W_t^j C_t^j}{\{\prod_{k=2}^K \pi_{t,k}^j(\overline{\boldsymbol{X}}_{k,t})\}\pi_{t,1}^j(\boldsymbol{X}_{1,t})}(Y_t - \theta^j).$$

As in Section 4 of the main paper, take $\overline{\boldsymbol{X}}_k^*(\mathbf{d}^j) = \{\boldsymbol{X}_1, \boldsymbol{X}_2^*(\mathbf{d}^j), \ldots, \boldsymbol{X}_k^*(\mathbf{d}^j)\}$, $k = 1, \ldots, K$.

To show (i), when $C_t^j = 1$, the consistency assumption given in Section 2.1 of the main paper implies that $Y_t = Y^*(\mathbf{d}^j)$ and $\overline{\boldsymbol{X}}_{K,t} = \overline{\boldsymbol{X}}_K^*(\mathbf{d}^j)$. Thus, if W_t^j is a function of $\mathfrak{D}_{t-1}$,

$$\begin{aligned}
&E\{M_t^j(\overline{\boldsymbol{X}}_{K,t}, \overline{\boldsymbol{A}}_{K,t}, Y_t; \theta^j)|\mathfrak{D}_{t-1}\} \\
&= W_t^j E\left[\left.\frac{C_t^j\{Y^*(\mathbf{d}^j) - \theta^j\}}{\prod_{k=2}^K \pi_{t,k}^j\{\overline{\boldsymbol{X}}_k^*(\mathbf{d}^j)\}\pi_{t,1}^j(\boldsymbol{X}_1)}\right| \mathfrak{D}_{t-1}\right] \\
&= W_t^j E\left(E\left[\left.\frac{C_t^j\{Y^*(\mathbf{d}^j) - \theta^j\}}{\prod_{k=2}^K \pi_{t,k}^j\{\overline{\boldsymbol{X}}_k^*(\mathbf{d}^j)\}\pi_{t,1}^j(\boldsymbol{X}_1)}\right| \mathcal{W}^*, \mathfrak{D}_{t-1}\right]\middle| \mathfrak{D}_{t-1}\right) \\
&= W_t^j E\left[\left.\frac{E(C_t^j|\mathcal{W}^*, \mathfrak{D}_{t-1})}{\prod_{k=2}^K \pi_{t,k}^j\{\overline{\boldsymbol{X}}_k^*(\mathbf{d}^j)\}\pi_{t,1}^j(\boldsymbol{X}_1)}\{Y^*(\mathbf{d}^j) - \theta^j\}\right| \mathfrak{D}_{t-1}\right] \\
&= W_t^j E\left[\left.\frac{\prod_{k=2}^K \pi_{t,k}^j\{\overline{\boldsymbol{X}}_k^*(\mathbf{d}^j)\}\pi_{t,1}^j(\boldsymbol{X}_1)}{\prod_{k=2}^K \pi_{t,k}^j\{\overline{\boldsymbol{X}}_k^*(\mathbf{d}^j)\}\pi_{t,1}^j(\boldsymbol{X}_1)}\{Y^*(\mathbf{d}^j) - \theta^j\}\right| \mathfrak{D}_{t-1}\right] \\
&= W_t^j E\{Y^*(\mathbf{d}^j) - \theta^j\} = 0,
\end{aligned}$$

where we have used the result analogous to that in Section 6.4.3 of Tsiatis et al. (2020) that, under the consistency, positivity, and sequential ignorability assumptions, $E(C_t^j|\mathcal{W}^*, \mathfrak{D}_{t-1}) = \prod_{k=2}^K \pi_{t,k}^j\{\overline{\boldsymbol{X}}_k^*(\mathbf{d}^j)\}\pi_{t,1}^j(\boldsymbol{X}_1)$ and the fact that $\mathcal{W}^*$ and thus $Y^*(\mathbf{d}^j)$ is independent of $\mathfrak{D}_{t-1}$.

To find W_t^j satisfying (ii), we have, using the consistency assumption, $(C_t^j)^2 = C_t^j$, and that $E(C_t^j|\mathcal{W}^*, \mathfrak{D}_{t-1}) = \prod_{k=2}^K \pi_{t,k}^j\{\overline{\boldsymbol{X}}_k^*(\mathbf{d}^j)\}\pi_{t,1}^j(\boldsymbol{X}_1)$, that

$$\begin{aligned}
E\{M_t^j(\overline{\boldsymbol{X}}_{K,t}, \overline{\boldsymbol{A}}_{K,t}, Y_t; \theta^j)^2|\mathfrak{D}_{t-1}\} &= (W_t^j)^2 E\left(\left.\frac{C_t^j\{Y^*(\mathbf{d}^j) - \theta^j\}^2}{\left[\prod_{k=2}^K \pi_{t,k}^j\{\overline{\boldsymbol{X}}_k^*(\mathbf{d}^j)\}\pi_{t,1}^j(\boldsymbol{X}_1)\right]^2}\right| \mathfrak{D}_{t-1}\right) \\
&= (W_t^j)^2 E\left(\left.\frac{E(C_t^j|\mathcal{W}^*, \mathfrak{D}_{t-1})}{\left[\prod_{k=2}^K \pi_{t,k}^j\{\overline{\boldsymbol{X}}_k^*(\mathbf{d}^j)\}\pi_{t,1}^j(\boldsymbol{X}_1)\right]^2}\{Y^*(\mathbf{d}^j) - \theta^j\}^2\right| \mathfrak{D}_{t-1}\right) \\
&= (W_t^j)^2 E\left[\left.\frac{\{Y^*(\mathbf{d}^j) - \theta^j\}^2}{\prod_{k=2}^K \pi_{t,k}^j\{\overline{\boldsymbol{X}}_k^*(\mathbf{d}^j)\}\pi_{t,1}^j(\boldsymbol{X}_1)}\right| \mathfrak{D}_{t-1}\right] \qquad \text{(B.1)}
\end{aligned}$$

as in (6) of the main paper.

To ensure (ii), we thus want to choose W_t^j so that (B.1) is a constant that can be j-specific but that does not depend on $\mathfrak{D}_{t-1}$. For definiteness and simplicity, consider $K = 2$; the following argument extends to general K. As in Section 4 of the main paper for the cancer pain management SMART, randomization at stage 1 ordinarily does not depend on covariate information, which implies that $\pi_{t,1}^j(\boldsymbol{X}_1) = \pi_{t,1}^j$ depending on $\mathfrak{D}_{t-1}$ and j but not X_1. Randomization at stages beyond stage 1 is typically to sets of feasible treatment options within each level of a binary variable such as response status, and depends on no other covariate information. Let $R^*(\mathbf{d}^j)$ be the potential binary response status variable taking on values 0 and 1 for a subject whose first stage treatment assignment is consistent with regime $\mathbf{d}^j$, where $R^*(\mathbf{d}^j)$ is a function of or component of $\overline{\boldsymbol{X}}_2^*(\mathbf{d}^j)$ ($R^*(\mathbf{d}^j) = X_{22}^*(\mathbf{d}^j)$ in the cancer pain management SMART). By the consistency assumption, the observed response status R is equal to $R^*(\mathbf{d}^j)$ when $C_t^j = 1$. Then, as in the cancer pain management SMART, it follows that $\pi_{t,2}^j\{\overline{\boldsymbol{X}}_2^*(\mathbf{d}^j)\} = \pi_{t,2}^{j(s)}$ when $R^*(\mathbf{d}^j) = s$, $s = 0, 1$, where for each r, $\pi_{t,2}^{j(s)}$ depends on $\mathfrak{D}_{t-1}$ and j but not on $\overline{\boldsymbol{X}}_2^*(\mathbf{d}^j)$, so that

$$\pi_{t,2}^j\{\overline{\boldsymbol{X}}_2^*(\mathbf{d}^j)\} = I\{R^*(\mathbf{d}^j) = 0\}\pi_{t,2}^{j(0)} + I\{R^*(\mathbf{d}^j) = 1\}\pi_{t,2}^{j(1)}. \tag{B.2}$$

Because $\mathcal{W}^* \perp\!\!\!\perp \mathfrak{D}_{t-1}$, generalizing (7) of the main paper, it follows that (B.1) is equal to

$$(W_t^j)^2 \left(\frac{\mu^{j(1)}}{\pi_{t,2}^{j(1)}\pi_{t,1}^j} + \frac{\mu^{j(0)}}{\pi_{t,2}^{j(0)}\pi_{t,1}^j} \right), \tag{B.3}$$

where $\mu^{j(s)} = E[I\{R^*(\mathbf{d}^j) = s\}\{Y^*(\mathbf{d}^j) - \theta^j\}^2]$, $s = 0, 1$. Thus, we wish to choose W_t^j so that (B.3) is equal to a constant depending only on j and not on $\mathfrak{D}_{t-1}$. Denoting this constant as Ξ^j, the weights should be chosen so that

$$W_t^j = (\Xi^j)^{1/2} \left(\frac{\mu^{j(1)}}{\pi_{t,2}^{j(1)}\pi_{t,1}^j} + \frac{\mu^{j(0)}}{\pi_{t,2}^{j(0)}\pi_{t,1}^j} \right)^{-1/2}. \tag{B.4}$$

Of course, in practice the constant Ξ^j is unknown. If there is a burn-in period of length t^* weeks during which nonadaptive randomization takes place with $W_t^j \equiv 1$, $t \leqslant t^*$, from (B.3),

it must be the case that Ξ^j satisfies

$$\Xi^j = \frac{\mu^{j(1)}}{\pi^{j(1)}_{t^*,2}\pi^j_{t^*,1}} + \frac{\mu^{j(0)}}{\pi^{j(0)}_{t^*,2}\pi^j_{t^*,1}}, \tag{B.5}$$

where the randomization probabilities during the burn-in period are fixed, so that $\pi^{j(s)}_{t^*,2}$, $s = 0, 1$, and $\pi^j_{t^*,1}$ are known constants not depending on the data. Thus, if $\mu^{j(s)}$, $s = 0, 1$, were known, so that Ξ^j is known, the weights for $t \geqslant t^* + 1$ should be chosen as in (B.4) with Ξ^j as in (B.5).

These observations suggest our proposed scheme for implementation in practice. We propose to obtain weights in this spirit by estimating Ξ^j by an estimator $\widehat{\Xi}^j_{t^*}$, say, based on the data from a burn-in period of length t^* weeks during which nonadaptive randomization takes place with $W^j_t \equiv 1$, $t \leqslant t^*$, and then treat the estimate as fixed and known henceforth. Specifically, let $\widehat{\mu}^{j(s)}_t$ be estimators for $\mu^{j(s)}$, $s = 0, 1$, at any time t based on $\mathfrak{D}_{t-1}$, as in the case of the cancer pain management SMART (in the original notation) in Section 4 of the main paper; with the indexing scheme here (by t), $\widehat{\mu}^{j(s)}_t = t^{-1}\sum_{u=1}^{t} I(R_u = s)C^j_u(Y_u - \widetilde{\theta}^j_t)^2/(\pi^{j(s)}_{u,2}\pi^j_{u,1})$, $s = 0, 1$, where $\widetilde{\theta}^j_t$ is an estimator for θ^j using $\mathfrak{D}_{t-1}$ (e.g., the unweighted IPW estimator). Define

$$\widehat{\Xi}^j_t = \frac{\widehat{\mu}^{j(1)}_t}{\pi^{j(1)}_{t,2}\pi^j_{t,1}} + \frac{\widehat{\mu}^{j(0)}_t}{\pi^{j(0)}_{t,2}\pi^j_{t,1}}.$$

Then (B.5) implies that the estimator $\widehat{\Xi}_{t^*,j}$ for Ξ^j based on the burn-in data is

$$\widehat{\Xi}^j_{t^*} = \frac{\widehat{\mu}^{j(1)}_{t^*}}{\pi^{j(1)}_{t^*,2}\pi^j_{t^*,1}} + \frac{\widehat{\mu}^{j(0)}_{t^*}}{\pi^{j(0)}_{t^*,2}\pi^j_{t^*,1}},$$

where as above $\pi^{j(s)}_{t^*,2}$, $s = 0, 1$, and $\pi^j_{t^*,1}$ are known constants not depending on the data. Treating $\widehat{\Xi}^j_{t^*}$ as fixed and known, for $t \geqslant t^* + 1$, from (B.4), take $W^j_t = (\widehat{\Xi}^j_{t^*}/\widehat{\Xi}^j_t)^{1/2}$. Because $\widehat{\Xi}^j_{t^*}$ depends only on $\mathfrak{D}_{t^*} \subseteq \mathfrak{D}_{t-1}$ for $t \geqslant t^* + 1$, and $\widehat{\Xi}^j_t$ depends only on $\mathfrak{D}_{t-1}$, the weights W^j_t for $t \geqslant t^* + 1$ depend on $\mathfrak{D}_{t-1}$, as required.

WIPW Estimator After Sequential Randomization

Again consider $K = 2$ for definiteness. The preceding scheme for obtaining weights as-

sumes that up-front randomization is used, so that, returning to the original notation, for a subject who entered the SMART at time τ, the probabilities in the denominator of (1) for that subject's contribution are determined at that point based on $\mathfrak{D}_{\tau-1}$. Thus, if up-front randomization was used, the estimators for $\mu_j^{(s)}$, $s = 0, 1$, and the corresponding weight W_τ^j for a such a subject depend on $\pi_{\tau,2}^{j(s)}$, $s = 0, 1$, and $\pi_{\tau,1}^j$.

If the sequential randomization scheme is used, this calculation should be modified. In obtaining the WIPW estimator for θ^j at the end of the trial, for a subject who entered the SMART at time τ and then reached stage 2 at time $\tau + v$, say, the probabilities in the denominator of (1) should be $\pi_{\tau,1}^j$ and $\pi_{\tau+v,2}^{j(s)}$, $s = 0, 1$. Likewise, the estimators for $\mu_j^{(s)}$, $s = 0, 1$, and the corresponding weight should be calculated based on $\pi_{\tau+v,2}^{j(s)}$, $s = 0, 1$, and $\pi_{\tau,1}^j$, so that the weight for this subject depends on $\mathfrak{D}_{\tau+v-1}$ and thus can be written as $W_{\tau+v}^j$. When $\tau + v < t^*$, $W_{\tau+v}^j = 1$.

WAIPW Estimator After Up-Front Randomization

Analogous to the developments for the WIPW estimator for θ^j for embedded regime j, using the indexing scheme (by t) in Section 4 of the main paper, we identify the corresponding estimating function $M_t^j(\overline{\boldsymbol{X}}_{K,t}, \overline{\boldsymbol{A}}_{K,t}, Y_t; \theta^j)$, demonstrate that (i) $E\{M_t^j(\overline{\boldsymbol{X}}_{K,t}, \overline{\boldsymbol{A}}_{K,t}, Y_t; \theta^j)|\mathfrak{D}_{t-1}\} = 0$, and determine weights $W_t^{A,j}$ so that (ii) the variance $E[\{M_t^j(\overline{\boldsymbol{X}}_{K,t}, \overline{\boldsymbol{A}}_{K,t}, Y_t; \theta^j)\}^2|\mathfrak{D}_{t-1}]$ is a constant depending only on j. We demonstrate for $K = 2$; the argument extends to general K.

From (2) of the main paper, for $K = 2$ it is straightforward that

$$M_t^j(\overline{\boldsymbol{X}}_{2,t}, \overline{\boldsymbol{A}}_{2,t}, Y_t; \theta^j) = W_t^{A,j} \Bigg[\frac{C_t^j Y_t}{\pi_{t,2}^j(\overline{\boldsymbol{X}}_{2,t})\pi_{t,1}^j(\boldsymbol{X}_{1,t})} - \left\{ \frac{\overline{C}_{1,t}^j}{\pi_{t,1}^j(\boldsymbol{X}_{1,t})} - 1 \right\} \widehat{Q}_{1,t}^j(\boldsymbol{X}_{1,t})$$
$$- \left\{ \frac{C^j}{\pi_{t,2}^j(\overline{\boldsymbol{X}}_{2,t})\pi_{t,1}^j(\boldsymbol{X}_{1,t}1)} - \frac{\overline{C}_{1,t}^j}{\pi_{t,1}^j(\boldsymbol{X}_{1,t})} \right\} \widehat{Q}_{2,t}^j(\overline{\boldsymbol{X}}_{2,t}) - \theta^j \Bigg],$$

where as in the main paper $\overline{C}_{1,t}^j = I\{A_{1,t} = d_1^j(\boldsymbol{X}_{1,t})\}$, and for brevity we write $\widehat{Q}_{2,t}^j(\overline{\boldsymbol{X}}_{2,t}) = Q_2\{\overline{\boldsymbol{X}}_{2,t}, \overline{d}_2^j(\overline{\boldsymbol{X}}_{2,t}; \widehat{\boldsymbol{\beta}}_{2,t}\}$ and $\widehat{Q}_{1,t}^j(\boldsymbol{X}_{1,t}) = Q_1^j\{\boldsymbol{X}_{1,t}, d_1^j(\boldsymbol{X}_{1,t}); \widehat{\boldsymbol{\beta}}_{1,t}^j\}$, which are fitted using $\mathfrak{D}_{t-1}$. To show (i), using the consistency assumption and rearranging, similar to the argument

for the WIPW estimator, if $W_t^{A,j}$ is a function of $\mathfrak{D}_{t-1}$,

$$E\{M_t^j(\overline{\boldsymbol{X}}_{2,t}, \overline{\boldsymbol{A}}_{2,t}, Y_t; \theta^j)|\mathfrak{D}_{t-1}\} = W_t^{A,j} \Bigg[E\big[\{Y^*(\mathbf{d}^j) - \theta^j\}|\mathfrak{D}_{t-1}\big] \tag{B.6}$$

$$+ E\left(\left[\frac{\overline{C}_{1,t}^j}{\pi_{t,1}^j(\boldsymbol{X}_1)} - 1\right]\{Y^*(\mathbf{d}^j) - \widehat{Q}_{1,t}^j(\boldsymbol{X}_1)\}\middle|\mathfrak{D}_{t-1}\right) \tag{B.7}$$

$$+ \left(\left[\frac{C_t^j}{\pi_{t,2}^j\{\overline{\boldsymbol{X}}_2^*(\mathbf{d}^j)\}\pi_{t,1}^j(\boldsymbol{X}_1)} - \frac{\overline{C}_{1,t}^j}{\pi_{t,1}^j(\boldsymbol{X}_1)}\right][Y^*(\mathbf{d}^j) - \widehat{Q}_{2,t}^j\{\overline{\boldsymbol{X}}_k^*(\mathbf{d}^j)\}]\middle|\mathfrak{D}_{t-1}\right)\Bigg]. \tag{B.8}$$

Using $\mathcal{W}^* \perp\!\!\!\perp \mathfrak{D}_{t-1}$, it follows that $E[\{Y^*(\mathbf{d}^j) - \theta^j\}|\mathfrak{D}_{t-1}] = E[\{Y^*(\mathbf{d}^j) - \theta^j\}] = 0$, so that the conditional expectation in (B.6) is zero. Using arguments similar to those for the WIPW estimator, the conditional expectations in (B.7) and (B.8) can also be shown to be equal to zero using $E(C_t^j|\mathcal{W}^*, \mathfrak{D}_{t-1}) = \pi_{t,2}^j\{\overline{\boldsymbol{X}}_2^*(\mathbf{d}^j)\}\pi_{t,1}^j(\boldsymbol{X}_1)$ and $E(\overline{C}_{1,t}^j|\mathcal{W}^*, \mathfrak{D}_{t-1}) = \pi_{t,1}^j(\boldsymbol{X}_1)$. Thus, (i) holds.

To find $E[\{M_t^j(\overline{\boldsymbol{X}}_{K,t}, \overline{\boldsymbol{A}}_{K,t}, Y_t; \theta^j)\}^2|\mathfrak{D}_{t-1}]$, first note that the conditional (on $\mathfrak{D}_{t-1}$) expectations of crossproduct terms in $\{M_t^j(\overline{\boldsymbol{X}}_{K,t}, \overline{\boldsymbol{A}}_{K,t}, Y_t; \theta^j)\}^2$ are equal to zero by similar arguments. For example, it is straightforward that

$$(W_t^{A,j})^2 E\left(\left[\frac{\overline{C}_{1,t}^j}{\pi_{t,1}^j(\boldsymbol{X}_1)} - 1\right]\{Y^*(\mathbf{d}^j) - \theta^j\}\{Y^*(\mathbf{d}^j) - \widehat{Q}_{1,t}^j(\boldsymbol{X}_1)\}\middle|\mathfrak{D}_{t-1}\right) = 0$$

using $\mathcal{W}^* \perp\!\!\!\perp \mathfrak{D}_{t-1}$ and $E(\overline{C}_{1,t}^j|\mathcal{W}^*, \mathfrak{D}_{t-1}) = \pi_{t,1}^j(\boldsymbol{X}_1)$. Thus,

$$E[\{M_t^j(\overline{\boldsymbol{X}}_{K,t}, \overline{\boldsymbol{A}}_{K,t}, Y_t; \theta^j)\}^2|\mathfrak{D}_{t-1}] = (W_t^{A,j})^2 \Bigg[E[\{Y^*(\mathbf{d}^j) - \theta^j\}^2|\mathfrak{D}_{t-1}] \tag{B.9}$$

$$+ E\left(\left[\frac{\overline{C}_{1,t}^j}{\pi_{t,1}^j(\boldsymbol{X}_1)} - 1\right]^2\{Y^*(\mathbf{d}^j) - \widehat{Q}_{1,t}^j(\boldsymbol{X}_1)\}^2\middle|\mathfrak{D}_{t-1}\right) \tag{B.10}$$

$$+ \left(\left[\frac{C_t^j}{\pi_{t,2}^j\{\overline{\boldsymbol{X}}_2^*(\mathbf{d}^j)\}\pi_{t,1}^j(\boldsymbol{X}_1)} - \frac{\overline{C}_{1,t}^j}{\pi_{t,1}^j(\boldsymbol{X}_1)}\right]^2[Y^*(\mathbf{d}^j) - \widehat{Q}_{2,t}^j\{\overline{\boldsymbol{X}}_k^*(\mathbf{d}^j)\}]^2\middle|\mathfrak{D}_{t-1}\right)\Bigg]. \tag{B.11}$$

In (B.9), using $\mathcal{W}^* \perp\!\!\!\perp \mathfrak{D}_{t-1}$, $E[\{Y^*(\mathbf{d}^j) - \theta^j\}^2|\mathfrak{D}_{t-1}] = E[\{Y^*(\mathbf{d}^j) - \theta^j\}^2]$. Again using $\mathcal{W}^* \perp\!\!\!\perp \mathfrak{D}_{t-1}$ and the fact that $E(\overline{C}_{1,t}^j|\mathcal{W}^*, \mathfrak{D}_{t-1}) = \pi_{t,1}^j(\boldsymbol{X}_1)$ implies that

$$E\left[\left\{\frac{\overline{C}_{1,t}^j}{\pi_{t,1}^j(\boldsymbol{X}_1)} - 1\right\}^2\middle|\mathcal{W}^*, \mathfrak{D}_{t-1}\right] = \frac{1 - \pi_{t,1}^j(\boldsymbol{X}_1)}{\pi_{t,1}^j(\boldsymbol{X}_1)},$$

so that the conditional expectation in (B.10) is

$$E\left[\{Y^*(\mathbf{d}^j)-\widehat{Q}^j_{1,t}(\boldsymbol{X}_1)\}^2\left\{\frac{1-\pi^j_{t,1}(\boldsymbol{X}_1)}{\pi^j_{t,1}(\boldsymbol{X}_1)}\right\}\middle|\mathfrak{D}_{t-1}\right].$$

Finally, using $E(C^j_t|\mathcal{W}^*,\mathfrak{D}_{t-1})=\pi^j_{t,2}\{\overline{\boldsymbol{X}}^*_2(\mathbf{d}^j)\}\pi^j_{t,1}(\boldsymbol{X}_1)$ and $E(\overline{C}^j_{1,t}|\mathcal{W}^*,\mathfrak{D}_{t-1})=\pi^j_{t,1}(\boldsymbol{X}_1)$, it is straightforward to derive that

$$E\left(\left[\frac{C^j_t}{\pi^j_{t,2}\{\overline{\boldsymbol{X}}^*_2(\mathbf{d}^j)\}\pi^j_{t,1}(\boldsymbol{X}_1)}-\frac{\overline{C}^j_{1,t}}{\pi^j_{t,1}(\boldsymbol{X}_1)}\right]^2\middle|\mathcal{W}^*,\mathfrak{D}_{t-1}\right)=\frac{1-\pi^j_{t,2}\{\overline{\boldsymbol{X}}^*_2(\mathbf{d}^j)\}}{\pi^j_{t,2}\{\overline{\boldsymbol{X}}^*_2(\mathbf{d}^j)\}\pi^j_{t,1}(\boldsymbol{X}_1)}.$$

Using this result, the conditional expectation in (B.11) is

$$E\left([Y^*(\mathbf{d}^j)-\widehat{Q}^j_{2,t}\{\overline{\boldsymbol{X}}^*_k(\mathbf{d}^j)\}]^2\frac{1-\pi^j_{t,2}\{\overline{\boldsymbol{X}}^*_2(\mathbf{d}^j)\}}{\pi^j_{t,2}\{\overline{\boldsymbol{X}}^*_2(\mathbf{d}^j)\}\pi^j_{t,1}(\boldsymbol{X}_1)}\middle|\mathfrak{D}_{t-1}\right).$$

Substituting the foregoing results in (B.9), (B.10), and (B.11) yields

$$\begin{aligned}E[\{M^j_t(\overline{\boldsymbol{X}}_{K,t},\overline{\boldsymbol{A}}_{K,t},Y_t;\theta^j)\}^2|\mathfrak{D}_{t-1}]&=(W^{A,j}_t)^2\Bigg\{E\big[\{Y^*(\mathbf{d}^j)-\theta^j\}^2\big]\\&+E\left[\{Y^*(\mathbf{d}^j)-\widehat{Q}^j_{1,t}(\boldsymbol{X}_1)\}^2\left\{\frac{1-\pi^j_{t,1}(\boldsymbol{X}_1)}{\pi^j_{t,1}(\boldsymbol{X}_1)}\right\}\middle|\mathfrak{D}_{t-1}\right]\\&+E\left([Y^*(\mathbf{d}^j)-\widehat{Q}^j_{2,t}\{\overline{\boldsymbol{X}}^*_k(\mathbf{d}^j)\}]^2\left[\frac{1-\pi^j_{t,2}\{\overline{\boldsymbol{X}}^*_2(\mathbf{d}^j)\}}{\pi^j_{t,2}\{\overline{\boldsymbol{X}}^*_2(\mathbf{d}^j)\}\pi^j_{t,1}(\boldsymbol{X}_1)}\right]\middle|\mathfrak{D}_{t-1}\right)\Bigg\}.\end{aligned}\tag{B.12}$$

Define $\pi^j_{t,1}$ and $\pi^{j(s)}_{t,2}$, $s=0,1$, which depend only on $\mathfrak{D}_{t-1}$ and j, and $R^*(\mathbf{d}^j)$ as for the WIPW estimator, so that $\pi^j_{t,2}\{\overline{\boldsymbol{X}}^*_2(\mathbf{d}^j)\}=I\{R^*(\mathbf{d}^j)=0\}\pi^{j(0)}_{t,2}+I\{R^*(\mathbf{d}^j)=1\}\pi^{j(1)}_{t,2}$ as in (B.2). Then (B.12) can be rewritten as

$$(W^{A,j}_t)^2\Bigg\{E\big[\{Y^*(\mathbf{d}^j)-\theta^j\}\big]^2\tag{B.13}$$

$$+\left(\frac{1-\pi^j_{t,1}}{\pi^j_{t,1}}\right)E\left[\{Y^*(\mathbf{d}^j)-\widehat{Q}^j_{1,t}(\boldsymbol{X}_1)\}^2\middle|\mathfrak{D}_{t-1}\right]\tag{B.14}$$

$$+\left(\frac{1-\pi^{j(0)}_{t,2}}{\pi^{j(0)}_{t,2}\pi^j_{t,1}}\right)E\left(I\{R^*(\mathbf{d}^j)=0\}[Y^*(\mathbf{d}^j)-\widehat{Q}^j_{2,t}\{\overline{\boldsymbol{X}}^*_k(\mathbf{d}^j)\}]^2\middle|\mathfrak{D}_{t-1}\right)\tag{B.15}$$

$$+\left(\frac{1-\pi^{j(1)}_{t,2}}{\pi^{j(1)}_{t,2}\pi^j_{t,1}}\right)E\left(I\{R^*(\mathbf{d}^j)=1\}[Y^*(\mathbf{d}^j)-\widehat{Q}^j_{2,t}\{\overline{\boldsymbol{X}}^*_k(\mathbf{d}^j)\}]^2\middle|\mathfrak{D}_{t-1}\right)\Bigg\}.\tag{B.16}$$

Write the expectations in (B.13)–(B.16) respectively as $\nu^j=E[\{Y^*(\mathbf{d}^j)-\theta^j\}]^2$, $\nu^j_1=E[\{Y^*(\mathbf{d}^j)-\widehat{Q}^j_{1,t}(\boldsymbol{X}_1)\}^2|\mathfrak{D}_{t-1}]$, and

$$\nu^{j(s)}_2=E\left(I\{R^*(\mathbf{d}^j)=1\}[Y^*(\mathbf{d}^j)-\widehat{Q}^j_{2,t}\{\overline{\boldsymbol{X}}^*_k(\mathbf{d}^j)\}]^2\middle|\mathfrak{D}_{t-1}\right),\quad s=0,1,$$

so that the variance (B.12) can be written succinctly as

$$(W_t^{A,j})^2 \left\{ \nu^j + \nu_1^j \left(\frac{1-\pi_{t,1}^j}{\pi_{t,1}^j} \right) + \nu_2^{j(0)} \left(\frac{1-\pi_{t,2}^{j(0)}}{\pi_{t,2}^{j(0)}\pi_{t,1}^j} \right) + \nu_2^{j(1)} \left(\frac{1-\pi_{t,2}^{j(1)}}{\pi_{t,2}^{j(1)}\pi_{t,1}^j} \right) \right\}. \tag{B.17}$$

Thus, as for the WIPW estimator, we wish to choose $W_t^{A,j}$ so that (B.17) is a constant depending on j but not $\mathfrak{D}_{t-1}$. Denoting this constant as $\Xi^{A,j}$, the weights should be chosen so that

$$W_t^{A,j} = (\Xi^{A,j})^{1/2} \left\{ \nu_j + \nu_j^j \left(\frac{1-\pi_{t,1}^j}{\pi_{t,1}^j} \right) + \nu_2^{j(0)} \left(\frac{1-\pi_{t,2}^{j(0)}}{\pi_{t,2}^{j(0)}\pi_{t,1}^j} \right) + \nu_2^{j(1)} \left(\frac{1-\pi_{t,2}^{j(1)}}{\pi_{t,2}^{j(1)}\pi_{t,1}^j} \right) \right\}^{-1/2}. \tag{B.18}$$

With a burn-in period of length t^* weeks of nonadaptive randomization with $W_t^{A,j} \equiv 1$, from (B.18), it must be that

$$\Xi^{A,j} = \nu^j + \nu_1^j \left(\frac{1-\pi_{t^*,1}^j}{\pi_{t^*,1}^j} \right) + \nu_2^{j(0)} \left(\frac{1-\pi_{t^*,2}^{j(0)}}{\pi_{t^*,2}^{j(0)}\pi_{t^*,1}^j} \right) + \nu_2^{j(1)} \left(\frac{1-\pi_{t^*,2}^{j(1)}}{\pi_{t,2}^{j(1)}\pi_{t^*,1}^j} \right), \tag{B.19}$$

where as before the randomization probabilities during the burn-in period are fixed, so that $\pi_{t^*,2}^{j(s)}$, $s=0,1$, and $\pi_{t^*,1}^j$ are known constants not depending on the data. Thus, if ν^j, ν_1^j, and $\nu_2^{j(s)}$, $s=0,1$, were known, so that $\Xi^{A,j}$ is known, the weights for $t \geqslant t^*+1$ should be chosen as in (B.18) with $\Xi^{A,j}$ as in (B.19).

Analogous to the approach for the WIPW estimator, the proposed implementation scheme is as follows. Obtain weights by estimating $\Xi^{A,j}$ by an estimator $\widehat{\Xi}_{t^*}^{A,j}$ based on the data from a burn-in period of length t^* weeks with nonadaptive randomization with $W_t^{A,j} \equiv 1$, $t \leqslant t^*$, and then going forward treat the estimate as fixed and known. Letting $\widehat{\nu}_t^j$, $\widehat{\nu}_{t,1}^j$, and $\widehat{\nu}_{t,2}^{j(s)}$, $s=0,1$, be estimators for ν^j, ν_1^j, and $\nu_2^{j(s)}$, $s=0,1$, at any time t based on $\mathfrak{D}_{t-1}$, define

$$\widehat{\Xi}_t^{A,j} = \widehat{\nu}_t^j + \widehat{\nu}_{t,1}^j \left(\frac{1-\pi_{t,1}^j}{\pi_{t,1}^j} \right) + \widehat{\nu}_{t,2}^{j(0)} \left(\frac{1-\pi_{t,2}^{j(0)}}{\pi_{t^*,2}^{j(0)}\pi_{t,1}^j} \right) + \widehat{\nu}_{t,2}^{j(1)} \left(\frac{1-\pi_{t,2}^{j(1)}}{\pi_{t,2}^{j(1)}\pi_{t,1}^j} \right). \tag{B.20}$$

Then (B.19) implies that the estimator $\widehat{\Xi}_{t^*}^{A,j}$ for $\Xi^{A,j}$ based on the burn-in data is

$$\widehat{\Xi}_{t^*}^{A,j} = \widehat{\nu}_{t^*}^j + \nu_1^j \left(\frac{1-\pi_{t^*,1}^j}{\pi_{t^*,1}^j} \right) + \widehat{\nu}_{t^*,2}^{j(0)} \left(\frac{1-\pi_{t^*,2}^{j(0)}}{\pi_{t^*,2}^{j(0)}\pi_{t^*,1}^j} \right) + \widehat{\nu}_{t^*,2}^{j(1)} \left(\frac{1-\pi_{t^*,2}^{j(1)}}{\pi_{t,2}^{j(1)}\pi_{t^*,1}^j} \right).$$

Then as for the WIPW estimator, treating $\widehat{\Xi}_{t^*}^{A,j}$ as fixed and known, for $t \geqslant t^*+1$, from

(B.18), take $W_t^{A,j} = (\widehat{\Xi}_{t^*}^{A,j}/\widehat{\Xi}_t^{A,j})^{1/2}$. Because $\widehat{\Xi}_{t^*}^{A,j}$ depends only on $\mathfrak{D}_{t^*} \subseteq \mathfrak{D}_{t-1}$ for $t \geqslant t^*+1$, and $\widehat{\Xi}_t^{A,j}$ depends only on $\mathfrak{D}_{t-1}$, the resulting weights $W_t^{A,j}$ for $t \geqslant t^*+1$ depend on $\mathfrak{D}_{t-1}$.

Estimators for estimators for ν^j, ν_1^j, and $\nu_2^{j(s)}$, $s=0,1$, at any time t based on $\mathfrak{D}_{t-1}$ can be constructed using inverse probability weighting. With the current indexing scheme and with R the observed response status, estimators can be obtained as

$$\widehat{\nu}_t^j = t^{-1}\sum_{u=1}^{t} \frac{C_u^j (Y_u - \widetilde{\theta}_{t,j})^2}{I(R_u=0)\pi_{u,2}^{j(0)}\pi_{u,1}^j + I(R_u=1)\pi_{u,2}^{j(1)}\pi_{u,1}^j},$$

$$\widehat{\nu}_{t,1}^j = t^{-1}\sum_{u=1}^{t} \frac{C_u^j \{Y_u - \widehat{Q}_{1,t}^j(\boldsymbol{X}_{1,u})\}^2}{I(R_u=0)\pi_{u,2}^{j(0)}\pi_{u,1}^j + I(R_u=1)\pi_{u,2}^{j(1)}\pi_{u,1}^j},$$

$$\widehat{\nu}_{t,2}^{j(s)} = t^{-1}\sum_{u=1}^{t} \frac{C_u^j I(R_u=s)\{Y_u - \widehat{Q}_{2,t}^j(\overline{\boldsymbol{X}}_{2,u})\}^2}{\pi_{u,2}^{j(s)}\pi_{u,1}^j}, \quad s=0,1.$$

where $\widetilde{\theta}_t^j$ is an estimator for θ^j using $\mathfrak{D}_{t-1}$ (e.g., the unweighted IPW estimator). In the original notation, estimators can be obtained at week t as

$$\widehat{\nu}_t^j = N_t^{-1}\sum_{i=1}^{N} \frac{\Delta_{t-1,i} C_i^j (Y_i - \widetilde{\theta}_t^j)^2}{I(R_i=0)\pi_{\tau_i,2}^{j(0)}\pi_{\tau_i,1}^j + I(R_i=1)\pi_{\tau_i,2}^{j(1)}\pi_{\tau_i,1}^j},$$

$$\widehat{\nu}_{t,1}^j = N_t^{-1}\sum_{i=1}^{N} \frac{\Delta_{t-1,i} C_i^j \{Y_i - \widehat{Q}_{1,t}^j(\boldsymbol{X}_{1,i})\}^2}{I(R_i=0)\pi_{\tau_i,2}^{j(0)}\pi_{\tau_i,1}^j + I(R_i=1)\pi_{\tau_i,2}^{j(1)}\pi_{\tau_i,1}^j},$$

$$\widehat{\nu}_{t,2}^{j(s)} = N_t^{-1}\sum_{i=1}^{N} \frac{\Delta_{t-1,i} C_i^j I(R_i=r)\{Y_i - \widehat{Q}_{2,t}^j(\overline{\boldsymbol{X}}_{2,i})\}^2}{\pi_{\tau_i,2}^{j(s)}\pi_{\tau_i,1}^j}, \quad s=0,1.$$

WAIPW After Sequential Randomization

The considerations for obtaining the WAIPW estimator at the end of the trial following sequential randomization are analogous to those for WIPW. Again take $K=2$, and consider a subject who entered the SMART at time τ and then reached stage 2 at time $\tau+v$. As for the WIPW estimator, that denominators in (2) should be constructed using $\pi_{\tau_i,1}^j$ and $\pi_{\tau_i+v,2}^{j(s)}$, $s=0,1$. Likewise, in forming the weight for such a subject, in (B.20) use $\pi_{\tau_i,1}^j$ and $\pi_{\tau_i+v,2}^{j(s)}$, $s=0,1$, and take

$$\widehat{\nu}_t^j = N_t^{-1}\sum_{i=1}^{N} \frac{\Delta_{t-1,i} C_i^j (Y_i - \widetilde{\theta}_t^j)^2}{I(R_i=0)\pi_{\tau_i+v,2}^{j(0)}\pi_{\tau_i,1}^j + I(R_i=1)\pi_{\tau_i+v,2}^{j(1)}\pi_{\tau_i,1}^j},$$

$$\widehat{\nu}_{t,1}^{j} = N_t^{-1} \sum_{i=1}^{N} \frac{\Delta_{t-1,i} C_i^j \{Y_i - \widehat{Q}_{1,\tau_i+v}^j(\boldsymbol{X}_{1,i})\}^2}{I(R_i = 0)\pi_{\tau_i+v,2}^{j(0)}\pi_{\tau_i,1}^{j} + I(R_i = 1)\pi_{\tau_i+v,2}^{j(1)}\pi_{\tau_i,1}^{j}},$$

$$\widehat{\nu}_{t,2}^{j(s)} = N_t^{-1} \sum_{i=1}^{N} \frac{\Delta_{t-1,i} C_i^j I(R_i = r)\{Y_i - \widehat{Q}_{2,\tau_i+v}^j(\overline{\boldsymbol{X}}_{2,i})\}^2}{\pi_{\tau_i+v,2}^{j(s)}\pi_{i,1}^{j}}, \quad s = 0, 1.$$

Verifying Assumptions for the WIPW and WAIPW Estimators

First, denote the following estimating functions corresponding to the WIPW and WAIPW estimators, respectively:

$$M_t^{WIPW,j}(\overline{\boldsymbol{X}}_{K,t}, \overline{\boldsymbol{A}}_{K,t}, Y_t; \theta^j) = \frac{W_t^j C_t^j}{\{\prod_{k=2}^{K} \pi_{t,k}^j(\overline{\boldsymbol{X}}_{k,t})\}\pi_{t,1}^j(\boldsymbol{X}_{1,t})}(Y_t - \theta^j),$$

$$M_t^{WAIPW,j}(\overline{\boldsymbol{X}}_{2,t}, \overline{\boldsymbol{A}}_{2,t}, Y_t; \theta^j) = W_t^{A,j} \left[\frac{C_t^j Y_t}{\pi_{t,2}^j(\overline{\boldsymbol{X}}_{2,t})\pi_{t,1}^j(\boldsymbol{X}_{1,t})} - \left\{ \frac{\overline{C}_{1,t}^j}{\pi_{t,1}^j(\boldsymbol{X}_{1,t})} - 1 \right\} \widehat{Q}_{1,t}^j(\boldsymbol{X}_{1,t}) \right.$$
$$\left. - \left\{ \frac{C^j}{\pi_{t,2}^j(\overline{\boldsymbol{X}}_{2,t})\pi_{t,1}^j(\boldsymbol{X}_{1,t}1)} - \frac{\overline{C}_{1,t}^j}{\pi_{t,1}^j(\boldsymbol{X}_{1,t})} \right\} \widehat{Q}_{2,t}^j(\overline{\boldsymbol{X}}_{2,t}) - \theta^j \right].$$

Note that these are estimating $\theta^j \in \mathbb{R}$, so $p = 1$. To reduce the notational burden, we use the short-hand notation $M_t^{WIPW,j}(\theta^j)$ and $M_t^{WAIPW,j}(\theta^j)$. Additionally, denote $\mathcal{M}_t^{WIPW,j}(\theta^j) = \sum_{t=1}^{T} M_t^{WIPW,j}(\theta^j)$ and $\mathcal{M}_t^{WAIPW,j}(\theta^j) = \sum_{t=1}^{T} M_t^{WAIPW,j}(\theta^j)$. We introduce the notation θ^{j*} to denote the true parameter, whereas θ^j for a generic value.

We now consider assumptions (A0)-(A8) in Web Appendix B and demonstrate that each assumption holds for the foregoing estimating functions.

For the first assumption (A0), we verify that $\{\mathcal{M}_t^{WIPW,j}(\theta^{j*})\}_{t\geqslant 1}$ and $\{\mathcal{M}_t^{WAIPW,j}(\theta^{j*})\}_{t\geqslant 1}$ are a Martingale difference sequences with respect to $\{\mathcal{F}_t\}_{t\geqslant 1}$ where $\mathcal{F}_t = \mathfrak{D}_t$. As shown in previous sections, $E\left\{M_t^{WIPW}(\theta^{j*}) \mid \mathfrak{D}_{t-1}\right\} = 0$ and $E\left\{M_t^{WAIPW}(\theta^{j*}) \mid \mathfrak{D}_{t-1}\right\} = 0$, which implies that both are Martingale difference sequences.

Assumption (A1) is that $\mathcal{M}(\theta)$ is continuously differentiable with respect to θ is invertible.

We trivially assume that $C_t^j \neq 0$ for all t. Consider the derivatives:

$$\nabla_\theta \mathcal{M}_T^{WIPW,j}(\theta^j) = -\sum_{t=1}^{T} \frac{W_t^j C_t^j}{\{\prod_{k=2}^K \pi_{t,k}^j(\overline{\boldsymbol{X}}_{k,t})\}\pi_{t,1}^j(\boldsymbol{X}_{1,t})},$$
$$\nabla_\theta \mathcal{M}_T^{WIPW,j}(\theta^j) = -\sum_{t=1}^{T} W_t^{A,j}.$$

The positivity assumption implies $\{\prod_{k=2}^K \pi_{t,k}^j(\overline{\boldsymbol{X}}_{k,t})\}\pi_{t,1}^j(\boldsymbol{X}_{1,t}) > 0$ for all t. By construction, $W_t^j \neq 0$ and $W_t^{A,j} \neq 0$ for all t (e.g., burn-in period), which verifies (A1).

Assumption (A2) states that $\lambda_{min}\left[\sum_{t=1}^T \text{var}\{M_t(\theta^*) \mid \mathcal{F}_{t-1}\}\right] \longrightarrow \infty$. Because we are estimating a scalar θ, this reduces to $\sum_{t=1}^T \text{var}\{M_t(\theta^*) \mid \mathcal{F}_{t-1}\} \longrightarrow \infty$. Because of how we choose the weights, $E\left[\{M_t^{WIPW,j}(\theta^{j*})\}^2 \mid \mathfrak{D}_{t-1}\right] = \sigma > 0$ and likewise for $E\left[\{M_t^{WAIPW,j}(\theta^{j*})\}^2 \mid \mathfrak{D}_{t-1}\right]$. Hence, (A2) holds for both estimators. As mentioned in Section 2.3 of the main paper, a clipping constant may be necessary to ensure the positivity assumption. The positivity assumption implies $E(C_t^j \mid \mathcal{W}^*, \mathfrak{D}_{t-1}) > 0$ with probability one for all t, which is necessary for (A2) to hold.

Assumption (A3) states:

$$\sup_{\theta \in \boldsymbol{\Theta}} \left|\left| \left\{\xi_T^{-1/2}(\theta^*)\nabla_\theta \mathcal{M}_T(\theta)\xi_T^{-1/2}(\theta^*)\right\}^{-1} \right|\right| = O_P(1).$$

Let $I_t^{WIPW,j}(\epsilon) = I(W_t^j C_t^j/\{\prod_{k=2}^K \pi_{t,k}^j(\overline{\boldsymbol{X}}_{k,t})\}\pi_{t,1}^j(\boldsymbol{X}_{1,t}) > \epsilon)$. With the positivity assumption, the trivial assumption that $C_j^t \neq 0$ for all t, and because $W_t^j > 0$ as long as $Y_t \neq \widetilde{\theta}^j$, which occurs infinitely often in non-degenerate distributions on Y, we can use the filtration Borel-Cantelli Lemma to determine there exists a $\theta > 0, \tau > 0$ such that $P\{I^{WIPW,j}(\epsilon) \mid \mathfrak{D}_{t-1}\} > \tau$. This implies $-\sum_{t=1}^T W_t^j C_t^j/\{\prod_{k=2}^K \pi_{t,k}^j(\overline{\boldsymbol{X}}_{k,t})\}\pi_{t,1}^j(\boldsymbol{X}_{1,t}) = O_P(T)$. Define $I^{WAIPW,j}(\epsilon) = I(W_t^{A,j} > \epsilon)$ and take similar steps to determine $-\sum_{t=1}^T W_t^{A,j} = O_P(T)$.

it holds that $W_t^j C_t^j/\{\prod_{k=2}^K \pi_{t,k}^j(\overline{\boldsymbol{X}}_{k,t})\}\pi_{t,1}^j(\boldsymbol{X}_{1,t}) > 0$ and $W_t^{A,j} > 0$ occur with non-zero probability.

For the WIPW estimator,

$$\sup_{\theta \in \boldsymbol{\Theta}} \left|\left| \left\{ \xi_T^{-1/2}(\theta^*) \nabla_\theta \mathcal{M}_t(\theta) \xi_T^{-1/2}(\theta^*) \right\}^{-1} \right|\right| = \left|\left| \frac{\sum_{t=1}^T E\left[\{M_t^{WIPW,j}(\theta^{j*})\}^2 \mid \mathfrak{D}_{t-1} \right]}{-\sum_{t=1}^T W_t^j C_t^j / \{\prod_{k=2}^K \pi_{t,k}^j(\overline{\boldsymbol{X}}_{k,t})\} \pi_{t,1}^j(\boldsymbol{X}_{1,t})} \right|\right|$$

$$= \left|\left| \frac{T\sigma}{O_P(T)} \right|\right|$$

$$= \left|\left| \frac{O_P(T)}{O_P(T)} \right|\right|$$

$$= O_P(1),$$

and similarly, for the WAIPW estimator,

$$\sup_{\theta \in \boldsymbol{\Theta}} \left|\left| \left\{ \xi_T^{-1/2}(\theta^*) \nabla_\theta \mathcal{M}_t(\theta) \xi_T^{-1/2}(\theta^*) \right\}^{-1} \right|\right| = \left|\left| \frac{\sum_{t=1}^T E\left[\left\{ M_t^{WAIPW,j}(\theta^{j*}) \right\}^2 \mid \mathfrak{D}_{t-1} \right]}{-\sum_{i=1} W_t^{A,j}} \right|\right|$$

$$= \left|\left| \frac{T\sigma}{O_P(T)} \right|\right|$$

$$= \left|\left| \frac{O_P(T)}{O_P(T)} \right|\right|$$

$$= O_P(1).$$

Assumption (A4) states: $\xi_T^{-1/2}(\theta^*)\mathcal{M}_T(\theta^*) = O_P(1)$.

For the WIPW estimator

$$\xi_T^{-1/2}(\theta^*)\mathcal{M}_T(\theta^*) = \frac{\sum_{t=1}^T M_t^{WIPW,j}(\theta^{j*})}{\left(\sum_{t=1}^T E\left[\left\{ M_t^{WIPW,j}(\theta^{j*}) \right\}^2 \mid \mathfrak{D}_{t-1} \right] \right)^{1/2}}$$

$$= \frac{T^{-1}\sum_{t=1}^T M_t^{WIPW,j}(\theta^{j*})}{T^{-1} \left(\sum_{t=1}^T E\left[\left\{ M_t^{WIPW,j}(\theta^{j*}) \right\}^2 \mid \mathfrak{D}_{t-1} \right] \right)^{1/2}}$$

$$= \frac{T^{-1}\sum_{t=1}^T M_t^{WIPW,j}(\theta^{j*})}{\sigma^{1/2}}$$

$$= O_P(1),$$

because $T^{-1}\sum_{t=1}^T M_t^{WIPW,j}(\theta^{j*}) \xrightarrow{p} 0$, a result of the strong law of large numbers for Martingale difference sequences (Sheu and Yao, 1984).

Similarly, for the WAIPW estimator,

$$\begin{aligned}
\xi_T^{-1/2}(\theta^*)\mathcal{M}_T(\theta^*) &= \frac{\sum_{t=1}^T M_t^{WAIPW,j}(\theta^{j*})}{\left(\sum_{t=1}^T E\left[\{M_t^{WIPW,j}(\theta^j)\}^2 \mid \mathfrak{D}_{t-1}\right]\right)^{1/2}} \\
&= \frac{T^{-1}\sum_{t=1}^T M_t^{WAIPW,j}(\theta^{j*})}{T^{-1}\left(\sum_{t=1}^T E\left[\{M_t^{WIPW,j}(\theta^j)\}^2 \mid \mathfrak{D}_{t-1}\right]\right)^{1/2}} \\
&= \frac{T^{-1}\sum_{t=1}^T M_t^{WAIPW,j}(\theta^{j*})}{\sigma^{1/2}} \\
&= O_P(1).
\end{aligned}$$

Assumption (A5) states $\xi_T^{-1/2}(\theta^*)\mathcal{M}_T(\theta^*) \xrightarrow{D} \mathcal{N}(0,1)$. Because $M_t^{WIPW,j}(\theta^{j*})$ and $M_t^{WAIPW,j}(\theta^{j*})$ are Martingale difference sequences (A0), and $\Sigma_{t=1}^T E\left[\{M_t^{WIPW,j}(\theta^{j*})\}^2 \mid \mathfrak{D}_{t-1}\right] \longrightarrow \infty$ with probability one, and likewise for WAIPW (A2) the Martingale central limit theorem (Hall and Heyde, 1980) guarantees:

$$\frac{\sum_{t=1}^T M_t^{WIPW,j}(\theta^{j*})}{\left(\sum_{t=1}^T E\left[\{M_t^{WIPW,j}(\theta^j)\}^2 \mid \mathfrak{D}_{t-1}\right]\right)^{1/2}} \xrightarrow{D} \mathcal{N}(0,1),$$
$$\frac{\sum_{t=1}^T M_t^{WAIPW,j}(\theta^{j*})}{\left(\sum_{t=1}^T E\left[\{M_t^{WAIPW,j}(\theta^j)\}^2 \mid \mathfrak{D}_{t-1}\right]\right)^{1/2}} \xrightarrow{D} \mathcal{N}(0,1).$$

Assumption (A6) states for any consistent estimator: $\widetilde{\theta}_T \xrightarrow{p} \theta^*$

$$\left|\left|\xi_T^{-1/2}(\theta^*)\left\{\nabla_\theta\mathcal{M}_T(\widetilde{\theta}_T) - \nabla_\theta\mathcal{M}_T(\theta^*)\right\}\xi_T^{-1/2}(\theta^*)\right|\right| \xrightarrow{p} 0.$$

For both WIPW and WAIPW, $\nabla_\theta\mathcal{M}_T(\widetilde{\theta}_T^j) = \nabla_\theta\mathcal{M}_T(\theta^{j*})$, so this holds.

Assumption (A7) states that there exists fixed and positive value σ' such that

$$-\xi_T^{-1/2}(\theta^*)\nabla_\theta\mathcal{M}_T(\theta^*)\xi_T^{-1/2}(\theta^*) \xrightarrow{p} \sigma',$$

Due to the Martingale convergence theorem, there exists some $\tau < \infty$ such that the following hold: $T^{-1}\sum_{t=1}^T E\left[\{M_t^{WIPW,j}(\theta^{j*})\}^2 \mid \mathfrak{D}_{t-1}\right] \xrightarrow{p} \sigma$ and $T^{-1}\sum_{t=1}^T W_t^{A,j} \xrightarrow{p} \tau$.

For the WIPW estimator,

$$\begin{aligned}
-\xi_T^{-1/2}(\theta^*)\nabla_\theta \mathcal{M}_T(\theta^*)\xi_T^{-1/2}(\theta^*) &= \frac{T^{-1}\sum_{t=1}^T W_t^j C_t^j / \{\prod_{k=2}^K \pi_{t,k}^j(\overline{\boldsymbol{X}}_{k,t})\}\pi_{t,1}^j(\boldsymbol{X}_{1,t})}{T^{-1}\sum_{t=1}^T E\left[\{M_t^{WIPW,j}(\theta^{j*})\}^2 \mid \mathfrak{D}_{t-1}\right]} \\
&= \frac{T^{-1}\sum_{t=1}^T W_t^j C_t^j / \{\prod_{k=2}^K \pi_{t,k}^j(\overline{\boldsymbol{X}}_{k,t})\}\pi_{t,1}^j(\boldsymbol{X}_{1,t})}{\sigma} \\
&\xrightarrow{p} \frac{\tau}{\sigma} \\
&> 0.
\end{aligned}$$

Similarly, for the WAIPW estimator,

$$\begin{aligned}
-\xi_T^{-1/2}(\theta^*)\nabla_{\boldsymbol{\theta}} \mathcal{M}_T(\theta^*)\xi_T^{-1/2}(\theta^*) &= \frac{T^{-1}\sum_{t=1}^T W_t^{A,j}}{T^{-1}\sum_{t=1}^T E\left[\{M_t^{WAIPW,j}(\theta^{j})\}^2 \mid \mathfrak{D}_{t-1}\right]} \\
&= \frac{T^{-1}\sum_{t=1}^T W_t^{A,j}}{\sigma} \\
&\xrightarrow{p} \frac{\tau'}{\sigma} \\
&> 0
\end{aligned}$$

Assumption (A8) states $\widehat{\theta}_T$ satisfies $\xi_T^{-1/2}(\theta^*)\mathcal{M}_T(\widehat{\theta}_T) \xrightarrow{p} 0$ as $T \to \infty$.

Both $E\left\{M_t^{WIPW,j}(\widehat{\theta}_t^{WIPW,j})\right\} = 0$ and $E\left\{M_t^{WAIPW,j}(\widehat{\theta}_t^{WAIPW,j})\right\} = 0$.

For WIPW, due to the the strong law of large numbers of Martingale differences:

$$\begin{aligned}
\xi_T^{-1/2}(\theta^*)\mathcal{M}_T(\widehat{\theta}_T) &= \frac{T^{-1}\mathcal{M}_t^{WIPW,j}(\widehat{\theta}_t^j)}{T^{-1}\sum_{t=1}^T E\left[\left\{M_t^{WIPW,j}(\widehat{\theta}_t^{WIPW,j})\right\}^2 \mid \mathfrak{D}_{t-1}\right]} \\
&\xrightarrow{p} 0/\sigma
\end{aligned}$$

and likewise for WAIPW:

$$\begin{aligned}
\xi_T^{-1/2}(\theta^*)\mathcal{M}_T(\widehat{\theta}_T) &= \frac{T^{-1}\mathcal{M}_t^{WAIPW,j}(\widehat{\theta}_t^j)}{T^{-1}\sum_{t=1}^T E\left[\left\{M_t^{WAIPW,j}(\widehat{\theta}_t^{WAIPW,j})\right\}^2 \mid \mathfrak{D}_{t-1}\right]} \\
&\xrightarrow{p} 0/\sigma
\end{aligned}$$

References

Almirall, D., Nahum-Shani, I., Sherwood, N. E., and Murphy, S. A. (2014). Introduction to SMART designs for the development of adaptive interventions: with application to weight loss research. *Translational Behovioral Medicine* **4,** 260–274.

Cheung, Y. K., Chakraborty, B., and Davidson, K. W. (2014). Sequential multiple assignment randomized trial (SMART) with adaptive randomization for quality improvement in depression treatment program. *Biometrics* **71,** 450–459.

Godambe, V. P. (1985). The foundations of finite sample estimation in stochastic processes. *Biometrika* **72,** 419–428.

Godambe, V. P. and Heyde, C. C. (2010). Quasi-likelihood and optimal estimation. *International Statistical Review* **55,** 386–399.

Hall, P. and Heyde, C. C. (1980). *Martingale Limit Theory and its Application.* Academic Press, New York.

Heyde, C. C. (1997). *Quasi-Likelihood and Its Application: A General Approach to Optimal Parameter Estimation.* Springer, New York.

Khoury, K., Meisel, J. L., Yau, C., Rugo, H. S., Nanda, R., Davidian, M., and et al. (2024). Datopotamab–deruxtecan in early-stage breast cancer: the sequential multiple assignment randomized I-SPY2.2 phase 2 trial. *Nature Medicine,* in press, https://doi.org/10.1038/s41591-024-03266-2.

Manschot, C., Laber, E., and Davidian, M. (2023). Interim monitoring of sequential multiple assignment randomized trials using partial information. *Biometrics* **79,** 2881–2894.

Shatsky, R. A., Trivedi, M. A., Yau, C., Nanda, R., Rugo, H., Davidian, M., and et al. (2024). Datopotamab–deruxtecan plus durvalumab in early-stage breast cancer: the sequential multiple assignment randomized I-SPY2.2 phase 2 trial. *Nature Medicine,* in press, https://doi.org/10.1038/s41591-024-03267-1.

Sheu, S. S. and Yao, Y. S. (1984). A strong law of large numbers for martingales. *Proceedings of the American Mathematical Society* **92,** 283–287.

Tsiatis, A. A. (2006). *Semiparametric Theory and Missing Data.* Springer, New York.

Tsiatis, A. A., Davidian, M., Holloway, S., and Laber, E. B. (2020). *Dynamic Treatment Regimes: Statistical Methods for Precision Medicine.* Chapman and Hall/CRC Press, Boca Raton, Florida.

Table A.1

Simulation results using up-front RAR based on TS for 5000 Monte Carlo (MC) replications for the scenario in Section 5.1 of the main paper, $N = 325$. Columns indicate the randomization method: WAIPW(0.5) is TS based on the WAIPW estimator with $c_t = 0.5$ for all t and AIPW(1) uses $c_t = 1$ for all t; WIPW(c_t) and IAIPW(c_t) are defined similarly. SR denotes simple, uniform randomization. Mean Y denotes the MC average mean outcome for the 1000 individuals in the trial; lower mean outcomes are more favorable. Proportion A_1 Opt is the MC average proportion of subjects assigned the optimal treatment at the first stage; Proportion Regime Opt is the MC average proportion of subjects who were consistent with following the optimal regime $\mathbf{d}^8$ in the trial. For estimation results, $\mathbf{d}^8$ Est Opt denotes the proportion of trials we correctly estimate $\mathbf{d}^8$ to be the optimal regime; $\mathbf{d}^7$ or $\mathbf{d}^8$ Est Opt is the proportion of trials we estimate either regime 7 or 8 to be the optimal regime; and $\mathcal{V}(\mathbf{d}^8)$ MSE is the MC mean squared error for regime 8. For the estimation results, the term in parentheses, e.g., (IPW), denotes the estimator used after the trial is completed. $\mathcal{V}(\mathbf{d}^8)$ 95% CI is the MC proportion of 95% confidence intervals that cover the true value; the term in parentheses is the estimator used to construct the confidence interval. $\mathcal{V}(\mathbf{d}^8)$ 95% LB for lower confidence bounds $\mathcal{V}(\mathbf{d}^8)$ 95% UB for upper confidence bounds are defined similarly. Bold values indicate the most favorable result among the randomization methods. Standard deviations of entries are in parentheses.

	SR	WIPW(0.5)	WIPW(1)	WAIPW(0.5)	WAIPW(1)	IAIPW(0.5)	IAIPW(1)
In Trial							
Mean Y	-1.380 (0.001)	-1.626 (0.001)	-1.740 (0.001)	-1.625 (0.001)	**-1.741** (0.001)	-1.621 (0.001)	-1.740 (0.001)
Proportion A_1 Opt	0.500 (0.000)	0.613 (0.000)	**0.667** (0.000)	0.613 (0.000)	0.666 (0.000)	0.612 (0.000)	0.666 (0.000)
Proportion Regime Opt	0.250 (0.000)	0.323 (0.001)	0.361 (0.001)	0.329 (0.001)	0.374 (0.001)	0.327 (0.001)	**0.377** (0.001)
Estimation							
$\mathbf{d}^8$ Est Opt (IPW)	0.366 (0.007)	0.353 (0.007)	0.354 (0.007)	**0.374** (0.007)	0.351 (0.007)	0.347 (0.007)	0.364 (0.007)
$\mathbf{d}^8$ Est Opt (WIPW)	0.338 (0.007)	0.356 (0.007)	0.357 (0.007)	0.372 (0.007)	0.365 (0.007)	0.355 (0.007)	**0.378** (0.007)
$\mathbf{d}^8$ Est Opt (AIPW)	0.410 (0.007)	0.420 (0.007)	0.410 (0.007)	**0.438** (0.007)	0.421 (0.007)	0.405 (0.007)	0.434 (0.007)
$\mathbf{d}^8$ Est Opt (WAIPW)	0.400 (0.007)	0.413 (0.007)	0.405 (0.007)	**0.432** (0.007)	0.409 (0.007)	0.398 (0.007)	0.423 (0.007)
$\mathbf{d}^7$ or $\mathbf{d}^8$ Est Opt (IPW)	0.678 (0.007)	0.683 (0.007)	0.665 (0.007)	0.674 (0.007)	0.656 (0.007)	0.665 (0.007)	**0.688** (0.007)
$\mathbf{d}^7$ or $\mathbf{d}^8$ Est Opt (WIPW)	0.630 (0.007)	0.686 (0.007)	0.678 (0.007)	0.679 (0.007)	0.677 (0.007)	0.675 (0.006)	**0.687** (0.007)
$\mathbf{d}^7$ or $\mathbf{d}^8$ Est Opt (AIPW)	0.725 (0.006)	0.740 (0.006)	0.726 (0.006)	0.739 (0.006)	0.728 (0.006)	0.731 (0.006)	**0.752** (0.006)
$\mathbf{d}^7$ or $\mathbf{d}^8$ Est Opt (WAIPW)	0.721 (0.006)	0.744 (0.006)	0.732 (0.006)	0.744 (0.006)	0.737 (0.006)	0.731 (0.006)	**0.756** (0.006)
$\mathcal{V}(\mathbf{d}^8)$ MSE $\times 10^2$ (IPW)	25.995 (0.527)	**21.792** (0.446)	24.250 (0.632)	22.795 (0.464)	23.745 (0.548)	22.204 (0.470)	23.739 (0.591)
$\mathcal{V}(\mathbf{d}^8)$ MSE $\times 10^2$ (WIPW)	49.423 (1.001)	21.012 (0.427)	21.786 (0.514)	21.867 (0.451)	21.098 (0.457)	21.201 (0.449)	**20.635** (0.470)
$\mathcal{V}(\mathbf{d}^8)$ MSE $\times 10^2$ (AIPW)	17.044 (0.350)	15.215 (0.307)	16.172 (0.393)	15.093 (0.307)	15.959 (0.359)	**15.053** (0.308)	15.373 (0.367)
$\mathcal{V}(\mathbf{d}^8)$ MSE $\times 10^2$ (WAIPW)	17.354 (0.358)	14.936 (0.303)	15.139 (0.337)	14.902 (0.308)	15.158 (0.322)	14.843 (0.305)	**14.432** (0.323)
Coverage							
$\mathcal{V}(\mathbf{d}^8)$ 95% CI (IPW)	0.942 (0.003)	0.950 (0.003)	0.945 (0.003)	0.943 (0.003)	0.945 (0.003)	0.950 (0.003)	0.948 (0.003)
$\mathcal{V}(\mathbf{d}^8)$ 95% CI (WIPW)	0.939 (0.003)	0.947 (0.003)	0.945 (0.003)	0.940 (0.003)	0.941 (0.003)	0.950 (0.003)	0.947 (0.003)
$\mathcal{V}(\mathbf{d}^8)$ 95% CI (AIPW)	0.941 (0.003)	0.945 (0.003)	0.942 (0.003)	0.944 (0.003)	0.947 (0.003)	0.949 (0.003)	0.947 (0.003)
$\mathcal{V}(\mathbf{d}^8)$ 95% CI (WAIPW)	0.941 (0.003)	0.943 (0.003)	0.939 (0.003)	0.941 (0.003)	0.945 (0.003)	0.946 (0.003)	0.945 (0.003)
$\mathcal{V}(\mathbf{d}^8)$ 95% LB (IPW)	0.944 (0.003)	0.959 (0.003)	0.953 (0.003)	0.949 (0.003)	0.952 (0.003)	0.942 (0.003)	0.950 (0.003)
$\mathcal{V}(\mathbf{d}^8)$ 95% LB (WIPW)	0.944 (0.003)	0.954 (0.003)	0.949 (0.003)	0.943 (0.003)	0.948 (0.003)	0.939 (0.003)	0.948 (0.003)
$\mathcal{V}(\mathbf{d}^8)$ 95% LB (AIPW)	0.942 (0.003)	0.950 (0.003)	0.946 (0.003)	0.947 (0.003)	0.948 (0.003)	0.943 (0.003)	0.947 (0.003)
$\mathcal{V}(\mathbf{d}^8)$ 95% LB (WAIPW)	0.943 (0.003)	0.949 (0.003)	0.941 (0.003)	0.941 (0.003)	0.943 (0.003)	0.943 (0.003)	0.942 (0.003)
$\mathcal{V}(\mathbf{d}^8)$ 95% UB (IPW)	0.945 (0.003)	0.944 (0.003)	0.947 (0.003)	0.946 (0.003)	0.942 (0.003)	0.951 (0.003)	0.944 (0.003)
$\mathcal{V}(\mathbf{d}^8)$ 95% UB (WIPW)	0.943 (0.003)	0.944 (0.003)	0.950 (0.003)	0.942 (0.003)	0.945 (0.003)	0.952 (0.003)	0.949 (0.003)
$\mathcal{V}(\mathbf{d}^8)$ 95% UB (AIPW)	0.946 (0.003)	0.939 (0.003)	0.946 (0.003)	0.946 (0.003)	0.944 (0.003)	0.950 (0.003)	0.948 (0.003)
$\mathcal{V}(\mathbf{d}^8)$ 95% UB (WAIPW)	0.946 (0.003)	0.942 (0.003)	0.945 (0.003)	0.945 (0.003)	0.944 (0.003)	0.948 (0.003)	0.952 (0.003)

Table A.2

Simulation results using sequential RAR based on TS for 5000 Monte Carlo replications for the scenario in Section 5.2 of the main paper, $N = 325$. Columns indicate the randomization method: TS(0.25) is TS via Q-learning with $c_t = 0.25$ for all t, TS(0.50), TS(0.75), and TS(1) are defined similarly. AR-1 is the conservatively-tuned version of SMART-AR and AR-2 is the more aggressive version. SR denotes simple, uniform randomization. All entries are defined as in Table A.1.

	SR	TS(0.25)	TS(0.50)	TS(0.75)	TS(1)	AR-1	AR-2
In Trial							
Mean Y	-1.380 (0.001)	-1.654 (0.002)	-1.691 (0.002)	-1.711 (0.002)	**-1.723** (0.002)	-1.700 (0.001)	-1.694 (0.001)
Proportion A_1 Opt	0.500 (0.000)	0.624 (0.001)	0.639 (0.001)	0.648 (0.001)	**0.653** (0.001)	**0.653** (0.000)	**0.653** (0.000)
Proportion Regime Opt	0.250 (0.000)	0.330 (0.001)	0.353 (0.001)	0.370 (0.001)	**0.378** (0.001)	0.306 (0.001)	0.289 (0.001)
Estimation							
$\mathbf{d}^8$ Est Opt (IPW)	0.366 (0.007)	0.375 (0.007)	0.382 (0.007)	0.368 (0.007)	0.348 (0.007)	**0.384** (0.007)	0.373 (0.007)
$\mathbf{d}^8$ Est Opt (WIPW)	0.338 (0.007)	0.382 (0.007)	**0.390** (0.007)	0.375 (0.007)	0.362 (0.007)	0.388 (0.007)	0.375 (0.007)
$\mathbf{d}^8$ Est Opt (AIPW)	0.410 (0.007)	0.415 (0.007)	0.423 (0.007)	0.407 (0.007)	0.417 (0.007)	**0.428** (0.007)	0.422 (0.007)
$\mathbf{d}^8$ Est Opt (WAIPW)	0.400 (0.007)	0.411 (0.007)	0.421 (0.007)	0.413 (0.007)	0.372 (0.007)	**0.437** (0.007)	0.432 (0.007)
$\mathbf{d}^7$ or $\mathbf{d}^8$ Est Opt (IPW)	0.678 (0.007)	0.667 (0.007)	0.675 (0.007)	0.670 (0.007)	0.643 (0.007)	**0.683** (0.007)	0.670 (0.007)
$\mathbf{d}^7$ or $\mathbf{d}^8$ Est Opt (WIPW)	0.630 (0.007)	0.681 (0.007)	0.690 (0.007)	0.692 (0.007)	0.676 (0.007)	**0.691** (0.007)	0.679 (0.007)
$\mathbf{d}^7$ or $\mathbf{d}^8$ Est Opt (AIPW)	0.725 (0.006)	0.734 (0.006)	0.736 (0.006)	0.732 (0.006)	0.696 (0.007)	0.745 (0.006)	**0.746** (0.006)
$\mathbf{d}^7$ or $\mathbf{d}^8$ Est Opt (WAIPW)	0.721 (0.006)	0.737 (0.006)	0.743 (0.006)	**0.751** (0.006)	0.724 (0.006)	0.745 (0.006)	**0.751** (0.006)
$\mathcal{V}(\mathbf{d}^8)$ MSE $\times 10^2$ (IPW)	25.995 (0.527)	**21.063** (0.447)	21.975 (0.479)	23.712 (0.572)	27.351 (0.848)	23.555 (0.485)	29.626 (0.680)
$\mathcal{V}(\mathbf{d}^8)$ MSE $\times 10^2$ (WIPW)	49.423 (1.001)	**13.944** (0.327)	14.614 (0.316)	15.653 (0.369)	17.421 (0.634)	14.974 (0.302)	18.487 (0.414)
$\mathcal{V}(\mathbf{d}^8)$ MSE $\times 10^2$ (AIPW)	17.044 (0.350)	**13.944** (0.327)	14.641 (0.316)	15.653 (0.369)	17.421 (0.495)	14.974 (0.302)	18.487 (0.414)
$\mathcal{V}(\mathbf{d}^8)$ MSE $\times 10^2$ (WAIPW)	17.354 (0.358)	**13.674** (0.286)	14.326 (0.312)	15.192 (0.338)	16.468 (0.609)	14.832 (0.300)	17.759 (0.389)
Coverage							
$\mathcal{V}(\mathbf{d}^8)$ 95% CI (IPW)	0.942 (0.003)	0.949 (0.003)	0.941 (0.003)	0.945 (0.003)	0.939 (0.003)	0.943 (0.003)	0.940 (0.003)
$\mathcal{V}(\mathbf{d}^8)$ 95% CI (WIPW)	0.955 (0.003)	0.948 (0.003)	0.942 (0.003)	0.944 (0.003)	0.940 (0.003)	0.945 (0.003)	0.939 (0.003)
$\mathcal{V}(\mathbf{d}^8)$ 95% CI (AIPW)	0.941 (0.003)	0.950 (0.003)	0.943 (0.003)	0.946 (0.003)	0.942 (0.003)	0.950 (0.003)	0.939 (0.003)
$\mathcal{V}(\mathbf{d}^8)$ 95% CI (WAIPW)	0.941 (0.003)	0.948 (0.003)	0.943 (0.003)	0.942 (0.003)	0.938 (0.003)	0.946 (0.003)	0.935 (0.003)
$\mathcal{V}(\mathbf{d}^8)$ 95% LB (IPW)	0.944 (0.003)	0.947 (0.003)	0.951 (0.003)	0.947 (0.003)	0.958 (0.003)	0.949 (0.003)	0.939 (0.003)
$\mathcal{V}(\mathbf{d}^8)$ 95% LB (WIPW)	0.944 (0.003)	0.945 (0.003)	0.948 (0.003)	0.942 (0.003)	0.944 (0.003)	0.949 (0.003)	0.943 (0.003)
$\mathcal{V}(\mathbf{d}^8)$ 95% LB (AIPW)	0.942 (0.003)	0.944 (0.003)	0.946 (0.003)	0.947 (0.003)	0.948 (0.003)	0.943 (0.003)	0.939 (0.003)
$\mathcal{V}(\mathbf{d}^8)$ 95% LB (WAIPW)	0.943 (0.003)	0.944 (0.003)	0.942 (0.003)	0.942 (0.003)	0.939 (0.003)	0.944 (0.003)	0.941 (0.003)
$\mathcal{V}(\mathbf{d}^8)$ 95% UB (IPW)	0.945 (0.003)	0.953 (0.003)	0.942 (0.003)	0.941 (0.003)	0.941 (0.003)	0.948 (0.003)	0.944 (0.003)
$\mathcal{V}(\mathbf{d}^8)$ 95% UB (WIPW)	0.943 (0.003)	0.946 (0.003)	0.945 (0.003)	0.945 (0.003)	0.943 (0.003)	0.947 (0.003)	0.943 (0.003)
$\mathcal{V}(\mathbf{d}^8)$ 95% UB (AIPW)	0.946 (0.003)	0.958 (0.003)	0.949 (0.003)	0.949 (0.003)	0.944 (0.003)	0.952 (0.003)	0.948 (0.003)
$\mathcal{V}(\mathbf{d}^8)$ 95% UB (WAIPW)	0.946 (0.003)	0.957 (0.003)	0.951 (0.003)	0.946 (0.003)	0.944 (0.003)	0.949 (0.003)	0.942 (0.003)
