# Supplementary material for: Adaptive randomization methods for sequential multiple assignment randomized trials (smarts) via thompson sampling
Source: Biometrics. 2024 Dec 16;80(4):ujae152. doi: 10.1093/biomtc/ujae152 (PMC11647911; doi:10.1093/biomtc/ujae152)
Supplement: ujae152_Supplemental_Files — Web Appendices A–C, referenced in Sections 3–5, and code to implement the simulations, are available with this paper at the Biometrics website on Oxford Academic. [file ujae152_supplemental_files.zip › WebAppendixC_Norwood_etal_R1.pdf]

# Web Appendix C: Adapative Randomization Methods for Sequential Multiple Assignment Randomized Trials (SMARTs) via Thompson Sampling

Peter Norwood, Marie Davidian, Eric Laber

## C.1 Cancer Pain Management SMART, Continuous Outcome, Different Mean Outcomes

### C.1.1 Simulation Details

This is the scenario presented in the main paper, but with only selected results. We present results of a simulation study involving 5000 Monte Carlo trials under a scenario mimicking the cancer pain SMART introduced in Section 5 of the main paper.

Each trial enrolls $N = 1000$ subjects, with enrollment times uniform over (integer) weeks 1-24. Upon enrollment, we draw baseline pain score $X_1 \sim N(5,1)$ and assign stage 1 treatment $A_1 \in \square_1 = \{0,1\}$. Six weeks after $A_1$ is assigned, second-stage pain score is generated as $X_{2,1} = \gamma_{1,0} + \gamma_{1,1}X_1 + \gamma_{1,2}A_1 + \varepsilon_1$, where $\varepsilon_1 \sim N(0,1)$, and response status after the first stage is $X_{2,2} = I(X_{2,1} < 0.7X_1)$, which, with $A_1$, dictates the feasible subset of $\square_2 = \{0,1,2,3,4,5\}$ from which stage 2 treatment $A_2$ is assigned. Six weeks later, the outcome is generated as $Y = \gamma_{2,0} + \gamma_{2,1}X_1 + \gamma_{2,2}A_1 + \gamma_{2,3}X_{2,1} + \gamma_{2,4}I(A_2 = 1) + \gamma_{2,5}I(A_2 = 2 \text{ or } 5) + \gamma_{2,6}I(A_2 = 3) + \gamma_{2,7}I(A_2 = 4) + \varepsilon_2$, where $\varepsilon_2 \sim N(0,1)$. With $\gamma_1 = (\gamma_{1,0}, \gamma_{1,1}, \gamma_{1,2})^T = (0.00, 0.90, -1.50)^T$ and $\gamma_2 = (\gamma_{2,0}, \ldots, \gamma_{2,7}) = (0.00, 0.30, -0.75, 0.60, -0.25, -0.75, -0.75, -0.85)^T$, for the $m = 8$ embedded regimes defined in Figure 1, $\{\square(\mathbf{d}^1), \ldots, \square(\mathbf{d}^8)\} = (\theta_1, \ldots, \theta_8) = (-0.126, -0.374, -0.500, -0.251, -2.408, -2.401, -2.494, -2.501)$, so that regime 8 is optimal, as larger reductions in pain are desirable.

The burn-in period ends at the time $t^*$ when each of the $m = 8$ regimes has at least 25 subjects who have completed the trial with experience consistent with following the regime.

We compare the performance of up-front RAR using TS with $c_t = 0.25, 0.50, 0.75$ and 1 for all $t$ based on the IPW, WIPW, AIPW, WAIPW, and IAIPW estimators. For the AIPW, WAIPW and IAIPW estimators, $Q_2(\overline{\mathbf{X}_2}, \overline{\mathbf{a}_2}; \beta_2) = \beta_{2,0} + \beta_{2,1}x_1 + \beta_{2,2}I(a_1 = 1) + \beta_{2,3}x_{2,1} + \beta_{2,4}I(a_2 = 1) + \beta_{2,5}I(a_2 = 2 \text{ or } 5) + \beta_{2,6}I(a_2 = 3) + \beta_{2,7}I(a_2 = 4)$ and $Q_1^j(x_1, a_1; \beta_1^j) = \beta_{1,0}^j + \beta_{1,1}^j x_1 + \beta_{1,2}^j a_1 + \beta_{1,3}^j x_1 a_1$.

The sequential methods include Q-learning-based sequential RAR approach using TS with $c_t = 0.25, 0.50, 0.75$ and 1 to SR and two SMART-AR methods, a conservatively-tuned (AR-1) and a more aggressive (AR-2) version. To implement all RAR methods, we posit linear models $Q_2(\overline{\mathbf{X}_2}, \overline{\mathbf{a}_2}; \beta_2) = \beta_{2,0} + \beta_{2,1}x_1 + \beta_{2,2}I(a_1 = 1) + \beta_{2,3}x_{2,1} + \beta_{2,4}I(a_2 = 1) + \beta_{2,5}I(a_2 = 2 \text{ or } 5) + \beta_{2,6}I(a_2 = 3) + \beta_{2,7}I(a_2 = 4)$ and $Q_1(x_1, a_1; \beta_1) = \beta_{1,0} + \beta_{1,1}x_1 + \beta_{1,2}a_1$. For sequential RAR, we set $B_1 = b_2 \times b_1 = 32 \times 32 = 1024$ and $B_2 = 1000$. The tuning parameters for AR-1 are $b = 10, \tau = 0.5$ and $b = 100, \tau = 0.025$ for AR-2 and $\lambda_t = t^{-1}\tau^{(1-b)}$ for both. A clipping constant of 0.05 was imposed on all sequential methods.

At each week, for each RAR method, newly-enrolled subjects are assigned stage 1 treatment using the same randomization probability. Already-enrolled subjects who have reached stage 2 at this week and require stage 2 randomization are partitioned into four groups based on $(a_1, x_{2,2}) = (0,0), (0,1), (1,0), (1,1)$. Within each group, randomization probabilities are calculated; thus, second-stage probabilities are specific to each stage 1 treatment-response status combination. For both AR methods, we use the sample average of $X_1$ to calculate the the components that make up the stage 2 probabilities.

### C.1.2 In Trial Results

In the following table, for each randomization scheme, we report the Monte Carlo average of sample mean outcomes achieved by the 1000 subjects in each trial and its Monte Carlo standard error (multiplied by 1000), the sample mean proportion of the 1000 subjects that received the optimal first stage treatment and its Monte Carlo standard error, the sample mean proportion of the 1000 subjects consistent with the optimal regime and its Monte Carlo standard error, and the sample mean proportion of the 1000 subjects consistent with either regime 7 or 8 (two best) and its Monte Carlo standard error.

In Trial Results for the Cancer Pain Management SMART, Continuous Outcome, Different Mean Outcomes Scenario.

| Randomization Method | Mean Y | SE Mean Y x 10^3 | Mean Proportion A1=1 | SE Mean Proportion A1=1 x 10^3 | Mean Proportion Cd8=1 | SE Mean Proportion Cd8=1 x 10^3 | Mean Proportion Cd7=1 or Cd8=1 | SE Mean Proportion Cd7=1 or Cd8=1 x 10^3 |
|---|---|---|---|---|---|---|---|---|
| AIPW(0.25) | -1.605 | 0.824 | 0.604 | 0.254 | 0.328 | 0.476 | 0.349 | 0.476 |
| AIPW(0.5) | -1.793 | 0.797 | 0.690 | 0.228 | 0.403 | 0.974 | 0.426 | 0.971 |
| AIPW(0.75) | -1.941 | 0.801 | 0.759 | 0.207 | 0.467 | 1.479 | 0.492 | 1.472 |
| AIPW(1) | -1.994 | 0.782 | 0.782 | 0.199 | 0.491 | 1.712 | 0.516 | 1.704 |
| AR-1 | -1.950 | 0.787 | 0.775 | 0.202 | 0.320 | 1.038 | 0.349 | 0.998 |
| AR-2 | -1.957 | 0.773 | 0.775 | 0.196 | 0.351 | 0.582 | 0.380 | 0.556 |
| IAIPW(0.25) | -1.606 | 0.827 | 0.603 | 0.258 | 0.328 | 0.470 | 0.349 | 0.472 |
| IAIPW(0.5) | -1.793 | 0.810 | 0.690 | 0.239 | 0.402 | 0.983 | 0.425 | 0.978 |

| Randomization Method | Mean Y | SE Mean Y x 10^3 | Mean Proportion A1=1 | SE Mean Proportion A1=1 x 10^3 | Mean Proportion Cd8=1 | SE Mean Proportion Cd8=1 x 10^3 | Mean Proportion Cd7=1 or Cd8=1 | SE Mean Proportion Cd7=1 or Cd8=1 x 10^3 |
|---|---|---|---|---|---|---|---|---|
| IAIPW(0.75) | -1.943 | 0.800 | 0.758 | 0.201 | 0.465 | 1.445 | 0.490 | 1.438 |
| IAIPW(1) | -1.993 | 0.794 | 0.782 | 0.193 | 0.491 | 1.712 | 0.517 | 1.700 |
| IPW(0.25) | -1.607 | 0.839 | 0.605 | 0.250 | 0.322 | 0.500 | 0.343 | 0.501 |
| IPW(0.5) | -1.796 | 0.806 | 0.692 | 0.230 | 0.389 | 1.036 | 0.413 | 1.035 |
| IPW(0.75) | -1.940 | 0.786 | 0.759 | 0.199 | 0.444 | 1.531 | 0.469 | 1.528 |
| IPW(1) | -1.991 | 0.795 | 0.782 | 0.195 | 0.463 | 1.817 | 0.490 | 1.817 |
| SR | -1.380 | 0.793 | 0.500 | 0.223 | 0.250 | 0.195 | 0.267 | 0.201 |
| TS(0.25) | -1.976 | 1.347 | 0.772 | 0.594 | 0.445 | 0.921 | 0.471 | 0.933 |
| TS(0.50) | -1.999 | 1.283 | 0.780 | 0.556 | 0.491 | 1.396 | 0.516 | 1.398 |
| TS(0.75) | -2.014 | 1.232 | 0.785 | 0.521 | 0.517 | 1.708 | 0.541 | 1.700 |
| TS(1) | -2.026 | 1.127 | 0.790 | 0.458 | 0.538 | 1.925 | 0.561 | 1.909 |
| WAIPW(0.25) | -1.605 | 0.820 | 0.604 | 0.253 | 0.328 | 0.466 | 0.349 | 0.466 |
| WAIPW(0.5) | -1.794 | 0.788 | 0.691 | 0.236 | 0.401 | 0.956 | 0.425 | 0.952 |
| WAIPW(0.75) | -1.943 | 0.780 | 0.758 | 0.202 | 0.468 | 1.472 | 0.493 | 1.465 |
| WAIPW(1) | -1.996 | 0.776 | 0.782 | 0.195 | 0.498 | 1.720 | 0.524 | 1.717 |
| WIPW(0.25) | -1.608 | 0.820 | 0.605 | 0.249 | 0.322 | 0.498 | 0.343 | 0.499 |
| WIPW(0.5) | -1.795 | 0.798 | 0.691 | 0.232 | 0.390 | 1.017 | 0.414 | 1.016 |
| WIPW(0.75) | -1.942 | 0.772 | 0.759 | 0.204 | 0.447 | 1.558 | 0.473 | 1.555 |
| WIPW(1) | -1.992 | 0.790 | 0.782 | 0.197 | 0.470 | 1.821 | 0.497 | 1.816 |

### C.1.3 Estimation Results

In the following table, for each randomization method, regime, and estimator, we report the true value of the regime, the Monte Carlo mean estimate and its standard deviation, the mean normalized estimate and its standard deviation, the mean bias, the confidence interval coverage and its standard error (multiplied by 1000), the lower bound coverage and its standard error, the upper bound coverage and its standard error, the mean confidence interval length and its standard error, and the mean squared error (multiplied by 1000) and its standard error.

Estimation Results for the Cancer Pain Management SMART, Continuous Outcome, Different Mean Outcomes Scenario.

| Randomization Method | Regime | Estimator | True Value x 10^3 | Mean Estimate x 10^3 | SD Estimates x 10^3 | Mean Normalized | SD Normalized | Mean Bias | CI Coverage | SE CI Coverage x 10^3 | LB Coverage | SE LB Coverage x 10^3 | UB Coverage | SE UB Coverage x 10^3 | Mean CI Length | SE CI Length x 10^3 | MSE x 10^3 | SE MSE x 10^3 |
|---|---|---|---|---|---|---|---|---|---|---|---|---|---|---|---|---|---|---|
| AIPW(0.25) | 1 | IPW | -0.126 | -0.122 | 0.101 | 0.040 | 1.006 | 0.004 | 0.948 | 3.135 | 0.939 | 3.380 | 0.956 | 2.907 | 3.92 | 0 | 10.118 | 0.199 |
| | 1 | WIPW | -0.126 | -0.123 | 0.125 | 0.026 | 1.015 | 0.003 | 0.944 | 3.258 | 0.942 | 3.317 | 0.951 | 3.042 | 3.92 | 0 | 15.749 | 0.310 |
| | 1 | AIPW | -0.126 | -0.123 | 0.082 | 0.038 | 1.017 | 0.003 | 0.943 | 3.290 | 0.940 | 3.359 | 0.950 | 3.094 | 3.92 | 0 | 6.707 | 0.132 |
| | 1 | WAIPW | -0.126 | -0.123 | 0.082 | 0.037 | 1.015 | 0.003 | 0.945 | 3.230 | 0.942 | 3.312 | 0.952 | 3.018 | 3.92 | 0 | 6.656 | 0.131 |
| AIPW(0.5) | 1 | IPW | -0.126 | -0.127 | 0.124 | -0.016 | 1.009 | -0.002 | 0.945 | 3.213 | 0.951 | 3.053 | 0.948 | 3.140 | 3.92 | 0 | 15.485 | 0.308 |
| | 1 | WIPW | -0.126 | -0.127 | 0.163 | -0.009 | 1.013 | -0.001 | 0.947 | 3.157 | 0.947 | 3.157 | 0.947 | 3.163 | 3.92 | 0 | 26.457 | 0.533 |
| | 1 | AIPW | -0.126 | -0.127 | 0.098 | -0.015 | 1.002 | -0.001 | 0.952 | 3.017 | 0.951 | 3.041 | 0.947 | 3.174 | 3.92 | 0 | 9.596 | 0.190 |
| | 1 | WAIPW | -0.126 | -0.127 | 0.092 | -0.013 | 0.998 | -0.001 | 0.955 | 2.944 | 0.954 | 2.951 | 0.948 | 3.146 | 3.92 | 0 | 8.501 | 0.168 |
| AIPW(0.75) | 1 | IPW | -0.126 | -0.121 | 0.173 | 0.028 | 1.031 | 0.005 | 0.943 | 3.290 | 0.944 | 3.258 | 0.944 | 3.241 | 3.92 | 0 | 29.828 | 0.595 |
| | 1 | WIPW | -0.126 | -0.120 | 0.226 | 0.024 | 1.049 | 0.006 | 0.937 | 3.442 | 0.941 | 3.343 | 0.944 | 3.263 | 3.92 | 0 | 51.250 | 1.034 |
| | 1 | AIPW | -0.126 | -0.121 | 0.134 | 0.029 | 1.014 | 0.004 | 0.949 | 3.112 | 0.947 | 3.158 | 0.951 | 3.059 | 3.92 | 0 | 17.875 | 0.346 |
| | 1 | WAIPW | -0.126 | -0.122 | 0.111 | 0.034 | 1.009 | 0.004 | 0.950 | 3.089 | 0.945 | 3.219 | 0.950 | 3.089 | 3.92 | 0 | 12.346 | 0.240 |
| AIPW(1) | 1 | IPW | -0.126 | -0.125 | 0.249 | 0.004 | 1.097 | 0.001 | 0.923 | 3.762 | 0.938 | 3.411 | 0.937 | 3.436 | 3.92 | 0 | 62.016 | 1.258 |
| | 1 | WIPW | -0.126 | -0.125 | 0.319 | 0.006 | 1.142 | 0.001 | 0.915 | 3.949 | 0.930 | 3.613 | 0.930 | 3.609 | 3.92 | 0 | 101.886 | 2.081 |
| | 1 | AIPW | -0.126 | -0.125 | 0.191 | -0.008 | 1.009 | 0.001 | 0.950 | 3.077 | 0.949 | 3.100 | 0.949 | 3.106 | 3.92 | 0 | 36.549 | 0.757 |
| | 1 | WAIPW | -0.126 | -0.125 | 0.125 | 0.003 | 0.997 | 0.001 | 0.953 | 2.999 | 0.951 | 3.041 | 0.950 | 3.094 | 3.92 | 0 | 15.612 | 0.309 |
| AR-1 | 1 | IPW | -0.126 | -0.126 | 0.184 | 0.016 | 1.060 | 0.000 | 0.937 | 3.441 | 0.938 | 3.421 | 0.946 | 3.186 | 3.92 | 0 | 33.924 | 0.700 |

| Randomization Method | Regime | Estimator | True Value x 10^3 | Mean Estimate x 10^3 | SD Estimates x 10^3 | Mean Normalized | SD Normalized | Mean Bias | CI Coverage | SE CI Coverage x 10^3 | LB Coverage | SE LB Coverage x 10^3 | UB Coverage x 10^3 | SE UB Coverage x 10^3 | Mean CI Length | SE CI Length x 10^3 | MSE x 10^3 | SE MSE x 10^3 |
|---|---|---|---|---|---|---|---|---|---|---|---|---|---|---|---|---|---|---|
| | 1 | WIPW | -0.126 | -0.127 | 0.137 | 0.001 | 1.035 | -0.002 | 0.941 | 3.343 | 0.942 | 3.301 | 0.947 | 3.180 | 3.92 | 0 | 18.656 | 0.393 |
| | 1 | AIPW | -0.126 | -0.127 | 0.146 | 0.002 | 1.027 | -0.001 | 0.942 | 3.311 | 0.946 | 3.208 | 0.942 | 3.301 | 3.92 | 0 | 21.387 | 0.445 |
| | 1 | WAIPW | -0.126 | -0.129 | 0.132 | -0.020 | 1.020 | -0.003 | 0.946 | 3.197 | 0.949 | 3.100 | 0.944 | 3.257 | 3.92 | 0 | 17.425 | 0.357 |
| AR-2 | 1 | IPW | -0.126 | -0.123 | 0.192 | 0.029 | 1.049 | 0.003 | 0.935 | 3.492 | 0.939 | 3.395 | 0.946 | 3.197 | 3.92 | 0 | 36.677 | 0.738 |
| | 1 | WIPW | -0.126 | -0.124 | 0.138 | 0.026 | 1.010 | 0.002 | 0.948 | 3.146 | 0.946 | 3.208 | 0.953 | 2.993 | 3.92 | 0 | 19.020 | 0.382 |
| | 1 | AIPW | -0.126 | -0.124 | 0.150 | 0.010 | 1.015 | 0.001 | 0.947 | 3.169 | 0.949 | 3.106 | 0.947 | 3.163 | 3.92 | 0 | 22.577 | 0.441 |
| | 1 | WAIPW | -0.126 | -0.126 | 0.129 | 0.000 | 1.005 | 0.000 | 0.951 | 3.065 | 0.952 | 3.029 | 0.948 | 3.129 | 3.92 | 0 | 16.551 | 0.322 |
| IAIPW(0.25) | 1 | IPW | -0.126 | -0.128 | 0.102 | -0.021 | 1.021 | -0.002 | 0.942 | 3.296 | 0.946 | 3.191 | 0.947 | 3.169 | 3.92 | 0 | 10.427 | 0.211 |
| | 1 | WIPW | -0.126 | -0.127 | 0.101 | -0.017 | 1.019 | -0.002 | 0.943 | 3.285 | 0.947 | 3.180 | 0.948 | 3.146 | 3.92 | 0 | 10.146 | 0.204 |
| | 1 | AIPW | -0.126 | -0.126 | 0.082 | -0.006 | 1.015 | 0.000 | 0.944 | 3.252 | 0.947 | 3.175 | 0.943 | 3.269 | 3.92 | 0 | 6.646 | 0.135 |
| | 1 | WAIPW | -0.126 | -0.126 | 0.081 | -0.003 | 1.015 | 0.000 | 0.945 | 3.230 | 0.944 | 3.252 | 0.944 | 3.241 | 3.92 | 0 | 6.637 | 0.134 |
| IAIPW(0.5) | 1 | IPW | -0.126 | -0.126 | 0.124 | -0.003 | 1.005 | 0.000 | 0.947 | 3.169 | 0.953 | 2.987 | 0.944 | 3.263 | 3.92 | 0 | 15.310 | 0.309 |
| | 1 | WIPW | -0.126 | -0.125 | 0.114 | 0.005 | 0.997 | 0.001 | 0.951 | 3.053 | 0.954 | 2.969 | 0.947 | 3.163 | 3.92 | 0 | 12.935 | 0.261 |
| | 1 | AIPW | -0.126 | -0.124 | 0.098 | 0.011 | 1.008 | 0.001 | 0.948 | 3.135 | 0.949 | 3.100 | 0.951 | 3.059 | 3.92 | 0 | 9.658 | 0.197 |
| | 1 | WAIPW | -0.126 | -0.124 | 0.092 | 0.018 | 1.000 | 0.002 | 0.950 | 3.071 | 0.947 | 3.157 | 0.950 | 3.083 | 3.92 | 0 | 8.505 | 0.174 |
| IAIPW(0.75) | 1 | IPW | -0.126 | -0.120 | 0.174 | 0.034 | 1.037 | 0.005 | 0.939 | 3.375 | 0.943 | 3.290 | 0.945 | 3.225 | 3.92 | 0 | 30.147 | 0.602 |
| | 1 | WIPW | -0.126 | -0.122 | 0.138 | 0.030 | 1.016 | 0.004 | 0.945 | 3.219 | 0.945 | 3.225 | 0.951 | 3.042 | 3.92 | 0 | 19.182 | 0.381 |
| | 1 | AIPW | -0.126 | -0.123 | 0.135 | 0.013 | 1.021 | 0.003 | 0.943 | 3.279 | 0.946 | 3.203 | 0.947 | 3.163 | 3.92 | 0 | 18.163 | 0.351 |
| | 1 | WAIPW | -0.126 | -0.124 | 0.112 | 0.013 | 1.014 | 0.002 | 0.949 | 3.106 | 0.946 | 3.203 | 0.950 | 3.083 | 3.92 | 0 | 12.466 | 0.240 |
| IAIPW(1) | 1 | IPW | -0.126 | -0.124 | 0.253 | 0.007 | 1.109 | 0.002 | 0.923 | 3.771 | 0.928 | 3.651 | 0.936 | 3.472 | 3.92 | 0 | 63.940 | 1.307 |
| | 1 | WIPW | -0.126 | -0.124 | 0.158 | 0.016 | 1.029 | 0.002 | 0.944 | 3.252 | 0.942 | 3.311 | 0.948 | 3.152 | 3.92 | 0 | 24.858 | 0.499 |
| | 1 | AIPW | -0.126 | -0.124 | 0.196 | -0.002 | 1.033 | 0.002 | 0.944 | 3.257 | 0.943 | 3.274 | 0.945 | 3.219 | 3.92 | 0 | 38.606 | 0.791 |
| | 1 | WAIPW | -0.126 | -0.124 | 0.128 | 0.010 | 1.020 | 0.002 | 0.947 | 3.163 | 0.944 | 3.263 | 0.951 | 3.065 | 3.92 | 0 | 16.440 | 0.330 |
| IPW(0.25) | 1 | IPW | -0.126 | -0.125 | 0.101 | 0.008 | 1.009 | 0.001 | 0.948 | 3.135 | 0.947 | 3.158 | 0.951 | 3.059 | 3.92 | 0 | 10.229 | 0.210 |
| | 1 | WIPW | -0.126 | -0.126 | 0.126 | 0.000 | 1.016 | 0.000 | 0.946 | 3.203 | 0.950 | 3.089 | 0.947 | 3.163 | 3.92 | 0 | 15.883 | 0.325 |
| | 1 | AIPW | -0.126 | -0.124 | 0.083 | 0.018 | 1.022 | 0.002 | 0.943 | 3.279 | 0.942 | 3.306 | 0.949 | 3.118 | 3.92 | 0 | 6.812 | 0.135 |
| | 1 | WAIPW | -0.126 | -0.124 | 0.082 | 0.017 | 1.019 | 0.001 | 0.945 | 3.230 | 0.944 | 3.252 | 0.944 | 3.241 | 3.92 | 0 | 6.750 | 0.134 |
| IPW(0.5) | 1 | IPW | -0.126 | -0.125 | 0.128 | 0.010 | 1.031 | 0.001 | 0.945 | 3.235 | 0.947 | 3.174 | 0.946 | 3.208 | 3.92 | 0 | 16.279 | 0.327 |
| | 1 | WIPW | -0.126 | -0.124 | 0.167 | 0.008 | 1.042 | 0.001 | 0.940 | 3.348 | 0.943 | 3.274 | 0.942 | 3.295 | 3.92 | 0 | 28.038 | 0.554 |
| | 1 | AIPW | -0.126 | -0.125 | 0.101 | 0.006 | 1.028 | 0.001 | 0.946 | 3.186 | 0.946 | 3.191 | 0.949 | 3.106 | 3.92 | 0 | 10.108 | 0.204 |
| | 1 | WAIPW | -0.126 | -0.125 | 0.094 | 0.010 | 1.019 | 0.001 | 0.944 | 3.241 | 0.949 | 3.123 | 0.948 | 3.140 | 3.92 | 0 | 8.871 | 0.178 |
| IPW(0.75) | 1 | IPW | -0.126 | -0.127 | 0.176 | -0.002 | 1.049 | -0.001 | 0.940 | 3.370 | 0.942 | 3.301 | 0.945 | 3.214 | 3.92 | 0 | 31.046 | 0.621 |
| | 1 | WIPW | -0.126 | -0.125 | 0.230 | 0.006 | 1.062 | 0.001 | 0.938 | 3.416 | 0.938 | 3.406 | 0.942 | 3.317 | 3.92 | 0 | 52.755 | 1.067 |
| | 1 | AIPW | -0.126 | -0.126 | 0.137 | -0.011 | 1.033 | -0.001 | 0.944 | 3.241 | 0.946 | 3.186 | 0.940 | 3.349 | 3.92 | 0 | 18.721 | 0.377 |
| | 1 | WAIPW | -0.126 | -0.127 | 0.113 | -0.015 | 1.022 | -0.001 | 0.947 | 3.158 | 0.950 | 3.089 | 0.941 | 3.343 | 3.92 | 0 | 12.668 | 0.258 |
| IPW(1) | 1 | IPW | -0.126 | -0.124 | 0.255 | 0.011 | 1.129 | 0.002 | 0.917 | 3.902 | 0.928 | 3.647 | 0.931 | 3.580 | 3.92 | 0 | 65.182 | 1.321 |
| | 1 | WIPW | -0.126 | -0.123 | 0.326 | 0.014 | 1.176 | 0.003 | 0.908 | 4.084 | 0.923 | 3.771 | 0.927 | 3.689 | 3.92 | 0 | 106.564 | 2.191 |
| | 1 | AIPW | -0.126 | -0.124 | 0.196 | -0.002 | 1.031 | 0.002 | 0.949 | 3.117 | 0.944 | 3.252 | 0.945 | 3.219 | 3.92 | 0 | 38.353 | 0.786 |
| | 1 | WAIPW | -0.126 | -0.124 | 0.128 | 0.007 | 1.019 | 0.002 | 0.948 | 3.129 | 0.948 | 3.146 | 0.949 | 3.106 | 3.92 | 0 | 16.350 | 0.334 |
| SR | 1 | IPW | -0.126 | -0.125 | 0.089 | 0.004 | 1.021 | 0.000 | 0.944 | 3.241 | 0.949 | 3.123 | 0.949 | 3.123 | 3.92 | 0 | 7.970 | 0.164 |
| | 1 | WIPW | -0.126 | -0.126 | 0.106 | 0.001 | 1.014 | 0.000 | 0.946 | 3.186 | 0.948 | 3.129 | 0.948 | 3.152 | 3.92 | 0 | 11.142 | 0.231 |
| | 1 | AIPW | -0.126 | -0.125 | 0.073 | 0.006 | 1.017 | 0.000 | 0.946 | 3.197 | 0.948 | 3.146 | 0.944 | 3.246 | 3.92 | 0 | 5.297 | 0.111 |
| | 1 | WAIPW | -0.126 | -0.125 | 0.074 | 0.005 | 1.019 | 0.000 | 0.946 | 3.202 | 0.948 | 3.140 | 0.943 | 3.274 | 3.92 | 0 | 5.418 | 0.113 |
| TS(0.25) | 1 | IPW | -0.126 | -0.182 | 0.515 | -0.152 | 2.332 | -0.056 | 0.780 | 5.859 | 0.869 | 4.772 | 0.808 | 5.569 | 3.92 | 0 | 267.818 | 7.354 |

| Randomization Method | Regime | Estimator | True Value x 10^3 | Mean Estimate x 10^3 | SD Estimates x 10^3 | Mean Normalized | SD Normalized | Mean Bias | CI Coverage | SE CI Coverage x 10^3 | LB Coverage | SE LB Coverage x 10^3 | UB Coverage x 10^3 | SE UB Coverage x 10^3 | Mean CI Length | SE CI Length x 10^3 | MSE x 10^3 | SE MSE x 10^3 |
|---|---|---|---|---|---|---|---|---|---|---|---|---|---|---|---|---|---|---|
| | 1 | WIPW | -0.126 | -0.124 | 0.195 | -0.019 | 1.074 | 0.002 | 0.935 | 3.482 | 0.942 | 3.301 | 0.938 | 3.401 | 3.92 | 0 | 38.194 | 1.356 |
| | 1 | AIPW | -0.126 | -0.130 | 1.034 | 0.111 | 1.159 | -0.005 | 0.932 | 3.556 | 0.913 | 3.982 | 0.956 | 2.907 | 3.92 | 0 | 1068.952 | 191.109 |
| | 1 | WAIPW | -0.126 | -0.119 | 0.189 | 0.064 | 1.052 | 0.007 | 0.945 | 3.235 | 0.939 | 3.385 | 0.954 | 2.975 | 3.92 | 0 | 35.845 | 2.290 |
| TS(0.50) | 1 | IPW | -0.126 | -0.176 | 0.555 | -0.149 | 2.806 | -0.051 | 0.733 | 6.260 | 0.856 | 4.960 | 0.793 | 5.726 | 3.92 | 0 | 310.949 | 10.388 |
| | 1 | WIPW | -0.126 | -0.123 | 0.216 | -0.025 | 1.090 | 0.003 | 0.931 | 3.575 | 0.937 | 3.431 | 0.935 | 3.487 | 3.92 | 0 | 46.662 | 1.689 |
| | 1 | AIPW | -0.126 | -0.085 | 1.013 | 0.175 | 1.171 | 0.041 | 0.925 | 3.734 | 0.911 | 4.035 | 0.959 | 2.818 | 3.92 | 0 | 1027.068 | 160.975 |
| | 1 | WAIPW | -0.126 | -0.115 | 0.208 | 0.079 | 1.053 | 0.010 | 0.946 | 3.191 | 0.939 | 3.380 | 0.954 | 2.957 | 3.92 | 0 | 43.447 | 2.971 |
| TS(0.75) | 1 | IPW | -0.126 | -0.179 | 0.570 | -0.101 | 3.547 | -0.053 | 0.734 | 6.251 | 0.863 | 4.860 | 0.791 | 5.755 | 3.92 | 0 | 328.131 | 10.739 |
| | 1 | WIPW | -0.126 | -0.119 | 0.231 | -0.009 | 1.107 | 0.007 | 0.923 | 3.780 | 0.938 | 3.421 | 0.932 | 3.565 | 3.92 | 0 | 53.201 | 2.149 |
| | 1 | AIPW | -0.126 | -0.107 | 1.407 | 0.155 | 1.197 | 0.018 | 0.917 | 3.906 | 0.906 | 4.131 | 0.949 | 3.106 | 3.92 | 0 | 1979.166 | 254.632 |
| | 1 | WAIPW | -0.126 | -0.117 | 0.239 | 0.079 | 1.051 | 0.009 | 0.940 | 3.364 | 0.938 | 3.401 | 0.955 | 2.944 | 3.92 | 0 | 56.967 | 4.566 |
| TS(1) | 1 | IPW | -0.126 | -0.183 | 0.555 | -0.128 | 4.247 | -0.057 | 0.739 | 6.208 | 0.863 | 4.857 | 0.796 | 5.695 | 3.92 | 0 | 311.494 | 10.589 |
| | 1 | WIPW | -0.126 | -0.123 | 0.234 | -0.023 | 1.139 | 0.003 | 0.915 | 3.936 | 0.923 | 3.766 | 0.928 | 3.665 | 3.92 | 0 | 54.614 | 2.022 |
| | 1 | AIPW | -0.126 | -0.121 | 1.487 | 0.173 | 1.226 | 0.004 | 0.909 | 4.060 | 0.900 | 4.236 | 0.950 | 3.071 | 3.92 | 0 | 2209.388 | 272.675 |
| | 1 | WAIPW | -0.126 | -0.121 | 0.249 | 0.047 | 1.072 | 0.004 | 0.937 | 3.426 | 0.935 | 3.482 | 0.949 | 3.100 | 3.92 | 0 | 61.941 | 5.257 |
| WAIPW(0.25) | 1 | IPW | -0.126 | -0.125 | 0.103 | 0.011 | 1.026 | 0.001 | 0.940 | 3.359 | 0.947 | 3.158 | 0.944 | 3.252 | 3.92 | 0 | 10.521 | 0.210 |
| | 1 | WIPW | -0.126 | -0.125 | 0.101 | 0.014 | 1.023 | 0.001 | 0.942 | 3.317 | 0.947 | 3.169 | 0.946 | 3.203 | 3.92 | 0 | 10.208 | 0.205 |
| | 1 | AIPW | -0.126 | -0.124 | 0.083 | 0.022 | 1.024 | 0.002 | 0.942 | 3.301 | 0.945 | 3.225 | 0.948 | 3.152 | 3.92 | 0 | 6.814 | 0.136 |
| | 1 | WAIPW | -0.126 | -0.124 | 0.082 | 0.019 | 1.019 | 0.002 | 0.945 | 3.219 | 0.945 | 3.230 | 0.947 | 3.169 | 3.92 | 0 | 6.726 | 0.136 |
| WAIPW(0.5) | 1 | IPW | -0.126 | -0.125 | 0.127 | 0.010 | 1.028 | 0.001 | 0.937 | 3.436 | 0.945 | 3.213 | 0.943 | 3.274 | 3.92 | 0 | 16.089 | 0.317 |
| | 1 | WIPW | -0.126 | -0.124 | 0.117 | 0.015 | 1.026 | 0.002 | 0.942 | 3.306 | 0.944 | 3.241 | 0.942 | 3.295 | 3.92 | 0 | 13.757 | 0.276 |
| | 1 | AIPW | -0.126 | -0.125 | 0.101 | 0.006 | 1.027 | 0.001 | 0.946 | 3.191 | 0.946 | 3.208 | 0.944 | 3.241 | 3.92 | 0 | 10.123 | 0.201 |
| | 1 | WAIPW | -0.126 | -0.125 | 0.095 | 0.010 | 1.025 | 0.001 | 0.946 | 3.191 | 0.947 | 3.180 | 0.945 | 3.230 | 3.92 | 0 | 8.989 | 0.180 |
| WAIPW(0.75) | 1 | IPW | -0.126 | -0.131 | 0.175 | -0.032 | 1.049 | -0.006 | 0.939 | 3.390 | 0.948 | 3.141 | 0.937 | 3.432 | 3.92 | 0 | 30.624 | 0.602 |
| | 1 | WIPW | -0.126 | -0.131 | 0.141 | -0.037 | 1.035 | -0.005 | 0.944 | 3.252 | 0.949 | 3.106 | 0.940 | 3.359 | 3.92 | 0 | 19.812 | 0.390 |
| | 1 | AIPW | -0.126 | -0.128 | 0.136 | -0.027 | 1.036 | -0.003 | 0.943 | 3.279 | 0.949 | 3.106 | 0.940 | 3.364 | 3.92 | 0 | 18.536 | 0.374 |
| | 1 | WAIPW | -0.126 | -0.128 | 0.112 | -0.024 | 1.023 | -0.002 | 0.951 | 3.042 | 0.953 | 3.006 | 0.942 | 3.317 | 3.92 | 0 | 12.581 | 0.252 |
| WAIPW(1) | 1 | IPW | -0.126 | -0.127 | 0.253 | -0.008 | 1.110 | -0.002 | 0.920 | 3.833 | 0.935 | 3.482 | 0.926 | 3.707 | 3.92 | 0 | 64.195 | 1.303 |
| | 1 | WIPW | -0.126 | -0.127 | 0.158 | -0.010 | 1.031 | -0.001 | 0.943 | 3.274 | 0.945 | 3.219 | 0.937 | 3.426 | 3.92 | 0 | 25.027 | 0.502 |
| | 1 | AIPW | -0.126 | -0.126 | 0.197 | -0.011 | 1.044 | 0.000 | 0.944 | 3.263 | 0.946 | 3.186 | 0.942 | 3.317 | 3.92 | 0 | 38.904 | 0.794 |
| | 1 | WAIPW | -0.126 | -0.126 | 0.129 | -0.008 | 1.026 | 0.000 | 0.944 | 3.246 | 0.946 | 3.186 | 0.943 | 3.268 | 3.92 | 0 | 16.551 | 0.333 |
| WIPW(0.25) | 1 | IPW | -0.126 | -0.126 | 0.101 | -0.001 | 1.007 | 0.000 | 0.950 | 3.089 | 0.955 | 2.938 | 0.948 | 3.146 | 3.92 | 0 | 10.224 | 0.205 |
| | 1 | WIPW | -0.126 | -0.126 | 0.100 | 0.001 | 1.008 | 0.000 | 0.952 | 3.036 | 0.954 | 2.969 | 0.948 | 3.152 | 3.92 | 0 | 10.009 | 0.200 |
| | 1 | AIPW | -0.126 | -0.126 | 0.080 | -0.004 | 0.995 | 0.000 | 0.953 | 3.000 | 0.950 | 3.083 | 0.953 | 3.000 | 3.92 | 0 | 6.474 | 0.131 |
| | 1 | WAIPW | -0.126 | -0.126 | 0.080 | -0.004 | 0.997 | 0.000 | 0.952 | 3.036 | 0.951 | 3.048 | 0.951 | 3.059 | 3.92 | 0 | 6.473 | 0.131 |
| WIPW(0.5) | 1 | IPW | -0.126 | -0.126 | 0.124 | -0.004 | 1.004 | 0.000 | 0.955 | 2.938 | 0.952 | 3.029 | 0.950 | 3.094 | 3.92 | 0 | 15.429 | 0.305 |
| | 1 | WIPW | -0.126 | -0.126 | 0.115 | -0.003 | 1.000 | 0.000 | 0.955 | 2.938 | 0.952 | 3.029 | 0.949 | 3.100 | 3.92 | 0 | 13.109 | 0.262 |
| | 1 | AIPW | -0.126 | -0.126 | 0.099 | -0.006 | 1.005 | 0.000 | 0.952 | 3.017 | 0.954 | 2.969 | 0.945 | 3.235 | 3.92 | 0 | 9.757 | 0.195 |
| | 1 | WAIPW | -0.126 | -0.126 | 0.092 | -0.005 | 0.994 | 0.000 | 0.954 | 2.963 | 0.957 | 2.882 | 0.949 | 3.100 | 3.92 | 0 | 8.496 | 0.169 |
| WIPW(0.75) | 1 | IPW | -0.126 | -0.122 | 0.175 | 0.024 | 1.042 | 0.004 | 0.938 | 3.421 | 0.939 | 3.375 | 0.946 | 3.186 | 3.92 | 0 | 30.597 | 0.611 |
| | 1 | WIPW | -0.126 | -0.123 | 0.140 | 0.022 | 1.027 | 0.003 | 0.944 | 3.252 | 0.939 | 3.396 | 0.948 | 3.129 | 3.92 | 0 | 19.605 | 0.386 |
| | 1 | AIPW | -0.126 | -0.123 | 0.135 | 0.017 | 1.016 | 0.003 | 0.947 | 3.180 | 0.943 | 3.274 | 0.950 | 3.083 | 3.92 | 0 | 18.164 | 0.360 |
| | 1 | WAIPW | -0.126 | -0.124 | 0.112 | 0.018 | 1.015 | 0.002 | 0.947 | 3.163 | 0.946 | 3.203 | 0.951 | 3.059 | 3.92 | 0 | 12.542 | 0.244 |
| WIPW(1) | 1 | IPW | -0.126 | -0.125 | 0.255 | 0.000 | 1.113 | 0.001 | 0.922 | 3.784 | 0.932 | 3.551 | 0.930 | 3.609 | 3.92 | 0 | 64.929 | 1.281 |

| Randomization Method | Regime | Estimator | True Value x 10^3 | Mean Estimate x 10^3 | SD Estimates x 10^3 | Mean Normalized | SD Normalized | Mean Bias | CI Coverage | SE CI Coverage x 10^3 | LB Coverage | SE LB Coverage x 10^3 | UB Coverage x 10^3 | SE UB Coverage x 10^3 | Mean CI Length | SE CI Length x 10^3 | MSE x 10^3 | SE MSE x 10^3 |
|---|---|---|---|---|---|---|---|---|---|---|---|---|---|---|---|---|---|---|
| | 1 | WIPW | -0.126 | -0.125 | 0.159 | 0.001 | 1.036 | 0.000 | 0.941 | 3.338 | 0.947 | 3.157 | 0.940 | 3.359 | 3.92 | 0 | 25.278 | 0.514 |
| | 1 | AIPW | -0.126 | -0.126 | 0.195 | -0.014 | 1.031 | 0.000 | 0.940 | 3.348 | 0.949 | 3.123 | 0.940 | 3.348 | 3.92 | 0 | 38.028 | 0.764 |
| | 1 | WAIPW | -0.126 | -0.127 | 0.129 | -0.015 | 1.029 | -0.001 | 0.943 | 3.284 | 0.946 | 3.197 | 0.941 | 3.338 | 3.92 | 0 | 16.626 | 0.332 |
| AIPW(0.25) | 2 | IPW | -0.374 | -0.372 | 0.095 | 0.021 | 0.995 | 0.002 | 0.953 | 3.000 | 0.950 | 3.083 | 0.956 | 2.888 | 3.92 | 0 | 9.002 | 0.180 |
| | 2 | WIPW | -0.374 | -0.372 | 0.119 | 0.013 | 1.009 | 0.002 | 0.948 | 3.129 | 0.949 | 3.118 | 0.949 | 3.123 | 3.92 | 0 | 14.115 | 0.280 |
| | 2 | AIPW | -0.374 | -0.372 | 0.078 | 0.029 | 1.003 | 0.002 | 0.949 | 3.106 | 0.943 | 3.269 | 0.954 | 2.975 | 3.92 | 0 | 6.122 | 0.121 |
| | 2 | WAIPW | -0.374 | -0.372 | 0.078 | 0.028 | 1.000 | 0.002 | 0.948 | 3.129 | 0.944 | 3.263 | 0.954 | 2.975 | 3.92 | 0 | 6.094 | 0.121 |
| AIPW(0.5) | 2 | IPW | -0.374 | -0.375 | 0.119 | -0.009 | 1.015 | -0.001 | 0.946 | 3.202 | 0.951 | 3.059 | 0.946 | 3.197 | 3.92 | 0 | 14.185 | 0.285 |
| | 2 | WIPW | -0.374 | -0.374 | 0.157 | -0.001 | 1.025 | 0.001 | 0.943 | 3.290 | 0.944 | 3.241 | 0.949 | 3.100 | 3.92 | 0 | 24.579 | 0.489 |
| | 2 | AIPW | -0.374 | -0.375 | 0.097 | -0.015 | 1.020 | -0.001 | 0.946 | 3.186 | 0.948 | 3.140 | 0.945 | 3.224 | 3.92 | 0 | 9.378 | 0.186 |
| | 2 | WAIPW | -0.374 | -0.376 | 0.091 | -0.023 | 1.012 | -0.002 | 0.947 | 3.157 | 0.950 | 3.094 | 0.946 | 3.191 | 3.92 | 0 | 8.288 | 0.164 |
| AIPW(0.75) | 2 | IPW | -0.374 | -0.373 | 0.166 | 0.005 | 1.038 | 0.001 | 0.939 | 3.375 | 0.944 | 3.263 | 0.944 | 3.263 | 3.92 | 0 | 27.490 | 0.547 |
| | 2 | WIPW | -0.374 | -0.373 | 0.216 | 0.003 | 1.051 | 0.001 | 0.938 | 3.416 | 0.941 | 3.338 | 0.944 | 3.252 | 3.92 | 0 | 46.750 | 0.941 |
| | 2 | AIPW | -0.374 | -0.373 | 0.131 | 0.000 | 1.017 | 0.001 | 0.948 | 3.152 | 0.951 | 3.053 | 0.945 | 3.236 | 3.92 | 0 | 17.150 | 0.348 |
| | 2 | WAIPW | -0.374 | -0.374 | 0.109 | -0.002 | 1.014 | 0.000 | 0.948 | 3.152 | 0.950 | 3.077 | 0.949 | 3.112 | 3.92 | 0 | 11.808 | 0.240 |
| AIPW(1) | 2 | IPW | -0.374 | -0.375 | 0.237 | -0.006 | 1.094 | -0.001 | 0.927 | 3.670 | 0.937 | 3.436 | 0.931 | 3.580 | 3.92 | 0 | 56.260 | 1.132 |
| | 2 | WIPW | -0.374 | -0.375 | 0.303 | -0.003 | 1.135 | -0.001 | 0.916 | 3.919 | 0.930 | 3.604 | 0.930 | 3.604 | 3.92 | 0 | 92.092 | 1.876 |
| | 2 | AIPW | -0.374 | -0.376 | 0.188 | -0.027 | 1.033 | -0.002 | 0.946 | 3.208 | 0.946 | 3.186 | 0.943 | 3.284 | 3.92 | 0 | 35.277 | 0.721 |
| | 2 | WAIPW | -0.374 | -0.377 | 0.124 | -0.027 | 1.021 | -0.002 | 0.942 | 3.301 | 0.949 | 3.117 | 0.946 | 3.208 | 3.92 | 0 | 15.296 | 0.305 |
| AR-1 | 2 | IPW | -0.374 | -0.392 | 0.342 | -0.186 | 1.288 | -0.018 | 0.882 | 4.563 | 0.934 | 3.507 | 0.871 | 4.741 | 3.92 | 0 | 117.470 | 3.072 |
| | 2 | WIPW | -0.374 | -0.383 | 0.194 | -0.095 | 1.098 | -0.008 | 0.930 | 3.599 | 0.952 | 3.017 | 0.920 | 3.841 | 3.92 | 0 | 37.574 | 0.880 |
| | 2 | AIPW | -0.374 | -0.380 | 0.295 | -0.081 | 1.095 | -0.006 | 0.939 | 3.375 | 0.951 | 3.059 | 0.932 | 3.561 | 3.92 | 0 | 87.150 | 2.721 |
| | 2 | WAIPW | -0.374 | -0.383 | 0.172 | -0.076 | 1.062 | -0.009 | 0.941 | 3.333 | 0.952 | 3.029 | 0.936 | 3.457 | 3.92 | 0 | 29.700 | 0.742 |
| AR-2 | 2 | IPW | -0.374 | -0.377 | 0.258 | -0.055 | 1.137 | -0.003 | 0.918 | 3.880 | 0.943 | 3.284 | 0.916 | 3.919 | 3.92 | 0 | 66.575 | 1.457 |
| | 2 | WIPW | -0.374 | -0.375 | 0.162 | -0.015 | 1.031 | -0.001 | 0.943 | 3.290 | 0.951 | 3.059 | 0.938 | 3.416 | 3.92 | 0 | 26.165 | 0.556 |
| | 2 | AIPW | -0.374 | -0.372 | 0.201 | -0.005 | 1.030 | 0.002 | 0.949 | 3.100 | 0.953 | 3.005 | 0.945 | 3.235 | 3.92 | 0 | 40.405 | 0.991 |
| | 2 | WAIPW | -0.374 | -0.376 | 0.140 | -0.015 | 1.002 | -0.002 | 0.957 | 2.882 | 0.953 | 2.993 | 0.947 | 3.163 | 3.92 | 0 | 19.597 | 0.427 |
| IAIPW(0.25) | 2 | IPW | -0.374 | -0.376 | 0.097 | -0.019 | 1.013 | -0.002 | 0.943 | 3.269 | 0.952 | 3.018 | 0.943 | 3.269 | 3.92 | 0 | 9.324 | 0.187 |
| | 2 | WIPW | -0.374 | -0.376 | 0.096 | -0.019 | 1.015 | -0.002 | 0.946 | 3.208 | 0.951 | 3.059 | 0.942 | 3.296 | 3.92 | 0 | 9.122 | 0.183 |
| | 2 | AIPW | -0.374 | -0.375 | 0.080 | -0.015 | 1.018 | -0.001 | 0.951 | 3.059 | 0.951 | 3.059 | 0.945 | 3.225 | 3.92 | 0 | 6.340 | 0.124 |
| | 2 | WAIPW | -0.374 | -0.375 | 0.080 | -0.015 | 1.017 | -0.001 | 0.949 | 3.106 | 0.947 | 3.158 | 0.950 | 3.083 | 3.92 | 0 | 6.325 | 0.124 |
| IAIPW(0.5) | 2 | IPW | -0.374 | -0.378 | 0.120 | -0.028 | 1.020 | -0.003 | 0.949 | 3.112 | 0.950 | 3.071 | 0.947 | 3.180 | 3.92 | 0 | 14.416 | 0.289 |
| | 2 | WIPW | -0.374 | -0.377 | 0.110 | -0.027 | 1.009 | -0.003 | 0.951 | 3.041 | 0.951 | 3.047 | 0.946 | 3.208 | 3.92 | 0 | 12.114 | 0.242 |
| | 2 | AIPW | -0.374 | -0.377 | 0.097 | -0.027 | 1.025 | -0.002 | 0.951 | 3.059 | 0.948 | 3.146 | 0.949 | 3.123 | 3.92 | 0 | 9.434 | 0.185 |
| | 2 | WAIPW | -0.374 | -0.376 | 0.091 | -0.022 | 1.015 | -0.002 | 0.953 | 2.987 | 0.949 | 3.100 | 0.947 | 3.163 | 3.92 | 0 | 8.309 | 0.164 |
| IAIPW(0.75) | 2 | IPW | -0.374 | -0.375 | 0.165 | -0.001 | 1.036 | 0.000 | 0.940 | 3.359 | 0.943 | 3.274 | 0.945 | 3.214 | 3.92 | 0 | 27.183 | 0.561 |
| | 2 | WIPW | -0.374 | -0.374 | 0.132 | -0.001 | 1.016 | 0.000 | 0.945 | 3.225 | 0.951 | 3.065 | 0.949 | 3.112 | 3.92 | 0 | 17.384 | 0.359 |
| | 2 | AIPW | -0.374 | -0.375 | 0.131 | -0.015 | 1.031 | -0.001 | 0.941 | 3.343 | 0.947 | 3.169 | 0.942 | 3.317 | 3.92 | 0 | 17.285 | 0.357 |
| | 2 | WAIPW | -0.374 | -0.374 | 0.109 | -0.006 | 1.017 | 0.000 | 0.945 | 3.225 | 0.947 | 3.163 | 0.947 | 3.158 | 3.92 | 0 | 11.825 | 0.246 |
| IAIPW(1) | 2 | IPW | -0.374 | -0.375 | 0.240 | -0.007 | 1.103 | -0.001 | 0.926 | 3.698 | 0.933 | 3.546 | 0.934 | 3.517 | 3.92 | 0 | 57.596 | 1.157 |
| | 2 | WIPW | -0.374 | -0.376 | 0.151 | -0.015 | 1.035 | -0.002 | 0.944 | 3.257 | 0.947 | 3.174 | 0.943 | 3.284 | 3.92 | 0 | 22.758 | 0.463 |
| | 2 | AIPW | -0.374 | -0.373 | 0.190 | -0.011 | 1.029 | 0.001 | 0.947 | 3.163 | 0.946 | 3.191 | 0.944 | 3.252 | 3.92 | 0 | 35.956 | 0.748 |
| | 2 | WAIPW | -0.374 | -0.374 | 0.125 | -0.009 | 1.030 | 0.000 | 0.942 | 3.311 | 0.941 | 3.322 | 0.946 | 3.191 | 3.92 | 0 | 15.623 | 0.308 |
| IPW(0.25) | 2 | IPW | -0.374 | -0.376 | 0.098 | -0.016 | 1.024 | -0.002 | 0.946 | 3.203 | 0.948 | 3.135 | 0.944 | 3.263 | 3.92 | 0 | 9.600 | 0.195 |

| Randomization Method | Regime | Estimator | True Value x 10^3 | Mean Estimate x 10^3 | SD Estimates x 10^3 | Mean Normalized | SD Normalized | Mean Bias | CI Coverage | SE CI Coverage x 10^3 | LB Coverage | SE LB Coverage x 10^3 | UB Coverage x 10^3 | SE UB Coverage x 10^3 | Mean CI Length | SE CI Length x 10^3 | MSE x 10^3 | SE MSE x 10^3 |
|---|---|---|---|---|---|---|---|---|---|---|---|---|---|---|---|---|---|---|
| | 2 | WIPW | -0.374 | -0.377 | 0.122 | -0.021 | 1.028 | -0.003 | 0.944 | 3.241 | 0.950 | 3.089 | 0.940 | 3.370 | 3.92 | 0 | 14.778 | 0.300 |
| | 2 | AIPW | -0.374 | -0.376 | 0.081 | -0.022 | 1.028 | -0.002 | 0.947 | 3.158 | 0.945 | 3.225 | 0.943 | 3.269 | 3.92 | 0 | 6.494 | 0.132 |
| | 2 | WAIPW | -0.374 | -0.376 | 0.081 | -0.023 | 1.026 | -0.002 | 0.946 | 3.197 | 0.946 | 3.203 | 0.945 | 3.230 | 3.92 | 0 | 6.485 | 0.133 |
| IPW(0.5) | 2 | IPW | -0.374 | -0.375 | 0.122 | -0.009 | 1.036 | -0.001 | 0.941 | 3.327 | 0.945 | 3.230 | 0.944 | 3.246 | 3.92 | 0 | 14.915 | 0.295 |
| | 2 | WIPW | -0.374 | -0.376 | 0.159 | -0.016 | 1.039 | -0.002 | 0.940 | 3.348 | 0.944 | 3.252 | 0.939 | 3.385 | 3.92 | 0 | 25.377 | 0.498 |
| | 2 | AIPW | -0.374 | -0.374 | 0.100 | -0.006 | 1.053 | 0.000 | 0.937 | 3.436 | 0.943 | 3.268 | 0.945 | 3.230 | 3.92 | 0 | 9.992 | 0.198 |
| | 2 | WAIPW | -0.374 | -0.374 | 0.094 | -0.004 | 1.041 | 0.000 | 0.940 | 3.369 | 0.943 | 3.290 | 0.945 | 3.224 | 3.92 | 0 | 8.759 | 0.174 |
| IPW(0.75) | 2 | IPW | -0.374 | -0.374 | 0.162 | 0.001 | 1.020 | 0.000 | 0.944 | 3.258 | 0.948 | 3.152 | 0.945 | 3.214 | 3.92 | 0 | 26.383 | 0.519 |
| | 2 | WIPW | -0.374 | -0.374 | 0.212 | 0.003 | 1.033 | 0.000 | 0.940 | 3.364 | 0.949 | 3.123 | 0.949 | 3.118 | 3.92 | 0 | 44.960 | 0.899 |
| | 2 | AIPW | -0.374 | -0.375 | 0.131 | -0.009 | 1.017 | -0.001 | 0.944 | 3.258 | 0.948 | 3.135 | 0.948 | 3.135 | 3.92 | 0 | 17.194 | 0.350 |
| | 2 | WAIPW | -0.374 | -0.375 | 0.109 | -0.008 | 1.017 | 0.000 | 0.943 | 3.285 | 0.948 | 3.129 | 0.946 | 3.203 | 3.92 | 0 | 11.918 | 0.242 |
| IPW(1) | 2 | IPW | -0.374 | -0.374 | 0.239 | 0.002 | 1.099 | 0.000 | 0.924 | 3.743 | 0.928 | 3.647 | 0.931 | 3.580 | 3.92 | 0 | 57.248 | 1.159 |
| | 2 | WIPW | -0.374 | -0.374 | 0.306 | 0.006 | 1.139 | 0.001 | 0.913 | 3.986 | 0.923 | 3.775 | 0.928 | 3.661 | 3.92 | 0 | 93.569 | 1.927 |
| | 2 | AIPW | -0.374 | -0.375 | 0.187 | -0.014 | 1.032 | -0.001 | 0.946 | 3.186 | 0.944 | 3.252 | 0.945 | 3.224 | 3.92 | 0 | 35.086 | 0.715 |
| | 2 | WAIPW | -0.374 | -0.375 | 0.124 | -0.010 | 1.028 | -0.001 | 0.941 | 3.333 | 0.947 | 3.180 | 0.947 | 3.157 | 3.92 | 0 | 15.444 | 0.313 |
| SR | 2 | IPW | -0.374 | -0.375 | 0.084 | -0.011 | 1.009 | -0.001 | 0.948 | 3.135 | 0.952 | 3.029 | 0.947 | 3.174 | 3.92 | 0 | 7.063 | 0.140 |
| | 2 | WIPW | -0.374 | -0.374 | 0.100 | -0.003 | 1.013 | 0.000 | 0.949 | 3.112 | 0.950 | 3.094 | 0.944 | 3.263 | 3.92 | 0 | 10.076 | 0.197 |
| | 2 | AIPW | -0.374 | -0.375 | 0.070 | -0.008 | 1.013 | -0.001 | 0.948 | 3.135 | 0.949 | 3.100 | 0.944 | 3.252 | 3.92 | 0 | 4.954 | 0.099 |
| | 2 | WAIPW | -0.374 | -0.375 | 0.071 | -0.007 | 1.013 | -0.001 | 0.948 | 3.135 | 0.951 | 3.059 | 0.942 | 3.295 | 3.92 | 0 | 5.071 | 0.100 |
| TS(0.25) | 2 | IPW | -0.374 | -0.362 | 0.404 | 0.095 | 1.625 | 0.012 | 0.844 | 5.127 | 0.869 | 4.775 | 0.891 | 4.401 | 3.92 | 0 | 163.108 | 3.911 |
| | 2 | WIPW | -0.374 | -0.369 | 0.167 | 0.041 | 1.067 | 0.005 | 0.939 | 3.390 | 0.927 | 3.675 | 0.947 | 3.169 | 3.92 | 0 | 27.915 | 0.640 |
| | 2 | AIPW | -0.374 | -0.375 | 0.489 | 0.040 | 1.125 | -0.001 | 0.941 | 3.338 | 0.933 | 3.526 | 0.945 | 3.219 | 3.92 | 0 | 238.995 | 67.219 |
| | 2 | WAIPW | -0.374 | -0.368 | 0.174 | 0.049 | 1.066 | 0.006 | 0.941 | 3.343 | 0.936 | 3.462 | 0.951 | 3.041 | 3.92 | 0 | 30.179 | 1.228 |
| TS(0.50) | 2 | IPW | -0.374 | -0.346 | 0.422 | 0.168 | 1.641 | 0.028 | 0.823 | 5.401 | 0.849 | 5.064 | 0.898 | 4.273 | 3.92 | 0 | 179.201 | 5.148 |
| | 2 | WIPW | -0.374 | -0.366 | 0.183 | 0.068 | 1.097 | 0.008 | 0.929 | 3.642 | 0.921 | 3.819 | 0.947 | 3.174 | 3.92 | 0 | 33.635 | 0.993 |
| | 2 | AIPW | -0.374 | -0.363 | 0.513 | 0.082 | 1.151 | 0.011 | 0.930 | 3.604 | 0.928 | 3.647 | 0.951 | 3.047 | 3.92 | 0 | 263.420 | 42.233 |
| | 2 | WAIPW | -0.374 | -0.364 | 0.182 | 0.091 | 1.080 | 0.010 | 0.937 | 3.436 | 0.927 | 3.679 | 0.951 | 3.047 | 3.92 | 0 | 33.366 | 1.309 |
| TS(0.75) | 2 | IPW | -0.374 | -0.345 | 0.426 | 0.152 | 1.779 | 0.029 | 0.824 | 5.391 | 0.852 | 5.025 | 0.897 | 4.299 | 3.92 | 0 | 182.411 | 5.376 |
| | 2 | WIPW | -0.374 | -0.365 | 0.189 | 0.089 | 1.092 | 0.009 | 0.931 | 3.580 | 0.926 | 3.693 | 0.948 | 3.146 | 3.92 | 0 | 35.720 | 1.009 |
| | 2 | AIPW | -0.374 | -0.379 | 0.711 | 0.059 | 1.151 | -0.005 | 0.929 | 3.642 | 0.924 | 3.757 | 0.946 | 3.191 | 3.92 | 0 | 505.265 | 78.950 |
| | 2 | WAIPW | -0.374 | -0.366 | 0.199 | 0.089 | 1.074 | 0.008 | 0.941 | 3.322 | 0.933 | 3.536 | 0.952 | 3.023 | 3.92 | 0 | 39.487 | 2.171 |
| TS(1) | 2 | IPW | -0.374 | -0.330 | 0.430 | 0.193 | 1.968 | 0.044 | 0.822 | 5.415 | 0.839 | 5.201 | 0.898 | 4.277 | 3.92 | 0 | 187.175 | 5.905 |
| | 2 | WIPW | -0.374 | -0.364 | 0.191 | 0.090 | 1.094 | 0.010 | 0.926 | 3.698 | 0.925 | 3.734 | 0.944 | 3.263 | 3.92 | 0 | 36.593 | 1.161 |
| | 2 | AIPW | -0.374 | -0.377 | 0.830 | 0.074 | 1.167 | -0.003 | 0.921 | 3.806 | 0.923 | 3.771 | 0.944 | 3.241 | 3.92 | 0 | 688.478 | 132.587 |
| | 2 | WAIPW | -0.374 | -0.368 | 0.205 | 0.080 | 1.068 | 0.006 | 0.938 | 3.406 | 0.934 | 3.517 | 0.950 | 3.094 | 3.92 | 0 | 42.073 | 2.774 |
| WAIPW(0.25) | 2 | IPW | -0.374 | -0.375 | 0.097 | -0.010 | 1.015 | -0.001 | 0.945 | 3.230 | 0.945 | 3.225 | 0.946 | 3.203 | 3.92 | 0 | 9.367 | 0.190 |
| | 2 | WIPW | -0.374 | -0.375 | 0.095 | -0.009 | 1.013 | -0.001 | 0.948 | 3.146 | 0.946 | 3.203 | 0.945 | 3.214 | 3.92 | 0 | 9.103 | 0.182 |
| | 2 | AIPW | -0.374 | -0.374 | 0.079 | 0.001 | 1.015 | 0.000 | 0.946 | 3.191 | 0.947 | 3.169 | 0.948 | 3.141 | 3.92 | 0 | 6.286 | 0.128 |
| | 2 | WAIPW | -0.374 | -0.374 | 0.079 | -0.001 | 1.011 | 0.000 | 0.946 | 3.197 | 0.949 | 3.112 | 0.950 | 3.077 | 3.92 | 0 | 6.249 | 0.126 |
| WAIPW(0.5) | 2 | IPW | -0.374 | -0.377 | 0.120 | -0.022 | 1.024 | -0.002 | 0.943 | 3.284 | 0.947 | 3.169 | 0.945 | 3.213 | 3.92 | 0 | 14.482 | 0.296 |
| | 2 | WIPW | -0.374 | -0.376 | 0.111 | -0.017 | 1.018 | -0.002 | 0.947 | 3.174 | 0.948 | 3.135 | 0.946 | 3.197 | 3.92 | 0 | 12.317 | 0.248 |
| | 2 | AIPW | -0.374 | -0.377 | 0.097 | -0.035 | 1.030 | -0.003 | 0.944 | 3.263 | 0.950 | 3.088 | 0.945 | 3.213 | 3.92 | 0 | 9.496 | 0.190 |
| | 2 | WAIPW | -0.374 | -0.377 | 0.092 | -0.030 | 1.025 | -0.003 | 0.943 | 3.284 | 0.949 | 3.100 | 0.945 | 3.230 | 3.92 | 0 | 8.460 | 0.168 |
| WAIPW(0.75) | 2 | IPW | -0.374 | -0.381 | 0.167 | -0.043 | 1.048 | -0.007 | 0.935 | 3.492 | 0.946 | 3.197 | 0.936 | 3.452 | 3.92 | 0 | 27.806 | 0.565 |

| Randomization Method | Regime | Estimator | True Value x 10^3 | Mean Estimate x 10^3 | SD Estimates x 10^3 | Mean Normalized | SD Normalized | Mean Bias | CI Coverage | SE CI Coverage x 10^3 | LB Coverage | SE LB Coverage x 10^3 | UB Coverage x 10^3 | SE UB Coverage x 10^3 | Mean CI Length | SE CI Length x 10^3 | MSE x 10^3 | SE MSE x 10^3 |
|---|---|---|---|---|---|---|---|---|---|---|---|---|---|---|---|---|---|---|
| | 2 | WIPW | -0.374 | -0.380 | 0.133 | -0.044 | 1.024 | -0.005 | 0.941 | 3.343 | 0.951 | 3.059 | 0.937 | 3.442 | 3.92 | 0 | 17.615 | 0.359 |
| | 2 | AIPW | -0.374 | -0.379 | 0.132 | -0.043 | 1.035 | -0.005 | 0.943 | 3.269 | 0.953 | 3.000 | 0.940 | 3.359 | 3.92 | 0 | 17.526 | 0.353 |
| | 2 | WAIPW | -0.374 | -0.378 | 0.109 | -0.042 | 1.024 | -0.004 | 0.945 | 3.219 | 0.953 | 3.006 | 0.941 | 3.333 | 3.92 | 0 | 11.989 | 0.242 |
| WAIPW(1) | 2 | IPW | -0.374 | -0.376 | 0.240 | -0.012 | 1.097 | -0.002 | 0.925 | 3.730 | 0.935 | 3.492 | 0.937 | 3.441 | 3.92 | 0 | 57.646 | 1.188 |
| | 2 | WIPW | -0.374 | -0.377 | 0.150 | -0.019 | 1.018 | -0.003 | 0.945 | 3.224 | 0.950 | 3.094 | 0.943 | 3.274 | 3.92 | 0 | 22.402 | 0.458 |
| | 2 | AIPW | -0.374 | -0.376 | 0.192 | -0.018 | 1.048 | -0.001 | 0.939 | 3.390 | 0.947 | 3.169 | 0.937 | 3.447 | 3.92 | 0 | 36.988 | 0.754 |
| | 2 | WAIPW | -0.374 | -0.376 | 0.126 | -0.018 | 1.029 | -0.002 | 0.945 | 3.235 | 0.948 | 3.152 | 0.942 | 3.311 | 3.92 | 0 | 15.858 | 0.314 |
| WIPW(0.25) | 2 | IPW | -0.374 | -0.375 | 0.096 | -0.009 | 1.006 | -0.001 | 0.948 | 3.135 | 0.952 | 3.012 | 0.948 | 3.129 | 3.92 | 0 | 9.270 | 0.186 |
| | 2 | WIPW | -0.374 | -0.375 | 0.095 | -0.008 | 1.003 | -0.001 | 0.948 | 3.141 | 0.952 | 3.036 | 0.950 | 3.094 | 3.92 | 0 | 8.999 | 0.180 |
| | 2 | AIPW | -0.374 | -0.375 | 0.079 | -0.015 | 1.009 | -0.001 | 0.947 | 3.175 | 0.946 | 3.186 | 0.951 | 3.065 | 3.92 | 0 | 6.286 | 0.125 |
| | 2 | WAIPW | -0.374 | -0.375 | 0.079 | -0.014 | 1.007 | -0.001 | 0.949 | 3.118 | 0.947 | 3.163 | 0.951 | 3.065 | 3.92 | 0 | 6.265 | 0.123 |
| WIPW(0.5) | 2 | IPW | -0.374 | -0.375 | 0.120 | -0.012 | 1.024 | -0.001 | 0.944 | 3.257 | 0.947 | 3.169 | 0.947 | 3.157 | 3.92 | 0 | 14.496 | 0.288 |
| | 2 | WIPW | -0.374 | -0.375 | 0.111 | -0.009 | 1.020 | -0.001 | 0.944 | 3.252 | 0.946 | 3.202 | 0.947 | 3.180 | 3.92 | 0 | 12.315 | 0.245 |
| | 2 | AIPW | -0.374 | -0.375 | 0.097 | -0.011 | 1.022 | -0.001 | 0.944 | 3.263 | 0.950 | 3.094 | 0.945 | 3.213 | 3.92 | 0 | 9.411 | 0.183 |
| | 2 | WAIPW | -0.374 | -0.375 | 0.091 | -0.009 | 1.017 | -0.001 | 0.946 | 3.197 | 0.948 | 3.152 | 0.945 | 3.213 | 3.92 | 0 | 8.369 | 0.163 |
| WIPW(0.75) | 2 | IPW | -0.374 | -0.374 | 0.167 | -0.001 | 1.049 | 0.000 | 0.936 | 3.467 | 0.942 | 3.296 | 0.939 | 3.375 | 3.92 | 0 | 27.921 | 0.565 |
| | 2 | WIPW | -0.374 | -0.375 | 0.133 | -0.005 | 1.024 | -0.001 | 0.943 | 3.279 | 0.947 | 3.175 | 0.945 | 3.219 | 3.92 | 0 | 17.630 | 0.354 |
| | 2 | AIPW | -0.374 | -0.374 | 0.133 | -0.008 | 1.038 | 0.000 | 0.939 | 3.380 | 0.945 | 3.236 | 0.946 | 3.186 | 3.92 | 0 | 17.711 | 0.354 |
| | 2 | WAIPW | -0.374 | -0.375 | 0.110 | -0.011 | 1.027 | -0.001 | 0.942 | 3.317 | 0.945 | 3.225 | 0.946 | 3.197 | 3.92 | 0 | 12.085 | 0.241 |
| WIPW(1) | 2 | IPW | -0.374 | -0.374 | 0.240 | 0.001 | 1.102 | 0.000 | 0.923 | 3.766 | 0.932 | 3.556 | 0.933 | 3.531 | 3.92 | 0 | 57.433 | 1.147 |
| | 2 | WIPW | -0.374 | -0.375 | 0.148 | -0.003 | 1.016 | 0.000 | 0.946 | 3.208 | 0.943 | 3.274 | 0.949 | 3.106 | 3.92 | 0 | 22.030 | 0.436 |
| | 2 | AIPW | -0.374 | -0.374 | 0.190 | -0.009 | 1.034 | 0.000 | 0.946 | 3.186 | 0.944 | 3.263 | 0.944 | 3.263 | 3.92 | 0 | 36.262 | 0.743 |
| | 2 | WAIPW | -0.374 | -0.375 | 0.124 | -0.013 | 1.015 | -0.001 | 0.947 | 3.180 | 0.947 | 3.163 | 0.945 | 3.235 | 3.92 | 0 | 15.346 | 0.304 |
| AIPW(0.25) | 3 | IPW | -0.500 | -0.500 | 0.096 | -0.009 | 0.991 | -0.001 | 0.953 | 3.006 | 0.952 | 3.012 | 0.952 | 3.030 | 3.92 | 0 | 9.301 | 0.185 |
| | 3 | WIPW | -0.500 | -0.500 | 0.120 | -0.006 | 1.000 | 0.000 | 0.949 | 3.100 | 0.950 | 3.077 | 0.950 | 3.089 | 3.92 | 0 | 14.473 | 0.289 |
| | 3 | AIPW | -0.500 | -0.500 | 0.079 | -0.010 | 0.995 | -0.001 | 0.951 | 3.059 | 0.954 | 2.963 | 0.949 | 3.100 | 3.92 | 0 | 6.208 | 0.126 |
| | 3 | WAIPW | -0.500 | -0.500 | 0.079 | -0.010 | 0.997 | -0.001 | 0.951 | 3.065 | 0.954 | 2.969 | 0.950 | 3.089 | 3.92 | 0 | 6.208 | 0.125 |
| AIPW(0.5) | 3 | IPW | -0.500 | -0.498 | 0.123 | 0.010 | 1.020 | 0.001 | 0.945 | 3.224 | 0.946 | 3.208 | 0.948 | 3.140 | 3.92 | 0 | 15.020 | 0.301 |
| | 3 | WIPW | -0.500 | -0.498 | 0.160 | 0.010 | 1.022 | 0.002 | 0.948 | 3.152 | 0.946 | 3.197 | 0.946 | 3.197 | 3.92 | 0 | 25.624 | 0.513 |
| | 3 | AIPW | -0.500 | -0.500 | 0.098 | -0.003 | 1.025 | 0.000 | 0.945 | 3.230 | 0.946 | 3.202 | 0.946 | 3.202 | 3.92 | 0 | 9.638 | 0.190 |
| | 3 | WAIPW | -0.500 | -0.500 | 0.093 | -0.007 | 1.024 | -0.001 | 0.945 | 3.235 | 0.947 | 3.157 | 0.943 | 3.284 | 3.92 | 0 | 8.591 | 0.169 |
| AIPW(0.75) | 3 | IPW | -0.500 | -0.499 | 0.167 | 0.001 | 1.034 | 0.000 | 0.939 | 3.396 | 0.944 | 3.263 | 0.946 | 3.203 | 3.92 | 0 | 28.026 | 0.582 |
| | 3 | WIPW | -0.500 | -0.498 | 0.219 | 0.004 | 1.052 | 0.001 | 0.933 | 3.527 | 0.941 | 3.343 | 0.945 | 3.214 | 3.92 | 0 | 47.769 | 0.981 |
| | 3 | AIPW | -0.500 | -0.497 | 0.131 | 0.023 | 1.009 | 0.002 | 0.945 | 3.236 | 0.943 | 3.290 | 0.952 | 3.030 | 3.92 | 0 | 17.147 | 0.340 |
| | 3 | WAIPW | -0.500 | -0.498 | 0.108 | 0.013 | 1.001 | 0.001 | 0.948 | 3.146 | 0.945 | 3.214 | 0.952 | 3.018 | 3.92 | 0 | 11.720 | 0.230 |
| AIPW(1) | 3 | IPW | -0.500 | -0.496 | 0.248 | 0.012 | 1.111 | 0.004 | 0.918 | 3.876 | 0.927 | 3.684 | 0.936 | 3.472 | 3.92 | 0 | 61.437 | 1.268 |
| | 3 | WIPW | -0.500 | -0.494 | 0.317 | 0.014 | 1.152 | 0.006 | 0.910 | 4.052 | 0.922 | 3.784 | 0.929 | 3.637 | 3.92 | 0 | 100.193 | 2.105 |
| | 3 | AIPW | -0.500 | -0.499 | 0.190 | 0.011 | 1.030 | 0.000 | 0.944 | 3.246 | 0.940 | 3.348 | 0.948 | 3.135 | 3.92 | 0 | 35.987 | 0.740 |
| | 3 | WAIPW | -0.500 | -0.500 | 0.126 | -0.004 | 1.024 | -0.001 | 0.940 | 3.354 | 0.948 | 3.152 | 0.947 | 3.174 | 3.92 | 0 | 15.826 | 0.331 |
| AR-1 | 3 | IPW | -0.500 | -0.522 | 0.414 | -0.153 | 1.386 | -0.022 | 0.850 | 5.053 | 0.914 | 3.970 | 0.868 | 4.794 | 3.92 | 0 | 171.763 | 4.116 |
| | 3 | WIPW | -0.500 | -0.510 | 0.260 | -0.101 | 1.164 | -0.011 | 0.908 | 4.084 | 0.939 | 3.385 | 0.903 | 4.194 | 3.92 | 0 | 67.707 | 1.566 |
| | 3 | AIPW | -0.500 | -0.504 | 0.334 | -0.056 | 1.074 | -0.004 | 0.946 | 3.186 | 0.955 | 2.932 | 0.933 | 3.546 | 3.92 | 0 | 111.225 | 3.149 |
| | 3 | WAIPW | -0.500 | -0.505 | 0.240 | -0.053 | 1.058 | -0.005 | 0.946 | 3.197 | 0.953 | 2.999 | 0.936 | 3.472 | 3.92 | 0 | 57.669 | 1.694 |
| AR-2 | 3 | IPW | -0.500 | -0.499 | 0.291 | -0.021 | 1.169 | 0.001 | 0.906 | 4.135 | 0.929 | 3.623 | 0.913 | 3.994 | 3.92 | 0 | 84.648 | 1.804 |

| Randomization Method | Regime | Estimator | True Value x 10^3 | Mean Estimate x 10^3 | SD Estimates x 10^3 | Mean Normalized | SD Normalized | Mean Bias | CI Coverage | SE CI Coverage x 10^3 | LB Coverage | SE LB Coverage x 10^3 | UB Coverage x 10^3 | SE UB Coverage x 10^3 | Mean CI Length | SE CI Length x 10^3 | MSE x 10^3 | SE MSE x 10^3 |
|---|---|---|---|---|---|---|---|---|---|---|---|---|---|---|---|---|---|---|
| | 3 | WIPW | -0.500 | -0.496 | 0.190 | 0.008 | 1.061 | 0.003 | 0.935 | 3.492 | 0.943 | 3.274 | 0.938 | 3.401 | 3.92 | 0 | 36.072 | 0.767 |
| | 3 | AIPW | -0.500 | -0.494 | 0.226 | 0.020 | 1.046 | 0.005 | 0.943 | 3.268 | 0.944 | 3.263 | 0.941 | 3.343 | 3.92 | 0 | 50.974 | 1.172 |
| | 3 | WAIPW | -0.500 | -0.495 | 0.171 | 0.021 | 1.029 | 0.004 | 0.951 | 3.053 | 0.945 | 3.213 | 0.946 | 3.191 | 3.92 | 0 | 29.154 | 0.644 |
| IAIPW(0.25) | 3 | IPW | -0.500 | -0.500 | 0.097 | -0.007 | 1.000 | -0.001 | 0.948 | 3.146 | 0.951 | 3.042 | 0.947 | 3.169 | 3.92 | 0 | 9.453 | 0.192 |
| | 3 | WIPW | -0.500 | -0.500 | 0.096 | -0.005 | 1.003 | 0.000 | 0.950 | 3.071 | 0.952 | 3.024 | 0.947 | 3.169 | 3.92 | 0 | 9.272 | 0.187 |
| | 3 | AIPW | -0.500 | -0.501 | 0.080 | -0.017 | 1.008 | -0.001 | 0.945 | 3.230 | 0.954 | 2.975 | 0.946 | 3.191 | 3.92 | 0 | 6.352 | 0.129 |
| | 3 | WAIPW | -0.500 | -0.501 | 0.080 | -0.015 | 1.007 | -0.001 | 0.948 | 3.129 | 0.952 | 3.018 | 0.946 | 3.203 | 3.92 | 0 | 6.330 | 0.128 |
| IAIPW(0.5) | 3 | IPW | -0.500 | -0.500 | 0.123 | -0.005 | 1.027 | -0.001 | 0.942 | 3.311 | 0.944 | 3.257 | 0.946 | 3.197 | 3.92 | 0 | 15.163 | 0.307 |
| | 3 | WIPW | -0.500 | -0.501 | 0.113 | -0.008 | 1.018 | -0.001 | 0.944 | 3.257 | 0.947 | 3.180 | 0.946 | 3.202 | 3.92 | 0 | 12.761 | 0.256 |
| | 3 | AIPW | -0.500 | -0.500 | 0.099 | -0.005 | 1.036 | -0.001 | 0.939 | 3.390 | 0.946 | 3.186 | 0.943 | 3.279 | 3.92 | 0 | 9.862 | 0.201 |
| | 3 | WAIPW | -0.500 | -0.501 | 0.093 | -0.009 | 1.028 | -0.001 | 0.940 | 3.364 | 0.946 | 3.208 | 0.946 | 3.202 | 3.92 | 0 | 8.687 | 0.174 |
| IAIPW(0.75) | 3 | IPW | -0.500 | -0.504 | 0.171 | -0.025 | 1.052 | -0.004 | 0.938 | 3.411 | 0.944 | 3.252 | 0.940 | 3.354 | 3.92 | 0 | 29.136 | 0.595 |
| | 3 | WIPW | -0.500 | -0.503 | 0.137 | -0.025 | 1.038 | -0.004 | 0.941 | 3.343 | 0.945 | 3.219 | 0.942 | 3.312 | 3.92 | 0 | 18.816 | 0.381 |
| | 3 | AIPW | -0.500 | -0.500 | 0.132 | -0.002 | 1.022 | -0.001 | 0.945 | 3.230 | 0.945 | 3.219 | 0.953 | 3.006 | 3.92 | 0 | 17.391 | 0.359 |
| | 3 | WAIPW | -0.500 | -0.500 | 0.109 | -0.004 | 1.014 | -0.001 | 0.947 | 3.175 | 0.944 | 3.263 | 0.951 | 3.065 | 3.92 | 0 | 11.957 | 0.244 |
| IAIPW(1) | 3 | IPW | -0.500 | -0.499 | 0.247 | 0.003 | 1.125 | 0.000 | 0.918 | 3.889 | 0.925 | 3.716 | 0.936 | 3.467 | 3.92 | 0 | 61.087 | 1.198 |
| | 3 | WIPW | -0.500 | -0.500 | 0.154 | -0.007 | 1.046 | -0.001 | 0.938 | 3.416 | 0.941 | 3.333 | 0.939 | 3.375 | 3.92 | 0 | 23.754 | 0.465 |
| | 3 | AIPW | -0.500 | -0.499 | 0.193 | 0.009 | 1.043 | 0.001 | 0.943 | 3.290 | 0.936 | 3.452 | 0.945 | 3.224 | 3.92 | 0 | 37.250 | 0.736 |
| | 3 | WAIPW | -0.500 | -0.500 | 0.126 | -0.003 | 1.026 | 0.000 | 0.945 | 3.224 | 0.941 | 3.322 | 0.946 | 3.186 | 3.92 | 0 | 15.827 | 0.309 |
| IPW(0.25) | 3 | IPW | -0.500 | -0.501 | 0.099 | -0.020 | 1.012 | -0.002 | 0.947 | 3.169 | 0.952 | 3.030 | 0.946 | 3.186 | 3.92 | 0 | 9.786 | 0.202 |
| | 3 | WIPW | -0.500 | -0.501 | 0.123 | -0.013 | 1.020 | -0.002 | 0.947 | 3.169 | 0.950 | 3.077 | 0.940 | 3.354 | 3.92 | 0 | 15.214 | 0.312 |
| | 3 | AIPW | -0.500 | -0.502 | 0.080 | -0.028 | 1.008 | -0.002 | 0.953 | 2.988 | 0.949 | 3.100 | 0.949 | 3.106 | 3.92 | 0 | 6.411 | 0.133 |
| | 3 | WAIPW | -0.500 | -0.502 | 0.080 | -0.029 | 1.004 | -0.002 | 0.954 | 2.957 | 0.952 | 3.024 | 0.952 | 3.030 | 3.92 | 0 | 6.337 | 0.132 |
| IPW(0.5) | 3 | IPW | -0.500 | -0.501 | 0.122 | -0.011 | 1.016 | -0.001 | 0.946 | 3.202 | 0.944 | 3.263 | 0.949 | 3.117 | 3.92 | 0 | 14.904 | 0.301 |
| | 3 | WIPW | -0.500 | -0.501 | 0.160 | -0.009 | 1.023 | -0.001 | 0.944 | 3.257 | 0.939 | 3.375 | 0.949 | 3.123 | 3.92 | 0 | 25.571 | 0.526 |
| | 3 | AIPW | -0.500 | -0.501 | 0.098 | -0.008 | 1.024 | -0.001 | 0.944 | 3.241 | 0.945 | 3.213 | 0.950 | 3.077 | 3.92 | 0 | 9.663 | 0.192 |
| | 3 | WAIPW | -0.500 | -0.501 | 0.093 | -0.012 | 1.019 | -0.001 | 0.948 | 3.152 | 0.947 | 3.174 | 0.949 | 3.106 | 3.92 | 0 | 8.557 | 0.169 |
| IPW(0.75) | 3 | IPW | -0.500 | -0.499 | 0.167 | 0.003 | 1.026 | 0.000 | 0.944 | 3.252 | 0.947 | 3.169 | 0.947 | 3.158 | 3.92 | 0 | 27.893 | 0.592 |
| | 3 | WIPW | -0.500 | -0.500 | 0.219 | 0.000 | 1.042 | 0.000 | 0.941 | 3.338 | 0.946 | 3.186 | 0.948 | 3.129 | 3.92 | 0 | 47.889 | 1.025 |
| | 3 | AIPW | -0.500 | -0.498 | 0.131 | 0.017 | 1.010 | 0.002 | 0.949 | 3.100 | 0.945 | 3.230 | 0.950 | 3.071 | 3.92 | 0 | 17.185 | 0.343 |
| | 3 | WAIPW | -0.500 | -0.498 | 0.109 | 0.019 | 1.006 | 0.002 | 0.948 | 3.141 | 0.946 | 3.208 | 0.950 | 3.094 | 3.92 | 0 | 11.845 | 0.238 |
| IPW(1) | 3 | IPW | -0.500 | -0.502 | 0.241 | -0.013 | 1.099 | -0.002 | 0.923 | 3.762 | 0.938 | 3.416 | 0.935 | 3.492 | 3.92 | 0 | 58.254 | 1.157 |
| | 3 | WIPW | -0.500 | -0.503 | 0.309 | -0.017 | 1.145 | -0.003 | 0.914 | 3.970 | 0.932 | 3.565 | 0.927 | 3.679 | 3.92 | 0 | 95.441 | 1.912 |
| | 3 | AIPW | -0.500 | -0.500 | 0.193 | 0.014 | 1.052 | 0.000 | 0.939 | 3.375 | 0.941 | 3.338 | 0.941 | 3.322 | 3.92 | 0 | 37.277 | 0.766 |
| | 3 | WAIPW | -0.500 | -0.499 | 0.126 | 0.008 | 1.030 | 0.000 | 0.946 | 3.191 | 0.944 | 3.246 | 0.946 | 3.197 | 3.92 | 0 | 15.921 | 0.314 |
| SR | 3 | IPW | -0.500 | -0.499 | 0.086 | 0.006 | 1.017 | 0.000 | 0.943 | 3.290 | 0.951 | 3.065 | 0.946 | 3.202 | 3.92 | 0 | 7.439 | 0.151 |
| | 3 | WIPW | -0.500 | -0.498 | 0.103 | 0.015 | 1.025 | 0.001 | 0.944 | 3.246 | 0.947 | 3.174 | 0.949 | 3.117 | 3.92 | 0 | 10.701 | 0.212 |
| | 3 | AIPW | -0.500 | -0.499 | 0.071 | 0.004 | 1.016 | 0.000 | 0.947 | 3.174 | 0.947 | 3.169 | 0.947 | 3.163 | 3.92 | 0 | 5.107 | 0.106 |
| | 3 | WAIPW | -0.500 | -0.499 | 0.072 | 0.006 | 1.017 | 0.000 | 0.950 | 3.083 | 0.947 | 3.157 | 0.949 | 3.112 | 3.92 | 0 | 5.216 | 0.109 |
| TS(0.25) | 3 | IPW | -0.500 | -0.501 | 0.359 | 0.025 | 1.292 | -0.001 | 0.879 | 4.616 | 0.894 | 4.350 | 0.905 | 4.151 | 3.92 | 0 | 128.631 | 2.718 |
| | 3 | WIPW | -0.500 | -0.503 | 0.140 | -0.038 | 1.023 | -0.003 | 0.946 | 3.208 | 0.951 | 3.047 | 0.944 | 3.246 | 3.92 | 0 | 19.723 | 0.407 |
| | 3 | AIPW | -0.500 | -0.501 | 0.295 | 0.012 | 1.076 | -0.002 | 0.947 | 3.157 | 0.941 | 3.327 | 0.945 | 3.230 | 3.92 | 0 | 87.128 | 2.018 |
| | 3 | WAIPW | -0.500 | -0.503 | 0.128 | -0.042 | 1.037 | -0.003 | 0.945 | 3.213 | 0.945 | 3.230 | 0.943 | 3.290 | 3.92 | 0 | 16.268 | 0.368 |
| TS(0.50) | 3 | IPW | -0.500 | -0.493 | 0.339 | 0.026 | 1.289 | 0.006 | 0.881 | 4.576 | 0.904 | 4.174 | 0.912 | 4.011 | 3.92 | 0 | 114.954 | 2.576 |

| Randomization Method | Regime | Estimator | True Value x 10^3 | Mean Estimate x 10^3 | SD Estimates x 10^3 | Mean Normalized | SD Normalized | Mean Bias | CI Coverage | SE CI Coverage x 10^3 | LB Coverage | SE LB Coverage x 10^3 | UB Coverage x 10^3 | SE UB Coverage x 10^3 | Mean CI Length | SE CI Length x 10^3 | MSE x 10^3 | SE MSE x 10^3 |
|---|---|---|---|---|---|---|---|---|---|---|---|---|---|---|---|---|---|---|
| | 3 | WIPW | -0.500 | -0.501 | 0.137 | -0.023 | 1.026 | -0.002 | 0.942 | 3.311 | 0.946 | 3.186 | 0.944 | 3.241 | 3.92 | 0 | 18.716 | 0.397 |
| | 3 | AIPW | -0.500 | -0.497 | 0.275 | 0.019 | 1.052 | 0.003 | 0.949 | 3.106 | 0.943 | 3.268 | 0.951 | 3.047 | 3.92 | 0 | 75.346 | 1.805 |
| | 3 | WAIPW | -0.500 | -0.501 | 0.115 | -0.025 | 1.010 | -0.002 | 0.949 | 3.123 | 0.950 | 3.088 | 0.947 | 3.174 | 3.92 | 0 | 13.337 | 0.297 |
| TS(0.75) | 3 | IPW | -0.500 | -0.511 | 0.338 | -0.026 | 1.267 | -0.012 | 0.885 | 4.509 | 0.908 | 4.096 | 0.903 | 4.190 | 3.92 | 0 | 114.326 | 2.510 |
| | 3 | WIPW | -0.500 | -0.504 | 0.137 | -0.031 | 1.013 | -0.004 | 0.946 | 3.208 | 0.950 | 3.094 | 0.947 | 3.180 | 3.92 | 0 | 18.842 | 0.404 |
| | 3 | AIPW | -0.500 | -0.512 | 0.278 | -0.034 | 1.072 | -0.013 | 0.950 | 3.083 | 0.941 | 3.333 | 0.939 | 3.380 | 3.92 | 0 | 77.665 | 1.816 |
| | 3 | WAIPW | -0.500 | -0.504 | 0.116 | -0.043 | 1.016 | -0.004 | 0.946 | 3.186 | 0.952 | 3.029 | 0.942 | 3.311 | 3.92 | 0 | 13.561 | 0.312 |
| TS(1) | 3 | IPW | -0.500 | -0.501 | 0.335 | 0.009 | 1.256 | -0.001 | 0.882 | 4.556 | 0.908 | 4.092 | 0.909 | 4.068 | 3.92 | 0 | 112.085 | 2.582 |
| | 3 | WIPW | -0.500 | -0.508 | 0.141 | -0.060 | 1.022 | -0.008 | 0.944 | 3.263 | 0.954 | 2.969 | 0.940 | 3.348 | 3.92 | 0 | 19.905 | 0.410 |
| | 3 | AIPW | -0.500 | -0.502 | 0.277 | -0.007 | 1.057 | -0.003 | 0.947 | 3.169 | 0.945 | 3.230 | 0.946 | 3.186 | 3.92 | 0 | 76.641 | 2.035 |
| | 3 | WAIPW | -0.500 | -0.505 | 0.119 | -0.055 | 1.031 | -0.006 | 0.940 | 3.364 | 0.952 | 3.017 | 0.939 | 3.385 | 3.92 | 0 | 14.142 | 0.316 |
| WAIPW(0.25) | 3 | IPW | -0.500 | -0.500 | 0.099 | -0.010 | 1.016 | -0.001 | 0.944 | 3.241 | 0.950 | 3.083 | 0.949 | 3.106 | 3.92 | 0 | 9.754 | 0.194 |
| | 3 | WIPW | -0.500 | -0.500 | 0.097 | -0.010 | 1.016 | -0.001 | 0.945 | 3.225 | 0.947 | 3.180 | 0.951 | 3.048 | 3.92 | 0 | 9.504 | 0.188 |
| | 3 | AIPW | -0.500 | -0.500 | 0.080 | -0.003 | 1.015 | 0.000 | 0.946 | 3.208 | 0.948 | 3.152 | 0.950 | 3.089 | 3.92 | 0 | 6.440 | 0.132 |
| | 3 | WAIPW | -0.500 | -0.500 | 0.080 | -0.001 | 1.017 | 0.000 | 0.945 | 3.214 | 0.948 | 3.146 | 0.948 | 3.135 | 3.92 | 0 | 6.457 | 0.131 |
| WAIPW(0.5) | 3 | IPW | -0.500 | -0.502 | 0.122 | -0.025 | 1.019 | -0.003 | 0.941 | 3.343 | 0.949 | 3.100 | 0.942 | 3.301 | 3.92 | 0 | 14.860 | 0.300 |
| | 3 | WIPW | -0.500 | -0.502 | 0.112 | -0.021 | 1.008 | -0.002 | 0.942 | 3.306 | 0.952 | 3.035 | 0.943 | 3.290 | 3.92 | 0 | 12.507 | 0.252 |
| | 3 | AIPW | -0.500 | -0.502 | 0.098 | -0.021 | 1.026 | -0.002 | 0.944 | 3.257 | 0.946 | 3.202 | 0.943 | 3.284 | 3.92 | 0 | 9.636 | 0.193 |
| | 3 | WAIPW | -0.500 | -0.501 | 0.092 | -0.016 | 1.014 | -0.002 | 0.945 | 3.213 | 0.947 | 3.163 | 0.946 | 3.202 | 3.92 | 0 | 8.465 | 0.170 |
| WAIPW(0.75) | 3 | IPW | -0.500 | -0.503 | 0.169 | -0.020 | 1.046 | -0.003 | 0.937 | 3.442 | 0.947 | 3.163 | 0.940 | 3.364 | 3.92 | 0 | 28.594 | 0.575 |
| | 3 | WIPW | -0.500 | -0.502 | 0.135 | -0.018 | 1.019 | -0.002 | 0.947 | 3.180 | 0.951 | 3.065 | 0.946 | 3.208 | 3.92 | 0 | 18.149 | 0.370 |
| | 3 | AIPW | -0.500 | -0.502 | 0.131 | -0.015 | 1.008 | -0.002 | 0.944 | 3.252 | 0.951 | 3.059 | 0.949 | 3.118 | 3.92 | 0 | 17.053 | 0.350 |
| | 3 | WAIPW | -0.500 | -0.502 | 0.108 | -0.021 | 1.002 | -0.002 | 0.949 | 3.118 | 0.953 | 2.994 | 0.950 | 3.077 | 3.92 | 0 | 11.693 | 0.245 |
| WAIPW(1) | 3 | IPW | -0.500 | -0.501 | 0.244 | -0.007 | 1.094 | -0.002 | 0.927 | 3.670 | 0.934 | 3.502 | 0.934 | 3.507 | 3.92 | 0 | 59.414 | 1.183 |
| | 3 | WIPW | -0.500 | -0.503 | 0.151 | -0.020 | 1.009 | -0.003 | 0.949 | 3.106 | 0.951 | 3.041 | 0.945 | 3.230 | 3.92 | 0 | 22.795 | 0.460 |
| | 3 | AIPW | -0.500 | -0.501 | 0.192 | 0.005 | 1.033 | -0.002 | 0.945 | 3.230 | 0.944 | 3.263 | 0.942 | 3.301 | 3.92 | 0 | 36.682 | 0.774 |
| | 3 | WAIPW | -0.500 | -0.502 | 0.124 | -0.014 | 1.010 | -0.003 | 0.944 | 3.241 | 0.950 | 3.071 | 0.949 | 3.117 | 3.92 | 0 | 15.466 | 0.319 |
| WIPW(0.25) | 3 | IPW | -0.500 | -0.499 | 0.099 | 0.002 | 1.011 | 0.000 | 0.948 | 3.146 | 0.948 | 3.152 | 0.947 | 3.158 | 3.92 | 0 | 9.734 | 0.195 |
| | 3 | WIPW | -0.500 | -0.499 | 0.097 | 0.006 | 1.010 | 0.001 | 0.949 | 3.123 | 0.949 | 3.123 | 0.948 | 3.146 | 3.92 | 0 | 9.468 | 0.190 |
| | 3 | AIPW | -0.500 | -0.499 | 0.080 | 0.003 | 1.007 | 0.000 | 0.951 | 3.042 | 0.952 | 3.012 | 0.946 | 3.186 | 3.92 | 0 | 6.369 | 0.127 |
| | 3 | WAIPW | -0.500 | -0.499 | 0.080 | 0.007 | 1.005 | 0.001 | 0.951 | 3.059 | 0.952 | 3.030 | 0.946 | 3.191 | 3.92 | 0 | 6.331 | 0.127 |
| WIPW(0.5) | 3 | IPW | -0.500 | -0.498 | 0.123 | 0.009 | 1.026 | 0.001 | 0.947 | 3.174 | 0.943 | 3.290 | 0.944 | 3.252 | 3.92 | 0 | 15.214 | 0.306 |
| | 3 | WIPW | -0.500 | -0.498 | 0.113 | 0.010 | 1.020 | 0.001 | 0.944 | 3.246 | 0.947 | 3.163 | 0.945 | 3.213 | 3.92 | 0 | 12.877 | 0.257 |
| | 3 | AIPW | -0.500 | -0.498 | 0.098 | 0.013 | 1.019 | 0.001 | 0.947 | 3.180 | 0.947 | 3.174 | 0.947 | 3.180 | 3.92 | 0 | 9.649 | 0.192 |
| | 3 | WAIPW | -0.500 | -0.498 | 0.092 | 0.014 | 1.014 | 0.001 | 0.948 | 3.140 | 0.949 | 3.123 | 0.949 | 3.123 | 3.92 | 0 | 8.514 | 0.167 |
| WIPW(0.75) | 3 | IPW | -0.500 | -0.500 | 0.172 | -0.004 | 1.056 | -0.001 | 0.938 | 3.411 | 0.945 | 3.236 | 0.943 | 3.285 | 3.92 | 0 | 29.625 | 0.597 |
| | 3 | WIPW | -0.500 | -0.501 | 0.136 | -0.009 | 1.023 | -0.001 | 0.949 | 3.123 | 0.946 | 3.186 | 0.948 | 3.152 | 3.92 | 0 | 18.455 | 0.370 |
| | 3 | AIPW | -0.500 | -0.501 | 0.134 | -0.001 | 1.023 | -0.001 | 0.949 | 3.106 | 0.944 | 3.252 | 0.951 | 3.065 | 3.92 | 0 | 17.913 | 0.363 |
| | 3 | WAIPW | -0.500 | -0.501 | 0.110 | -0.007 | 1.012 | -0.001 | 0.951 | 3.059 | 0.948 | 3.146 | 0.948 | 3.129 | 3.92 | 0 | 12.127 | 0.247 |
| WIPW(1) | 3 | IPW | -0.500 | -0.503 | 0.249 | -0.013 | 1.127 | -0.003 | 0.919 | 3.850 | 0.930 | 3.609 | 0.928 | 3.651 | 3.92 | 0 | 61.912 | 1.236 |
| | 3 | WIPW | -0.500 | -0.502 | 0.156 | -0.018 | 1.041 | -0.002 | 0.937 | 3.447 | 0.944 | 3.246 | 0.942 | 3.295 | 3.92 | 0 | 24.221 | 0.497 |
| | 3 | AIPW | -0.500 | -0.500 | 0.193 | 0.010 | 1.029 | -0.001 | 0.943 | 3.284 | 0.939 | 3.385 | 0.946 | 3.202 | 3.92 | 0 | 37.077 | 0.763 |
| | 3 | WAIPW | -0.500 | -0.501 | 0.126 | -0.003 | 1.014 | -0.001 | 0.947 | 3.169 | 0.947 | 3.157 | 0.948 | 3.129 | 3.92 | 0 | 15.826 | 0.323 |
| AIPW(0.25) | 4 | IPW | -0.251 | -0.250 | 0.105 | 0.010 | 1.013 | 0.001 | 0.944 | 3.241 | 0.943 | 3.269 | 0.946 | 3.197 | 3.92 | 0 | 10.966 | 0.216 |

| Randomization Method | Regime | Estimator | True Value x 10^3 | Mean Estimate x 10^3 | SD Estimates x 10^3 | Mean Normalized | SD Normalized | Mean Bias | CI Coverage | SE CI Coverage x 10^3 | LB Coverage | SE LB Coverage x 10^3 | UB Coverage x 10^3 | SE UB Coverage x 10^3 | Mean CI Length | SE CI Length x 10^3 | MSE x 10^3 | SE MSE x 10^3 |
|---|---|---|---|---|---|---|---|---|---|---|---|---|---|---|---|---|---|---|
| | 4 | WIPW | -0.251 | -0.250 | 0.131 | 0.006 | 1.025 | 0.001 | 0.943 | 3.290 | 0.944 | 3.252 | 0.946 | 3.186 | 3.92 | 0 | 17.170 | 0.339 |
| | 4 | AIPW | -0.251 | -0.251 | 0.084 | -0.001 | 1.024 | 0.000 | 0.944 | 3.252 | 0.945 | 3.214 | 0.947 | 3.180 | 3.92 | 0 | 7.098 | 0.139 |
| | 4 | WAIPW | -0.251 | -0.251 | 0.084 | -0.002 | 1.022 | 0.000 | 0.948 | 3.129 | 0.948 | 3.152 | 0.950 | 3.083 | 3.92 | 0 | 7.022 | 0.138 |
| AIPW(0.5) | 4 | IPW | -0.251 | -0.250 | 0.129 | 0.005 | 1.008 | 0.001 | 0.948 | 3.135 | 0.951 | 3.047 | 0.947 | 3.169 | 3.92 | 0 | 16.530 | 0.333 |
| | 4 | WIPW | -0.251 | -0.251 | 0.169 | 0.000 | 1.016 | 0.000 | 0.946 | 3.202 | 0.950 | 3.077 | 0.946 | 3.191 | 3.92 | 0 | 28.419 | 0.578 |
| | 4 | AIPW | -0.251 | -0.251 | 0.101 | -0.003 | 1.009 | 0.000 | 0.950 | 3.083 | 0.949 | 3.100 | 0.948 | 3.129 | 3.92 | 0 | 10.157 | 0.198 |
| | 4 | WAIPW | -0.251 | -0.251 | 0.095 | -0.001 | 1.007 | 0.000 | 0.951 | 3.053 | 0.950 | 3.094 | 0.947 | 3.169 | 3.92 | 0 | 9.011 | 0.176 |
| AIPW(0.75) | 4 | IPW | -0.251 | -0.247 | 0.177 | 0.026 | 1.023 | 0.004 | 0.945 | 3.214 | 0.945 | 3.225 | 0.950 | 3.089 | 3.92 | 0 | 31.197 | 0.630 |
| | 4 | WIPW | -0.251 | -0.245 | 0.232 | 0.026 | 1.044 | 0.006 | 0.937 | 3.447 | 0.943 | 3.285 | 0.945 | 3.219 | 3.92 | 0 | 53.695 | 1.107 |
| | 4 | AIPW | -0.251 | -0.245 | 0.137 | 0.049 | 1.024 | 0.006 | 0.944 | 3.247 | 0.939 | 3.390 | 0.952 | 3.024 | 3.92 | 0 | 18.858 | 0.381 |
| | 4 | WAIPW | -0.251 | -0.246 | 0.113 | 0.044 | 1.013 | 0.005 | 0.948 | 3.146 | 0.942 | 3.301 | 0.954 | 2.963 | 3.92 | 0 | 12.895 | 0.264 |
| AIPW(1) | 4 | IPW | -0.251 | -0.247 | 0.261 | 0.016 | 1.115 | 0.004 | 0.921 | 3.806 | 0.934 | 3.512 | 0.931 | 3.585 | 3.92 | 0 | 68.197 | 1.372 |
| | 4 | WIPW | -0.251 | -0.246 | 0.333 | 0.015 | 1.160 | 0.005 | 0.913 | 3.994 | 0.930 | 3.604 | 0.928 | 3.661 | 3.92 | 0 | 111.144 | 2.265 |
| | 4 | AIPW | -0.251 | -0.248 | 0.197 | 0.026 | 1.024 | 0.003 | 0.946 | 3.191 | 0.942 | 3.317 | 0.952 | 3.011 | 3.92 | 0 | 38.993 | 0.818 |
| | 4 | WAIPW | -0.251 | -0.249 | 0.129 | 0.021 | 1.015 | 0.002 | 0.946 | 3.202 | 0.942 | 3.311 | 0.950 | 3.077 | 3.92 | 0 | 16.730 | 0.333 |
| AR-1 | 4 | IPW | -0.251 | -0.243 | 0.284 | 0.175 | 1.198 | 0.008 | 0.895 | 4.339 | 0.882 | 4.566 | 0.951 | 3.041 | 3.92 | 0 | 80.663 | 1.876 |
| | 4 | WIPW | -0.251 | -0.248 | 0.166 | 0.081 | 1.040 | 0.003 | 0.945 | 3.235 | 0.931 | 3.594 | 0.953 | 2.993 | 3.92 | 0 | 27.537 | 0.597 |
| | 4 | AIPW | -0.251 | -0.250 | 0.215 | 0.036 | 1.042 | 0.001 | 0.943 | 3.268 | 0.944 | 3.263 | 0.949 | 3.100 | 3.92 | 0 | 46.050 | 1.323 |
| | 4 | WAIPW | -0.251 | -0.246 | 0.133 | 0.078 | 1.026 | 0.005 | 0.941 | 3.343 | 0.938 | 3.416 | 0.951 | 3.041 | 3.92 | 0 | 17.583 | 0.399 |
| AR-2 | 4 | IPW | -0.251 | -0.241 | 0.238 | 0.119 | 1.109 | 0.010 | 0.922 | 3.784 | 0.913 | 3.994 | 0.952 | 3.017 | 3.92 | 0 | 56.515 | 1.150 |
| | 4 | WIPW | -0.251 | -0.245 | 0.155 | 0.080 | 1.032 | 0.006 | 0.946 | 3.197 | 0.933 | 3.531 | 0.955 | 2.920 | 3.92 | 0 | 23.903 | 0.488 |
| | 4 | AIPW | -0.251 | -0.246 | 0.182 | 0.056 | 1.040 | 0.005 | 0.943 | 3.279 | 0.935 | 3.497 | 0.952 | 3.017 | 3.92 | 0 | 33.044 | 0.697 |
| | 4 | WAIPW | -0.251 | -0.246 | 0.129 | 0.074 | 1.033 | 0.005 | 0.940 | 3.359 | 0.933 | 3.531 | 0.954 | 2.969 | 3.92 | 0 | 16.738 | 0.350 |
| IAIPW(0.25) | 4 | IPW | -0.251 | -0.252 | 0.102 | -0.012 | 0.989 | -0.001 | 0.948 | 3.135 | 0.951 | 3.059 | 0.951 | 3.065 | 3.92 | 0 | 10.406 | 0.212 |
| | 4 | WIPW | -0.251 | -0.252 | 0.101 | -0.007 | 0.988 | -0.001 | 0.950 | 3.094 | 0.951 | 3.053 | 0.953 | 3.000 | 3.92 | 0 | 10.134 | 0.207 |
| | 4 | AIPW | -0.251 | -0.252 | 0.082 | -0.010 | 0.995 | -0.001 | 0.950 | 3.094 | 0.951 | 3.065 | 0.953 | 2.981 | 3.92 | 0 | 6.684 | 0.133 |
| | 4 | WAIPW | -0.251 | -0.252 | 0.082 | -0.006 | 0.997 | -0.001 | 0.949 | 3.100 | 0.948 | 3.141 | 0.952 | 3.018 | 3.92 | 0 | 6.692 | 0.133 |
| IAIPW(0.5) | 4 | IPW | -0.251 | -0.248 | 0.129 | 0.017 | 1.013 | 0.003 | 0.951 | 3.065 | 0.950 | 3.077 | 0.949 | 3.112 | 3.92 | 0 | 16.667 | 0.337 |
| | 4 | WIPW | -0.251 | -0.248 | 0.119 | 0.020 | 1.008 | 0.003 | 0.951 | 3.059 | 0.950 | 3.083 | 0.950 | 3.083 | 3.92 | 0 | 14.138 | 0.285 |
| | 4 | AIPW | -0.251 | -0.248 | 0.100 | 0.031 | 1.005 | 0.003 | 0.947 | 3.157 | 0.940 | 3.348 | 0.953 | 2.993 | 3.92 | 0 | 10.013 | 0.203 |
| | 4 | WAIPW | -0.251 | -0.248 | 0.094 | 0.028 | 1.000 | 0.003 | 0.948 | 3.146 | 0.943 | 3.284 | 0.955 | 2.944 | 3.92 | 0 | 8.837 | 0.178 |
| IAIPW(0.75) | 4 | IPW | -0.251 | -0.249 | 0.181 | 0.013 | 1.049 | 0.002 | 0.935 | 3.497 | 0.942 | 3.301 | 0.940 | 3.354 | 3.92 | 0 | 32.851 | 0.675 |
| | 4 | WIPW | -0.251 | -0.250 | 0.145 | 0.006 | 1.030 | 0.001 | 0.937 | 3.442 | 0.945 | 3.236 | 0.942 | 3.301 | 3.92 | 0 | 21.049 | 0.422 |
| | 4 | AIPW | -0.251 | -0.248 | 0.139 | 0.027 | 1.037 | 0.003 | 0.940 | 3.370 | 0.938 | 3.401 | 0.948 | 3.141 | 3.92 | 0 | 19.407 | 0.397 |
| | 4 | WAIPW | -0.251 | -0.250 | 0.116 | 0.014 | 1.037 | 0.001 | 0.939 | 3.375 | 0.942 | 3.317 | 0.945 | 3.214 | 3.92 | 0 | 13.520 | 0.275 |
| IAIPW(1) | 4 | IPW | -0.251 | -0.249 | 0.263 | 0.017 | 1.125 | 0.002 | 0.919 | 3.855 | 0.929 | 3.642 | 0.932 | 3.565 | 3.92 | 0 | 69.091 | 1.381 |
| | 4 | WIPW | -0.251 | -0.248 | 0.163 | 0.020 | 1.035 | 0.003 | 0.940 | 3.369 | 0.945 | 3.235 | 0.947 | 3.174 | 3.92 | 0 | 26.663 | 0.549 |
| | 4 | AIPW | -0.251 | -0.250 | 0.202 | 0.019 | 1.044 | 0.001 | 0.938 | 3.411 | 0.937 | 3.441 | 0.948 | 3.129 | 3.92 | 0 | 40.652 | 0.812 |
| | 4 | WAIPW | -0.251 | -0.250 | 0.131 | 0.010 | 1.017 | 0.001 | 0.946 | 3.197 | 0.942 | 3.301 | 0.952 | 3.023 | 3.92 | 0 | 17.041 | 0.343 |
| IPW(0.25) | 4 | IPW | -0.251 | -0.251 | 0.105 | -0.002 | 1.015 | 0.000 | 0.944 | 3.258 | 0.944 | 3.258 | 0.948 | 3.152 | 3.92 | 0 | 11.083 | 0.220 |
| | 4 | WIPW | -0.251 | -0.251 | 0.131 | 0.002 | 1.017 | 0.000 | 0.941 | 3.333 | 0.947 | 3.158 | 0.946 | 3.208 | 3.92 | 0 | 17.073 | 0.352 |
| | 4 | AIPW | -0.251 | -0.250 | 0.084 | 0.009 | 1.011 | 0.001 | 0.945 | 3.225 | 0.949 | 3.118 | 0.951 | 3.048 | 3.92 | 0 | 6.973 | 0.137 |
| | 4 | WAIPW | -0.251 | -0.251 | 0.083 | 0.006 | 1.009 | 0.000 | 0.948 | 3.152 | 0.950 | 3.077 | 0.947 | 3.169 | 3.92 | 0 | 6.898 | 0.135 |
| IPW(0.5) | 4 | IPW | -0.251 | -0.251 | 0.131 | 0.003 | 1.026 | 0.000 | 0.946 | 3.202 | 0.945 | 3.235 | 0.947 | 3.174 | 3.92 | 0 | 17.121 | 0.344 |

| Randomization Method | Regime | Estimator | True Value x 10^3 | Mean Estimate x 10^3 | SD Estimates x 10^3 | Mean Normalized | SD Normalized | Mean Bias | CI Coverage | SE CI Coverage x 10^3 | LB Coverage | SE LB Coverage x 10^3 | UB Coverage x 10^3 | SE UB Coverage x 10^3 | Mean CI Length | SE CI Length x 10^3 | MSE x 10^3 | SE MSE x 10^3 |
|---|---|---|---|---|---|---|---|---|---|---|---|---|---|---|---|---|---|---|
| | 4 | WIPW | -0.251 | -0.250 | 0.170 | 0.004 | 1.026 | 0.001 | 0.941 | 3.343 | 0.948 | 3.140 | 0.946 | 3.191 | 3.92 | 0 | 29.029 | 0.581 |
| | 4 | AIPW | -0.251 | -0.251 | 0.102 | -0.001 | 1.022 | 0.000 | 0.948 | 3.140 | 0.948 | 3.129 | 0.946 | 3.202 | 3.92 | 0 | 10.405 | 0.210 |
| | 4 | WAIPW | -0.251 | -0.251 | 0.096 | -0.002 | 1.016 | 0.000 | 0.948 | 3.152 | 0.948 | 3.129 | 0.946 | 3.208 | 3.92 | 0 | 9.168 | 0.184 |
| IPW(0.75) | 4 | IPW | -0.251 | -0.251 | 0.178 | -0.001 | 1.029 | 0.000 | 0.942 | 3.301 | 0.943 | 3.285 | 0.946 | 3.186 | 3.92 | 0 | 31.558 | 0.631 |
| | 4 | WIPW | -0.251 | -0.251 | 0.234 | 0.001 | 1.052 | 0.000 | 0.939 | 3.375 | 0.940 | 3.349 | 0.944 | 3.263 | 3.92 | 0 | 54.653 | 1.108 |
| | 4 | AIPW | -0.251 | -0.250 | 0.137 | 0.015 | 1.010 | 0.001 | 0.947 | 3.175 | 0.944 | 3.241 | 0.951 | 3.065 | 3.92 | 0 | 18.647 | 0.372 |
| | 4 | WAIPW | -0.251 | -0.250 | 0.112 | 0.011 | 1.001 | 0.001 | 0.948 | 3.135 | 0.949 | 3.106 | 0.951 | 3.053 | 3.92 | 0 | 12.640 | 0.254 |
| IPW(1) | 4 | IPW | -0.251 | -0.251 | 0.265 | -0.003 | 1.134 | 0.000 | 0.916 | 3.919 | 0.926 | 3.702 | 0.931 | 3.585 | 3.92 | 0 | 69.991 | 1.387 |
| | 4 | WIPW | -0.251 | -0.252 | 0.338 | -0.006 | 1.180 | -0.001 | 0.908 | 4.096 | 0.922 | 3.788 | 0.924 | 3.748 | 3.92 | 0 | 113.967 | 2.295 |
| | 4 | AIPW | -0.251 | -0.249 | 0.202 | 0.027 | 1.053 | 0.002 | 0.936 | 3.472 | 0.935 | 3.492 | 0.945 | 3.224 | 3.92 | 0 | 40.865 | 0.823 |
| | 4 | WAIPW | -0.251 | -0.249 | 0.131 | 0.025 | 1.030 | 0.002 | 0.944 | 3.246 | 0.941 | 3.338 | 0.946 | 3.202 | 3.92 | 0 | 17.282 | 0.341 |
| SR | 4 | IPW | -0.251 | -0.250 | 0.091 | 0.014 | 1.003 | 0.001 | 0.949 | 3.106 | 0.949 | 3.106 | 0.956 | 2.913 | 3.92 | 0 | 8.211 | 0.170 |
| | 4 | WIPW | -0.251 | -0.249 | 0.109 | 0.014 | 1.014 | 0.002 | 0.944 | 3.257 | 0.948 | 3.140 | 0.948 | 3.152 | 3.92 | 0 | 11.956 | 0.244 |
| | 4 | AIPW | -0.251 | -0.250 | 0.073 | 0.014 | 1.001 | 0.001 | 0.945 | 3.219 | 0.948 | 3.140 | 0.954 | 2.957 | 3.92 | 0 | 5.374 | 0.113 |
| | 4 | WAIPW | -0.251 | -0.250 | 0.074 | 0.016 | 1.002 | 0.001 | 0.947 | 3.169 | 0.946 | 3.186 | 0.953 | 2.981 | 3.92 | 0 | 5.463 | 0.115 |
| TS(0.25) | 4 | IPW | -0.251 | -0.352 | 0.478 | -0.386 | 1.965 | -0.101 | 0.808 | 5.571 | 0.916 | 3.923 | 0.802 | 5.638 | 3.92 | 0 | 238.388 | 6.374 |
| | 4 | WIPW | -0.251 | -0.283 | 0.265 | -0.286 | 1.199 | -0.032 | 0.908 | 4.088 | 0.959 | 2.791 | 0.883 | 4.549 | 3.92 | 0 | 71.167 | 3.296 |
| | 4 | AIPW | -0.251 | -0.257 | 0.959 | 0.096 | 1.137 | -0.006 | 0.935 | 3.497 | 0.912 | 3.999 | 0.958 | 2.824 | 3.92 | 0 | 919.035 | 177.384 |
| | 4 | WAIPW | -0.251 | -0.256 | 0.640 | 0.068 | 1.122 | -0.005 | 0.935 | 3.487 | 0.920 | 3.846 | 0.954 | 2.963 | 3.92 | 0 | 409.161 | 80.343 |
| TS(0.50) | 4 | IPW | -0.251 | -0.368 | 0.491 | -0.497 | 2.140 | -0.117 | 0.770 | 5.954 | 0.912 | 4.007 | 0.768 | 5.970 | 3.92 | 0 | 254.843 | 8.687 |
| | 4 | WIPW | -0.251 | -0.282 | 0.304 | -0.358 | 1.326 | -0.031 | 0.875 | 4.681 | 0.951 | 3.053 | 0.843 | 5.145 | 3.92 | 0 | 93.495 | 4.885 |
| | 4 | AIPW | -0.251 | -0.219 | 0.921 | 0.136 | 1.149 | 0.032 | 0.925 | 3.716 | 0.906 | 4.120 | 0.961 | 2.752 | 3.92 | 0 | 849.648 | 156.636 |
| | 4 | WAIPW | -0.251 | -0.228 | 0.758 | 0.118 | 1.124 | 0.023 | 0.931 | 3.575 | 0.914 | 3.965 | 0.960 | 2.785 | 3.92 | 0 | 575.613 | 103.607 |
| TS(0.75) | 4 | IPW | -0.251 | -0.415 | 0.507 | -0.589 | 2.766 | -0.164 | 0.751 | 6.119 | 0.925 | 3.734 | 0.737 | 6.228 | 3.92 | 0 | 283.608 | 8.904 |
| | 4 | WIPW | -0.251 | -0.300 | 0.358 | -0.501 | 1.492 | -0.049 | 0.821 | 5.427 | 0.947 | 3.163 | 0.797 | 5.685 | 3.92 | 0 | 130.370 | 5.516 |
| | 4 | AIPW | -0.251 | -0.240 | 1.253 | 0.109 | 1.154 | 0.011 | 0.923 | 3.771 | 0.903 | 4.178 | 0.958 | 2.831 | 3.92 | 0 | 1570.998 | 246.240 |
| | 4 | WAIPW | -0.251 | -0.246 | 1.155 | 0.106 | 1.137 | 0.005 | 0.926 | 3.693 | 0.911 | 4.031 | 0.957 | 2.882 | 3.92 | 0 | 1334.229 | 206.275 |
| TS(1) | 4 | IPW | -0.251 | -0.439 | 0.484 | -0.699 | 3.081 | -0.188 | 0.746 | 6.155 | 0.938 | 3.411 | 0.721 | 6.345 | 3.92 | 0 | 269.661 | 7.823 |
| | 4 | WIPW | -0.251 | -0.318 | 0.373 | -0.619 | 1.590 | -0.067 | 0.804 | 5.619 | 0.951 | 3.065 | 0.772 | 5.934 | 3.92 | 0 | 143.307 | 5.700 |
| | 4 | AIPW | -0.251 | -0.246 | 1.265 | 0.140 | 1.179 | 0.005 | 0.917 | 3.893 | 0.899 | 4.269 | 0.958 | 2.824 | 3.92 | 0 | 1599.248 | 237.157 |
| | 4 | WAIPW | -0.251 | -0.242 | 1.334 | 0.144 | 1.160 | 0.009 | 0.919 | 3.855 | 0.902 | 4.209 | 0.958 | 2.824 | 3.92 | 0 | 1780.566 | 306.441 |
| WAIPW(0.25) | 4 | IPW | -0.251 | -0.250 | 0.103 | 0.005 | 0.993 | 0.001 | 0.952 | 3.024 | 0.948 | 3.146 | 0.954 | 2.957 | 3.92 | 0 | 10.551 | 0.216 |
| | 4 | WIPW | -0.251 | -0.250 | 0.101 | 0.007 | 0.993 | 0.001 | 0.952 | 3.030 | 0.949 | 3.112 | 0.953 | 2.994 | 3.92 | 0 | 10.276 | 0.210 |
| | 4 | AIPW | -0.251 | -0.250 | 0.082 | 0.015 | 1.001 | 0.001 | 0.945 | 3.219 | 0.948 | 3.135 | 0.950 | 3.094 | 3.92 | 0 | 6.800 | 0.141 |
| | 4 | WAIPW | -0.251 | -0.250 | 0.082 | 0.016 | 0.997 | 0.001 | 0.947 | 3.180 | 0.948 | 3.129 | 0.952 | 3.030 | 3.92 | 0 | 6.715 | 0.138 |
| WAIPW(0.5) | 4 | IPW | -0.251 | -0.251 | 0.128 | -0.002 | 1.003 | 0.000 | 0.947 | 3.163 | 0.947 | 3.174 | 0.950 | 3.088 | 3.92 | 0 | 16.412 | 0.329 |
| | 4 | WIPW | -0.251 | -0.251 | 0.118 | 0.001 | 0.999 | 0.000 | 0.949 | 3.117 | 0.950 | 3.094 | 0.951 | 3.059 | 3.92 | 0 | 13.924 | 0.278 |
| | 4 | AIPW | -0.251 | -0.249 | 0.100 | 0.019 | 0.997 | 0.002 | 0.953 | 2.999 | 0.947 | 3.180 | 0.953 | 2.999 | 3.92 | 0 | 9.904 | 0.193 |
| | 4 | WAIPW | -0.251 | -0.249 | 0.094 | 0.021 | 0.994 | 0.002 | 0.953 | 2.999 | 0.947 | 3.157 | 0.950 | 3.077 | 3.92 | 0 | 8.793 | 0.171 |
| WAIPW(0.75) | 4 | IPW | -0.251 | -0.253 | 0.179 | -0.008 | 1.042 | -0.002 | 0.941 | 3.328 | 0.945 | 3.230 | 0.945 | 3.230 | 3.92 | 0 | 32.125 | 0.641 |
| | 4 | WIPW | -0.251 | -0.253 | 0.143 | -0.011 | 1.019 | -0.002 | 0.944 | 3.252 | 0.948 | 3.146 | 0.944 | 3.252 | 3.92 | 0 | 20.558 | 0.411 |
| | 4 | AIPW | -0.251 | -0.251 | 0.138 | 0.001 | 1.024 | 0.000 | 0.943 | 3.269 | 0.948 | 3.146 | 0.942 | 3.296 | 3.92 | 0 | 19.128 | 0.400 |
| | 4 | WAIPW | -0.251 | -0.252 | 0.114 | -0.003 | 1.011 | -0.001 | 0.947 | 3.169 | 0.950 | 3.077 | 0.947 | 3.169 | 3.92 | 0 | 12.930 | 0.268 |
| WAIPW(1) | 4 | IPW | -0.251 | -0.252 | 0.262 | -0.004 | 1.117 | -0.001 | 0.921 | 3.819 | 0.933 | 3.536 | 0.929 | 3.623 | 3.92 | 0 | 68.875 | 1.382 |

| Randomization Method | Regime | Estimator | True Value x 10^3 | Mean Estimate x 10^3 | SD Estimates x 10^3 | Mean Normalized | SD Normalized | Mean Bias | CI Coverage | SE CI Coverage x 10^3 | LB Coverage | SE LB Coverage x 10^3 | UB Coverage x 10^3 | SE UB Coverage x 10^3 | Mean CI Length | SE CI Length x 10^3 | MSE x 10^3 | SE MSE x 10^3 |
|---|---|---|---|---|---|---|---|---|---|---|---|---|---|---|---|---|---|---|
| | 4 | WIPW | -0.251 | -0.253 | 0.162 | -0.014 | 1.026 | -0.002 | 0.944 | 3.241 | 0.945 | 3.230 | 0.944 | 3.263 | 3.92 | 0 | 26.370 | 0.549 |
| | 4 | AIPW | -0.251 | -0.252 | 0.201 | 0.013 | 1.038 | -0.001 | 0.941 | 3.333 | 0.940 | 3.359 | 0.948 | 3.146 | 3.92 | 0 | 40.539 | 0.890 |
| | 4 | WAIPW | -0.251 | -0.252 | 0.131 | -0.002 | 1.017 | -0.001 | 0.950 | 3.071 | 0.946 | 3.197 | 0.948 | 3.135 | 3.92 | 0 | 17.130 | 0.352 |
| WIPW(0.25) | 4 | IPW | -0.251 | -0.250 | 0.105 | 0.009 | 1.007 | 0.001 | 0.952 | 3.012 | 0.948 | 3.152 | 0.949 | 3.100 | 3.92 | 0 | 10.937 | 0.211 |
| | 4 | WIPW | -0.251 | -0.250 | 0.103 | 0.012 | 1.004 | 0.001 | 0.953 | 3.006 | 0.948 | 3.146 | 0.953 | 2.981 | 3.92 | 0 | 10.581 | 0.204 |
| | 4 | AIPW | -0.251 | -0.250 | 0.083 | 0.014 | 1.003 | 0.001 | 0.949 | 3.100 | 0.951 | 3.053 | 0.949 | 3.112 | 3.92 | 0 | 6.858 | 0.139 |
| | 4 | WAIPW | -0.251 | -0.250 | 0.082 | 0.015 | 0.999 | 0.001 | 0.952 | 3.018 | 0.949 | 3.112 | 0.950 | 3.077 | 3.92 | 0 | 6.756 | 0.136 |
| WIPW(0.5) | 4 | IPW | -0.251 | -0.249 | 0.128 | 0.015 | 0.997 | 0.002 | 0.953 | 2.987 | 0.947 | 3.157 | 0.951 | 3.041 | 3.92 | 0 | 16.306 | 0.326 |
| | 4 | WIPW | -0.251 | -0.249 | 0.118 | 0.013 | 0.996 | 0.002 | 0.951 | 3.065 | 0.950 | 3.094 | 0.954 | 2.975 | 3.92 | 0 | 13.918 | 0.277 |
| | 4 | AIPW | -0.251 | -0.250 | 0.101 | 0.016 | 1.007 | 0.001 | 0.949 | 3.123 | 0.950 | 3.077 | 0.948 | 3.135 | 3.92 | 0 | 10.185 | 0.204 |
| | 4 | WAIPW | -0.251 | -0.250 | 0.095 | 0.015 | 1.000 | 0.001 | 0.950 | 3.083 | 0.950 | 3.071 | 0.949 | 3.100 | 3.92 | 0 | 8.943 | 0.177 |
| WIPW(0.75) | 4 | IPW | -0.251 | -0.249 | 0.181 | 0.018 | 1.041 | 0.002 | 0.939 | 3.380 | 0.943 | 3.279 | 0.950 | 3.089 | 3.92 | 0 | 32.682 | 0.642 |
| | 4 | WIPW | -0.251 | -0.249 | 0.144 | 0.015 | 1.022 | 0.002 | 0.947 | 3.163 | 0.946 | 3.197 | 0.951 | 3.042 | 3.92 | 0 | 20.835 | 0.415 |
| | 4 | AIPW | -0.251 | -0.249 | 0.138 | 0.017 | 1.022 | 0.002 | 0.943 | 3.279 | 0.942 | 3.317 | 0.949 | 3.112 | 3.92 | 0 | 19.165 | 0.382 |
| | 4 | WAIPW | -0.251 | -0.249 | 0.115 | 0.017 | 1.019 | 0.002 | 0.943 | 3.279 | 0.944 | 3.263 | 0.953 | 3.006 | 3.92 | 0 | 13.157 | 0.262 |
| WIPW(1) | 4 | IPW | -0.251 | -0.252 | 0.265 | -0.005 | 1.116 | -0.001 | 0.923 | 3.780 | 0.936 | 3.452 | 0.934 | 3.507 | 3.92 | 0 | 70.072 | 1.406 |
| | 4 | WIPW | -0.251 | -0.253 | 0.167 | -0.010 | 1.050 | -0.002 | 0.937 | 3.447 | 0.946 | 3.191 | 0.938 | 3.421 | 3.92 | 0 | 27.848 | 0.548 |
| | 4 | AIPW | -0.251 | -0.252 | 0.201 | 0.009 | 1.036 | -0.001 | 0.941 | 3.327 | 0.945 | 3.224 | 0.947 | 3.169 | 3.92 | 0 | 40.476 | 0.820 |
| | 4 | WAIPW | -0.251 | -0.252 | 0.133 | -0.002 | 1.030 | -0.001 | 0.942 | 3.311 | 0.944 | 3.241 | 0.944 | 3.257 | 3.92 | 0 | 17.599 | 0.346 |
| AIPW(0.25) | 5 | IPW | -2.408 | -2.408 | 0.087 | -0.023 | 1.002 | 0.000 | 0.949 | 3.112 | 0.953 | 3.000 | 0.947 | 3.158 | 3.92 | 0 | 7.562 | 0.154 |
| | 5 | WIPW | -2.408 | -2.408 | 0.099 | -0.009 | 0.984 | 0.000 | 0.950 | 3.094 | 0.954 | 2.951 | 0.953 | 3.006 | 3.92 | 0 | 9.707 | 0.200 |
| | 5 | AIPW | -2.408 | -2.407 | 0.071 | -0.016 | 1.004 | 0.000 | 0.949 | 3.100 | 0.951 | 3.048 | 0.951 | 3.048 | 3.92 | 0 | 5.022 | 0.099 |
| | 5 | WAIPW | -2.408 | -2.406 | 0.071 | 0.005 | 1.005 | 0.002 | 0.951 | 3.059 | 0.949 | 3.112 | 0.953 | 2.988 | 3.92 | 0 | 5.061 | 0.100 |
| AIPW(0.5) | 5 | IPW | -2.408 | -2.406 | 0.090 | -0.022 | 0.992 | 0.002 | 0.952 | 3.029 | 0.951 | 3.041 | 0.953 | 2.993 | 3.92 | 0 | 8.065 | 0.173 |
| | 5 | WIPW | -2.408 | -2.403 | 0.105 | 0.021 | 1.008 | 0.005 | 0.947 | 3.174 | 0.944 | 3.263 | 0.952 | 3.011 | 3.92 | 0 | 11.115 | 0.246 |
| | 5 | AIPW | -2.408 | -2.406 | 0.072 | -0.014 | 0.998 | 0.002 | 0.952 | 3.029 | 0.954 | 2.975 | 0.949 | 3.106 | 3.92 | 0 | 5.247 | 0.113 |
| | 5 | WAIPW | -2.408 | -2.403 | 0.072 | 0.028 | 1.000 | 0.005 | 0.950 | 3.077 | 0.947 | 3.174 | 0.951 | 3.059 | 3.92 | 0 | 5.170 | 0.110 |
| AIPW(0.75) | 5 | IPW | -2.408 | -2.406 | 0.103 | -0.054 | 1.020 | 0.002 | 0.945 | 3.219 | 0.951 | 3.042 | 0.942 | 3.301 | 3.92 | 0 | 10.674 | 0.257 |
| | 5 | WIPW | -2.408 | -2.401 | 0.120 | 0.015 | 1.032 | 0.007 | 0.944 | 3.263 | 0.940 | 3.364 | 0.942 | 3.306 | 3.92 | 0 | 14.357 | 0.363 |
| | 5 | AIPW | -2.408 | -2.405 | 0.081 | -0.043 | 1.017 | 0.003 | 0.944 | 3.258 | 0.951 | 3.048 | 0.939 | 3.396 | 3.92 | 0 | 6.644 | 0.161 |
| | 5 | WAIPW | -2.408 | -2.400 | 0.078 | 0.028 | 1.023 | 0.007 | 0.943 | 3.290 | 0.943 | 3.285 | 0.945 | 3.236 | 3.92 | 0 | 6.103 | 0.140 |
| AIPW(1) | 5 | IPW | -2.408 | -2.406 | 0.126 | -0.083 | 1.038 | 0.002 | 0.940 | 3.359 | 0.954 | 2.969 | 0.936 | 3.457 | 3.92 | 0 | 15.755 | 0.432 |
| | 5 | WIPW | -2.408 | -2.397 | 0.138 | 0.007 | 1.034 | 0.011 | 0.947 | 3.180 | 0.946 | 3.191 | 0.943 | 3.268 | 3.92 | 0 | 19.284 | 0.590 |
| | 5 | AIPW | -2.408 | -2.407 | 0.095 | -0.094 | 1.013 | 0.000 | 0.947 | 3.157 | 0.954 | 2.975 | 0.943 | 3.290 | 3.92 | 0 | 9.067 | 0.257 |
| | 5 | WAIPW | -2.408 | -2.400 | 0.082 | 0.005 | 1.019 | 0.007 | 0.947 | 3.174 | 0.946 | 3.186 | 0.952 | 3.035 | 3.92 | 0 | 6.737 | 0.167 |
| AR-1 | 5 | IPW | -2.408 | -2.407 | 0.079 | 0.015 | 1.012 | 0.001 | 0.948 | 3.140 | 0.947 | 3.180 | 0.947 | 3.157 | 3.92 | 0 | 6.251 | 0.135 |
| | 5 | WIPW | -2.408 | -2.408 | 0.077 | -0.013 | 1.019 | -0.001 | 0.947 | 3.174 | 0.952 | 3.029 | 0.941 | 3.322 | 3.92 | 0 | 5.874 | 0.129 |
| | 5 | AIPW | -2.408 | -2.406 | 0.064 | 0.036 | 1.002 | 0.001 | 0.948 | 3.146 | 0.946 | 3.197 | 0.954 | 2.963 | 3.92 | 0 | 4.087 | 0.084 |
| | 5 | WAIPW | -2.408 | -2.407 | 0.063 | 0.016 | 1.012 | 0.000 | 0.944 | 3.263 | 0.945 | 3.213 | 0.949 | 3.100 | 3.92 | 0 | 3.976 | 0.083 |
| AR-2 | 5 | IPW | -2.408 | -2.408 | 0.075 | 0.003 | 0.993 | 0.000 | 0.949 | 3.100 | 0.953 | 2.999 | 0.950 | 3.083 | 3.92 | 0 | 5.629 | 0.116 |
| | 5 | WIPW | -2.408 | -2.408 | 0.072 | -0.006 | 0.988 | -0.001 | 0.950 | 3.088 | 0.953 | 2.993 | 0.948 | 3.146 | 3.92 | 0 | 5.162 | 0.107 |
| | 5 | AIPW | -2.408 | -2.406 | 0.062 | 0.035 | 0.991 | 0.002 | 0.952 | 3.029 | 0.945 | 3.230 | 0.956 | 2.901 | 3.92 | 0 | 3.855 | 0.077 |
| | 5 | WAIPW | -2.408 | -2.407 | 0.061 | 0.025 | 0.992 | 0.001 | 0.950 | 3.083 | 0.950 | 3.094 | 0.953 | 2.981 | 3.92 | 0 | 3.704 | 0.074 |
| IAIPW(0.25) | 5 | IPW | -2.408 | -2.408 | 0.090 | -0.029 | 1.025 | -0.001 | 0.942 | 3.301 | 0.949 | 3.106 | 0.941 | 3.328 | 3.92 | 0 | 8.018 | 0.160 |

| Randomization Method | Regime | Estimator | True Value x 10^3 | Mean Estimate x 10^3 | SD Estimates x 10^3 | Mean Normalized | SD Normalized | Mean Bias | CI Coverage | SE CI Coverage x 10^3 | LB Coverage | SE LB Coverage x 10^3 | UB Coverage x 10^3 | SE UB Coverage x 10^3 | Mean CI Length | SE CI Length x 10^3 | MSE x 10^3 | SE MSE x 10^3 |
|---|---|---|---|---|---|---|---|---|---|---|---|---|---|---|---|---|---|---|
| | 5 | WIPW | -2.408 | -2.407 | 0.089 | -0.010 | 1.024 | 0.001 | 0.944 | 3.252 | 0.947 | 3.163 | 0.944 | 3.247 | 3.92 | 0 | 7.927 | 0.158 |
| | 5 | AIPW | -2.408 | -2.409 | 0.072 | -0.034 | 1.020 | -0.001 | 0.944 | 3.241 | 0.948 | 3.141 | 0.945 | 3.214 | 3.92 | 0 | 5.230 | 0.108 |
| | 5 | WAIPW | -2.408 | -2.408 | 0.072 | -0.020 | 1.018 | 0.000 | 0.943 | 3.285 | 0.948 | 3.141 | 0.946 | 3.186 | 3.92 | 0 | 5.243 | 0.107 |
| IAIPW(0.5) | 5 | IPW | -2.408 | -2.407 | 0.091 | -0.041 | 1.006 | 0.000 | 0.948 | 3.140 | 0.952 | 3.023 | 0.946 | 3.186 | 3.92 | 0 | 8.284 | 0.176 |
| | 5 | WIPW | -2.408 | -2.403 | 0.089 | 0.005 | 1.010 | 0.004 | 0.947 | 3.157 | 0.948 | 3.140 | 0.950 | 3.094 | 3.92 | 0 | 7.938 | 0.167 |
| | 5 | AIPW | -2.408 | -2.407 | 0.073 | -0.034 | 1.010 | 0.001 | 0.945 | 3.230 | 0.951 | 3.059 | 0.948 | 3.135 | 3.92 | 0 | 5.393 | 0.112 |
| | 5 | WAIPW | -2.408 | -2.404 | 0.073 | 0.009 | 1.016 | 0.004 | 0.944 | 3.263 | 0.944 | 3.246 | 0.951 | 3.047 | 3.92 | 0 | 5.354 | 0.112 |
| IAIPW(0.75) | 5 | IPW | -2.408 | -2.407 | 0.100 | -0.062 | 1.004 | 0.001 | 0.948 | 3.135 | 0.955 | 2.945 | 0.940 | 3.349 | 3.92 | 0 | 10.069 | 0.248 |
| | 5 | WIPW | -2.408 | -2.401 | 0.093 | 0.004 | 1.004 | 0.006 | 0.953 | 2.988 | 0.949 | 3.100 | 0.950 | 3.094 | 3.92 | 0 | 8.680 | 0.204 |
| | 5 | AIPW | -2.408 | -2.408 | 0.080 | -0.075 | 1.012 | 0.000 | 0.947 | 3.175 | 0.956 | 2.914 | 0.938 | 3.421 | 3.92 | 0 | 6.368 | 0.149 |
| | 5 | WAIPW | -2.408 | -2.403 | 0.076 | -0.002 | 1.018 | 0.005 | 0.946 | 3.197 | 0.949 | 3.106 | 0.942 | 3.301 | 3.92 | 0 | 5.862 | 0.132 |
| IAIPW(1) | 5 | IPW | -2.408 | -2.407 | 0.125 | -0.086 | 1.022 | 0.001 | 0.945 | 3.235 | 0.950 | 3.071 | 0.941 | 3.333 | 3.92 | 0 | 15.614 | 0.467 |
| | 5 | WIPW | -2.408 | -2.399 | 0.101 | 0.001 | 1.010 | 0.008 | 0.948 | 3.129 | 0.950 | 3.083 | 0.951 | 3.059 | 3.92 | 0 | 10.208 | 0.253 |
| | 5 | AIPW | -2.408 | -2.407 | 0.095 | -0.093 | 1.017 | 0.000 | 0.944 | 3.257 | 0.956 | 2.888 | 0.939 | 3.375 | 3.92 | 0 | 9.036 | 0.265 |
| | 5 | WAIPW | -2.408 | -2.401 | 0.083 | 0.002 | 1.026 | 0.007 | 0.942 | 3.295 | 0.944 | 3.246 | 0.946 | 3.208 | 3.92 | 0 | 6.855 | 0.170 |
| IPW(0.25) | 5 | IPW | -2.408 | -2.408 | 0.088 | -0.031 | 1.016 | 0.000 | 0.944 | 3.241 | 0.950 | 3.094 | 0.944 | 3.247 | 3.92 | 0 | 7.678 | 0.155 |
| | 5 | WIPW | -2.408 | -2.406 | 0.100 | -0.001 | 1.015 | 0.001 | 0.946 | 3.197 | 0.951 | 3.065 | 0.946 | 3.203 | 3.92 | 0 | 10.024 | 0.203 |
| | 5 | AIPW | -2.408 | -2.408 | 0.072 | -0.014 | 1.022 | 0.000 | 0.948 | 3.135 | 0.949 | 3.100 | 0.945 | 3.214 | 3.92 | 0 | 5.134 | 0.101 |
| | 5 | WAIPW | -2.408 | -2.407 | 0.072 | 0.000 | 1.023 | 0.001 | 0.947 | 3.180 | 0.947 | 3.163 | 0.946 | 3.191 | 3.92 | 0 | 5.177 | 0.102 |
| IPW(0.5) | 5 | IPW | -2.408 | -2.407 | 0.089 | -0.051 | 0.995 | 0.001 | 0.951 | 3.059 | 0.954 | 2.957 | 0.947 | 3.180 | 3.92 | 0 | 7.887 | 0.172 |
| | 5 | WIPW | -2.408 | -2.404 | 0.102 | 0.003 | 1.003 | 0.004 | 0.949 | 3.100 | 0.951 | 3.065 | 0.949 | 3.123 | 3.92 | 0 | 10.479 | 0.230 |
| | 5 | AIPW | -2.408 | -2.408 | 0.072 | -0.032 | 1.012 | 0.000 | 0.945 | 3.230 | 0.950 | 3.071 | 0.946 | 3.202 | 3.92 | 0 | 5.231 | 0.111 |
| | 5 | WAIPW | -2.408 | -2.406 | 0.072 | -0.005 | 1.013 | 0.002 | 0.946 | 3.186 | 0.950 | 3.077 | 0.949 | 3.100 | 3.92 | 0 | 5.136 | 0.108 |
| IPW(0.75) | 5 | IPW | -2.408 | -2.406 | 0.099 | -0.090 | 0.996 | 0.001 | 0.951 | 3.053 | 0.954 | 2.951 | 0.946 | 3.203 | 3.92 | 0 | 9.796 | 0.282 |
| | 5 | WIPW | -2.408 | -2.399 | 0.112 | 0.010 | 1.009 | 0.009 | 0.950 | 3.083 | 0.942 | 3.301 | 0.952 | 3.030 | 3.92 | 0 | 12.633 | 0.374 |
| | 5 | AIPW | -2.408 | -2.406 | 0.077 | -0.029 | 0.995 | 0.002 | 0.948 | 3.135 | 0.950 | 3.089 | 0.946 | 3.186 | 3.92 | 0 | 5.856 | 0.138 |
| | 5 | WAIPW | -2.408 | -2.403 | 0.073 | 0.022 | 0.996 | 0.005 | 0.951 | 3.042 | 0.946 | 3.197 | 0.952 | 3.030 | 3.92 | 0 | 5.381 | 0.124 |
| IPW(1) | 5 | IPW | -2.408 | -2.409 | 0.116 | -0.137 | 1.015 | -0.002 | 0.945 | 3.219 | 0.958 | 2.824 | 0.935 | 3.497 | 3.92 | 0 | 13.419 | 0.399 |
| | 5 | WIPW | -2.408 | -2.399 | 0.128 | -0.006 | 1.043 | 0.009 | 0.940 | 3.369 | 0.943 | 3.284 | 0.940 | 3.364 | 3.92 | 0 | 16.560 | 0.511 |
| | 5 | AIPW | -2.408 | -2.409 | 0.090 | -0.078 | 1.012 | -0.001 | 0.943 | 3.290 | 0.954 | 2.969 | 0.942 | 3.306 | 3.92 | 0 | 8.020 | 0.227 |
| | 5 | WAIPW | -2.408 | -2.404 | 0.078 | -0.007 | 1.020 | 0.004 | 0.939 | 3.375 | 0.948 | 3.152 | 0.945 | 3.224 | 3.92 | 0 | 6.114 | 0.145 |
| SR | 5 | IPW | -2.408 | -2.408 | 0.090 | 0.002 | 1.003 | 0.000 | 0.947 | 3.169 | 0.953 | 3.005 | 0.944 | 3.241 | 3.92 | 0 | 8.157 | 0.163 |
| | 5 | WIPW | -2.408 | -2.408 | 0.109 | 0.000 | 1.012 | 0.000 | 0.945 | 3.219 | 0.947 | 3.169 | 0.949 | 3.106 | 3.92 | 0 | 11.775 | 0.237 |
| | 5 | AIPW | -2.408 | -2.407 | 0.074 | 0.008 | 1.009 | 0.001 | 0.945 | 3.224 | 0.949 | 3.100 | 0.946 | 3.197 | 3.92 | 0 | 5.424 | 0.106 |
| | 5 | WAIPW | -2.408 | -2.407 | 0.074 | 0.009 | 1.010 | 0.001 | 0.945 | 3.224 | 0.948 | 3.135 | 0.946 | 3.191 | 3.92 | 0 | 5.519 | 0.108 |
| TS(0.25) | 5 | IPW | -2.408 | -2.405 | 0.091 | -0.010 | 0.993 | 0.003 | 0.952 | 3.017 | 0.952 | 3.017 | 0.950 | 3.094 | 3.92 | 0 | 8.303 | 0.298 |
| | 5 | WIPW | -2.408 | -2.402 | 0.083 | 0.028 | 0.997 | 0.006 | 0.951 | 3.041 | 0.949 | 3.123 | 0.953 | 2.981 | 3.92 | 0 | 6.960 | 0.174 |
| | 5 | AIPW | -2.408 | -2.405 | 0.075 | 0.003 | 1.003 | 0.003 | 0.948 | 3.152 | 0.948 | 3.146 | 0.951 | 3.041 | 3.92 | 0 | 5.646 | 0.233 |
| | 5 | WAIPW | -2.408 | -2.402 | 0.070 | 0.040 | 1.014 | 0.005 | 0.946 | 3.197 | 0.940 | 3.348 | 0.951 | 3.047 | 3.92 | 0 | 4.933 | 0.128 |
| TS(0.50) | 5 | IPW | -2.408 | -2.408 | 0.105 | -0.068 | 0.995 | 0.000 | 0.956 | 2.901 | 0.957 | 2.856 | 0.946 | 3.186 | 3.92 | 0 | 11.045 | 0.388 |
| | 5 | WIPW | -2.408 | -2.400 | 0.095 | 0.002 | 0.998 | 0.007 | 0.952 | 3.035 | 0.947 | 3.163 | 0.950 | 3.094 | 3.92 | 0 | 8.992 | 0.240 |
| | 5 | AIPW | -2.408 | -2.406 | 0.083 | -0.055 | 0.995 | 0.002 | 0.950 | 3.077 | 0.956 | 2.907 | 0.947 | 3.163 | 3.92 | 0 | 6.901 | 0.251 |
| | 5 | WAIPW | -2.408 | -2.400 | 0.078 | 0.019 | 1.013 | 0.007 | 0.944 | 3.252 | 0.947 | 3.174 | 0.951 | 3.065 | 3.92 | 0 | 6.161 | 0.164 |
| TS(0.75) | 5 | IPW | -2.408 | -2.404 | 0.136 | -0.076 | 1.051 | 0.004 | 0.941 | 3.343 | 0.949 | 3.117 | 0.942 | 3.306 | 3.92 | 0 | 18.513 | 0.702 |

| Randomization Method | Regime | Estimator | True Value x 10^3 | Mean Estimate x 10^3 | SD Estimates x 10^3 | Mean Normalized | SD Normalized | Mean Bias | CI Coverage | SE CI Coverage x 10^3 | LB Coverage | SE LB Coverage x 10^3 | UB Coverage x 10^3 | SE UB Coverage x 10^3 | Mean CI Length | SE CI Length x 10^3 | MSE x 10^3 | SE MSE x 10^3 |
|---|---|---|---|---|---|---|---|---|---|---|---|---|---|---|---|---|---|---|
| | 5 | WIPW | -2.408 | -2.395 | 0.112 | 0.013 | 1.039 | 0.013 | 0.943 | 3.284 | 0.943 | 3.279 | 0.952 | 3.029 | 3.92 | 0 | 12.697 | 0.383 |
| | 5 | AIPW | -2.408 | -2.404 | 0.105 | -0.075 | 1.027 | 0.003 | 0.944 | 3.252 | 0.948 | 3.146 | 0.945 | 3.219 | 3.92 | 0 | 11.022 | 0.453 |
| | 5 | WAIPW | -2.408 | -2.396 | 0.091 | 0.027 | 1.031 | 0.012 | 0.940 | 3.354 | 0.937 | 3.436 | 0.953 | 2.981 | 3.92 | 0 | 8.349 | 0.272 |
| TS(1) | 5 | IPW | -2.408 | -2.405 | 0.165 | -0.091 | 1.057 | 0.003 | 0.937 | 3.441 | 0.944 | 3.241 | 0.935 | 3.482 | 3.92 | 0 | 27.167 | 0.940 |
| | 5 | WIPW | -2.408 | -2.394 | 0.120 | -0.003 | 1.016 | 0.013 | 0.945 | 3.224 | 0.946 | 3.191 | 0.950 | 3.077 | 3.92 | 0 | 14.670 | 0.410 |
| | 5 | AIPW | -2.408 | -2.406 | 0.125 | -0.095 | 1.035 | 0.002 | 0.941 | 3.333 | 0.947 | 3.163 | 0.941 | 3.343 | 3.92 | 0 | 15.547 | 0.568 |
| | 5 | WAIPW | -2.408 | -2.395 | 0.098 | 0.019 | 1.018 | 0.013 | 0.944 | 3.257 | 0.938 | 3.401 | 0.952 | 3.023 | 3.92 | 0 | 9.717 | 0.293 |
| WAIPW(0.25) | 5 | IPW | -2.408 | -2.408 | 0.087 | -0.015 | 1.001 | 0.000 | 0.949 | 3.112 | 0.950 | 3.089 | 0.946 | 3.197 | 3.92 | 0 | 7.600 | 0.153 |
| | 5 | WIPW | -2.408 | -2.405 | 0.087 | 0.010 | 1.003 | 0.002 | 0.948 | 3.152 | 0.947 | 3.158 | 0.947 | 3.175 | 3.92 | 0 | 7.568 | 0.153 |
| | 5 | AIPW | -2.408 | -2.408 | 0.070 | -0.017 | 0.994 | 0.000 | 0.954 | 2.957 | 0.954 | 2.969 | 0.947 | 3.163 | 3.92 | 0 | 4.914 | 0.097 |
| | 5 | WAIPW | -2.408 | -2.406 | 0.070 | 0.002 | 0.993 | 0.001 | 0.951 | 3.065 | 0.952 | 3.012 | 0.948 | 3.152 | 3.92 | 0 | 4.940 | 0.097 |
| WAIPW(0.5) | 5 | IPW | -2.408 | -2.409 | 0.089 | -0.057 | 0.995 | -0.001 | 0.950 | 3.094 | 0.957 | 2.869 | 0.945 | 3.213 | 3.92 | 0 | 7.982 | 0.175 |
| | 5 | WIPW | -2.408 | -2.405 | 0.087 | -0.016 | 0.994 | 0.002 | 0.950 | 3.077 | 0.952 | 3.035 | 0.949 | 3.123 | 3.92 | 0 | 7.583 | 0.165 |
| | 5 | AIPW | -2.408 | -2.408 | 0.071 | -0.044 | 0.983 | 0.000 | 0.952 | 3.011 | 0.953 | 2.981 | 0.950 | 3.071 | 3.92 | 0 | 5.085 | 0.109 |
| | 5 | WAIPW | -2.408 | -2.405 | 0.070 | -0.003 | 0.983 | 0.003 | 0.953 | 2.987 | 0.951 | 3.065 | 0.952 | 3.017 | 3.92 | 0 | 4.975 | 0.106 |
| WAIPW(0.75) | 5 | IPW | -2.408 | -2.405 | 0.104 | -0.052 | 1.010 | 0.002 | 0.943 | 3.290 | 0.950 | 3.094 | 0.946 | 3.191 | 3.92 | 0 | 10.728 | 0.269 |
| | 5 | WIPW | -2.408 | -2.399 | 0.095 | 0.021 | 1.009 | 0.008 | 0.945 | 3.225 | 0.945 | 3.214 | 0.952 | 3.036 | 3.92 | 0 | 9.130 | 0.216 |
| | 5 | AIPW | -2.408 | -2.407 | 0.081 | -0.070 | 1.005 | 0.000 | 0.949 | 3.112 | 0.954 | 2.963 | 0.946 | 3.208 | 3.92 | 0 | 6.562 | 0.164 |
| | 5 | WAIPW | -2.408 | -2.402 | 0.077 | 0.004 | 1.013 | 0.006 | 0.950 | 3.083 | 0.945 | 3.219 | 0.951 | 3.048 | 3.92 | 0 | 6.035 | 0.144 |
| WAIPW(1) | 5 | IPW | -2.408 | -2.409 | 0.127 | -0.102 | 1.026 | -0.001 | 0.941 | 3.343 | 0.949 | 3.123 | 0.935 | 3.487 | 3.92 | 0 | 16.098 | 0.475 |
| | 5 | WIPW | -2.408 | -2.400 | 0.103 | -0.011 | 1.015 | 0.008 | 0.945 | 3.235 | 0.950 | 3.088 | 0.942 | 3.295 | 3.92 | 0 | 10.590 | 0.270 |
| | 5 | AIPW | -2.408 | -2.408 | 0.096 | -0.101 | 1.002 | -0.001 | 0.945 | 3.213 | 0.954 | 2.975 | 0.942 | 3.306 | 3.92 | 0 | 9.219 | 0.264 |
| | 5 | WAIPW | -2.408 | -2.401 | 0.082 | -0.001 | 1.006 | 0.007 | 0.946 | 3.208 | 0.945 | 3.235 | 0.952 | 3.011 | 3.92 | 0 | 6.848 | 0.171 |
| WIPW(0.25) | 5 | IPW | -2.408 | -2.408 | 0.088 | -0.037 | 1.014 | -0.001 | 0.947 | 3.163 | 0.953 | 3.006 | 0.944 | 3.241 | 3.92 | 0 | 7.663 | 0.153 |
| | 5 | WIPW | -2.408 | -2.406 | 0.087 | -0.007 | 1.016 | 0.002 | 0.946 | 3.208 | 0.950 | 3.077 | 0.945 | 3.230 | 3.92 | 0 | 7.625 | 0.153 |
| | 5 | AIPW | -2.408 | -2.408 | 0.071 | -0.013 | 1.017 | 0.000 | 0.947 | 3.180 | 0.949 | 3.100 | 0.947 | 3.169 | 3.92 | 0 | 5.060 | 0.100 |
| | 5 | WAIPW | -2.408 | -2.406 | 0.071 | 0.003 | 1.019 | 0.001 | 0.943 | 3.269 | 0.947 | 3.180 | 0.948 | 3.146 | 3.92 | 0 | 5.109 | 0.102 |
| WIPW(0.5) | 5 | IPW | -2.408 | -2.407 | 0.089 | -0.052 | 0.997 | 0.001 | 0.954 | 2.975 | 0.956 | 2.907 | 0.950 | 3.088 | 3.92 | 0 | 7.868 | 0.181 |
| | 5 | WIPW | -2.408 | -2.402 | 0.087 | 0.008 | 1.002 | 0.006 | 0.952 | 3.023 | 0.948 | 3.146 | 0.952 | 3.023 | 3.92 | 0 | 7.597 | 0.173 |
| | 5 | AIPW | -2.408 | -2.407 | 0.071 | -0.024 | 0.997 | 0.000 | 0.950 | 3.083 | 0.955 | 2.926 | 0.952 | 3.023 | 3.92 | 0 | 5.053 | 0.107 |
| | 5 | WAIPW | -2.408 | -2.405 | 0.071 | 0.009 | 1.002 | 0.003 | 0.948 | 3.140 | 0.950 | 3.094 | 0.954 | 2.951 | 3.92 | 0 | 5.001 | 0.106 |
| WIPW(0.75) | 5 | IPW | -2.408 | -2.406 | 0.097 | -0.083 | 0.988 | 0.002 | 0.951 | 3.048 | 0.961 | 2.738 | 0.946 | 3.186 | 3.92 | 0 | 9.446 | 0.250 |
| | 5 | WIPW | -2.408 | -2.397 | 0.091 | 0.015 | 0.999 | 0.010 | 0.948 | 3.141 | 0.948 | 3.129 | 0.950 | 3.083 | 3.92 | 0 | 8.407 | 0.210 |
| | 5 | AIPW | -2.408 | -2.407 | 0.077 | -0.041 | 0.996 | 0.001 | 0.948 | 3.135 | 0.956 | 2.895 | 0.945 | 3.214 | 3.92 | 0 | 5.862 | 0.139 |
| | 5 | WAIPW | -2.408 | -2.403 | 0.074 | 0.013 | 1.008 | 0.005 | 0.948 | 3.146 | 0.951 | 3.065 | 0.949 | 3.118 | 3.92 | 0 | 5.489 | 0.125 |
| WIPW(1) | 5 | IPW | -2.408 | -2.406 | 0.117 | -0.108 | 1.012 | 0.002 | 0.945 | 3.219 | 0.955 | 2.926 | 0.940 | 3.348 | 3.92 | 0 | 13.750 | 0.418 |
| | 5 | WIPW | -2.408 | -2.395 | 0.099 | 0.011 | 1.009 | 0.012 | 0.947 | 3.174 | 0.949 | 3.117 | 0.950 | 3.088 | 3.92 | 0 | 9.883 | 0.263 |
| | 5 | AIPW | -2.408 | -2.406 | 0.090 | -0.046 | 1.012 | 0.002 | 0.949 | 3.117 | 0.952 | 3.029 | 0.942 | 3.306 | 3.92 | 0 | 8.142 | 0.231 |
| | 5 | WAIPW | -2.408 | -2.401 | 0.079 | 0.018 | 1.017 | 0.006 | 0.945 | 3.213 | 0.948 | 3.146 | 0.946 | 3.186 | 3.92 | 0 | 6.321 | 0.150 |
| AIPW(0.25) | 6 | IPW | -2.401 | -2.401 | 0.088 | -0.020 | 1.007 | 0.000 | 0.951 | 3.059 | 0.952 | 3.012 | 0.948 | 3.141 | 3.92 | 0 | 7.796 | 0.157 |
| | 6 | WIPW | -2.401 | -2.401 | 0.100 | -0.010 | 0.989 | 0.000 | 0.951 | 3.065 | 0.954 | 2.969 | 0.950 | 3.077 | 3.92 | 0 | 10.014 | 0.205 |
| | 6 | AIPW | -2.401 | -2.400 | 0.072 | -0.013 | 1.010 | 0.001 | 0.950 | 3.089 | 0.949 | 3.123 | 0.953 | 3.006 | 3.92 | 0 | 5.121 | 0.103 |
| | 6 | WAIPW | -2.401 | -2.399 | 0.072 | 0.003 | 1.009 | 0.002 | 0.949 | 3.123 | 0.946 | 3.186 | 0.954 | 2.963 | 3.92 | 0 | 5.186 | 0.105 |
| AIPW(0.5) | 6 | IPW | -2.401 | -2.398 | 0.091 | -0.009 | 0.997 | 0.003 | 0.955 | 2.938 | 0.950 | 3.077 | 0.952 | 3.035 | 3.92 | 0 | 8.321 | 0.175 |

| Randomization Method | Regime | Estimator | True Value x 10^3 | Mean Estimate x 10^3 | SD Estimates x 10^3 | Mean Normalized | SD Normalized | Mean Bias | CI Coverage | SE CI Coverage x 10^3 | LB Coverage | SE LB Coverage x 10^3 | UB Coverage x 10^3 | SE UB Coverage x 10^3 | Mean CI Length | SE CI Length x 10^3 | MSE x 10^3 | SE MSE x 10^3 |
|---|---|---|---|---|---|---|---|---|---|---|---|---|---|---|---|---|---|---|
| | 6 | WIPW | -2.401 | -2.395 | 0.108 | 0.032 | 1.018 | 0.006 | 0.943 | 3.274 | 0.942 | 3.306 | 0.950 | 3.094 | 3.92 | 0 | 11.597 | 0.253 |
| | 6 | AIPW | -2.401 | -2.398 | 0.073 | -0.004 | 1.000 | 0.003 | 0.952 | 3.011 | 0.950 | 3.077 | 0.950 | 3.071 | 3.92 | 0 | 5.319 | 0.115 |
| | 6 | WAIPW | -2.401 | -2.396 | 0.072 | 0.027 | 1.002 | 0.005 | 0.951 | 3.053 | 0.945 | 3.230 | 0.952 | 3.029 | 3.92 | 0 | 5.278 | 0.113 |
| AIPW(0.75) | 6 | IPW | -2.401 | -2.399 | 0.105 | -0.053 | 1.019 | 0.002 | 0.949 | 3.106 | 0.952 | 3.012 | 0.941 | 3.333 | 3.92 | 0 | 10.999 | 0.265 |
| | 6 | WIPW | -2.401 | -2.394 | 0.122 | 0.013 | 1.034 | 0.007 | 0.941 | 3.322 | 0.943 | 3.274 | 0.945 | 3.219 | 3.92 | 0 | 14.828 | 0.380 |
| | 6 | AIPW | -2.401 | -2.398 | 0.082 | -0.040 | 1.018 | 0.003 | 0.942 | 3.301 | 0.948 | 3.141 | 0.940 | 3.370 | 3.92 | 0 | 6.757 | 0.164 |
| | 6 | WAIPW | -2.401 | -2.394 | 0.078 | 0.022 | 1.020 | 0.007 | 0.944 | 3.258 | 0.942 | 3.296 | 0.945 | 3.236 | 3.92 | 0 | 6.205 | 0.142 |
| AIPW(1) | 6 | IPW | -2.401 | -2.398 | 0.128 | -0.074 | 1.038 | 0.003 | 0.943 | 3.290 | 0.952 | 3.029 | 0.937 | 3.447 | 3.92 | 0 | 16.377 | 0.465 |
| | 6 | WIPW | -2.401 | -2.390 | 0.142 | -0.002 | 1.043 | 0.011 | 0.942 | 3.295 | 0.947 | 3.174 | 0.941 | 3.333 | 3.92 | 0 | 20.403 | 0.640 |
| | 6 | AIPW | -2.401 | -2.400 | 0.096 | -0.082 | 1.020 | 0.001 | 0.946 | 3.208 | 0.950 | 3.094 | 0.941 | 3.338 | 3.92 | 0 | 9.302 | 0.262 |
| | 6 | WAIPW | -2.401 | -2.394 | 0.083 | 0.006 | 1.023 | 0.007 | 0.947 | 3.180 | 0.943 | 3.274 | 0.948 | 3.146 | 3.92 | 0 | 7.007 | 0.172 |
| AR-1 | 6 | IPW | -2.401 | -2.399 | 0.079 | 0.049 | 1.018 | 0.002 | 0.946 | 3.191 | 0.943 | 3.284 | 0.951 | 3.047 | 3.92 | 0 | 6.218 | 0.134 |
| | 6 | WIPW | -2.401 | -2.400 | 0.077 | 0.025 | 1.027 | 0.001 | 0.942 | 3.295 | 0.944 | 3.246 | 0.946 | 3.197 | 3.92 | 0 | 5.874 | 0.128 |
| | 6 | AIPW | -2.401 | -2.400 | 0.065 | 0.031 | 1.024 | 0.001 | 0.938 | 3.406 | 0.945 | 3.235 | 0.948 | 3.152 | 3.92 | 0 | 4.217 | 0.089 |
| | 6 | WAIPW | -2.401 | -2.401 | 0.065 | 0.022 | 1.030 | 0.000 | 0.938 | 3.421 | 0.943 | 3.284 | 0.945 | 3.213 | 3.92 | 0 | 4.173 | 0.089 |
| AR-2 | 6 | IPW | -2.401 | -2.399 | 0.075 | 0.036 | 0.995 | 0.002 | 0.950 | 3.088 | 0.947 | 3.163 | 0.951 | 3.065 | 3.92 | 0 | 5.627 | 0.114 |
| | 6 | WIPW | -2.401 | -2.400 | 0.072 | 0.026 | 0.995 | 0.001 | 0.948 | 3.140 | 0.947 | 3.157 | 0.951 | 3.041 | 3.92 | 0 | 5.201 | 0.106 |
| | 6 | AIPW | -2.401 | -2.399 | 0.062 | 0.039 | 0.994 | 0.002 | 0.952 | 3.029 | 0.946 | 3.186 | 0.953 | 2.987 | 3.92 | 0 | 3.886 | 0.077 |
| | 6 | WAIPW | -2.401 | -2.399 | 0.062 | 0.033 | 0.994 | 0.002 | 0.951 | 3.059 | 0.949 | 3.117 | 0.953 | 3.005 | 3.92 | 0 | 3.790 | 0.078 |
| IAIPW(0.25) | 6 | IPW | -2.401 | -2.401 | 0.090 | -0.027 | 1.025 | 0.000 | 0.940 | 3.354 | 0.950 | 3.071 | 0.943 | 3.285 | 3.92 | 0 | 8.123 | 0.168 |
| | 6 | WIPW | -2.401 | -2.400 | 0.090 | -0.009 | 1.024 | 0.001 | 0.941 | 3.343 | 0.948 | 3.146 | 0.945 | 3.236 | 3.92 | 0 | 8.041 | 0.166 |
| | 6 | AIPW | -2.401 | -2.402 | 0.072 | -0.029 | 1.018 | -0.001 | 0.943 | 3.274 | 0.949 | 3.118 | 0.944 | 3.241 | 3.92 | 0 | 5.244 | 0.107 |
| | 6 | WAIPW | -2.401 | -2.401 | 0.073 | -0.019 | 1.016 | 0.000 | 0.947 | 3.158 | 0.947 | 3.169 | 0.947 | 3.169 | 3.92 | 0 | 5.288 | 0.108 |
| IAIPW(0.5) | 6 | IPW | -2.401 | -2.400 | 0.093 | -0.029 | 1.012 | 0.001 | 0.943 | 3.290 | 0.951 | 3.053 | 0.945 | 3.224 | 3.92 | 0 | 8.560 | 0.185 |
| | 6 | WIPW | -2.401 | -2.396 | 0.090 | 0.016 | 1.016 | 0.005 | 0.943 | 3.268 | 0.945 | 3.224 | 0.950 | 3.094 | 3.92 | 0 | 8.214 | 0.177 |
| | 6 | AIPW | -2.401 | -2.400 | 0.074 | -0.026 | 1.015 | 0.001 | 0.944 | 3.241 | 0.947 | 3.169 | 0.943 | 3.268 | 3.92 | 0 | 5.502 | 0.116 |
| | 6 | WAIPW | -2.401 | -2.397 | 0.074 | 0.008 | 1.023 | 0.004 | 0.941 | 3.338 | 0.944 | 3.263 | 0.945 | 3.230 | 3.92 | 0 | 5.497 | 0.116 |
| IAIPW(0.75) | 6 | IPW | -2.401 | -2.401 | 0.101 | -0.067 | 1.001 | 0.000 | 0.952 | 3.024 | 0.958 | 2.850 | 0.943 | 3.279 | 3.92 | 0 | 10.204 | 0.250 |
| | 6 | WIPW | -2.401 | -2.395 | 0.094 | 0.000 | 1.006 | 0.006 | 0.951 | 3.053 | 0.953 | 2.981 | 0.949 | 3.112 | 3.92 | 0 | 8.919 | 0.208 |
| | 6 | AIPW | -2.401 | -2.401 | 0.080 | -0.069 | 1.006 | 0.000 | 0.950 | 3.089 | 0.956 | 2.907 | 0.943 | 3.290 | 3.92 | 0 | 6.385 | 0.149 |
| | 6 | WAIPW | -2.401 | -2.397 | 0.077 | -0.007 | 1.013 | 0.004 | 0.946 | 3.191 | 0.948 | 3.135 | 0.948 | 3.152 | 3.92 | 0 | 5.979 | 0.136 |
| IAIPW(1) | 6 | IPW | -2.401 | -2.399 | 0.128 | -0.079 | 1.033 | 0.002 | 0.945 | 3.235 | 0.949 | 3.100 | 0.937 | 3.436 | 3.92 | 0 | 16.281 | 0.479 |
| | 6 | WIPW | -2.401 | -2.391 | 0.103 | 0.011 | 1.014 | 0.010 | 0.947 | 3.174 | 0.948 | 3.140 | 0.950 | 3.094 | 3.92 | 0 | 10.664 | 0.269 |
| | 6 | AIPW | -2.401 | -2.400 | 0.096 | -0.076 | 1.018 | 0.001 | 0.945 | 3.230 | 0.952 | 3.011 | 0.942 | 3.311 | 3.92 | 0 | 9.213 | 0.271 |
| | 6 | WAIPW | -2.401 | -2.394 | 0.084 | 0.015 | 1.025 | 0.007 | 0.943 | 3.290 | 0.942 | 3.306 | 0.949 | 3.106 | 3.92 | 0 | 7.070 | 0.175 |
| IPW(0.25) | 6 | IPW | -2.401 | -2.402 | 0.088 | -0.037 | 1.010 | -0.001 | 0.948 | 3.146 | 0.952 | 3.012 | 0.942 | 3.306 | 3.92 | 0 | 7.676 | 0.153 |
| | 6 | WIPW | -2.401 | -2.400 | 0.101 | -0.006 | 1.011 | 0.001 | 0.946 | 3.203 | 0.952 | 3.018 | 0.948 | 3.152 | 3.92 | 0 | 10.100 | 0.204 |
| | 6 | AIPW | -2.401 | -2.401 | 0.072 | -0.013 | 1.017 | 0.000 | 0.947 | 3.169 | 0.951 | 3.053 | 0.949 | 3.112 | 3.92 | 0 | 5.113 | 0.101 |
| | 6 | WAIPW | -2.401 | -2.400 | 0.072 | -0.006 | 1.017 | 0.001 | 0.950 | 3.094 | 0.950 | 3.077 | 0.950 | 3.089 | 3.92 | 0 | 5.171 | 0.102 |
| IPW(0.5) | 6 | IPW | -2.401 | -2.400 | 0.091 | -0.044 | 1.012 | 0.001 | 0.948 | 3.152 | 0.952 | 3.029 | 0.943 | 3.279 | 3.92 | 0 | 8.222 | 0.184 |
| | 6 | WIPW | -2.401 | -2.396 | 0.104 | 0.012 | 1.015 | 0.005 | 0.944 | 3.257 | 0.949 | 3.106 | 0.944 | 3.252 | 3.92 | 0 | 10.885 | 0.244 |
| | 6 | AIPW | -2.401 | -2.400 | 0.072 | -0.020 | 1.011 | 0.001 | 0.945 | 3.213 | 0.950 | 3.083 | 0.944 | 3.263 | 3.92 | 0 | 5.237 | 0.111 |
| | 6 | WAIPW | -2.401 | -2.399 | 0.072 | 0.002 | 1.012 | 0.002 | 0.946 | 3.208 | 0.946 | 3.191 | 0.947 | 3.180 | 3.92 | 0 | 5.182 | 0.110 |
| IPW(0.75) | 6 | IPW | -2.401 | -2.399 | 0.100 | -0.077 | 1.004 | 0.003 | 0.946 | 3.203 | 0.954 | 2.969 | 0.945 | 3.225 | 3.92 | 0 | 10.068 | 0.287 |

| Randomization Method | Regime | Estimator | True Value x 10^3 | Mean Estimate x 10^3 | SD Estimates x 10^3 | Mean Normalized | SD Normalized | Mean Bias | CI Coverage | SE CI Coverage x 10^3 | LB Coverage | SE LB Coverage x 10^3 | UB Coverage x 10^3 | SE UB Coverage x 10^3 | Mean CI Length | SE CI Length x 10^3 | MSE x 10^3 | SE MSE x 10^3 |
|---|---|---|---|---|---|---|---|---|---|---|---|---|---|---|---|---|---|---|
| | 6 | WIPW | -2.401 | -2.392 | 0.114 | 0.007 | 1.014 | 0.009 | 0.947 | 3.163 | 0.946 | 3.191 | 0.948 | 3.141 | 3.92 | 0 | 13.023 | 0.382 |
| | 6 | AIPW | -2.401 | -2.399 | 0.077 | -0.023 | 0.993 | 0.002 | 0.954 | 2.951 | 0.951 | 3.042 | 0.946 | 3.191 | 3.92 | 0 | 5.868 | 0.135 |
| | 6 | WAIPW | -2.401 | -2.396 | 0.074 | 0.022 | 0.998 | 0.005 | 0.955 | 2.926 | 0.948 | 3.141 | 0.952 | 3.030 | 3.92 | 0 | 5.475 | 0.122 |
| IPW(1) | 6 | IPW | -2.401 | -2.402 | 0.117 | -0.133 | 1.021 | -0.001 | 0.946 | 3.202 | 0.960 | 2.758 | 0.935 | 3.497 | 3.92 | 0 | 13.590 | 0.399 |
| | 6 | WIPW | -2.401 | -2.391 | 0.130 | -0.004 | 1.047 | 0.010 | 0.938 | 3.416 | 0.946 | 3.197 | 0.943 | 3.290 | 3.92 | 0 | 17.069 | 0.525 |
| | 6 | AIPW | -2.401 | -2.402 | 0.090 | -0.074 | 1.014 | -0.001 | 0.948 | 3.140 | 0.953 | 3.005 | 0.940 | 3.359 | 3.92 | 0 | 8.079 | 0.227 |
| | 6 | WAIPW | -2.401 | -2.398 | 0.079 | -0.010 | 1.021 | 0.004 | 0.944 | 3.263 | 0.947 | 3.163 | 0.945 | 3.224 | 3.92 | 0 | 6.185 | 0.147 |
| SR | 6 | IPW | -2.401 | -2.400 | 0.092 | 0.008 | 1.012 | 0.001 | 0.948 | 3.135 | 0.949 | 3.117 | 0.947 | 3.157 | 3.92 | 0 | 8.378 | 0.167 |
| | 6 | WIPW | -2.401 | -2.401 | 0.109 | 0.004 | 1.016 | 0.000 | 0.942 | 3.301 | 0.946 | 3.202 | 0.948 | 3.152 | 3.92 | 0 | 11.955 | 0.240 |
| | 6 | AIPW | -2.401 | -2.400 | 0.074 | 0.011 | 1.011 | 0.001 | 0.945 | 3.235 | 0.943 | 3.268 | 0.950 | 3.088 | 3.92 | 0 | 5.476 | 0.108 |
| | 6 | WAIPW | -2.401 | -2.401 | 0.075 | 0.005 | 1.012 | 0.000 | 0.944 | 3.257 | 0.947 | 3.163 | 0.948 | 3.140 | 3.92 | 0 | 5.619 | 0.111 |
| TS(0.25) | 6 | IPW | -2.401 | -2.396 | 0.093 | 0.009 | 1.001 | 0.005 | 0.951 | 3.047 | 0.951 | 3.041 | 0.953 | 2.993 | 3.92 | 0 | 8.748 | 0.317 |
| | 6 | WIPW | -2.401 | -2.393 | 0.086 | 0.047 | 1.005 | 0.008 | 0.952 | 3.029 | 0.947 | 3.174 | 0.955 | 2.920 | 3.92 | 0 | 7.463 | 0.215 |
| | 6 | AIPW | -2.401 | -2.398 | 0.076 | 0.012 | 0.995 | 0.003 | 0.951 | 3.065 | 0.950 | 3.088 | 0.953 | 2.987 | 3.92 | 0 | 5.747 | 0.242 |
| | 6 | WAIPW | -2.401 | -2.395 | 0.072 | 0.052 | 1.007 | 0.006 | 0.950 | 3.094 | 0.941 | 3.338 | 0.954 | 2.969 | 3.92 | 0 | 5.249 | 0.224 |
| TS(0.50) | 6 | IPW | -2.401 | -2.399 | 0.107 | -0.053 | 0.999 | 0.002 | 0.951 | 3.047 | 0.952 | 3.035 | 0.950 | 3.077 | 3.92 | 0 | 11.500 | 0.402 |
| | 6 | WIPW | -2.401 | -2.391 | 0.097 | 0.023 | 1.005 | 0.010 | 0.949 | 3.112 | 0.945 | 3.230 | 0.958 | 2.843 | 3.92 | 0 | 9.557 | 0.265 |
| | 6 | AIPW | -2.401 | -2.398 | 0.084 | -0.043 | 0.994 | 0.003 | 0.949 | 3.123 | 0.952 | 3.011 | 0.949 | 3.117 | 3.92 | 0 | 7.036 | 0.254 |
| | 6 | WAIPW | -2.401 | -2.392 | 0.079 | 0.033 | 1.011 | 0.009 | 0.943 | 3.268 | 0.945 | 3.224 | 0.952 | 3.035 | 3.92 | 0 | 6.374 | 0.171 |
| TS(0.75) | 6 | IPW | -2.401 | -2.395 | 0.139 | -0.071 | 1.054 | 0.006 | 0.938 | 3.421 | 0.951 | 3.065 | 0.936 | 3.462 | 3.92 | 0 | 19.399 | 0.721 |
| | 6 | WIPW | -2.401 | -2.386 | 0.115 | 0.025 | 1.045 | 0.015 | 0.939 | 3.375 | 0.939 | 3.390 | 0.945 | 3.224 | 3.92 | 0 | 13.477 | 0.407 |
| | 6 | AIPW | -2.401 | -2.396 | 0.106 | -0.055 | 1.030 | 0.005 | 0.942 | 3.295 | 0.947 | 3.174 | 0.948 | 3.152 | 3.92 | 0 | 11.348 | 0.457 |
| | 6 | WAIPW | -2.401 | -2.387 | 0.093 | 0.048 | 1.040 | 0.014 | 0.940 | 3.364 | 0.933 | 3.546 | 0.953 | 2.981 | 3.92 | 0 | 8.822 | 0.286 |
| TS(1) | 6 | IPW | -2.401 | -2.396 | 0.169 | -0.090 | 1.060 | 0.005 | 0.935 | 3.492 | 0.945 | 3.230 | 0.937 | 3.447 | 3.92 | 0 | 28.576 | 0.992 |
| | 6 | WIPW | -2.401 | -2.384 | 0.125 | 0.009 | 1.027 | 0.017 | 0.942 | 3.306 | 0.945 | 3.224 | 0.946 | 3.186 | 3.92 | 0 | 15.891 | 0.455 |
| | 6 | AIPW | -2.401 | -2.397 | 0.127 | -0.069 | 1.043 | 0.004 | 0.941 | 3.327 | 0.947 | 3.157 | 0.944 | 3.257 | 3.92 | 0 | 16.018 | 0.572 |
| | 6 | WAIPW | -2.401 | -2.387 | 0.101 | 0.044 | 1.027 | 0.014 | 0.943 | 3.274 | 0.939 | 3.375 | 0.953 | 2.999 | 3.92 | 0 | 10.383 | 0.307 |
| WAIPW(0.25) | 6 | IPW | -2.401 | -2.400 | 0.088 | -0.004 | 1.001 | 0.001 | 0.948 | 3.135 | 0.951 | 3.042 | 0.948 | 3.146 | 3.92 | 0 | 7.733 | 0.158 |
| | 6 | WIPW | -2.401 | -2.398 | 0.088 | 0.021 | 1.002 | 0.003 | 0.947 | 3.158 | 0.949 | 3.112 | 0.948 | 3.146 | 3.92 | 0 | 7.694 | 0.158 |
| | 6 | AIPW | -2.401 | -2.400 | 0.070 | -0.010 | 0.987 | 0.001 | 0.953 | 3.000 | 0.956 | 2.914 | 0.951 | 3.048 | 3.92 | 0 | 4.880 | 0.096 |
| | 6 | WAIPW | -2.401 | -2.399 | 0.070 | 0.005 | 0.987 | 0.002 | 0.951 | 3.053 | 0.954 | 2.975 | 0.950 | 3.083 | 3.92 | 0 | 4.953 | 0.098 |
| WAIPW(0.5) | 6 | IPW | -2.401 | -2.402 | 0.090 | -0.047 | 0.992 | -0.001 | 0.949 | 3.117 | 0.956 | 2.901 | 0.948 | 3.140 | 3.92 | 0 | 8.144 | 0.181 |
| | 6 | WIPW | -2.401 | -2.398 | 0.088 | -0.004 | 0.991 | 0.003 | 0.949 | 3.106 | 0.951 | 3.041 | 0.952 | 3.017 | 3.92 | 0 | 7.766 | 0.172 |
| | 6 | AIPW | -2.401 | -2.400 | 0.072 | -0.032 | 0.984 | 0.001 | 0.949 | 3.100 | 0.952 | 3.035 | 0.953 | 2.981 | 3.92 | 0 | 5.151 | 0.113 |
| | 6 | WAIPW | -2.401 | -2.398 | 0.071 | 0.003 | 0.982 | 0.003 | 0.949 | 3.117 | 0.949 | 3.112 | 0.955 | 2.926 | 3.92 | 0 | 5.068 | 0.110 |
| WAIPW(0.75) | 6 | IPW | -2.401 | -2.398 | 0.106 | -0.051 | 1.024 | 0.003 | 0.947 | 3.175 | 0.951 | 3.053 | 0.944 | 3.263 | 3.92 | 0 | 11.202 | 0.273 |
| | 6 | WIPW | -2.401 | -2.392 | 0.097 | 0.020 | 1.019 | 0.009 | 0.949 | 3.112 | 0.944 | 3.263 | 0.952 | 3.030 | 3.92 | 0 | 9.509 | 0.220 |
| | 6 | AIPW | -2.401 | -2.400 | 0.081 | -0.061 | 1.006 | 0.001 | 0.946 | 3.186 | 0.954 | 2.963 | 0.949 | 3.106 | 3.92 | 0 | 6.602 | 0.164 |
| | 6 | WAIPW | -2.401 | -2.395 | 0.078 | 0.003 | 1.011 | 0.006 | 0.947 | 3.158 | 0.950 | 3.094 | 0.955 | 2.926 | 3.92 | 0 | 6.100 | 0.144 |
| WAIPW(1) | 6 | IPW | -2.401 | -2.402 | 0.128 | -0.096 | 1.020 | -0.001 | 0.942 | 3.311 | 0.952 | 3.023 | 0.942 | 3.301 | 3.92 | 0 | 16.282 | 0.481 |
| | 6 | WIPW | -2.401 | -2.394 | 0.102 | -0.008 | 1.000 | 0.007 | 0.948 | 3.146 | 0.951 | 3.047 | 0.949 | 3.123 | 3.92 | 0 | 10.455 | 0.261 |
| | 6 | AIPW | -2.401 | -2.402 | 0.096 | -0.095 | 1.002 | -0.001 | 0.947 | 3.174 | 0.951 | 3.059 | 0.944 | 3.252 | 3.92 | 0 | 9.247 | 0.263 |
| | 6 | WAIPW | -2.401 | -2.395 | 0.083 | -0.007 | 1.001 | 0.006 | 0.949 | 3.123 | 0.946 | 3.202 | 0.952 | 3.023 | 3.92 | 0 | 6.868 | 0.166 |
| WIPW(0.25) | 6 | IPW | -2.401 | -2.401 | 0.087 | -0.032 | 0.997 | 0.000 | 0.952 | 3.018 | 0.954 | 2.975 | 0.949 | 3.112 | 3.92 | 0 | 7.496 | 0.150 |

| Randomization Method | Regime | Estimator | True Value x 10^3 | Mean Estimate x 10^3 | SD Estimates x 10^3 | Mean Normalized | SD Normalized | Mean Bias | CI Coverage | SE CI Coverage x 10^3 | LB Coverage | SE LB Coverage x 10^3 | UB Coverage x 10^3 | SE UB Coverage x 10^3 | Mean CI Length | SE CI Length x 10^3 | MSE x 10^3 | SE MSE x 10^3 |
|---|---|---|---|---|---|---|---|---|---|---|---|---|---|---|---|---|---|---|
| | 6 | WIPW | -2.401 | -2.399 | 0.086 | -0.004 | 0.999 | 0.002 | 0.951 | 3.059 | 0.950 | 3.077 | 0.950 | 3.083 | 3.92 | 0 | 7.456 | 0.150 |
| | 6 | AIPW | -2.401 | -2.400 | 0.071 | -0.003 | 1.011 | 0.001 | 0.948 | 3.141 | 0.952 | 3.024 | 0.948 | 3.129 | 3.92 | 0 | 5.043 | 0.100 |
| | 6 | WAIPW | -2.401 | -2.400 | 0.071 | 0.006 | 1.012 | 0.001 | 0.946 | 3.186 | 0.949 | 3.123 | 0.947 | 3.180 | 3.92 | 0 | 5.105 | 0.101 |
| WIPW(0.5) | 6 | IPW | -2.401 | -2.399 | 0.088 | -0.038 | 0.986 | 0.002 | 0.953 | 2.993 | 0.956 | 2.894 | 0.949 | 3.112 | 3.92 | 0 | 7.804 | 0.181 |
| | 6 | WIPW | -2.401 | -2.394 | 0.087 | 0.022 | 0.991 | 0.007 | 0.951 | 3.059 | 0.949 | 3.112 | 0.954 | 2.957 | 3.92 | 0 | 7.548 | 0.173 |
| | 6 | AIPW | -2.401 | -2.400 | 0.071 | -0.010 | 0.995 | 0.001 | 0.953 | 2.999 | 0.952 | 3.035 | 0.952 | 3.035 | 3.92 | 0 | 5.068 | 0.109 |
| | 6 | WAIPW | -2.401 | -2.398 | 0.071 | 0.014 | 1.003 | 0.003 | 0.949 | 3.106 | 0.949 | 3.112 | 0.952 | 3.011 | 3.92 | 0 | 5.073 | 0.109 |
| WIPW(0.75) | 6 | IPW | -2.401 | -2.400 | 0.097 | -0.094 | 0.985 | 0.001 | 0.950 | 3.094 | 0.959 | 2.798 | 0.944 | 3.258 | 3.92 | 0 | 9.485 | 0.250 |
| | 6 | WIPW | -2.401 | -2.392 | 0.091 | 0.001 | 0.993 | 0.009 | 0.947 | 3.158 | 0.952 | 3.012 | 0.952 | 3.030 | 3.92 | 0 | 8.431 | 0.211 |
| | 6 | AIPW | -2.401 | -2.400 | 0.077 | -0.042 | 0.995 | 0.001 | 0.952 | 3.036 | 0.956 | 2.895 | 0.946 | 3.197 | 3.92 | 0 | 5.909 | 0.141 |
| | 6 | WAIPW | -2.401 | -2.397 | 0.075 | 0.003 | 1.008 | 0.004 | 0.949 | 3.106 | 0.946 | 3.191 | 0.951 | 3.065 | 3.92 | 0 | 5.576 | 0.129 |
| WIPW(1) | 6 | IPW | -2.401 | -2.400 | 0.118 | -0.119 | 1.020 | 0.001 | 0.944 | 3.241 | 0.954 | 2.975 | 0.938 | 3.421 | 3.92 | 0 | 14.000 | 0.407 |
| | 6 | WIPW | -2.401 | -2.389 | 0.100 | 0.002 | 1.013 | 0.012 | 0.947 | 3.163 | 0.945 | 3.230 | 0.948 | 3.129 | 3.92 | 0 | 10.057 | 0.256 |
| | 6 | AIPW | -2.401 | -2.399 | 0.091 | -0.043 | 1.017 | 0.002 | 0.947 | 3.174 | 0.951 | 3.059 | 0.943 | 3.279 | 3.92 | 0 | 8.242 | 0.231 |
| | 6 | WAIPW | -2.401 | -2.395 | 0.080 | 0.014 | 1.020 | 0.006 | 0.946 | 3.186 | 0.944 | 3.257 | 0.948 | 3.135 | 3.92 | 0 | 6.429 | 0.153 |
| AIPW(0.25) | 7 | IPW | -2.494 | -2.492 | 0.081 | 0.009 | 0.991 | 0.002 | 0.953 | 2.988 | 0.953 | 2.981 | 0.955 | 2.926 | 3.92 | 0 | 6.601 | 0.135 |
| | 7 | WIPW | -2.494 | -2.492 | 0.091 | 0.012 | 1.001 | 0.002 | 0.949 | 3.112 | 0.948 | 3.135 | 0.955 | 2.932 | 3.92 | 0 | 8.220 | 0.166 |
| | 7 | AIPW | -2.494 | -2.492 | 0.067 | 0.021 | 0.993 | 0.002 | 0.955 | 2.945 | 0.951 | 3.059 | 0.951 | 3.065 | 3.92 | 0 | 4.484 | 0.092 |
| | 7 | WAIPW | -2.494 | -2.492 | 0.067 | 0.023 | 0.996 | 0.002 | 0.954 | 2.975 | 0.950 | 3.071 | 0.949 | 3.100 | 3.92 | 0 | 4.520 | 0.093 |
| AIPW(0.5) | 7 | IPW | -2.494 | -2.492 | 0.079 | -0.003 | 0.996 | 0.002 | 0.951 | 3.053 | 0.954 | 2.951 | 0.947 | 3.174 | 3.92 | 0 | 6.217 | 0.129 |
| | 7 | WIPW | -2.494 | -2.491 | 0.085 | 0.018 | 1.013 | 0.003 | 0.947 | 3.180 | 0.948 | 3.146 | 0.947 | 3.169 | 3.92 | 0 | 7.163 | 0.149 |
| | 7 | AIPW | -2.494 | -2.493 | 0.066 | 0.000 | 1.008 | 0.001 | 0.946 | 3.191 | 0.944 | 3.246 | 0.949 | 3.106 | 3.92 | 0 | 4.315 | 0.091 |
| | 7 | WAIPW | -2.494 | -2.492 | 0.065 | 0.012 | 1.013 | 0.002 | 0.944 | 3.241 | 0.941 | 3.322 | 0.952 | 3.029 | 3.92 | 0 | 4.259 | 0.091 |
| AIPW(0.75) | 7 | IPW | -2.494 | -2.492 | 0.079 | -0.010 | 1.003 | 0.002 | 0.952 | 3.030 | 0.949 | 3.100 | 0.950 | 3.071 | 3.92 | 0 | 6.311 | 0.145 |
| | 7 | WIPW | -2.494 | -2.489 | 0.083 | 0.020 | 1.014 | 0.005 | 0.942 | 3.301 | 0.952 | 3.018 | 0.945 | 3.219 | 3.92 | 0 | 6.871 | 0.177 |
| | 7 | AIPW | -2.494 | -2.492 | 0.066 | 0.006 | 1.011 | 0.002 | 0.945 | 3.214 | 0.947 | 3.175 | 0.951 | 3.065 | 3.92 | 0 | 4.319 | 0.094 |
| | 7 | WAIPW | -2.494 | -2.490 | 0.065 | 0.031 | 1.018 | 0.004 | 0.943 | 3.279 | 0.941 | 3.338 | 0.950 | 3.083 | 3.92 | 0 | 4.193 | 0.094 |
| AIPW(1) | 7 | IPW | -2.494 | -2.493 | 0.086 | -0.047 | 1.011 | 0.001 | 0.945 | 3.224 | 0.959 | 2.798 | 0.940 | 3.369 | 3.92 | 0 | 7.354 | 0.237 |
| | 7 | WIPW | -2.494 | -2.492 | 0.091 | -0.043 | 1.055 | 0.002 | 0.936 | 3.467 | 0.953 | 2.993 | 0.932 | 3.561 | 3.92 | 0 | 8.301 | 0.325 |
| | 7 | AIPW | -2.494 | -2.493 | 0.068 | -0.018 | 1.010 | 0.001 | 0.948 | 3.135 | 0.954 | 2.969 | 0.945 | 3.219 | 3.92 | 0 | 4.667 | 0.131 |
| | 7 | WAIPW | -2.494 | -2.492 | 0.067 | 0.006 | 1.023 | 0.002 | 0.946 | 3.191 | 0.949 | 3.100 | 0.948 | 3.129 | 3.92 | 0 | 4.478 | 0.114 |
| AR-1 | 7 | IPW | -2.494 | -2.490 | 0.091 | 0.070 | 1.018 | 0.004 | 0.946 | 3.208 | 0.939 | 3.375 | 0.951 | 3.041 | 3.92 | 0 | 8.336 | 0.173 |
| | 7 | WIPW | -2.494 | -2.493 | 0.088 | 0.035 | 1.022 | 0.001 | 0.946 | 3.208 | 0.941 | 3.327 | 0.949 | 3.117 | 3.92 | 0 | 7.734 | 0.159 |
| | 7 | AIPW | -2.494 | -2.493 | 0.072 | 0.048 | 1.016 | 0.001 | 0.944 | 3.241 | 0.945 | 3.224 | 0.951 | 3.065 | 3.92 | 0 | 5.248 | 0.106 |
| | 7 | WAIPW | -2.494 | -2.494 | 0.071 | 0.023 | 1.013 | 0.000 | 0.947 | 3.169 | 0.947 | 3.169 | 0.950 | 3.088 | 3.92 | 0 | 5.107 | 0.102 |
| AR-2 | 7 | IPW | -2.494 | -2.493 | 0.082 | 0.033 | 1.014 | 0.001 | 0.946 | 3.191 | 0.944 | 3.263 | 0.952 | 3.011 | 3.92 | 0 | 6.765 | 0.145 |
| | 7 | WIPW | -2.494 | -2.494 | 0.080 | 0.020 | 1.016 | 0.000 | 0.947 | 3.169 | 0.943 | 3.279 | 0.951 | 3.047 | 3.92 | 0 | 6.392 | 0.136 |
| | 7 | AIPW | -2.494 | -2.494 | 0.067 | 0.017 | 1.009 | 0.000 | 0.950 | 3.094 | 0.950 | 3.083 | 0.950 | 3.077 | 3.92 | 0 | 4.459 | 0.089 |
| | 7 | WAIPW | -2.494 | -2.494 | 0.067 | 0.005 | 1.012 | 0.000 | 0.949 | 3.123 | 0.951 | 3.047 | 0.948 | 3.135 | 3.92 | 0 | 4.430 | 0.091 |
| IAIPW(0.25) | 7 | IPW | -2.494 | -2.494 | 0.080 | -0.014 | 0.973 | 0.000 | 0.956 | 2.901 | 0.956 | 2.914 | 0.949 | 3.112 | 3.92 | 0 | 6.398 | 0.127 |
| | 7 | WIPW | -2.494 | -2.493 | 0.079 | 0.001 | 0.976 | 0.001 | 0.954 | 2.951 | 0.954 | 2.957 | 0.949 | 3.106 | 3.92 | 0 | 6.306 | 0.126 |
| | 7 | AIPW | -2.494 | -2.494 | 0.067 | -0.007 | 0.989 | 0.000 | 0.953 | 3.006 | 0.955 | 2.938 | 0.949 | 3.123 | 3.92 | 0 | 4.437 | 0.089 |
| | 7 | WAIPW | -2.494 | -2.493 | 0.067 | 0.002 | 0.992 | 0.001 | 0.952 | 3.024 | 0.952 | 3.030 | 0.951 | 3.048 | 3.92 | 0 | 4.484 | 0.091 |
| IAIPW(0.5) | 7 | IPW | -2.494 | -2.492 | 0.080 | 0.001 | 1.008 | 0.002 | 0.950 | 3.083 | 0.951 | 3.065 | 0.949 | 3.100 | 3.92 | 0 | 6.382 | 0.134 |

| Randomization Method | Regime | Estimator | True Value x 10^3 | Mean Estimate x 10^3 | SD Estimates x 10^3 | Mean Normalized | SD Normalized | Mean Bias | CI Coverage | SE CI Coverage x 10^3 | LB Coverage | SE LB Coverage x 10^3 | UB Coverage x 10^3 | SE UB Coverage x 10^3 | Mean CI Length | SE CI Length x 10^3 | MSE x 10^3 | SE MSE x 10^3 |
|---|---|---|---|---|---|---|---|---|---|---|---|---|---|---|---|---|---|---|
| | 7 | WIPW | -2.494 | -2.490 | 0.077 | 0.022 | 1.007 | 0.004 | 0.950 | 3.077 | 0.946 | 3.202 | 0.952 | 3.017 | 3.92 | 0 | 5.979 | 0.128 |
| | 7 | AIPW | -2.494 | -2.492 | 0.067 | 0.009 | 1.017 | 0.002 | 0.948 | 3.152 | 0.945 | 3.213 | 0.951 | 3.047 | 3.92 | 0 | 4.434 | 0.091 |
| | 7 | WAIPW | -2.494 | -2.491 | 0.066 | 0.019 | 1.023 | 0.003 | 0.946 | 3.186 | 0.942 | 3.306 | 0.949 | 3.106 | 3.92 | 0 | 4.381 | 0.091 |
| IAIPW(0.75) | 7 | IPW | -2.494 | -2.494 | 0.079 | -0.040 | 1.002 | 0.000 | 0.952 | 3.030 | 0.954 | 2.969 | 0.948 | 3.135 | 3.92 | 0 | 6.192 | 0.134 |
| | 7 | WIPW | -2.494 | -2.491 | 0.075 | -0.008 | 1.010 | 0.003 | 0.948 | 3.129 | 0.950 | 3.077 | 0.948 | 3.141 | 3.92 | 0 | 5.702 | 0.125 |
| | 7 | AIPW | -2.494 | -2.493 | 0.065 | -0.015 | 1.001 | 0.001 | 0.947 | 3.169 | 0.953 | 2.994 | 0.948 | 3.135 | 3.92 | 0 | 4.175 | 0.091 |
| | 7 | WAIPW | -2.494 | -2.492 | 0.064 | 0.003 | 1.010 | 0.002 | 0.946 | 3.197 | 0.946 | 3.186 | 0.948 | 3.146 | 3.92 | 0 | 4.087 | 0.089 |
| IAIPW(1) | 7 | IPW | -2.494 | -2.492 | 0.085 | -0.035 | 1.014 | 0.002 | 0.950 | 3.071 | 0.951 | 3.047 | 0.944 | 3.241 | 3.92 | 0 | 7.237 | 0.175 |
| | 7 | WIPW | -2.494 | -2.488 | 0.078 | 0.017 | 1.012 | 0.006 | 0.950 | 3.094 | 0.949 | 3.123 | 0.948 | 3.146 | 3.92 | 0 | 6.126 | 0.141 |
| | 7 | AIPW | -2.494 | -2.491 | 0.067 | 0.011 | 1.000 | 0.003 | 0.950 | 3.088 | 0.950 | 3.088 | 0.952 | 3.023 | 3.92 | 0 | 4.498 | 0.103 |
| | 7 | WAIPW | -2.494 | -2.489 | 0.066 | 0.043 | 1.016 | 0.005 | 0.947 | 3.157 | 0.944 | 3.257 | 0.957 | 2.856 | 3.92 | 0 | 4.376 | 0.102 |
| IPW(0.25) | 7 | IPW | -2.494 | -2.492 | 0.084 | -0.002 | 1.007 | 0.002 | 0.951 | 3.048 | 0.946 | 3.197 | 0.951 | 3.065 | 3.92 | 0 | 7.015 | 0.147 |
| | 7 | WIPW | -2.494 | -2.490 | 0.093 | 0.024 | 1.006 | 0.004 | 0.948 | 3.129 | 0.944 | 3.258 | 0.952 | 3.018 | 3.92 | 0 | 8.595 | 0.183 |
| | 7 | AIPW | -2.494 | -2.492 | 0.069 | 0.013 | 1.017 | 0.002 | 0.947 | 3.169 | 0.948 | 3.129 | 0.947 | 3.175 | 3.92 | 0 | 4.726 | 0.094 |
| | 7 | WAIPW | -2.494 | -2.492 | 0.069 | 0.013 | 1.018 | 0.002 | 0.947 | 3.163 | 0.945 | 3.219 | 0.950 | 3.083 | 3.92 | 0 | 4.763 | 0.095 |
| IPW(0.5) | 7 | IPW | -2.494 | -2.494 | 0.082 | -0.046 | 1.015 | 0.000 | 0.947 | 3.163 | 0.950 | 3.077 | 0.942 | 3.295 | 3.92 | 0 | 6.658 | 0.143 |
| | 7 | WIPW | -2.494 | -2.491 | 0.088 | 0.007 | 1.025 | 0.003 | 0.950 | 3.088 | 0.947 | 3.169 | 0.948 | 3.152 | 3.92 | 0 | 7.764 | 0.170 |
| | 7 | AIPW | -2.494 | -2.494 | 0.067 | -0.022 | 1.019 | 0.000 | 0.948 | 3.135 | 0.951 | 3.065 | 0.946 | 3.186 | 3.92 | 0 | 4.468 | 0.095 |
| | 7 | WAIPW | -2.494 | -2.493 | 0.066 | -0.005 | 1.024 | 0.001 | 0.949 | 3.117 | 0.948 | 3.129 | 0.948 | 3.129 | 3.92 | 0 | 4.412 | 0.094 |
| IPW(0.75) | 7 | IPW | -2.494 | -2.491 | 0.081 | -0.019 | 0.989 | 0.003 | 0.950 | 3.094 | 0.956 | 2.901 | 0.953 | 2.994 | 3.92 | 0 | 6.597 | 0.166 |
| | 7 | WIPW | -2.494 | -2.488 | 0.085 | 0.026 | 1.005 | 0.006 | 0.954 | 2.975 | 0.949 | 3.100 | 0.950 | 3.077 | 3.92 | 0 | 7.230 | 0.182 |
| | 7 | AIPW | -2.494 | -2.492 | 0.066 | 0.006 | 1.012 | 0.002 | 0.950 | 3.089 | 0.945 | 3.219 | 0.951 | 3.059 | 3.92 | 0 | 4.382 | 0.097 |
| | 7 | WAIPW | -2.494 | -2.491 | 0.065 | 0.027 | 1.025 | 0.003 | 0.945 | 3.225 | 0.943 | 3.274 | 0.952 | 3.024 | 3.92 | 0 | 4.292 | 0.095 |
| IPW(1) | 7 | IPW | -2.494 | -2.492 | 0.086 | -0.064 | 1.001 | 0.002 | 0.948 | 3.140 | 0.957 | 2.875 | 0.939 | 3.385 | 3.92 | 0 | 7.476 | 0.207 |
| | 7 | WIPW | -2.494 | -2.487 | 0.091 | 0.005 | 1.011 | 0.007 | 0.948 | 3.152 | 0.951 | 3.041 | 0.946 | 3.186 | 3.92 | 0 | 8.239 | 0.254 |
| | 7 | AIPW | -2.494 | -2.493 | 0.068 | -0.014 | 1.010 | 0.001 | 0.948 | 3.140 | 0.950 | 3.094 | 0.946 | 3.202 | 3.92 | 0 | 4.693 | 0.107 |
| | 7 | WAIPW | -2.494 | -2.490 | 0.066 | 0.019 | 1.022 | 0.004 | 0.942 | 3.317 | 0.941 | 3.322 | 0.948 | 3.152 | 3.92 | 0 | 4.430 | 0.100 |
| SR | 7 | IPW | -2.494 | -2.492 | 0.090 | 0.024 | 0.994 | 0.002 | 0.950 | 3.071 | 0.948 | 3.129 | 0.953 | 2.981 | 3.92 | 0 | 8.191 | 0.165 |
| | 7 | WIPW | -2.494 | -2.490 | 0.110 | 0.030 | 1.017 | 0.004 | 0.943 | 3.279 | 0.941 | 3.333 | 0.950 | 3.094 | 3.92 | 0 | 12.138 | 0.245 |
| | 7 | AIPW | -2.494 | -2.492 | 0.074 | 0.027 | 1.011 | 0.002 | 0.947 | 3.157 | 0.942 | 3.306 | 0.949 | 3.106 | 3.92 | 0 | 5.493 | 0.114 |
| | 7 | WAIPW | -2.494 | -2.492 | 0.075 | 0.022 | 1.012 | 0.002 | 0.946 | 3.186 | 0.945 | 3.235 | 0.952 | 3.029 | 3.92 | 0 | 5.627 | 0.117 |
| TS(0.25) | 7 | IPW | -2.494 | -2.490 | 0.077 | 0.024 | 1.003 | 0.004 | 0.949 | 3.112 | 0.948 | 3.140 | 0.950 | 3.094 | 3.92 | 0 | 5.970 | 0.156 |
| | 7 | WIPW | -2.494 | -2.489 | 0.073 | 0.044 | 1.008 | 0.005 | 0.946 | 3.186 | 0.946 | 3.208 | 0.951 | 3.041 | 3.92 | 0 | 5.339 | 0.118 |
| | 7 | AIPW | -2.494 | -2.491 | 0.064 | 0.029 | 1.002 | 0.003 | 0.946 | 3.208 | 0.947 | 3.157 | 0.952 | 3.023 | 3.92 | 0 | 4.069 | 0.110 |
| | 7 | WAIPW | -2.494 | -2.490 | 0.062 | 0.044 | 1.007 | 0.004 | 0.943 | 3.268 | 0.946 | 3.208 | 0.950 | 3.071 | 3.92 | 0 | 3.880 | 0.099 |
| TS(0.50) | 7 | IPW | -2.494 | -2.491 | 0.077 | 0.008 | 0.990 | 0.003 | 0.949 | 3.100 | 0.950 | 3.083 | 0.949 | 3.112 | 3.92 | 0 | 5.914 | 0.177 |
| | 7 | WIPW | -2.494 | -2.488 | 0.072 | 0.036 | 0.995 | 0.006 | 0.950 | 3.071 | 0.949 | 3.100 | 0.948 | 3.135 | 3.92 | 0 | 5.275 | 0.122 |
| | 7 | AIPW | -2.494 | -2.491 | 0.064 | 0.020 | 1.005 | 0.003 | 0.953 | 2.999 | 0.946 | 3.208 | 0.948 | 3.146 | 3.92 | 0 | 4.047 | 0.086 |
| | 7 | WAIPW | -2.494 | -2.490 | 0.063 | 0.038 | 1.013 | 0.004 | 0.947 | 3.157 | 0.940 | 3.359 | 0.951 | 3.059 | 3.92 | 0 | 3.951 | 0.089 |
| TS(0.75) | 7 | IPW | -2.494 | -2.491 | 0.081 | -0.019 | 0.994 | 0.003 | 0.951 | 3.047 | 0.954 | 2.957 | 0.946 | 3.191 | 3.92 | 0 | 6.541 | 0.167 |
| | 7 | WIPW | -2.494 | -2.487 | 0.076 | 0.035 | 0.995 | 0.007 | 0.953 | 2.999 | 0.947 | 3.174 | 0.953 | 3.005 | 3.92 | 0 | 5.819 | 0.149 |
| | 7 | AIPW | -2.494 | -2.490 | 0.066 | 0.031 | 0.998 | 0.004 | 0.954 | 2.951 | 0.948 | 3.152 | 0.954 | 2.963 | 3.92 | 0 | 4.404 | 0.114 |
| | 7 | WAIPW | -2.494 | -2.488 | 0.066 | 0.063 | 1.009 | 0.006 | 0.949 | 3.117 | 0.942 | 3.317 | 0.958 | 2.837 | 3.92 | 0 | 4.339 | 0.118 |
| TS(1) | 7 | IPW | -2.494 | -2.492 | 0.089 | -0.065 | 1.007 | 0.002 | 0.946 | 3.191 | 0.956 | 2.888 | 0.939 | 3.395 | 3.92 | 0 | 7.886 | 0.241 |

| Randomization Method | Regime | Estimator | True Value x 10^3 | Mean Estimate x 10^3 | SD Estimates x 10^3 | Mean Normalized | SD Normalized | Mean Bias | CI Coverage | SE CI Coverage x 10^3 | LB Coverage | SE LB Coverage x 10^3 | UB Coverage x 10^3 | SE UB Coverage x 10^3 | Mean CI Length | SE CI Length x 10^3 | MSE x 10^3 | SE MSE x 10^3 |
|---|---|---|---|---|---|---|---|---|---|---|---|---|---|---|---|---|---|---|
| | 7 | WIPW | -2.494 | -2.487 | 0.081 | 0.003 | 1.000 | 0.007 | 0.948 | 3.140 | 0.956 | 2.913 | 0.947 | 3.174 | 3.92 | 0 | 6.551 | 0.185 |
| | 7 | AIPW | -2.494 | -2.492 | 0.070 | -0.003 | 0.993 | 0.002 | 0.955 | 2.932 | 0.954 | 2.951 | 0.953 | 3.005 | 3.92 | 0 | 4.882 | 0.148 |
| | 7 | WAIPW | -2.494 | -2.490 | 0.068 | 0.035 | 0.997 | 0.004 | 0.956 | 2.913 | 0.949 | 3.117 | 0.957 | 2.869 | 3.92 | 0 | 4.601 | 0.163 |
| WAIPW(0.25) | 7 | IPW | -2.494 | -2.492 | 0.083 | 0.005 | 1.006 | 0.002 | 0.949 | 3.118 | 0.947 | 3.175 | 0.948 | 3.141 | 3.92 | 0 | 6.821 | 0.143 |
| | 7 | WIPW | -2.494 | -2.491 | 0.082 | 0.017 | 1.006 | 0.003 | 0.949 | 3.123 | 0.944 | 3.241 | 0.949 | 3.106 | 3.92 | 0 | 6.696 | 0.141 |
| | 7 | AIPW | -2.494 | -2.493 | 0.068 | 0.004 | 1.006 | 0.001 | 0.947 | 3.163 | 0.948 | 3.135 | 0.952 | 3.030 | 3.92 | 0 | 4.595 | 0.093 |
| | 7 | WAIPW | -2.494 | -2.492 | 0.068 | 0.009 | 1.010 | 0.002 | 0.947 | 3.175 | 0.947 | 3.158 | 0.953 | 2.988 | 3.92 | 0 | 4.642 | 0.094 |
| WAIPW(0.5) | 7 | IPW | -2.494 | -2.491 | 0.078 | 0.012 | 0.983 | 0.003 | 0.954 | 2.969 | 0.954 | 2.975 | 0.954 | 2.969 | 3.92 | 0 | 6.074 | 0.127 |
| | 7 | WIPW | -2.494 | -2.489 | 0.076 | 0.035 | 0.991 | 0.005 | 0.951 | 3.053 | 0.950 | 3.088 | 0.954 | 2.963 | 3.92 | 0 | 5.803 | 0.123 |
| | 7 | AIPW | -2.494 | -2.491 | 0.064 | 0.019 | 0.986 | 0.003 | 0.952 | 3.029 | 0.950 | 3.083 | 0.954 | 2.957 | 3.92 | 0 | 4.130 | 0.085 |
| | 7 | WAIPW | -2.494 | -2.491 | 0.064 | 0.029 | 0.996 | 0.003 | 0.951 | 3.059 | 0.948 | 3.146 | 0.954 | 2.951 | 3.92 | 0 | 4.119 | 0.085 |
| WAIPW(0.75) | 7 | IPW | -2.494 | -2.494 | 0.078 | -0.038 | 0.993 | 0.000 | 0.954 | 2.975 | 0.956 | 2.888 | 0.947 | 3.163 | 3.92 | 0 | 6.159 | 0.137 |
| | 7 | WIPW | -2.494 | -2.490 | 0.075 | 0.000 | 1.002 | 0.004 | 0.949 | 3.100 | 0.950 | 3.077 | 0.950 | 3.083 | 3.92 | 0 | 5.662 | 0.132 |
| | 7 | AIPW | -2.494 | -2.493 | 0.065 | -0.019 | 1.009 | 0.001 | 0.950 | 3.094 | 0.954 | 2.963 | 0.948 | 3.152 | 3.92 | 0 | 4.281 | 0.096 |
| | 7 | WAIPW | -2.494 | -2.492 | 0.065 | 0.002 | 1.023 | 0.002 | 0.945 | 3.219 | 0.951 | 3.065 | 0.947 | 3.163 | 3.92 | 0 | 4.202 | 0.096 |
| WAIPW(1) | 7 | IPW | -2.494 | -2.492 | 0.084 | -0.042 | 1.009 | 0.002 | 0.946 | 3.208 | 0.954 | 2.963 | 0.944 | 3.252 | 3.92 | 0 | 7.083 | 0.188 |
| | 7 | WIPW | -2.494 | -2.489 | 0.078 | 0.004 | 1.011 | 0.005 | 0.946 | 3.186 | 0.948 | 3.129 | 0.951 | 3.065 | 3.92 | 0 | 6.098 | 0.156 |
| | 7 | AIPW | -2.494 | -2.493 | 0.067 | -0.029 | 0.999 | 0.001 | 0.950 | 3.094 | 0.950 | 3.071 | 0.950 | 3.094 | 3.92 | 0 | 4.534 | 0.114 |
| | 7 | WAIPW | -2.494 | -2.492 | 0.066 | -0.003 | 1.010 | 0.002 | 0.948 | 3.152 | 0.945 | 3.219 | 0.953 | 2.993 | 3.92 | 0 | 4.348 | 0.109 |
| WIPW(0.25) | 7 | IPW | -2.494 | -2.493 | 0.084 | -0.006 | 1.008 | 0.001 | 0.946 | 3.208 | 0.951 | 3.059 | 0.947 | 3.169 | 3.92 | 0 | 6.998 | 0.144 |
| | 7 | WIPW | -2.494 | -2.491 | 0.083 | 0.014 | 1.014 | 0.003 | 0.944 | 3.252 | 0.947 | 3.169 | 0.946 | 3.208 | 3.92 | 0 | 6.961 | 0.144 |
| | 7 | AIPW | -2.494 | -2.493 | 0.069 | 0.009 | 1.013 | 0.001 | 0.942 | 3.317 | 0.947 | 3.163 | 0.946 | 3.208 | 3.92 | 0 | 4.719 | 0.097 |
| | 7 | WAIPW | -2.494 | -2.492 | 0.069 | 0.012 | 1.016 | 0.002 | 0.941 | 3.322 | 0.947 | 3.180 | 0.945 | 3.230 | 3.92 | 0 | 4.753 | 0.097 |
| WIPW(0.5) | 7 | IPW | -2.494 | -2.494 | 0.079 | -0.040 | 0.986 | 0.000 | 0.952 | 3.023 | 0.954 | 2.951 | 0.950 | 3.077 | 3.92 | 0 | 6.316 | 0.139 |
| | 7 | WIPW | -2.494 | -2.490 | 0.078 | -0.002 | 0.998 | 0.004 | 0.947 | 3.163 | 0.949 | 3.100 | 0.950 | 3.077 | 3.92 | 0 | 6.113 | 0.136 |
| | 7 | AIPW | -2.494 | -2.494 | 0.065 | -0.014 | 0.997 | 0.000 | 0.951 | 3.047 | 0.956 | 2.913 | 0.949 | 3.123 | 3.92 | 0 | 4.267 | 0.086 |
| | 7 | WAIPW | -2.494 | -2.493 | 0.065 | -0.007 | 1.004 | 0.001 | 0.951 | 3.053 | 0.954 | 2.969 | 0.949 | 3.123 | 3.92 | 0 | 4.217 | 0.086 |
| WIPW(0.75) | 7 | IPW | -2.494 | -2.495 | 0.080 | -0.077 | 0.980 | -0.001 | 0.956 | 2.901 | 0.960 | 2.772 | 0.948 | 3.135 | 3.92 | 0 | 6.421 | 0.168 |
| | 7 | WIPW | -2.494 | -2.489 | 0.077 | -0.015 | 0.996 | 0.005 | 0.951 | 3.053 | 0.951 | 3.048 | 0.953 | 2.994 | 3.92 | 0 | 5.991 | 0.157 |
| | 7 | AIPW | -2.494 | -2.495 | 0.064 | -0.036 | 0.977 | -0.001 | 0.953 | 2.994 | 0.955 | 2.932 | 0.952 | 3.024 | 3.92 | 0 | 4.105 | 0.089 |
| | 7 | WAIPW | -2.494 | -2.493 | 0.063 | -0.011 | 0.989 | 0.001 | 0.951 | 3.059 | 0.952 | 3.036 | 0.953 | 3.006 | 3.92 | 0 | 4.012 | 0.090 |
| WIPW(1) | 7 | IPW | -2.494 | -2.494 | 0.086 | -0.077 | 0.995 | 0.000 | 0.953 | 2.993 | 0.960 | 2.772 | 0.943 | 3.279 | 3.92 | 0 | 7.451 | 0.233 |
| | 7 | WIPW | -2.494 | -2.487 | 0.081 | 0.006 | 1.012 | 0.007 | 0.948 | 3.140 | 0.947 | 3.174 | 0.950 | 3.083 | 3.92 | 0 | 6.568 | 0.186 |
| | 7 | AIPW | -2.494 | -2.493 | 0.068 | -0.018 | 0.996 | 0.001 | 0.952 | 3.035 | 0.956 | 2.901 | 0.952 | 3.029 | 3.92 | 0 | 4.591 | 0.119 |
| | 7 | WAIPW | -2.494 | -2.491 | 0.065 | 0.011 | 1.005 | 0.003 | 0.951 | 3.059 | 0.949 | 3.112 | 0.954 | 2.969 | 3.92 | 0 | 4.206 | 0.100 |
| AIPW(0.25) | 8 | IPW | -2.501 | -2.499 | 0.081 | 0.004 | 0.995 | 0.001 | 0.951 | 3.048 | 0.952 | 3.030 | 0.953 | 2.988 | 3.92 | 0 | 6.570 | 0.138 |
| | 8 | WIPW | -2.501 | -2.499 | 0.090 | 0.010 | 1.005 | 0.002 | 0.949 | 3.112 | 0.952 | 3.036 | 0.950 | 3.089 | 3.92 | 0 | 8.168 | 0.170 |
| | 8 | AIPW | -2.501 | -2.499 | 0.067 | 0.016 | 0.992 | 0.002 | 0.955 | 2.932 | 0.950 | 3.071 | 0.953 | 2.994 | 3.92 | 0 | 4.442 | 0.093 |
| | 8 | WAIPW | -2.501 | -2.498 | 0.067 | 0.024 | 0.997 | 0.002 | 0.953 | 3.006 | 0.949 | 3.100 | 0.953 | 3.006 | 3.92 | 0 | 4.466 | 0.094 |
| AIPW(0.5) | 8 | IPW | -2.501 | -2.500 | 0.079 | -0.017 | 1.006 | 0.001 | 0.950 | 3.083 | 0.953 | 2.981 | 0.947 | 3.174 | 3.92 | 0 | 6.177 | 0.130 |
| | 8 | WIPW | -2.501 | -2.498 | 0.083 | 0.006 | 1.010 | 0.002 | 0.949 | 3.117 | 0.947 | 3.180 | 0.952 | 3.017 | 3.92 | 0 | 6.914 | 0.146 |
| | 8 | AIPW | -2.501 | -2.500 | 0.065 | -0.010 | 1.010 | 0.001 | 0.946 | 3.197 | 0.951 | 3.059 | 0.945 | 3.213 | 3.92 | 0 | 4.287 | 0.090 |
| | 8 | WAIPW | -2.501 | -2.498 | 0.065 | 0.010 | 1.017 | 0.002 | 0.943 | 3.279 | 0.946 | 3.186 | 0.948 | 3.152 | 3.92 | 0 | 4.202 | 0.090 |
| AIPW(0.75) | 8 | IPW | -2.501 | -2.499 | 0.078 | -0.012 | 1.013 | 0.002 | 0.947 | 3.158 | 0.946 | 3.191 | 0.950 | 3.083 | 3.92 | 0 | 6.156 | 0.140 |

| Randomization Method | Regime | Estimator | True Value x 10^3 | Mean Estimate x 10^3 | SD Estimates x 10^3 | Mean Normalized | SD Normalized | Mean Bias | CI Coverage | SE CI Coverage x 10^3 | LB Coverage | SE LB Coverage x 10^3 | UB Coverage x 10^3 | SE UB Coverage x 10^3 | Mean CI Length | SE CI Length x 10^3 | MSE x 10^3 | SE MSE x 10^3 |
|---|---|---|---|---|---|---|---|---|---|---|---|---|---|---|---|---|---|---|
| | 8 | WIPW | -2.501 | -2.496 | 0.080 | 0.024 | 1.010 | 0.004 | 0.946 | 3.203 | 0.946 | 3.186 | 0.948 | 3.141 | 3.92 | 0 | 6.354 | 0.156 |
| | 8 | AIPW | -2.501 | -2.499 | 0.065 | 0.001 | 1.013 | 0.002 | 0.946 | 3.203 | 0.950 | 3.083 | 0.947 | 3.175 | 3.92 | 0 | 4.250 | 0.092 |
| | 8 | WAIPW | -2.501 | -2.496 | 0.064 | 0.034 | 1.022 | 0.004 | 0.944 | 3.252 | 0.944 | 3.241 | 0.947 | 3.163 | 3.92 | 0 | 4.072 | 0.089 |
| AIPW(1) | 8 | IPW | -2.501 | -2.501 | 0.083 | -0.059 | 1.009 | 0.000 | 0.949 | 3.112 | 0.955 | 2.932 | 0.943 | 3.284 | 3.92 | 0 | 6.913 | 0.217 |
| | 8 | WIPW | -2.501 | -2.498 | 0.085 | -0.025 | 1.021 | 0.002 | 0.947 | 3.180 | 0.950 | 3.094 | 0.941 | 3.338 | 3.92 | 0 | 7.268 | 0.290 |
| | 8 | AIPW | -2.501 | -2.500 | 0.067 | -0.034 | 1.005 | 0.000 | 0.953 | 2.999 | 0.958 | 2.850 | 0.945 | 3.230 | 3.92 | 0 | 4.539 | 0.133 |
| | 8 | WAIPW | -2.501 | -2.498 | 0.065 | 0.002 | 1.021 | 0.003 | 0.950 | 3.077 | 0.952 | 3.035 | 0.946 | 3.208 | 3.92 | 0 | 4.216 | 0.110 |
| AR-1 | 8 | IPW | -2.501 | -2.498 | 0.091 | 0.057 | 1.010 | 0.002 | 0.949 | 3.100 | 0.940 | 3.359 | 0.952 | 3.017 | 3.92 | 0 | 8.369 | 0.177 |
| | 8 | WIPW | -2.501 | -2.502 | 0.088 | 0.021 | 1.015 | -0.001 | 0.949 | 3.100 | 0.946 | 3.191 | 0.949 | 3.106 | 3.92 | 0 | 7.669 | 0.158 |
| | 8 | AIPW | -2.501 | -2.499 | 0.072 | 0.065 | 1.004 | 0.002 | 0.950 | 3.088 | 0.944 | 3.257 | 0.954 | 2.969 | 3.92 | 0 | 5.207 | 0.109 |
| | 8 | WAIPW | -2.501 | -2.502 | 0.071 | 0.027 | 1.009 | -0.001 | 0.948 | 3.152 | 0.946 | 3.208 | 0.952 | 3.029 | 3.92 | 0 | 5.010 | 0.104 |
| AR-2 | 8 | IPW | -2.501 | -2.501 | 0.082 | 0.009 | 1.013 | -0.001 | 0.948 | 3.152 | 0.948 | 3.146 | 0.950 | 3.071 | 3.92 | 0 | 6.759 | 0.140 |
| | 8 | WIPW | -2.501 | -2.503 | 0.080 | -0.004 | 1.014 | -0.002 | 0.947 | 3.180 | 0.949 | 3.112 | 0.948 | 3.140 | 3.92 | 0 | 6.365 | 0.131 |
| | 8 | AIPW | -2.501 | -2.501 | 0.067 | 0.018 | 1.005 | 0.000 | 0.952 | 3.023 | 0.949 | 3.112 | 0.951 | 3.053 | 3.92 | 0 | 4.422 | 0.087 |
| | 8 | WAIPW | -2.501 | -2.502 | 0.066 | 0.000 | 1.009 | -0.001 | 0.951 | 3.047 | 0.951 | 3.041 | 0.947 | 3.180 | 3.92 | 0 | 4.318 | 0.086 |
| IAIPW(0.25) | 8 | IPW | -2.501 | -2.501 | 0.079 | -0.016 | 0.973 | 0.000 | 0.956 | 2.895 | 0.956 | 2.895 | 0.951 | 3.053 | 3.92 | 0 | 6.287 | 0.128 |
| | 8 | WIPW | -2.501 | -2.500 | 0.079 | -0.001 | 0.975 | 0.001 | 0.956 | 2.914 | 0.955 | 2.945 | 0.953 | 2.981 | 3.92 | 0 | 6.182 | 0.126 |
| | 8 | AIPW | -2.501 | -2.501 | 0.066 | -0.012 | 0.989 | 0.000 | 0.954 | 2.951 | 0.957 | 2.882 | 0.949 | 3.112 | 3.92 | 0 | 4.396 | 0.088 |
| | 8 | WAIPW | -2.501 | -2.500 | 0.066 | 0.000 | 0.992 | 0.001 | 0.953 | 2.994 | 0.952 | 3.018 | 0.953 | 2.981 | 3.92 | 0 | 4.413 | 0.089 |
| IAIPW(0.5) | 8 | IPW | -2.501 | -2.500 | 0.079 | -0.012 | 1.003 | 0.001 | 0.951 | 3.059 | 0.950 | 3.077 | 0.949 | 3.100 | 3.92 | 0 | 6.192 | 0.129 |
| | 8 | WIPW | -2.501 | -2.498 | 0.076 | 0.007 | 1.003 | 0.003 | 0.949 | 3.117 | 0.947 | 3.169 | 0.952 | 3.035 | 3.92 | 0 | 5.778 | 0.122 |
| | 8 | AIPW | -2.501 | -2.499 | 0.066 | 0.000 | 1.017 | 0.001 | 0.950 | 3.088 | 0.950 | 3.083 | 0.944 | 3.252 | 3.92 | 0 | 4.384 | 0.089 |
| | 8 | WAIPW | -2.501 | -2.498 | 0.065 | 0.018 | 1.021 | 0.003 | 0.947 | 3.180 | 0.944 | 3.241 | 0.948 | 3.146 | 3.92 | 0 | 4.268 | 0.089 |
| IAIPW(0.75) | 8 | IPW | -2.501 | -2.500 | 0.078 | -0.033 | 1.005 | 0.000 | 0.951 | 3.042 | 0.952 | 3.024 | 0.946 | 3.197 | 3.92 | 0 | 6.041 | 0.132 |
| | 8 | WIPW | -2.501 | -2.497 | 0.074 | 0.002 | 1.012 | 0.004 | 0.948 | 3.141 | 0.951 | 3.065 | 0.950 | 3.089 | 3.92 | 0 | 5.480 | 0.120 |
| | 8 | AIPW | -2.501 | -2.500 | 0.064 | -0.021 | 1.005 | 0.000 | 0.946 | 3.191 | 0.954 | 2.975 | 0.949 | 3.106 | 3.92 | 0 | 4.143 | 0.089 |
| | 8 | WAIPW | -2.501 | -2.498 | 0.063 | 0.007 | 1.017 | 0.002 | 0.944 | 3.241 | 0.949 | 3.118 | 0.948 | 3.152 | 3.92 | 0 | 4.010 | 0.088 |
| IAIPW(1) | 8 | IPW | -2.501 | -2.500 | 0.082 | -0.040 | 1.009 | 0.001 | 0.950 | 3.077 | 0.953 | 3.005 | 0.944 | 3.246 | 3.92 | 0 | 6.693 | 0.162 |
| | 8 | WIPW | -2.501 | -2.496 | 0.076 | 0.005 | 1.016 | 0.005 | 0.948 | 3.129 | 0.945 | 3.224 | 0.947 | 3.163 | 3.92 | 0 | 5.736 | 0.136 |
| | 8 | AIPW | -2.501 | -2.499 | 0.066 | -0.010 | 0.994 | 0.002 | 0.950 | 3.083 | 0.952 | 3.011 | 0.948 | 3.135 | 3.92 | 0 | 4.324 | 0.101 |
| | 8 | WAIPW | -2.501 | -2.496 | 0.064 | 0.028 | 1.013 | 0.004 | 0.944 | 3.241 | 0.945 | 3.224 | 0.950 | 3.077 | 3.92 | 0 | 4.113 | 0.096 |
| IPW(0.25) | 8 | IPW | -2.501 | -2.498 | 0.083 | 0.007 | 1.004 | 0.002 | 0.948 | 3.146 | 0.947 | 3.163 | 0.955 | 2.932 | 3.92 | 0 | 6.873 | 0.148 |
| | 8 | WIPW | -2.501 | -2.496 | 0.092 | 0.031 | 1.005 | 0.004 | 0.949 | 3.118 | 0.948 | 3.135 | 0.955 | 2.938 | 3.92 | 0 | 8.484 | 0.183 |
| | 8 | AIPW | -2.501 | -2.499 | 0.069 | 0.013 | 1.019 | 0.001 | 0.947 | 3.175 | 0.947 | 3.180 | 0.949 | 3.123 | 3.92 | 0 | 4.720 | 0.096 |
| | 8 | WAIPW | -2.501 | -2.499 | 0.069 | 0.019 | 1.022 | 0.002 | 0.945 | 3.225 | 0.944 | 3.247 | 0.949 | 3.106 | 3.92 | 0 | 4.739 | 0.096 |
| IPW(0.5) | 8 | IPW | -2.501 | -2.502 | 0.081 | -0.056 | 1.013 | -0.001 | 0.945 | 3.235 | 0.955 | 2.926 | 0.938 | 3.401 | 3.92 | 0 | 6.504 | 0.138 |
| | 8 | WIPW | -2.501 | -2.498 | 0.086 | -0.004 | 1.022 | 0.002 | 0.947 | 3.169 | 0.950 | 3.077 | 0.945 | 3.224 | 3.92 | 0 | 7.472 | 0.167 |
| | 8 | AIPW | -2.501 | -2.502 | 0.067 | -0.034 | 1.020 | -0.001 | 0.947 | 3.157 | 0.951 | 3.047 | 0.946 | 3.191 | 3.92 | 0 | 4.429 | 0.093 |
| | 8 | WAIPW | -2.501 | -2.500 | 0.066 | -0.012 | 1.024 | 0.001 | 0.947 | 3.157 | 0.947 | 3.157 | 0.945 | 3.230 | 3.92 | 0 | 4.323 | 0.091 |
| IPW(0.75) | 8 | IPW | -2.501 | -2.499 | 0.080 | -0.037 | 0.995 | 0.002 | 0.947 | 3.169 | 0.953 | 2.988 | 0.946 | 3.208 | 3.92 | 0 | 6.474 | 0.164 |
| | 8 | WIPW | -2.501 | -2.495 | 0.084 | 0.024 | 1.008 | 0.006 | 0.950 | 3.089 | 0.944 | 3.258 | 0.952 | 3.030 | 3.92 | 0 | 7.047 | 0.186 |
| | 8 | AIPW | -2.501 | -2.499 | 0.066 | -0.001 | 1.016 | 0.002 | 0.948 | 3.129 | 0.950 | 3.094 | 0.950 | 3.083 | 3.92 | 0 | 4.377 | 0.097 |
| | 8 | WAIPW | -2.501 | -2.497 | 0.065 | 0.027 | 1.026 | 0.004 | 0.946 | 3.191 | 0.946 | 3.197 | 0.951 | 3.059 | 3.92 | 0 | 4.209 | 0.094 |
| IPW(1) | 8 | IPW | -2.501 | -2.499 | 0.085 | -0.063 | 0.993 | 0.001 | 0.950 | 3.094 | 0.957 | 2.856 | 0.944 | 3.257 | 3.92 | 0 | 7.196 | 0.202 |

| Randomization Method | Regime | Estimator | True Value x 10^3 | Mean Estimate x 10^3 | SD Estimates x 10^3 | Mean Normalized | SD Normalized | Mean Bias | CI Coverage | SE CI Coverage x 10^3 | LB Coverage | SE LB Coverage x 10^3 | UB Coverage x 10^3 | SE UB Coverage x 10^3 | Mean CI Length | SE CI Length x 10^3 | MSE x 10^3 | SE MSE x 10^3 |
|---|---|---|---|---|---|---|---|---|---|---|---|---|---|---|---|---|---|---|
| | 8 | WIPW | -2.501 | -2.495 | 0.089 | 0.002 | 1.014 | 0.006 | 0.942 | 3.306 | 0.948 | 3.152 | 0.944 | 3.241 | 3.92 | 0 | 7.879 | 0.244 |
| | 8 | AIPW | -2.501 | -2.500 | 0.068 | -0.017 | 1.009 | 0.001 | 0.945 | 3.224 | 0.949 | 3.123 | 0.946 | 3.197 | 3.92 | 0 | 4.622 | 0.105 |
| | 8 | WAIPW | -2.501 | -2.497 | 0.066 | 0.018 | 1.019 | 0.004 | 0.940 | 3.348 | 0.943 | 3.284 | 0.946 | 3.191 | 3.92 | 0 | 4.306 | 0.097 |
| SR | 8 | IPW | -2.501 | -2.499 | 0.090 | 0.016 | 0.995 | 0.002 | 0.949 | 3.112 | 0.952 | 3.011 | 0.950 | 3.071 | 3.92 | 0 | 8.143 | 0.165 |
| | 8 | WIPW | -2.501 | -2.498 | 0.109 | 0.026 | 1.010 | 0.003 | 0.943 | 3.268 | 0.946 | 3.191 | 0.946 | 3.197 | 3.92 | 0 | 11.853 | 0.238 |
| | 8 | AIPW | -2.501 | -2.499 | 0.074 | 0.024 | 1.009 | 0.002 | 0.944 | 3.252 | 0.945 | 3.235 | 0.952 | 3.017 | 3.92 | 0 | 5.442 | 0.114 |
| | 8 | WAIPW | -2.501 | -2.499 | 0.074 | 0.025 | 1.008 | 0.002 | 0.947 | 3.174 | 0.946 | 3.191 | 0.952 | 3.017 | 3.92 | 0 | 5.521 | 0.115 |
| TS(0.25) | 8 | IPW | -2.501 | -2.499 | 0.075 | 0.001 | 1.002 | 0.002 | 0.940 | 3.364 | 0.948 | 3.135 | 0.945 | 3.224 | 3.92 | 0 | 5.563 | 0.122 |
| | 8 | WIPW | -2.501 | -2.497 | 0.071 | 0.022 | 1.005 | 0.003 | 0.940 | 3.348 | 0.946 | 3.202 | 0.947 | 3.157 | 3.92 | 0 | 5.037 | 0.107 |
| | 8 | AIPW | -2.501 | -2.499 | 0.063 | 0.016 | 1.004 | 0.002 | 0.947 | 3.174 | 0.948 | 3.129 | 0.946 | 3.197 | 3.92 | 0 | 3.956 | 0.087 |
| | 8 | WAIPW | -2.501 | -2.498 | 0.061 | 0.031 | 1.012 | 0.003 | 0.942 | 3.295 | 0.946 | 3.191 | 0.949 | 3.112 | 3.92 | 0 | 3.769 | 0.079 |
| TS(0.50) | 8 | IPW | -2.501 | -2.500 | 0.074 | -0.017 | 0.989 | 0.001 | 0.951 | 3.047 | 0.952 | 3.017 | 0.950 | 3.077 | 3.92 | 0 | 5.414 | 0.116 |
| | 8 | WIPW | -2.501 | -2.497 | 0.069 | 0.015 | 0.994 | 0.004 | 0.951 | 3.065 | 0.948 | 3.152 | 0.951 | 3.041 | 3.92 | 0 | 4.801 | 0.105 |
| | 8 | AIPW | -2.501 | -2.499 | 0.062 | 0.000 | 0.992 | 0.002 | 0.950 | 3.088 | 0.948 | 3.135 | 0.951 | 3.041 | 3.92 | 0 | 3.827 | 0.082 |
| | 8 | WAIPW | -2.501 | -2.497 | 0.061 | 0.023 | 1.004 | 0.003 | 0.947 | 3.157 | 0.945 | 3.235 | 0.953 | 2.999 | 3.92 | 0 | 3.685 | 0.082 |
| TS(0.75) | 8 | IPW | -2.501 | -2.499 | 0.077 | -0.030 | 0.994 | 0.001 | 0.953 | 2.999 | 0.955 | 2.920 | 0.950 | 3.094 | 3.92 | 0 | 5.862 | 0.150 |
| | 8 | WIPW | -2.501 | -2.496 | 0.072 | 0.015 | 1.003 | 0.005 | 0.952 | 3.029 | 0.950 | 3.094 | 0.951 | 3.041 | 3.92 | 0 | 5.146 | 0.138 |
| | 8 | AIPW | -2.501 | -2.499 | 0.063 | -0.004 | 0.978 | 0.002 | 0.958 | 2.837 | 0.955 | 2.920 | 0.952 | 3.029 | 3.92 | 0 | 4.008 | 0.100 |
| | 8 | WAIPW | -2.501 | -2.496 | 0.062 | 0.034 | 0.996 | 0.004 | 0.951 | 3.053 | 0.947 | 3.169 | 0.957 | 2.882 | 3.92 | 0 | 3.814 | 0.101 |
| TS(1) | 8 | IPW | -2.501 | -2.501 | 0.081 | -0.070 | 0.991 | -0.001 | 0.954 | 2.951 | 0.960 | 2.758 | 0.946 | 3.197 | 3.92 | 0 | 6.595 | 0.199 |
| | 8 | WIPW | -2.501 | -2.497 | 0.072 | -0.013 | 0.987 | 0.004 | 0.954 | 2.951 | 0.954 | 2.969 | 0.952 | 3.017 | 3.92 | 0 | 5.165 | 0.140 |
| | 8 | AIPW | -2.501 | -2.501 | 0.066 | -0.047 | 0.968 | 0.000 | 0.954 | 2.969 | 0.957 | 2.882 | 0.952 | 3.017 | 3.92 | 0 | 4.327 | 0.130 |
| | 8 | WAIPW | -2.501 | -2.498 | 0.061 | -0.002 | 0.983 | 0.003 | 0.952 | 3.023 | 0.950 | 3.083 | 0.956 | 2.907 | 3.92 | 0 | 3.784 | 0.096 |
| WAIPW(0.25) | 8 | IPW | -2.501 | -2.500 | 0.081 | -0.005 | 0.997 | 0.001 | 0.951 | 3.065 | 0.953 | 2.981 | 0.952 | 3.024 | 3.92 | 0 | 6.589 | 0.135 |
| | 8 | WIPW | -2.501 | -2.499 | 0.080 | 0.006 | 1.000 | 0.002 | 0.948 | 3.135 | 0.950 | 3.083 | 0.954 | 2.969 | 3.92 | 0 | 6.480 | 0.134 |
| | 8 | AIPW | -2.501 | -2.500 | 0.068 | -0.003 | 1.007 | 0.001 | 0.949 | 3.112 | 0.948 | 3.146 | 0.948 | 3.141 | 3.92 | 0 | 4.561 | 0.093 |
| | 8 | WAIPW | -2.501 | -2.499 | 0.068 | 0.006 | 1.010 | 0.001 | 0.948 | 3.141 | 0.947 | 3.169 | 0.951 | 3.048 | 3.92 | 0 | 4.572 | 0.093 |
| WAIPW(0.5) | 8 | IPW | -2.501 | -2.499 | 0.077 | 0.002 | 0.982 | 0.002 | 0.954 | 2.963 | 0.954 | 2.969 | 0.955 | 2.926 | 3.92 | 0 | 5.915 | 0.126 |
| | 8 | WIPW | -2.501 | -2.497 | 0.075 | 0.024 | 0.989 | 0.004 | 0.953 | 2.999 | 0.950 | 3.088 | 0.955 | 2.926 | 3.92 | 0 | 5.597 | 0.122 |
| | 8 | AIPW | -2.501 | -2.499 | 0.064 | 0.006 | 0.984 | 0.002 | 0.952 | 3.023 | 0.951 | 3.065 | 0.954 | 2.957 | 3.92 | 0 | 4.075 | 0.085 |
| | 8 | WAIPW | -2.501 | -2.498 | 0.063 | 0.022 | 0.992 | 0.003 | 0.951 | 3.047 | 0.947 | 3.157 | 0.956 | 2.913 | 3.92 | 0 | 4.005 | 0.084 |
| WAIPW(0.75) | 8 | IPW | -2.501 | -2.501 | 0.078 | -0.041 | 0.998 | 0.000 | 0.951 | 3.059 | 0.956 | 2.914 | 0.941 | 3.328 | 3.92 | 0 | 6.033 | 0.137 |
| | 8 | WIPW | -2.501 | -2.498 | 0.074 | -0.006 | 1.010 | 0.003 | 0.947 | 3.169 | 0.950 | 3.094 | 0.946 | 3.208 | 3.92 | 0 | 5.507 | 0.132 |
| | 8 | AIPW | -2.501 | -2.501 | 0.065 | -0.030 | 1.006 | 0.000 | 0.948 | 3.152 | 0.955 | 2.926 | 0.948 | 3.146 | 3.92 | 0 | 4.196 | 0.094 |
| | 8 | WAIPW | -2.501 | -2.498 | 0.064 | 0.001 | 1.022 | 0.002 | 0.945 | 3.214 | 0.951 | 3.053 | 0.946 | 3.186 | 3.92 | 0 | 4.079 | 0.093 |
| WAIPW(1) | 8 | IPW | -2.501 | -2.499 | 0.082 | -0.039 | 1.010 | 0.001 | 0.947 | 3.163 | 0.954 | 2.975 | 0.942 | 3.311 | 3.92 | 0 | 6.714 | 0.187 |
| | 8 | WIPW | -2.501 | -2.496 | 0.076 | 0.006 | 1.018 | 0.005 | 0.944 | 3.241 | 0.947 | 3.169 | 0.946 | 3.197 | 3.92 | 0 | 5.765 | 0.153 |
| | 8 | AIPW | -2.501 | -2.500 | 0.067 | -0.035 | 1.003 | 0.000 | 0.954 | 2.969 | 0.954 | 2.969 | 0.949 | 3.112 | 3.92 | 0 | 4.456 | 0.114 |
| | 8 | WAIPW | -2.501 | -2.498 | 0.065 | 0.002 | 1.020 | 0.003 | 0.948 | 3.146 | 0.947 | 3.174 | 0.949 | 3.100 | 3.92 | 0 | 4.196 | 0.108 |
| WIPW(0.25) | 8 | IPW | -2.501 | -2.500 | 0.082 | -0.007 | 1.000 | 0.001 | 0.949 | 3.100 | 0.954 | 2.963 | 0.949 | 3.123 | 3.92 | 0 | 6.764 | 0.138 |
| | 8 | WIPW | -2.501 | -2.498 | 0.082 | 0.014 | 1.006 | 0.003 | 0.946 | 3.203 | 0.951 | 3.065 | 0.949 | 3.118 | 3.92 | 0 | 6.717 | 0.137 |
| | 8 | AIPW | -2.501 | -2.500 | 0.068 | -0.001 | 1.010 | 0.001 | 0.942 | 3.312 | 0.948 | 3.141 | 0.945 | 3.236 | 3.92 | 0 | 4.646 | 0.094 |
| | 8 | WAIPW | -2.501 | -2.499 | 0.068 | 0.009 | 1.013 | 0.001 | 0.941 | 3.338 | 0.947 | 3.175 | 0.944 | 3.252 | 3.92 | 0 | 4.651 | 0.093 |
| WIPW(0.5) | 8 | IPW | -2.501 | -2.502 | 0.079 | -0.050 | 0.989 | -0.001 | 0.950 | 3.094 | 0.960 | 2.758 | 0.944 | 3.241 | 3.92 | 0 | 6.191 | 0.130 |

| Randomization Method | Regime | Estimator | True Value x 10^3 | Mean Estimate x 10^3 | SD Estimates x 10^3 | Mean Normalized | SD Normalized | Mean Bias | CI Coverage | SE CI Coverage x 10^3 | LB Coverage | SE LB Coverage x 10^3 | UB Coverage x 10^3 | SE UB Coverage x 10^3 | Mean CI Length | SE CI Length x 10^3 | MSE x 10^3 | SE MSE x 10^3 |
|---|---|---|---|---|---|---|---|---|---|---|---|---|---|---|---|---|---|---|
| | 8 | WIPW | -2.501 | -2.498 | 0.077 | -0.012 | 1.005 | 0.003 | 0.948 | 3.146 | 0.954 | 2.975 | 0.947 | 3.180 | 3.92 | 0 | 5.979 | 0.127 |
| | 8 | AIPW | -2.501 | -2.501 | 0.065 | -0.028 | 0.994 | -0.001 | 0.952 | 3.011 | 0.959 | 2.791 | 0.947 | 3.180 | 3.92 | 0 | 4.194 | 0.083 |
| | 8 | WAIPW | -2.501 | -2.500 | 0.064 | -0.012 | 1.001 | 0.000 | 0.951 | 3.053 | 0.957 | 2.875 | 0.946 | 3.191 | 3.92 | 0 | 4.115 | 0.082 |
| WIPW(0.75) | 8 | IPW | -2.501 | -2.501 | 0.080 | -0.061 | 0.981 | 0.000 | 0.958 | 2.824 | 0.961 | 2.732 | 0.950 | 3.089 | 3.92 | 0 | 6.320 | 0.165 |
| | 8 | WIPW | -2.501 | -2.495 | 0.077 | -0.004 | 0.998 | 0.005 | 0.954 | 2.969 | 0.951 | 3.053 | 0.951 | 3.042 | 3.92 | 0 | 5.883 | 0.153 |
| | 8 | AIPW | -2.501 | -2.501 | 0.064 | -0.033 | 0.982 | -0.001 | 0.954 | 2.957 | 0.954 | 2.957 | 0.952 | 3.030 | 3.92 | 0 | 4.113 | 0.090 |
| | 8 | WAIPW | -2.501 | -2.499 | 0.063 | -0.004 | 0.991 | 0.002 | 0.949 | 3.112 | 0.954 | 2.975 | 0.953 | 2.988 | 3.92 | 0 | 3.942 | 0.089 |
| WIPW(1) | 8 | IPW | -2.501 | -2.499 | 0.086 | -0.062 | 1.000 | 0.001 | 0.949 | 3.106 | 0.960 | 2.765 | 0.940 | 3.348 | 3.92 | 0 | 7.346 | 0.238 |
| | 8 | WIPW | -2.501 | -2.493 | 0.080 | 0.012 | 1.020 | 0.008 | 0.945 | 3.230 | 0.948 | 3.129 | 0.944 | 3.246 | 3.92 | 0 | 6.465 | 0.187 |
| | 8 | AIPW | -2.501 | -2.500 | 0.068 | -0.021 | 1.001 | 0.001 | 0.951 | 3.065 | 0.955 | 2.938 | 0.948 | 3.140 | 3.92 | 0 | 4.628 | 0.123 |
| | 8 | WAIPW | -2.501 | -2.498 | 0.065 | 0.010 | 1.011 | 0.003 | 0.950 | 3.088 | 0.950 | 3.083 | 0.952 | 3.023 | 3.92 | 0 | 4.193 | 0.104 |

In the following table, for each randomization method and estimator, we present the proportion of trials that the optimal regime is estimated to be optimal using all 1000 subjects' data and its corresponding Monte Carlo standard error (multiplied by 1000).

Identifying the Optimal Regime Results for the Cancer Pain Management SMART, Continuous Outcome, Different Mean Outcomes Scenario.

| Randomization Method | Estimator | Proportion Estimating d1 Optimal | SE Proprtion d1 x 10^3 | Proportion Estimating d2 Optimal | SE Proprtion d2 x 10^3 | Proportion Estimating d3 Optimal | SE Proprtion d3 x 10^3 | Proportion Estimating d4 Optimal | SE Proprtion d4 x 10^3 | Proportion Estimating d5 Optimal | SE Proprtion d5 x 10^3 | Proportion Estimating d6 Optimal | SE Proprtion d6 x 10^3 | Proportion Estimating d7 Optimal | SE Proprtion d7 x 10^3 | Proportion Estimating d8 Optimal | SE Proprtion d8 x 10^3 |
|---|---|---|---|---|---|---|---|---|---|---|---|---|---|---|---|---|---|
| AIPW(0.25) | IPW | 0.000 | 0.000 | 0.000 | 0.000 | 0 | 0 | 0.000 | 0.000 | 0.106 | 4.347 | 0.090 | 4.052 | 0.348 | 6.739 | 0.456 | 7.044 |
| | WIPW | 0.000 | 0.000 | 0.000 | 0.000 | 0 | 0 | 0.000 | 0.000 | 0.127 | 4.707 | 0.103 | 4.296 | 0.339 | 6.696 | 0.431 | 7.004 |
| | AIPW | 0.000 | 0.000 | 0.000 | 0.000 | 0 | 0 | 0.000 | 0.000 | 0.077 | 3.780 | 0.049 | 3.042 | 0.321 | 6.606 | 0.553 | 7.033 |
| | WAIPW | 0.000 | 0.000 | 0.000 | 0.000 | 0 | 0 | 0.000 | 0.000 | 0.077 | 3.771 | 0.049 | 3.065 | 0.339 | 6.695 | 0.535 | 7.055 |
| AIPW(0.5) | IPW | 0.000 | 0.000 | 0.000 | 0.000 | 0 | 0 | 0.000 | 0.000 | 0.114 | 4.498 | 0.090 | 4.040 | 0.342 | 6.709 | 0.454 | 7.042 |
| | WIPW | 0.000 | 0.000 | 0.000 | 0.000 | 0 | 0 | 0.000 | 0.000 | 0.123 | 4.649 | 0.101 | 4.269 | 0.339 | 6.696 | 0.436 | 7.014 |
| | AIPW | 0.000 | 0.000 | 0.000 | 0.000 | 0 | 0 | 0.000 | 0.000 | 0.074 | 3.712 | 0.042 | 2.831 | 0.326 | 6.628 | 0.558 | 7.024 |
| | WAIPW | 0.000 | 0.000 | 0.000 | 0.000 | 0 | 0 | 0.000 | 0.000 | 0.075 | 3.716 | 0.041 | 2.798 | 0.342 | 6.708 | 0.543 | 7.046 |
| AIPW(0.75) | IPW | 0.000 | 0.000 | 0.000 | 0.000 | 0 | 0 | 0.000 | 0.000 | 0.121 | 4.610 | 0.095 | 4.155 | 0.342 | 6.709 | 0.442 | 7.024 |
| | WIPW | 0.000 | 0.000 | 0.000 | 0.000 | 0 | 0 | 0.000 | 0.000 | 0.129 | 4.741 | 0.108 | 4.383 | 0.339 | 6.694 | 0.425 | 6.991 |
| | AIPW | 0.000 | 0.000 | 0.000 | 0.000 | 0 | 0 | 0.000 | 0.000 | 0.074 | 3.712 | 0.046 | 2.969 | 0.333 | 6.666 | 0.546 | 7.041 |
| | WAIPW | 0.000 | 0.000 | 0.000 | 0.000 | 0 | 0 | 0.000 | 0.000 | 0.069 | 3.595 | 0.042 | 2.844 | 0.349 | 6.743 | 0.539 | 7.050 |
| AIPW(1) | IPW | 0.000 | 0.000 | 0.000 | 0.000 | 0 | 0 | 0.000 | 0.000 | 0.137 | 4.857 | 0.107 | 4.376 | 0.327 | 6.637 | 0.429 | 7.000 |
| | WIPW | 0.000 | 0.000 | 0.000 | 0.000 | 0 | 0 | 0.000 | 0.000 | 0.127 | 4.716 | 0.114 | 4.492 | 0.348 | 6.736 | 0.411 | 6.959 |
| | AIPW | 0.000 | 0.000 | 0.000 | 0.000 | 0 | 0 | 0.000 | 0.000 | 0.087 | 3.978 | 0.050 | 3.071 | 0.332 | 6.659 | 0.532 | 7.057 |
| | WAIPW | 0.000 | 0.000 | 0.000 | 0.000 | 0 | 0 | 0.000 | 0.000 | 0.068 | 3.561 | 0.043 | 2.882 | 0.356 | 6.774 | 0.532 | 7.057 |
| AR-1 | IPW | 0.000 | 0.000 | 0.000 | 0.000 | 0 | 0 | 0.000 | 0.000 | 0.113 | 4.481 | 0.077 | 3.766 | 0.342 | 6.709 | 0.468 | 7.057 |
| | WIPW | 0.000 | 0.000 | 0.000 | 0.000 | 0 | 0 | 0.000 | 0.000 | 0.104 | 4.314 | 0.076 | 3.743 | 0.341 | 6.704 | 0.480 | 7.066 |
| | AIPW | 0.000 | 0.000 | 0.000 | 0.000 | 0 | 0 | 0.000 | 0.000 | 0.069 | 3.590 | 0.041 | 2.805 | 0.344 | 6.718 | 0.546 | 7.042 |
| | WAIPW | 0.000 | 0.000 | 0.000 | 0.000 | 0 | 0 | 0.000 | 0.000 | 0.064 | 3.457 | 0.039 | 2.745 | 0.339 | 6.693 | 0.558 | 7.023 |
| AR-2 | IPW | 0.000 | 0.000 | 0.000 | 0.000 | 0 | 0 | 0.000 | 0.000 | 0.106 | 4.354 | 0.072 | 3.651 | 0.338 | 6.690 | 0.484 | 7.068 |
| | WIPW | 0.000 | 0.000 | 0.000 | 0.000 | 0 | 0 | 0.000 | 0.000 | 0.101 | 4.254 | 0.069 | 3.585 | 0.343 | 6.713 | 0.488 | 7.070 |
| | AIPW | 0.000 | 0.000 | 0.000 | 0.000 | 0 | 0 | 0.000 | 0.000 | 0.063 | 3.426 | 0.033 | 2.527 | 0.335 | 6.677 | 0.569 | 7.004 |
| | WAIPW | 0.000 | 0.000 | 0.000 | 0.000 | 0 | 0 | 0.000 | 0.000 | 0.056 | 3.263 | 0.031 | 2.467 | 0.331 | 6.654 | 0.582 | 6.977 |
| IAIPW(0.25) | IPW | 0.000 | 0.000 | 0.000 | 0.000 | 0 | 0 | 0.000 | 0.000 | 0.116 | 4.533 | 0.089 | 4.032 | 0.347 | 6.731 | 0.448 | 7.033 |
| | WIPW | 0.000 | 0.000 | 0.000 | 0.000 | 0 | 0 | 0.000 | 0.000 | 0.113 | 4.475 | 0.088 | 3.999 | 0.355 | 6.767 | 0.445 | 7.028 |
| | AIPW | 0.000 | 0.000 | 0.000 | 0.000 | 0 | 0 | 0.000 | 0.000 | 0.076 | 3.753 | 0.044 | 2.914 | 0.327 | 6.634 | 0.553 | 7.033 |

| Randomization Method | Estimator | Proportion Estimating d1 Optimal | SE Proprtion d1 x 10^3 | Proportion Estimating d2 Optimal | SE Proprtion d2 x 10^3 | Proportion Estimating d3 Optimal | SE Proprtion d3 x 10^3 | Proportion Estimating d4 Optimal | SE Proprtion d4 x 10^3 | Proportion Estimating d5 Optimal | SE Proprtion d5 x 10^3 | Proportion Estimating d6 Optimal | SE Proprtion d6 x 10^3 | Proportion Estimating d7 Optimal | SE Proprtion d7 x 10^3 | Proportion Estimating d8 Optimal | SE Proprtion d8 x 10^3 |
|---|---|---|---|---|---|---|---|---|---|---|---|---|---|---|---|---|---|
| | WAIPW | 0.000 | 0.000 | 0.000 | 0.000 | 0 | 0 | 0.000 | 0.000 | 0.079 | 3.811 | 0.047 | 3.006 | 0.336 | 6.680 | 0.538 | 7.051 |
| IAIPW(0.5) | IPW | 0.000 | 0.000 | 0.000 | 0.000 | 0 | 0 | 0.000 | 0.000 | 0.118 | 4.563 | 0.089 | 4.023 | 0.353 | 6.757 | 0.441 | 7.022 |
| | WIPW | 0.000 | 0.000 | 0.000 | 0.000 | 0 | 0 | 0.000 | 0.000 | 0.112 | 4.457 | 0.083 | 3.893 | 0.354 | 6.762 | 0.452 | 7.039 |
| | AIPW | 0.000 | 0.000 | 0.000 | 0.000 | 0 | 0 | 0.000 | 0.000 | 0.075 | 3.730 | 0.049 | 3.053 | 0.320 | 6.600 | 0.555 | 7.028 |
| | WAIPW | 0.000 | 0.000 | 0.000 | 0.000 | 0 | 0 | 0.000 | 0.000 | 0.073 | 3.675 | 0.049 | 3.047 | 0.333 | 6.667 | 0.545 | 7.043 |
| IAIPW(0.75) | IPW | 0.000 | 0.000 | 0.000 | 0.000 | 0 | 0 | 0.000 | 0.000 | 0.115 | 4.512 | 0.093 | 4.112 | 0.355 | 6.766 | 0.437 | 7.016 |
| | WIPW | 0.000 | 0.000 | 0.000 | 0.000 | 0 | 0 | 0.000 | 0.000 | 0.104 | 4.314 | 0.087 | 3.987 | 0.364 | 6.806 | 0.445 | 7.029 |
| | AIPW | 0.000 | 0.000 | 0.000 | 0.000 | 0 | 0 | 0.000 | 0.000 | 0.065 | 3.487 | 0.044 | 2.907 | 0.344 | 6.721 | 0.546 | 7.041 |
| | WAIPW | 0.000 | 0.000 | 0.000 | 0.000 | 0 | 0 | 0.000 | 0.000 | 0.060 | 3.370 | 0.044 | 2.888 | 0.359 | 6.786 | 0.537 | 7.053 |
| IAIPW(1) | IPW | 0.000 | 0.000 | 0.000 | 0.000 | 0 | 0 | 0.000 | 0.000 | 0.130 | 4.760 | 0.113 | 4.474 | 0.327 | 6.634 | 0.430 | 7.003 |
| | WIPW | 0.000 | 0.000 | 0.000 | 0.000 | 0 | 0 | 0.000 | 0.000 | 0.107 | 4.368 | 0.090 | 4.056 | 0.343 | 6.715 | 0.460 | 7.049 |
| | AIPW | 0.000 | 0.000 | 0.000 | 0.000 | 0 | 0 | 0.000 | 0.000 | 0.089 | 4.023 | 0.053 | 3.157 | 0.324 | 6.620 | 0.534 | 7.055 |
| | WAIPW | 0.000 | 0.000 | 0.000 | 0.000 | 0 | 0 | 0.000 | 0.000 | 0.071 | 3.623 | 0.048 | 3.035 | 0.342 | 6.708 | 0.539 | 7.050 |
| IPW(0.25) | IPW | 0.000 | 0.000 | 0.000 | 0.000 | 0 | 0 | 0.000 | 0.000 | 0.112 | 4.457 | 0.090 | 4.048 | 0.361 | 6.794 | 0.437 | 7.015 |
| | WIPW | 0.000 | 0.000 | 0.000 | 0.000 | 0 | 0 | 0.000 | 0.000 | 0.127 | 4.716 | 0.106 | 4.347 | 0.352 | 6.753 | 0.415 | 6.970 |
| | AIPW | 0.000 | 0.000 | 0.000 | 0.000 | 0 | 0 | 0.000 | 0.000 | 0.073 | 3.680 | 0.049 | 3.042 | 0.331 | 6.658 | 0.547 | 7.041 |
| | WAIPW | 0.000 | 0.000 | 0.000 | 0.000 | 0 | 0 | 0.000 | 0.000 | 0.076 | 3.757 | 0.047 | 2.994 | 0.335 | 6.674 | 0.542 | 7.047 |
| IPW(0.5) | IPW | 0.000 | 0.000 | 0.000 | 0.000 | 0 | 0 | 0.000 | 0.000 | 0.106 | 4.350 | 0.081 | 3.859 | 0.349 | 6.742 | 0.464 | 7.053 |
| | WIPW | 0.000 | 0.000 | 0.000 | 0.000 | 0 | 0 | 0.000 | 0.000 | 0.124 | 4.655 | 0.099 | 4.220 | 0.341 | 6.705 | 0.437 | 7.015 |
| | AIPW | 0.000 | 0.000 | 0.000 | 0.000 | 0 | 0 | 0.000 | 0.000 | 0.085 | 3.940 | 0.043 | 2.863 | 0.317 | 6.583 | 0.555 | 7.029 |
| | WAIPW | 0.000 | 0.000 | 0.000 | 0.000 | 0 | 0 | 0.000 | 0.000 | 0.081 | 3.855 | 0.042 | 2.831 | 0.332 | 6.659 | 0.546 | 7.042 |
| IPW(0.75) | IPW | 0.000 | 0.000 | 0.000 | 0.000 | 0 | 0 | 0.000 | 0.000 | 0.110 | 4.419 | 0.082 | 3.885 | 0.336 | 6.679 | 0.472 | 7.061 |
| | WIPW | 0.000 | 0.000 | 0.000 | 0.000 | 0 | 0 | 0.000 | 0.000 | 0.124 | 4.665 | 0.104 | 4.311 | 0.340 | 6.701 | 0.432 | 7.006 |
| | AIPW | 0.000 | 0.000 | 0.000 | 0.000 | 0 | 0 | 0.000 | 0.000 | 0.075 | 3.726 | 0.045 | 2.920 | 0.326 | 6.628 | 0.555 | 7.029 |
| | WAIPW | 0.000 | 0.000 | 0.000 | 0.000 | 0 | 0 | 0.000 | 0.000 | 0.068 | 3.566 | 0.045 | 2.920 | 0.339 | 6.694 | 0.548 | 7.039 |
| IPW(1) | IPW | 0.000 | 0.000 | 0.000 | 0.000 | 0 | 0 | 0.000 | 0.000 | 0.124 | 4.655 | 0.093 | 4.112 | 0.340 | 6.702 | 0.443 | 7.025 |
| | WIPW | 0.000 | 0.000 | 0.000 | 0.000 | 0 | 0 | 0.000 | 0.000 | 0.136 | 4.845 | 0.108 | 4.386 | 0.340 | 6.700 | 0.416 | 6.972 |
| | AIPW | 0.000 | 0.000 | 0.000 | 0.000 | 0 | 0 | 0.000 | 0.000 | 0.090 | 4.040 | 0.061 | 3.395 | 0.326 | 6.629 | 0.523 | 7.064 |
| | WAIPW | 0.000 | 0.000 | 0.000 | 0.000 | 0 | 0 | 0.000 | 0.000 | 0.074 | 3.707 | 0.050 | 3.088 | 0.345 | 6.722 | 0.531 | 7.058 |
| SR | IPW | 0.000 | 0.000 | 0.000 | 0.000 | 0 | 0 | 0.000 | 0.000 | 0.123 | 4.652 | 0.099 | 4.220 | 0.345 | 6.724 | 0.433 | 7.007 |
| | WIPW | 0.000 | 0.000 | 0.000 | 0.000 | 0 | 0 | 0.000 | 0.000 | 0.149 | 5.034 | 0.115 | 4.519 | 0.339 | 6.696 | 0.397 | 6.919 |
| | AIPW | 0.000 | 0.000 | 0.000 | 0.000 | 0 | 0 | 0.000 | 0.000 | 0.088 | 3.999 | 0.056 | 3.246 | 0.327 | 6.636 | 0.529 | 7.060 |
| | WAIPW | 0.000 | 0.000 | 0.000 | 0.000 | 0 | 0 | 0.000 | 0.000 | 0.089 | 4.031 | 0.057 | 3.284 | 0.338 | 6.690 | 0.516 | 7.068 |
| TS(0.25) | IPW | 0.000 | 0.200 | 0.000 | 0.000 | 0 | 0 | 0.000 | 0.000 | 0.108 | 4.383 | 0.079 | 3.811 | 0.343 | 6.713 | 0.471 | 7.060 |
| | WIPW | 0.000 | 0.000 | 0.000 | 0.000 | 0 | 0 | 0.000 | 0.000 | 0.099 | 4.217 | 0.075 | 3.716 | 0.347 | 6.733 | 0.480 | 7.066 |
| | AIPW | 0.002 | 0.663 | 0.001 | 0.346 | 0 | 0 | 0.003 | 0.799 | 0.072 | 3.647 | 0.035 | 2.585 | 0.320 | 6.595 | 0.568 | 7.006 |
| | WAIPW | 0.000 | 0.000 | 0.000 | 0.000 | 0 | 0 | 0.004 | 0.870 | 0.066 | 3.517 | 0.033 | 2.519 | 0.328 | 6.641 | 0.569 | 7.004 |
| TS(0.50) | IPW | 0.001 | 0.447 | 0.000 | 0.000 | 0 | 0 | 0.000 | 0.000 | 0.116 | 4.533 | 0.091 | 4.072 | 0.328 | 6.641 | 0.463 | 7.053 |
| | WIPW | 0.000 | 0.000 | 0.000 | 0.000 | 0 | 0 | 0.000 | 0.000 | 0.105 | 4.328 | 0.082 | 3.876 | 0.342 | 6.710 | 0.471 | 7.060 |
| | AIPW | 0.004 | 0.870 | 0.003 | 0.747 | 0 | 0 | 0.006 | 1.055 | 0.071 | 3.637 | 0.040 | 2.778 | 0.327 | 6.635 | 0.549 | 7.037 |
| | WAIPW | 0.000 | 0.000 | 0.000 | 0.000 | 0 | 0 | 0.005 | 1.017 | 0.064 | 3.472 | 0.042 | 2.824 | 0.341 | 6.704 | 0.548 | 7.039 |
| TS(0.75) | IPW | 0.001 | 0.447 | 0.000 | 0.200 | 0 | 0 | 0.000 | 0.200 | 0.138 | 4.884 | 0.110 | 4.429 | 0.327 | 6.634 | 0.423 | 6.988 |
| | WIPW | 0.000 | 0.000 | 0.000 | 0.000 | 0 | 0 | 0.000 | 0.000 | 0.116 | 4.526 | 0.092 | 4.080 | 0.341 | 6.703 | 0.452 | 7.039 |
| | AIPW | 0.004 | 0.936 | 0.006 | 1.092 | 0 | 0 | 0.008 | 1.275 | 0.089 | 4.027 | 0.053 | 3.163 | 0.328 | 6.640 | 0.512 | 7.070 |

| Randomization Method | Estimator | Proportion Estimating d1 Optimal | SE Proprtion d1 x 10^3 | Proportion Estimating d2 Optimal | SE Proprtion d2 x 10^3 | Proportion Estimating d3 Optimal | SE Proprtion d3 x 10^3 | Proportion Estimating d4 Optimal | SE Proprtion d4 x 10^3 | Proportion Estimating d5 Optimal | SE Proprtion d5 x 10^3 | Proportion Estimating d6 Optimal | SE Proprtion d6 x 10^3 | Proportion Estimating d7 Optimal | SE Proprtion d7 x 10^3 | Proportion Estimating d8 Optimal | SE Proprtion d8 x 10^3 |
|---|---|---|---|---|---|---|---|---|---|---|---|---|---|---|---|---|---|
| | WAIPW | 0.000 | 0.000 | 0.000 | 0.200 | 0 | 0 | 0.009 | 1.365 | 0.071 | 3.628 | 0.046 | 2.969 | 0.343 | 6.714 | 0.530 | 7.059 |
| TS(1) | IPW | 0.002 | 0.600 | 0.000 | 0.283 | 0 | 0 | 0.000 | 0.000 | 0.153 | 5.094 | 0.121 | 4.609 | 0.320 | 6.597 | 0.404 | 6.940 |
| | WIPW | 0.000 | 0.000 | 0.000 | 0.000 | 0 | 0 | 0.000 | 0.000 | 0.121 | 4.616 | 0.095 | 4.143 | 0.350 | 6.748 | 0.434 | 7.009 |
| | AIPW | 0.005 | 0.978 | 0.008 | 1.260 | 0 | 0 | 0.009 | 1.336 | 0.108 | 4.383 | 0.072 | 3.656 | 0.315 | 6.569 | 0.484 | 7.068 |
| | WAIPW | 0.000 | 0.000 | 0.000 | 0.200 | 0 | 0 | 0.012 | 1.553 | 0.075 | 3.716 | 0.053 | 3.157 | 0.341 | 6.706 | 0.519 | 7.067 |
| WAIPW(0.25) | IPW | 0.000 | 0.000 | 0.000 | 0.000 | 0 | 0 | 0.000 | 0.000 | 0.115 | 4.512 | 0.088 | 4.015 | 0.341 | 6.704 | 0.456 | 7.044 |
| | WIPW | 0.000 | 0.000 | 0.000 | 0.000 | 0 | 0 | 0.000 | 0.000 | 0.114 | 4.502 | 0.085 | 3.936 | 0.346 | 6.727 | 0.455 | 7.043 |
| | AIPW | 0.000 | 0.000 | 0.000 | 0.000 | 0 | 0 | 0.000 | 0.000 | 0.078 | 3.793 | 0.045 | 2.920 | 0.325 | 6.623 | 0.553 | 7.032 |
| | WAIPW | 0.000 | 0.000 | 0.000 | 0.000 | 0 | 0 | 0.000 | 0.000 | 0.077 | 3.771 | 0.045 | 2.932 | 0.337 | 6.684 | 0.541 | 7.048 |
| WAIPW(0.5) | IPW | 0.000 | 0.000 | 0.000 | 0.000 | 0 | 0 | 0.000 | 0.000 | 0.115 | 4.512 | 0.088 | 4.011 | 0.347 | 6.734 | 0.449 | 7.035 |
| | WIPW | 0.000 | 0.000 | 0.000 | 0.000 | 0 | 0 | 0.000 | 0.000 | 0.111 | 4.446 | 0.081 | 3.859 | 0.345 | 6.724 | 0.463 | 7.052 |
| | AIPW | 0.000 | 0.000 | 0.000 | 0.000 | 0 | 0 | 0.000 | 0.000 | 0.073 | 3.689 | 0.040 | 2.758 | 0.328 | 6.639 | 0.559 | 7.022 |
| | WAIPW | 0.000 | 0.000 | 0.000 | 0.000 | 0 | 0 | 0.000 | 0.000 | 0.071 | 3.628 | 0.040 | 2.778 | 0.334 | 6.673 | 0.555 | 7.029 |
| WAIPW(0.75) | IPW | 0.000 | 0.000 | 0.000 | 0.000 | 0 | 0 | 0.000 | 0.000 | 0.115 | 4.509 | 0.099 | 4.217 | 0.346 | 6.729 | 0.440 | 7.021 |
| | WIPW | 0.000 | 0.000 | 0.000 | 0.000 | 0 | 0 | 0.000 | 0.000 | 0.101 | 4.266 | 0.086 | 3.966 | 0.353 | 6.758 | 0.460 | 7.049 |
| | AIPW | 0.000 | 0.000 | 0.000 | 0.000 | 0 | 0 | 0.000 | 0.000 | 0.078 | 3.789 | 0.044 | 2.907 | 0.321 | 6.605 | 0.557 | 7.026 |
| | WAIPW | 0.000 | 0.000 | 0.000 | 0.000 | 0 | 0 | 0.000 | 0.000 | 0.070 | 3.619 | 0.042 | 2.837 | 0.343 | 6.713 | 0.545 | 7.043 |
| WAIPW(1) | IPW | 0.000 | 0.000 | 0.000 | 0.000 | 0 | 0 | 0.000 | 0.000 | 0.141 | 4.925 | 0.110 | 4.429 | 0.340 | 6.698 | 0.409 | 6.954 |
| | WIPW | 0.000 | 0.000 | 0.000 | 0.000 | 0 | 0 | 0.000 | 0.000 | 0.118 | 4.556 | 0.086 | 3.970 | 0.360 | 6.788 | 0.436 | 7.014 |
| | AIPW | 0.000 | 0.000 | 0.000 | 0.000 | 0 | 0 | 0.000 | 0.000 | 0.082 | 3.880 | 0.057 | 3.284 | 0.338 | 6.688 | 0.523 | 7.064 |
| | WAIPW | 0.000 | 0.000 | 0.000 | 0.000 | 0 | 0 | 0.000 | 0.000 | 0.068 | 3.561 | 0.045 | 2.944 | 0.363 | 6.801 | 0.524 | 7.064 |
| WIPW(0.25) | IPW | 0.000 | 0.000 | 0.000 | 0.000 | 0 | 0 | 0.000 | 0.000 | 0.116 | 4.530 | 0.088 | 3.999 | 0.349 | 6.740 | 0.448 | 7.033 |
| | WIPW | 0.000 | 0.000 | 0.000 | 0.000 | 0 | 0 | 0.000 | 0.000 | 0.113 | 4.485 | 0.087 | 3.982 | 0.350 | 6.746 | 0.450 | 7.036 |
| | AIPW | 0.000 | 0.000 | 0.000 | 0.000 | 0 | 0 | 0.000 | 0.000 | 0.082 | 3.872 | 0.043 | 2.882 | 0.328 | 6.642 | 0.547 | 7.041 |
| | WAIPW | 0.000 | 0.000 | 0.000 | 0.000 | 0 | 0 | 0.000 | 0.000 | 0.077 | 3.780 | 0.044 | 2.914 | 0.342 | 6.708 | 0.537 | 7.053 |
| WIPW(0.5) | IPW | 0.000 | 0.000 | 0.000 | 0.000 | 0 | 0 | 0.000 | 0.000 | 0.109 | 4.404 | 0.080 | 3.828 | 0.349 | 6.743 | 0.462 | 7.052 |
| | WIPW | 0.000 | 0.000 | 0.000 | 0.000 | 0 | 0 | 0.000 | 0.000 | 0.107 | 4.379 | 0.077 | 3.775 | 0.348 | 6.735 | 0.468 | 7.057 |
| | AIPW | 0.000 | 0.000 | 0.000 | 0.000 | 0 | 0 | 0.000 | 0.000 | 0.073 | 3.679 | 0.042 | 2.824 | 0.324 | 6.618 | 0.562 | 7.018 |
| | WAIPW | 0.000 | 0.000 | 0.000 | 0.000 | 0 | 0 | 0.000 | 0.000 | 0.068 | 3.556 | 0.045 | 2.944 | 0.339 | 6.693 | 0.548 | 7.039 |
| WIPW(0.75) | IPW | 0.000 | 0.000 | 0.000 | 0.000 | 0 | 0 | 0.000 | 0.000 | 0.105 | 4.329 | 0.088 | 4.015 | 0.360 | 6.790 | 0.447 | 7.031 |
| | WIPW | 0.000 | 0.000 | 0.000 | 0.000 | 0 | 0 | 0.000 | 0.000 | 0.103 | 4.307 | 0.082 | 3.877 | 0.362 | 6.798 | 0.452 | 7.040 |
| | AIPW | 0.000 | 0.000 | 0.000 | 0.000 | 0 | 0 | 0.000 | 0.000 | 0.066 | 3.512 | 0.050 | 3.077 | 0.333 | 6.668 | 0.551 | 7.035 |
| | WAIPW | 0.000 | 0.000 | 0.000 | 0.000 | 0 | 0 | 0.000 | 0.000 | 0.060 | 3.370 | 0.048 | 3.018 | 0.346 | 6.729 | 0.546 | 7.042 |
| WIPW(1) | IPW | 0.000 | 0.000 | 0.000 | 0.000 | 0 | 0 | 0.000 | 0.000 | 0.122 | 4.622 | 0.097 | 4.182 | 0.338 | 6.689 | 0.444 | 7.027 |
| | WIPW | 0.000 | 0.000 | 0.000 | 0.000 | 0 | 0 | 0.000 | 0.000 | 0.105 | 4.336 | 0.083 | 3.893 | 0.352 | 6.755 | 0.460 | 7.050 |
| | AIPW | 0.000 | 0.000 | 0.000 | 0.000 | 0 | 0 | 0.000 | 0.000 | 0.082 | 3.889 | 0.062 | 3.401 | 0.322 | 6.608 | 0.534 | 7.055 |
| | WAIPW | 0.000 | 0.000 | 0.000 | 0.000 | 0 | 0 | 0.000 | 0.000 | 0.072 | 3.661 | 0.046 | 2.969 | 0.341 | 6.707 | 0.540 | 7.049 |

The following QQ-plots display the normalized estimates for regime 1 and regime 8 from all 5000 trials under WAIPW(1) and TS(1) randomization for all four estimators.

Regime 1 Normalized Estimates for WAIPW(1) Randomization

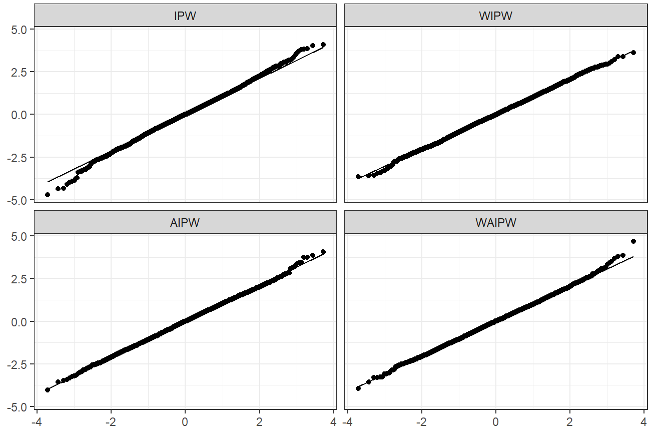


Regime 1 Normalized Estimates for TS(1) Randomization

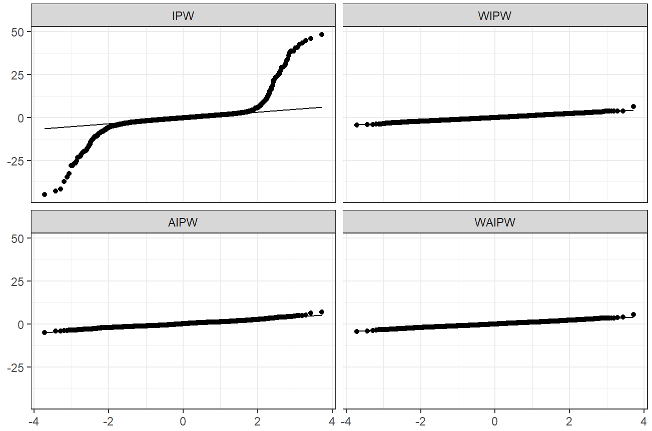


Regime 8 Normalized Estimates for WAIPW(1) Randomization

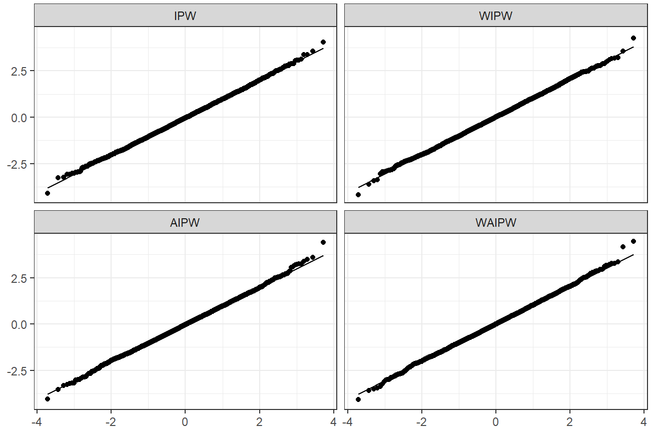

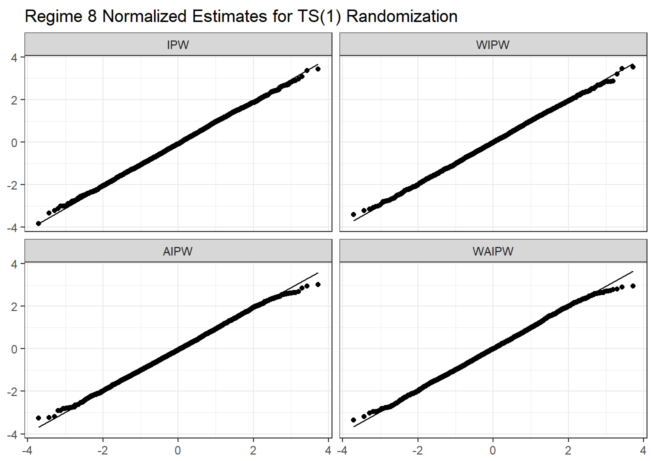


# Cancer Pain Management SMART, Continuous Outcome, Similar Different Mean Outcomes

### C.2.1 Simulation Details

This simulation study is the same as in Section C.1 except for the data generating process. We describe it in full below.

We present results of a simulation study involving 5000 Monte Carlo trials under a scenario mimicking the cancer pain SMART introduced in Section 1.
Each trial enrolls $N = 1000$ subjects, with enrollment times uniform over (integer) weeks 1-24. Upon enrollment, we draw baseline pain score $X_1 \sim N(5, 1)$ and assign stage 1 treatment $A_1 \in \square_1 = \{0, 1\}$. Six weeks after $A_1$ is assigned, second-stage pain score is generated as $X_{2,1} = \gamma_{1,0} + \gamma_{1,1}X_1 + \gamma_{1,2}A_1 + \varepsilon_1$, where $\varepsilon_1 \sim N(0, 1)$, and response status after the first stage is $X_{2,2} = I(X_{2,1} < 0.7X_1)$, which, with $A_1$, dictates the feasible subset of $\square_2 = \{0, 1, 2, 3, 4, 5\}$ from which stage 2 treatment $A_2$ is assigned. Six weeks later, the outcome is generated as $Y = \gamma_{2,0} + \gamma_{2,1}X_1 + \gamma_{2,2}A_1 + \gamma_{2,3}X_{2,1} + \gamma_{2,4}I(A_2 = 1) + \gamma_{2,5}I(A_2 = 2 \text{ or } 5) + \gamma_{2,6}I(A_2 = 3) + \gamma_{2,7}I(A_2 = 4) + \varepsilon_2$, where $\varepsilon_2 \sim N(0, 1)$. With $\gamma_1 = (\gamma_{1,0}, \gamma_{1,1}, \gamma_{1,2})^T = (0.00, 0.90, -0.001)^T$ and $\gamma_2 = (\gamma_{2,0}, \dots, \gamma_{2,7}) = (0.00, 0.05, -0.001, 0.10, -0.001, -0.001, -0.001, -0.001)^T$, for the $m = 8$ embedded regimes defined in Figure 1, $1000 * \{\square(\mathbf{d}^1), \dots, \square(\mathbf{d}^8)\} = (\theta_1, \dots, \theta_8) = (-0.500, -0.500, -1.000, -1.000, -2.100, -2.100, -2.100, -2.100)$. Regimes 5-8 are technically optimal, but the difference is marginal.

The burn-in period ends at the time $t^*$ when each of the $m = 8$ regimes has at least 25 subjects who have completed the trial with experience consistent with following the regime.

We compare the performance of up-front RAR using TS with $c_t = 0.50$ and 1 for all $t$ based on the IPW, WIPW, AIPW, WAIPW, and IAIPW estimators. For the AIPW, WAIPW and IAIPW estimators, $Q_2(\overline{\mathbf{X}}_2, \overline{\mathbf{a}}_2; \beta_2) = \beta_{2,0} + \beta_{2,1}x_1 + \beta_{2,2}I(a_1 = 1) + \beta_{2,3}x_{2,1} + \beta_{2,4}I(a_2 = 1) + \beta_{2,5}I(a_2 = 2 \text{ or } 5) + \beta_{2,6}I(a_2 = 3) + \beta_{2,7}I(a_2 = 4)$ and $Q_1^j(x_1, a_1; \beta_1^j) = \beta_{1,0}^j + \beta_{1,1}^j x_1 + \beta_{1,2}^j a_1 + \beta_{1,3}^j x_1 a_1$.

The sequential methods include Q-learning-based sequential RAR approach using TS with $c_t = 0.25, 0.50, 0.75$ and 1 to SR and two SMART-AR methods, a conservatively-tuned (AR-1) and a more aggressive (AR-2) version. To implement all RAR methods, we posit linear models $Q_2(\overline{\mathbf{X}}_2, \overline{\mathbf{a}}_2; \beta_2) = \beta_{2,0} + \beta_{2,1}x_1 + \beta_{2,2}I(a_1 = 1) + \beta_{2,3}x_{2,1} + \beta_{2,4}I(a_2 = 1) + \beta_{2,5}I(a_2 = 2 \text{ or } 5) + \beta_{2,6}I(a_2 = 3) + \beta_{2,7}I(a_2 = 4)$ and $Q_1(x_1, a_1; \beta_1) = \beta_{1,0} + \beta_{1,1}x_1 + \beta_{1,2}a_1$. For sequential RAR, we set $B_1 = b_2 \times b_1 = 32 \times 32 = 1024$ and $B_2 = 1000$. The tuning parameters for AR-1 are $b = 10, \tau = 0.5$ and $b = 100, \tau = 0.025$ for AR-2 and $\lambda_t = t^{-1}\tau^{(1-b)}$ for both. A clipping constant of 0.05 was imposed on all sequential methods.

At each week, for each RAR method, newly-enrolled subjects are assigned stage 1 treatment using the same randomization probability. Already-enrolled subjects who have reached stage 2 at this week and require stage 2 randomization are partitioned into four groups based on $(a_1, x_{2,2}) = (0, 0), (0, 1), (1, 0), (1, 1)$. Within each group, randomization probabilities are calculated; thus, second-stage probabilities are specific to each stage 1 treatment-response status combination. For both AR methods, we use the sample average of $X_1$ to calculate the the components that make up the stage 2 probabilities.

Tables and figures in the following sections are as in Section C.1.

### C.2.2 In Trial Results

In Trial Results for the Cancer Pain Management SMART, Continuous Outcome, Similar Mean Outcomes Scenario

| Randomization Method | Mean Y | SE Mean Y x 10^3 | Mean Proportion A1=1 | SE Mean Proportion A1=1 x 10^3 | Mean Proportion Cd8=1 | SE Mean Proportion Cd8=1 x 10^3 |
|---|---|---|---|---|---|---|
| AIPW(0.5) | -0.001 | 0.456 | 0.500 | 1.100 | 0.250 | 1099.667 |
| AIPW(1) | -0.002 | 0.455 | 0.501 | 1.664 | 0.249 | 1664.474 |

| Randomization Method | Mean Y | SE Mean Y x 10^3 | Mean Proportion A1=1 | SE Mean Proportion A1=1 x 10^3 | Mean Proportion Cd8=1 | SE Mean Proportion Cd8=1 x 10^3 |
|---|---|---|---|---|---|---|
| AR-1 | -0.003 | 0.457 | 0.531 | 2.887 | 0.272 | 2886.657 |
| AR-2 | -0.002 | 0.456 | 0.530 | 2.587 | 0.267 | 2587.419 |
| IAIPW(0.5) | -0.001 | 0.455 | 0.500 | 1.103 | 0.250 | 1102.759 |
| IAIPW(1) | -0.002 | 0.461 | 0.504 | 1.675 | 0.251 | 1675.153 |
| IPW(0.5) | -0.001 | 0.457 | 0.502 | 1.077 | 0.251 | 1077.086 |
| IPW(1) | -0.001 | 0.450 | 0.506 | 1.680 | 0.252 | 1679.996 |
| SR | -0.001 | 0.455 | 0.500 | 0.226 | 0.250 | 225.569 |
| TS(0.25) | -0.002 | 0.450 | 0.501 | 1.076 | 0.249 | 1076.350 |
| TS(0.50) | -0.001 | 0.452 | 0.500 | 1.546 | 0.245 | 1546.387 |
| TS(0.75) | -0.001 | 0.457 | 0.497 | 1.961 | 0.243 | 1961.443 |
| TS(1) | -0.001 | 0.456 | 0.503 | 2.253 | 0.240 | 2252.960 |
| WAIPW(0.5) | -0.001 | 0.448 | 0.503 | 1.122 | 0.251 | 1121.924 |
| WAIPW(1) | -0.001 | 0.459 | 0.507 | 1.839 | 0.254 | 1838.605 |
| WIPW(0.5) | -0.002 | 0.459 | 0.503 | 1.087 | 0.251 | 1087.339 |
| WIPW(1) | -0.002 | 0.453 | 0.501 | 1.823 | 0.250 | 1823.420 |

### C.2.3 Estimation Results

Estimation Results for the Cancer Pain Management SMART, Continuous Outcome, Similar Mean Outcomes Scenario

| Randomization Method | Regime | Estimator | True Value x 10^3 | Mean Estimate x 10^3 | SD Estimates x 10^3 | Mean Normalized | SD Normalized | Mean Bias | CI Coverage | SE CI Coverage x 10^3 | LB Coverage | SE LB Coverage x 10^3 | UB Coverage | SE UB Coverage x 10^3 | Mean CI Length | SE CI Length x 10^3 | MSE x 10^3 | SE MSE x 10^3 |
|---|---|---|---|---|---|---|---|---|---|---|---|---|---|---|---|---|---|---|
| AIPW(0.5) | 1 | IPW | -0.5 | 0.133 | 0.071 | -0.061 | 1.019 | 0.001 | 0.946 | 3.202 | 0.948 | 3.146 | 0.940 | 3.359 | 0.271 | 0.528 | 4.999 | 0.112 |
| | 1 | WIPW | -0.5 | 3.461 | 0.086 | 0.003 | 1.023 | 0.004 | 0.946 | 3.208 | 0.939 | 3.395 | 0.947 | 3.157 | 0.326 | 0.866 | 7.354 | 0.165 |
| | 1 | AIPW | -0.5 | 0.170 | 0.070 | -0.059 | 1.023 | 0.001 | 0.942 | 3.295 | 0.949 | 3.112 | 0.941 | 3.333 | 0.268 | 0.526 | 4.898 | 0.107 |
| | 1 | WAIPW | -0.5 | 4.223 | 0.069 | 0.005 | 1.022 | 0.005 | 0.946 | 3.186 | 0.944 | 3.246 | 0.947 | 3.180 | 0.264 | 0.461 | 4.765 | 0.104 |
| AIPW(1) | 1 | IPW | -0.5 | -1.742 | 0.095 | -0.133 | 1.032 | -0.001 | 0.939 | 3.375 | 0.954 | 2.951 | 0.933 | 3.531 | 0.349 | 1.650 | 8.996 | 0.240 |
| | 1 | WIPW | -0.5 | 7.901 | 0.110 | -0.004 | 1.033 | 0.008 | 0.939 | 3.390 | 0.943 | 3.268 | 0.947 | 3.169 | 0.400 | 2.075 | 12.256 | 0.343 |
| | 1 | AIPW | -0.5 | -1.441 | 0.094 | -0.122 | 1.028 | -0.001 | 0.939 | 3.375 | 0.954 | 2.957 | 0.937 | 3.441 | 0.349 | 1.742 | 8.900 | 0.238 |
| | 1 | WAIPW | -0.5 | 7.450 | 0.078 | 0.000 | 1.017 | 0.008 | 0.947 | 3.169 | 0.944 | 3.246 | 0.948 | 3.129 | 0.295 | 0.903 | 6.136 | 0.154 |
| AR-1 | 1 | IPW | -0.5 | -0.898 | 0.130 | 0.000 | 1.056 | 0.000 | 0.935 | 3.477 | 0.941 | 3.338 | 0.942 | 3.317 | 0.435 | 3.042 | 16.824 | 0.535 |
| | 1 | WIPW | -0.5 | -3.647 | 0.088 | -0.025 | 1.009 | -0.003 | 0.949 | 3.112 | 0.950 | 3.077 | 0.947 | 3.180 | 0.322 | 1.572 | 7.736 | 0.208 |
| | 1 | AIPW | -0.5 | -0.866 | 0.130 | -0.014 | 1.024 | 0.000 | 0.946 | 3.186 | 0.948 | 3.152 | 0.946 | 3.186 | 0.445 | 3.497 | 16.788 | 0.554 |
| | 1 | WAIPW | -0.5 | -4.076 | 0.092 | -0.039 | 1.006 | -0.004 | 0.949 | 3.106 | 0.954 | 2.963 | 0.947 | 3.180 | 0.338 | 1.853 | 8.473 | 0.228 |
| AR-2 | 1 | IPW | -0.5 | -2.039 | 0.109 | -0.003 | 1.054 | -0.002 | 0.939 | 3.380 | 0.940 | 3.348 | 0.941 | 3.343 | 0.376 | 2.384 | 11.842 | 0.337 |
| | 1 | WIPW | -0.5 | -1.779 | 0.080 | -0.005 | 1.018 | -0.001 | 0.946 | 3.186 | 0.949 | 3.106 | 0.946 | 3.202 | 0.298 | 1.240 | 6.476 | 0.161 |
| | 1 | AIPW | -0.5 | -1.833 | 0.109 | 0.000 | 1.035 | -0.001 | 0.944 | 3.241 | 0.943 | 3.268 | 0.944 | 3.246 | 0.378 | 2.527 | 11.793 | 0.345 |
| | 1 | WAIPW | -0.5 | -1.974 | 0.084 | -0.008 | 1.024 | -0.001 | 0.945 | 3.219 | 0.951 | 3.059 | 0.948 | 3.152 | 0.309 | 1.449 | 7.140 | 0.180 |
| IAIPW(0.5) | 1 | IPW | -0.5 | -1.320 | 0.069 | -0.079 | 1.000 | -0.001 | 0.951 | 3.059 | 0.956 | 2.894 | 0.942 | 3.306 | 0.271 | 0.523 | 4.781 | 0.099 |
| | 1 | WIPW | -0.5 | 3.187 | 0.068 | -0.009 | 1.001 | 0.004 | 0.952 | 3.023 | 0.951 | 3.047 | 0.951 | 3.065 | 0.264 | 0.456 | 4.577 | 0.095 |
| | 1 | AIPW | -0.5 | -1.186 | 0.069 | -0.075 | 1.005 | -0.001 | 0.950 | 3.088 | 0.954 | 2.969 | 0.940 | 3.354 | 0.268 | 0.511 | 4.704 | 0.099 |
| | 1 | WAIPW | -0.5 | 3.514 | 0.068 | -0.002 | 1.006 | 0.004 | 0.950 | 3.077 | 0.949 | 3.106 | 0.947 | 3.174 | 0.264 | 0.450 | 4.588 | 0.096 |
| IAIPW(1) | 1 | IPW | -0.5 | 0.505 | 0.099 | -0.119 | 1.055 | 0.001 | 0.934 | 3.521 | 0.945 | 3.219 | 0.932 | 3.551 | 0.353 | 1.716 | 9.871 | 0.271 |
| | 1 | WIPW | -0.5 | 8.977 | 0.080 | 0.008 | 1.029 | 0.009 | 0.945 | 3.230 | 0.943 | 3.279 | 0.946 | 3.208 | 0.298 | 0.931 | 6.533 | 0.165 |
| | 1 | AIPW | -0.5 | 0.912 | 0.098 | -0.103 | 1.047 | 0.001 | 0.936 | 3.457 | 0.947 | 3.163 | 0.936 | 3.457 | 0.353 | 1.829 | 9.664 | 0.261 |
| | 1 | WAIPW | -0.5 | 9.259 | 0.080 | 0.017 | 1.035 | 0.010 | 0.941 | 3.338 | 0.942 | 3.311 | 0.948 | 3.135 | 0.298 | 0.941 | 6.539 | 0.162 |
| IPW(0.5) | 1 | IPW | -0.5 | 1.656 | 0.070 | -0.037 | 1.000 | 0.002 | 0.949 | 3.112 | 0.954 | 2.957 | 0.950 | 3.077 | 0.271 | 0.514 | 4.863 | 0.106 |

| Randomization Method | Regime | Estimator | True Value x 10^3 | Mean Estimate x 10^3 | SD Estimates x 10^3 | Mean Normalized | SD Normalized | Mean Bias | CI Coverage | SE CI Coverage x 10^3 | LB Coverage | SE LB Coverage x 10^3 | UB Coverage x 10^3 | SE UB Coverage x 10^3 | Mean CI Length | SE CI Length x 10^3 | MSE x 10^3 | SE MSE x 10^3 |
|---|---|---|---|---|---|---|---|---|---|---|---|---|---|---|---|---|---|---|
| | 1 | WIPW | -0.5 | 5.509 | 0.085 | 0.031 | 1.008 | 0.006 | 0.948 | 3.152 | 0.948 | 3.152 | 0.949 | 3.117 | 0.326 | 0.849 | 7.198 | 0.156 |
| | 1 | AIPW | -0.5 | 1.786 | 0.069 | -0.031 | 1.005 | 0.002 | 0.948 | 3.140 | 0.954 | 2.951 | 0.950 | 3.071 | 0.268 | 0.521 | 4.773 | 0.104 |
| | 1 | WAIPW | -0.5 | 6.171 | 0.068 | 0.039 | 1.004 | 0.007 | 0.950 | 3.088 | 0.948 | 3.146 | 0.955 | 2.944 | 0.264 | 0.454 | 4.675 | 0.101 |
| IPW(1) | 1 | IPW | -0.5 | 0.583 | 0.097 | -0.119 | 1.037 | 0.001 | 0.939 | 3.375 | 0.951 | 3.041 | 0.934 | 3.521 | 0.351 | 1.645 | 9.411 | 0.260 |
| | 1 | WIPW | -0.5 | 10.848 | 0.115 | 0.013 | 1.046 | 0.011 | 0.943 | 3.284 | 0.936 | 3.462 | 0.947 | 3.169 | 0.402 | 2.070 | 13.330 | 0.405 |
| | 1 | AIPW | -0.5 | 0.373 | 0.097 | -0.109 | 1.028 | 0.001 | 0.941 | 3.322 | 0.949 | 3.112 | 0.939 | 3.390 | 0.352 | 1.785 | 9.340 | 0.257 |
| | 1 | WAIPW | -0.5 | 8.800 | 0.079 | 0.017 | 1.017 | 0.009 | 0.942 | 3.295 | 0.942 | 3.306 | 0.954 | 2.975 | 0.297 | 0.927 | 6.325 | 0.155 |
| SR | 1 | IPW | -0.5 | 0.148 | 0.064 | 0.009 | 1.004 | 0.001 | 0.953 | 2.999 | 0.951 | 3.065 | 0.953 | 2.993 | 0.251 | 0.189 | 4.115 | 0.082 |
| | 1 | WIPW | -0.5 | -0.236 | 0.077 | 0.002 | 1.011 | 0.000 | 0.950 | 3.088 | 0.952 | 3.029 | 0.948 | 3.146 | 0.299 | 0.279 | 5.876 | 0.119 |
| | 1 | AIPW | -0.5 | 0.236 | 0.063 | 0.012 | 1.006 | 0.001 | 0.953 | 3.005 | 0.949 | 3.123 | 0.950 | 3.077 | 0.248 | 0.183 | 4.016 | 0.080 |
| | 1 | WAIPW | -0.5 | 0.159 | 0.064 | 0.011 | 1.005 | 0.001 | 0.952 | 3.029 | 0.950 | 3.088 | 0.951 | 3.065 | 0.251 | 0.188 | 4.100 | 0.081 |
| TS(0.25) | 1 | IPW | -0.5 | 1.811 | 0.084 | 0.000 | 1.020 | 0.002 | 0.949 | 3.123 | 0.952 | 3.011 | 0.948 | 3.152 | 0.280 | 1.761 | 6.977 | 0.288 |
| | 1 | WIPW | -0.5 | 2.229 | 0.068 | 0.017 | 1.010 | 0.003 | 0.946 | 3.191 | 0.951 | 3.047 | 0.952 | 3.017 | 0.260 | 0.467 | 4.638 | 0.105 |
| | 1 | AIPW | -0.5 | 2.023 | 0.085 | 0.004 | 1.020 | 0.003 | 0.947 | 3.157 | 0.950 | 3.088 | 0.950 | 3.094 | 0.286 | 2.506 | 7.308 | 0.331 |
| | 1 | WAIPW | -0.5 | 2.342 | 0.069 | 0.018 | 1.017 | 0.003 | 0.945 | 3.230 | 0.948 | 3.146 | 0.949 | 3.106 | 0.262 | 0.650 | 4.758 | 0.111 |
| TS(0.50) | 1 | IPW | -0.5 | -2.455 | 0.084 | -0.063 | 1.015 | -0.002 | 0.947 | 3.163 | 0.955 | 2.944 | 0.941 | 3.338 | 0.299 | 1.699 | 7.092 | 0.254 |
| | 1 | WIPW | -0.5 | 0.816 | 0.071 | -0.022 | 1.005 | 0.001 | 0.951 | 3.053 | 0.952 | 3.011 | 0.945 | 3.230 | 0.274 | 0.668 | 5.077 | 0.110 |
| | 1 | AIPW | -0.5 | -2.252 | 0.087 | -0.057 | 1.023 | -0.002 | 0.945 | 3.224 | 0.951 | 3.065 | 0.940 | 3.364 | 0.303 | 2.301 | 7.556 | 0.307 |
| | 1 | WAIPW | -0.5 | 1.012 | 0.073 | -0.017 | 1.012 | 0.002 | 0.947 | 3.169 | 0.950 | 3.083 | 0.943 | 3.279 | 0.277 | 0.834 | 5.274 | 0.121 |
| TS(0.75) | 1 | IPW | -0.5 | -1.106 | 0.101 | -0.065 | 1.026 | -0.001 | 0.945 | 3.235 | 0.952 | 3.035 | 0.941 | 3.333 | 0.339 | 2.235 | 10.114 | 0.433 |
| | 1 | WIPW | -0.5 | 5.095 | 0.079 | 0.009 | 1.009 | 0.006 | 0.944 | 3.263 | 0.947 | 3.169 | 0.952 | 3.029 | 0.296 | 1.059 | 6.270 | 0.163 |
| | 1 | AIPW | -0.5 | -1.220 | 0.106 | -0.045 | 1.037 | -0.001 | 0.943 | 3.279 | 0.945 | 3.224 | 0.947 | 3.163 | 0.348 | 3.244 | 11.186 | 0.685 |
| | 1 | WAIPW | -0.5 | 4.757 | 0.081 | 0.016 | 1.023 | 0.005 | 0.943 | 3.284 | 0.941 | 3.338 | 0.952 | 3.035 | 0.303 | 1.345 | 6.630 | 0.176 |
| TS(1) | 1 | IPW | -0.5 | 0.138 | 0.122 | -0.066 | 1.062 | 0.001 | 0.938 | 3.416 | 0.944 | 3.257 | 0.940 | 3.364 | 0.387 | 2.803 | 14.845 | 0.577 |
| | 1 | WIPW | -0.5 | 6.294 | 0.090 | 0.003 | 1.031 | 0.007 | 0.945 | 3.213 | 0.939 | 3.385 | 0.951 | 3.059 | 0.320 | 1.406 | 8.099 | 0.233 |
| | 1 | AIPW | -0.5 | 0.904 | 0.135 | -0.037 | 1.063 | 0.001 | 0.938 | 3.411 | 0.938 | 3.421 | 0.950 | 3.094 | 0.405 | 4.768 | 18.184 | 1.924 |
| | 1 | WAIPW | -0.5 | 6.121 | 0.093 | 0.019 | 1.037 | 0.007 | 0.942 | 3.295 | 0.936 | 3.462 | 0.955 | 2.944 | 0.331 | 1.772 | 8.629 | 0.249 |
| WAIPW(0.5) | 1 | IPW | -0.5 | 1.314 | 0.071 | -0.042 | 1.015 | 0.002 | 0.947 | 3.163 | 0.948 | 3.135 | 0.945 | 3.235 | 0.272 | 0.535 | 5.021 | 0.105 |
| | 1 | WIPW | -0.5 | 5.466 | 0.069 | 0.022 | 1.016 | 0.006 | 0.946 | 3.208 | 0.945 | 3.219 | 0.949 | 3.123 | 0.266 | 0.466 | 4.821 | 0.100 |
| | 1 | AIPW | -0.5 | 1.354 | 0.070 | -0.038 | 1.020 | 0.002 | 0.945 | 3.230 | 0.946 | 3.186 | 0.945 | 3.230 | 0.269 | 0.526 | 4.930 | 0.103 |
| | 1 | WAIPW | -0.5 | 5.588 | 0.069 | 0.026 | 1.021 | 0.006 | 0.944 | 3.241 | 0.945 | 3.230 | 0.949 | 3.117 | 0.265 | 0.459 | 4.822 | 0.100 |
| WAIPW(1) | 1 | IPW | -0.5 | 1.882 | 0.096 | -0.095 | 1.038 | 0.002 | 0.941 | 3.322 | 0.951 | 3.041 | 0.937 | 3.431 | 0.350 | 1.738 | 9.286 | 0.253 |
| | 1 | WIPW | -0.5 | 11.202 | 0.080 | 0.039 | 1.025 | 0.012 | 0.941 | 3.327 | 0.941 | 3.322 | 0.948 | 3.140 | 0.299 | 0.972 | 6.560 | 0.164 |
| | 1 | AIPW | -0.5 | 1.973 | 0.095 | -0.080 | 1.037 | 0.002 | 0.943 | 3.279 | 0.949 | 3.123 | 0.939 | 3.385 | 0.349 | 1.825 | 9.054 | 0.241 |
| | 1 | WAIPW | -0.5 | 11.536 | 0.080 | 0.049 | 1.029 | 0.012 | 0.944 | 3.263 | 0.938 | 3.421 | 0.952 | 3.029 | 0.298 | 0.979 | 6.487 | 0.158 |
| WIPW(0.5) | 1 | IPW | -0.5 | 0.044 | 0.070 | -0.062 | 1.000 | 0.001 | 0.952 | 3.035 | 0.957 | 2.875 | 0.947 | 3.180 | 0.271 | 0.514 | 4.860 | 0.113 |
| | 1 | WIPW | -0.5 | 4.409 | 0.068 | 0.007 | 1.001 | 0.005 | 0.950 | 3.077 | 0.951 | 3.065 | 0.952 | 3.017 | 0.264 | 0.446 | 4.661 | 0.108 |
| | 1 | AIPW | -0.5 | -0.033 | 0.069 | -0.059 | 1.005 | 0.000 | 0.951 | 3.059 | 0.954 | 2.969 | 0.947 | 3.174 | 0.268 | 0.520 | 4.783 | 0.111 |
| | 1 | WAIPW | -0.5 | 4.210 | 0.068 | 0.008 | 1.007 | 0.005 | 0.946 | 3.202 | 0.948 | 3.140 | 0.952 | 3.035 | 0.264 | 0.457 | 4.672 | 0.107 |
| WIPW(1) | 1 | IPW | -0.5 | -2.122 | 0.095 | -0.151 | 1.036 | -0.002 | 0.938 | 3.411 | 0.953 | 2.993 | 0.928 | 3.656 | 0.345 | 1.683 | 8.973 | 0.235 |
| | 1 | WIPW | -0.5 | 7.906 | 0.078 | -0.011 | 1.018 | 0.008 | 0.947 | 3.157 | 0.948 | 3.129 | 0.946 | 3.208 | 0.296 | 0.949 | 6.224 | 0.152 |
| | 1 | AIPW | -0.5 | -2.003 | 0.094 | -0.135 | 1.031 | -0.002 | 0.944 | 3.263 | 0.951 | 3.053 | 0.933 | 3.536 | 0.346 | 1.823 | 8.872 | 0.230 |
| | 1 | WAIPW | -0.5 | 7.676 | 0.078 | -0.004 | 1.021 | 0.008 | 0.946 | 3.202 | 0.947 | 3.169 | 0.946 | 3.202 | 0.297 | 0.998 | 6.222 | 0.149 |
| AIPW(0.5) | 2 | IPW | -0.5 | 0.015 | 0.071 | -0.060 | 1.024 | 0.001 | 0.945 | 3.219 | 0.949 | 3.123 | 0.940 | 3.359 | 0.270 | 0.521 | 5.058 | 0.113 |

| Randomization Method | Regime | Estimator | True Value x 10^3 | Mean Estimate x 10^3 | SD Estimates x 10^3 | Mean Normalized | SD Normalized | Mean Bias | CI Coverage | SE CI Coverage x 10^3 | LB Coverage | SE LB Coverage x 10^3 | UB Coverage | SE UB Coverage x 10^3 | Mean CI Length | SE CI Length x 10^3 | MSE x 10^3 | SE MSE x 10^3 |
|---|---|---|---|---|---|---|---|---|---|---|---|---|---|---|---|---|---|---|
| | 2 | WIPW | -0.5 | 3.874 | 0.086 | 0.011 | 1.019 | 0.004 | 0.946 | 3.191 | 0.946 | 3.186 | 0.950 | 3.077 | 0.325 | 0.852 | 7.335 | 0.167 |
| | 2 | AIPW | -0.5 | 0.161 | 0.071 | -0.057 | 1.034 | 0.001 | 0.942 | 3.311 | 0.947 | 3.157 | 0.940 | 3.354 | 0.267 | 0.516 | 4.995 | 0.110 |
| | 2 | WAIPW | -0.5 | 4.081 | 0.070 | 0.004 | 1.036 | 0.005 | 0.942 | 3.311 | 0.941 | 3.333 | 0.947 | 3.169 | 0.264 | 0.454 | 4.899 | 0.106 |
| AIPW(1) | 2 | IPW | -0.5 | -0.805 | 0.096 | -0.126 | 1.023 | 0.000 | 0.942 | 3.311 | 0.955 | 2.926 | 0.935 | 3.487 | 0.352 | 1.700 | 9.171 | 0.256 |
| | 2 | WIPW | -0.5 | 9.130 | 0.111 | 0.007 | 1.030 | 0.010 | 0.942 | 3.306 | 0.945 | 3.235 | 0.949 | 3.117 | 0.402 | 2.123 | 12.424 | 0.358 |
| | 2 | AIPW | -0.5 | -0.566 | 0.096 | -0.112 | 1.027 | 0.000 | 0.941 | 3.343 | 0.952 | 3.017 | 0.937 | 3.436 | 0.352 | 1.792 | 9.157 | 0.253 |
| | 2 | WAIPW | -0.5 | 8.749 | 0.079 | 0.017 | 1.018 | 0.009 | 0.942 | 3.311 | 0.944 | 3.263 | 0.949 | 3.106 | 0.297 | 0.930 | 6.311 | 0.165 |
| AR-1 | 2 | IPW | -0.5 | -0.251 | 0.120 | -0.055 | 1.052 | 0.000 | 0.935 | 3.492 | 0.941 | 3.338 | 0.939 | 3.395 | 0.418 | 2.488 | 14.395 | 0.382 |
| | 2 | WIPW | -0.5 | 2.723 | 0.085 | -0.007 | 1.018 | 0.003 | 0.944 | 3.246 | 0.947 | 3.180 | 0.948 | 3.129 | 0.316 | 1.278 | 7.270 | 0.171 |
| | 2 | AIPW | -0.5 | -0.458 | 0.119 | -0.065 | 1.033 | 0.000 | 0.943 | 3.284 | 0.947 | 3.163 | 0.939 | 3.385 | 0.421 | 2.697 | 14.078 | 0.382 |
| | 2 | WAIPW | -0.5 | 0.406 | 0.089 | -0.050 | 1.013 | 0.001 | 0.948 | 3.152 | 0.951 | 3.053 | 0.944 | 3.252 | 0.335 | 1.548 | 7.992 | 0.190 |
| AR-2 | 2 | IPW | -0.5 | -3.283 | 0.104 | -0.096 | 1.034 | -0.003 | 0.946 | 3.202 | 0.951 | 3.041 | 0.937 | 3.441 | 0.368 | 2.095 | 10.739 | 0.284 |
| | 2 | WIPW | -0.5 | 3.084 | 0.079 | -0.015 | 1.017 | 0.004 | 0.948 | 3.135 | 0.946 | 3.191 | 0.945 | 3.230 | 0.296 | 1.085 | 6.242 | 0.143 |
| | 2 | AIPW | -0.5 | -3.396 | 0.103 | -0.094 | 1.026 | -0.003 | 0.946 | 3.191 | 0.954 | 2.969 | 0.937 | 3.426 | 0.368 | 2.200 | 10.641 | 0.281 |
| | 2 | WAIPW | -0.5 | 1.124 | 0.083 | -0.044 | 1.017 | 0.002 | 0.942 | 3.301 | 0.948 | 3.129 | 0.943 | 3.274 | 0.308 | 1.315 | 6.857 | 0.162 |
| IAIPW(0.5) | 2 | IPW | -0.5 | -0.315 | 0.070 | -0.064 | 1.010 | 0.000 | 0.947 | 3.180 | 0.955 | 2.938 | 0.942 | 3.306 | 0.271 | 0.531 | 4.894 | 0.102 |
| | 2 | WIPW | -0.5 | 4.000 | 0.068 | 0.003 | 1.008 | 0.004 | 0.947 | 3.180 | 0.949 | 3.100 | 0.948 | 3.146 | 0.264 | 0.463 | 4.651 | 0.097 |
| | 2 | AIPW | -0.5 | 0.037 | 0.069 | -0.057 | 1.015 | 0.001 | 0.944 | 3.246 | 0.955 | 2.944 | 0.942 | 3.306 | 0.268 | 0.517 | 4.799 | 0.099 |
| | 2 | WAIPW | -0.5 | 4.607 | 0.068 | 0.013 | 1.017 | 0.005 | 0.945 | 3.213 | 0.948 | 3.140 | 0.949 | 3.123 | 0.264 | 0.454 | 4.717 | 0.098 |
| IAIPW(1) | 2 | IPW | -0.5 | 1.677 | 0.099 | -0.106 | 1.047 | 0.002 | 0.940 | 3.354 | 0.948 | 3.146 | 0.940 | 3.369 | 0.353 | 1.717 | 9.864 | 0.265 |
| | 2 | WIPW | -0.5 | 9.895 | 0.081 | 0.019 | 1.025 | 0.010 | 0.944 | 3.263 | 0.941 | 3.322 | 0.953 | 3.005 | 0.298 | 0.938 | 6.601 | 0.169 |
| | 2 | AIPW | -0.5 | 2.179 | 0.099 | -0.088 | 1.058 | 0.003 | 0.938 | 3.411 | 0.943 | 3.284 | 0.945 | 3.224 | 0.353 | 1.834 | 9.855 | 0.264 |
| | 2 | WAIPW | -0.5 | 10.103 | 0.081 | 0.027 | 1.041 | 0.011 | 0.940 | 3.354 | 0.939 | 3.375 | 0.953 | 3.005 | 0.298 | 0.948 | 6.743 | 0.173 |
| IPW(0.5) | 2 | IPW | -0.5 | 1.374 | 0.069 | -0.038 | 0.996 | 0.002 | 0.950 | 3.077 | 0.952 | 3.011 | 0.945 | 3.224 | 0.271 | 0.512 | 4.822 | 0.112 |
| | 2 | WIPW | -0.5 | 5.449 | 0.084 | 0.034 | 1.005 | 0.006 | 0.946 | 3.208 | 0.943 | 3.268 | 0.951 | 3.059 | 0.325 | 0.845 | 7.137 | 0.166 |
| | 2 | AIPW | -0.5 | 1.563 | 0.069 | -0.032 | 1.002 | 0.002 | 0.945 | 3.219 | 0.950 | 3.077 | 0.948 | 3.152 | 0.268 | 0.517 | 4.723 | 0.109 |
| | 2 | WAIPW | -0.5 | 5.725 | 0.068 | 0.033 | 0.998 | 0.006 | 0.948 | 3.146 | 0.947 | 3.157 | 0.952 | 3.023 | 0.264 | 0.456 | 4.609 | 0.106 |
| IPW(1) | 2 | IPW | -0.5 | 0.400 | 0.098 | -0.124 | 1.049 | 0.001 | 0.937 | 3.426 | 0.951 | 3.059 | 0.933 | 3.536 | 0.351 | 1.649 | 9.574 | 0.285 |
| | 2 | WIPW | -0.5 | 10.634 | 0.116 | 0.011 | 1.063 | 0.011 | 0.937 | 3.441 | 0.939 | 3.380 | 0.940 | 3.369 | 0.401 | 2.056 | 13.586 | 0.435 |
| | 2 | AIPW | -0.5 | 0.336 | 0.098 | -0.110 | 1.054 | 0.001 | 0.934 | 3.502 | 0.945 | 3.235 | 0.938 | 3.406 | 0.351 | 1.778 | 9.541 | 0.279 |
| | 2 | WAIPW | -0.5 | 8.953 | 0.080 | 0.017 | 1.035 | 0.009 | 0.941 | 3.327 | 0.941 | 3.343 | 0.948 | 3.135 | 0.297 | 0.934 | 6.438 | 0.172 |
| SR | 2 | IPW | -0.5 | 0.879 | 0.064 | 0.022 | 1.007 | 0.001 | 0.952 | 3.011 | 0.949 | 3.100 | 0.951 | 3.053 | 0.251 | 0.185 | 4.136 | 0.083 |
| | 2 | WIPW | -0.5 | 1.187 | 0.076 | 0.022 | 1.001 | 0.002 | 0.951 | 3.059 | 0.942 | 3.301 | 0.951 | 3.047 | 0.299 | 0.277 | 5.792 | 0.115 |
| | 2 | AIPW | -0.5 | 0.796 | 0.064 | 0.020 | 1.012 | 0.001 | 0.952 | 3.029 | 0.948 | 3.129 | 0.951 | 3.047 | 0.248 | 0.184 | 4.055 | 0.083 |
| | 2 | WAIPW | -0.5 | 1.019 | 0.064 | 0.023 | 1.010 | 0.002 | 0.952 | 3.011 | 0.948 | 3.135 | 0.953 | 2.993 | 0.251 | 0.189 | 4.148 | 0.084 |
| TS(0.25) | 2 | IPW | -0.5 | -1.605 | 0.085 | -0.069 | 1.039 | -0.001 | 0.940 | 3.369 | 0.955 | 2.944 | 0.938 | 3.406 | 0.278 | 1.752 | 7.236 | 0.330 |
| | 2 | WIPW | -0.5 | 3.177 | 0.070 | -0.008 | 1.027 | 0.004 | 0.945 | 3.219 | 0.952 | 3.011 | 0.944 | 3.252 | 0.260 | 0.585 | 4.847 | 0.120 |
| | 2 | AIPW | -0.5 | -1.344 | 0.090 | -0.053 | 1.051 | -0.001 | 0.941 | 3.322 | 0.948 | 3.140 | 0.939 | 3.390 | 0.285 | 2.881 | 8.179 | 0.478 |
| | 2 | WAIPW | -0.5 | 3.077 | 0.071 | -0.007 | 1.039 | 0.004 | 0.942 | 3.317 | 0.946 | 3.186 | 0.945 | 3.219 | 0.261 | 0.740 | 4.997 | 0.130 |
| TS(0.50) | 2 | IPW | -0.5 | -0.626 | 0.091 | -0.098 | 1.016 | 0.000 | 0.948 | 3.146 | 0.955 | 2.932 | 0.944 | 3.246 | 0.300 | 1.817 | 8.225 | 0.466 |
| | 2 | WIPW | -0.5 | 7.099 | 0.074 | 0.007 | 1.002 | 0.008 | 0.951 | 3.059 | 0.949 | 3.112 | 0.956 | 2.913 | 0.274 | 0.966 | 5.518 | 0.157 |
| | 2 | AIPW | -0.5 | 0.091 | 0.100 | -0.081 | 1.035 | 0.001 | 0.948 | 3.135 | 0.944 | 3.241 | 0.946 | 3.197 | 0.308 | 3.090 | 9.947 | 0.967 |
| | 2 | WAIPW | -0.5 | 6.757 | 0.075 | 0.006 | 1.018 | 0.007 | 0.949 | 3.123 | 0.942 | 3.301 | 0.953 | 2.981 | 0.277 | 1.102 | 5.711 | 0.168 |
| TS(0.75) | 2 | IPW | -0.5 | -1.111 | 0.116 | -0.138 | 1.043 | -0.001 | 0.938 | 3.416 | 0.951 | 3.065 | 0.935 | 3.482 | 0.342 | 2.797 | 13.361 | 0.761 |

| Randomization Method | Regime | Estimator | True Value x 10^3 | Mean Estimate x 10^3 | SD Estimates x 10^3 | Mean Normalized | SD Normalized | Mean Bias | CI Coverage | SE CI Coverage x 10^3 | LB Coverage | SE LB Coverage x 10^3 | UB Coverage | SE UB Coverage x 10^3 | Mean CI Length | SE CI Length x 10^3 | MSE x 10^3 | SE MSE x 10^3 |
|---|---|---|---|---|---|---|---|---|---|---|---|---|---|---|---|---|---|---|
| | 2 | WIPW | -0.5 | 10.843 | 0.084 | 0.007 | 1.023 | 0.011 | 0.946 | 3.202 | 0.940 | 3.364 | 0.951 | 3.041 | 0.293 | 1.448 | 7.128 | 0.239 |
| | 2 | AIPW | -0.5 | 0.053 | 0.123 | -0.102 | 1.066 | 0.001 | 0.938 | 3.416 | 0.944 | 3.263 | 0.941 | 3.327 | 0.359 | 4.676 | 15.229 | 1.132 |
| | 2 | WAIPW | -0.5 | 10.164 | 0.086 | 0.009 | 1.047 | 0.011 | 0.937 | 3.436 | 0.936 | 3.452 | 0.948 | 3.140 | 0.298 | 1.716 | 7.436 | 0.239 |
| TS(1) | 2 | IPW | -0.5 | 3.588 | 0.143 | -0.110 | 1.121 | 0.004 | 0.934 | 3.512 | 0.939 | 3.375 | 0.936 | 3.452 | 0.391 | 3.431 | 20.407 | 1.197 |
| | 2 | WIPW | -0.5 | 16.300 | 0.094 | 0.041 | 1.044 | 0.017 | 0.946 | 3.202 | 0.934 | 3.502 | 0.952 | 3.029 | 0.316 | 1.839 | 9.196 | 0.335 |
| | 2 | AIPW | -0.5 | 1.219 | 0.336 | -0.044 | 1.159 | 0.002 | 0.926 | 3.702 | 0.925 | 3.721 | 0.944 | 3.246 | 0.448 | 17.904 | 112.816 | 57.321 |
| | 2 | WAIPW | -0.5 | 15.306 | 0.099 | 0.051 | 1.066 | 0.016 | 0.939 | 3.385 | 0.927 | 3.670 | 0.950 | 3.094 | 0.326 | 2.690 | 10.077 | 0.517 |
| WAIPW(0.5) | 2 | IPW | -0.5 | -0.265 | 0.071 | -0.066 | 1.015 | 0.000 | 0.944 | 3.241 | 0.953 | 2.981 | 0.938 | 3.416 | 0.271 | 0.537 | 4.986 | 0.110 |
| | 2 | WIPW | -0.5 | 4.123 | 0.069 | 0.001 | 1.016 | 0.005 | 0.946 | 3.208 | 0.947 | 3.169 | 0.945 | 3.213 | 0.265 | 0.467 | 4.762 | 0.104 |
| | 2 | AIPW | -0.5 | -0.037 | 0.070 | -0.059 | 1.026 | 0.000 | 0.944 | 3.257 | 0.950 | 3.071 | 0.940 | 3.369 | 0.268 | 0.527 | 4.912 | 0.106 |
| | 2 | WAIPW | -0.5 | 4.288 | 0.069 | 0.005 | 1.027 | 0.005 | 0.944 | 3.241 | 0.943 | 3.290 | 0.945 | 3.230 | 0.264 | 0.460 | 4.804 | 0.104 |
| WAIPW(1) | 2 | IPW | -0.5 | 2.142 | 0.097 | -0.093 | 1.041 | 0.003 | 0.939 | 3.395 | 0.951 | 3.065 | 0.935 | 3.477 | 0.351 | 1.776 | 9.439 | 0.264 |
| | 2 | WIPW | -0.5 | 11.479 | 0.080 | 0.038 | 1.017 | 0.012 | 0.948 | 3.146 | 0.947 | 3.180 | 0.951 | 3.041 | 0.299 | 0.991 | 6.465 | 0.160 |
| | 2 | AIPW | -0.5 | 2.012 | 0.096 | -0.080 | 1.047 | 0.003 | 0.940 | 3.359 | 0.945 | 3.230 | 0.938 | 3.421 | 0.350 | 1.864 | 9.211 | 0.246 |
| | 2 | WAIPW | -0.5 | 11.616 | 0.079 | 0.045 | 1.026 | 0.012 | 0.943 | 3.274 | 0.940 | 3.359 | 0.954 | 2.969 | 0.298 | 0.996 | 6.433 | 0.155 |
| WIPW(0.5) | 2 | IPW | -0.5 | -0.929 | 0.071 | -0.078 | 1.017 | 0.000 | 0.948 | 3.140 | 0.954 | 2.969 | 0.944 | 3.252 | 0.270 | 0.518 | 4.971 | 0.109 |
| | 2 | WIPW | -0.5 | 3.392 | 0.069 | -0.010 | 1.017 | 0.004 | 0.946 | 3.208 | 0.948 | 3.152 | 0.950 | 3.077 | 0.264 | 0.450 | 4.749 | 0.102 |
| | 2 | AIPW | -0.5 | -0.831 | 0.070 | -0.072 | 1.021 | 0.000 | 0.945 | 3.219 | 0.953 | 2.999 | 0.942 | 3.311 | 0.267 | 0.525 | 4.886 | 0.107 |
| | 2 | WAIPW | -0.5 | 3.280 | 0.069 | -0.008 | 1.024 | 0.004 | 0.945 | 3.213 | 0.945 | 3.235 | 0.947 | 3.169 | 0.264 | 0.461 | 4.790 | 0.103 |
| WIPW(1) | 2 | IPW | -0.5 | 1.259 | 0.097 | -0.114 | 1.068 | 0.002 | 0.932 | 3.556 | 0.950 | 3.077 | 0.925 | 3.734 | 0.345 | 1.693 | 9.480 | 0.253 |
| | 2 | WIPW | -0.5 | 10.259 | 0.080 | 0.018 | 1.040 | 0.011 | 0.940 | 3.369 | 0.943 | 3.279 | 0.946 | 3.197 | 0.296 | 0.952 | 6.517 | 0.165 |
| | 2 | AIPW | -0.5 | 0.989 | 0.097 | -0.099 | 1.071 | 0.001 | 0.933 | 3.531 | 0.948 | 3.140 | 0.927 | 3.675 | 0.346 | 1.840 | 9.382 | 0.250 |
| | 2 | WAIPW | -0.5 | 9.834 | 0.080 | 0.022 | 1.048 | 0.010 | 0.937 | 3.447 | 0.943 | 3.274 | 0.948 | 3.140 | 0.297 | 1.004 | 6.538 | 0.162 |
| AIPW(0.5) | 3 | IPW | -1.0 | -1.292 | 0.069 | -0.069 | 1.000 | 0.000 | 0.951 | 3.041 | 0.954 | 2.969 | 0.946 | 3.208 | 0.270 | 0.525 | 4.799 | 0.109 |
| | 3 | WIPW | -1.0 | 2.503 | 0.084 | 0.006 | 1.010 | 0.004 | 0.947 | 3.157 | 0.945 | 3.213 | 0.950 | 3.083 | 0.325 | 0.868 | 7.104 | 0.164 |
| | 3 | AIPW | -1.0 | -1.321 | 0.069 | -0.069 | 1.005 | 0.000 | 0.949 | 3.100 | 0.953 | 3.005 | 0.946 | 3.186 | 0.267 | 0.520 | 4.720 | 0.108 |
| | 3 | WAIPW | -1.0 | 2.755 | 0.067 | -0.006 | 1.000 | 0.004 | 0.951 | 3.065 | 0.951 | 3.059 | 0.952 | 3.023 | 0.263 | 0.456 | 4.563 | 0.103 |
| AIPW(1) | 3 | IPW | -1.0 | -1.284 | 0.095 | -0.125 | 1.025 | 0.000 | 0.944 | 3.257 | 0.954 | 2.969 | 0.935 | 3.492 | 0.352 | 1.694 | 9.007 | 0.236 |
| | 3 | WIPW | -1.0 | 8.921 | 0.110 | 0.011 | 1.028 | 0.010 | 0.943 | 3.279 | 0.940 | 3.369 | 0.952 | 3.035 | 0.401 | 2.097 | 12.284 | 0.345 |
| | 3 | AIPW | -1.0 | -1.257 | 0.095 | -0.117 | 1.020 | 0.000 | 0.948 | 3.146 | 0.955 | 2.920 | 0.938 | 3.406 | 0.352 | 1.801 | 8.935 | 0.238 |
| | 3 | WAIPW | -1.0 | 8.078 | 0.078 | 0.014 | 1.016 | 0.009 | 0.948 | 3.146 | 0.944 | 3.246 | 0.950 | 3.071 | 0.296 | 0.918 | 6.176 | 0.154 |
| AR-1 | 3 | IPW | -1.0 | -0.765 | 0.120 | -0.062 | 1.060 | 0.000 | 0.937 | 3.447 | 0.946 | 3.202 | 0.934 | 3.507 | 0.416 | 2.532 | 14.378 | 0.377 |
| | 3 | WIPW | -1.0 | 6.418 | 0.086 | 0.037 | 1.028 | 0.007 | 0.941 | 3.333 | 0.944 | 3.246 | 0.948 | 3.152 | 0.319 | 1.288 | 7.431 | 0.174 |
| | 3 | AIPW | -1.0 | -0.978 | 0.121 | -0.070 | 1.041 | 0.000 | 0.942 | 3.301 | 0.952 | 3.029 | 0.939 | 3.380 | 0.420 | 2.923 | 14.546 | 0.537 |
| | 3 | WAIPW | -1.0 | 5.372 | 0.090 | 0.013 | 1.031 | 0.006 | 0.940 | 3.369 | 0.949 | 3.112 | 0.946 | 3.202 | 0.334 | 1.569 | 8.216 | 0.201 |
| AR-2 | 3 | IPW | -1.0 | -2.365 | 0.101 | -0.081 | 1.016 | -0.001 | 0.946 | 3.186 | 0.952 | 3.035 | 0.945 | 3.235 | 0.369 | 2.101 | 10.223 | 0.269 |
| | 3 | WIPW | -1.0 | 5.207 | 0.077 | 0.017 | 0.997 | 0.006 | 0.951 | 3.065 | 0.947 | 3.174 | 0.955 | 2.944 | 0.297 | 1.066 | 5.999 | 0.140 |
| | 3 | AIPW | -1.0 | -2.504 | 0.101 | -0.085 | 1.005 | -0.002 | 0.951 | 3.065 | 0.953 | 2.981 | 0.947 | 3.163 | 0.370 | 2.236 | 10.287 | 0.279 |
| | 3 | WAIPW | -1.0 | 3.624 | 0.081 | -0.008 | 1.001 | 0.005 | 0.949 | 3.123 | 0.953 | 3.005 | 0.951 | 3.047 | 0.307 | 1.265 | 6.519 | 0.157 |
| IAIPW(0.5) | 3 | IPW | -1.0 | -0.717 | 0.070 | -0.063 | 1.005 | 0.000 | 0.949 | 3.106 | 0.956 | 2.913 | 0.944 | 3.257 | 0.271 | 0.534 | 4.895 | 0.109 |
| | 3 | WIPW | -1.0 | 3.867 | 0.068 | 0.008 | 1.005 | 0.005 | 0.948 | 3.135 | 0.947 | 3.169 | 0.950 | 3.077 | 0.264 | 0.465 | 4.682 | 0.104 |
| | 3 | AIPW | -1.0 | -0.460 | 0.069 | -0.059 | 1.011 | 0.001 | 0.946 | 3.202 | 0.952 | 3.029 | 0.944 | 3.241 | 0.268 | 0.527 | 4.829 | 0.108 |
| | 3 | WAIPW | -1.0 | 4.174 | 0.069 | 0.014 | 1.014 | 0.005 | 0.950 | 3.094 | 0.944 | 3.252 | 0.948 | 3.135 | 0.264 | 0.461 | 4.735 | 0.104 |
| IAIPW(1) | 3 | IPW | -1.0 | -0.129 | 0.096 | -0.115 | 1.035 | 0.001 | 0.941 | 3.322 | 0.951 | 3.047 | 0.935 | 3.477 | 0.353 | 1.668 | 9.240 | 0.249 |

| Randomization Method | Regime | Estimator | True Value x 10^3 | Mean Estimate x 10^3 | SD Estimates x 10^3 | Mean Normalized | SD Normalized | Mean Bias | CI Coverage | SE CI Coverage x 10^3 | LB Coverage | SE LB Coverage x 10^3 | UB Coverage x 10^3 | SE UB Coverage x 10^3 | Mean CI Length | SE CI Length x 10^3 | MSE x 10^3 | SE MSE x 10^3 |
|---|---|---|---|---|---|---|---|---|---|---|---|---|---|---|---|---|---|---|
| | 3 | WIPW | -1.0 | 9.384 | 0.079 | 0.021 | 1.017 | 0.010 | 0.946 | 3.197 | 0.942 | 3.306 | 0.951 | 3.065 | 0.298 | 0.910 | 6.354 | 0.156 |
| | 3 | AIPW | -1.0 | -0.061 | 0.096 | -0.104 | 1.033 | 0.001 | 0.942 | 3.295 | 0.951 | 3.059 | 0.937 | 3.431 | 0.352 | 1.792 | 9.218 | 0.249 |
| | 3 | WAIPW | -1.0 | 9.339 | 0.079 | 0.027 | 1.025 | 0.010 | 0.943 | 3.274 | 0.941 | 3.322 | 0.952 | 3.017 | 0.297 | 0.926 | 6.408 | 0.158 |
| IPW(0.5) | 3 | IPW | -1.0 | -0.583 | 0.069 | -0.059 | 0.993 | 0.000 | 0.950 | 3.083 | 0.953 | 2.987 | 0.950 | 3.083 | 0.270 | 0.509 | 4.706 | 0.098 |
| | 3 | WIPW | -1.0 | 3.272 | 0.084 | 0.013 | 1.005 | 0.004 | 0.949 | 3.100 | 0.949 | 3.100 | 0.953 | 3.005 | 0.324 | 0.844 | 7.041 | 0.151 |
| | 3 | AIPW | -1.0 | -0.502 | 0.068 | -0.055 | 1.000 | 0.000 | 0.950 | 3.077 | 0.955 | 2.932 | 0.946 | 3.186 | 0.267 | 0.521 | 4.642 | 0.097 |
| | 3 | WAIPW | -1.0 | 3.507 | 0.067 | 0.008 | 0.998 | 0.005 | 0.948 | 3.140 | 0.949 | 3.100 | 0.953 | 3.005 | 0.263 | 0.458 | 4.508 | 0.094 |
| IPW(1) | 3 | IPW | -1.0 | 1.090 | 0.096 | -0.102 | 1.028 | 0.002 | 0.941 | 3.338 | 0.951 | 3.047 | 0.940 | 3.348 | 0.349 | 1.623 | 9.184 | 0.267 |
| | 3 | WIPW | -1.0 | 11.198 | 0.112 | 0.029 | 1.038 | 0.012 | 0.941 | 3.322 | 0.939 | 3.380 | 0.948 | 3.140 | 0.400 | 2.043 | 12.778 | 0.393 |
| | 3 | AIPW | -1.0 | 0.842 | 0.096 | -0.092 | 1.027 | 0.002 | 0.946 | 3.208 | 0.945 | 3.219 | 0.943 | 3.290 | 0.350 | 1.769 | 9.213 | 0.265 |
| | 3 | WAIPW | -1.0 | 9.594 | 0.078 | 0.037 | 1.010 | 0.011 | 0.949 | 3.117 | 0.938 | 3.406 | 0.954 | 2.951 | 0.296 | 0.923 | 6.166 | 0.161 |
| SR | 3 | IPW | -1.0 | 0.462 | 0.063 | 0.023 | 0.982 | 0.001 | 0.952 | 3.017 | 0.949 | 3.106 | 0.953 | 2.981 | 0.251 | 0.186 | 3.920 | 0.078 |
| | 3 | WIPW | -1.0 | 0.364 | 0.076 | 0.018 | 0.996 | 0.001 | 0.949 | 3.123 | 0.944 | 3.257 | 0.955 | 2.944 | 0.299 | 0.278 | 5.706 | 0.115 |
| | 3 | AIPW | -1.0 | 0.305 | 0.062 | 0.021 | 0.986 | 0.001 | 0.952 | 3.017 | 0.952 | 3.029 | 0.953 | 2.987 | 0.248 | 0.183 | 3.860 | 0.078 |
| | 3 | WAIPW | -1.0 | 0.446 | 0.063 | 0.023 | 0.988 | 0.001 | 0.951 | 3.047 | 0.951 | 3.065 | 0.954 | 2.957 | 0.251 | 0.188 | 3.969 | 0.080 |
| TS(0.25) | 3 | IPW | -1.0 | -2.845 | 0.082 | -0.066 | 1.009 | -0.002 | 0.944 | 3.257 | 0.952 | 3.035 | 0.945 | 3.230 | 0.277 | 1.711 | 6.726 | 0.319 |
| | 3 | WIPW | -1.0 | -0.378 | 0.068 | -0.033 | 0.999 | 0.001 | 0.947 | 3.174 | 0.953 | 2.981 | 0.949 | 3.106 | 0.262 | 0.628 | 4.650 | 0.118 |
| | 3 | AIPW | -1.0 | -2.868 | 0.089 | -0.056 | 1.018 | -0.002 | 0.946 | 3.197 | 0.952 | 3.029 | 0.945 | 3.224 | 0.284 | 2.780 | 7.947 | 0.543 |
| | 3 | WAIPW | -1.0 | -0.675 | 0.071 | -0.033 | 1.009 | 0.000 | 0.949 | 3.123 | 0.950 | 3.094 | 0.948 | 3.140 | 0.265 | 1.076 | 5.079 | 0.196 |
| TS(0.50) | 3 | IPW | -1.0 | -0.912 | 0.093 | -0.082 | 1.031 | 0.000 | 0.941 | 3.333 | 0.950 | 3.083 | 0.940 | 3.348 | 0.301 | 1.984 | 8.685 | 0.539 |
| | 3 | WIPW | -1.0 | 2.300 | 0.077 | -0.036 | 1.022 | 0.003 | 0.943 | 3.290 | 0.950 | 3.088 | 0.943 | 3.268 | 0.283 | 1.116 | 6.006 | 0.178 |
| | 3 | AIPW | -1.0 | -0.410 | 0.104 | -0.064 | 1.038 | 0.001 | 0.940 | 3.359 | 0.945 | 3.224 | 0.942 | 3.311 | 0.310 | 3.441 | 10.829 | 1.050 |
| | 3 | WAIPW | -1.0 | 1.863 | 0.080 | -0.035 | 1.030 | 0.003 | 0.942 | 3.301 | 0.946 | 3.186 | 0.942 | 3.295 | 0.288 | 1.494 | 6.440 | 0.213 |
| TS(0.75) | 3 | IPW | -1.0 | 1.710 | 0.113 | -0.068 | 1.049 | 0.003 | 0.944 | 3.252 | 0.944 | 3.263 | 0.939 | 3.375 | 0.343 | 2.765 | 12.693 | 0.703 |
| | 3 | WIPW | -1.0 | 6.105 | 0.089 | -0.013 | 1.030 | 0.007 | 0.943 | 3.284 | 0.943 | 3.284 | 0.948 | 3.140 | 0.311 | 1.741 | 8.053 | 0.286 |
| | 3 | AIPW | -1.0 | 3.034 | 0.123 | -0.035 | 1.054 | 0.004 | 0.940 | 3.359 | 0.941 | 3.333 | 0.946 | 3.186 | 0.359 | 4.658 | 15.229 | 1.253 |
| | 3 | WAIPW | -1.0 | 5.605 | 0.099 | 0.001 | 1.050 | 0.007 | 0.939 | 3.380 | 0.936 | 3.472 | 0.949 | 3.123 | 0.327 | 2.856 | 9.866 | 0.459 |
| TS(1) | 3 | IPW | -1.0 | 1.878 | 0.139 | -0.092 | 1.114 | 0.003 | 0.934 | 3.517 | 0.940 | 3.359 | 0.931 | 3.594 | 0.393 | 3.449 | 19.391 | 1.063 |
| | 3 | WIPW | -1.0 | 8.257 | 0.110 | -0.025 | 1.063 | 0.009 | 0.937 | 3.436 | 0.937 | 3.447 | 0.938 | 3.416 | 0.346 | 2.303 | 12.187 | 0.610 |
| | 3 | AIPW | -1.0 | -0.625 | 0.334 | -0.030 | 1.100 | 0.000 | 0.935 | 3.487 | 0.930 | 3.618 | 0.943 | 3.274 | 0.450 | 17.788 | 111.758 | 58.824 |
| | 3 | WAIPW | -1.0 | 4.487 | 0.234 | 0.012 | 1.094 | 0.005 | 0.931 | 3.585 | 0.926 | 3.712 | 0.947 | 3.180 | 0.390 | 11.817 | 54.715 | 26.188 |
| WAIPW(0.5) | 3 | IPW | -1.0 | -1.602 | 0.070 | -0.072 | 1.003 | -0.001 | 0.947 | 3.180 | 0.955 | 2.932 | 0.943 | 3.290 | 0.271 | 0.526 | 4.867 | 0.107 |
| | 3 | WIPW | -1.0 | 2.869 | 0.068 | -0.004 | 1.001 | 0.004 | 0.949 | 3.123 | 0.950 | 3.094 | 0.947 | 3.157 | 0.265 | 0.461 | 4.632 | 0.100 |
| | 3 | AIPW | -1.0 | -1.225 | 0.069 | -0.065 | 1.007 | 0.000 | 0.946 | 3.202 | 0.954 | 2.963 | 0.944 | 3.263 | 0.268 | 0.518 | 4.754 | 0.103 |
| | 3 | WAIPW | -1.0 | 3.310 | 0.068 | 0.003 | 1.005 | 0.004 | 0.948 | 3.135 | 0.947 | 3.169 | 0.951 | 3.059 | 0.264 | 0.455 | 4.621 | 0.100 |
| WAIPW(1) | 3 | IPW | -1.0 | 1.523 | 0.095 | -0.095 | 1.022 | 0.003 | 0.945 | 3.219 | 0.952 | 3.017 | 0.941 | 3.322 | 0.348 | 1.725 | 8.988 | 0.248 |
| | 3 | WIPW | -1.0 | 9.995 | 0.078 | 0.031 | 1.002 | 0.011 | 0.949 | 3.106 | 0.945 | 3.230 | 0.958 | 2.843 | 0.298 | 0.967 | 6.216 | 0.157 |
| | 3 | AIPW | -1.0 | 1.346 | 0.094 | -0.086 | 1.019 | 0.002 | 0.947 | 3.169 | 0.950 | 3.088 | 0.944 | 3.257 | 0.348 | 1.833 | 8.791 | 0.241 |
| | 3 | WAIPW | -1.0 | 10.085 | 0.078 | 0.035 | 1.008 | 0.011 | 0.948 | 3.152 | 0.942 | 3.306 | 0.954 | 2.951 | 0.297 | 0.985 | 6.237 | 0.157 |
| WIPW(0.5) | 3 | IPW | -1.0 | -2.036 | 0.070 | -0.084 | 1.013 | -0.001 | 0.945 | 3.235 | 0.956 | 2.907 | 0.942 | 3.311 | 0.271 | 0.520 | 4.917 | 0.107 |
| | 3 | WIPW | -1.0 | 2.369 | 0.069 | -0.016 | 1.013 | 0.003 | 0.944 | 3.252 | 0.949 | 3.112 | 0.948 | 3.129 | 0.264 | 0.454 | 4.711 | 0.102 |
| | 3 | AIPW | -1.0 | -1.996 | 0.069 | -0.081 | 1.019 | -0.001 | 0.945 | 3.230 | 0.953 | 3.005 | 0.939 | 3.375 | 0.267 | 0.534 | 4.819 | 0.104 |
| | 3 | WAIPW | -1.0 | 2.221 | 0.069 | -0.014 | 1.021 | 0.003 | 0.943 | 3.268 | 0.947 | 3.180 | 0.947 | 3.163 | 0.263 | 0.469 | 4.711 | 0.101 |
| WIPW(1) | 3 | IPW | -1.0 | 1.568 | 0.095 | -0.107 | 1.037 | 0.003 | 0.944 | 3.257 | 0.954 | 2.969 | 0.931 | 3.594 | 0.345 | 1.675 | 9.101 | 0.252 |

| Randomization Method | Regime | Estimator | True Value x 10^3 | Mean Estimate x 10^3 | SD Estimates x 10^3 | Mean Normalized | SD Normalized | Mean Bias | CI Coverage | SE CI Coverage x 10^3 | LB Coverage | SE LB Coverage x 10^3 | UB Coverage | SE UB Coverage x 10^3 | Mean CI Length | SE CI Length x 10^3 | MSE x 10^3 | SE MSE x 10^3 |
|---|---|---|---|---|---|---|---|---|---|---|---|---|---|---|---|---|---|---|
| | 3 | WIPW | -1.0 | 10.370 | 0.078 | 0.028 | 1.015 | 0.011 | 0.946 | 3.208 | 0.947 | 3.174 | 0.950 | 3.088 | 0.296 | 0.940 | 6.270 | 0.160 |
| | 3 | AIPW | -1.0 | 1.331 | 0.094 | -0.095 | 1.031 | 0.002 | 0.943 | 3.274 | 0.952 | 3.017 | 0.935 | 3.477 | 0.345 | 1.794 | 8.913 | 0.240 |
| | 3 | WAIPW | -1.0 | 9.908 | 0.078 | 0.031 | 1.014 | 0.011 | 0.947 | 3.169 | 0.943 | 3.274 | 0.951 | 3.041 | 0.296 | 0.985 | 6.201 | 0.153 |
| AIPW(0.5) | 4 | IPW | -1.0 | -1.163 | 0.069 | -0.070 | 1.001 | 0.000 | 0.949 | 3.123 | 0.961 | 2.725 | 0.941 | 3.327 | 0.271 | 0.531 | 4.809 | 0.109 |
| | 4 | WIPW | -1.0 | 1.866 | 0.084 | -0.006 | 1.005 | 0.003 | 0.944 | 3.241 | 0.950 | 3.083 | 0.947 | 3.180 | 0.326 | 0.874 | 7.107 | 0.161 |
| | 4 | AIPW | -1.0 | -1.313 | 0.069 | -0.072 | 1.007 | 0.000 | 0.947 | 3.174 | 0.960 | 2.765 | 0.943 | 3.279 | 0.268 | 0.526 | 4.727 | 0.106 |
| | 4 | WAIPW | -1.0 | 2.797 | 0.068 | -0.008 | 1.006 | 0.004 | 0.949 | 3.100 | 0.956 | 2.907 | 0.951 | 3.059 | 0.264 | 0.458 | 4.581 | 0.102 |
| AIPW(1) | 4 | IPW | -1.0 | -1.988 | 0.094 | -0.132 | 1.021 | -0.001 | 0.942 | 3.317 | 0.956 | 2.888 | 0.936 | 3.472 | 0.350 | 1.653 | 8.866 | 0.227 |
| | 4 | WIPW | -1.0 | 8.060 | 0.110 | 0.005 | 1.021 | 0.009 | 0.943 | 3.268 | 0.945 | 3.230 | 0.950 | 3.088 | 0.399 | 2.094 | 12.179 | 0.337 |
| | 4 | AIPW | -1.0 | -2.131 | 0.093 | -0.128 | 1.016 | -0.001 | 0.943 | 3.279 | 0.953 | 2.987 | 0.938 | 3.416 | 0.349 | 1.751 | 8.666 | 0.219 |
| | 4 | WAIPW | -1.0 | 7.068 | 0.077 | -0.001 | 1.014 | 0.008 | 0.946 | 3.191 | 0.947 | 3.169 | 0.950 | 3.083 | 0.294 | 0.909 | 6.056 | 0.143 |
| AR-1 | 4 | IPW | -1.0 | -1.766 | 0.130 | 0.023 | 1.067 | -0.001 | 0.932 | 3.565 | 0.940 | 3.354 | 0.939 | 3.395 | 0.434 | 3.182 | 16.833 | 0.495 |
| | 4 | WIPW | -1.0 | -1.638 | 0.092 | 0.037 | 1.031 | -0.001 | 0.939 | 3.380 | 0.944 | 3.241 | 0.946 | 3.202 | 0.330 | 1.793 | 8.456 | 0.223 |
| | 4 | AIPW | -1.0 | -1.387 | 0.130 | 0.018 | 1.040 | 0.000 | 0.940 | 3.364 | 0.946 | 3.186 | 0.942 | 3.306 | 0.443 | 3.693 | 16.866 | 0.551 |
| | 4 | WAIPW | -1.0 | -1.313 | 0.100 | 0.033 | 1.038 | 0.000 | 0.942 | 3.311 | 0.945 | 3.219 | 0.943 | 3.279 | 0.353 | 2.323 | 9.981 | 0.297 |
| AR-2 | 4 | IPW | -1.0 | -0.941 | 0.107 | 0.028 | 1.037 | 0.000 | 0.941 | 3.333 | 0.942 | 3.311 | 0.947 | 3.169 | 0.377 | 2.423 | 11.344 | 0.323 |
| | 4 | WIPW | -1.0 | -0.147 | 0.079 | 0.038 | 1.008 | 0.001 | 0.946 | 3.197 | 0.948 | 3.152 | 0.952 | 3.035 | 0.300 | 1.287 | 6.304 | 0.152 |
| | 4 | AIPW | -1.0 | -0.941 | 0.106 | 0.027 | 1.024 | 0.000 | 0.942 | 3.317 | 0.945 | 3.219 | 0.948 | 3.140 | 0.379 | 2.579 | 11.211 | 0.325 |
| | 4 | WAIPW | -1.0 | -0.488 | 0.084 | 0.035 | 1.015 | 0.001 | 0.946 | 3.186 | 0.948 | 3.135 | 0.950 | 3.071 | 0.314 | 1.580 | 7.025 | 0.180 |
| IAIPW(0.5) | 4 | IPW | -1.0 | -1.619 | 0.070 | -0.080 | 1.014 | -0.001 | 0.948 | 3.135 | 0.956 | 2.913 | 0.940 | 3.348 | 0.271 | 0.539 | 4.939 | 0.103 |
| | 4 | WIPW | -1.0 | 2.997 | 0.069 | -0.009 | 1.016 | 0.004 | 0.948 | 3.140 | 0.948 | 3.146 | 0.948 | 3.152 | 0.264 | 0.472 | 4.746 | 0.100 |
| | 4 | AIPW | -1.0 | -1.683 | 0.070 | -0.080 | 1.021 | -0.001 | 0.947 | 3.163 | 0.953 | 2.993 | 0.943 | 3.290 | 0.268 | 0.527 | 4.846 | 0.101 |
| | 4 | WAIPW | -1.0 | 3.042 | 0.069 | -0.007 | 1.024 | 0.004 | 0.945 | 3.213 | 0.945 | 3.230 | 0.948 | 3.140 | 0.263 | 0.461 | 4.728 | 0.100 |
| IAIPW(1) | 4 | IPW | -1.0 | -1.339 | 0.095 | -0.124 | 1.025 | 0.000 | 0.941 | 3.338 | 0.955 | 2.926 | 0.937 | 3.431 | 0.353 | 1.655 | 8.969 | 0.236 |
| | 4 | WIPW | -1.0 | 7.794 | 0.077 | 0.009 | 0.998 | 0.009 | 0.949 | 3.123 | 0.953 | 2.999 | 0.952 | 3.017 | 0.297 | 0.898 | 5.965 | 0.146 |
| | 4 | AIPW | -1.0 | -1.328 | 0.094 | -0.117 | 1.016 | 0.000 | 0.948 | 3.135 | 0.956 | 2.913 | 0.943 | 3.284 | 0.353 | 1.775 | 8.848 | 0.232 |
| | 4 | WAIPW | -1.0 | 7.900 | 0.077 | 0.014 | 1.001 | 0.009 | 0.949 | 3.106 | 0.949 | 3.100 | 0.953 | 3.005 | 0.297 | 0.911 | 5.961 | 0.144 |
| IPW(0.5) | 4 | IPW | -1.0 | -0.205 | 0.070 | -0.058 | 1.017 | 0.001 | 0.948 | 3.152 | 0.948 | 3.129 | 0.945 | 3.213 | 0.271 | 0.508 | 4.934 | 0.106 |
| | 4 | WIPW | -1.0 | 3.311 | 0.085 | 0.006 | 1.018 | 0.004 | 0.946 | 3.191 | 0.948 | 3.152 | 0.950 | 3.094 | 0.325 | 0.843 | 7.185 | 0.153 |
| | 4 | AIPW | -1.0 | -0.278 | 0.070 | -0.056 | 1.023 | 0.001 | 0.943 | 3.290 | 0.948 | 3.129 | 0.943 | 3.284 | 0.268 | 0.520 | 4.852 | 0.103 |
| | 4 | WAIPW | -1.0 | 3.913 | 0.069 | 0.010 | 1.024 | 0.005 | 0.940 | 3.348 | 0.943 | 3.279 | 0.946 | 3.197 | 0.263 | 0.457 | 4.742 | 0.101 |
| IPW(1) | 4 | IPW | -1.0 | 1.080 | 0.096 | -0.104 | 1.035 | 0.002 | 0.940 | 3.354 | 0.951 | 3.065 | 0.938 | 3.411 | 0.349 | 1.611 | 9.221 | 0.243 |
| | 4 | WIPW | -1.0 | 11.003 | 0.112 | 0.026 | 1.047 | 0.012 | 0.941 | 3.338 | 0.937 | 3.426 | 0.946 | 3.208 | 0.400 | 2.028 | 12.788 | 0.346 |
| | 4 | AIPW | -1.0 | 0.878 | 0.096 | -0.099 | 1.025 | 0.002 | 0.946 | 3.191 | 0.954 | 2.969 | 0.940 | 3.359 | 0.350 | 1.772 | 9.166 | 0.240 |
| | 4 | WAIPW | -1.0 | 9.432 | 0.078 | 0.028 | 1.014 | 0.010 | 0.948 | 3.152 | 0.946 | 3.208 | 0.949 | 3.100 | 0.296 | 0.932 | 6.183 | 0.147 |
| SR | 4 | IPW | -1.0 | -0.333 | 0.064 | 0.011 | 0.997 | 0.001 | 0.954 | 2.951 | 0.952 | 3.017 | 0.950 | 3.088 | 0.251 | 0.185 | 4.052 | 0.081 |
| | 4 | WIPW | -1.0 | -1.250 | 0.076 | -0.002 | 0.999 | 0.000 | 0.954 | 2.957 | 0.954 | 2.951 | 0.948 | 3.140 | 0.299 | 0.276 | 5.726 | 0.112 |
| | 4 | AIPW | -1.0 | -0.255 | 0.063 | 0.013 | 1.000 | 0.001 | 0.952 | 3.023 | 0.951 | 3.059 | 0.950 | 3.077 | 0.248 | 0.183 | 3.972 | 0.079 |
| | 4 | WAIPW | -1.0 | -0.323 | 0.064 | 0.012 | 0.999 | 0.001 | 0.954 | 2.975 | 0.948 | 3.152 | 0.951 | 3.041 | 0.251 | 0.187 | 4.043 | 0.079 |
| TS(0.25) | 4 | IPW | -1.0 | 0.661 | 0.082 | -0.022 | 1.020 | 0.002 | 0.944 | 3.241 | 0.951 | 3.059 | 0.943 | 3.274 | 0.279 | 1.747 | 6.736 | 0.298 |
| | 4 | WIPW | -1.0 | -0.503 | 0.069 | -0.026 | 1.017 | 0.000 | 0.944 | 3.241 | 0.952 | 3.029 | 0.943 | 3.274 | 0.261 | 0.537 | 4.753 | 0.111 |
| | 4 | AIPW | -1.0 | 0.498 | 0.085 | -0.025 | 1.021 | 0.001 | 0.947 | 3.174 | 0.949 | 3.117 | 0.944 | 3.263 | 0.283 | 2.411 | 7.166 | 0.374 |
| | 4 | WAIPW | -1.0 | -0.634 | 0.069 | -0.028 | 1.023 | 0.000 | 0.945 | 3.235 | 0.948 | 3.129 | 0.940 | 3.354 | 0.261 | 0.669 | 4.795 | 0.115 |
| TS(0.50) | 4 | IPW | -1.0 | -2.740 | 0.087 | -0.086 | 1.009 | -0.002 | 0.951 | 3.065 | 0.956 | 2.907 | 0.937 | 3.441 | 0.298 | 1.848 | 7.538 | 0.383 |

| Randomization Method | Regime | Estimator | True Value x 10^3 | Mean Estimate x 10^3 | SD Estimates x 10^3 | Mean Normalized | SD Normalized | Mean Bias | CI Coverage | SE CI Coverage x 10^3 | LB Coverage | SE LB Coverage x 10^3 | UB Coverage x 10^3 | SE UB Coverage x 10^3 | Mean CI Length | SE CI Length x 10^3 | MSE x 10^3 | SE MSE x 10^3 |
|---|---|---|---|---|---|---|---|---|---|---|---|---|---|---|---|---|---|---|
| | 4 | WIPW | -1.0 | -2.436 | 0.073 | -0.085 | 1.008 | -0.001 | 0.946 | 3.186 | 0.958 | 2.843 | 0.940 | 3.359 | 0.276 | 0.777 | 5.330 | 0.133 |
| | 4 | AIPW | -1.0 | -2.752 | 0.091 | -0.083 | 1.011 | -0.002 | 0.946 | 3.197 | 0.959 | 2.811 | 0.937 | 3.431 | 0.303 | 2.808 | 8.310 | 0.602 |
| | 4 | WAIPW | -1.0 | -2.355 | 0.073 | -0.082 | 1.015 | -0.001 | 0.944 | 3.252 | 0.956 | 2.901 | 0.939 | 3.395 | 0.277 | 0.893 | 5.369 | 0.132 |
| TS(0.75) | 4 | IPW | -1.0 | 2.380 | 0.104 | -0.045 | 1.036 | 0.003 | 0.942 | 3.317 | 0.944 | 3.246 | 0.942 | 3.311 | 0.337 | 2.272 | 10.794 | 0.549 |
| | 4 | WIPW | -1.0 | 3.142 | 0.084 | -0.034 | 1.032 | 0.004 | 0.940 | 3.354 | 0.945 | 3.230 | 0.941 | 3.327 | 0.303 | 1.234 | 7.155 | 0.211 |
| | 4 | AIPW | -1.0 | 1.760 | 0.108 | -0.042 | 1.033 | 0.003 | 0.944 | 3.257 | 0.947 | 3.169 | 0.941 | 3.338 | 0.345 | 3.280 | 11.728 | 0.894 |
| | 4 | WAIPW | -1.0 | 2.886 | 0.085 | -0.029 | 1.045 | 0.004 | 0.936 | 3.467 | 0.942 | 3.311 | 0.940 | 3.359 | 0.306 | 1.457 | 7.303 | 0.213 |
| TS(1) | 4 | IPW | -1.0 | -0.562 | 0.122 | -0.096 | 1.053 | 0.000 | 0.937 | 3.426 | 0.950 | 3.083 | 0.936 | 3.472 | 0.385 | 2.961 | 14.867 | 0.630 |
| | 4 | WIPW | -1.0 | 0.617 | 0.096 | -0.098 | 1.044 | 0.002 | 0.936 | 3.467 | 0.947 | 3.157 | 0.933 | 3.541 | 0.331 | 1.687 | 9.275 | 0.293 |
| | 4 | AIPW | -1.0 | -0.940 | 0.130 | -0.086 | 1.035 | 0.000 | 0.944 | 3.252 | 0.949 | 3.100 | 0.941 | 3.327 | 0.400 | 4.448 | 16.830 | 1.169 |
| | 4 | WAIPW | -1.0 | 0.264 | 0.098 | -0.090 | 1.052 | 0.001 | 0.933 | 3.546 | 0.943 | 3.290 | 0.934 | 3.512 | 0.338 | 2.036 | 9.683 | 0.338 |
| WAIPW(0.5) | 4 | IPW | -1.0 | -0.003 | 0.071 | -0.053 | 1.014 | 0.001 | 0.949 | 3.123 | 0.952 | 3.017 | 0.949 | 3.100 | 0.272 | 0.537 | 5.002 | 0.111 |
| | 4 | WIPW | -1.0 | 4.499 | 0.069 | 0.016 | 1.012 | 0.005 | 0.947 | 3.174 | 0.946 | 3.202 | 0.952 | 3.035 | 0.265 | 0.470 | 4.770 | 0.105 |
| | 4 | AIPW | -1.0 | 0.166 | 0.070 | -0.050 | 1.018 | 0.001 | 0.945 | 3.213 | 0.950 | 3.083 | 0.946 | 3.202 | 0.269 | 0.535 | 4.892 | 0.108 |
| | 4 | WAIPW | -1.0 | 4.914 | 0.069 | 0.022 | 1.016 | 0.006 | 0.944 | 3.241 | 0.944 | 3.246 | 0.952 | 3.023 | 0.264 | 0.465 | 4.749 | 0.105 |
| WAIPW(1) | 4 | IPW | -1.0 | 1.451 | 0.098 | -0.100 | 1.045 | 0.002 | 0.941 | 3.343 | 0.948 | 3.129 | 0.938 | 3.401 | 0.347 | 1.730 | 9.562 | 0.290 |
| | 4 | WIPW | -1.0 | 9.699 | 0.080 | 0.024 | 1.019 | 0.011 | 0.944 | 3.246 | 0.941 | 3.333 | 0.953 | 2.981 | 0.297 | 0.969 | 6.497 | 0.171 |
| | 4 | AIPW | -1.0 | 1.307 | 0.096 | -0.095 | 1.035 | 0.002 | 0.942 | 3.306 | 0.949 | 3.106 | 0.944 | 3.263 | 0.346 | 1.822 | 9.298 | 0.276 |
| | 4 | WAIPW | -1.0 | 9.746 | 0.079 | 0.028 | 1.023 | 0.011 | 0.942 | 3.311 | 0.938 | 3.401 | 0.953 | 2.993 | 0.296 | 0.980 | 6.431 | 0.165 |
| WIPW(0.5) | 4 | IPW | -1.0 | -0.956 | 0.070 | -0.068 | 1.007 | 0.000 | 0.948 | 3.146 | 0.956 | 2.913 | 0.942 | 3.317 | 0.271 | 0.517 | 4.889 | 0.109 |
| | 4 | WIPW | -1.0 | 3.368 | 0.068 | -0.001 | 1.006 | 0.004 | 0.947 | 3.180 | 0.952 | 3.017 | 0.949 | 3.123 | 0.264 | 0.450 | 4.666 | 0.104 |
| | 4 | AIPW | -1.0 | -1.197 | 0.069 | -0.067 | 1.015 | 0.000 | 0.945 | 3.230 | 0.953 | 2.981 | 0.942 | 3.295 | 0.268 | 0.526 | 4.789 | 0.105 |
| | 4 | WAIPW | -1.0 | 3.108 | 0.068 | 0.000 | 1.016 | 0.004 | 0.944 | 3.241 | 0.949 | 3.112 | 0.947 | 3.163 | 0.263 | 0.460 | 4.654 | 0.102 |
| WIPW(1) | 4 | IPW | -1.0 | -1.757 | 0.092 | -0.136 | 1.005 | -0.001 | 0.945 | 3.224 | 0.957 | 2.869 | 0.940 | 3.364 | 0.346 | 1.657 | 8.523 | 0.226 |
| | 4 | WIPW | -1.0 | 7.791 | 0.077 | 0.001 | 0.990 | 0.009 | 0.951 | 3.053 | 0.950 | 3.088 | 0.951 | 3.065 | 0.297 | 0.928 | 5.986 | 0.147 |
| | 4 | AIPW | -1.0 | -1.661 | 0.092 | -0.125 | 1.003 | -0.001 | 0.946 | 3.186 | 0.957 | 2.856 | 0.942 | 3.306 | 0.345 | 1.765 | 8.421 | 0.227 |
| | 4 | WAIPW | -1.0 | 7.528 | 0.077 | 0.003 | 0.997 | 0.009 | 0.947 | 3.163 | 0.949 | 3.112 | 0.950 | 3.071 | 0.296 | 0.966 | 5.980 | 0.145 |
| AIPW(0.5) | 5 | IPW | -2.1 | 0.233 | 0.071 | -0.034 | 1.021 | 0.002 | 0.948 | 3.146 | 0.949 | 3.117 | 0.943 | 3.274 | 0.271 | 0.525 | 4.995 | 0.103 |
| | 5 | WIPW | -2.1 | 4.193 | 0.084 | 0.032 | 1.009 | 0.006 | 0.949 | 3.112 | 0.944 | 3.246 | 0.951 | 3.041 | 0.326 | 0.861 | 7.174 | 0.151 |
| | 5 | AIPW | -2.1 | 0.251 | 0.070 | -0.032 | 1.025 | 0.002 | 0.946 | 3.208 | 0.948 | 3.129 | 0.942 | 3.306 | 0.268 | 0.517 | 4.889 | 0.101 |
| | 5 | WAIPW | -2.1 | 4.226 | 0.069 | 0.030 | 1.027 | 0.006 | 0.944 | 3.263 | 0.941 | 3.322 | 0.951 | 3.065 | 0.264 | 0.453 | 4.806 | 0.101 |
| AIPW(1) | 5 | IPW | -2.1 | -2.841 | 0.097 | -0.136 | 1.046 | -0.001 | 0.934 | 3.502 | 0.953 | 2.993 | 0.932 | 3.556 | 0.350 | 1.671 | 9.355 | 0.249 |
| | 5 | WIPW | -2.1 | 7.280 | 0.113 | -0.001 | 1.052 | 0.009 | 0.936 | 3.452 | 0.939 | 3.375 | 0.941 | 3.338 | 0.399 | 2.080 | 12.762 | 0.361 |
| | 5 | AIPW | -2.1 | -2.634 | 0.096 | -0.124 | 1.040 | -0.001 | 0.936 | 3.452 | 0.952 | 3.023 | 0.937 | 3.447 | 0.349 | 1.779 | 9.243 | 0.248 |
| | 5 | WAIPW | -2.1 | 5.910 | 0.079 | -0.003 | 1.036 | 0.008 | 0.941 | 3.338 | 0.944 | 3.257 | 0.947 | 3.169 | 0.295 | 0.926 | 6.350 | 0.156 |
| AR-1 | 5 | IPW | -2.1 | -1.738 | 0.113 | 0.003 | 1.041 | 0.000 | 0.940 | 3.354 | 0.942 | 3.306 | 0.946 | 3.202 | 0.382 | 2.739 | 12.753 | 0.388 |
| | 5 | WIPW | -2.1 | 0.172 | 0.082 | 0.033 | 1.014 | 0.002 | 0.946 | 3.197 | 0.946 | 3.191 | 0.952 | 3.017 | 0.301 | 1.503 | 6.736 | 0.175 |
| | 5 | AIPW | -2.1 | -1.776 | 0.114 | -0.006 | 1.023 | 0.000 | 0.945 | 3.219 | 0.948 | 3.146 | 0.944 | 3.246 | 0.387 | 3.082 | 12.937 | 0.437 |
| | 5 | WAIPW | -2.1 | 0.045 | 0.089 | 0.021 | 1.021 | 0.002 | 0.947 | 3.157 | 0.947 | 3.169 | 0.948 | 3.135 | 0.318 | 1.950 | 7.861 | 0.224 |
| AR-2 | 5 | IPW | -2.1 | -3.441 | 0.092 | -0.030 | 1.031 | -0.001 | 0.943 | 3.268 | 0.948 | 3.140 | 0.941 | 3.322 | 0.328 | 1.996 | 8.496 | 0.245 |
| | 5 | WIPW | -2.1 | -1.722 | 0.072 | -0.004 | 1.000 | 0.000 | 0.949 | 3.117 | 0.951 | 3.053 | 0.950 | 3.083 | 0.276 | 1.087 | 5.181 | 0.124 |
| | 5 | AIPW | -2.1 | -3.990 | 0.091 | -0.035 | 1.016 | -0.002 | 0.948 | 3.152 | 0.951 | 3.047 | 0.945 | 3.213 | 0.328 | 2.105 | 8.340 | 0.238 |
| | 5 | WAIPW | -2.1 | -2.415 | 0.075 | -0.013 | 0.998 | 0.000 | 0.949 | 3.100 | 0.949 | 3.100 | 0.949 | 3.112 | 0.285 | 1.288 | 5.574 | 0.138 |
| IAIPW(0.5) | 5 | IPW | -2.1 | -1.357 | 0.071 | -0.061 | 1.021 | 0.001 | 0.945 | 3.224 | 0.951 | 3.065 | 0.942 | 3.295 | 0.271 | 0.527 | 5.027 | 0.108 |

| Randomization Method | Regime | Estimator | True Value x 10^3 | Mean Estimate x 10^3 | SD Estimates x 10^3 | Mean Normalized | SD Normalized | Mean Bias | CI Coverage | SE CI Coverage x 10^3 | LB Coverage | SE LB Coverage x 10^3 | UB Coverage | SE UB Coverage x 10^3 | Mean CI Length | SE CI Length x 10^3 | MSE x 10^3 | SE MSE x 10^3 |
|---|---|---|---|---|---|---|---|---|---|---|---|---|---|---|---|---|---|---|
| | 5 | WIPW | -2.1 | 3.127 | 0.069 | 0.010 | 1.022 | 0.005 | 0.945 | 3.235 | 0.945 | 3.230 | 0.947 | 3.163 | 0.264 | 0.459 | 4.823 | 0.103 |
| | 5 | AIPW | -2.1 | -1.234 | 0.070 | -0.056 | 1.026 | 0.001 | 0.944 | 3.241 | 0.953 | 2.999 | 0.939 | 3.385 | 0.267 | 0.518 | 4.903 | 0.104 |
| | 5 | WAIPW | -2.1 | 2.953 | 0.069 | 0.010 | 1.026 | 0.005 | 0.942 | 3.306 | 0.946 | 3.202 | 0.945 | 3.219 | 0.263 | 0.454 | 4.787 | 0.101 |
| IAIPW(1) | 5 | IPW | -2.1 | -0.019 | 0.098 | -0.107 | 1.055 | 0.002 | 0.935 | 3.492 | 0.947 | 3.174 | 0.932 | 3.565 | 0.349 | 1.655 | 9.547 | 0.259 |
| | 5 | WIPW | -2.1 | 7.330 | 0.080 | 0.011 | 1.034 | 0.009 | 0.941 | 3.338 | 0.941 | 3.322 | 0.946 | 3.191 | 0.295 | 0.910 | 6.472 | 0.163 |
| | 5 | AIPW | -2.1 | 0.073 | 0.097 | -0.097 | 1.045 | 0.002 | 0.934 | 3.507 | 0.946 | 3.202 | 0.937 | 3.436 | 0.350 | 1.769 | 9.336 | 0.250 |
| | 5 | WAIPW | -2.1 | 7.340 | 0.080 | 0.015 | 1.041 | 0.009 | 0.936 | 3.457 | 0.939 | 3.390 | 0.946 | 3.208 | 0.295 | 0.926 | 6.502 | 0.160 |
| IPW(0.5) | 5 | IPW | -2.1 | -2.340 | 0.069 | -0.072 | 1.007 | 0.000 | 0.946 | 3.208 | 0.955 | 2.944 | 0.942 | 3.311 | 0.270 | 0.517 | 4.789 | 0.100 |
| | 5 | WIPW | -2.1 | 1.303 | 0.084 | 0.001 | 1.009 | 0.003 | 0.951 | 3.053 | 0.951 | 3.053 | 0.950 | 3.083 | 0.323 | 0.856 | 7.079 | 0.152 |
| | 5 | AIPW | -2.1 | -2.450 | 0.069 | -0.069 | 1.012 | 0.000 | 0.945 | 3.213 | 0.952 | 3.017 | 0.942 | 3.317 | 0.266 | 0.528 | 4.704 | 0.098 |
| | 5 | WAIPW | -2.1 | 1.920 | 0.067 | -0.001 | 1.009 | 0.004 | 0.947 | 3.157 | 0.947 | 3.169 | 0.949 | 3.106 | 0.262 | 0.464 | 4.568 | 0.096 |
| IPW(1) | 5 | IPW | -2.1 | -1.352 | 0.094 | -0.124 | 1.027 | 0.001 | 0.941 | 3.343 | 0.952 | 3.017 | 0.935 | 3.482 | 0.343 | 1.595 | 8.819 | 0.236 |
| | 5 | WIPW | -2.1 | 10.274 | 0.109 | 0.032 | 1.025 | 0.012 | 0.942 | 3.295 | 0.943 | 3.284 | 0.949 | 3.117 | 0.393 | 2.014 | 12.112 | 0.338 |
| | 5 | AIPW | -2.1 | -1.133 | 0.094 | -0.109 | 1.024 | 0.001 | 0.944 | 3.246 | 0.952 | 3.029 | 0.939 | 3.380 | 0.344 | 1.735 | 8.807 | 0.241 |
| | 5 | WAIPW | -2.1 | 7.080 | 0.078 | 0.015 | 1.013 | 0.009 | 0.947 | 3.157 | 0.943 | 3.279 | 0.952 | 3.017 | 0.293 | 0.922 | 6.158 | 0.153 |
| SR | 5 | IPW | -2.1 | -2.566 | 0.065 | -0.006 | 1.016 | 0.000 | 0.944 | 3.263 | 0.950 | 3.077 | 0.943 | 3.274 | 0.251 | 0.188 | 4.207 | 0.085 |
| | 5 | WIPW | -2.1 | -2.175 | 0.078 | 0.000 | 1.027 | 0.000 | 0.944 | 3.263 | 0.945 | 3.224 | 0.944 | 3.263 | 0.299 | 0.279 | 6.097 | 0.124 |
| | 5 | AIPW | -2.1 | -2.517 | 0.064 | -0.007 | 1.022 | 0.000 | 0.941 | 3.343 | 0.951 | 3.059 | 0.944 | 3.252 | 0.248 | 0.183 | 4.146 | 0.084 |
| | 5 | WAIPW | -2.1 | -2.572 | 0.065 | -0.008 | 1.024 | 0.000 | 0.941 | 3.327 | 0.950 | 3.077 | 0.943 | 3.290 | 0.251 | 0.187 | 4.265 | 0.086 |
| TS(0.25) | 5 | IPW | -2.1 | -1.910 | 0.081 | -0.011 | 1.009 | 0.000 | 0.948 | 3.140 | 0.949 | 3.117 | 0.949 | 3.123 | 0.279 | 1.655 | 6.553 | 0.272 |
| | 5 | WIPW | -2.1 | -2.177 | 0.067 | -0.019 | 1.000 | 0.000 | 0.950 | 3.077 | 0.949 | 3.123 | 0.949 | 3.117 | 0.261 | 0.475 | 4.525 | 0.096 |
| | 5 | AIPW | -2.1 | -1.786 | 0.085 | -0.006 | 1.010 | 0.000 | 0.950 | 3.088 | 0.948 | 3.152 | 0.949 | 3.117 | 0.285 | 2.437 | 7.205 | 0.400 |
| | 5 | WAIPW | -2.1 | -1.993 | 0.068 | -0.014 | 1.005 | 0.000 | 0.949 | 3.117 | 0.947 | 3.174 | 0.947 | 3.169 | 0.263 | 0.654 | 4.611 | 0.103 |
| TS(0.50) | 5 | IPW | -2.1 | -1.806 | 0.088 | -0.038 | 1.015 | 0.000 | 0.946 | 3.202 | 0.950 | 3.077 | 0.945 | 3.235 | 0.301 | 1.803 | 7.749 | 0.316 |
| | 5 | WIPW | -2.1 | -2.158 | 0.073 | -0.042 | 1.010 | 0.000 | 0.947 | 3.174 | 0.952 | 3.011 | 0.947 | 3.174 | 0.279 | 0.813 | 5.365 | 0.129 |
| | 5 | AIPW | -2.1 | -2.124 | 0.091 | -0.035 | 1.014 | 0.000 | 0.949 | 3.106 | 0.950 | 3.083 | 0.949 | 3.123 | 0.306 | 2.585 | 8.365 | 0.412 |
| | 5 | WAIPW | -2.1 | -2.738 | 0.075 | -0.042 | 1.016 | -0.001 | 0.946 | 3.191 | 0.950 | 3.071 | 0.948 | 3.129 | 0.281 | 1.164 | 5.618 | 0.171 |
| TS(0.75) | 5 | IPW | -2.1 | -1.837 | 0.102 | -0.050 | 1.043 | 0.000 | 0.943 | 3.274 | 0.949 | 3.106 | 0.941 | 3.322 | 0.342 | 2.154 | 10.409 | 0.383 |
| | 5 | WIPW | -2.1 | -2.613 | 0.086 | -0.069 | 1.032 | -0.001 | 0.941 | 3.327 | 0.953 | 2.993 | 0.941 | 3.327 | 0.309 | 1.319 | 7.352 | 0.213 |
| | 5 | AIPW | -2.1 | -2.062 | 0.110 | -0.041 | 1.043 | 0.000 | 0.942 | 3.295 | 0.945 | 3.224 | 0.945 | 3.235 | 0.352 | 3.268 | 12.070 | 0.830 |
| | 5 | WAIPW | -2.1 | -3.399 | 0.089 | -0.059 | 1.040 | -0.001 | 0.942 | 3.311 | 0.945 | 3.219 | 0.942 | 3.311 | 0.316 | 1.826 | 7.897 | 0.267 |
| TS(1) | 5 | IPW | -2.1 | -2.394 | 0.120 | -0.070 | 1.058 | 0.000 | 0.940 | 3.369 | 0.946 | 3.202 | 0.935 | 3.482 | 0.385 | 2.850 | 14.456 | 0.628 |
| | 5 | WIPW | -2.1 | -2.396 | 0.096 | -0.085 | 1.042 | 0.000 | 0.937 | 3.431 | 0.950 | 3.077 | 0.933 | 3.541 | 0.338 | 1.751 | 9.205 | 0.283 |
| | 5 | AIPW | -2.1 | -2.067 | 0.131 | -0.035 | 1.061 | 0.000 | 0.940 | 3.348 | 0.940 | 3.354 | 0.944 | 3.252 | 0.405 | 4.597 | 17.092 | 1.067 |
| | 5 | WAIPW | -2.1 | -2.409 | 0.102 | -0.051 | 1.049 | 0.000 | 0.940 | 3.369 | 0.945 | 3.219 | 0.939 | 3.375 | 0.350 | 2.422 | 10.304 | 0.434 |
| WAIPW(0.5) | 5 | IPW | -2.1 | -1.657 | 0.070 | -0.062 | 1.008 | 0.000 | 0.949 | 3.123 | 0.950 | 3.077 | 0.943 | 3.290 | 0.270 | 0.534 | 4.883 | 0.106 |
| | 5 | WIPW | -2.1 | 2.659 | 0.068 | 0.005 | 1.009 | 0.005 | 0.947 | 3.169 | 0.946 | 3.202 | 0.949 | 3.112 | 0.263 | 0.467 | 4.679 | 0.101 |
| | 5 | AIPW | -2.1 | -1.981 | 0.069 | -0.063 | 1.015 | 0.000 | 0.944 | 3.246 | 0.950 | 3.094 | 0.943 | 3.268 | 0.266 | 0.517 | 4.774 | 0.103 |
| | 5 | WAIPW | -2.1 | 2.091 | 0.068 | -0.001 | 1.017 | 0.004 | 0.944 | 3.246 | 0.944 | 3.263 | 0.948 | 3.152 | 0.262 | 0.455 | 4.670 | 0.101 |
| WAIPW(1) | 5 | IPW | -2.1 | 0.137 | 0.095 | -0.108 | 1.041 | 0.002 | 0.940 | 3.359 | 0.948 | 3.146 | 0.937 | 3.441 | 0.343 | 1.711 | 9.046 | 0.259 |
| | 5 | WIPW | -2.1 | 9.066 | 0.079 | 0.025 | 1.028 | 0.011 | 0.945 | 3.213 | 0.938 | 3.401 | 0.950 | 3.088 | 0.294 | 0.972 | 6.408 | 0.160 |
| | 5 | AIPW | -2.1 | 0.075 | 0.094 | -0.098 | 1.034 | 0.002 | 0.943 | 3.284 | 0.949 | 3.123 | 0.941 | 3.327 | 0.344 | 1.834 | 8.880 | 0.253 |
| | 5 | WAIPW | -2.1 | 9.024 | 0.079 | 0.028 | 1.033 | 0.011 | 0.942 | 3.301 | 0.941 | 3.322 | 0.952 | 3.023 | 0.295 | 0.991 | 6.433 | 0.162 |
| WIPW(0.5) | 5 | IPW | -2.1 | -1.675 | 0.070 | -0.062 | 1.011 | 0.000 | 0.946 | 3.191 | 0.951 | 3.047 | 0.943 | 3.274 | 0.269 | 0.513 | 4.851 | 0.104 |

| Randomization Method | Regime | Estimator | True Value x 10^3 | Mean Estimate x 10^3 | SD Estimates x 10^3 | Mean Normalized | SD Normalized | Mean Bias | CI Coverage | SE CI Coverage x 10^3 | LB Coverage | SE LB Coverage x 10^3 | UB Coverage x 10^3 | SE UB Coverage x 10^3 | Mean CI Length | SE CI Length x 10^3 | MSE x 10^3 | SE MSE x 10^3 |
|---|---|---|---|---|---|---|---|---|---|---|---|---|---|---|---|---|---|---|
| | 5 | WIPW | -2.1 | 2.618 | 0.068 | 0.004 | 1.007 | 0.005 | 0.948 | 3.140 | 0.949 | 3.123 | 0.951 | 3.059 | 0.263 | 0.449 | 4.629 | 0.100 |
| | 5 | AIPW | -2.1 | -1.648 | 0.069 | -0.056 | 1.015 | 0.000 | 0.948 | 3.140 | 0.952 | 3.017 | 0.942 | 3.295 | 0.266 | 0.522 | 4.754 | 0.102 |
| | 5 | WAIPW | -2.1 | 2.380 | 0.068 | 0.006 | 1.012 | 0.004 | 0.944 | 3.241 | 0.947 | 3.180 | 0.951 | 3.053 | 0.263 | 0.461 | 4.626 | 0.098 |
| WIPW(1) | 5 | IPW | -2.1 | -1.193 | 0.095 | -0.121 | 1.040 | 0.001 | 0.943 | 3.290 | 0.953 | 2.993 | 0.931 | 3.580 | 0.342 | 1.680 | 9.062 | 0.250 |
| | 5 | WIPW | -2.1 | 7.706 | 0.079 | 0.010 | 1.028 | 0.010 | 0.946 | 3.186 | 0.943 | 3.268 | 0.947 | 3.180 | 0.293 | 0.937 | 6.348 | 0.160 |
| | 5 | AIPW | -2.1 | -1.232 | 0.094 | -0.107 | 1.040 | 0.001 | 0.943 | 3.274 | 0.951 | 3.059 | 0.934 | 3.512 | 0.341 | 1.804 | 8.924 | 0.239 |
| | 5 | WAIPW | -2.1 | 7.341 | 0.079 | 0.015 | 1.035 | 0.009 | 0.947 | 3.169 | 0.940 | 3.364 | 0.949 | 3.112 | 0.294 | 0.978 | 6.352 | 0.157 |
| AIPW(0.5) | 6 | IPW | -2.1 | 0.141 | 0.071 | -0.037 | 1.020 | 0.002 | 0.948 | 3.152 | 0.952 | 3.035 | 0.946 | 3.208 | 0.270 | 0.520 | 5.017 | 0.109 |
| | 6 | WIPW | -2.1 | 4.747 | 0.085 | 0.037 | 1.019 | 0.007 | 0.946 | 3.191 | 0.946 | 3.208 | 0.952 | 3.029 | 0.324 | 0.852 | 7.279 | 0.162 |
| | 6 | AIPW | -2.1 | 0.477 | 0.070 | -0.029 | 1.030 | 0.003 | 0.945 | 3.213 | 0.946 | 3.197 | 0.949 | 3.117 | 0.267 | 0.514 | 4.949 | 0.107 |
| | 6 | WAIPW | -2.1 | 4.258 | 0.069 | 0.030 | 1.027 | 0.006 | 0.947 | 3.180 | 0.939 | 3.385 | 0.952 | 3.035 | 0.263 | 0.453 | 4.832 | 0.102 |
| AIPW(1) | 6 | IPW | -2.1 | -3.941 | 0.096 | -0.149 | 1.036 | -0.002 | 0.937 | 3.426 | 0.955 | 2.944 | 0.932 | 3.551 | 0.348 | 1.685 | 9.169 | 0.254 |
| | 6 | WIPW | -2.1 | 6.684 | 0.113 | -0.005 | 1.046 | 0.009 | 0.935 | 3.487 | 0.939 | 3.395 | 0.943 | 3.274 | 0.397 | 2.119 | 12.801 | 0.380 |
| | 6 | AIPW | -2.1 | -3.612 | 0.095 | -0.135 | 1.038 | -0.002 | 0.939 | 3.380 | 0.953 | 2.981 | 0.934 | 3.512 | 0.348 | 1.766 | 8.970 | 0.236 |
| | 6 | WAIPW | -2.1 | 5.325 | 0.078 | -0.014 | 1.028 | 0.007 | 0.940 | 3.354 | 0.948 | 3.140 | 0.941 | 3.322 | 0.295 | 0.932 | 6.156 | 0.151 |
| AR-1 | 6 | IPW | -2.1 | -1.677 | 0.108 | -0.043 | 1.017 | 0.000 | 0.945 | 3.230 | 0.953 | 2.981 | 0.943 | 3.284 | 0.372 | 2.407 | 11.582 | 0.374 |
| | 6 | WIPW | -2.1 | 4.873 | 0.081 | 0.056 | 1.017 | 0.007 | 0.947 | 3.169 | 0.941 | 3.338 | 0.953 | 3.005 | 0.295 | 1.237 | 6.574 | 0.160 |
| | 6 | AIPW | -2.1 | -1.746 | 0.108 | -0.049 | 1.010 | 0.000 | 0.948 | 3.135 | 0.955 | 2.944 | 0.941 | 3.327 | 0.375 | 2.677 | 11.641 | 0.413 |
| | 6 | WAIPW | -2.1 | 4.224 | 0.084 | 0.036 | 1.022 | 0.006 | 0.947 | 3.169 | 0.944 | 3.263 | 0.951 | 3.053 | 0.306 | 1.464 | 7.160 | 0.185 |
| AR-2 | 6 | IPW | -2.1 | -1.920 | 0.090 | -0.055 | 1.020 | 0.000 | 0.946 | 3.208 | 0.950 | 3.088 | 0.941 | 3.327 | 0.325 | 1.869 | 8.062 | 0.233 |
| | 6 | WIPW | -2.1 | 3.173 | 0.073 | 0.026 | 1.020 | 0.005 | 0.943 | 3.274 | 0.945 | 3.213 | 0.950 | 3.071 | 0.273 | 0.979 | 5.416 | 0.135 |
| | 6 | AIPW | -2.1 | -2.113 | 0.089 | -0.058 | 1.012 | 0.000 | 0.948 | 3.146 | 0.954 | 2.969 | 0.942 | 3.306 | 0.324 | 1.951 | 7.878 | 0.231 |
| | 6 | WAIPW | -2.1 | 2.296 | 0.075 | 0.010 | 1.016 | 0.004 | 0.947 | 3.163 | 0.947 | 3.157 | 0.947 | 3.180 | 0.280 | 1.125 | 5.648 | 0.143 |
| IAIPW(0.5) | 6 | IPW | -2.1 | -2.471 | 0.070 | -0.072 | 1.010 | 0.000 | 0.949 | 3.112 | 0.955 | 2.932 | 0.940 | 3.369 | 0.271 | 0.523 | 4.879 | 0.106 |
| | 6 | WIPW | -2.1 | 1.926 | 0.068 | -0.004 | 1.009 | 0.004 | 0.951 | 3.053 | 0.951 | 3.065 | 0.947 | 3.169 | 0.264 | 0.457 | 4.664 | 0.101 |
| | 6 | AIPW | -2.1 | -2.217 | 0.069 | -0.065 | 1.021 | 0.000 | 0.944 | 3.241 | 0.952 | 3.023 | 0.940 | 3.364 | 0.267 | 0.506 | 4.818 | 0.105 |
| | 6 | WAIPW | -2.1 | 2.159 | 0.069 | 0.002 | 1.020 | 0.004 | 0.943 | 3.274 | 0.947 | 3.157 | 0.949 | 3.117 | 0.263 | 0.448 | 4.730 | 0.104 |
| IAIPW(1) | 6 | IPW | -2.1 | -1.935 | 0.097 | -0.132 | 1.063 | 0.000 | 0.933 | 3.531 | 0.946 | 3.191 | 0.930 | 3.604 | 0.347 | 1.647 | 9.485 | 0.252 |
| | 6 | WIPW | -2.1 | 6.429 | 0.079 | -0.005 | 1.034 | 0.009 | 0.941 | 3.333 | 0.942 | 3.311 | 0.945 | 3.230 | 0.294 | 0.921 | 6.320 | 0.153 |
| | 6 | AIPW | -2.1 | -1.660 | 0.097 | -0.122 | 1.062 | 0.000 | 0.937 | 3.431 | 0.945 | 3.230 | 0.932 | 3.556 | 0.347 | 1.748 | 9.412 | 0.252 |
| | 6 | WAIPW | -2.1 | 6.318 | 0.079 | -0.004 | 1.042 | 0.008 | 0.936 | 3.462 | 0.944 | 3.252 | 0.943 | 3.279 | 0.294 | 0.923 | 6.371 | 0.153 |
| IPW(0.5) | 6 | IPW | -2.1 | -2.482 | 0.068 | -0.070 | 0.992 | 0.000 | 0.954 | 2.951 | 0.956 | 2.901 | 0.946 | 3.197 | 0.270 | 0.510 | 4.651 | 0.097 |
| | 6 | WIPW | -2.1 | 0.656 | 0.084 | -0.004 | 1.010 | 0.003 | 0.947 | 3.169 | 0.954 | 2.969 | 0.947 | 3.163 | 0.324 | 0.846 | 7.010 | 0.149 |
| | 6 | AIPW | -2.1 | -2.174 | 0.067 | -0.062 | 1.000 | 0.000 | 0.949 | 3.100 | 0.954 | 2.951 | 0.947 | 3.180 | 0.266 | 0.518 | 4.555 | 0.094 |
| | 6 | WAIPW | -2.1 | 1.929 | 0.067 | 0.002 | 0.998 | 0.004 | 0.949 | 3.100 | 0.949 | 3.100 | 0.951 | 3.053 | 0.263 | 0.457 | 4.458 | 0.091 |
| IPW(1) | 6 | IPW | -2.1 | -2.817 | 0.093 | -0.137 | 1.024 | -0.001 | 0.945 | 3.224 | 0.951 | 3.059 | 0.938 | 3.416 | 0.342 | 1.603 | 8.611 | 0.234 |
| | 6 | WIPW | -2.1 | 8.755 | 0.109 | 0.019 | 1.036 | 0.011 | 0.937 | 3.441 | 0.940 | 3.359 | 0.947 | 3.157 | 0.393 | 2.025 | 12.078 | 0.343 |
| | 6 | AIPW | -2.1 | -2.637 | 0.093 | -0.121 | 1.029 | -0.001 | 0.944 | 3.263 | 0.948 | 3.152 | 0.940 | 3.359 | 0.343 | 1.730 | 8.557 | 0.233 |
| | 6 | WAIPW | -2.1 | 5.735 | 0.077 | -0.003 | 1.018 | 0.008 | 0.950 | 3.088 | 0.946 | 3.208 | 0.952 | 3.035 | 0.293 | 0.922 | 6.044 | 0.152 |
| SR | 6 | IPW | -2.1 | -1.829 | 0.065 | 0.006 | 1.022 | 0.000 | 0.945 | 3.219 | 0.944 | 3.246 | 0.947 | 3.169 | 0.251 | 0.185 | 4.265 | 0.085 |
| | 6 | WIPW | -2.1 | -2.487 | 0.077 | -0.004 | 1.017 | 0.000 | 0.948 | 3.146 | 0.948 | 3.152 | 0.946 | 3.208 | 0.299 | 0.278 | 5.985 | 0.120 |
| | 6 | AIPW | -2.1 | -1.758 | 0.065 | 0.008 | 1.027 | 0.000 | 0.945 | 3.224 | 0.946 | 3.208 | 0.946 | 3.197 | 0.248 | 0.180 | 4.192 | 0.084 |
| | 6 | WAIPW | -2.1 | -1.673 | 0.066 | 0.009 | 1.029 | 0.000 | 0.942 | 3.295 | 0.946 | 3.202 | 0.948 | 3.146 | 0.251 | 0.185 | 4.319 | 0.087 |
| TS(0.25) | 6 | IPW | -2.1 | -1.434 | 0.083 | -0.052 | 1.013 | 0.001 | 0.946 | 3.197 | 0.953 | 2.981 | 0.941 | 3.338 | 0.280 | 1.867 | 6.823 | 0.289 |

| Randomization Method | Regime | Estimator | True Value x 10^3 | Mean Estimate x 10^3 | SD Estimates x 10^3 | Mean Normalized | SD Normalized | Mean Bias | CI Coverage | SE CI Coverage x 10^3 | LB Coverage | SE LB Coverage x 10^3 | UB Coverage | SE UB Coverage x 10^3 | Mean CI Length | SE CI Length x 10^3 | MSE x 10^3 | SE MSE x 10^3 |
|---|---|---|---|---|---|---|---|---|---|---|---|---|---|---|---|---|---|---|
| | 6 | WIPW | -2.1 | 0.504 | 0.069 | -0.023 | 1.007 | 0.003 | 0.948 | 3.135 | 0.952 | 3.017 | 0.943 | 3.274 | 0.263 | 0.743 | 4.783 | 0.119 |
| | 6 | AIPW | -2.1 | -1.216 | 0.087 | -0.037 | 1.035 | 0.001 | 0.938 | 3.406 | 0.946 | 3.191 | 0.942 | 3.301 | 0.288 | 2.969 | 7.594 | 0.394 |
| | 6 | WAIPW | -2.1 | 0.286 | 0.072 | -0.021 | 1.027 | 0.002 | 0.942 | 3.311 | 0.947 | 3.174 | 0.942 | 3.301 | 0.266 | 1.419 | 5.223 | 0.265 |
| TS(0.50) | 6 | IPW | -2.1 | -1.678 | 0.096 | -0.092 | 1.006 | 0.000 | 0.947 | 3.180 | 0.954 | 2.951 | 0.940 | 3.348 | 0.301 | 1.963 | 9.146 | 0.886 |
| | 6 | WIPW | -2.1 | 1.790 | 0.077 | -0.046 | 1.000 | 0.004 | 0.948 | 3.135 | 0.952 | 3.023 | 0.948 | 3.152 | 0.282 | 1.125 | 5.989 | 0.313 |
| | 6 | AIPW | -2.1 | -0.990 | 0.114 | -0.072 | 1.018 | 0.001 | 0.944 | 3.246 | 0.954 | 2.969 | 0.946 | 3.191 | 0.314 | 4.348 | 13.029 | 2.720 |
| | 6 | WAIPW | -2.1 | 1.168 | 0.082 | -0.050 | 1.016 | 0.003 | 0.945 | 3.224 | 0.952 | 3.023 | 0.946 | 3.197 | 0.288 | 1.756 | 6.685 | 0.436 |
| TS(0.75) | 6 | IPW | -2.1 | -1.533 | 0.118 | -0.111 | 1.118 | 0.001 | 0.936 | 3.472 | 0.950 | 3.077 | 0.935 | 3.482 | 0.349 | 2.899 | 13.867 | 0.850 |
| | 6 | WIPW | -2.1 | 4.031 | 0.092 | -0.057 | 1.047 | 0.006 | 0.940 | 3.364 | 0.946 | 3.186 | 0.943 | 3.284 | 0.315 | 1.776 | 8.508 | 0.290 |
| | 6 | AIPW | -2.1 | -2.346 | 0.162 | -0.070 | 1.090 | 0.000 | 0.932 | 3.556 | 0.943 | 3.274 | 0.941 | 3.322 | 0.380 | 7.907 | 26.245 | 4.311 |
| | 6 | WAIPW | -2.1 | 2.409 | 0.119 | -0.040 | 1.075 | 0.005 | 0.931 | 3.580 | 0.938 | 3.406 | 0.943 | 3.290 | 0.336 | 4.867 | 14.141 | 3.216 |
| TS(1) | 6 | IPW | -2.1 | -2.948 | 0.141 | -0.133 | 1.146 | -0.001 | 0.928 | 3.651 | 0.943 | 3.268 | 0.928 | 3.651 | 0.388 | 3.652 | 19.793 | 1.012 |
| | 6 | WIPW | -2.1 | 3.565 | 0.108 | -0.084 | 1.075 | 0.006 | 0.934 | 3.521 | 0.941 | 3.338 | 0.935 | 3.492 | 0.341 | 2.281 | 11.776 | 0.569 |
| | 6 | AIPW | -2.1 | 0.471 | 0.197 | -0.076 | 1.148 | 0.003 | 0.925 | 3.716 | 0.931 | 3.585 | 0.938 | 3.421 | 0.433 | 9.066 | 38.750 | 10.323 |
| | 6 | WAIPW | -2.1 | 4.295 | 0.141 | -0.056 | 1.108 | 0.006 | 0.926 | 3.707 | 0.931 | 3.575 | 0.937 | 3.431 | 0.373 | 5.384 | 19.991 | 3.280 |
| WAIPW(0.5) | 6 | IPW | -2.1 | -1.814 | 0.071 | -0.065 | 1.026 | 0.000 | 0.945 | 3.230 | 0.949 | 3.117 | 0.945 | 3.235 | 0.270 | 0.537 | 5.029 | 0.110 |
| | 6 | WIPW | -2.1 | 2.568 | 0.069 | 0.003 | 1.023 | 0.005 | 0.944 | 3.257 | 0.942 | 3.317 | 0.951 | 3.047 | 0.263 | 0.470 | 4.789 | 0.104 |
| | 6 | AIPW | -2.1 | -1.572 | 0.070 | -0.059 | 1.032 | 0.001 | 0.942 | 3.301 | 0.948 | 3.135 | 0.946 | 3.197 | 0.266 | 0.529 | 4.933 | 0.107 |
| | 6 | WAIPW | -2.1 | 2.878 | 0.069 | 0.008 | 1.033 | 0.005 | 0.940 | 3.364 | 0.941 | 3.338 | 0.951 | 3.053 | 0.263 | 0.466 | 4.841 | 0.105 |
| WAIPW(1) | 6 | IPW | -2.1 | 0.171 | 0.094 | -0.103 | 1.037 | 0.002 | 0.942 | 3.295 | 0.954 | 2.975 | 0.934 | 3.502 | 0.344 | 1.728 | 8.870 | 0.236 |
| | 6 | WIPW | -2.1 | 9.048 | 0.078 | 0.029 | 1.018 | 0.011 | 0.947 | 3.157 | 0.945 | 3.213 | 0.951 | 3.053 | 0.295 | 0.982 | 6.269 | 0.154 |
| | 6 | AIPW | -2.1 | 0.529 | 0.093 | -0.088 | 1.038 | 0.003 | 0.942 | 3.306 | 0.951 | 3.059 | 0.937 | 3.426 | 0.343 | 1.808 | 8.613 | 0.223 |
| | 6 | WAIPW | -2.1 | 9.445 | 0.079 | 0.035 | 1.028 | 0.012 | 0.946 | 3.208 | 0.939 | 3.375 | 0.951 | 3.059 | 0.295 | 0.984 | 6.316 | 0.153 |
| WIPW(0.5) | 6 | IPW | -2.1 | -3.105 | 0.070 | -0.080 | 1.013 | -0.001 | 0.947 | 3.163 | 0.957 | 2.863 | 0.940 | 3.359 | 0.269 | 0.509 | 4.837 | 0.106 |
| | 6 | WIPW | -2.1 | 1.295 | 0.068 | -0.012 | 1.010 | 0.003 | 0.947 | 3.157 | 0.952 | 3.035 | 0.946 | 3.202 | 0.263 | 0.449 | 4.601 | 0.099 |
| | 6 | AIPW | -2.1 | -3.048 | 0.069 | -0.074 | 1.015 | -0.001 | 0.944 | 3.252 | 0.957 | 2.863 | 0.939 | 3.380 | 0.266 | 0.519 | 4.718 | 0.104 |
| | 6 | WAIPW | -2.1 | 0.924 | 0.068 | -0.012 | 1.011 | 0.003 | 0.948 | 3.135 | 0.951 | 3.059 | 0.948 | 3.135 | 0.263 | 0.463 | 4.580 | 0.098 |
| WIPW(1) | 6 | IPW | -2.1 | -1.947 | 0.094 | -0.132 | 1.030 | 0.000 | 0.937 | 3.431 | 0.952 | 3.017 | 0.932 | 3.570 | 0.343 | 1.689 | 8.920 | 0.246 |
| | 6 | WIPW | -2.1 | 6.708 | 0.079 | -0.006 | 1.022 | 0.009 | 0.943 | 3.274 | 0.944 | 3.241 | 0.948 | 3.129 | 0.295 | 0.945 | 6.275 | 0.156 |
| | 6 | AIPW | -2.1 | -1.770 | 0.094 | -0.115 | 1.038 | 0.000 | 0.940 | 3.354 | 0.948 | 3.140 | 0.934 | 3.517 | 0.344 | 1.830 | 8.881 | 0.238 |
| | 6 | WAIPW | -2.1 | 6.462 | 0.079 | 0.000 | 1.031 | 0.009 | 0.941 | 3.343 | 0.939 | 3.380 | 0.945 | 3.230 | 0.295 | 0.994 | 6.305 | 0.153 |
| AIPW(0.5) | 7 | IPW | -2.1 | -1.913 | 0.071 | -0.067 | 1.033 | 0.000 | 0.947 | 3.157 | 0.955 | 2.920 | 0.938 | 3.411 | 0.270 | 0.515 | 5.044 | 0.106 |
| | 7 | WIPW | -2.1 | 2.263 | 0.085 | 0.009 | 1.021 | 0.004 | 0.946 | 3.197 | 0.943 | 3.274 | 0.950 | 3.083 | 0.324 | 0.847 | 7.188 | 0.157 |
| | 7 | AIPW | -2.1 | -1.455 | 0.070 | -0.059 | 1.037 | 0.001 | 0.945 | 3.224 | 0.953 | 2.987 | 0.940 | 3.348 | 0.267 | 0.510 | 4.946 | 0.104 |
| | 7 | WAIPW | -2.1 | 2.735 | 0.069 | 0.007 | 1.035 | 0.005 | 0.944 | 3.263 | 0.946 | 3.197 | 0.946 | 3.186 | 0.263 | 0.449 | 4.838 | 0.103 |
| AIPW(1) | 7 | IPW | -2.1 | -2.135 | 0.094 | -0.123 | 1.024 | 0.000 | 0.941 | 3.322 | 0.954 | 2.951 | 0.938 | 3.406 | 0.350 | 1.659 | 8.819 | 0.220 |
| | 7 | WIPW | -2.1 | 8.513 | 0.109 | 0.021 | 1.027 | 0.011 | 0.942 | 3.295 | 0.946 | 3.202 | 0.949 | 3.106 | 0.400 | 2.079 | 12.041 | 0.315 |
| | 7 | AIPW | -2.1 | -1.755 | 0.093 | -0.111 | 1.019 | 0.000 | 0.944 | 3.246 | 0.955 | 2.944 | 0.942 | 3.306 | 0.349 | 1.745 | 8.634 | 0.215 |
| | 7 | WAIPW | -2.1 | 7.278 | 0.078 | 0.020 | 1.013 | 0.009 | 0.948 | 3.140 | 0.943 | 3.274 | 0.952 | 3.029 | 0.295 | 0.905 | 6.109 | 0.141 |
| AR-1 | 7 | IPW | -2.1 | -4.984 | 0.108 | -0.090 | 1.050 | -0.003 | 0.937 | 3.431 | 0.950 | 3.094 | 0.935 | 3.477 | 0.371 | 2.329 | 11.708 | 0.330 |
| | 7 | WIPW | -2.1 | -0.509 | 0.081 | -0.030 | 1.031 | 0.002 | 0.943 | 3.279 | 0.947 | 3.180 | 0.943 | 3.279 | 0.292 | 1.199 | 6.505 | 0.158 |
| | 7 | AIPW | -2.1 | -4.912 | 0.107 | -0.096 | 1.037 | -0.003 | 0.944 | 3.257 | 0.949 | 3.106 | 0.936 | 3.467 | 0.373 | 2.543 | 11.491 | 0.337 |
| | 7 | WAIPW | -2.1 | -1.843 | 0.084 | -0.060 | 1.030 | 0.000 | 0.944 | 3.263 | 0.951 | 3.065 | 0.942 | 3.306 | 0.304 | 1.442 | 7.055 | 0.178 |
| AR-2 | 7 | IPW | -2.1 | -0.345 | 0.092 | -0.050 | 1.038 | 0.002 | 0.941 | 3.327 | 0.949 | 3.106 | 0.940 | 3.364 | 0.323 | 1.802 | 8.409 | 0.233 |

| Randomization Method | Regime | Estimator | True Value x 10^3 | Mean Estimate x 10^3 | SD Estimates x 10^3 | Mean Normalized | SD Normalized | Mean Bias | CI Coverage | SE CI Coverage x 10^3 | LB Coverage | SE LB Coverage x 10^3 | UB Coverage | SE UB Coverage x 10^3 | Mean CI Length | SE CI Length x 10^3 | MSE x 10^3 | SE MSE x 10^3 |
|---|---|---|---|---|---|---|---|---|---|---|---|---|---|---|---|---|---|---|
| | 7 | WIPW | -2.1 | 2.549 | 0.075 | 0.004 | 1.031 | 0.005 | 0.940 | 3.364 | 0.946 | 3.197 | 0.943 | 3.274 | 0.273 | 0.960 | 5.579 | 0.134 |
| | 7 | AIPW | -2.1 | -0.528 | 0.091 | -0.051 | 1.030 | 0.002 | 0.942 | 3.301 | 0.949 | 3.123 | 0.940 | 3.369 | 0.323 | 1.892 | 8.235 | 0.231 |
| | 7 | WAIPW | -2.1 | 1.437 | 0.077 | -0.015 | 1.033 | 0.004 | 0.943 | 3.268 | 0.948 | 3.146 | 0.940 | 3.354 | 0.280 | 1.133 | 5.919 | 0.149 |
| IAIPW(0.5) | 7 | IPW | -2.1 | -2.715 | 0.069 | -0.074 | 0.995 | -0.001 | 0.954 | 2.957 | 0.958 | 2.850 | 0.945 | 3.224 | 0.271 | 0.519 | 4.743 | 0.103 |
| | 7 | WIPW | -2.1 | 1.879 | 0.067 | -0.004 | 0.994 | 0.004 | 0.955 | 2.944 | 0.951 | 3.059 | 0.951 | 3.041 | 0.264 | 0.455 | 4.517 | 0.098 |
| | 7 | AIPW | -2.1 | -2.508 | 0.068 | -0.069 | 1.002 | 0.000 | 0.951 | 3.065 | 0.955 | 2.920 | 0.943 | 3.290 | 0.267 | 0.514 | 4.673 | 0.103 |
| | 7 | WAIPW | -2.1 | 2.132 | 0.067 | 0.001 | 1.000 | 0.004 | 0.951 | 3.059 | 0.952 | 3.011 | 0.951 | 3.059 | 0.263 | 0.454 | 4.537 | 0.101 |
| IAIPW(1) | 7 | IPW | -2.1 | -5.724 | 0.093 | -0.161 | 1.009 | -0.004 | 0.945 | 3.219 | 0.960 | 2.772 | 0.935 | 3.477 | 0.348 | 1.656 | 8.577 | 0.228 |
| | 7 | WIPW | -2.1 | 4.148 | 0.077 | -0.027 | 1.003 | 0.006 | 0.946 | 3.202 | 0.949 | 3.112 | 0.946 | 3.191 | 0.295 | 0.910 | 5.946 | 0.149 |
| | 7 | AIPW | -2.1 | -5.647 | 0.092 | -0.153 | 1.004 | -0.004 | 0.944 | 3.246 | 0.956 | 2.888 | 0.938 | 3.406 | 0.348 | 1.750 | 8.513 | 0.230 |
| | 7 | WAIPW | -2.1 | 4.006 | 0.077 | -0.025 | 1.007 | 0.006 | 0.946 | 3.186 | 0.948 | 3.146 | 0.946 | 3.197 | 0.295 | 0.912 | 5.971 | 0.150 |
| IPW(0.5) | 7 | IPW | -2.1 | -3.252 | 0.069 | -0.084 | 1.006 | -0.001 | 0.948 | 3.146 | 0.956 | 2.913 | 0.943 | 3.284 | 0.269 | 0.508 | 4.796 | 0.100 |
| | 7 | WIPW | -2.1 | -0.369 | 0.084 | -0.019 | 1.007 | 0.002 | 0.947 | 3.169 | 0.952 | 3.035 | 0.944 | 3.252 | 0.324 | 0.847 | 6.994 | 0.151 |
| | 7 | AIPW | -2.1 | -2.847 | 0.069 | -0.076 | 1.015 | -0.001 | 0.947 | 3.169 | 0.955 | 2.932 | 0.940 | 3.364 | 0.266 | 0.515 | 4.727 | 0.098 |
| | 7 | WAIPW | -2.1 | 1.420 | 0.068 | -0.009 | 1.014 | 0.004 | 0.948 | 3.135 | 0.946 | 3.186 | 0.948 | 3.135 | 0.262 | 0.456 | 4.609 | 0.095 |
| IPW(1) | 7 | IPW | -2.1 | -2.401 | 0.094 | -0.130 | 1.040 | 0.000 | 0.940 | 3.359 | 0.951 | 3.065 | 0.937 | 3.431 | 0.342 | 1.586 | 8.912 | 0.247 |
| | 7 | WIPW | -2.1 | 8.772 | 0.110 | 0.023 | 1.050 | 0.011 | 0.938 | 3.401 | 0.936 | 3.452 | 0.947 | 3.169 | 0.392 | 1.990 | 12.326 | 0.348 |
| | 7 | AIPW | -2.1 | -1.928 | 0.094 | -0.115 | 1.032 | 0.000 | 0.944 | 3.241 | 0.949 | 3.123 | 0.940 | 3.348 | 0.343 | 1.729 | 8.877 | 0.256 |
| | 7 | WAIPW | -2.1 | 6.228 | 0.078 | 0.006 | 1.019 | 0.008 | 0.942 | 3.306 | 0.942 | 3.301 | 0.952 | 3.035 | 0.292 | 0.911 | 6.150 | 0.158 |
| SR | 7 | IPW | -2.1 | -1.749 | 0.065 | 0.005 | 1.015 | 0.000 | 0.950 | 3.071 | 0.951 | 3.059 | 0.950 | 3.077 | 0.251 | 0.186 | 4.210 | 0.084 |
| | 7 | WIPW | -2.1 | -1.479 | 0.077 | 0.006 | 1.010 | 0.001 | 0.949 | 3.112 | 0.947 | 3.157 | 0.947 | 3.169 | 0.299 | 0.280 | 5.914 | 0.122 |
| | 7 | AIPW | -2.1 | -1.641 | 0.064 | 0.007 | 1.020 | 0.000 | 0.947 | 3.169 | 0.949 | 3.117 | 0.949 | 3.112 | 0.248 | 0.186 | 4.131 | 0.082 |
| | 7 | WAIPW | -2.1 | -1.330 | 0.065 | 0.012 | 1.019 | 0.001 | 0.950 | 3.094 | 0.947 | 3.169 | 0.950 | 3.088 | 0.251 | 0.190 | 4.217 | 0.083 |
| TS(0.25) | 7 | IPW | -2.1 | -2.767 | 0.083 | -0.061 | 0.995 | -0.001 | 0.950 | 3.094 | 0.955 | 2.932 | 0.948 | 3.146 | 0.279 | 1.928 | 6.824 | 0.338 |
| | 7 | WIPW | -2.1 | 0.923 | 0.067 | -0.006 | 0.987 | 0.003 | 0.950 | 3.094 | 0.953 | 2.987 | 0.951 | 3.041 | 0.261 | 0.619 | 4.472 | 0.109 |
| | 7 | AIPW | -2.1 | -2.744 | 0.089 | -0.052 | 0.997 | -0.001 | 0.949 | 3.100 | 0.956 | 2.913 | 0.950 | 3.094 | 0.288 | 3.023 | 7.977 | 0.605 |
| | 7 | WAIPW | -2.1 | 0.660 | 0.067 | -0.008 | 0.991 | 0.003 | 0.950 | 3.083 | 0.952 | 3.035 | 0.951 | 3.053 | 0.261 | 0.754 | 4.539 | 0.114 |
| TS(0.50) | 7 | IPW | -2.1 | 0.180 | 0.096 | -0.054 | 1.024 | 0.002 | 0.944 | 3.263 | 0.951 | 3.047 | 0.941 | 3.333 | 0.302 | 1.964 | 9.286 | 0.803 |
| | 7 | WIPW | -2.1 | 6.237 | 0.075 | 0.032 | 1.005 | 0.008 | 0.948 | 3.152 | 0.946 | 3.202 | 0.950 | 3.071 | 0.276 | 0.945 | 5.643 | 0.186 |
| | 7 | AIPW | -2.1 | 0.994 | 0.113 | -0.037 | 1.020 | 0.003 | 0.945 | 3.224 | 0.951 | 3.053 | 0.943 | 3.268 | 0.314 | 4.409 | 12.759 | 2.521 |
| | 7 | WAIPW | -2.1 | 5.501 | 0.076 | 0.027 | 1.016 | 0.008 | 0.947 | 3.157 | 0.945 | 3.219 | 0.947 | 3.174 | 0.278 | 1.127 | 5.801 | 0.192 |
| TS(0.75) | 7 | IPW | -2.1 | -2.197 | 0.116 | -0.096 | 1.190 | 0.000 | 0.931 | 3.594 | 0.945 | 3.219 | 0.934 | 3.512 | 0.351 | 2.931 | 13.425 | 0.743 |
| | 7 | WIPW | -2.1 | 6.886 | 0.084 | 0.006 | 1.038 | 0.009 | 0.942 | 3.295 | 0.941 | 3.333 | 0.945 | 3.230 | 0.301 | 1.429 | 7.095 | 0.200 |
| | 7 | AIPW | -2.1 | -2.870 | 0.161 | -0.059 | 1.086 | -0.001 | 0.933 | 3.531 | 0.937 | 3.436 | 0.939 | 3.385 | 0.384 | 7.905 | 25.766 | 4.068 |
| | 7 | WAIPW | -2.1 | 6.646 | 0.088 | 0.016 | 1.056 | 0.009 | 0.941 | 3.343 | 0.934 | 3.507 | 0.948 | 3.135 | 0.307 | 1.852 | 7.740 | 0.284 |
| TS(1) | 7 | IPW | -2.1 | -2.069 | 0.140 | -0.099 | 1.166 | 0.000 | 0.926 | 3.707 | 0.942 | 3.306 | 0.930 | 3.618 | 0.388 | 3.293 | 19.567 | 1.119 |
| | 7 | WIPW | -2.1 | 9.789 | 0.092 | 0.022 | 1.036 | 0.012 | 0.941 | 3.338 | 0.937 | 3.436 | 0.951 | 3.059 | 0.319 | 1.745 | 8.517 | 0.300 |
| | 7 | AIPW | -2.1 | -1.579 | 0.236 | -0.049 | 1.107 | 0.001 | 0.927 | 3.684 | 0.935 | 3.477 | 0.941 | 3.343 | 0.443 | 11.282 | 55.636 | 16.513 |
| | 7 | WAIPW | -2.1 | 9.177 | 0.097 | 0.031 | 1.055 | 0.011 | 0.936 | 3.462 | 0.932 | 3.570 | 0.953 | 2.993 | 0.329 | 2.482 | 9.466 | 0.449 |
| WAIPW(0.5) | 7 | IPW | -2.1 | -0.964 | 0.070 | -0.049 | 1.018 | 0.001 | 0.947 | 3.169 | 0.952 | 3.035 | 0.946 | 3.202 | 0.270 | 0.532 | 4.957 | 0.108 |
| | 7 | WIPW | -2.1 | 3.295 | 0.069 | 0.017 | 1.016 | 0.005 | 0.949 | 3.106 | 0.947 | 3.180 | 0.953 | 3.005 | 0.264 | 0.465 | 4.747 | 0.103 |
| | 7 | AIPW | -2.1 | -0.457 | 0.070 | -0.040 | 1.026 | 0.002 | 0.945 | 3.213 | 0.950 | 3.094 | 0.947 | 3.169 | 0.267 | 0.521 | 4.910 | 0.108 |
| | 7 | WAIPW | -2.1 | 4.008 | 0.069 | 0.028 | 1.025 | 0.006 | 0.944 | 3.252 | 0.943 | 3.290 | 0.955 | 2.932 | 0.263 | 0.457 | 4.807 | 0.105 |
| WAIPW(1) | 7 | IPW | -2.1 | -2.061 | 0.094 | -0.127 | 1.039 | 0.000 | 0.942 | 3.295 | 0.955 | 2.932 | 0.935 | 3.497 | 0.342 | 1.697 | 8.889 | 0.252 |

| Randomization Method | Regime | Estimator | True Value x 10^3 | Mean Estimate x 10^3 | SD Estimates x 10^3 | Mean Normalized | SD Normalized | Mean Bias | CI Coverage | SE CI Coverage x 10^3 | LB Coverage | SE LB Coverage x 10^3 | UB Coverage | SE UB Coverage x 10^3 | Mean CI Length | SE CI Length x 10^3 | MSE x 10^3 | SE MSE x 10^3 |
|---|---|---|---|---|---|---|---|---|---|---|---|---|---|---|---|---|---|---|
| | 7 | WIPW | -2.1 | 6.734 | 0.078 | 0.000 | 1.020 | 0.009 | 0.945 | 3.219 | 0.947 | 3.163 | 0.948 | 3.129 | 0.294 | 0.966 | 6.194 | 0.155 |
| | 7 | AIPW | -2.1 | -1.684 | 0.093 | -0.116 | 1.033 | 0.000 | 0.945 | 3.219 | 0.953 | 2.987 | 0.938 | 3.411 | 0.342 | 1.784 | 8.733 | 0.242 |
| | 7 | WAIPW | -2.1 | 7.048 | 0.078 | 0.005 | 1.023 | 0.009 | 0.945 | 3.224 | 0.942 | 3.295 | 0.952 | 3.023 | 0.294 | 0.974 | 6.205 | 0.156 |
| WIPW(0.5) | 7 | IPW | -2.1 | -2.749 | 0.069 | -0.074 | 1.004 | -0.001 | 0.952 | 3.011 | 0.954 | 2.963 | 0.944 | 3.257 | 0.270 | 0.515 | 4.827 | 0.102 |
| | 7 | WIPW | -2.1 | 1.692 | 0.068 | -0.006 | 1.004 | 0.004 | 0.953 | 2.981 | 0.948 | 3.135 | 0.952 | 3.029 | 0.263 | 0.450 | 4.613 | 0.099 |
| | 7 | AIPW | -2.1 | -2.700 | 0.069 | -0.070 | 1.013 | -0.001 | 0.947 | 3.163 | 0.951 | 3.041 | 0.943 | 3.268 | 0.267 | 0.522 | 4.782 | 0.103 |
| | 7 | WAIPW | -2.1 | 1.396 | 0.068 | -0.007 | 1.014 | 0.003 | 0.947 | 3.174 | 0.945 | 3.230 | 0.951 | 3.047 | 0.263 | 0.461 | 4.669 | 0.100 |
| WIPW(1) | 7 | IPW | -2.1 | -2.070 | 0.095 | -0.133 | 1.037 | 0.000 | 0.939 | 3.385 | 0.954 | 2.969 | 0.933 | 3.531 | 0.344 | 1.664 | 8.928 | 0.241 |
| | 7 | WIPW | -2.1 | 7.388 | 0.079 | 0.004 | 1.023 | 0.009 | 0.943 | 3.279 | 0.943 | 3.279 | 0.948 | 3.135 | 0.295 | 0.938 | 6.264 | 0.152 |
| | 7 | AIPW | -2.1 | -1.772 | 0.095 | -0.118 | 1.038 | 0.000 | 0.940 | 3.359 | 0.952 | 3.029 | 0.937 | 3.431 | 0.344 | 1.820 | 8.961 | 0.238 |
| | 7 | WAIPW | -2.1 | 7.100 | 0.079 | 0.009 | 1.026 | 0.009 | 0.942 | 3.317 | 0.940 | 3.364 | 0.950 | 3.094 | 0.296 | 0.992 | 6.309 | 0.152 |
| AIPW(0.5) | 8 | IPW | -2.1 | -1.741 | 0.070 | -0.059 | 1.010 | 0.000 | 0.948 | 3.140 | 0.954 | 2.951 | 0.942 | 3.306 | 0.271 | 0.517 | 4.837 | 0.105 |
| | 8 | WIPW | -2.1 | 1.772 | 0.085 | 0.009 | 1.017 | 0.004 | 0.947 | 3.180 | 0.948 | 3.140 | 0.950 | 3.083 | 0.326 | 0.854 | 7.204 | 0.156 |
| | 8 | AIPW | -2.1 | -1.681 | 0.069 | -0.057 | 1.016 | 0.000 | 0.946 | 3.208 | 0.952 | 3.023 | 0.940 | 3.348 | 0.268 | 0.508 | 4.772 | 0.103 |
| | 8 | WAIPW | -2.1 | 2.718 | 0.068 | 0.011 | 1.012 | 0.005 | 0.944 | 3.257 | 0.947 | 3.163 | 0.948 | 3.135 | 0.263 | 0.446 | 4.621 | 0.100 |
| AIPW(1) | 8 | IPW | -2.1 | -1.082 | 0.096 | -0.116 | 1.036 | 0.001 | 0.936 | 3.452 | 0.953 | 3.005 | 0.930 | 3.613 | 0.351 | 1.644 | 9.221 | 0.242 |
| | 8 | WIPW | -2.1 | 8.761 | 0.112 | 0.014 | 1.038 | 0.011 | 0.940 | 3.348 | 0.940 | 3.348 | 0.947 | 3.157 | 0.401 | 2.045 | 12.634 | 0.351 |
| | 8 | AIPW | -2.1 | -0.776 | 0.095 | -0.108 | 1.028 | 0.001 | 0.941 | 3.322 | 0.951 | 3.041 | 0.932 | 3.551 | 0.351 | 1.765 | 8.981 | 0.232 |
| | 8 | WAIPW | -2.1 | 7.762 | 0.079 | 0.020 | 1.026 | 0.010 | 0.942 | 3.306 | 0.942 | 3.301 | 0.944 | 3.252 | 0.296 | 0.915 | 6.339 | 0.152 |
| AR-1 | 8 | IPW | -2.1 | -5.043 | 0.114 | -0.015 | 1.059 | -0.003 | 0.938 | 3.401 | 0.944 | 3.252 | 0.938 | 3.416 | 0.380 | 2.707 | 12.943 | 0.410 |
| | 8 | WIPW | -2.1 | -5.950 | 0.080 | -0.029 | 1.020 | -0.004 | 0.944 | 3.241 | 0.952 | 3.017 | 0.942 | 3.317 | 0.292 | 1.414 | 6.356 | 0.168 |
| | 8 | AIPW | -2.1 | -4.942 | 0.114 | -0.018 | 1.044 | -0.003 | 0.945 | 3.219 | 0.946 | 3.186 | 0.943 | 3.284 | 0.384 | 2.992 | 13.034 | 0.438 |
| | 8 | WAIPW | -2.1 | -6.764 | 0.084 | -0.046 | 1.031 | -0.005 | 0.946 | 3.202 | 0.952 | 3.035 | 0.941 | 3.333 | 0.303 | 1.633 | 7.049 | 0.195 |
| AR-2 | 8 | IPW | -2.1 | -2.190 | 0.093 | -0.010 | 1.038 | 0.000 | 0.943 | 3.274 | 0.943 | 3.279 | 0.945 | 3.224 | 0.327 | 1.963 | 8.651 | 0.233 |
| | 8 | WIPW | -2.1 | -2.827 | 0.073 | -0.013 | 1.021 | -0.001 | 0.942 | 3.311 | 0.945 | 3.230 | 0.947 | 3.157 | 0.273 | 1.056 | 5.312 | 0.123 |
| | 8 | AIPW | -2.1 | -2.405 | 0.092 | -0.013 | 1.036 | 0.000 | 0.942 | 3.295 | 0.944 | 3.241 | 0.943 | 3.268 | 0.327 | 2.046 | 8.503 | 0.230 |
| | 8 | WAIPW | -2.1 | -3.563 | 0.075 | -0.025 | 1.030 | -0.001 | 0.942 | 3.295 | 0.946 | 3.197 | 0.945 | 3.213 | 0.280 | 1.210 | 5.691 | 0.134 |
| IAIPW(0.5) | 8 | IPW | -2.1 | -1.458 | 0.069 | -0.058 | 0.998 | 0.001 | 0.953 | 2.981 | 0.954 | 2.963 | 0.948 | 3.152 | 0.271 | 0.527 | 4.766 | 0.098 |
| | 8 | WIPW | -2.1 | 3.016 | 0.067 | 0.011 | 0.997 | 0.005 | 0.955 | 2.944 | 0.948 | 3.135 | 0.955 | 2.926 | 0.264 | 0.459 | 4.569 | 0.094 |
| | 8 | AIPW | -2.1 | -1.525 | 0.069 | -0.057 | 1.004 | 0.001 | 0.950 | 3.088 | 0.951 | 3.053 | 0.947 | 3.174 | 0.267 | 0.520 | 4.692 | 0.096 |
| | 8 | WAIPW | -2.1 | 2.758 | 0.067 | 0.010 | 1.004 | 0.005 | 0.949 | 3.106 | 0.945 | 3.224 | 0.954 | 2.969 | 0.263 | 0.452 | 4.577 | 0.095 |
| IAIPW(1) | 8 | IPW | -2.1 | -3.861 | 0.094 | -0.146 | 1.016 | -0.002 | 0.941 | 3.338 | 0.957 | 2.869 | 0.933 | 3.546 | 0.349 | 1.670 | 8.862 | 0.242 |
| | 8 | WIPW | -2.1 | 5.945 | 0.077 | -0.006 | 1.004 | 0.008 | 0.945 | 3.224 | 0.948 | 3.152 | 0.949 | 3.106 | 0.295 | 0.902 | 6.051 | 0.154 |
| | 8 | AIPW | -2.1 | -3.914 | 0.094 | -0.140 | 1.010 | -0.002 | 0.938 | 3.411 | 0.955 | 2.920 | 0.936 | 3.467 | 0.350 | 1.783 | 8.777 | 0.242 |
| | 8 | WAIPW | -2.1 | 5.785 | 0.078 | -0.004 | 1.013 | 0.008 | 0.944 | 3.257 | 0.946 | 3.202 | 0.945 | 3.219 | 0.295 | 0.923 | 6.083 | 0.154 |
| IPW(0.5) | 8 | IPW | -2.1 | -3.147 | 0.069 | -0.083 | 1.005 | -0.001 | 0.947 | 3.174 | 0.953 | 2.987 | 0.937 | 3.436 | 0.269 | 0.511 | 4.810 | 0.100 |
| | 8 | WIPW | -2.1 | 0.189 | 0.083 | -0.012 | 0.999 | 0.002 | 0.949 | 3.123 | 0.948 | 3.129 | 0.950 | 3.083 | 0.323 | 0.850 | 6.975 | 0.153 |
| | 8 | AIPW | -2.1 | -3.123 | 0.069 | -0.080 | 1.015 | -0.001 | 0.943 | 3.284 | 0.951 | 3.059 | 0.936 | 3.462 | 0.266 | 0.521 | 4.747 | 0.099 |
| | 8 | WAIPW | -2.1 | 1.509 | 0.068 | -0.009 | 1.015 | 0.004 | 0.943 | 3.274 | 0.948 | 3.140 | 0.942 | 3.295 | 0.262 | 0.457 | 4.625 | 0.097 |
| IPW(1) | 8 | IPW | -2.1 | -0.664 | 0.096 | -0.114 | 1.045 | 0.001 | 0.942 | 3.306 | 0.949 | 3.100 | 0.933 | 3.526 | 0.343 | 1.605 | 9.193 | 0.257 |
| | 8 | WIPW | -2.1 | 10.807 | 0.113 | 0.037 | 1.051 | 0.013 | 0.940 | 3.359 | 0.934 | 3.502 | 0.947 | 3.169 | 0.394 | 2.032 | 12.849 | 0.381 |
| | 8 | AIPW | -2.1 | -0.424 | 0.096 | -0.104 | 1.041 | 0.002 | 0.943 | 3.279 | 0.949 | 3.106 | 0.937 | 3.441 | 0.344 | 1.741 | 9.169 | 0.257 |
| | 8 | WAIPW | -2.1 | 7.907 | 0.079 | 0.023 | 1.030 | 0.010 | 0.947 | 3.180 | 0.940 | 3.359 | 0.950 | 3.071 | 0.293 | 0.912 | 6.315 | 0.158 |
| SR | 8 | IPW | -2.1 | -2.322 | 0.064 | -0.004 | 1.008 | 0.000 | 0.944 | 3.241 | 0.947 | 3.169 | 0.953 | 2.993 | 0.251 | 0.186 | 4.136 | 0.084 |

| Randomization Method | Regime | Estimator | True Value x 10^3 | Mean Estimate x 10^3 | SD Estimates x 10^3 | Mean Normalized | SD Normalized | Mean Bias | CI Coverage | SE CI Coverage x 10^3 | LB Coverage | SE LB Coverage x 10^3 | UB Coverage x 10^3 | SE UB Coverage x 10^3 | Mean CI Length | SE CI Length x 10^3 | MSE x 10^3 | SE MSE x 10^3 |
|---|---|---|---|---|---|---|---|---|---|---|---|---|---|---|---|---|---|---|
| | 8 | WIPW | -2.1 | -1.290 | 0.077 | 0.010 | 1.020 | 0.001 | 0.945 | 3.230 | 0.943 | 3.284 | 0.949 | 3.112 | 0.299 | 0.280 | 5.994 | 0.119 |
| | 8 | AIPW | -2.1 | -2.399 | 0.064 | -0.005 | 1.017 | 0.000 | 0.942 | 3.306 | 0.944 | 3.252 | 0.950 | 3.094 | 0.248 | 0.186 | 4.069 | 0.082 |
| | 8 | WAIPW | -2.1 | -2.285 | 0.064 | -0.003 | 1.017 | 0.000 | 0.944 | 3.241 | 0.945 | 3.219 | 0.947 | 3.180 | 0.250 | 0.190 | 4.152 | 0.084 |
| TS(0.25) | 8 | IPW | -2.1 | -3.224 | 0.081 | -0.049 | 1.009 | -0.001 | 0.948 | 3.146 | 0.955 | 2.944 | 0.943 | 3.268 | 0.278 | 1.685 | 6.566 | 0.280 |
| | 8 | WIPW | -2.1 | -1.204 | 0.067 | -0.019 | 1.002 | 0.001 | 0.948 | 3.140 | 0.952 | 3.023 | 0.950 | 3.094 | 0.259 | 0.476 | 4.502 | 0.101 |
| | 8 | AIPW | -2.1 | -3.313 | 0.085 | -0.048 | 1.002 | -0.001 | 0.950 | 3.088 | 0.954 | 2.951 | 0.947 | 3.180 | 0.284 | 2.527 | 7.257 | 0.403 |
| | 8 | WAIPW | -2.1 | -1.082 | 0.068 | -0.017 | 1.001 | 0.001 | 0.949 | 3.100 | 0.948 | 3.140 | 0.952 | 3.029 | 0.261 | 0.653 | 4.570 | 0.106 |
| TS(0.50) | 8 | IPW | -2.1 | 0.170 | 0.089 | -0.042 | 1.019 | 0.002 | 0.943 | 3.284 | 0.948 | 3.135 | 0.943 | 3.279 | 0.300 | 1.782 | 7.899 | 0.364 |
| | 8 | WIPW | -2.1 | 4.325 | 0.073 | 0.026 | 1.017 | 0.006 | 0.945 | 3.213 | 0.943 | 3.284 | 0.948 | 3.129 | 0.273 | 0.744 | 5.388 | 0.130 |
| | 8 | AIPW | -2.1 | -0.140 | 0.092 | -0.044 | 1.016 | 0.002 | 0.944 | 3.241 | 0.952 | 3.035 | 0.943 | 3.268 | 0.305 | 2.703 | 8.549 | 0.463 |
| | 8 | WAIPW | -2.1 | 4.071 | 0.074 | 0.023 | 1.023 | 0.006 | 0.944 | 3.246 | 0.943 | 3.274 | 0.947 | 3.157 | 0.276 | 0.930 | 5.587 | 0.144 |
| TS(0.75) | 8 | IPW | -2.1 | -2.777 | 0.102 | -0.099 | 1.035 | -0.001 | 0.943 | 3.290 | 0.951 | 3.047 | 0.936 | 3.457 | 0.340 | 2.357 | 10.348 | 0.390 |
| | 8 | WIPW | -2.1 | 3.558 | 0.081 | -0.020 | 1.026 | 0.006 | 0.947 | 3.169 | 0.945 | 3.230 | 0.941 | 3.322 | 0.292 | 1.094 | 6.530 | 0.184 |
| | 8 | AIPW | -2.1 | -2.587 | 0.107 | -0.100 | 1.022 | 0.000 | 0.946 | 3.208 | 0.955 | 2.944 | 0.936 | 3.472 | 0.351 | 3.369 | 11.553 | 0.719 |
| | 8 | WAIPW | -2.1 | 3.769 | 0.083 | -0.014 | 1.031 | 0.006 | 0.945 | 3.224 | 0.948 | 3.146 | 0.943 | 3.279 | 0.300 | 1.389 | 6.967 | 0.213 |
| TS(1) | 8 | IPW | -2.1 | -1.621 | 0.121 | -0.098 | 1.072 | 0.000 | 0.934 | 3.502 | 0.945 | 3.213 | 0.933 | 3.536 | 0.381 | 2.800 | 14.663 | 0.694 |
| | 8 | WIPW | -2.1 | 7.747 | 0.087 | 0.010 | 1.022 | 0.010 | 0.944 | 3.241 | 0.940 | 3.348 | 0.947 | 3.157 | 0.312 | 1.417 | 7.725 | 0.225 |
| | 8 | AIPW | -2.1 | -4.118 | 0.181 | -0.084 | 1.041 | -0.002 | 0.940 | 3.364 | 0.951 | 3.047 | 0.940 | 3.359 | 0.407 | 8.240 | 32.912 | 13.212 |
| | 8 | WAIPW | -2.1 | 8.690 | 0.091 | 0.030 | 1.029 | 0.011 | 0.940 | 3.369 | 0.939 | 3.390 | 0.949 | 3.106 | 0.322 | 1.817 | 8.353 | 0.254 |
| WAIPW(0.5) | 8 | IPW | -2.1 | -0.957 | 0.070 | -0.049 | 1.008 | 0.001 | 0.948 | 3.152 | 0.953 | 2.987 | 0.948 | 3.140 | 0.270 | 0.525 | 4.870 | 0.111 |
| | 8 | WIPW | -2.1 | 3.341 | 0.068 | 0.018 | 1.004 | 0.005 | 0.949 | 3.123 | 0.946 | 3.197 | 0.954 | 2.975 | 0.263 | 0.460 | 4.642 | 0.106 |
| | 8 | AIPW | -2.1 | -0.865 | 0.069 | -0.046 | 1.020 | 0.001 | 0.946 | 3.202 | 0.949 | 3.123 | 0.944 | 3.241 | 0.267 | 0.520 | 4.817 | 0.109 |
| | 8 | WAIPW | -2.1 | 3.359 | 0.068 | 0.019 | 1.015 | 0.005 | 0.946 | 3.186 | 0.945 | 3.235 | 0.952 | 3.023 | 0.263 | 0.457 | 4.664 | 0.107 |
| WAIPW(1) | 8 | IPW | -2.1 | -2.050 | 0.093 | -0.124 | 1.024 | 0.000 | 0.941 | 3.343 | 0.954 | 2.951 | 0.933 | 3.531 | 0.341 | 1.682 | 8.632 | 0.249 |
| | 8 | WIPW | -2.1 | 7.135 | 0.078 | 0.005 | 1.013 | 0.009 | 0.945 | 3.219 | 0.945 | 3.230 | 0.945 | 3.213 | 0.293 | 0.957 | 6.110 | 0.151 |
| | 8 | AIPW | -2.1 | -2.137 | 0.092 | -0.120 | 1.019 | 0.000 | 0.944 | 3.252 | 0.957 | 2.863 | 0.935 | 3.497 | 0.343 | 1.812 | 8.547 | 0.247 |
| | 8 | WAIPW | -2.1 | 7.083 | 0.078 | 0.004 | 1.022 | 0.009 | 0.942 | 3.306 | 0.943 | 3.279 | 0.947 | 3.180 | 0.293 | 0.986 | 6.165 | 0.153 |
| WIPW(0.5) | 8 | IPW | -2.1 | -1.411 | 0.069 | -0.058 | 1.006 | 0.001 | 0.947 | 3.169 | 0.956 | 2.894 | 0.942 | 3.306 | 0.270 | 0.516 | 4.817 | 0.108 |
| | 8 | WIPW | -2.1 | 3.250 | 0.068 | 0.014 | 1.007 | 0.005 | 0.947 | 3.169 | 0.952 | 3.035 | 0.950 | 3.077 | 0.264 | 0.452 | 4.630 | 0.104 |
| | 8 | AIPW | -2.1 | -1.300 | 0.069 | -0.054 | 1.011 | 0.001 | 0.946 | 3.191 | 0.954 | 2.957 | 0.942 | 3.317 | 0.267 | 0.524 | 4.721 | 0.105 |
| | 8 | WAIPW | -2.1 | 3.184 | 0.068 | 0.015 | 1.014 | 0.005 | 0.948 | 3.140 | 0.950 | 3.077 | 0.949 | 3.100 | 0.263 | 0.463 | 4.621 | 0.103 |
| WIPW(1) | 8 | IPW | -2.1 | -1.531 | 0.095 | -0.125 | 1.043 | 0.001 | 0.939 | 3.395 | 0.954 | 2.951 | 0.933 | 3.541 | 0.342 | 1.643 | 9.011 | 0.242 |
| | 8 | WIPW | -2.1 | 7.623 | 0.079 | 0.009 | 1.024 | 0.010 | 0.947 | 3.180 | 0.945 | 3.224 | 0.949 | 3.112 | 0.294 | 0.926 | 6.314 | 0.154 |
| | 8 | AIPW | -2.1 | -1.234 | 0.095 | -0.116 | 1.037 | 0.001 | 0.943 | 3.268 | 0.954 | 2.957 | 0.935 | 3.477 | 0.342 | 1.782 | 8.970 | 0.246 |
| | 8 | WAIPW | -2.1 | 7.454 | 0.079 | 0.011 | 1.029 | 0.010 | 0.944 | 3.252 | 0.944 | 3.263 | 0.949 | 3.117 | 0.294 | 0.968 | 6.302 | 0.152 |

Identifying the Optimal Regime Results for the Cancer Pain Management SMART, Continuous Outcome, Similar Mean Outcomes Scenario

| Randomization Method | Estimator | Proportion Estimating d1 Optimal | SE Proprtion d1 x 10^3 | Proportion Estimating d2 Optimal | SE Proprtion d2 x 10^3 | Proportion Estimating d3 Optimal | SE Proprtion d3 x 10^3 | Proportion Estimating d4 Optimal | SE Proprtion d4 x 10^3 | Proportion Estimating d5 Optimal | SE Proprtion d5 x 10^3 | Proportion Estimating d6 Optimal | SE Proprtion d6 x 10^3 | Proportion Estimating d7 Optimal | SE Proprtion d7 x 10^3 | Proportion Estimating d8 Optimal | SE Proprtion d8 x 10^3 |
|---|---|---|---|---|---|---|---|---|---|---|---|---|---|---|---|---|---|
| AIPW(0.5) | IPW | 0.128 | 4.719 | 0.124 | 4.658 | 0.127 | 4.706 | 0.126 | 4.694 | 0.121 | 4.606 | 0.125 | 4.674 | 0.130 | 4.757 | 0.120 | 4.603 |
| | WIPW | 0.130 | 4.753 | 0.125 | 4.684 | 0.126 | 4.700 | 0.132 | 4.781 | 0.115 | 4.505 | 0.118 | 4.570 | 0.124 | 4.655 | 0.130 | 4.760 |
| | AIPW | 0.125 | 4.678 | 0.120 | 4.593 | 0.127 | 4.703 | 0.130 | 4.753 | 0.121 | 4.616 | 0.125 | 4.671 | 0.129 | 4.735 | 0.124 | 4.668 |
| | WAIPW | 0.129 | 4.744 | 0.121 | 4.606 | 0.124 | 4.668 | 0.126 | 4.690 | 0.125 | 4.671 | 0.124 | 4.668 | 0.133 | 4.809 | 0.118 | 4.556 |
| AIPW(1) | IPW | 0.120 | 4.599 | 0.126 | 4.687 | 0.127 | 4.716 | 0.124 | 4.655 | 0.130 | 4.757 | 0.134 | 4.815 | 0.119 | 4.580 | 0.120 | 4.603 |

| Randomization Method | Estimator | Proportion Estimating d1 Optimal | SE Proprtion d1 x 10^3 | Proportion Estimating d2 Optimal | SE Proprtion d2 x 10^3 | Proportion Estimating d3 Optimal | SE Proprtion d3 x 10^3 | Proportion Estimating d4 Optimal | SE Proprtion d4 x 10^3 | Proportion Estimating d5 Optimal | SE Proprtion d5 x 10^3 | Proportion Estimating d6 Optimal | SE Proprtion d6 x 10^3 | Proportion Estimating d7 Optimal | SE Proprtion d7 x 10^3 | Proportion Estimating d8 Optimal | SE Proprtion d8 x 10^3 |
|---|---|---|---|---|---|---|---|---|---|---|---|---|---|---|---|---|---|
| | WIPW | 0.120 | 4.599 | 0.127 | 4.709 | 0.120 | 4.599 | 0.126 | 4.697 | 0.129 | 4.738 | 0.133 | 4.797 | 0.127 | 4.703 | 0.118 | 4.570 |
| | AIPW | 0.129 | 4.735 | 0.125 | 4.671 | 0.121 | 4.619 | 0.123 | 4.639 | 0.128 | 4.728 | 0.136 | 4.842 | 0.119 | 4.573 | 0.120 | 4.603 |
| | WAIPW | 0.123 | 4.652 | 0.125 | 4.674 | 0.124 | 4.665 | 0.124 | 4.658 | 0.130 | 4.763 | 0.132 | 4.794 | 0.120 | 4.603 | 0.121 | 4.606 |
| AR-1 | IPW | 0.126 | 4.690 | 0.134 | 4.812 | 0.123 | 4.652 | 0.129 | 4.738 | 0.120 | 4.589 | 0.113 | 4.478 | 0.129 | 4.744 | 0.127 | 4.703 |
| | WIPW | 0.131 | 4.769 | 0.122 | 4.629 | 0.111 | 4.436 | 0.150 | 5.053 | 0.114 | 4.502 | 0.110 | 4.418 | 0.127 | 4.706 | 0.136 | 4.842 |
| | AIPW | 0.130 | 4.750 | 0.138 | 4.884 | 0.125 | 4.684 | 0.130 | 4.753 | 0.116 | 4.529 | 0.110 | 4.432 | 0.126 | 4.697 | 0.124 | 4.665 |
| | WAIPW | 0.133 | 4.803 | 0.129 | 4.747 | 0.110 | 4.432 | 0.142 | 4.937 | 0.120 | 4.596 | 0.108 | 4.390 | 0.126 | 4.697 | 0.131 | 4.772 |
| AR-2 | IPW | 0.132 | 4.794 | 0.137 | 4.857 | 0.125 | 4.684 | 0.133 | 4.809 | 0.123 | 4.652 | 0.113 | 4.485 | 0.111 | 4.446 | 0.124 | 4.665 |
| | WIPW | 0.139 | 4.890 | 0.124 | 4.658 | 0.113 | 4.471 | 0.136 | 4.848 | 0.131 | 4.778 | 0.107 | 4.365 | 0.115 | 4.505 | 0.136 | 4.851 |
| | AIPW | 0.137 | 4.857 | 0.132 | 4.794 | 0.122 | 4.622 | 0.132 | 4.791 | 0.127 | 4.713 | 0.115 | 4.505 | 0.112 | 4.457 | 0.124 | 4.655 |
| | WAIPW | 0.139 | 4.896 | 0.127 | 4.706 | 0.113 | 4.471 | 0.134 | 4.818 | 0.126 | 4.697 | 0.111 | 4.439 | 0.113 | 4.485 | 0.137 | 4.863 |
| IAIPW(0.5) | IPW | 0.119 | 4.583 | 0.125 | 4.684 | 0.123 | 4.649 | 0.134 | 4.821 | 0.128 | 4.725 | 0.126 | 4.687 | 0.128 | 4.722 | 0.117 | 4.539 |
| | WIPW | 0.122 | 4.626 | 0.124 | 4.668 | 0.125 | 4.684 | 0.128 | 4.725 | 0.126 | 4.687 | 0.127 | 4.703 | 0.128 | 4.722 | 0.120 | 4.603 |
| | AIPW | 0.116 | 4.526 | 0.125 | 4.674 | 0.119 | 4.583 | 0.139 | 4.890 | 0.130 | 4.760 | 0.122 | 4.629 | 0.128 | 4.728 | 0.121 | 4.613 |
| | WAIPW | 0.120 | 4.599 | 0.125 | 4.681 | 0.119 | 4.580 | 0.128 | 4.719 | 0.127 | 4.706 | 0.127 | 4.703 | 0.130 | 4.760 | 0.124 | 4.668 |
| IAIPW(1) | IPW | 0.132 | 4.784 | 0.119 | 4.580 | 0.123 | 4.642 | 0.127 | 4.709 | 0.120 | 4.593 | 0.130 | 4.750 | 0.124 | 4.655 | 0.126 | 4.700 |
| | WIPW | 0.128 | 4.719 | 0.116 | 4.529 | 0.123 | 4.639 | 0.122 | 4.622 | 0.129 | 4.738 | 0.131 | 4.778 | 0.130 | 4.750 | 0.122 | 4.636 |
| | AIPW | 0.125 | 4.681 | 0.127 | 4.703 | 0.118 | 4.559 | 0.125 | 4.671 | 0.123 | 4.649 | 0.129 | 4.741 | 0.129 | 4.744 | 0.124 | 4.668 |
| | WAIPW | 0.125 | 4.678 | 0.116 | 4.536 | 0.120 | 4.596 | 0.123 | 4.639 | 0.127 | 4.709 | 0.135 | 4.833 | 0.129 | 4.738 | 0.125 | 4.681 |
| IPW(0.5) | IPW | 0.115 | 4.516 | 0.121 | 4.609 | 0.117 | 4.539 | 0.129 | 4.735 | 0.127 | 4.703 | 0.130 | 4.753 | 0.137 | 4.860 | 0.126 | 4.687 |
| | WIPW | 0.124 | 4.655 | 0.114 | 4.502 | 0.122 | 4.622 | 0.121 | 4.619 | 0.131 | 4.775 | 0.129 | 4.741 | 0.131 | 4.769 | 0.128 | 4.725 |
| | AIPW | 0.109 | 4.404 | 0.121 | 4.609 | 0.125 | 4.671 | 0.127 | 4.703 | 0.127 | 4.703 | 0.129 | 4.747 | 0.139 | 4.896 | 0.124 | 4.661 |
| | WAIPW | 0.111 | 4.450 | 0.122 | 4.632 | 0.123 | 4.649 | 0.125 | 4.671 | 0.126 | 4.697 | 0.132 | 4.794 | 0.135 | 4.836 | 0.125 | 4.674 |
| IPW(1) | IPW | 0.121 | 4.613 | 0.131 | 4.775 | 0.117 | 4.553 | 0.123 | 4.652 | 0.124 | 4.665 | 0.129 | 4.741 | 0.130 | 4.763 | 0.123 | 4.652 |
| | WIPW | 0.129 | 4.735 | 0.127 | 4.706 | 0.123 | 4.649 | 0.123 | 4.639 | 0.121 | 4.613 | 0.124 | 4.665 | 0.130 | 4.757 | 0.124 | 4.655 |
| | AIPW | 0.123 | 4.649 | 0.136 | 4.842 | 0.118 | 4.563 | 0.120 | 4.599 | 0.124 | 4.655 | 0.131 | 4.775 | 0.123 | 4.642 | 0.125 | 4.684 |
| | WAIPW | 0.121 | 4.619 | 0.129 | 4.741 | 0.117 | 4.549 | 0.123 | 4.645 | 0.130 | 4.753 | 0.128 | 4.732 | 0.123 | 4.645 | 0.128 | 4.728 |
| SR | IPW | 0.123 | 4.639 | 0.123 | 4.649 | 0.111 | 4.443 | 0.125 | 4.674 | 0.127 | 4.713 | 0.131 | 4.775 | 0.129 | 4.744 | 0.131 | 4.769 |
| | WIPW | 0.122 | 4.629 | 0.119 | 4.586 | 0.121 | 4.619 | 0.128 | 4.728 | 0.129 | 4.735 | 0.131 | 4.766 | 0.124 | 4.661 | 0.126 | 4.690 |
| | AIPW | 0.119 | 4.576 | 0.126 | 4.690 | 0.118 | 4.563 | 0.124 | 4.658 | 0.125 | 4.681 | 0.130 | 4.753 | 0.128 | 4.719 | 0.131 | 4.772 |
| | WAIPW | 0.122 | 4.629 | 0.127 | 4.716 | 0.114 | 4.495 | 0.123 | 4.642 | 0.129 | 4.735 | 0.128 | 4.732 | 0.126 | 4.697 | 0.131 | 4.766 |
| TS(0.25) | IPW | 0.117 | 4.539 | 0.123 | 4.639 | 0.126 | 4.697 | 0.123 | 4.649 | 0.126 | 4.690 | 0.129 | 4.735 | 0.120 | 4.599 | 0.137 | 4.860 |
| | WIPW | 0.119 | 4.576 | 0.117 | 4.539 | 0.126 | 4.694 | 0.131 | 4.766 | 0.131 | 4.775 | 0.131 | 4.775 | 0.115 | 4.512 | 0.131 | 4.766 |
| | AIPW | 0.114 | 4.502 | 0.121 | 4.606 | 0.126 | 4.700 | 0.127 | 4.716 | 0.130 | 4.760 | 0.128 | 4.728 | 0.118 | 4.559 | 0.135 | 4.833 |
| | WAIPW | 0.115 | 4.505 | 0.117 | 4.553 | 0.125 | 4.684 | 0.129 | 4.744 | 0.134 | 4.824 | 0.131 | 4.778 | 0.115 | 4.509 | 0.133 | 4.800 |
| TS(0.50) | IPW | 0.137 | 4.860 | 0.111 | 4.439 | 0.132 | 4.787 | 0.125 | 4.671 | 0.133 | 4.806 | 0.120 | 4.596 | 0.124 | 4.665 | 0.118 | 4.570 |
| | WIPW | 0.137 | 4.863 | 0.096 | 4.163 | 0.136 | 4.848 | 0.140 | 4.913 | 0.148 | 5.020 | 0.125 | 4.671 | 0.105 | 4.328 | 0.114 | 4.492 |
| | AIPW | 0.139 | 4.893 | 0.109 | 4.404 | 0.131 | 4.775 | 0.127 | 4.713 | 0.133 | 4.797 | 0.121 | 4.613 | 0.119 | 4.580 | 0.121 | 4.616 |
| | WAIPW | 0.129 | 4.738 | 0.097 | 4.178 | 0.136 | 4.845 | 0.138 | 4.884 | 0.147 | 5.005 | 0.129 | 4.738 | 0.110 | 4.418 | 0.115 | 4.516 |
| TS(0.75) | IPW | 0.127 | 4.706 | 0.118 | 4.570 | 0.126 | 4.687 | 0.110 | 4.422 | 0.132 | 4.781 | 0.121 | 4.609 | 0.136 | 4.848 | 0.131 | 4.772 |
| | WIPW | 0.120 | 4.596 | 0.095 | 4.151 | 0.133 | 4.806 | 0.126 | 4.694 | 0.157 | 5.151 | 0.127 | 4.713 | 0.120 | 4.589 | 0.121 | 4.619 |
| | AIPW | 0.127 | 4.709 | 0.120 | 4.589 | 0.120 | 4.589 | 0.112 | 4.467 | 0.136 | 4.845 | 0.116 | 4.526 | 0.139 | 4.890 | 0.131 | 4.772 |
| | WAIPW | 0.117 | 4.539 | 0.092 | 4.096 | 0.134 | 4.824 | 0.126 | 4.700 | 0.158 | 5.159 | 0.129 | 4.738 | 0.120 | 4.589 | 0.124 | 4.658 |
| TS(1) | IPW | 0.130 | 4.753 | 0.117 | 4.543 | 0.126 | 4.690 | 0.119 | 4.580 | 0.132 | 4.784 | 0.126 | 4.694 | 0.136 | 4.851 | 0.115 | 4.505 |

| Randomization Method | Estimator | Proportion Estimating d1 Optimal | SE Proprtion d1 x 10^3 | Proportion Estimating d2 Optimal | SE Proprtion d2 x 10^3 | Proportion Estimating d3 Optimal | SE Proprtion d3 x 10^3 | Proportion Estimating d4 Optimal | SE Proprtion d4 x 10^3 | Proportion Estimating d5 Optimal | SE Proprtion d5 x 10^3 | Proportion Estimating d6 Optimal | SE Proprtion d6 x 10^3 | Proportion Estimating d7 Optimal | SE Proprtion d7 x 10^3 | Proportion Estimating d8 Optimal | SE Proprtion d8 x 10^3 |
|---|---|---|---|---|---|---|---|---|---|---|---|---|---|---|---|---|---|
| | WIPW | 0.121 | 4.619 | 0.083 | 3.898 | 0.143 | 4.951 | 0.144 | 4.960 | 0.159 | 5.175 | 0.142 | 4.943 | 0.105 | 4.339 | 0.102 | 4.288 |
| | AIPW | 0.137 | 4.869 | 0.112 | 4.460 | 0.124 | 4.655 | 0.122 | 4.632 | 0.131 | 4.778 | 0.125 | 4.674 | 0.130 | 4.757 | 0.119 | 4.573 |
| | WAIPW | 0.122 | 4.626 | 0.083 | 3.906 | 0.143 | 4.954 | 0.144 | 4.966 | 0.158 | 5.164 | 0.149 | 5.042 | 0.104 | 4.314 | 0.096 | 4.170 |
| WAIPW(0.5) | IPW | 0.122 | 4.629 | 0.128 | 4.732 | 0.126 | 4.687 | 0.120 | 4.596 | 0.123 | 4.649 | 0.133 | 4.800 | 0.123 | 4.645 | 0.125 | 4.678 |
| | WIPW | 0.121 | 4.616 | 0.124 | 4.668 | 0.127 | 4.706 | 0.117 | 4.539 | 0.130 | 4.760 | 0.130 | 4.750 | 0.127 | 4.706 | 0.124 | 4.668 |
| | AIPW | 0.122 | 4.626 | 0.133 | 4.806 | 0.123 | 4.649 | 0.113 | 4.471 | 0.125 | 4.681 | 0.135 | 4.827 | 0.127 | 4.703 | 0.123 | 4.642 |
| | WAIPW | 0.124 | 4.661 | 0.134 | 4.815 | 0.125 | 4.674 | 0.114 | 4.502 | 0.128 | 4.719 | 0.127 | 4.706 | 0.124 | 4.668 | 0.124 | 4.665 |
| WAIPW(1) | IPW | 0.119 | 4.576 | 0.126 | 4.690 | 0.125 | 4.678 | 0.125 | 4.684 | 0.126 | 4.687 | 0.122 | 4.629 | 0.130 | 4.763 | 0.127 | 4.709 |
| | WIPW | 0.124 | 4.658 | 0.120 | 4.593 | 0.125 | 4.674 | 0.125 | 4.684 | 0.128 | 4.725 | 0.123 | 4.649 | 0.128 | 4.728 | 0.127 | 4.706 |
| | AIPW | 0.118 | 4.559 | 0.128 | 4.722 | 0.129 | 4.738 | 0.122 | 4.632 | 0.124 | 4.658 | 0.120 | 4.593 | 0.134 | 4.821 | 0.126 | 4.687 |
| | WAIPW | 0.125 | 4.671 | 0.120 | 4.599 | 0.125 | 4.684 | 0.124 | 4.655 | 0.127 | 4.713 | 0.123 | 4.652 | 0.129 | 4.744 | 0.126 | 4.700 |
| WIPW(0.5) | IPW | 0.122 | 4.626 | 0.125 | 4.671 | 0.126 | 4.700 | 0.119 | 4.586 | 0.122 | 4.636 | 0.134 | 4.824 | 0.126 | 4.687 | 0.125 | 4.684 |
| | WIPW | 0.120 | 4.589 | 0.124 | 4.665 | 0.127 | 4.706 | 0.123 | 4.642 | 0.127 | 4.713 | 0.130 | 4.763 | 0.125 | 4.681 | 0.124 | 4.658 |
| | AIPW | 0.116 | 4.536 | 0.127 | 4.703 | 0.126 | 4.700 | 0.122 | 4.636 | 0.126 | 4.700 | 0.131 | 4.766 | 0.125 | 4.674 | 0.126 | 4.700 |
| | WAIPW | 0.118 | 4.566 | 0.125 | 4.684 | 0.127 | 4.703 | 0.121 | 4.616 | 0.127 | 4.713 | 0.131 | 4.775 | 0.125 | 4.678 | 0.125 | 4.681 |
| WIPW(1) | IPW | 0.121 | 4.619 | 0.130 | 4.750 | 0.117 | 4.546 | 0.122 | 4.622 | 0.123 | 4.649 | 0.129 | 4.744 | 0.132 | 4.781 | 0.126 | 4.700 |
| | WIPW | 0.123 | 4.642 | 0.119 | 4.583 | 0.118 | 4.556 | 0.120 | 4.589 | 0.132 | 4.784 | 0.128 | 4.728 | 0.134 | 4.821 | 0.127 | 4.703 |
| | AIPW | 0.124 | 4.655 | 0.125 | 4.674 | 0.117 | 4.553 | 0.122 | 4.622 | 0.126 | 4.687 | 0.130 | 4.763 | 0.128 | 4.725 | 0.129 | 4.735 |
| | WAIPW | 0.121 | 4.616 | 0.122 | 4.629 | 0.118 | 4.570 | 0.121 | 4.613 | 0.130 | 4.763 | 0.131 | 4.775 | 0.130 | 4.760 | 0.126 | 4.687 |

Regime 1 Normalized Estimates for WAIPW(1) Randomization

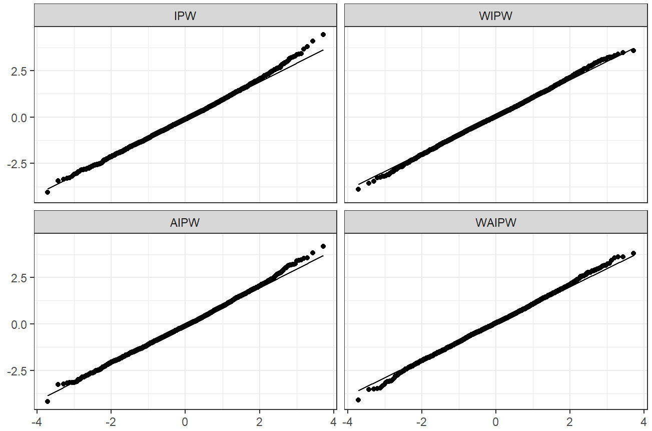


Regime 1 Normalized Estimates for TS(1) Randomization

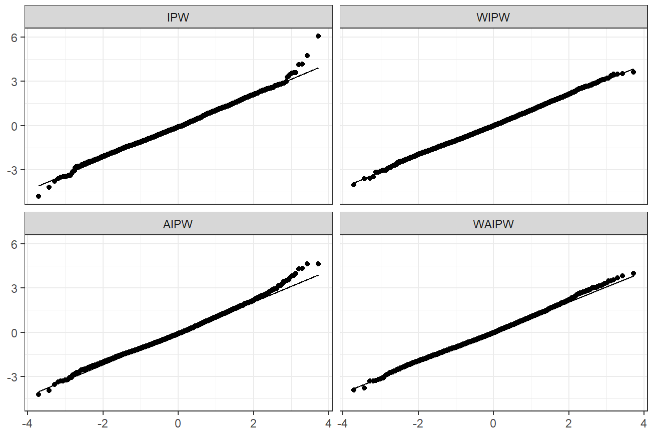


Regime 8 Normalized Estimates for WAIPW(1) Randomization

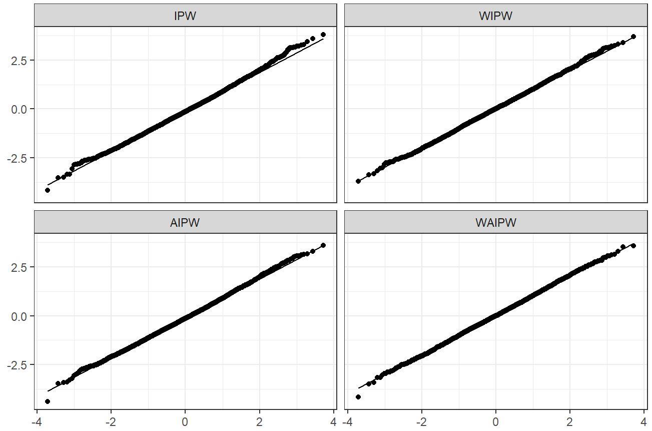

Regime 8 Normalized Estimates for TS(1) Randomization

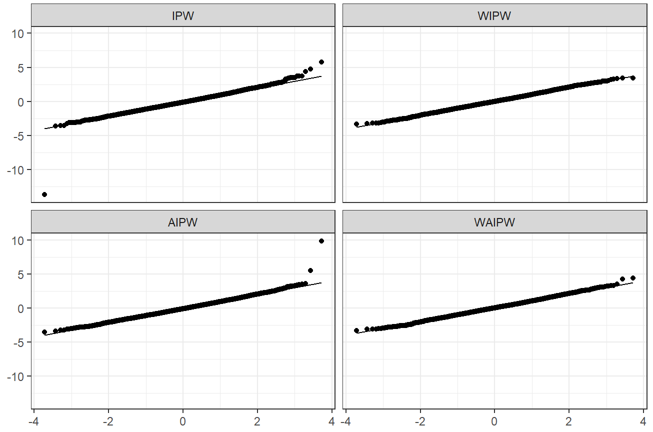


## C.3 Cancer Pain Management SMART, Continuous Outcome, Equal Mean Outcomes (Null Scenario)

### C.3.1 Simulation Details

This simulation study is the same as in Section C.1 and C.2 except for the data generating process. We describe it in full below.

We present results of a simulation study involving 5000 Monte Carlo trials under a scenario mimicking the cancer pain SMART introduced in Section 1.
Each trial enrolls $N = 1000$ subjects, with enrollment times uniform over (integer) weeks 1-24. Upon enrollment, we draw baseline pain score $X_1 \sim N(5, 1)$ and assign stage 1 treatment $A_1 \in \square_1 = \{0, 1\}$. Six weeks after $A_1$ is assigned, second-stage pain score is generated as $X_{2,1} = \gamma_{1,0} + \gamma_{1,1}X_1 + \gamma_{1,2}A_1 + \varepsilon_1$, where $\varepsilon_1 \sim N(0, 1)$, and response status after the first stage is $X_{2,2} = I(X_{2,1} < 0.7X_1)$, which, with $A_1$, dictates the feasible subset of $\square_2 = \{0, 1, 2, 3, 4, 5\}$ from which stage 2 treatment $A_2$ is assigned. Six weeks later, the outcome is generated as $Y = \gamma_{2,0} + \gamma_{2,1}X_1 + \gamma_{2,2}A_1 + \gamma_{2,3}X_{2,1} + \gamma_{2,4}I(A_2 = 1) + \gamma_{2,5}I(A_2 = 2 \text{ or } 5) + \gamma_{2,6}I(A_2 = 3) + \gamma_{2,7}I(A_2 = 4) + \varepsilon_2$, where $\varepsilon_2 \sim N(0, 1)$. With $\gamma_1 = (\gamma_{1,0}, \gamma_{1,1}, \gamma_{1,2})^T = (0.00, 0.90, 0.00)^T$ and $\gamma_2 = (\gamma_{2,0}, \ldots, \gamma_{2,7}) = (0.00, 0.05, 0.00, 0.10, 0.00, 0.00, 0.00, 0.00)^T$, for the $m = 8$ embedded regimes defined in Figure 1,
$\{\square(\mathbf{d}^1), \ldots, \square(\mathbf{d}^8)\} = (\theta_1, \ldots, \theta_8) = (-0.002, -0.002, -0.002, -0.002, -0.002, -0.002, -0.002, -0.002).$

The burn-in period ends at the time $t^*$ when each of the $m = 8$ regimes has at least 25 subjects who have completed the trial with experience consistent with following the regime.

For demonstration, we randomize via WAIPW(0.5) and TS(0.5). The models used for both are the same as previous simulations.

At each week, for each RAR method, newly-enrolled subjects are assigned stage 1 treatment using the same randomization probability. Already-enrolled subjects who have reached stage 2 at this week and require stage 2 randomization are partitioned into four groups based on $(a_1, x_{2,2}) = (0, 0), (0, 1), (1, 0), (1, 1)$. Within each group, randomization probabilities are calculated; thus, second-stage probabilities are specific to each stage 1 treatment-response status combination. For both AR methods, we use the sample average of $X_1$ to calculate the the components that make up the stage 2 probabilities.

For both randomization methods, we test two null hypothesis: (i) $\square(\mathbf{d}^8) = \square(\mathbf{d}^1)$ and (ii) $\square(\mathbf{d}^1) = \square(\mathbf{d}^2) = \cdots = \square(\mathbf{d}^8)$. The former is tested with a contrast and Z-test and the latter is tested with a chi-square test. We use a chi-square distribution with 5 degrees of freedom rather than 8 because, as noted by Wu, Want, and Wahed (2023), there is a linear depedency among the four regimes starting with the same stage 1 treatment.

Contrast and Chi-Sq Test Results in the Fully-Null Scenario

| Randomization Method | Estimator | Mean Contrast Z | SD Contrast Z | Type One Error Contrast | Type One Error Chi-Sq |
|---|---|---|---|---|---|
| TS(0.5) | AIPW | -0.015 | 1.011 | 0.048 | 0.066 |
| | IPW | -0.009 | 1.019 | 0.051 | 0.057 |
| | WAIPW | 0.007 | 1.013 | 0.051 | 0.064 |
| | WIPW | 0.010 | 1.007 | 0.053 | 0.055 |
| WAIPW(0.5) | AIPW | 0.021 | 0.992 | 0.048 | 0.052 |
| | IPW | 0.022 | 0.989 | 0.049 | 0.046 |
| | WAIPW | 0.026 | 0.998 | 0.051 | 0.050 |
| | WIPW | 0.024 | 0.996 | 0.051 | 0.046 |

# C.4 Cancer Pain Management SMART, Binary Outcome, Different Mean Outcomes

## C.4.1 Simulation Details

We present results of a simulation study involving 5000 Monte Carlo trials under a scenario similar to the cancer pain SMART introduced in Section 1.
Each trial enrolls $N = 1000$ subjects, with enrollment times uniform over (integer) weeks 1-24. Upon enrollment, we draw baseline pain score $X_1 \sim N(5, 1)$ and assign stage 1 treatment $A_1 \in \square_1 = \{0, 1\}$. Six weeks after $A_1$ is assigned, second-stage pain score is generated as $X_{2,1} = \gamma_{1,0} + \gamma_{1,1}X_1 + \gamma_{1,2}A_1 + \varepsilon_1$, where $\varepsilon_1 \sim N(0, 1)$, and response status after the first stage is $X_{2,2} = I(X_{2,1} < 0.7X_1)$, which, with $A_1$, dictates the feasible subset of $\square_2 = \{0, 1, 2, 3, 4, 5\}$ from which stage 2 treatment $A_2$ is assigned. Six weeks later, the binary outcome is generated as $E(Y) = \mu\{\gamma_{2,0} + \gamma_{2,1}X_1 + \gamma_{2,2}A_1 + \gamma_{2,3}X_{2,1} + \gamma_{2,4}I(A_2 = 1) + \gamma_{2,5}I(A_2 = 2 \text{ or } 5) + \gamma_{2,6}I(A_2 = 3) + \gamma_{2,7}I(A_2 = 4)\}$, where $\mu(x) = e^x/(1 + e^x)$. Here $Y = 1$ is the more favorable outcome. With $\gamma_1 = (\gamma_{1,0}, \gamma_{1,1}, \gamma_{1,2})^T = (0.00, 0.90, -0.001)^T$ and $\gamma_2 = (\gamma_{2,0}, \dots, \gamma_{2,7}) = c(0.000, 0.100, 0.100, 0.100, 0.250, 0.100, 0.100, 0.500)^T$, for the $m = 8$ embedded regimes defined in Figure 1, $\{\square(\mathbf{d}^1), \dots, \square(\mathbf{d}^8)\} = (\theta_1, \dots, \theta_8) = (0.531, 0.513, 0.544, 0.562, 0.519, 0.513, 0.603, 0.609)$. Regime 8s is the optimal regime.

The burn-in period ends at the time $t^*$ when each of the $m = 8$ regimes has at least 50 subjects who have completed the trial with experience consistent with following the regime.

We compare the performance of up-front RAR using TS with $c_t = 0.5$ for all $t$ based on the WIPW estimator. For the AIPW, WAIPW and IAIPW estimators,
$Q_2(\overline{\mathbf{X}_2}, \overline{\mathbf{a}_2}; \beta_2) = \mu\{\beta_{2,0} + \beta_{2,1}x_1 + \beta_{2,2}I(a_1 = 1) + \beta_{2,3}x_{2,1} + \beta_{2,4}I(a_2 = 1) + \beta_{2,5}I(a_2 = 2 \text{ or } 5) + \beta_{2,6}I(a_2 = 3) + \beta_{2,7}I(a_2 = 4)\}$ and $Q_1^j(x_1, a_1; \beta_1^j) = \beta_{1,0}^j + \beta_{1,1}^j x_1 + \beta_{1,2}^j a_1 + \beta_{1,3}^j x_1 a_1$. Note that the stage 2 model is a logistic regression model and the stage 1 model is a linear model with the log-odds as the psuedo-outcome.

The sequential methods include Q-learning-based sequential RAR approach using TS with $c_t = 0.50$ To implement all RAR methods, we posit a logistic regression model
$Q_2(\overline{\mathbf{X}_2}, \overline{\mathbf{a}_2}; \beta_2) = \mu\{\beta_{2,0} + \beta_{2,1}x_1 + \beta_{2,2}I(a_1 = 1) + \beta_{2,3}x_{2,1} + \beta_{2,4}I(a_2 = 1) + \beta_{2,5}I(a_2 = 2 \text{ or } 5) + \beta_{2,6}I(a_2 = 3) + \beta_{2,7}I(a_2 = 4)\}$ and a linear model with the log-odds as the psuedo outcome $Q_1(x_1, a_1; \beta_1) = \beta_{1,0} + \beta_{1,1}x_1 + \beta_{1,2}a_1$. For sequential RAR, we set $B_1 = b_2 \times b_1 = 32 \times 32 = 1024$ and $B_2 = 1000$. The tuning parameters for AR-1 are $b = 10, \tau = 0.5$ and $b = 100, \tau = 0.025$ for AR-2 and $\lambda_t = t^{-1}\tau^{(1-b)}$ for both. A clipping constant of 0.05 was imposed for the sequential methods.

At each week, for each RAR method, newly-enrolled subjects are assigned stage 1 treatment using the same randomization probability. Already-enrolled subjects who have reached stage 2 at this week and require stage 2 randomization are partitioned into four groups based on $(a_1, x_{2,2}) = (0,0), (0,1), (1,0), (1,1)$. Within each group, randomization probabilities are calculated; thus, second-stage probabilities are specific to each stage 1 treatment-response status combination.

Tables and figures in the following sections are as in Section C.1.

## C.4.2 In Trial Results

In Trial Results for the Binary Outcome Scenario

| Randomization Method | Mean Y | SE Mean Y x 10^3 | Mean Proportion A1=1 | SE Mean Proportion A1=1 x 10^3 | Mean Proportion Cd8=1 | SE Mean Proportion Cd8=1 x 10^3 | Mean Proportion Cd7=1 or Cd8=1 | SE Mean Proportion Cd7=1 or Cd8=1 x 10^3 |
|---|---|---|---|---|---|---|---|---|
| TS(0.5) | 0.559 | 0.236 | 0.491 | 1.195 | 0.325 | 1.099 | 0.341 | 1.157 |
| WAIPW(0.5) | 0.557 | 0.228 | 0.539 | 0.913 | 0.337 | 0.910 | 0.354 | 0.932 |

## C.4.3 Estimation Results

Estimation Results for the Binary Outcome Scenario

| Randomization Method | Regime | Estimator | True Value | Mean Estimate | SD Estimates | Mean Normalized | SD Normalized | Mean Bias | CI Coverage | SE CI Coverage x 10^3 | LB Coverage | SE LB Coverage x 10^3 | UB Coverage | SE UB Coverage x 10^3 | Mean CI Length | SE CI Length x 10^3 | MSE x 10^3 | SE MSE x 10^3 |
|---|---|---|---|---|---|---|---|---|---|---|---|---|---|---|---|---|---|---|
| TS(0.5) | 1 | IPW | 0.531 | 0.531 | 0.041 | 0.059 | 1.018 | 0.000 | 0.944 | 3.254 | 0.941 | 3.330 | 0.953 | 2.986 | 0.149 | 0.640 | 1.707 | 0.057 |
| | 1 | WIPW | 0.531 | 0.529 | 0.036 | 0.000 | 1.004 | -0.002 | 0.948 | 3.141 | 0.952 | 3.035 | 0.951 | 3.041 | 0.138 | 0.307 | 1.278 | 0.030 |
| | 1 | AIPW | 0.531 | 0.531 | 0.045 | 0.033 | 1.037 | 0.000 | 0.941 | 3.341 | 0.943 | 3.287 | 0.945 | 3.226 | 0.152 | 1.282 | 2.038 | 0.198 |
| | 1 | WAIPW | 0.531 | 0.529 | 0.036 | -0.008 | 1.017 | -0.002 | 0.942 | 3.293 | 0.949 | 3.106 | 0.947 | 3.170 | 0.138 | 0.362 | 1.296 | 0.030 |
| WAIPW(0.5) | 1 | IPW | 0.531 | 0.530 | 0.036 | 0.048 | 1.007 | 0.000 | 0.953 | 3.005 | 0.947 | 3.169 | 0.954 | 2.963 | 0.140 | 0.205 | 1.274 | 0.025 |
| | 1 | WIPW | 0.531 | 0.529 | 0.034 | -0.005 | 0.995 | -0.002 | 0.956 | 2.907 | 0.952 | 3.029 | 0.951 | 3.041 | 0.136 | 0.166 | 1.170 | 0.023 |
| | 1 | AIPW | 0.531 | 0.530 | 0.036 | 0.045 | 1.016 | 0.000 | 0.950 | 3.094 | 0.946 | 3.191 | 0.950 | 3.071 | 0.139 | 0.210 | 1.261 | 0.025 |
| | 1 | WAIPW | 0.531 | 0.529 | 0.034 | -0.005 | 1.002 | -0.002 | 0.955 | 2.938 | 0.953 | 2.993 | 0.948 | 3.146 | 0.135 | 0.168 | 1.169 | 0.023 |
| TS(0.5) | 2 | IPW | 0.513 | 0.513 | 0.046 | 0.105 | 1.032 | 0.000 | 0.939 | 3.384 | 0.929 | 3.629 | 0.959 | 2.814 | 0.161 | 0.784 | 2.102 | 0.076 |
| | 2 | WIPW | 0.513 | 0.509 | 0.038 | -0.004 | 1.021 | -0.004 | 0.942 | 3.298 | 0.943 | 3.276 | 0.949 | 3.118 | 0.145 | 0.369 | 1.486 | 0.035 |
| | 2 | AIPW | 0.513 | 0.513 | 0.051 | 0.079 | 1.051 | 0.000 | 0.939 | 3.384 | 0.932 | 3.566 | 0.951 | 3.047 | 0.166 | 1.550 | 2.557 | 0.215 |

| Randomization Method | Regime | Estimator | True Value | Mean Estimate | SD Estimates | Mean Normalized | SD Normalized | Mean Bias | CI Coverage | SE CI Coverage x 10^3 | LB Coverage | SE LB Coverage x 10^3 | UB Coverage | SE UB Coverage x 10^3 | Mean CI Length | SE CI Length x 10^3 | MSE x 10^3 | SE MSE x 10^3 |
|---|---|---|---|---|---|---|---|---|---|---|---|---|---|---|---|---|---|---|
| | 2 | WAIPW | 0.513 | 0.509 | 0.039 | -0.010 | 1.036 | -0.004 | 0.938 | 3.415 | 0.942 | 3.309 | 0.945 | 3.232 | 0.146 | 0.420 | 1.516 | 0.036 |
| WAIPW(0.5) | 2 | IPW | 0.513 | 0.512 | 0.037 | 0.029 | 0.996 | -0.001 | 0.949 | 3.100 | 0.947 | 3.169 | 0.953 | 2.987 | 0.146 | 0.193 | 1.340 | 0.027 |
| | 2 | WIPW | 0.513 | 0.511 | 0.035 | -0.018 | 0.980 | -0.002 | 0.957 | 2.875 | 0.954 | 2.951 | 0.950 | 3.071 | 0.140 | 0.150 | 1.201 | 0.024 |
| | 2 | AIPW | 0.513 | 0.512 | 0.036 | 0.027 | 1.004 | -0.001 | 0.948 | 3.146 | 0.946 | 3.197 | 0.951 | 3.047 | 0.145 | 0.202 | 1.332 | 0.027 |
| | 2 | WAIPW | 0.513 | 0.511 | 0.035 | -0.016 | 0.988 | -0.002 | 0.953 | 2.987 | 0.953 | 2.993 | 0.951 | 3.065 | 0.140 | 0.155 | 1.210 | 0.024 |
| TS(0.5) | 3 | IPW | 0.544 | 0.545 | 0.039 | 0.081 | 1.015 | 0.000 | 0.945 | 3.238 | 0.941 | 3.341 | 0.955 | 2.930 | 0.140 | 0.632 | 1.507 | 0.054 |
| | 3 | WIPW | 0.544 | 0.544 | 0.036 | 0.063 | 1.011 | 0.000 | 0.945 | 3.215 | 0.942 | 3.309 | 0.952 | 3.035 | 0.136 | 0.463 | 1.288 | 0.034 |
| | 3 | AIPW | 0.544 | 0.545 | 0.042 | 0.069 | 1.026 | 0.001 | 0.943 | 3.276 | 0.942 | 3.314 | 0.950 | 3.077 | 0.143 | 1.135 | 1.773 | 0.127 |
| | 3 | WAIPW | 0.544 | 0.544 | 0.038 | 0.060 | 1.021 | 0.000 | 0.940 | 3.362 | 0.944 | 3.243 | 0.953 | 3.005 | 0.139 | 0.708 | 1.425 | 0.051 |
| WAIPW(0.5) | 3 | IPW | 0.544 | 0.544 | 0.035 | 0.048 | 1.003 | 0.000 | 0.949 | 3.112 | 0.942 | 3.301 | 0.955 | 2.920 | 0.136 | 0.212 | 1.207 | 0.027 |
| | 3 | WIPW | 0.544 | 0.542 | 0.033 | -0.009 | 0.994 | -0.002 | 0.951 | 3.047 | 0.952 | 3.035 | 0.951 | 3.047 | 0.132 | 0.176 | 1.124 | 0.024 |
| | 3 | AIPW | 0.544 | 0.544 | 0.035 | 0.049 | 1.006 | 0.000 | 0.948 | 3.152 | 0.942 | 3.301 | 0.957 | 2.882 | 0.136 | 0.216 | 1.196 | 0.026 |
| | 3 | WAIPW | 0.544 | 0.542 | 0.034 | -0.003 | 0.999 | -0.002 | 0.950 | 3.083 | 0.948 | 3.135 | 0.951 | 3.065 | 0.133 | 0.179 | 1.129 | 0.024 |
| TS(0.5) | 4 | IPW | 0.562 | 0.562 | 0.034 | 0.060 | 0.994 | 0.000 | 0.953 | 2.986 | 0.945 | 3.226 | 0.959 | 2.795 | 0.126 | 0.488 | 1.145 | 0.032 |
| | 4 | WIPW | 0.562 | 0.563 | 0.032 | 0.073 | 1.002 | 0.001 | 0.950 | 3.077 | 0.945 | 3.238 | 0.962 | 2.713 | 0.122 | 0.224 | 1.005 | 0.022 |
| | 4 | AIPW | 0.562 | 0.562 | 0.035 | 0.059 | 0.994 | 0.000 | 0.956 | 2.899 | 0.947 | 3.181 | 0.960 | 2.788 | 0.127 | 0.711 | 1.206 | 0.050 |
| | 4 | WAIPW | 0.562 | 0.563 | 0.032 | 0.076 | 1.009 | 0.001 | 0.949 | 3.100 | 0.942 | 3.309 | 0.960 | 2.775 | 0.122 | 0.250 | 1.017 | 0.023 |
| WAIPW(0.5) | 4 | IPW | 0.562 | 0.562 | 0.034 | 0.067 | 1.004 | 0.000 | 0.950 | 3.088 | 0.941 | 3.327 | 0.954 | 2.951 | 0.130 | 0.219 | 1.127 | 0.024 |
| | 4 | WIPW | 0.562 | 0.560 | 0.033 | 0.006 | 1.003 | -0.002 | 0.950 | 3.083 | 0.945 | 3.213 | 0.950 | 3.083 | 0.127 | 0.189 | 1.069 | 0.023 |
| | 4 | AIPW | 0.562 | 0.562 | 0.034 | 0.069 | 1.012 | 0.000 | 0.945 | 3.230 | 0.938 | 3.416 | 0.954 | 2.975 | 0.130 | 0.220 | 1.124 | 0.024 |
| | 4 | WAIPW | 0.562 | 0.560 | 0.033 | 0.009 | 1.010 | -0.002 | 0.950 | 3.094 | 0.943 | 3.274 | 0.948 | 3.135 | 0.127 | 0.190 | 1.075 | 0.023 |
| TS(0.5) | 5 | IPW | 0.519 | 0.518 | 0.070 | 0.097 | 1.120 | -0.001 | 0.928 | 3.658 | 0.927 | 3.672 | 0.946 | 3.210 | 0.217 | 1.336 | 4.848 | 0.174 |
| | 5 | WIPW | 0.519 | 0.515 | 0.045 | -0.014 | 1.018 | -0.005 | 0.941 | 3.330 | 0.948 | 3.141 | 0.947 | 3.159 | 0.169 | 0.476 | 2.032 | 0.051 |
| | 5 | AIPW | 0.519 | 0.518 | 0.089 | 0.084 | 1.054 | -0.002 | 0.940 | 3.357 | 0.939 | 3.389 | 0.950 | 3.095 | 0.240 | 3.558 | 7.983 | 1.104 |
| | 5 | WAIPW | 0.519 | 0.515 | 0.045 | -0.014 | 1.016 | -0.005 | 0.942 | 3.309 | 0.948 | 3.141 | 0.945 | 3.215 | 0.170 | 0.559 | 2.047 | 0.053 |
| WAIPW(0.5) | 5 | IPW | 0.519 | 0.520 | 0.039 | 0.063 | 1.037 | 0.000 | 0.940 | 3.359 | 0.936 | 3.467 | 0.948 | 3.129 | 0.150 | 0.235 | 1.525 | 0.031 |
| | 5 | WIPW | 0.519 | 0.518 | 0.036 | 0.002 | 1.017 | -0.002 | 0.943 | 3.284 | 0.946 | 3.191 | 0.950 | 3.088 | 0.142 | 0.177 | 1.324 | 0.027 |
| | 5 | AIPW | 0.519 | 0.519 | 0.039 | 0.056 | 1.043 | 0.000 | 0.938 | 3.406 | 0.938 | 3.416 | 0.945 | 3.213 | 0.149 | 0.244 | 1.509 | 0.031 |
| | 5 | WAIPW | 0.519 | 0.518 | 0.036 | -0.002 | 1.023 | -0.002 | 0.942 | 3.306 | 0.947 | 3.169 | 0.946 | 3.197 | 0.141 | 0.179 | 1.318 | 0.027 |
| TS(0.5) | 6 | IPW | 0.513 | 0.511 | 0.070 | 0.091 | 1.134 | -0.002 | 0.924 | 3.746 | 0.930 | 3.605 | 0.943 | 3.287 | 0.219 | 1.344 | 4.917 | 0.178 |
| | 6 | WIPW | 0.513 | 0.507 | 0.045 | -0.032 | 1.017 | -0.006 | 0.945 | 3.238 | 0.951 | 3.059 | 0.945 | 3.232 | 0.170 | 0.480 | 2.075 | 0.053 |
| | 6 | AIPW | 0.513 | 0.511 | 0.090 | 0.079 | 1.065 | -0.002 | 0.939 | 3.389 | 0.941 | 3.336 | 0.945 | 3.226 | 0.242 | 3.556 | 8.026 | 1.136 |
| | 6 | WAIPW | 0.513 | 0.507 | 0.046 | -0.035 | 1.023 | -0.006 | 0.940 | 3.352 | 0.951 | 3.047 | 0.940 | 3.357 | 0.173 | 0.578 | 2.156 | 0.057 |
| WAIPW(0.5) | 6 | IPW | 0.513 | 0.513 | 0.039 | 0.049 | 1.032 | 0.000 | 0.941 | 3.338 | 0.940 | 3.354 | 0.949 | 3.123 | 0.150 | 0.233 | 1.524 | 0.031 |
| | 6 | WIPW | 0.513 | 0.511 | 0.036 | -0.010 | 1.011 | -0.002 | 0.945 | 3.230 | 0.951 | 3.047 | 0.946 | 3.202 | 0.142 | 0.174 | 1.317 | 0.027 |
| | 6 | AIPW | 0.513 | 0.513 | 0.039 | 0.039 | 1.039 | -0.001 | 0.937 | 3.447 | 0.943 | 3.290 | 0.945 | 3.235 | 0.149 | 0.243 | 1.510 | 0.031 |
| | 6 | WAIPW | 0.513 | 0.511 | 0.036 | -0.016 | 1.017 | -0.002 | 0.944 | 3.263 | 0.952 | 3.023 | 0.944 | 3.263 | 0.142 | 0.177 | 1.325 | 0.027 |
| TS(0.5) | 7 | IPW | 0.603 | 0.603 | 0.035 | 0.063 | 1.018 | -0.001 | 0.946 | 3.210 | 0.939 | 3.378 | 0.956 | 2.899 | 0.124 | 0.616 | 1.240 | 0.046 |
| | 7 | WIPW | 0.603 | 0.601 | 0.030 | 0.005 | 1.021 | -0.002 | 0.947 | 3.170 | 0.946 | 3.187 | 0.950 | 3.089 | 0.114 | 0.259 | 0.929 | 0.024 |
| | 7 | AIPW | 0.603 | 0.602 | 0.036 | 0.047 | 1.020 | -0.001 | 0.946 | 3.198 | 0.941 | 3.325 | 0.952 | 3.011 | 0.127 | 0.842 | 1.332 | 0.058 |
| | 7 | WAIPW | 0.603 | 0.601 | 0.032 | 0.003 | 1.031 | -0.002 | 0.940 | 3.352 | 0.947 | 3.181 | 0.944 | 3.249 | 0.116 | 0.341 | 0.997 | 0.026 |
| WAIPW(0.5) | 7 | IPW | 0.603 | 0.603 | 0.029 | 0.047 | 0.984 | 0.000 | 0.953 | 2.987 | 0.947 | 3.180 | 0.954 | 2.975 | 0.112 | 0.154 | 0.815 | 0.017 |
| | 7 | WIPW | 0.603 | 0.601 | 0.028 | -0.004 | 0.992 | -0.002 | 0.953 | 2.987 | 0.951 | 3.059 | 0.946 | 3.186 | 0.109 | 0.163 | 0.791 | 0.017 |
| | 7 | AIPW | 0.603 | 0.603 | 0.029 | 0.040 | 0.993 | -0.001 | 0.951 | 3.065 | 0.946 | 3.191 | 0.951 | 3.047 | 0.111 | 0.154 | 0.816 | 0.017 |

| Randomization Method | Regime | Estimator | True Value | Mean Estimate | SD Estimates | Mean Normalized | SD Normalized | Mean Bias | CI Coverage | SE CI Coverage x 10^3 | LB Coverage | SE LB Coverage x 10^3 | UB Coverage | SE UB Coverage x 10^3 | Mean CI Length | SE CI Length x 10^3 | MSE x 10^3 | SE MSE x 10^3 |
|---|---|---|---|---|---|---|---|---|---|---|---|---|---|---|---|---|---|---|
| | 7 | WAIPW | 0.603 | 0.601 | 0.028 | -0.008 | 0.999 | -0.002 | 0.949 | 3.106 | 0.949 | 3.106 | 0.945 | 3.224 | 0.110 | 0.166 | 0.807 | 0.018 |
| TS(0.5) | 8 | IPW | 0.609 | 0.609 | 0.035 | 0.059 | 1.015 | 0.000 | 0.947 | 3.170 | 0.940 | 3.368 | 0.954 | 2.974 | 0.121 | 0.600 | 1.191 | 0.044 |
| | 8 | WIPW | 0.609 | 0.609 | 0.029 | 0.020 | 1.013 | -0.001 | 0.948 | 3.141 | 0.944 | 3.249 | 0.951 | 3.041 | 0.111 | 0.220 | 0.860 | 0.021 |
| | 8 | AIPW | 0.609 | 0.609 | 0.036 | 0.058 | 1.011 | 0.000 | 0.948 | 3.147 | 0.939 | 3.384 | 0.953 | 2.986 | 0.124 | 0.824 | 1.263 | 0.054 |
| | 8 | WAIPW | 0.609 | 0.609 | 0.030 | 0.021 | 1.020 | -0.001 | 0.946 | 3.210 | 0.944 | 3.265 | 0.949 | 3.100 | 0.114 | 0.366 | 0.930 | 0.024 |
| WAIPW(0.5) | 8 | IPW | 0.609 | 0.609 | 0.028 | 0.062 | 0.986 | 0.000 | 0.954 | 2.957 | 0.946 | 3.197 | 0.960 | 2.772 | 0.111 | 0.153 | 0.797 | 0.017 |
| | 8 | WIPW | 0.609 | 0.608 | 0.028 | 0.012 | 0.994 | -0.002 | 0.952 | 3.011 | 0.948 | 3.146 | 0.950 | 3.077 | 0.108 | 0.164 | 0.768 | 0.017 |
| | 8 | AIPW | 0.609 | 0.609 | 0.028 | 0.060 | 0.989 | 0.000 | 0.953 | 2.981 | 0.946 | 3.191 | 0.960 | 2.765 | 0.111 | 0.154 | 0.791 | 0.017 |
| | 8 | WAIPW | 0.609 | 0.608 | 0.028 | 0.013 | 1.001 | -0.002 | 0.951 | 3.047 | 0.948 | 3.152 | 0.953 | 2.981 | 0.107 | 0.164 | 0.775 | 0.017 |

Identifying the Optimal Regime Results for the Binary Outcome Scenario

| Randomization Method | Estimator | Proportion Estimating d1 Optimal | SE Proprtion d1 x 10^3 | Proportion Estimating d2 Optimal | SE Proprtion d2 x 10^3 | Proportion Estimating d3 Optimal | SE Proprtion d3 x 10^3 | Proportion Estimating d4 Optimal | SE Proprtion d4 x 10^3 | Proportion Estimating d5 Optimal | SE Proprtion d5 x 10^3 | Proportion Estimating d6 Optimal | SE Proprtion d6 x 10^3 | Proportion Estimating d7 Optimal | SE Proprtion d7 x 10^3 | Proportion Estimating d8 Optimal | SE Proprtion d8 x 10^3 |
|---|---|---|---|---|---|---|---|---|---|---|---|---|---|---|---|---|---|
| TS(0.5) | IPW | 0.018 | 1.881 | 0.007 | 1.152 | 0.039 | 2.748 | 0.100 | 4.243 | 0.035 | 2.600 | 0.010 | 1.388 | 0.241 | 6.050 | 0.550 | 7.036 |
| | WIPW | 0.010 | 1.402 | 0.003 | 0.725 | 0.037 | 2.672 | 0.105 | 4.345 | 0.011 | 1.457 | 0.002 | 0.569 | 0.242 | 6.057 | 0.591 | 6.955 |
| | AIPW | 0.019 | 1.922 | 0.007 | 1.186 | 0.039 | 2.741 | 0.099 | 4.220 | 0.034 | 2.578 | 0.011 | 1.484 | 0.244 | 6.076 | 0.546 | 7.041 |
| | WAIPW | 0.011 | 1.471 | 0.003 | 0.752 | 0.040 | 2.775 | 0.106 | 4.348 | 0.012 | 1.511 | 0.002 | 0.603 | 0.258 | 6.189 | 0.569 | 7.004 |
| WAIPW(0.5) | IPW | 0.010 | 1.407 | 0.003 | 0.720 | 0.028 | 2.333 | 0.099 | 4.228 | 0.006 | 1.092 | 0.003 | 0.823 | 0.237 | 6.011 | 0.614 | 6.885 |
| | WIPW | 0.009 | 1.350 | 0.002 | 0.600 | 0.029 | 2.373 | 0.099 | 4.224 | 0.004 | 0.915 | 0.002 | 0.632 | 0.231 | 5.963 | 0.624 | 6.852 |
| | AIPW | 0.011 | 1.488 | 0.002 | 0.692 | 0.027 | 2.292 | 0.098 | 4.209 | 0.007 | 1.145 | 0.002 | 0.692 | 0.236 | 6.006 | 0.616 | 6.878 |
| | WAIPW | 0.011 | 1.448 | 0.002 | 0.632 | 0.027 | 2.292 | 0.098 | 4.205 | 0.007 | 1.179 | 0.002 | 0.632 | 0.239 | 6.028 | 0.615 | 6.883 |

Regime 1 Normalized Estimates for WAIPW(0.5) Randomization

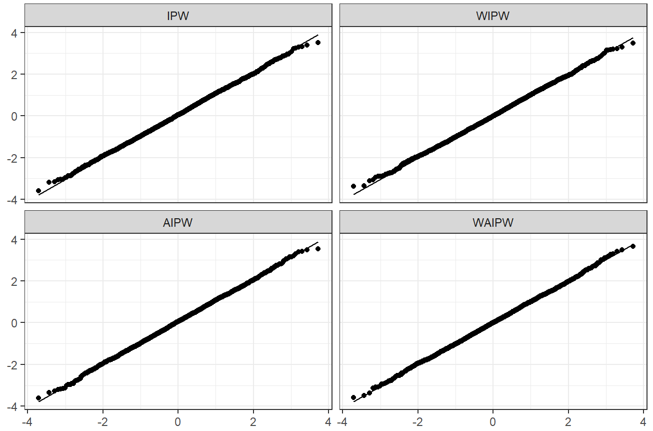


Regime 1 Normalized Estimates for TS(0.5) Randomization

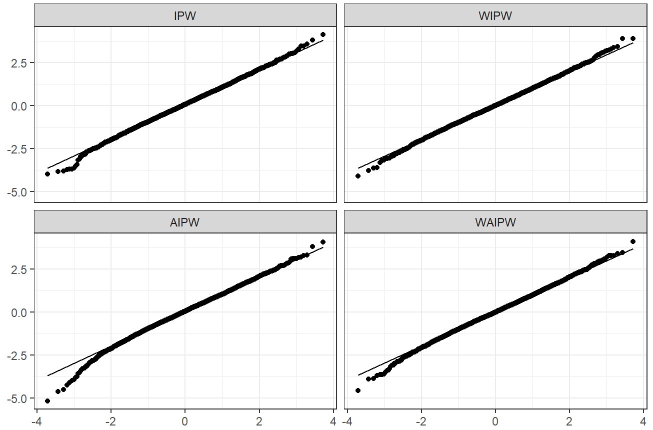


Regime 8 Normalized Estimates for WAIPW(0.5) Randomization

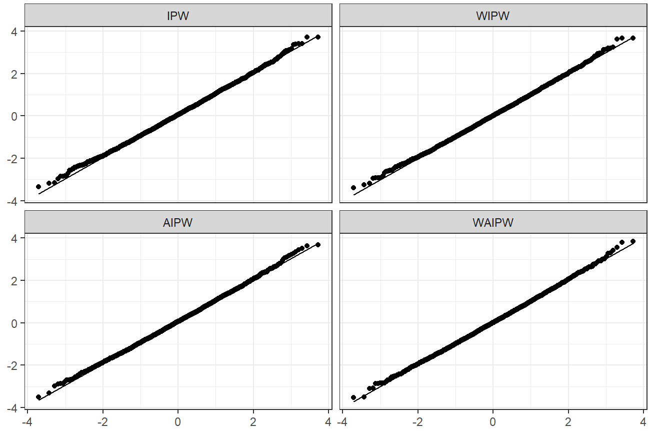

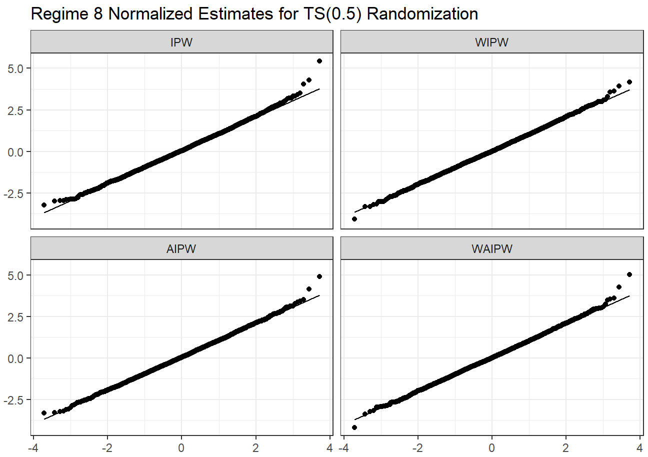


# C.5 Cancer Pain Management SMART, Continuous Outcome, Different Mean Outcomes

## C.5.1 Simulation Details

We present results of a simulation study involving 5000 Monte Carlo trials in a four-regime scenario similar to the pain management SMART, but where “responders” are not re-randomized.

Each trial enrolls $N = 1000$ subjects, with enrollment times uniform over (integer) weeks 1-24. Upon enrollment, we draw baseline pain score $X_1 \sim N(5, 1)$ and assign stage 1 treatment $A_1 \in \square_1 = \{0, 1\}$. Six weeks after $A_1$ is assigned, second-stage pain score is generated as $X_{2,1} = \gamma_{1,0} + \gamma_{1,1}X_1 + \gamma_{1,2}A_1 + \varepsilon_1$, where $\varepsilon_1 \sim N(0, 1)$, and response status after the first stage is $X_{2,2} = I(X_{2,1} < 0.7X_1)$, which dictates the feasible subset of $\square_2 = \{0, 1\}$ from which stage 2 treatment $A_2$ is assigned. Those with $X_{2,2} = 1$ are given $A_2 = A_1$, hence they are not re-randomized. Those with $X_{2,2} = 0$ are randomized to $\{0, 1\}$. Six weeks later, the outcome is generated as $Y = \gamma_{2,0} + \gamma_{2,1}X_1 + \gamma_{2,2}A_1 + \gamma_{2,3}X_{2,1} + \gamma_{2,4}A_2 + \varepsilon_2$, where $\varepsilon_2 \sim N(0, 1)$. With $\gamma_1 = (\gamma_{1,0}, \gamma_{1,1}, \gamma_{1,2})^T = (0.00, 0.90, -0.50)^T$ and $\gamma_2 = (\gamma_{2,0}, \dots, \gamma_{2,4}) = (0.00, 0.10, -0.075, 0.60, -0.075)^T$.

The four embedded regimes are as follows. Regime 1 assigns $A_1 = 0$ and $A_2 = 0$ if $X_{2,2} = 0$. Regime 2 assigns $A_1 = 0$ and $A_2 = 0$ if $X_{2,2} = 1$. Regime 3 assigns $A_1 = 1$ and $A_2 = 0$ if $X_{2,2} = 0$. Regime 4 assigns $A_1 = 1$ and $A_2 = 1$ if $X_{2,2} = 0$. The values for these regimes are $\{\square(\mathbf{d}^1), \dots, \square(\mathbf{d}^4)\} = (\theta_1, \dots, \theta_4) = (0.000 - 0.040 - 0.429, -0.452)$. Regime 4 is optimal.

The burn-in period ends at the time $t^*$ when each of the $m = 4$ regimes has at least 25 subjects who have completed the trial with experience consistent with following the regime.

For demonstration, we randomize via WAIPW(0.5) and TS(0.5).

For the WAIPW estimator in randomization and W/AIPW estimators in post-trial inference we use: $Q_2(\overline{\mathbf{X}_2}, \overline{\mathbf{a}_2}; \beta_2) = \beta_{2,0} + \beta_{2,1}x_1 + \beta_{2,2}a_1\beta_{2,3}x_{2,1} + \beta_{2,4}a_1$ and $Q_1^j(x_1, a_1; \beta_1^j) = \beta_{1,0}^j + \beta_{1,1}^j x_1 + \beta_{1,2}^j a_1 + \beta_{1,3}^j x_1 a_1$. Because responders are not re-randomized, we “carry back” their true outcome rather than creating a pseudo-outcome.

For TS(0.5), $Q_2(\overline{\mathbf{X}_2}, \overline{\mathbf{a}_2}; \beta_2) = \beta_{2,0} + \beta_{2,1}x_1 + \beta_{2,2}x_{2,2} + \beta_{2,3}a_2$ and $Q_1(x_1, a_1; \beta_1) = \beta_{1,0} + \beta_{1,1}x_1 + \beta_{1,2}a_1$. We fit separate models at stage two for those subjects that recieve $A_2 = 0$ vs $A_2 = 1$. Like with the W/AIPW estimators, we “carry back” responders true outcome rather than creating a pseudo-outcome. To compute the For sequential RAR, we set $B_1 = b_2 \times b_1 = 32 \times 32 = 1024$ and $B_2 = 1000$. A clipping constant of 0.05 was imposed on all sequential methods.

Tables and figures in the following sections are as in Section C.1, except the plots use regimes 1 and 4.

## C.5.2 Estimation Results

Estimation Results for the Four Regimes Scenario

| Randomization Method | Regime | Estimator | True Value | Mean Estimate | SD Estimates | Mean Normalized | SD Normalized | Mean Bias | CI Coverage | SE CI Coverage x 10^3 | LB Coverage | SE LB Coverage x 10^3 | UB Coverage | SE UB Coverage x 10^3 | Mean CI Length | SE CI Length x 10^3 | MSE x 10^3 | SE MSE x 10^3 |
|---|---|---|---|---|---|---|---|---|---|---|---|---|---|---|---|---|---|---|
| TS(0.5) | 1 | IPW | 0.000 | 0.005 | 0.289 | -0.005 | 1.240 | 0.004 | 0.889 | 4.436 | 0.915 | 3.953 | 0.908 | 4.084 | 0.959 | 5.321 | 83.497 | 1.790 |
| | 1 | WIPW | 0.000 | 0.005 | 0.134 | 0.003 | 1.046 | 0.004 | 0.939 | 3.385 | 0.944 | 3.252 | 0.941 | 3.327 | 0.506 | 1.517 | 17.947 | 0.368 |
| | 1 | AIPW | 0.000 | 0.005 | 0.256 | -0.003 | 1.059 | 0.005 | 0.947 | 3.157 | 0.942 | 3.311 | 0.940 | 3.364 | 0.907 | 5.527 | 65.695 | 1.569 |
| | 1 | WAIPW | 0.000 | 0.004 | 0.239 | -0.006 | 1.051 | 0.004 | 0.951 | 3.059 | 0.945 | 3.235 | 0.945 | 3.230 | 0.852 | 5.115 | 57.350 | 1.373 |
| WAIPW(0.5) | 1 | IPW | 0.000 | -0.001 | 0.121 | -0.037 | 1.031 | -0.001 | 0.940 | 3.359 | 0.952 | 3.017 | 0.936 | 3.467 | 0.464 | 0.714 | 14.561 | 0.294 |
| | 1 | WIPW | 0.000 | 0.000 | 0.106 | -0.021 | 1.022 | 0.000 | 0.941 | 3.333 | 0.949 | 3.106 | 0.938 | 3.421 | 0.409 | 0.525 | 11.221 | 0.226 |

| Randomization Method | Regime | Estimator | True Value | Mean Estimate | SD Estimates | Mean Normalized | SD Normalized | Mean Bias | CI Coverage | SE CI Coverage x 10^3 | LB Coverage | SE LB Coverage x 10^3 | UB Coverage | SE UB Coverage x 10^3 | Mean CI Length | SE CI Length x 10^3 | MSE x 10^3 | SE MSE x 10^3 |
|---|---|---|---|---|---|---|---|---|---|---|---|---|---|---|---|---|---|---|
| | 1 | AIPW | 0.000 | 0.002 | 0.103 | -0.006 | 1.024 | 0.002 | 0.941 | 3.327 | 0.949 | 3.106 | 0.943 | 3.290 | 0.397 | 0.627 | 10.557 | 0.209 |
| | 1 | WAIPW | 0.000 | 0.003 | 0.088 | 0.011 | 1.021 | 0.003 | 0.944 | 3.246 | 0.943 | 3.268 | 0.947 | 3.163 | 0.343 | 0.362 | 7.837 | 0.155 |
| TS(0.5) | 2 | IPW | -0.040 | -0.179 | 0.443 | -0.750 | 2.340 | -0.139 | 0.734 | 6.253 | 0.935 | 3.482 | 0.719 | 6.359 | 1.006 | 7.093 | 215.305 | 7.259 |
| | 2 | WIPW | -0.040 | -0.045 | 0.204 | -0.164 | 1.151 | -0.005 | 0.912 | 4.011 | 0.954 | 2.969 | 0.897 | 4.292 | 0.623 | 3.672 | 41.480 | 1.761 |
| | 2 | AIPW | -0.040 | -0.031 | 1.373 | 0.113 | 1.137 | 0.009 | 0.923 | 3.762 | 0.907 | 4.112 | 0.959 | 2.791 | 1.601 | 72.465 | 1884.320 | 345.671 |
| | 2 | WAIPW | -0.040 | -0.035 | 0.275 | 0.024 | 1.062 | 0.005 | 0.945 | 3.213 | 0.946 | 3.191 | 0.941 | 3.333 | 0.647 | 12.146 | 75.778 | 12.944 |
| WAIPW(0.5) | 2 | IPW | -0.040 | -0.035 | 0.115 | 0.013 | 1.015 | 0.005 | 0.946 | 3.197 | 0.953 | 3.005 | 0.942 | 3.295 | 0.454 | 0.743 | 13.286 | 0.267 |
| | 2 | WIPW | -0.040 | -0.033 | 0.101 | 0.044 | 1.003 | 0.007 | 0.948 | 3.135 | 0.948 | 3.146 | 0.953 | 2.981 | 0.401 | 0.548 | 10.287 | 0.208 |
| | 2 | AIPW | -0.040 | -0.035 | 0.099 | 0.025 | 1.014 | 0.005 | 0.948 | 3.146 | 0.949 | 3.123 | 0.944 | 3.241 | 0.390 | 0.632 | 9.857 | 0.196 |
| | 2 | WAIPW | -0.040 | -0.032 | 0.085 | 0.066 | 0.998 | 0.008 | 0.950 | 3.083 | 0.945 | 3.213 | 0.953 | 3.005 | 0.339 | 0.368 | 7.290 | 0.146 |
| TS(0.5) | 3 | IPW | -0.429 | -0.428 | 0.055 | 0.005 | 1.016 | 0.001 | 0.946 | 3.197 | 0.949 | 3.106 | 0.946 | 3.208 | 0.211 | 0.192 | 2.986 | 0.060 |
| | 3 | WIPW | -0.429 | -0.428 | 0.051 | 0.009 | 1.014 | 0.001 | 0.947 | 3.157 | 0.947 | 3.180 | 0.949 | 3.106 | 0.198 | 0.127 | 2.640 | 0.054 |
| | 3 | AIPW | -0.429 | -0.428 | 0.051 | 0.014 | 1.010 | 0.001 | 0.944 | 3.246 | 0.947 | 3.174 | 0.948 | 3.140 | 0.197 | 0.146 | 2.566 | 0.052 |
| | 3 | WAIPW | -0.429 | -0.428 | 0.049 | 0.016 | 1.012 | 0.001 | 0.943 | 3.274 | 0.946 | 3.197 | 0.948 | 3.140 | 0.191 | 0.111 | 2.433 | 0.049 |
| WAIPW(0.5) | 3 | IPW | -0.429 | -0.429 | 0.064 | -0.041 | 0.993 | 0.000 | 0.954 | 2.969 | 0.956 | 2.913 | 0.944 | 3.252 | 0.249 | 0.429 | 4.074 | 0.085 |
| | 3 | WIPW | -0.429 | -0.427 | 0.063 | -0.010 | 0.997 | 0.002 | 0.951 | 3.047 | 0.952 | 3.029 | 0.949 | 3.106 | 0.244 | 0.410 | 3.964 | 0.084 |
| | 3 | AIPW | -0.429 | -0.428 | 0.056 | -0.005 | 0.990 | 0.001 | 0.950 | 3.071 | 0.955 | 2.926 | 0.949 | 3.100 | 0.219 | 0.262 | 3.087 | 0.065 |
| | 3 | WAIPW | -0.429 | -0.426 | 0.055 | 0.021 | 0.992 | 0.003 | 0.948 | 3.129 | 0.952 | 3.017 | 0.954 | 2.957 | 0.217 | 0.273 | 3.057 | 0.065 |
| TS(0.5) | 4 | IPW | -0.452 | -0.462 | 0.163 | -0.464 | 1.394 | -0.009 | 0.852 | 5.017 | 0.978 | 2.093 | 0.811 | 5.535 | 0.516 | 3.928 | 26.671 | 0.672 |
| | 4 | WIPW | -0.452 | -0.451 | 0.118 | -0.120 | 1.106 | 0.001 | 0.922 | 3.788 | 0.964 | 2.642 | 0.907 | 4.104 | 0.422 | 2.104 | 13.842 | 0.335 |
| | 4 | AIPW | -0.452 | -0.451 | 0.125 | 0.003 | 1.023 | 0.001 | 0.954 | 2.957 | 0.952 | 3.029 | 0.953 | 2.999 | 0.416 | 3.580 | 15.543 | 0.491 |
| | 4 | WAIPW | -0.452 | -0.450 | 0.100 | 0.024 | 1.014 | 0.003 | 0.947 | 3.157 | 0.945 | 3.213 | 0.949 | 3.112 | 0.357 | 2.233 | 10.042 | 0.307 |
| WAIPW(0.5) | 4 | IPW | -0.452 | -0.450 | 0.061 | 0.004 | 1.012 | 0.002 | 0.950 | 3.088 | 0.949 | 3.106 | 0.947 | 3.163 | 0.234 | 0.301 | 3.683 | 0.075 |
| | 4 | WIPW | -0.452 | -0.449 | 0.059 | 0.029 | 1.014 | 0.003 | 0.949 | 3.123 | 0.943 | 3.274 | 0.951 | 3.047 | 0.228 | 0.299 | 3.548 | 0.073 |
| | 4 | AIPW | -0.452 | -0.451 | 0.054 | 0.013 | 1.004 | 0.002 | 0.949 | 3.123 | 0.947 | 3.157 | 0.950 | 3.094 | 0.210 | 0.199 | 2.925 | 0.058 |
| | 4 | WAIPW | -0.452 | -0.449 | 0.053 | 0.035 | 1.003 | 0.003 | 0.951 | 3.059 | 0.946 | 3.197 | 0.952 | 3.017 | 0.207 | 0.200 | 2.834 | 0.056 |

Identifying the Optimal Regime Results for the Four Regime Scenario

| Randomization Method | Estimator | Proportion Estimating d1 Optimal | SE Proprtion d1 x 10^3 | Proportion Estimating d2 Optimal | SE Proprtion d2 x 10^3 | Proportion Estimating d3 Optimal | SE Proprtion d3 x 10^3 | Proportion Estimating d4 Optimal | SE Proprtion d4 x 10^3 |
|---|---|---|---|---|---|---|---|---|---|
| TS(0.5) | IPW | 0.016 | 1.775 | 0.195 | 5.604 | 0.270 | 6.282 | 0.519 | 7.067 |
| | WIPW | 0.000 | 0.283 | 0.014 | 1.638 | 0.393 | 6.908 | 0.593 | 6.948 |
| | AIPW | 0.021 | 2.028 | 0.066 | 3.521 | 0.353 | 6.758 | 0.560 | 7.021 |
| | WAIPW | 0.023 | 2.120 | 0.019 | 1.931 | 0.365 | 6.811 | 0.593 | 6.949 |
| WAIPW(0.5) | IPW | 0.000 | 0.000 | 0.000 | 0.200 | 0.374 | 6.845 | 0.625 | 6.846 |
| | WIPW | 0.000 | 0.000 | 0.000 | 0.000 | 0.371 | 6.832 | 0.629 | 6.832 |
| | AIPW | 0.000 | 0.000 | 0.000 | 0.000 | 0.295 | 6.451 | 0.705 | 6.451 |
| | WAIPW | 0.000 | 0.000 | 0.000 | 0.000 | 0.298 | 6.469 | 0.702 | 6.469 |

Regime 1 Normalized Estimates for WAIPW(0.5) Randomization

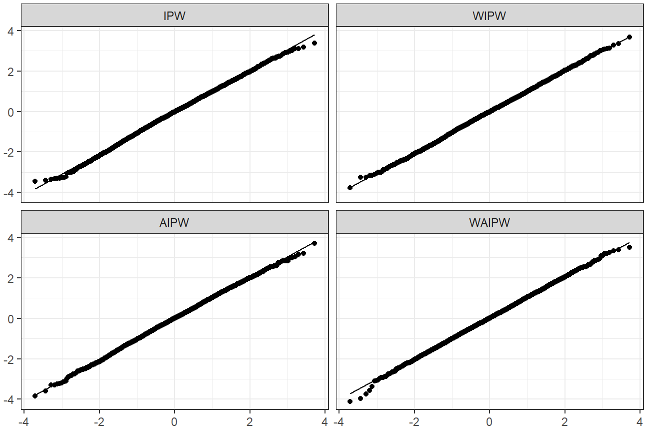


Regime 1 Normalized Estimates for TS(0.5) Randomization

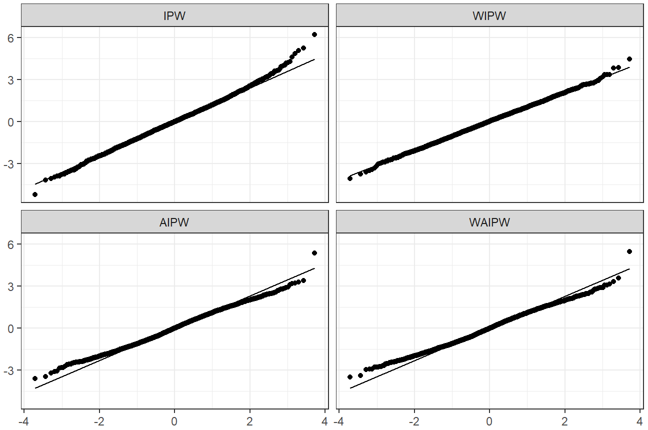


Regime 4 Normalized Estimates for WAIPW(0.5) Randomization

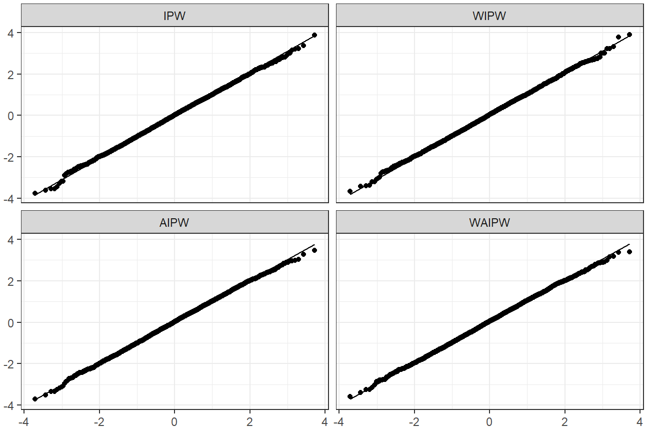

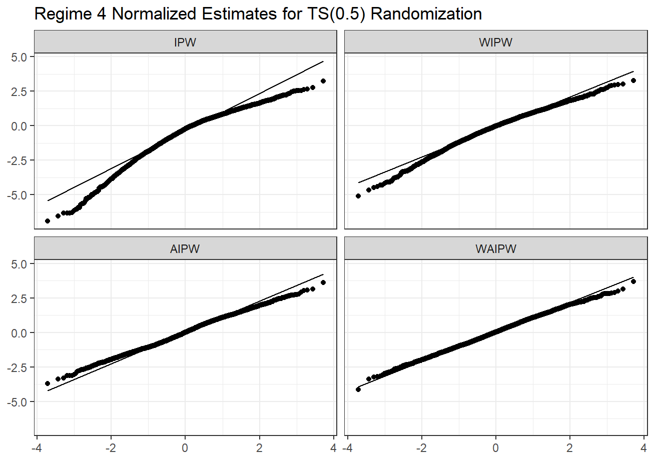


# C.6 Cancer Pain Management SMART, Continuous Outcome, Different Mean Outcomes, Small Sample Size (N=325)

## C.6.1 Simulation Details

Tables and figures in the following sections are as in Section C.1.

## C.6.2 In Trial Results

In Trial Results for the N=325 Continuous Outcome Scenario

| Randomization Method | Mean Y | SE Mean Y x 10^3 | Mean Proportion A1=1 | SE Mean Proportion A1=1 x 10^3 | Mean Proportion Cd8=1 | SE Mean Proportion Cd8=1 x 10^3 | Mean Proportion Cd7=1 or Cd8=1 | SE Mean Proportion Cd7=1 or Cd8=1 x 10^3 |
|---|---|---|---|---|---|---|---|---|
| AIPW(0.25) | -1.511 | 1.425 | 0.559 | 0.410 | 0.290 | 0.457 | 0.309 | 0.046 |
| AIPW(0.5) | -1.625 | 1.445 | 0.612 | 0.418 | 0.329 | 0.742 | 0.350 | 0.074 |
| AIPW(0.75) | -1.701 | 1.429 | 0.648 | 0.418 | 0.359 | 1.067 | 0.381 | 0.106 |
| AIPW(1) | -1.741 | 1.425 | 0.667 | 0.423 | 0.375 | 1.277 | 0.397 | 0.127 |
| AR-1 | -1.700 | 1.434 | 0.653 | 0.421 | 0.306 | 0.649 | 0.331 | 0.063 |
| AR-2 | -1.694 | 1.440 | 0.653 | 0.430 | 0.289 | 0.996 | 0.314 | 0.097 |
| IAIPW(0.25) | -1.511 | 1.412 | 0.560 | 0.409 | 0.290 | 0.446 | 0.310 | 0.045 |
| IAIPW(0.5) | -1.621 | 1.441 | 0.612 | 0.410 | 0.327 | 0.744 | 0.348 | 0.074 |
| IAIPW(0.75) | -1.701 | 1.429 | 0.648 | 0.431 | 0.358 | 1.044 | 0.380 | 0.104 |
| IAIPW(1) | -1.740 | 1.447 | 0.666 | 0.428 | 0.377 | 1.246 | 0.400 | 0.124 |
| IPW(0.25) | -1.510 | 1.392 | 0.560 | 0.394 | 0.288 | 0.452 | 0.307 | 0.046 |
| IPW(0.5) | -1.624 | 1.415 | 0.613 | 0.406 | 0.323 | 0.766 | 0.345 | 0.077 |
| IPW(0.75) | -1.702 | 1.457 | 0.648 | 0.426 | 0.348 | 1.076 | 0.371 | 0.108 |
| IPW(1) | -1.740 | 1.445 | 0.667 | 0.426 | 0.364 | 1.283 | 0.388 | 0.128 |
| SR | -1.380 | 1.359 | 0.500 | 0.389 | 0.250 | 0.337 | 0.267 | 0.034 |
| TS(0.25) | -1.654 | 1.936 | 0.624 | 0.786 | 0.330 | 0.716 | 0.352 | 0.075 |
| TS(0.50) | -1.691 | 1.809 | 0.639 | 0.692 | 0.353 | 0.991 | 0.375 | 0.101 |
| TS(0.75) | -1.711 | 1.707 | 0.648 | 0.647 | 0.370 | 1.243 | 0.393 | 0.125 |
| TS(1) | -1.723 | 1.708 | 0.653 | 0.641 | 0.378 | 1.432 | 0.400 | 0.143 |
| WAIPW(0.25) | -1.509 | 1.426 | 0.559 | 0.414 | 0.290 | 0.460 | 0.310 | 0.047 |
| WAIPW(0.5) | -1.625 | 1.425 | 0.613 | 0.417 | 0.329 | 0.757 | 0.351 | 0.076 |
| WAIPW(0.75) | -1.700 | 1.442 | 0.648 | 0.423 | 0.357 | 1.043 | 0.379 | 0.104 |

| Randomization Method | Mean Y | SE Mean Y x 10^3 | Mean Proportion A1=1 | SE Mean Proportion A1=1 x 10^3 | Mean Proportion Cd8=1 | SE Mean Proportion Cd8=1 x 10^3 | Mean Proportion Cd7=1 or Cd8=1 | SE Mean Proportion Cd7=1 or Cd8=1 x 10^3 |
|---|---|---|---|---|---|---|---|---|
| WAIPW(1) | -1.741 | 1.456 | 0.666 | 0.430 | 0.374 | 1.260 | 0.397 | 0.126 |
| WIPW(0.25) | -1.510 | 1.410 | 0.560 | 0.402 | 0.287 | 0.453 | 0.307 | 0.046 |
| WIPW(0.5) | -1.626 | 1.414 | 0.613 | 0.406 | 0.323 | 0.766 | 0.344 | 0.077 |
| WIPW(0.75) | -1.702 | 1.426 | 0.648 | 0.414 | 0.349 | 1.083 | 0.371 | 0.108 |
| WIPW(1) | -1.740 | 1.451 | 0.667 | 0.426 | 0.361 | 1.303 | 0.384 | 0.130 |

### C.6.3 Estimation Results

Estimation Results for the N=325 Continuous Outcome Scenario

| Randomization Method | Regime | Estimator | True Value | Mean Estimate | SD Estimates | Mean Normalized | SD Normalized | Mean Bias | CI Coverage | SE CI Coverage x 10^3 | LB Coverage | SE LB Coverage x 10^3 | UB Coverage | SE UB Coverage x 10^3 | Mean CI Length | SE CI Length x 10^3 | MSE x 10^3 | SE MSE x 10^3 |
|---|---|---|---|---|---|---|---|---|---|---|---|---|---|---|---|---|---|---|
| AIPW(0.25) | 1 | IPW | -0.126 | -0.128 | 0.170 | -0.016 | 1.032 | -0.002 | 0.945 | 3.219 | 0.948 | 3.152 | 0.940 | 3.354 | 0.650 | 0.987 | 28.740 | 0.573 |
| | 1 | WIPW | -0.126 | -0.130 | 0.256 | -0.021 | 1.096 | -0.004 | 0.925 | 3.734 | 0.939 | 3.385 | 0.927 | 3.675 | 0.948 | 2.408 | 65.733 | 1.303 |
| | 1 | AIPW | -0.126 | -0.129 | 0.137 | -0.026 | 1.033 | -0.003 | 0.942 | 3.295 | 0.950 | 3.094 | 0.940 | 3.354 | 0.526 | 0.684 | 18.742 | 0.386 |
| | 1 | WAIPW | -0.126 | -0.129 | 0.137 | -0.026 | 1.031 | -0.003 | 0.942 | 3.295 | 0.950 | 3.088 | 0.942 | 3.306 | 0.525 | 0.644 | 18.686 | 0.389 |
| AIPW(0.5) | 1 | IPW | -0.126 | -0.126 | 0.195 | -0.002 | 1.046 | 0.000 | 0.937 | 3.424 | 0.945 | 3.232 | 0.940 | 3.359 | 0.747 | 1.625 | 38.046 | 0.770 |
| | 1 | WIPW | -0.126 | -0.126 | 0.318 | -0.002 | 1.170 | 0.000 | 0.911 | 4.018 | 0.927 | 3.686 | 0.923 | 3.760 | 1.141 | 4.025 | 101.388 | 2.109 |
| | 1 | AIPW | -0.126 | -0.124 | 0.157 | 0.007 | 1.043 | 0.002 | 0.939 | 3.385 | 0.947 | 3.162 | 0.942 | 3.300 | 0.600 | 1.199 | 24.599 | 0.496 |
| | 1 | WAIPW | -0.126 | -0.123 | 0.147 | 0.014 | 1.032 | 0.002 | 0.943 | 3.286 | 0.945 | 3.225 | 0.947 | 3.176 | 0.568 | 0.854 | 21.726 | 0.433 |
| AIPW(0.75) | 1 | IPW | -0.126 | -0.126 | 0.251 | 0.003 | 1.124 | 0.000 | 0.921 | 3.815 | 0.928 | 3.647 | 0.926 | 3.693 | 0.909 | 3.157 | 63.008 | 1.261 |
| | 1 | WIPW | -0.126 | -0.130 | 0.406 | -0.008 | 1.329 | -0.004 | 0.867 | 4.803 | 0.900 | 4.251 | 0.897 | 4.306 | 1.333 | 6.829 | 164.674 | 3.349 |
| | 1 | AIPW | -0.126 | -0.127 | 0.198 | -0.016 | 1.067 | -0.001 | 0.937 | 3.447 | 0.944 | 3.241 | 0.934 | 3.502 | 0.743 | 2.664 | 39.278 | 0.808 |
| | 1 | WAIPW | -0.126 | -0.126 | 0.161 | -0.001 | 1.039 | 0.000 | 0.939 | 3.375 | 0.945 | 3.235 | 0.945 | 3.230 | 0.615 | 1.232 | 25.810 | 0.520 |
| AIPW(1) | 1 | IPW | -0.126 | -0.127 | 0.344 | 0.006 | 1.248 | -0.002 | 0.894 | 4.351 | 0.910 | 4.038 | 0.910 | 4.044 | 1.092 | 6.412 | 118.406 | 2.554 |
| | 1 | WIPW | -0.126 | -0.129 | 0.499 | 0.004 | 1.516 | -0.003 | 0.817 | 5.463 | 0.864 | 4.841 | 0.865 | 4.831 | 1.355 | 10.192 | 249.117 | 5.480 |
| | 1 | AIPW | -0.126 | -0.124 | 0.279 | -0.011 | 1.100 | 0.002 | 0.935 | 3.479 | 0.942 | 3.302 | 0.932 | 3.561 | 0.973 | 6.381 | 78.068 | 1.869 |
| | 1 | WAIPW | -0.126 | -0.125 | 0.174 | 0.005 | 1.045 | 0.001 | 0.940 | 3.350 | 0.945 | 3.220 | 0.944 | 3.238 | 0.656 | 1.870 | 30.139 | 0.640 |
| AR-1 | 1 | IPW | -0.126 | -0.124 | 0.272 | 0.021 | 1.155 | 0.002 | 0.911 | 4.027 | 0.925 | 3.721 | 0.925 | 3.721 | 0.953 | 4.207 | 74.125 | 1.594 |
| | 1 | WIPW | -0.126 | -0.128 | 0.196 | -0.004 | 1.057 | -0.002 | 0.936 | 3.452 | 0.943 | 3.284 | 0.939 | 3.375 | 0.739 | 1.668 | 38.282 | 0.813 |
| | 1 | AIPW | -0.126 | -0.125 | 0.222 | -0.007 | 1.095 | 0.000 | 0.928 | 3.665 | 0.935 | 3.487 | 0.933 | 3.536 | 0.806 | 3.968 | 49.482 | 1.058 |
| | 1 | WAIPW | -0.126 | -0.128 | 0.183 | -0.018 | 1.070 | -0.002 | 0.934 | 3.517 | 0.941 | 3.327 | 0.936 | 3.452 | 0.683 | 2.392 | 33.659 | 0.697 |
| AR-2 | 1 | IPW | -0.126 | -0.120 | 0.260 | 0.045 | 1.141 | 0.006 | 0.919 | 3.850 | 0.922 | 3.802 | 0.935 | 3.482 | 0.916 | 3.840 | 67.753 | 1.478 |
| | 1 | WIPW | -0.126 | -0.124 | 0.191 | 0.023 | 1.050 | 0.002 | 0.938 | 3.411 | 0.939 | 3.395 | 0.943 | 3.274 | 0.724 | 1.641 | 36.650 | 0.777 |
| | 1 | AIPW | -0.126 | -0.123 | 0.216 | 0.007 | 1.089 | 0.003 | 0.931 | 3.590 | 0.939 | 3.390 | 0.935 | 3.487 | 0.782 | 4.064 | 46.609 | 1.189 |
| | 1 | WAIPW | -0.126 | -0.127 | 0.185 | -0.010 | 1.062 | -0.001 | 0.940 | 3.369 | 0.941 | 3.322 | 0.939 | 3.390 | 0.695 | 2.576 | 34.259 | 0.702 |
| IAIPW(0.25) | 1 | IPW | -0.126 | -0.124 | 0.171 | 0.013 | 1.031 | 0.001 | 0.941 | 3.333 | 0.936 | 3.462 | 0.949 | 3.100 | 0.652 | 0.997 | 29.085 | 0.583 |
| | 1 | WIPW | -0.126 | -0.125 | 0.168 | 0.010 | 1.029 | 0.001 | 0.943 | 3.268 | 0.940 | 3.359 | 0.950 | 3.088 | 0.644 | 0.947 | 28.285 | 0.568 |
| | 1 | AIPW | -0.126 | -0.125 | 0.138 | -0.001 | 1.044 | 0.000 | 0.941 | 3.338 | 0.943 | 3.290 | 0.944 | 3.263 | 0.527 | 0.688 | 19.158 | 0.371 |
| | 1 | WAIPW | -0.126 | -0.125 | 0.138 | -0.001 | 1.042 | 0.000 | 0.942 | 3.295 | 0.942 | 3.306 | 0.943 | 3.274 | 0.526 | 0.651 | 19.081 | 0.368 |
| IAIPW(0.5) | 1 | IPW | -0.126 | -0.122 | 0.194 | 0.018 | 1.038 | 0.003 | 0.942 | 3.313 | 0.944 | 3.239 | 0.943 | 3.286 | 0.750 | 1.543 | 37.743 | 0.765 |
| | 1 | WIPW | -0.126 | -0.123 | 0.179 | 0.013 | 1.023 | 0.002 | 0.945 | 3.225 | 0.948 | 3.148 | 0.946 | 3.190 | 0.697 | 1.132 | 31.970 | 0.632 |
| | 1 | AIPW | -0.126 | -0.125 | 0.156 | -0.002 | 1.033 | 0.001 | 0.940 | 3.346 | 0.947 | 3.176 | 0.942 | 3.306 | 0.602 | 1.189 | 24.358 | 0.491 |
| | 1 | WAIPW | -0.126 | -0.125 | 0.146 | 0.002 | 1.021 | 0.001 | 0.945 | 3.218 | 0.947 | 3.155 | 0.944 | 3.239 | 0.569 | 0.846 | 21.386 | 0.427 |
| IAIPW(0.75) | 1 | IPW | -0.126 | -0.122 | 0.251 | 0.017 | 1.118 | 0.004 | 0.920 | 3.837 | 0.925 | 3.716 | 0.935 | 3.477 | 0.910 | 3.057 | 62.883 | 1.233 |
| | 1 | WIPW | -0.126 | -0.121 | 0.197 | 0.027 | 1.050 | 0.005 | 0.936 | 3.467 | 0.935 | 3.477 | 0.945 | 3.213 | 0.749 | 1.469 | 38.787 | 0.777 |
| | 1 | AIPW | -0.126 | -0.124 | 0.200 | 0.000 | 1.076 | 0.001 | 0.938 | 3.401 | 0.939 | 3.380 | 0.936 | 3.472 | 0.743 | 2.647 | 40.106 | 0.829 |

| Randomization Method | Regime | Estimator | True Value | Mean Estimate | SD Estimates | Mean Normalized | SD Normalized | Mean Bias | CI Coverage | SE CI Coverage x 10^3 | LB Coverage | SE LB Coverage x 10^3 | UB Coverage | SE UB Coverage x 10^3 | Mean CI Length | SE CI Length x 10^3 | MSE x 10^3 | SE MSE x 10^3 |
|---|---|---|---|---|---|---|---|---|---|---|---|---|---|---|---|---|---|---|
| | 1 | WAIPW | -0.126 | -0.124 | 0.161 | 0.012 | 1.043 | 0.002 | 0.944 | 3.263 | 0.940 | 3.354 | 0.946 | 3.197 | 0.614 | 1.227 | 25.861 | 0.518 |
| IAIPW(1) | 1 | IPW | -0.126 | -0.122 | 0.337 | 0.010 | 1.255 | 0.004 | 0.887 | 4.484 | 0.907 | 4.108 | 0.909 | 4.065 | 1.081 | 6.293 | 113.874 | 2.351 |
| | 1 | WIPW | -0.126 | -0.124 | 0.210 | 0.009 | 1.067 | 0.001 | 0.935 | 3.487 | 0.937 | 3.428 | 0.942 | 3.297 | 0.788 | 1.980 | 44.282 | 0.878 |
| | 1 | AIPW | -0.126 | -0.122 | 0.273 | -0.010 | 1.105 | 0.004 | 0.937 | 3.437 | 0.940 | 3.359 | 0.937 | 3.437 | 0.966 | 6.232 | 74.670 | 1.679 |
| | 1 | WAIPW | -0.126 | -0.123 | 0.175 | 0.009 | 1.069 | 0.002 | 0.936 | 3.466 | 0.937 | 3.445 | 0.941 | 3.337 | 0.653 | 1.833 | 30.765 | 0.632 |
| IPW(0.25) | 1 | IPW | -0.126 | -0.129 | 0.171 | -0.022 | 1.040 | -0.003 | 0.940 | 3.364 | 0.945 | 3.224 | 0.938 | 3.406 | 0.651 | 0.995 | 29.193 | 0.593 |
| | 1 | WIPW | -0.126 | -0.128 | 0.251 | -0.008 | 1.071 | -0.002 | 0.931 | 3.580 | 0.942 | 3.295 | 0.939 | 3.375 | 0.954 | 2.455 | 62.752 | 1.291 |
| | 1 | AIPW | -0.126 | -0.126 | 0.137 | -0.006 | 1.035 | 0.000 | 0.939 | 3.380 | 0.947 | 3.157 | 0.940 | 3.348 | 0.526 | 0.693 | 18.802 | 0.373 |
| | 1 | WAIPW | -0.126 | -0.126 | 0.137 | -0.004 | 1.033 | 0.000 | 0.941 | 3.333 | 0.948 | 3.152 | 0.940 | 3.359 | 0.526 | 0.653 | 18.717 | 0.371 |
| IPW(0.5) | 1 | IPW | -0.126 | -0.129 | 0.196 | -0.018 | 1.044 | -0.004 | 0.939 | 3.379 | 0.945 | 3.218 | 0.942 | 3.293 | 0.751 | 1.592 | 38.379 | 0.781 |
| | 1 | WIPW | -0.126 | -0.132 | 0.316 | -0.018 | 1.153 | -0.006 | 0.914 | 3.966 | 0.927 | 3.674 | 0.933 | 3.531 | 1.150 | 3.962 | 99.709 | 2.033 |
| | 1 | AIPW | -0.126 | -0.126 | 0.157 | -0.010 | 1.046 | -0.001 | 0.935 | 3.481 | 0.944 | 3.239 | 0.937 | 3.437 | 0.602 | 1.208 | 24.756 | 0.487 |
| | 1 | WAIPW | -0.126 | -0.126 | 0.148 | -0.003 | 1.039 | 0.000 | 0.943 | 3.280 | 0.944 | 3.246 | 0.942 | 3.313 | 0.569 | 0.865 | 21.968 | 0.431 |
| IPW(0.75) | 1 | IPW | -0.126 | -0.129 | 0.251 | -0.020 | 1.111 | -0.004 | 0.924 | 3.739 | 0.929 | 3.632 | 0.933 | 3.526 | 0.913 | 3.195 | 63.168 | 1.284 |
| | 1 | WIPW | -0.126 | -0.132 | 0.405 | -0.026 | 1.316 | -0.006 | 0.870 | 4.750 | 0.903 | 4.194 | 0.900 | 4.247 | 1.341 | 6.885 | 163.943 | 3.330 |
| | 1 | AIPW | -0.126 | -0.129 | 0.200 | -0.036 | 1.062 | -0.003 | 0.938 | 3.421 | 0.943 | 3.268 | 0.933 | 3.531 | 0.744 | 2.719 | 39.869 | 0.848 |
| | 1 | WAIPW | -0.126 | -0.129 | 0.162 | -0.028 | 1.042 | -0.003 | 0.936 | 3.457 | 0.943 | 3.268 | 0.937 | 3.426 | 0.616 | 1.257 | 26.212 | 0.537 |
| IPW(1) | 1 | IPW | -0.126 | -0.124 | 0.340 | 0.011 | 1.241 | 0.001 | 0.894 | 4.357 | 0.909 | 4.068 | 0.910 | 4.038 | 1.093 | 6.457 | 115.677 | 2.532 |
| | 1 | WIPW | -0.126 | -0.126 | 0.492 | 0.003 | 1.512 | 0.000 | 0.820 | 5.430 | 0.869 | 4.775 | 0.862 | 4.871 | 1.353 | 10.164 | 242.125 | 5.334 |
| | 1 | AIPW | -0.126 | -0.129 | 0.277 | -0.032 | 1.092 | -0.004 | 0.940 | 3.350 | 0.945 | 3.229 | 0.932 | 3.557 | 0.980 | 6.336 | 76.841 | 1.805 |
| | 1 | WAIPW | -0.126 | -0.125 | 0.173 | 0.002 | 1.042 | 0.001 | 0.937 | 3.441 | 0.945 | 3.225 | 0.941 | 3.333 | 0.658 | 1.884 | 29.985 | 0.628 |
| SR | 1 | IPW | -0.126 | -0.127 | 0.154 | -0.006 | 1.017 | -0.001 | 0.945 | 3.214 | 0.949 | 3.112 | 0.947 | 3.169 | 0.601 | 0.796 | 23.777 | 0.477 |
| | 1 | WIPW | -0.126 | -0.125 | 0.216 | 0.003 | 1.061 | 0.000 | 0.937 | 3.437 | 0.944 | 3.252 | 0.941 | 3.333 | 0.819 | 1.735 | 46.544 | 0.948 |
| | 1 | AIPW | -0.126 | -0.124 | 0.125 | 0.011 | 1.016 | 0.001 | 0.946 | 3.208 | 0.944 | 3.263 | 0.951 | 3.053 | 0.491 | 0.540 | 15.739 | 0.311 |
| | 1 | WAIPW | -0.126 | -0.124 | 0.127 | 0.013 | 1.019 | 0.002 | 0.943 | 3.285 | 0.946 | 3.203 | 0.950 | 3.071 | 0.495 | 0.545 | 16.096 | 0.320 |
| TS(0.25) | 1 | IPW | -0.126 | -0.130 | 0.385 | -0.020 | 1.939 | -0.005 | 0.856 | 4.963 | 0.898 | 4.281 | 0.892 | 4.390 | 0.863 | 6.080 | 148.544 | 6.230 |
| | 1 | WIPW | -0.126 | -0.120 | 0.210 | 0.013 | 1.050 | 0.005 | 0.941 | 3.343 | 0.941 | 3.338 | 0.940 | 3.354 | 0.773 | 2.485 | 44.311 | 1.062 |
| | 1 | AIPW | -0.126 | -0.124 | 1.023 | 0.061 | 1.160 | 0.002 | 0.920 | 3.841 | 0.925 | 3.725 | 0.940 | 3.348 | 1.203 | 54.008 | 1046.406 | 467.843 |
| | 1 | WAIPW | -0.126 | -0.118 | 0.191 | 0.045 | 1.049 | 0.007 | 0.943 | 3.279 | 0.939 | 3.385 | 0.951 | 3.041 | 0.669 | 4.623 | 36.694 | 1.711 |
| TS(0.50) | 1 | IPW | -0.126 | -0.124 | 0.396 | 0.021 | 2.016 | 0.002 | 0.848 | 5.083 | 0.896 | 4.317 | 0.889 | 4.439 | 0.924 | 6.006 | 156.572 | 6.296 |
| | 1 | WIPW | -0.126 | -0.115 | 0.223 | 0.032 | 1.054 | 0.010 | 0.939 | 3.375 | 0.935 | 3.482 | 0.947 | 3.157 | 0.814 | 2.716 | 49.823 | 1.206 |
| | 1 | AIPW | -0.126 | -0.103 | 0.825 | 0.118 | 1.190 | 0.022 | 0.913 | 3.982 | 0.910 | 4.052 | 0.945 | 3.224 | 1.285 | 41.525 | 681.675 | 164.144 |
| | 1 | WAIPW | -0.126 | -0.115 | 0.200 | 0.062 | 1.055 | 0.010 | 0.940 | 3.369 | 0.936 | 3.452 | 0.949 | 3.100 | 0.698 | 4.765 | 39.900 | 1.455 |
| TS(0.75) | 1 | IPW | -0.126 | -0.163 | 0.412 | -0.163 | 2.176 | -0.037 | 0.843 | 5.148 | 0.908 | 4.096 | 0.862 | 4.884 | 0.940 | 6.166 | 171.192 | 7.077 |
| | 1 | WIPW | -0.126 | -0.130 | 0.228 | -0.037 | 1.074 | -0.004 | 0.931 | 3.594 | 0.946 | 3.208 | 0.932 | 3.561 | 0.824 | 2.628 | 52.063 | 1.229 |
| | 1 | AIPW | -0.126 | -0.148 | 1.788 | 0.089 | 1.230 | -0.023 | 0.898 | 4.288 | 0.903 | 4.182 | 0.932 | 3.556 | 1.455 | 96.942 | 3195.030 | 1952.725 |
| | 1 | WAIPW | -0.126 | -0.123 | 0.229 | 0.030 | 1.067 | 0.003 | 0.934 | 3.521 | 0.939 | 3.390 | 0.940 | 3.359 | 0.712 | 7.754 | 52.247 | 8.529 |
| TS(1) | 1 | IPW | -0.126 | -0.142 | 0.440 | -0.045 | 2.748 | -0.016 | 0.839 | 5.201 | 0.896 | 4.317 | 0.875 | 4.681 | 0.962 | 6.751 | 193.477 | 8.049 |
| | 1 | WIPW | -0.126 | -0.119 | 0.239 | 0.016 | 1.088 | 0.007 | 0.930 | 3.613 | 0.936 | 3.467 | 0.940 | 3.359 | 0.841 | 2.949 | 56.992 | 1.529 |
| | 1 | AIPW | -0.126 | -0.140 | 2.040 | 0.142 | 1.250 | -0.014 | 0.899 | 4.269 | 0.896 | 4.310 | 0.939 | 3.390 | 1.675 | 110.525 | 4160.988 | 1504.744 |
| | 1 | WAIPW | -0.126 | -0.120 | 0.238 | 0.046 | 1.067 | 0.005 | 0.934 | 3.512 | 0.934 | 3.512 | 0.943 | 3.284 | 0.735 | 8.037 | 56.617 | 5.492 |
| WAIPW(0.25) | 1 | IPW | -0.126 | -0.125 | 0.169 | 0.008 | 1.026 | 0.001 | 0.944 | 3.257 | 0.946 | 3.191 | 0.946 | 3.202 | 0.652 | 0.985 | 28.518 | 0.570 |
| | 1 | WIPW | -0.126 | -0.124 | 0.166 | 0.010 | 1.023 | 0.001 | 0.944 | 3.252 | 0.946 | 3.186 | 0.947 | 3.169 | 0.643 | 0.934 | 27.719 | 0.554 |
| | 1 | AIPW | -0.126 | -0.125 | 0.137 | 0.006 | 1.030 | 0.001 | 0.939 | 3.395 | 0.943 | 3.284 | 0.942 | 3.306 | 0.527 | 0.694 | 18.733 | 0.374 |
| | 1 | WAIPW | -0.126 | -0.125 | 0.136 | 0.008 | 1.024 | 0.001 | 0.941 | 3.333 | 0.945 | 3.230 | 0.946 | 3.202 | 0.526 | 0.653 | 18.516 | 0.372 |

| Randomization Method | Regime | Estimator | True Value | Mean Estimate | SD Estimates | Mean Normalized | SD Normalized | Mean Bias | CI Coverage | SE CI Coverage x 10^3 | LB Coverage | SE LB Coverage x 10^3 | UB Coverage | SE UB Coverage x 10^3 | Mean CI Length | SE CI Length x 10^3 | MSE x 10^3 | SE MSE x 10^3 |
|---|---|---|---|---|---|---|---|---|---|---|---|---|---|---|---|---|---|---|
| WAIPW(0.5) | 1 | IPW | -0.126 | -0.126 | 0.198 | 0.002 | 1.057 | 0.000 | 0.934 | 3.518 | 0.943 | 3.286 | 0.938 | 3.411 | 0.749 | 1.545 | 39.306 | 0.765 |
| | 1 | WIPW | -0.126 | -0.125 | 0.183 | 0.003 | 1.041 | 0.000 | 0.943 | 3.273 | 0.945 | 3.232 | 0.941 | 3.326 | 0.696 | 1.142 | 33.319 | 0.652 |
| | 1 | AIPW | -0.126 | -0.124 | 0.156 | 0.013 | 1.038 | 0.002 | 0.939 | 3.379 | 0.941 | 3.320 | 0.942 | 3.306 | 0.599 | 1.185 | 24.409 | 0.482 |
| | 1 | WAIPW | -0.126 | -0.123 | 0.147 | 0.021 | 1.032 | 0.003 | 0.942 | 3.306 | 0.940 | 3.353 | 0.948 | 3.126 | 0.567 | 0.843 | 21.714 | 0.436 |
| WAIPW(0.75) | 1 | IPW | -0.126 | -0.124 | 0.252 | 0.006 | 1.128 | 0.001 | 0.921 | 3.819 | 0.928 | 3.665 | 0.932 | 3.565 | 0.909 | 3.080 | 63.307 | 1.258 |
| | 1 | WIPW | -0.126 | -0.126 | 0.200 | -0.004 | 1.069 | 0.000 | 0.936 | 3.472 | 0.938 | 3.401 | 0.938 | 3.411 | 0.748 | 1.493 | 39.997 | 0.789 |
| | 1 | AIPW | -0.126 | -0.125 | 0.199 | -0.010 | 1.080 | 0.001 | 0.927 | 3.670 | 0.936 | 3.457 | 0.935 | 3.477 | 0.737 | 2.652 | 39.734 | 0.837 |
| | 1 | WAIPW | -0.126 | -0.126 | 0.163 | -0.009 | 1.057 | 0.000 | 0.931 | 3.575 | 0.939 | 3.375 | 0.938 | 3.411 | 0.613 | 1.230 | 26.543 | 0.541 |
| WAIPW(1) | 1 | IPW | -0.126 | -0.128 | 0.344 | -0.007 | 1.250 | -0.003 | 0.886 | 4.501 | 0.909 | 4.075 | 0.904 | 4.160 | 1.086 | 6.365 | 118.330 | 2.622 |
| | 1 | WIPW | -0.126 | -0.127 | 0.213 | -0.006 | 1.067 | -0.001 | 0.931 | 3.585 | 0.939 | 3.394 | 0.939 | 3.390 | 0.790 | 2.053 | 45.257 | 0.914 |
| | 1 | AIPW | -0.126 | -0.125 | 0.274 | -0.025 | 1.082 | 0.001 | 0.942 | 3.293 | 0.944 | 3.261 | 0.935 | 3.487 | 0.971 | 6.250 | 75.174 | 1.745 |
| | 1 | WAIPW | -0.126 | -0.124 | 0.174 | 0.003 | 1.053 | 0.002 | 0.937 | 3.437 | 0.944 | 3.252 | 0.941 | 3.333 | 0.655 | 1.867 | 30.365 | 0.628 |
| WIPW(0.25) | 1 | IPW | -0.126 | -0.125 | 0.167 | 0.005 | 1.017 | 0.001 | 0.943 | 3.274 | 0.944 | 3.241 | 0.952 | 3.035 | 0.650 | 0.971 | 27.832 | 0.554 |
| | 1 | WIPW | -0.126 | -0.126 | 0.165 | 0.002 | 1.016 | 0.000 | 0.944 | 3.257 | 0.946 | 3.208 | 0.952 | 3.029 | 0.642 | 0.922 | 27.115 | 0.541 |
| | 1 | AIPW | -0.126 | -0.125 | 0.136 | 0.001 | 1.030 | 0.000 | 0.942 | 3.295 | 0.945 | 3.224 | 0.952 | 3.035 | 0.526 | 0.693 | 18.592 | 0.367 |
| | 1 | WAIPW | -0.126 | -0.126 | 0.136 | 0.000 | 1.028 | 0.000 | 0.943 | 3.279 | 0.945 | 3.219 | 0.951 | 3.047 | 0.526 | 0.653 | 18.504 | 0.363 |
| WIPW(0.5) | 1 | IPW | -0.126 | -0.121 | 0.197 | 0.021 | 1.045 | 0.004 | 0.939 | 3.385 | 0.942 | 3.306 | 0.945 | 3.225 | 0.750 | 1.582 | 38.742 | 0.796 |
| | 1 | WIPW | -0.126 | -0.122 | 0.180 | 0.018 | 1.028 | 0.003 | 0.946 | 3.190 | 0.945 | 3.211 | 0.947 | 3.162 | 0.697 | 1.166 | 32.561 | 0.658 |
| | 1 | AIPW | -0.126 | -0.126 | 0.157 | -0.004 | 1.037 | 0.000 | 0.940 | 3.359 | 0.944 | 3.239 | 0.943 | 3.273 | 0.602 | 1.205 | 24.759 | 0.507 |
| | 1 | WAIPW | -0.126 | -0.125 | 0.147 | 0.001 | 1.025 | 0.001 | 0.944 | 3.259 | 0.949 | 3.098 | 0.945 | 3.232 | 0.569 | 0.852 | 21.659 | 0.441 |
| WIPW(0.75) | 1 | IPW | -0.126 | -0.127 | 0.255 | -0.005 | 1.141 | -0.001 | 0.917 | 3.902 | 0.926 | 3.702 | 0.930 | 3.599 | 0.911 | 3.233 | 65.262 | 1.299 |
| | 1 | WIPW | -0.126 | -0.127 | 0.202 | -0.005 | 1.074 | -0.001 | 0.931 | 3.590 | 0.937 | 3.447 | 0.938 | 3.411 | 0.750 | 1.537 | 40.790 | 0.817 |
| | 1 | AIPW | -0.126 | -0.128 | 0.201 | -0.021 | 1.075 | -0.002 | 0.934 | 3.507 | 0.942 | 3.306 | 0.936 | 3.462 | 0.743 | 2.699 | 40.340 | 0.846 |
| | 1 | WAIPW | -0.126 | -0.127 | 0.163 | -0.010 | 1.054 | -0.001 | 0.936 | 3.452 | 0.939 | 3.385 | 0.941 | 3.338 | 0.615 | 1.253 | 26.525 | 0.547 |
| WIPW(1) | 1 | IPW | -0.126 | -0.124 | 0.332 | 0.008 | 1.243 | 0.002 | 0.887 | 4.469 | 0.910 | 4.055 | 0.909 | 4.061 | 1.084 | 6.421 | 110.362 | 2.361 |
| | 1 | WIPW | -0.126 | -0.123 | 0.209 | 0.011 | 1.056 | 0.003 | 0.934 | 3.512 | 0.941 | 3.342 | 0.943 | 3.266 | 0.790 | 2.032 | 43.725 | 0.891 |
| | 1 | AIPW | -0.126 | -0.123 | 0.274 | -0.005 | 1.097 | 0.003 | 0.939 | 3.394 | 0.939 | 3.390 | 0.935 | 3.474 | 0.973 | 6.368 | 75.146 | 1.703 |
| | 1 | WAIPW | -0.126 | -0.123 | 0.174 | 0.013 | 1.053 | 0.003 | 0.945 | 3.229 | 0.941 | 3.324 | 0.948 | 3.136 | 0.656 | 1.858 | 30.126 | 0.613 |
| AIPW(0.25) | 2 | IPW | -0.374 | -0.375 | 0.160 | -0.003 | 1.020 | 0.000 | 0.942 | 3.306 | 0.947 | 3.180 | 0.947 | 3.163 | 0.620 | 0.933 | 25.497 | 0.523 |
| | 2 | WIPW | -0.374 | -0.375 | 0.241 | -0.010 | 1.083 | -0.001 | 0.932 | 3.570 | 0.941 | 3.338 | 0.938 | 3.421 | 0.903 | 2.301 | 57.955 | 1.146 |
| | 2 | AIPW | -0.374 | -0.378 | 0.133 | -0.029 | 1.042 | -0.004 | 0.938 | 3.416 | 0.946 | 3.186 | 0.941 | 3.338 | 0.510 | 0.682 | 17.684 | 0.358 |
| | 2 | WAIPW | -0.374 | -0.378 | 0.133 | -0.031 | 1.037 | -0.004 | 0.941 | 3.338 | 0.949 | 3.123 | 0.941 | 3.333 | 0.511 | 0.642 | 17.621 | 0.358 |
| AIPW(0.5) | 2 | IPW | -0.374 | -0.375 | 0.185 | 0.000 | 1.045 | -0.001 | 0.940 | 3.366 | 0.942 | 3.300 | 0.941 | 3.326 | 0.712 | 1.513 | 34.202 | 0.650 |
| | 2 | WIPW | -0.374 | -0.375 | 0.299 | -0.001 | 1.163 | -0.001 | 0.907 | 4.113 | 0.926 | 3.691 | 0.922 | 3.799 | 1.087 | 3.741 | 89.508 | 1.764 |
| | 2 | AIPW | -0.374 | -0.377 | 0.153 | -0.024 | 1.067 | -0.003 | 0.934 | 3.512 | 0.946 | 3.183 | 0.934 | 3.506 | 0.580 | 1.189 | 23.385 | 0.454 |
| | 2 | WAIPW | -0.374 | -0.377 | 0.145 | -0.023 | 1.053 | -0.003 | 0.939 | 3.385 | 0.945 | 3.232 | 0.936 | 3.456 | 0.551 | 0.852 | 20.943 | 0.405 |
| AIPW(0.75) | 2 | IPW | -0.374 | -0.378 | 0.240 | -0.015 | 1.105 | -0.004 | 0.927 | 3.679 | 0.932 | 3.561 | 0.931 | 3.590 | 0.874 | 3.006 | 57.591 | 1.179 |
| | 2 | WIPW | -0.374 | -0.378 | 0.386 | -0.010 | 1.305 | -0.004 | 0.876 | 4.668 | 0.906 | 4.131 | 0.904 | 4.170 | 1.287 | 6.512 | 149.071 | 3.037 |
| | 2 | AIPW | -0.374 | -0.379 | 0.193 | -0.042 | 1.067 | -0.005 | 0.937 | 3.441 | 0.947 | 3.180 | 0.931 | 3.590 | 0.723 | 2.647 | 37.178 | 0.768 |
| | 2 | WAIPW | -0.374 | -0.379 | 0.157 | -0.036 | 1.047 | -0.005 | 0.943 | 3.268 | 0.949 | 3.106 | 0.937 | 3.426 | 0.599 | 1.231 | 24.797 | 0.501 |
| AIPW(1) | 2 | IPW | -0.374 | -0.376 | 0.325 | 0.004 | 1.235 | -0.002 | 0.891 | 4.405 | 0.911 | 4.024 | 0.911 | 4.028 | 1.033 | 6.121 | 105.415 | 2.364 |
| | 2 | WIPW | -0.374 | -0.377 | 0.470 | 0.005 | 1.504 | -0.003 | 0.821 | 5.424 | 0.869 | 4.770 | 0.868 | 4.790 | 1.276 | 9.690 | 221.003 | 5.090 |
| | 2 | AIPW | -0.374 | -0.375 | 0.268 | -0.027 | 1.130 | -0.001 | 0.930 | 3.613 | 0.936 | 3.453 | 0.928 | 3.656 | 0.929 | 6.305 | 71.825 | 1.624 |
| | 2 | WAIPW | -0.374 | -0.375 | 0.170 | -0.010 | 1.069 | -0.001 | 0.938 | 3.398 | 0.943 | 3.284 | 0.936 | 3.449 | 0.633 | 1.869 | 29.020 | 0.595 |
| AR-1 | 2 | IPW | -0.374 | -0.384 | 0.329 | -0.074 | 1.255 | -0.009 | 0.887 | 4.471 | 0.921 | 3.819 | 0.894 | 4.361 | 1.000 | 5.995 | 108.499 | 2.732 |

| Randomization Method | Regime | Estimator | True Value | Mean Estimate | SD Estimates | Mean Normalized | SD Normalized | Mean Bias | CI Coverage | SE CI Coverage x 10^3 | LB Coverage | SE LB Coverage x 10^3 | UB Coverage | SE UB Coverage x 10^3 | Mean CI Length | SE CI Length x 10^3 | MSE x 10^3 | SE MSE x 10^3 |
|---|---|---|---|---|---|---|---|---|---|---|---|---|---|---|---|---|---|---|
| | 2 | WIPW | -0.374 | -0.382 | 0.208 | -0.047 | 1.081 | -0.008 | 0.929 | 3.632 | 0.946 | 3.197 | 0.931 | 3.580 | 0.761 | 2.281 | 43.481 | 0.923 |
| | 2 | AIPW | -0.374 | -0.376 | 0.284 | -0.048 | 1.158 | -0.001 | 0.922 | 3.793 | 0.938 | 3.411 | 0.924 | 3.743 | 0.923 | 8.213 | 80.686 | 2.600 |
| | 2 | WAIPW | -0.374 | -0.381 | 0.184 | -0.050 | 1.087 | -0.007 | 0.929 | 3.628 | 0.943 | 3.290 | 0.932 | 3.565 | 0.671 | 2.964 | 33.771 | 0.804 |
| AR-2 | 2 | IPW | -0.374 | -0.385 | 0.362 | -0.109 | 1.352 | -0.010 | 0.868 | 4.794 | 0.907 | 4.112 | 0.882 | 4.570 | 0.968 | 6.265 | 130.974 | 4.413 |
| | 2 | WIPW | -0.374 | -0.376 | 0.216 | -0.031 | 1.072 | -0.002 | 0.932 | 3.551 | 0.945 | 3.224 | 0.931 | 3.585 | 0.778 | 2.956 | 46.438 | 1.064 |
| | 2 | AIPW | -0.374 | -0.373 | 0.401 | -0.063 | 1.212 | 0.001 | 0.907 | 4.116 | 0.931 | 3.580 | 0.913 | 3.982 | 1.029 | 16.477 | 160.635 | 10.116 |
| | 2 | WAIPW | -0.374 | -0.378 | 0.198 | -0.034 | 1.102 | -0.004 | 0.927 | 3.689 | 0.940 | 3.348 | 0.931 | 3.585 | 0.690 | 4.529 | 39.037 | 1.075 |
| IAIPW(0.25) | 2 | IPW | -0.374 | -0.380 | 0.156 | -0.034 | 0.998 | -0.006 | 0.947 | 3.163 | 0.955 | 2.944 | 0.951 | 3.059 | 0.620 | 0.934 | 24.461 | 0.501 |
| | 2 | WIPW | -0.374 | -0.380 | 0.154 | -0.035 | 0.997 | -0.006 | 0.947 | 3.157 | 0.954 | 2.957 | 0.951 | 3.047 | 0.612 | 0.883 | 23.816 | 0.490 |
| | 2 | AIPW | -0.374 | -0.378 | 0.131 | -0.033 | 1.021 | -0.004 | 0.945 | 3.230 | 0.948 | 3.129 | 0.943 | 3.290 | 0.510 | 0.695 | 17.055 | 0.347 |
| | 2 | WAIPW | -0.374 | -0.378 | 0.130 | -0.029 | 1.017 | -0.004 | 0.943 | 3.290 | 0.950 | 3.083 | 0.946 | 3.208 | 0.511 | 0.659 | 17.020 | 0.346 |
| IAIPW(0.5) | 2 | IPW | -0.374 | -0.370 | 0.188 | 0.017 | 1.041 | 0.004 | 0.936 | 3.450 | 0.942 | 3.293 | 0.942 | 3.313 | 0.715 | 1.526 | 35.270 | 0.721 |
| | 2 | WIPW | -0.374 | -0.371 | 0.172 | 0.018 | 1.022 | 0.003 | 0.940 | 3.359 | 0.939 | 3.379 | 0.946 | 3.190 | 0.664 | 1.125 | 29.540 | 0.607 |
| | 2 | AIPW | -0.374 | -0.374 | 0.155 | -0.005 | 1.065 | 0.000 | 0.931 | 3.591 | 0.940 | 3.346 | 0.938 | 3.418 | 0.582 | 1.206 | 23.971 | 0.495 |
| | 2 | WAIPW | -0.374 | -0.373 | 0.145 | 0.001 | 1.048 | 0.001 | 0.936 | 3.456 | 0.946 | 3.190 | 0.938 | 3.411 | 0.552 | 0.858 | 21.045 | 0.436 |
| IAIPW(0.75) | 2 | IPW | -0.374 | -0.373 | 0.238 | 0.007 | 1.112 | 0.001 | 0.920 | 3.846 | 0.930 | 3.618 | 0.934 | 3.512 | 0.865 | 2.997 | 56.726 | 1.160 |
| | 2 | WIPW | -0.374 | -0.373 | 0.189 | 0.005 | 1.049 | 0.001 | 0.938 | 3.406 | 0.939 | 3.395 | 0.939 | 3.375 | 0.713 | 1.451 | 35.561 | 0.719 |
| | 2 | AIPW | -0.374 | -0.376 | 0.196 | -0.017 | 1.098 | -0.002 | 0.926 | 3.698 | 0.941 | 3.333 | 0.934 | 3.517 | 0.716 | 2.626 | 38.504 | 0.818 |
| | 2 | WAIPW | -0.374 | -0.376 | 0.160 | -0.012 | 1.067 | -0.002 | 0.935 | 3.477 | 0.942 | 3.317 | 0.940 | 3.354 | 0.596 | 1.229 | 25.503 | 0.529 |
| IAIPW(1) | 2 | IPW | -0.374 | -0.363 | 0.322 | 0.040 | 1.233 | 0.011 | 0.891 | 4.411 | 0.910 | 4.044 | 0.914 | 3.955 | 1.033 | 6.031 | 103.697 | 2.280 |
| | 2 | WIPW | -0.374 | -0.367 | 0.200 | 0.039 | 1.061 | 0.007 | 0.934 | 3.520 | 0.939 | 3.394 | 0.942 | 3.315 | 0.751 | 1.904 | 39.939 | 0.825 |
| | 2 | AIPW | -0.374 | -0.367 | 0.265 | -0.007 | 1.130 | 0.007 | 0.925 | 3.726 | 0.931 | 3.581 | 0.933 | 3.545 | 0.933 | 6.199 | 70.376 | 1.563 |
| | 2 | WAIPW | -0.374 | -0.371 | 0.169 | 0.015 | 1.071 | 0.004 | 0.929 | 3.625 | 0.936 | 3.466 | 0.936 | 3.466 | 0.634 | 1.824 | 28.453 | 0.575 |
| IPW(0.25) | 2 | IPW | -0.374 | -0.374 | 0.159 | -0.002 | 1.012 | 0.000 | 0.949 | 3.112 | 0.950 | 3.088 | 0.948 | 3.129 | 0.622 | 0.937 | 25.239 | 0.509 |
| | 2 | WIPW | -0.374 | -0.377 | 0.237 | -0.014 | 1.053 | -0.003 | 0.938 | 3.401 | 0.944 | 3.252 | 0.940 | 3.354 | 0.909 | 2.317 | 56.063 | 1.140 |
| | 2 | AIPW | -0.374 | -0.375 | 0.132 | -0.007 | 1.026 | -0.001 | 0.945 | 3.230 | 0.947 | 3.180 | 0.944 | 3.246 | 0.511 | 0.687 | 17.306 | 0.346 |
| | 2 | WAIPW | -0.374 | -0.374 | 0.131 | -0.004 | 1.023 | 0.000 | 0.945 | 3.213 | 0.948 | 3.152 | 0.947 | 3.157 | 0.512 | 0.646 | 17.275 | 0.345 |
| IPW(0.5) | 2 | IPW | -0.374 | -0.373 | 0.185 | 0.008 | 1.040 | 0.001 | 0.935 | 3.475 | 0.944 | 3.239 | 0.942 | 3.300 | 0.714 | 1.500 | 34.285 | 0.694 |
| | 2 | WIPW | -0.374 | -0.371 | 0.300 | 0.015 | 1.154 | 0.003 | 0.910 | 4.038 | 0.926 | 3.691 | 0.931 | 3.573 | 1.092 | 3.712 | 90.201 | 1.875 |
| | 2 | AIPW | -0.374 | -0.373 | 0.153 | 0.000 | 1.055 | 0.001 | 0.938 | 3.418 | 0.947 | 3.169 | 0.939 | 3.379 | 0.584 | 1.207 | 23.502 | 0.474 |
| | 2 | WAIPW | -0.374 | -0.374 | 0.144 | -0.001 | 1.037 | 0.000 | 0.941 | 3.340 | 0.948 | 3.134 | 0.943 | 3.280 | 0.553 | 0.858 | 20.595 | 0.415 |
| IPW(0.75) | 2 | IPW | -0.374 | -0.379 | 0.234 | -0.026 | 1.092 | -0.005 | 0.929 | 3.623 | 0.940 | 3.354 | 0.932 | 3.570 | 0.873 | 3.042 | 54.970 | 1.131 |
| | 2 | WIPW | -0.374 | -0.382 | 0.381 | -0.031 | 1.299 | -0.008 | 0.876 | 4.661 | 0.912 | 4.015 | 0.894 | 4.350 | 1.284 | 6.602 | 145.038 | 2.988 |
| | 2 | AIPW | -0.374 | -0.376 | 0.192 | -0.033 | 1.080 | -0.002 | 0.933 | 3.541 | 0.947 | 3.180 | 0.929 | 3.632 | 0.722 | 2.687 | 36.828 | 0.762 |
| | 2 | WAIPW | -0.374 | -0.376 | 0.156 | -0.024 | 1.049 | -0.002 | 0.939 | 3.390 | 0.950 | 3.071 | 0.934 | 3.502 | 0.599 | 1.251 | 24.410 | 0.501 |
| IPW(1) | 2 | IPW | -0.374 | -0.380 | 0.321 | -0.005 | 1.216 | -0.006 | 0.896 | 4.312 | 0.914 | 3.962 | 0.911 | 4.017 | 1.039 | 6.017 | 103.226 | 2.233 |
| | 2 | WIPW | -0.374 | -0.384 | 0.464 | -0.006 | 1.480 | -0.010 | 0.829 | 5.327 | 0.873 | 4.707 | 0.866 | 4.811 | 1.289 | 9.531 | 215.353 | 4.735 |
| | 2 | AIPW | -0.374 | -0.382 | 0.264 | -0.048 | 1.109 | -0.008 | 0.933 | 3.536 | 0.944 | 3.243 | 0.924 | 3.741 | 0.937 | 6.227 | 69.825 | 1.607 |
| | 2 | WAIPW | -0.374 | -0.377 | 0.166 | -0.020 | 1.045 | -0.003 | 0.940 | 3.359 | 0.946 | 3.188 | 0.940 | 3.363 | 0.635 | 1.836 | 27.496 | 0.557 |
| SR | 2 | IPW | -0.374 | -0.378 | 0.147 | -0.023 | 1.011 | -0.003 | 0.949 | 3.112 | 0.952 | 3.030 | 0.944 | 3.258 | 0.574 | 0.761 | 21.657 | 0.420 |
| | 2 | WIPW | -0.374 | -0.375 | 0.202 | -0.002 | 1.028 | -0.001 | 0.941 | 3.322 | 0.948 | 3.141 | 0.949 | 3.100 | 0.783 | 1.639 | 40.829 | 0.816 |
| | 2 | AIPW | -0.374 | -0.375 | 0.122 | -0.005 | 1.020 | -0.001 | 0.945 | 3.236 | 0.944 | 3.252 | 0.947 | 3.175 | 0.476 | 0.542 | 14.964 | 0.291 |
| | 2 | WAIPW | -0.374 | -0.375 | 0.124 | -0.007 | 1.021 | -0.001 | 0.944 | 3.241 | 0.945 | 3.236 | 0.947 | 3.163 | 0.481 | 0.547 | 15.317 | 0.298 |
| TS(0.25) | 2 | IPW | -0.374 | -0.366 | 0.340 | 0.034 | 1.281 | 0.008 | 0.880 | 4.596 | 0.899 | 4.262 | 0.907 | 4.100 | 0.818 | 6.420 | 115.525 | 4.429 |
| | 2 | WIPW | -0.374 | -0.369 | 0.185 | 0.020 | 1.035 | 0.005 | 0.940 | 3.364 | 0.940 | 3.369 | 0.949 | 3.117 | 0.694 | 2.175 | 34.393 | 0.816 |

| Randomization Method | Regime | Estimator | True Value | Mean Estimate | SD Estimates | Mean Normalized | SD Normalized | Mean Bias | CI Coverage | SE CI Coverage x 10^3 | LB Coverage | SE LB Coverage x 10^3 | UB Coverage | SE UB Coverage x 10^3 | Mean CI Length | SE CI Length x 10^3 | MSE x 10^3 | SE MSE x 10^3 |
|---|---|---|---|---|---|---|---|---|---|---|---|---|---|---|---|---|---|---|
| | 2 | AIPW | -0.374 | -0.369 | 0.396 | 0.036 | 1.189 | 0.005 | 0.913 | 3.978 | 0.923 | 3.780 | 0.933 | 3.531 | 0.982 | 16.566 | 156.645 | 9.996 |
| | 2 | WAIPW | -0.374 | -0.368 | 0.178 | 0.032 | 1.063 | 0.006 | 0.935 | 3.492 | 0.935 | 3.482 | 0.945 | 3.219 | 0.632 | 4.038 | 31.832 | 0.888 |
| TS(0.50) | 2 | IPW | -0.374 | -0.352 | 0.350 | 0.080 | 1.407 | 0.022 | 0.870 | 4.750 | 0.888 | 4.467 | 0.905 | 4.147 | 0.860 | 6.391 | 123.191 | 4.545 |
| | 2 | WIPW | -0.374 | -0.364 | 0.196 | 0.039 | 1.060 | 0.010 | 0.934 | 3.502 | 0.929 | 3.623 | 0.944 | 3.241 | 0.718 | 2.294 | 38.430 | 0.864 |
| | 2 | AIPW | -0.374 | -0.366 | 0.437 | 0.048 | 1.201 | 0.008 | 0.907 | 4.100 | 0.914 | 3.957 | 0.934 | 3.502 | 1.017 | 18.940 | 190.635 | 17.016 |
| | 2 | WAIPW | -0.374 | -0.366 | 0.185 | 0.036 | 1.076 | 0.008 | 0.934 | 3.502 | 0.934 | 3.512 | 0.942 | 3.306 | 0.649 | 4.204 | 34.422 | 0.996 |
| TS(0.75) | 2 | IPW | -0.374 | -0.357 | 0.370 | 0.072 | 1.436 | 0.017 | 0.864 | 4.851 | 0.886 | 4.495 | 0.904 | 4.159 | 0.899 | 6.865 | 137.196 | 5.310 |
| | 2 | WIPW | -0.374 | -0.364 | 0.201 | 0.045 | 1.060 | 0.010 | 0.935 | 3.487 | 0.929 | 3.632 | 0.945 | 3.230 | 0.732 | 2.443 | 40.557 | 0.970 |
| | 2 | AIPW | -0.374 | -0.374 | 0.520 | 0.065 | 1.236 | 0.000 | 0.902 | 4.209 | 0.907 | 4.104 | 0.934 | 3.507 | 1.095 | 24.401 | 270.508 | 38.833 |
| | 2 | WAIPW | -0.374 | -0.365 | 0.197 | 0.053 | 1.096 | 0.010 | 0.930 | 3.613 | 0.930 | 3.604 | 0.944 | 3.246 | 0.674 | 4.909 | 38.983 | 1.214 |
| TS(1) | 2 | IPW | -0.374 | -0.353 | 0.382 | 0.114 | 1.648 | 0.021 | 0.860 | 4.902 | 0.877 | 4.642 | 0.910 | 4.048 | 0.934 | 7.083 | 145.989 | 5.303 |
| | 2 | WIPW | -0.374 | -0.365 | 0.207 | 0.055 | 1.068 | 0.009 | 0.941 | 3.343 | 0.935 | 3.477 | 0.949 | 3.100 | 0.751 | 2.730 | 42.906 | 1.037 |
| | 2 | AIPW | -0.374 | -0.384 | 1.096 | 0.077 | 1.256 | -0.010 | 0.900 | 4.239 | 0.906 | 4.131 | 0.933 | 3.526 | 1.190 | 58.593 | 1201.004 | 857.954 |
| | 2 | WAIPW | -0.374 | -0.366 | 0.211 | 0.060 | 1.088 | 0.008 | 0.929 | 3.637 | 0.934 | 3.521 | 0.943 | 3.284 | 0.694 | 6.312 | 44.682 | 3.390 |
| WAIPW(0.25) | 2 | IPW | -0.374 | -0.373 | 0.160 | 0.009 | 1.017 | 0.001 | 0.943 | 3.290 | 0.945 | 3.230 | 0.945 | 3.213 | 0.620 | 0.933 | 25.450 | 0.504 |
| | 2 | WIPW | -0.374 | -0.373 | 0.157 | 0.012 | 1.014 | 0.002 | 0.947 | 3.169 | 0.946 | 3.191 | 0.945 | 3.213 | 0.612 | 0.884 | 24.710 | 0.487 |
| | 2 | AIPW | -0.374 | -0.374 | 0.134 | 0.002 | 1.042 | 0.000 | 0.939 | 3.385 | 0.942 | 3.306 | 0.941 | 3.338 | 0.511 | 0.695 | 17.932 | 0.358 |
| | 2 | WAIPW | -0.374 | -0.374 | 0.133 | 0.001 | 1.036 | 0.000 | 0.939 | 3.395 | 0.944 | 3.252 | 0.945 | 3.235 | 0.512 | 0.654 | 17.793 | 0.352 |
| WAIPW(0.5) | 2 | IPW | -0.374 | -0.379 | 0.186 | -0.024 | 1.037 | -0.005 | 0.940 | 3.366 | 0.948 | 3.134 | 0.941 | 3.333 | 0.713 | 1.477 | 34.480 | 0.692 |
| | 2 | WIPW | -0.374 | -0.378 | 0.172 | -0.027 | 1.024 | -0.004 | 0.943 | 3.266 | 0.951 | 3.046 | 0.944 | 3.246 | 0.663 | 1.086 | 29.428 | 0.589 |
| | 2 | AIPW | -0.374 | -0.377 | 0.151 | -0.026 | 1.040 | -0.003 | 0.944 | 3.239 | 0.951 | 3.054 | 0.942 | 3.306 | 0.581 | 1.198 | 22.716 | 0.442 |
| | 2 | WAIPW | -0.374 | -0.376 | 0.142 | -0.019 | 1.029 | -0.002 | 0.943 | 3.273 | 0.951 | 3.039 | 0.944 | 3.252 | 0.551 | 0.846 | 20.247 | 0.392 |
| WAIPW(0.75) | 2 | IPW | -0.374 | -0.377 | 0.237 | -0.019 | 1.106 | -0.003 | 0.923 | 3.780 | 0.931 | 3.575 | 0.929 | 3.637 | 0.868 | 2.958 | 56.320 | 1.159 |
| | 2 | WIPW | -0.374 | -0.376 | 0.188 | -0.015 | 1.049 | -0.002 | 0.937 | 3.447 | 0.942 | 3.301 | 0.938 | 3.401 | 0.714 | 1.423 | 35.443 | 0.726 |
| | 2 | AIPW | -0.374 | -0.379 | 0.192 | -0.043 | 1.092 | -0.005 | 0.928 | 3.656 | 0.940 | 3.359 | 0.929 | 3.632 | 0.716 | 2.617 | 36.938 | 0.764 |
| | 2 | WAIPW | -0.374 | -0.379 | 0.158 | -0.036 | 1.065 | -0.004 | 0.937 | 3.447 | 0.944 | 3.246 | 0.936 | 3.467 | 0.596 | 1.218 | 24.969 | 0.512 |
| WAIPW(1) | 2 | IPW | -0.374 | -0.381 | 0.320 | -0.023 | 1.223 | -0.006 | 0.894 | 4.348 | 0.914 | 3.969 | 0.913 | 3.986 | 1.042 | 6.232 | 102.217 | 2.227 |
| | 2 | WIPW | -0.374 | -0.379 | 0.196 | -0.024 | 1.032 | -0.005 | 0.942 | 3.297 | 0.948 | 3.150 | 0.941 | 3.333 | 0.755 | 2.001 | 38.609 | 0.801 |
| | 2 | AIPW | -0.374 | -0.380 | 0.267 | -0.051 | 1.095 | -0.006 | 0.939 | 3.394 | 0.944 | 3.252 | 0.930 | 3.609 | 0.944 | 6.299 | 71.312 | 1.643 |
| | 2 | WAIPW | -0.374 | -0.378 | 0.168 | -0.027 | 1.046 | -0.003 | 0.939 | 3.372 | 0.946 | 3.183 | 0.940 | 3.368 | 0.639 | 1.861 | 28.120 | 0.583 |
| WIPW(0.25) | 2 | IPW | -0.374 | -0.375 | 0.159 | -0.003 | 1.017 | -0.001 | 0.946 | 3.202 | 0.946 | 3.186 | 0.946 | 3.197 | 0.621 | 0.944 | 25.324 | 0.506 |
| | 2 | WIPW | -0.374 | -0.375 | 0.157 | -0.002 | 1.012 | -0.001 | 0.949 | 3.117 | 0.946 | 3.197 | 0.947 | 3.169 | 0.613 | 0.894 | 24.524 | 0.490 |
| | 2 | AIPW | -0.374 | -0.376 | 0.135 | -0.016 | 1.049 | -0.002 | 0.938 | 3.406 | 0.943 | 3.268 | 0.941 | 3.343 | 0.511 | 0.691 | 18.166 | 0.358 |
| | 2 | WAIPW | -0.374 | -0.376 | 0.135 | -0.014 | 1.047 | -0.001 | 0.939 | 3.380 | 0.942 | 3.306 | 0.938 | 3.416 | 0.512 | 0.655 | 18.164 | 0.358 |
| WIPW(0.5) | 2 | IPW | -0.374 | -0.374 | 0.188 | -0.004 | 1.035 | 0.000 | 0.941 | 3.320 | 0.945 | 3.232 | 0.943 | 3.273 | 0.718 | 1.503 | 35.284 | 0.750 |
| | 2 | WIPW | -0.374 | -0.375 | 0.171 | -0.006 | 1.014 | -0.001 | 0.944 | 3.259 | 0.947 | 3.155 | 0.948 | 3.148 | 0.666 | 1.096 | 29.275 | 0.603 |
| | 2 | AIPW | -0.374 | -0.376 | 0.153 | -0.017 | 1.053 | -0.002 | 0.936 | 3.468 | 0.943 | 3.286 | 0.945 | 3.232 | 0.583 | 1.177 | 23.402 | 0.486 |
| | 2 | WAIPW | -0.374 | -0.376 | 0.144 | -0.014 | 1.036 | -0.002 | 0.940 | 3.346 | 0.945 | 3.232 | 0.943 | 3.273 | 0.552 | 0.833 | 20.671 | 0.423 |
| WIPW(0.75) | 2 | IPW | -0.374 | -0.371 | 0.241 | 0.014 | 1.122 | 0.003 | 0.919 | 3.863 | 0.924 | 3.753 | 0.932 | 3.556 | 0.868 | 2.984 | 58.139 | 1.172 |
| | 2 | WIPW | -0.374 | -0.373 | 0.187 | 0.010 | 1.042 | 0.002 | 0.941 | 3.343 | 0.938 | 3.416 | 0.944 | 3.263 | 0.715 | 1.449 | 35.009 | 0.704 |
| | 2 | AIPW | -0.374 | -0.375 | 0.193 | -0.017 | 1.082 | -0.001 | 0.929 | 3.642 | 0.938 | 3.416 | 0.931 | 3.575 | 0.718 | 2.690 | 37.373 | 0.761 |
| | 2 | WAIPW | -0.374 | -0.375 | 0.158 | -0.008 | 1.057 | -0.001 | 0.933 | 3.541 | 0.942 | 3.311 | 0.938 | 3.416 | 0.598 | 1.253 | 25.059 | 0.502 |
| WIPW(1) | 2 | IPW | -0.374 | -0.370 | 0.320 | 0.018 | 1.223 | 0.004 | 0.899 | 4.256 | 0.915 | 3.948 | 0.915 | 3.934 | 1.044 | 6.198 | 102.233 | 2.169 |
| | 2 | WIPW | -0.374 | -0.368 | 0.197 | 0.031 | 1.039 | 0.006 | 0.941 | 3.342 | 0.943 | 3.288 | 0.946 | 3.192 | 0.756 | 1.958 | 38.772 | 0.764 |
| | 2 | AIPW | -0.374 | -0.372 | 0.268 | -0.008 | 1.107 | 0.002 | 0.937 | 3.437 | 0.941 | 3.337 | 0.930 | 3.597 | 0.942 | 6.404 | 71.885 | 1.643 |

| Randomization Method | Regime | Estimator | True Value | Mean Estimate | SD Estimates | Mean Normalized | SD Normalized | Mean Bias | CI Coverage | SE CI Coverage x 10^3 | LB Coverage | SE LB Coverage x 10^3 | UB Coverage | SE UB Coverage x 10^3 | Mean CI Length | SE CI Length x 10^3 | MSE x 10^3 | SE MSE x 10^3 |
|---|---|---|---|---|---|---|---|---|---|---|---|---|---|---|---|---|---|---|
| | 2 | WAIPW | -0.374 | -0.371 | 0.167 | 0.019 | 1.046 | 0.003 | 0.942 | 3.315 | 0.943 | 3.270 | 0.943 | 3.275 | 0.638 | 1.902 | 27.803 | 0.532 |
| AIPW(0.25) | 3 | IPW | -0.500 | -0.500 | 0.161 | 0.001 | 1.008 | 0.000 | 0.943 | 3.274 | 0.947 | 3.174 | 0.949 | 3.117 | 0.632 | 0.940 | 25.915 | 0.536 |
| | 3 | WIPW | -0.500 | -0.499 | 0.242 | 0.001 | 1.060 | 0.000 | 0.936 | 3.462 | 0.941 | 3.327 | 0.942 | 3.295 | 0.920 | 2.318 | 58.553 | 1.201 |
| | 3 | AIPW | -0.500 | -0.498 | 0.132 | 0.010 | 1.019 | 0.001 | 0.944 | 3.257 | 0.941 | 3.322 | 0.949 | 3.117 | 0.516 | 0.690 | 17.466 | 0.353 |
| | 3 | WAIPW | -0.500 | -0.499 | 0.132 | 0.005 | 1.015 | 0.001 | 0.945 | 3.224 | 0.944 | 3.246 | 0.950 | 3.088 | 0.516 | 0.651 | 17.311 | 0.351 |
| AIPW(0.5) | 3 | IPW | -0.500 | -0.500 | 0.193 | -0.001 | 1.055 | 0.000 | 0.937 | 3.443 | 0.942 | 3.313 | 0.939 | 3.385 | 0.728 | 1.540 | 37.214 | 0.749 |
| | 3 | WIPW | -0.500 | -0.499 | 0.312 | 0.000 | 1.171 | 0.000 | 0.909 | 4.068 | 0.927 | 3.668 | 0.926 | 3.691 | 1.113 | 3.871 | 97.017 | 1.969 |
| | 3 | AIPW | -0.500 | -0.503 | 0.154 | -0.018 | 1.044 | -0.003 | 0.947 | 3.176 | 0.946 | 3.204 | 0.943 | 3.280 | 0.587 | 1.191 | 23.659 | 0.461 |
| | 3 | WAIPW | -0.500 | -0.502 | 0.144 | -0.017 | 1.028 | -0.003 | 0.946 | 3.190 | 0.948 | 3.141 | 0.945 | 3.218 | 0.557 | 0.855 | 20.749 | 0.399 |
| AIPW(0.75) | 3 | IPW | -0.500 | -0.501 | 0.243 | -0.009 | 1.100 | -0.002 | 0.923 | 3.766 | 0.935 | 3.492 | 0.930 | 3.618 | 0.890 | 3.062 | 58.930 | 1.212 |
| | 3 | WIPW | -0.500 | -0.500 | 0.393 | -0.009 | 1.300 | 0.000 | 0.871 | 4.744 | 0.904 | 4.174 | 0.900 | 4.247 | 1.311 | 6.596 | 154.495 | 3.243 |
| | 3 | AIPW | -0.500 | -0.502 | 0.192 | -0.011 | 1.065 | -0.003 | 0.930 | 3.604 | 0.943 | 3.290 | 0.938 | 3.401 | 0.727 | 2.642 | 36.964 | 0.766 |
| | 3 | WAIPW | -0.500 | -0.503 | 0.157 | -0.022 | 1.046 | -0.004 | 0.935 | 3.487 | 0.948 | 3.129 | 0.939 | 3.375 | 0.603 | 1.229 | 24.802 | 0.501 |
| AIPW(1) | 3 | IPW | -0.500 | -0.496 | 0.329 | 0.010 | 1.248 | 0.003 | 0.892 | 4.396 | 0.907 | 4.101 | 0.912 | 4.010 | 1.045 | 6.202 | 108.087 | 2.267 |
| | 3 | WIPW | -0.500 | -0.493 | 0.474 | 0.018 | 1.503 | 0.007 | 0.820 | 5.436 | 0.863 | 4.863 | 0.871 | 4.739 | 1.285 | 9.771 | 224.265 | 4.863 |
| | 3 | AIPW | -0.500 | -0.499 | 0.269 | 0.025 | 1.119 | 0.000 | 0.932 | 3.557 | 0.928 | 3.652 | 0.939 | 3.394 | 0.938 | 6.247 | 72.288 | 1.647 |
| | 3 | WAIPW | -0.500 | -0.500 | 0.171 | 0.000 | 1.061 | -0.001 | 0.938 | 3.398 | 0.941 | 3.342 | 0.941 | 3.328 | 0.640 | 1.841 | 29.179 | 0.614 |
| AR-1 | 3 | IPW | -0.500 | -0.512 | 0.363 | -0.052 | 1.292 | -0.013 | 0.880 | 4.599 | 0.907 | 4.100 | 0.893 | 4.376 | 1.071 | 7.029 | 132.121 | 3.411 |
| | 3 | WIPW | -0.500 | -0.505 | 0.233 | -0.029 | 1.089 | -0.005 | 0.929 | 3.642 | 0.937 | 3.436 | 0.931 | 3.575 | 0.839 | 3.083 | 54.345 | 1.211 |
| | 3 | AIPW | -0.500 | -0.500 | 0.315 | -0.038 | 1.128 | 0.000 | 0.933 | 3.526 | 0.943 | 3.274 | 0.932 | 3.561 | 1.016 | 9.212 | 99.365 | 2.998 |
| | 3 | WAIPW | -0.500 | -0.502 | 0.218 | -0.027 | 1.074 | -0.002 | 0.944 | 3.252 | 0.945 | 3.219 | 0.939 | 3.395 | 0.774 | 4.731 | 47.557 | 1.199 |
| AR-2 | 3 | IPW | -0.500 | -0.511 | 0.428 | -0.096 | 1.447 | -0.011 | 0.833 | 5.270 | 0.889 | 4.436 | 0.866 | 4.821 | 1.064 | 7.601 | 183.651 | 5.804 |
| | 3 | WIPW | -0.500 | -0.507 | 0.275 | -0.077 | 1.152 | -0.007 | 0.911 | 4.019 | 0.936 | 3.457 | 0.913 | 3.986 | 0.894 | 4.526 | 75.502 | 2.007 |
| | 3 | AIPW | -0.500 | -0.499 | 0.457 | -0.071 | 1.185 | 0.000 | 0.916 | 3.915 | 0.937 | 3.447 | 0.915 | 3.953 | 1.179 | 18.655 | 208.976 | 11.741 |
| | 3 | WAIPW | -0.500 | -0.502 | 0.287 | -0.055 | 1.119 | -0.003 | 0.926 | 3.702 | 0.942 | 3.311 | 0.924 | 3.743 | 0.862 | 9.841 | 82.592 | 4.191 |
| IAIPW(0.25) | 3 | IPW | -0.500 | -0.499 | 0.163 | 0.007 | 1.024 | 0.001 | 0.945 | 3.219 | 0.944 | 3.257 | 0.953 | 3.005 | 0.634 | 0.950 | 26.530 | 0.552 |
| | 3 | WIPW | -0.500 | -0.499 | 0.161 | 0.007 | 1.023 | 0.001 | 0.946 | 3.208 | 0.945 | 3.224 | 0.954 | 2.975 | 0.626 | 0.896 | 25.838 | 0.536 |
| | 3 | AIPW | -0.500 | -0.499 | 0.134 | 0.009 | 1.029 | 0.001 | 0.941 | 3.327 | 0.944 | 3.257 | 0.947 | 3.180 | 0.516 | 0.698 | 17.877 | 0.368 |
| | 3 | WAIPW | -0.500 | -0.498 | 0.133 | 0.013 | 1.026 | 0.001 | 0.941 | 3.338 | 0.943 | 3.284 | 0.948 | 3.135 | 0.516 | 0.660 | 17.796 | 0.363 |
| IAIPW(0.5) | 3 | IPW | -0.500 | -0.499 | 0.190 | 0.001 | 1.042 | 0.001 | 0.941 | 3.333 | 0.945 | 3.225 | 0.943 | 3.280 | 0.726 | 1.510 | 36.077 | 0.732 |
| | 3 | WIPW | -0.500 | -0.498 | 0.175 | 0.007 | 1.021 | 0.001 | 0.946 | 3.183 | 0.950 | 3.090 | 0.946 | 3.183 | 0.675 | 1.108 | 30.445 | 0.623 |
| | 3 | AIPW | -0.500 | -0.500 | 0.152 | -0.004 | 1.037 | -0.001 | 0.939 | 3.372 | 0.947 | 3.162 | 0.942 | 3.293 | 0.589 | 1.199 | 23.236 | 0.459 |
| | 3 | WAIPW | -0.500 | -0.500 | 0.144 | 0.000 | 1.026 | 0.000 | 0.943 | 3.273 | 0.951 | 3.046 | 0.943 | 3.286 | 0.558 | 0.853 | 20.648 | 0.414 |
| IAIPW(0.75) | 3 | IPW | -0.500 | -0.498 | 0.241 | 0.011 | 1.094 | 0.001 | 0.929 | 3.637 | 0.929 | 3.637 | 0.937 | 3.436 | 0.884 | 3.080 | 58.058 | 1.222 |
| | 3 | WIPW | -0.500 | -0.499 | 0.191 | 0.004 | 1.037 | 0.000 | 0.939 | 3.390 | 0.940 | 3.348 | 0.948 | 3.140 | 0.728 | 1.499 | 36.298 | 0.757 |
| | 3 | AIPW | -0.500 | -0.498 | 0.195 | 0.023 | 1.071 | 0.001 | 0.936 | 3.452 | 0.934 | 3.512 | 0.948 | 3.152 | 0.728 | 2.666 | 38.115 | 0.797 |
| | 3 | WAIPW | -0.500 | -0.499 | 0.159 | 0.011 | 1.046 | 0.001 | 0.942 | 3.301 | 0.938 | 3.421 | 0.947 | 3.174 | 0.604 | 1.242 | 25.215 | 0.519 |
| IAIPW(1) | 3 | IPW | -0.500 | -0.491 | 0.330 | 0.035 | 1.246 | 0.008 | 0.892 | 4.390 | 0.907 | 4.115 | 0.914 | 3.969 | 1.058 | 6.178 | 108.877 | 2.267 |
| | 3 | WIPW | -0.500 | -0.493 | 0.205 | 0.034 | 1.065 | 0.007 | 0.935 | 3.491 | 0.936 | 3.470 | 0.943 | 3.270 | 0.767 | 1.935 | 42.246 | 0.852 |
| | 3 | AIPW | -0.500 | -0.498 | 0.269 | 0.034 | 1.116 | 0.002 | 0.930 | 3.613 | 0.927 | 3.676 | 0.948 | 3.140 | 0.957 | 6.252 | 72.299 | 1.588 |
| | 3 | WAIPW | -0.500 | -0.497 | 0.168 | 0.020 | 1.049 | 0.002 | 0.936 | 3.458 | 0.937 | 3.428 | 0.948 | 3.150 | 0.644 | 1.833 | 28.382 | 0.569 |
| IPW(0.25) | 3 | IPW | -0.500 | -0.497 | 0.162 | 0.018 | 1.016 | 0.003 | 0.944 | 3.241 | 0.948 | 3.146 | 0.948 | 3.135 | 0.634 | 0.972 | 26.239 | 0.535 |
| | 3 | WIPW | -0.500 | -0.500 | 0.245 | 0.004 | 1.079 | 0.000 | 0.933 | 3.546 | 0.934 | 3.517 | 0.939 | 3.395 | 0.926 | 2.378 | 59.944 | 1.255 |
| | 3 | AIPW | -0.500 | -0.499 | 0.132 | 0.004 | 1.016 | 0.000 | 0.944 | 3.246 | 0.951 | 3.065 | 0.948 | 3.140 | 0.518 | 0.683 | 17.509 | 0.345 |
| | 3 | WAIPW | -0.500 | -0.499 | 0.131 | 0.006 | 1.009 | 0.001 | 0.946 | 3.186 | 0.951 | 3.041 | 0.948 | 3.140 | 0.518 | 0.644 | 17.273 | 0.339 |

| Randomization Method | Regime | Estimator | True Value | Mean Estimate | SD Estimates | Mean Normalized | SD Normalized | Mean Bias | CI Coverage | SE CI Coverage x 10^3 | LB Coverage | SE LB Coverage x 10^3 | UB Coverage | SE UB Coverage x 10^3 | Mean CI Length | SE CI Length x 10^3 | MSE x 10^3 | SE MSE x 10^3 |
|---|---|---|---|---|---|---|---|---|---|---|---|---|---|---|---|---|---|---|
| IPW(0.5) | 3 | IPW | -0.500 | -0.497 | 0.191 | 0.011 | 1.040 | 0.002 | 0.942 | 3.300 | 0.940 | 3.366 | 0.944 | 3.252 | 0.732 | 1.553 | 36.345 | 0.705 |
| | 3 | WIPW | -0.500 | -0.497 | 0.306 | 0.010 | 1.143 | 0.003 | 0.915 | 3.950 | 0.927 | 3.680 | 0.928 | 3.645 | 1.121 | 3.917 | 93.407 | 1.868 |
| | 3 | AIPW | -0.500 | -0.500 | 0.155 | 0.002 | 1.045 | 0.000 | 0.939 | 3.385 | 0.946 | 3.183 | 0.941 | 3.320 | 0.593 | 1.219 | 23.999 | 0.483 |
| | 3 | WAIPW | -0.500 | -0.500 | 0.145 | -0.002 | 1.033 | -0.001 | 0.941 | 3.320 | 0.942 | 3.293 | 0.945 | 3.218 | 0.560 | 0.863 | 21.116 | 0.422 |
| IPW(0.75) | 3 | IPW | -0.500 | -0.496 | 0.242 | 0.015 | 1.106 | 0.003 | 0.924 | 3.743 | 0.930 | 3.613 | 0.934 | 3.512 | 0.892 | 3.131 | 58.577 | 1.211 |
| | 3 | WIPW | -0.500 | -0.496 | 0.388 | 0.012 | 1.315 | 0.003 | 0.874 | 4.700 | 0.901 | 4.228 | 0.906 | 4.127 | 1.313 | 6.797 | 150.892 | 3.127 |
| | 3 | AIPW | -0.500 | -0.497 | 0.193 | 0.021 | 1.057 | 0.003 | 0.936 | 3.472 | 0.934 | 3.507 | 0.948 | 3.152 | 0.732 | 2.692 | 37.261 | 0.778 |
| | 3 | WAIPW | -0.500 | -0.496 | 0.157 | 0.022 | 1.033 | 0.003 | 0.942 | 3.295 | 0.941 | 3.327 | 0.948 | 3.135 | 0.605 | 1.244 | 24.566 | 0.501 |
| IPW(1) | 3 | IPW | -0.500 | -0.504 | 0.327 | -0.010 | 1.223 | -0.005 | 0.897 | 4.293 | 0.912 | 4.004 | 0.913 | 3.979 | 1.058 | 6.165 | 107.101 | 2.278 |
| | 3 | WIPW | -0.500 | -0.506 | 0.473 | -0.010 | 1.481 | -0.007 | 0.828 | 5.332 | 0.873 | 4.704 | 0.869 | 4.770 | 1.310 | 9.754 | 223.774 | 4.874 |
| | 3 | AIPW | -0.500 | -0.506 | 0.265 | 0.003 | 1.117 | -0.006 | 0.934 | 3.504 | 0.930 | 3.601 | 0.943 | 3.279 | 0.942 | 6.224 | 70.061 | 1.558 |
| | 3 | WAIPW | -0.500 | -0.503 | 0.170 | -0.013 | 1.057 | -0.003 | 0.933 | 3.545 | 0.944 | 3.261 | 0.938 | 3.398 | 0.641 | 1.816 | 28.766 | 0.586 |
| SR | 3 | IPW | -0.500 | -0.499 | 0.153 | 0.007 | 1.036 | 0.001 | 0.942 | 3.317 | 0.943 | 3.274 | 0.946 | 3.197 | 0.585 | 0.765 | 23.482 | 0.465 |
| | 3 | WIPW | -0.500 | -0.501 | 0.209 | -0.003 | 1.047 | -0.001 | 0.940 | 3.364 | 0.942 | 3.317 | 0.945 | 3.236 | 0.798 | 1.650 | 43.670 | 0.876 |
| | 3 | AIPW | -0.500 | -0.497 | 0.125 | 0.021 | 1.028 | 0.002 | 0.943 | 3.269 | 0.943 | 3.285 | 0.949 | 3.112 | 0.482 | 0.546 | 15.664 | 0.310 |
| | 3 | WAIPW | -0.500 | -0.497 | 0.127 | 0.019 | 1.029 | 0.002 | 0.944 | 3.258 | 0.943 | 3.290 | 0.949 | 3.100 | 0.487 | 0.552 | 16.014 | 0.315 |
| TS(0.25) | 3 | IPW | -0.500 | -0.490 | 0.342 | 0.033 | 1.265 | 0.009 | 0.887 | 4.474 | 0.896 | 4.317 | 0.906 | 4.131 | 0.842 | 7.149 | 117.235 | 4.060 |
| | 3 | WIPW | -0.500 | -0.498 | 0.179 | -0.002 | 1.023 | 0.001 | 0.949 | 3.123 | 0.946 | 3.197 | 0.948 | 3.129 | 0.688 | 1.805 | 32.184 | 0.701 |
| | 3 | AIPW | -0.500 | -0.490 | 0.349 | 0.030 | 1.138 | 0.009 | 0.925 | 3.721 | 0.932 | 3.556 | 0.941 | 3.338 | 0.953 | 13.887 | 121.803 | 4.895 |
| | 3 | WAIPW | -0.500 | -0.497 | 0.160 | 0.000 | 1.045 | 0.002 | 0.937 | 3.441 | 0.941 | 3.338 | 0.946 | 3.191 | 0.594 | 2.596 | 25.723 | 0.606 |
| TS(0.50) | 3 | IPW | -0.500 | -0.500 | 0.342 | 0.002 | 1.277 | 0.000 | 0.888 | 4.460 | 0.907 | 4.104 | 0.899 | 4.269 | 0.878 | 7.068 | 117.081 | 3.698 |
| | 3 | WIPW | -0.500 | -0.507 | 0.184 | -0.048 | 1.039 | -0.007 | 0.942 | 3.306 | 0.946 | 3.208 | 0.937 | 3.431 | 0.698 | 1.641 | 34.040 | 0.699 |
| | 3 | AIPW | -0.500 | -0.497 | 0.339 | 0.015 | 1.139 | 0.002 | 0.930 | 3.599 | 0.934 | 3.512 | 0.941 | 3.343 | 0.953 | 12.798 | 115.090 | 4.564 |
| | 3 | WAIPW | -0.500 | -0.504 | 0.160 | -0.040 | 1.051 | -0.005 | 0.938 | 3.406 | 0.947 | 3.163 | 0.939 | 3.395 | 0.593 | 2.144 | 25.507 | 0.576 |
| TS(0.75) | 3 | IPW | -0.500 | -0.495 | 0.352 | 0.025 | 1.271 | 0.005 | 0.887 | 4.474 | 0.898 | 4.277 | 0.916 | 3.932 | 0.929 | 7.521 | 123.604 | 3.875 |
| | 3 | WIPW | -0.500 | -0.498 | 0.187 | 0.001 | 1.032 | 0.002 | 0.944 | 3.252 | 0.944 | 3.246 | 0.948 | 3.140 | 0.715 | 1.616 | 34.909 | 0.751 |
| | 3 | AIPW | -0.500 | -0.494 | 0.341 | 0.041 | 1.125 | 0.005 | 0.933 | 3.536 | 0.934 | 3.512 | 0.950 | 3.094 | 1.002 | 13.266 | 116.212 | 4.423 |
| | 3 | WAIPW | -0.500 | -0.497 | 0.159 | 0.009 | 1.036 | 0.003 | 0.943 | 3.284 | 0.942 | 3.306 | 0.949 | 3.106 | 0.604 | 2.256 | 25.342 | 0.621 |
| TS(1) | 3 | IPW | -0.500 | -0.486 | 0.358 | 0.059 | 1.297 | 0.014 | 0.875 | 4.684 | 0.891 | 4.401 | 0.906 | 4.124 | 0.953 | 7.445 | 128.033 | 3.891 |
| | 3 | WIPW | -0.500 | -0.497 | 0.192 | 0.013 | 1.048 | 0.003 | 0.936 | 3.462 | 0.939 | 3.385 | 0.945 | 3.224 | 0.724 | 1.522 | 36.830 | 0.805 |
| | 3 | AIPW | -0.500 | -0.486 | 0.361 | 0.049 | 1.139 | 0.013 | 0.932 | 3.556 | 0.929 | 3.632 | 0.945 | 3.219 | 1.026 | 13.679 | 130.553 | 8.061 |
| | 3 | WAIPW | -0.500 | -0.497 | 0.163 | 0.014 | 1.061 | 0.002 | 0.936 | 3.452 | 0.938 | 3.421 | 0.946 | 3.191 | 0.607 | 1.899 | 26.649 | 0.663 |
| WAIPW(0.25) | 3 | IPW | -0.500 | -0.500 | 0.163 | -0.002 | 1.020 | 0.000 | 0.945 | 3.219 | 0.950 | 3.071 | 0.944 | 3.263 | 0.633 | 0.946 | 26.669 | 0.547 |
| | 3 | WIPW | -0.500 | -0.499 | 0.161 | 0.000 | 1.019 | 0.000 | 0.945 | 3.230 | 0.952 | 3.029 | 0.945 | 3.224 | 0.625 | 0.896 | 25.991 | 0.529 |
| | 3 | AIPW | -0.500 | -0.500 | 0.134 | -0.003 | 1.026 | -0.001 | 0.945 | 3.235 | 0.945 | 3.213 | 0.945 | 3.235 | 0.518 | 0.689 | 17.858 | 0.356 |
| | 3 | WAIPW | -0.500 | -0.500 | 0.133 | -0.005 | 1.023 | -0.001 | 0.945 | 3.230 | 0.946 | 3.202 | 0.946 | 3.208 | 0.518 | 0.649 | 17.745 | 0.352 |
| WAIPW(0.5) | 3 | IPW | -0.500 | -0.499 | 0.189 | 0.003 | 1.030 | 0.000 | 0.943 | 3.286 | 0.949 | 3.112 | 0.944 | 3.259 | 0.730 | 1.570 | 35.534 | 0.727 |
| | 3 | WIPW | -0.500 | -0.500 | 0.173 | 0.000 | 1.012 | 0.000 | 0.944 | 3.252 | 0.950 | 3.068 | 0.944 | 3.239 | 0.679 | 1.147 | 29.919 | 0.619 |
| | 3 | AIPW | -0.500 | -0.501 | 0.155 | -0.005 | 1.052 | -0.001 | 0.941 | 3.333 | 0.941 | 3.320 | 0.940 | 3.353 | 0.590 | 1.218 | 23.907 | 0.484 |
| | 3 | WAIPW | -0.500 | -0.501 | 0.146 | -0.009 | 1.041 | -0.001 | 0.939 | 3.379 | 0.945 | 3.225 | 0.938 | 3.398 | 0.559 | 0.861 | 21.228 | 0.434 |
| WAIPW(0.75) | 3 | IPW | -0.500 | -0.504 | 0.241 | -0.023 | 1.094 | -0.004 | 0.924 | 3.739 | 0.939 | 3.375 | 0.929 | 3.637 | 0.889 | 3.077 | 58.090 | 1.170 |
| | 3 | WIPW | -0.500 | -0.502 | 0.191 | -0.014 | 1.039 | -0.003 | 0.937 | 3.431 | 0.942 | 3.317 | 0.943 | 3.268 | 0.731 | 1.496 | 36.438 | 0.724 |
| | 3 | AIPW | -0.500 | -0.504 | 0.191 | -0.018 | 1.059 | -0.004 | 0.936 | 3.462 | 0.940 | 3.354 | 0.941 | 3.322 | 0.728 | 2.624 | 36.596 | 0.714 |
| | 3 | WAIPW | -0.500 | -0.503 | 0.156 | -0.023 | 1.034 | -0.004 | 0.939 | 3.390 | 0.942 | 3.306 | 0.942 | 3.306 | 0.604 | 1.217 | 24.491 | 0.486 |
| WAIPW(1) | 3 | IPW | -0.500 | -0.504 | 0.332 | 0.004 | 1.244 | -0.005 | 0.886 | 4.501 | 0.900 | 4.246 | 0.917 | 3.899 | 1.058 | 6.222 | 109.954 | 2.404 |

| Randomization Method | Regime | Estimator | True Value | Mean Estimate | SD Estimates | Mean Normalized | SD Normalized | Mean Bias | CI Coverage | SE CI Coverage x 10^3 | LB Coverage | SE LB Coverage x 10^3 | UB Coverage | SE UB Coverage x 10^3 | Mean CI Length | SE CI Length x 10^3 | MSE x 10^3 | SE MSE x 10^3 |
|---|---|---|---|---|---|---|---|---|---|---|---|---|---|---|---|---|---|---|
| | 3 | WIPW | -0.500 | -0.500 | 0.207 | 0.000 | 1.064 | -0.001 | 0.933 | 3.540 | 0.937 | 3.441 | 0.945 | 3.215 | 0.769 | 1.988 | 42.782 | 0.865 |
| | 3 | AIPW | -0.500 | -0.503 | 0.272 | 0.016 | 1.105 | -0.003 | 0.932 | 3.553 | 0.933 | 3.536 | 0.944 | 3.248 | 0.954 | 6.380 | 74.072 | 1.797 |
| | 3 | WAIPW | -0.500 | -0.501 | 0.171 | -0.003 | 1.054 | -0.001 | 0.938 | 3.420 | 0.937 | 3.437 | 0.943 | 3.266 | 0.645 | 1.868 | 29.263 | 0.615 |
| WIPW(0.25) | 3 | IPW | -0.500 | -0.500 | 0.166 | -0.001 | 1.036 | 0.000 | 0.942 | 3.301 | 0.943 | 3.268 | 0.946 | 3.197 | 0.635 | 0.960 | 27.513 | 0.566 |
| | 3 | WIPW | -0.500 | -0.499 | 0.163 | 0.001 | 1.033 | 0.000 | 0.941 | 3.322 | 0.944 | 3.252 | 0.946 | 3.191 | 0.626 | 0.906 | 26.689 | 0.548 |
| | 3 | AIPW | -0.500 | -0.499 | 0.135 | 0.003 | 1.033 | 0.000 | 0.939 | 3.395 | 0.943 | 3.284 | 0.945 | 3.219 | 0.518 | 0.686 | 18.169 | 0.366 |
| | 3 | WAIPW | -0.500 | -0.499 | 0.135 | 0.002 | 1.032 | 0.000 | 0.940 | 3.348 | 0.945 | 3.213 | 0.944 | 3.263 | 0.518 | 0.650 | 18.103 | 0.365 |
| WIPW(0.5) | 3 | IPW | -0.500 | -0.503 | 0.194 | -0.025 | 1.053 | -0.003 | 0.934 | 3.512 | 0.945 | 3.232 | 0.941 | 3.320 | 0.729 | 1.517 | 37.788 | 0.770 |
| | 3 | WIPW | -0.500 | -0.504 | 0.179 | -0.029 | 1.040 | -0.004 | 0.939 | 3.385 | 0.947 | 3.176 | 0.944 | 3.252 | 0.678 | 1.106 | 31.973 | 0.637 |
| | 3 | AIPW | -0.500 | -0.501 | 0.156 | -0.011 | 1.052 | -0.001 | 0.936 | 3.462 | 0.941 | 3.326 | 0.946 | 3.204 | 0.590 | 1.197 | 24.266 | 0.491 |
| | 3 | WAIPW | -0.500 | -0.501 | 0.146 | -0.014 | 1.039 | -0.002 | 0.937 | 3.443 | 0.945 | 3.225 | 0.944 | 3.246 | 0.558 | 0.845 | 21.320 | 0.429 |
| WIPW(0.75) | 3 | IPW | -0.500 | -0.496 | 0.242 | 0.017 | 1.101 | 0.004 | 0.927 | 3.670 | 0.930 | 3.618 | 0.938 | 3.401 | 0.889 | 3.041 | 58.478 | 1.172 |
| | 3 | WIPW | -0.500 | -0.498 | 0.189 | 0.010 | 1.033 | 0.002 | 0.943 | 3.279 | 0.945 | 3.230 | 0.944 | 3.241 | 0.731 | 1.467 | 35.887 | 0.727 |
| | 3 | AIPW | -0.500 | -0.498 | 0.194 | 0.017 | 1.059 | 0.002 | 0.943 | 3.290 | 0.936 | 3.467 | 0.945 | 3.219 | 0.731 | 2.680 | 37.766 | 0.779 |
| | 3 | WAIPW | -0.500 | -0.499 | 0.159 | 0.007 | 1.041 | 0.001 | 0.936 | 3.452 | 0.940 | 3.369 | 0.946 | 3.202 | 0.606 | 1.239 | 25.210 | 0.503 |
| WIPW(1) | 3 | IPW | -0.500 | -0.500 | 0.332 | -0.010 | 1.231 | -0.001 | 0.894 | 4.354 | 0.915 | 3.941 | 0.910 | 4.044 | 1.075 | 6.424 | 110.426 | 2.403 |
| | 3 | WIPW | -0.500 | -0.497 | 0.204 | 0.011 | 1.057 | 0.002 | 0.935 | 3.474 | 0.939 | 3.377 | 0.941 | 3.333 | 0.773 | 2.019 | 41.649 | 0.858 |
| | 3 | AIPW | -0.500 | -0.501 | 0.269 | 0.010 | 1.100 | -0.001 | 0.940 | 3.350 | 0.936 | 3.462 | 0.943 | 3.288 | 0.956 | 6.364 | 72.594 | 1.621 |
| | 3 | WAIPW | -0.500 | -0.499 | 0.169 | 0.004 | 1.048 | 0.001 | 0.941 | 3.328 | 0.945 | 3.234 | 0.945 | 3.220 | 0.645 | 1.883 | 28.669 | 0.577 |
| AIPW(0.25) | 4 | IPW | -0.251 | -0.253 | 0.173 | -0.014 | 1.023 | -0.002 | 0.943 | 3.290 | 0.946 | 3.202 | 0.948 | 3.146 | 0.671 | 1.035 | 29.925 | 0.605 |
| | 4 | WIPW | -0.251 | -0.253 | 0.262 | -0.014 | 1.095 | -0.002 | 0.926 | 3.712 | 0.937 | 3.436 | 0.936 | 3.457 | 0.978 | 2.530 | 68.795 | 1.400 |
| | 4 | AIPW | -0.251 | -0.250 | 0.140 | 0.011 | 1.034 | 0.001 | 0.938 | 3.406 | 0.942 | 3.311 | 0.949 | 3.117 | 0.538 | 0.683 | 19.496 | 0.391 |
| | 4 | WAIPW | -0.251 | -0.250 | 0.139 | 0.008 | 1.027 | 0.001 | 0.945 | 3.224 | 0.943 | 3.279 | 0.947 | 3.174 | 0.536 | 0.643 | 19.214 | 0.386 |
| AIPW(0.5) | 4 | IPW | -0.251 | -0.251 | 0.202 | 0.005 | 1.035 | 0.000 | 0.937 | 3.443 | 0.942 | 3.306 | 0.947 | 3.169 | 0.775 | 1.637 | 40.638 | 0.827 |
| | 4 | WIPW | -0.251 | -0.251 | 0.326 | 0.002 | 1.149 | 0.000 | 0.912 | 4.007 | 0.926 | 3.691 | 0.927 | 3.680 | 1.187 | 4.092 | 106.456 | 2.134 |
| | 4 | AIPW | -0.251 | -0.250 | 0.160 | 0.009 | 1.036 | 0.001 | 0.936 | 3.462 | 0.941 | 3.326 | 0.946 | 3.190 | 0.615 | 1.206 | 25.577 | 0.525 |
| | 4 | WAIPW | -0.251 | -0.249 | 0.150 | 0.016 | 1.024 | 0.002 | 0.942 | 3.293 | 0.943 | 3.286 | 0.950 | 3.076 | 0.581 | 0.857 | 22.431 | 0.458 |
| AIPW(0.75) | 4 | IPW | -0.251 | -0.247 | 0.256 | 0.015 | 1.109 | 0.004 | 0.924 | 3.753 | 0.927 | 3.679 | 0.934 | 3.507 | 0.940 | 3.184 | 65.637 | 1.320 |
| | 4 | WIPW | -0.251 | -0.249 | 0.418 | 0.011 | 1.330 | 0.002 | 0.864 | 4.845 | 0.893 | 4.372 | 0.897 | 4.295 | 1.379 | 6.864 | 174.411 | 3.540 |
| | 4 | AIPW | -0.251 | -0.250 | 0.199 | 0.015 | 1.058 | 0.001 | 0.940 | 3.348 | 0.938 | 3.401 | 0.948 | 3.146 | 0.756 | 2.610 | 39.525 | 0.815 |
| | 4 | WAIPW | -0.251 | -0.250 | 0.161 | 0.010 | 1.029 | 0.001 | 0.944 | 3.241 | 0.944 | 3.241 | 0.951 | 3.065 | 0.627 | 1.211 | 25.959 | 0.519 |
| AIPW(1) | 4 | IPW | -0.251 | -0.247 | 0.350 | 0.019 | 1.237 | 0.004 | 0.895 | 4.336 | 0.909 | 4.065 | 0.911 | 4.034 | 1.122 | 6.502 | 122.638 | 2.557 |
| | 4 | WIPW | -0.251 | -0.243 | 0.506 | 0.024 | 1.494 | 0.008 | 0.826 | 5.356 | 0.870 | 4.749 | 0.871 | 4.741 | 1.389 | 10.278 | 256.046 | 5.391 |
| | 4 | AIPW | -0.251 | -0.249 | 0.286 | 0.045 | 1.119 | 0.002 | 0.929 | 3.640 | 0.926 | 3.699 | 0.940 | 3.363 | 0.995 | 6.291 | 81.794 | 1.842 |
| | 4 | WAIPW | -0.251 | -0.250 | 0.178 | 0.016 | 1.057 | 0.001 | 0.934 | 3.499 | 0.938 | 3.411 | 0.946 | 3.197 | 0.670 | 1.853 | 31.579 | 0.663 |
| AR-1 | 4 | IPW | -0.251 | -0.247 | 0.320 | 0.089 | 1.214 | 0.004 | 0.902 | 4.213 | 0.907 | 4.116 | 0.927 | 3.684 | 1.044 | 5.705 | 102.590 | 2.404 |
| | 4 | WIPW | -0.251 | -0.248 | 0.209 | 0.038 | 1.055 | 0.003 | 0.940 | 3.354 | 0.938 | 3.411 | 0.952 | 3.023 | 0.783 | 1.851 | 43.668 | 0.916 |
| | 4 | AIPW | -0.251 | -0.250 | 0.264 | 0.005 | 1.124 | 0.001 | 0.928 | 3.651 | 0.934 | 3.517 | 0.934 | 3.517 | 0.912 | 6.068 | 69.511 | 1.660 |
| | 4 | WAIPW | -0.251 | -0.248 | 0.176 | 0.036 | 1.066 | 0.003 | 0.936 | 3.462 | 0.938 | 3.401 | 0.947 | 3.180 | 0.657 | 2.044 | 30.973 | 0.660 |
| AR-2 | 4 | IPW | -0.251 | -0.230 | 0.340 | 0.175 | 1.231 | 0.021 | 0.898 | 4.273 | 0.893 | 4.379 | 0.932 | 3.570 | 1.034 | 5.877 | 116.031 | 3.413 |
| | 4 | WIPW | -0.251 | -0.250 | 0.213 | 0.034 | 1.045 | 0.001 | 0.941 | 3.327 | 0.942 | 3.295 | 0.944 | 3.257 | 0.797 | 2.084 | 45.470 | 0.956 |
| | 4 | AIPW | -0.251 | -0.249 | 0.305 | -0.012 | 1.151 | 0.002 | 0.919 | 3.868 | 0.936 | 3.452 | 0.926 | 3.707 | 0.946 | 10.328 | 93.128 | 4.915 |
| | 4 | WAIPW | -0.251 | -0.249 | 0.175 | 0.030 | 1.071 | 0.002 | 0.932 | 3.551 | 0.939 | 3.390 | 0.937 | 3.447 | 0.652 | 2.381 | 30.779 | 0.686 |
| IAIPW(0.25) | 4 | IPW | -0.251 | -0.242 | 0.176 | 0.054 | 1.033 | 0.009 | 0.946 | 3.197 | 0.938 | 3.401 | 0.954 | 2.963 | 0.675 | 1.020 | 31.078 | 0.614 |
| | 4 | WIPW | -0.251 | -0.243 | 0.174 | 0.052 | 1.033 | 0.008 | 0.944 | 3.246 | 0.941 | 3.343 | 0.956 | 2.901 | 0.666 | 0.966 | 30.296 | 0.596 |

| Randomization Method | Regime | Estimator | True Value | Mean Estimate | SD Estimates | Mean Normalized | SD Normalized | Mean Bias | CI Coverage | SE CI Coverage x 10^3 | LB Coverage | SE LB Coverage x 10^3 | UB Coverage | SE UB Coverage x 10^3 | Mean CI Length | SE CI Length x 10^3 | MSE x 10^3 | SE MSE x 10^3 |
|---|---|---|---|---|---|---|---|---|---|---|---|---|---|---|---|---|---|---|
| | 4 | AIPW | -0.251 | -0.246 | 0.140 | 0.040 | 1.035 | 0.005 | 0.944 | 3.241 | 0.937 | 3.426 | 0.951 | 3.059 | 0.539 | 0.696 | 19.740 | 0.386 |
| | 4 | WAIPW | -0.251 | -0.246 | 0.140 | 0.039 | 1.034 | 0.005 | 0.944 | 3.263 | 0.937 | 3.426 | 0.952 | 3.011 | 0.537 | 0.658 | 19.638 | 0.384 |
| IAIPW(0.5) | 4 | IPW | -0.251 | -0.251 | 0.198 | -0.004 | 1.024 | 0.000 | 0.940 | 3.353 | 0.946 | 3.197 | 0.946 | 3.197 | 0.773 | 1.598 | 39.277 | 0.803 |
| | 4 | WIPW | -0.251 | -0.252 | 0.183 | -0.003 | 1.013 | -0.001 | 0.946 | 3.197 | 0.949 | 3.112 | 0.946 | 3.204 | 0.718 | 1.164 | 33.489 | 0.680 |
| | 4 | AIPW | -0.251 | -0.252 | 0.156 | -0.005 | 1.018 | -0.001 | 0.943 | 3.286 | 0.948 | 3.141 | 0.947 | 3.176 | 0.615 | 1.192 | 24.394 | 0.484 |
| | 4 | WAIPW | -0.251 | -0.251 | 0.147 | -0.002 | 1.012 | 0.000 | 0.948 | 3.141 | 0.946 | 3.183 | 0.947 | 3.155 | 0.581 | 0.843 | 21.738 | 0.440 |
| IAIPW(0.75) | 4 | IPW | -0.251 | -0.248 | 0.259 | 0.017 | 1.115 | 0.003 | 0.924 | 3.757 | 0.933 | 3.536 | 0.929 | 3.628 | 0.939 | 3.210 | 66.850 | 1.367 |
| | 4 | WIPW | -0.251 | -0.248 | 0.203 | 0.017 | 1.042 | 0.003 | 0.937 | 3.441 | 0.941 | 3.338 | 0.946 | 3.186 | 0.774 | 1.528 | 41.122 | 0.840 |
| | 4 | AIPW | -0.251 | -0.246 | 0.202 | 0.039 | 1.062 | 0.005 | 0.930 | 3.613 | 0.934 | 3.521 | 0.944 | 3.257 | 0.762 | 2.701 | 40.737 | 0.846 |
| | 4 | WAIPW | -0.251 | -0.247 | 0.164 | 0.033 | 1.039 | 0.004 | 0.938 | 3.406 | 0.942 | 3.311 | 0.946 | 3.197 | 0.630 | 1.245 | 26.898 | 0.545 |
| IAIPW(1) | 4 | IPW | -0.251 | -0.251 | 0.347 | 0.005 | 1.230 | 0.000 | 0.898 | 4.281 | 0.914 | 3.959 | 0.915 | 3.934 | 1.126 | 6.578 | 120.386 | 2.533 |
| | 4 | WIPW | -0.251 | -0.250 | 0.215 | 0.007 | 1.051 | 0.001 | 0.940 | 3.355 | 0.941 | 3.328 | 0.943 | 3.270 | 0.817 | 2.092 | 46.278 | 0.920 |
| | 4 | AIPW | -0.251 | -0.253 | 0.279 | 0.031 | 1.113 | -0.002 | 0.935 | 3.483 | 0.929 | 3.637 | 0.942 | 3.297 | 1.001 | 6.410 | 78.002 | 1.780 |
| | 4 | WAIPW | -0.251 | -0.250 | 0.176 | 0.013 | 1.050 | 0.001 | 0.939 | 3.394 | 0.943 | 3.284 | 0.947 | 3.174 | 0.670 | 1.885 | 30.797 | 0.645 |
| IPW(0.25) | 4 | IPW | -0.251 | -0.252 | 0.175 | -0.007 | 1.032 | -0.001 | 0.941 | 3.343 | 0.949 | 3.106 | 0.943 | 3.279 | 0.672 | 0.992 | 30.739 | 0.619 |
| | 4 | WIPW | -0.251 | -0.249 | 0.262 | 0.007 | 1.079 | 0.002 | 0.928 | 3.651 | 0.935 | 3.477 | 0.935 | 3.477 | 0.984 | 2.428 | 68.649 | 1.439 |
| | 4 | AIPW | -0.251 | -0.250 | 0.139 | 0.005 | 1.026 | 0.001 | 0.943 | 3.274 | 0.946 | 3.208 | 0.948 | 3.152 | 0.539 | 0.694 | 19.243 | 0.382 |
| | 4 | WAIPW | -0.251 | -0.250 | 0.138 | 0.006 | 1.021 | 0.001 | 0.946 | 3.191 | 0.947 | 3.180 | 0.948 | 3.135 | 0.537 | 0.655 | 19.022 | 0.376 |
| IPW(0.5) | 4 | IPW | -0.251 | -0.253 | 0.205 | -0.011 | 1.051 | -0.002 | 0.937 | 3.443 | 0.946 | 3.190 | 0.940 | 3.353 | 0.778 | 1.644 | 41.836 | 0.832 |
| | 4 | WIPW | -0.251 | -0.256 | 0.328 | -0.020 | 1.159 | -0.005 | 0.905 | 4.148 | 0.928 | 3.645 | 0.918 | 3.881 | 1.192 | 4.170 | 107.713 | 2.128 |
| | 4 | AIPW | -0.251 | -0.253 | 0.162 | -0.008 | 1.057 | -0.002 | 0.933 | 3.524 | 0.942 | 3.313 | 0.938 | 3.411 | 0.617 | 1.237 | 26.347 | 0.529 |
| | 4 | WAIPW | -0.251 | -0.252 | 0.152 | -0.003 | 1.044 | -0.001 | 0.939 | 3.372 | 0.946 | 3.197 | 0.941 | 3.333 | 0.582 | 0.881 | 23.124 | 0.468 |
| IPW(0.75) | 4 | IPW | -0.251 | -0.247 | 0.258 | 0.020 | 1.107 | 0.004 | 0.927 | 3.670 | 0.931 | 3.585 | 0.937 | 3.447 | 0.946 | 3.287 | 66.643 | 1.342 |
| | 4 | WIPW | -0.251 | -0.248 | 0.419 | 0.008 | 1.325 | 0.003 | 0.865 | 4.830 | 0.898 | 4.273 | 0.902 | 4.201 | 1.386 | 7.044 | 175.801 | 3.594 |
| | 4 | AIPW | -0.251 | -0.250 | 0.204 | 0.014 | 1.079 | 0.001 | 0.932 | 3.570 | 0.935 | 3.497 | 0.941 | 3.343 | 0.761 | 2.765 | 41.657 | 0.843 |
| | 4 | WAIPW | -0.251 | -0.249 | 0.166 | 0.015 | 1.052 | 0.002 | 0.936 | 3.472 | 0.943 | 3.279 | 0.944 | 3.252 | 0.628 | 1.258 | 27.531 | 0.556 |
| IPW(1) | 4 | IPW | -0.251 | -0.250 | 0.358 | 0.003 | 1.252 | 0.001 | 0.889 | 4.449 | 0.901 | 4.218 | 0.906 | 4.131 | 1.131 | 6.491 | 128.494 | 2.689 |
| | 4 | WIPW | -0.251 | -0.251 | 0.520 | -0.007 | 1.518 | 0.000 | 0.817 | 5.471 | 0.863 | 4.856 | 0.863 | 4.868 | 1.401 | 10.249 | 270.050 | 5.844 |
| | 4 | AIPW | -0.251 | -0.253 | 0.286 | 0.011 | 1.109 | -0.002 | 0.933 | 3.524 | 0.927 | 3.679 | 0.938 | 3.411 | 1.001 | 6.285 | 81.799 | 1.837 |
| | 4 | WAIPW | -0.251 | -0.251 | 0.178 | 0.004 | 1.053 | 0.000 | 0.941 | 3.328 | 0.941 | 3.324 | 0.943 | 3.288 | 0.671 | 1.842 | 31.645 | 0.636 |
| SR | 4 | IPW | -0.251 | -0.248 | 0.160 | 0.021 | 1.021 | 0.003 | 0.944 | 3.247 | 0.943 | 3.269 | 0.951 | 3.042 | 0.620 | 0.814 | 25.503 | 0.515 |
| | 4 | WIPW | -0.251 | -0.251 | 0.223 | 0.000 | 1.056 | 0.000 | 0.934 | 3.522 | 0.938 | 3.411 | 0.943 | 3.279 | 0.847 | 1.734 | 49.532 | 0.998 |
| | 4 | AIPW | -0.251 | -0.247 | 0.130 | 0.035 | 1.027 | 0.004 | 0.941 | 3.343 | 0.944 | 3.263 | 0.950 | 3.077 | 0.502 | 0.542 | 16.842 | 0.337 |
| | 4 | WAIPW | -0.251 | -0.247 | 0.131 | 0.035 | 1.025 | 0.004 | 0.940 | 3.349 | 0.944 | 3.263 | 0.949 | 3.118 | 0.506 | 0.547 | 17.061 | 0.341 |
| TS(0.25) | 4 | IPW | -0.251 | -0.264 | 0.393 | -0.071 | 1.635 | -0.013 | 0.872 | 4.725 | 0.906 | 4.120 | 0.891 | 4.408 | 0.901 | 6.872 | 154.754 | 6.321 |
| | 4 | WIPW | -0.251 | -0.256 | 0.238 | -0.069 | 1.096 | -0.005 | 0.934 | 3.507 | 0.949 | 3.112 | 0.930 | 3.618 | 0.807 | 3.273 | 56.894 | 2.427 |
| | 4 | AIPW | -0.251 | -0.245 | 1.007 | 0.061 | 1.205 | 0.006 | 0.910 | 4.044 | 0.916 | 3.927 | 0.932 | 3.556 | 1.190 | 53.284 | 1014.828 | 466.145 |
| | 4 | WAIPW | -0.251 | -0.248 | 0.442 | 0.043 | 1.109 | 0.003 | 0.932 | 3.551 | 0.928 | 3.661 | 0.946 | 3.191 | 0.820 | 21.655 | 195.007 | 75.018 |
| TS(0.50) | 4 | IPW | -0.251 | -0.287 | 0.402 | -0.143 | 1.824 | -0.036 | 0.864 | 4.845 | 0.921 | 3.811 | 0.867 | 4.797 | 0.961 | 6.905 | 162.954 | 6.014 |
| | 4 | WIPW | -0.251 | -0.262 | 0.277 | -0.139 | 1.140 | -0.011 | 0.922 | 3.788 | 0.952 | 3.029 | 0.909 | 4.064 | 0.874 | 4.317 | 76.675 | 2.850 |
| | 4 | AIPW | -0.251 | -0.234 | 0.777 | 0.090 | 1.216 | 0.017 | 0.910 | 4.040 | 0.914 | 3.974 | 0.940 | 3.354 | 1.249 | 39.189 | 604.285 | 163.399 |
| | 4 | WAIPW | -0.251 | -0.240 | 0.438 | 0.065 | 1.139 | 0.011 | 0.920 | 3.837 | 0.923 | 3.780 | 0.942 | 3.306 | 0.941 | 20.423 | 192.151 | 36.585 |
| TS(0.75) | 4 | IPW | -0.251 | -0.322 | 0.399 | -0.297 | 1.824 | -0.071 | 0.854 | 5.000 | 0.927 | 3.675 | 0.847 | 5.089 | 0.976 | 6.962 | 164.120 | 5.678 |
| | 4 | WIPW | -0.251 | -0.287 | 0.285 | -0.252 | 1.227 | -0.036 | 0.901 | 4.217 | 0.947 | 3.163 | 0.887 | 4.485 | 0.876 | 4.389 | 82.461 | 2.761 |
| | 4 | AIPW | -0.251 | -0.268 | 1.749 | 0.096 | 1.226 | -0.017 | 0.907 | 4.104 | 0.910 | 4.044 | 0.940 | 3.369 | 1.389 | 94.787 | 3059.449 | 1947.767 |

| Randomization Method | Regime | Estimator | True Value | Mean Estimate | SD Estimates | Mean Normalized | SD Normalized | Mean Bias | CI Coverage | SE CI Coverage x 10^3 | LB Coverage | SE LB Coverage x 10^3 | UB Coverage | SE UB Coverage x 10^3 | Mean CI Length | SE CI Length x 10^3 | MSE x 10^3 | SE MSE x 10^3 |
|---|---|---|---|---|---|---|---|---|---|---|---|---|---|---|---|---|---|---|
| | 4 | WAIPW | -0.251 | -0.260 | 0.917 | 0.074 | 1.156 | -0.009 | 0.918 | 3.876 | 0.918 | 3.885 | 0.943 | 3.290 | 1.090 | 48.212 | 840.074 | 303.407 |
| TS(1) | 4 | IPW | -0.251 | -0.304 | 0.427 | -0.190 | 2.397 | -0.053 | 0.854 | 4.988 | 0.920 | 3.837 | 0.854 | 5.000 | 0.993 | 7.237 | 185.110 | 7.128 |
| | 4 | WIPW | -0.251 | -0.273 | 0.320 | -0.215 | 1.274 | -0.022 | 0.900 | 4.247 | 0.941 | 3.327 | 0.889 | 4.443 | 0.894 | 4.961 | 102.921 | 4.304 |
| | 4 | AIPW | -0.251 | -0.243 | 1.758 | 0.144 | 1.229 | 0.008 | 0.909 | 4.072 | 0.902 | 4.209 | 0.942 | 3.317 | 1.535 | 94.867 | 3088.904 | 1230.153 |
| | 4 | WAIPW | -0.251 | -0.233 | 1.298 | 0.128 | 1.168 | 0.018 | 0.919 | 3.859 | 0.916 | 3.927 | 0.948 | 3.140 | 1.243 | 69.638 | 1683.618 | 948.241 |
| WAIPW(0.25) | 4 | IPW | -0.251 | -0.252 | 0.176 | -0.005 | 1.031 | -0.001 | 0.944 | 3.257 | 0.952 | 3.035 | 0.944 | 3.241 | 0.674 | 0.991 | 30.854 | 0.614 |
| | 4 | WIPW | -0.251 | -0.252 | 0.173 | -0.003 | 1.029 | -0.001 | 0.943 | 3.274 | 0.950 | 3.088 | 0.944 | 3.252 | 0.665 | 0.938 | 30.059 | 0.597 |
| | 4 | AIPW | -0.251 | -0.251 | 0.141 | 0.000 | 1.041 | 0.000 | 0.940 | 3.348 | 0.945 | 3.230 | 0.939 | 3.380 | 0.540 | 0.699 | 19.963 | 0.386 |
| | 4 | WAIPW | -0.251 | -0.251 | 0.141 | 0.001 | 1.040 | 0.000 | 0.943 | 3.279 | 0.947 | 3.180 | 0.942 | 3.317 | 0.538 | 0.658 | 19.852 | 0.379 |
| WAIPW(0.5) | 4 | IPW | -0.251 | -0.247 | 0.205 | 0.021 | 1.054 | 0.004 | 0.941 | 3.340 | 0.938 | 3.405 | 0.943 | 3.280 | 0.777 | 1.635 | 42.026 | 0.826 |
| | 4 | WIPW | -0.251 | -0.247 | 0.188 | 0.020 | 1.034 | 0.004 | 0.942 | 3.306 | 0.942 | 3.293 | 0.950 | 3.090 | 0.721 | 1.202 | 35.236 | 0.703 |
| | 4 | AIPW | -0.251 | -0.248 | 0.162 | 0.026 | 1.057 | 0.003 | 0.931 | 3.573 | 0.939 | 3.379 | 0.943 | 3.273 | 0.615 | 1.177 | 26.371 | 0.547 |
| | 4 | WAIPW | -0.251 | -0.248 | 0.152 | 0.025 | 1.042 | 0.003 | 0.934 | 3.512 | 0.940 | 3.366 | 0.945 | 3.218 | 0.581 | 0.838 | 23.128 | 0.479 |
| WAIPW(0.75) | 4 | IPW | -0.251 | -0.252 | 0.257 | -0.006 | 1.112 | -0.001 | 0.919 | 3.855 | 0.932 | 3.556 | 0.931 | 3.575 | 0.942 | 3.166 | 66.096 | 1.343 |
| | 4 | WIPW | -0.251 | -0.253 | 0.202 | -0.011 | 1.043 | -0.002 | 0.936 | 3.452 | 0.945 | 3.213 | 0.942 | 3.317 | 0.775 | 1.537 | 40.885 | 0.841 |
| | 4 | AIPW | -0.251 | -0.250 | 0.201 | 0.014 | 1.071 | 0.001 | 0.935 | 3.477 | 0.941 | 3.333 | 0.940 | 3.369 | 0.758 | 2.666 | 40.325 | 0.817 |
| | 4 | WAIPW | -0.251 | -0.251 | 0.164 | 0.003 | 1.045 | 0.000 | 0.940 | 3.348 | 0.945 | 3.235 | 0.942 | 3.317 | 0.628 | 1.244 | 26.848 | 0.540 |
| WAIPW(1) | 4 | IPW | -0.251 | -0.251 | 0.350 | 0.014 | 1.242 | 0.000 | 0.890 | 4.423 | 0.904 | 4.170 | 0.909 | 4.071 | 1.126 | 6.618 | 122.168 | 2.651 |
| | 4 | WIPW | -0.251 | -0.248 | 0.218 | 0.019 | 1.059 | 0.003 | 0.933 | 3.532 | 0.940 | 3.350 | 0.944 | 3.252 | 0.817 | 2.101 | 47.511 | 0.994 |
| | 4 | AIPW | -0.251 | -0.248 | 0.279 | 0.032 | 1.088 | 0.003 | 0.937 | 3.441 | 0.933 | 3.545 | 0.943 | 3.275 | 0.994 | 6.337 | 77.927 | 1.878 |
| | 4 | WAIPW | -0.251 | -0.247 | 0.176 | 0.025 | 1.045 | 0.004 | 0.934 | 3.499 | 0.936 | 3.462 | 0.947 | 3.178 | 0.668 | 1.858 | 30.958 | 0.678 |
| WIPW(0.25) | 4 | IPW | -0.251 | -0.250 | 0.175 | 0.009 | 1.025 | 0.001 | 0.938 | 3.401 | 0.947 | 3.169 | 0.945 | 3.230 | 0.674 | 1.005 | 30.450 | 0.598 |
| | 4 | WIPW | -0.251 | -0.250 | 0.172 | 0.007 | 1.024 | 0.001 | 0.941 | 3.327 | 0.946 | 3.191 | 0.947 | 3.174 | 0.665 | 0.947 | 29.658 | 0.585 |
| | 4 | AIPW | -0.251 | -0.249 | 0.140 | 0.016 | 1.033 | 0.002 | 0.940 | 3.359 | 0.943 | 3.268 | 0.949 | 3.117 | 0.539 | 0.692 | 19.622 | 0.395 |
| | 4 | WAIPW | -0.251 | -0.249 | 0.140 | 0.012 | 1.034 | 0.002 | 0.941 | 3.333 | 0.942 | 3.295 | 0.948 | 3.146 | 0.537 | 0.652 | 19.595 | 0.392 |
| WIPW(0.5) | 4 | IPW | -0.251 | -0.250 | 0.200 | 0.003 | 1.026 | 0.001 | 0.941 | 3.320 | 0.944 | 3.252 | 0.945 | 3.211 | 0.774 | 1.622 | 40.109 | 0.819 |
| | 4 | WIPW | -0.251 | -0.251 | 0.184 | -0.002 | 1.011 | 0.000 | 0.942 | 3.293 | 0.945 | 3.232 | 0.949 | 3.105 | 0.719 | 1.197 | 33.870 | 0.694 |
| | 4 | AIPW | -0.251 | -0.251 | 0.160 | 0.008 | 1.032 | 0.000 | 0.942 | 3.306 | 0.945 | 3.232 | 0.946 | 3.190 | 0.616 | 1.207 | 25.581 | 0.513 |
| | 4 | WAIPW | -0.251 | -0.251 | 0.148 | 0.005 | 1.014 | 0.000 | 0.943 | 3.266 | 0.950 | 3.068 | 0.949 | 3.098 | 0.582 | 0.858 | 22.036 | 0.442 |
| WIPW(0.75) | 4 | IPW | -0.251 | -0.252 | 0.263 | -0.015 | 1.128 | -0.001 | 0.918 | 3.889 | 0.932 | 3.565 | 0.925 | 3.734 | 0.943 | 3.247 | 68.896 | 1.409 |
| | 4 | WIPW | -0.251 | -0.252 | 0.206 | -0.014 | 1.058 | -0.001 | 0.935 | 3.477 | 0.946 | 3.208 | 0.935 | 3.497 | 0.776 | 1.562 | 42.373 | 0.872 |
| | 4 | AIPW | -0.251 | -0.251 | 0.205 | 0.011 | 1.076 | 0.000 | 0.937 | 3.447 | 0.937 | 3.436 | 0.944 | 3.246 | 0.763 | 2.704 | 42.002 | 0.840 |
| | 4 | WAIPW | -0.251 | -0.251 | 0.166 | 0.002 | 1.050 | 0.000 | 0.940 | 3.359 | 0.943 | 3.290 | 0.943 | 3.279 | 0.630 | 1.245 | 27.504 | 0.555 |
| WIPW(1) | 4 | IPW | -0.251 | -0.255 | 0.353 | -0.012 | 1.262 | -0.004 | 0.887 | 4.472 | 0.908 | 4.088 | 0.907 | 4.111 | 1.131 | 6.744 | 124.480 | 2.644 |
| | 4 | WIPW | -0.251 | -0.252 | 0.220 | -0.005 | 1.079 | -0.001 | 0.925 | 3.714 | 0.939 | 3.381 | 0.936 | 3.466 | 0.818 | 2.116 | 48.373 | 0.996 |
| | 4 | AIPW | -0.251 | -0.251 | 0.284 | 0.018 | 1.119 | 0.000 | 0.930 | 3.597 | 0.932 | 3.565 | 0.940 | 3.359 | 0.999 | 6.356 | 80.390 | 1.809 |
| | 4 | WAIPW | -0.251 | -0.251 | 0.180 | 0.001 | 1.069 | 0.000 | 0.936 | 3.449 | 0.938 | 3.407 | 0.940 | 3.355 | 0.670 | 1.848 | 32.230 | 0.649 |
| AIPW(0.25) | 5 | IPW | -2.408 | -2.407 | 0.153 | -0.012 | 1.008 | 0.000 | 0.947 | 3.169 | 0.952 | 3.017 | 0.944 | 3.257 | 0.601 | 0.815 | 23.377 | 0.476 |
| | 5 | WIPW | -2.408 | -2.409 | 0.205 | -0.011 | 1.034 | -0.001 | 0.942 | 3.317 | 0.945 | 3.235 | 0.945 | 3.230 | 0.787 | 1.797 | 41.822 | 0.883 |
| | 5 | AIPW | -2.408 | -2.409 | 0.125 | -0.019 | 1.016 | -0.001 | 0.946 | 3.208 | 0.949 | 3.123 | 0.945 | 3.230 | 0.488 | 0.547 | 15.737 | 0.319 |
| | 5 | WAIPW | -2.408 | -2.407 | 0.126 | -0.010 | 1.018 | 0.000 | 0.944 | 3.263 | 0.947 | 3.157 | 0.944 | 3.246 | 0.489 | 0.548 | 15.868 | 0.323 |
| AIPW(0.5) | 5 | IPW | -2.408 | -2.407 | 0.157 | -0.025 | 1.016 | 0.000 | 0.943 | 3.273 | 0.950 | 3.090 | 0.940 | 3.359 | 0.606 | 1.040 | 24.551 | 0.521 |
| | 5 | WIPW | -2.408 | -2.402 | 0.213 | 0.009 | 1.069 | 0.005 | 0.931 | 3.573 | 0.940 | 3.366 | 0.940 | 3.366 | 0.784 | 2.519 | 45.375 | 0.968 |
| | 5 | AIPW | -2.408 | -2.407 | 0.126 | -0.019 | 1.017 | 0.001 | 0.948 | 3.148 | 0.952 | 3.009 | 0.944 | 3.246 | 0.489 | 0.699 | 15.979 | 0.352 |
| | 5 | WAIPW | -2.408 | -2.404 | 0.126 | 0.001 | 1.024 | 0.004 | 0.944 | 3.259 | 0.948 | 3.148 | 0.947 | 3.169 | 0.486 | 0.678 | 15.946 | 0.348 |

| Randomization Method | Regime | Estimator | True Value | Mean Estimate | SD Estimates | Mean Normalized | SD Normalized | Mean Bias | CI Coverage | SE CI Coverage x 10^3 | LB Coverage | SE LB Coverage x 10^3 | UB Coverage | SE UB Coverage x 10^3 | Mean CI Length | SE CI Length x 10^3 | MSE x 10^3 | SE MSE x 10^3 |
|---|---|---|---|---|---|---|---|---|---|---|---|---|---|---|---|---|---|---|
| AIPW(0.75) | 5 | IPW | -2.408 | -2.406 | 0.168 | -0.031 | 1.034 | 0.002 | 0.940 | 3.364 | 0.945 | 3.235 | 0.945 | 3.235 | 0.632 | 1.730 | 28.078 | 0.623 |
| | 5 | WIPW | -2.408 | -2.397 | 0.231 | 0.028 | 1.092 | 0.011 | 0.928 | 3.661 | 0.931 | 3.575 | 0.939 | 3.375 | 0.813 | 3.849 | 53.335 | 1.350 |
| | 5 | AIPW | -2.408 | -2.405 | 0.134 | -0.018 | 1.034 | 0.003 | 0.943 | 3.268 | 0.946 | 3.202 | 0.944 | 3.241 | 0.506 | 1.218 | 17.864 | 0.395 |
| | 5 | WAIPW | -2.408 | -2.399 | 0.131 | 0.024 | 1.039 | 0.008 | 0.942 | 3.306 | 0.940 | 3.369 | 0.946 | 3.197 | 0.493 | 0.923 | 17.143 | 0.365 |
| AIPW(1) | 5 | IPW | -2.408 | -2.403 | 0.186 | -0.020 | 1.054 | 0.004 | 0.936 | 3.458 | 0.940 | 3.346 | 0.941 | 3.333 | 0.669 | 3.000 | 34.674 | 0.965 |
| | 5 | WIPW | -2.408 | -2.394 | 0.253 | 0.042 | 1.121 | 0.014 | 0.923 | 3.760 | 0.927 | 3.687 | 0.938 | 3.411 | 0.849 | 5.128 | 64.286 | 1.879 |
| | 5 | AIPW | -2.408 | -2.404 | 0.147 | -0.023 | 1.033 | 0.004 | 0.939 | 3.390 | 0.945 | 3.234 | 0.948 | 3.145 | 0.539 | 2.490 | 21.503 | 0.571 |
| | 5 | WAIPW | -2.408 | -2.397 | 0.133 | 0.030 | 1.025 | 0.011 | 0.941 | 3.333 | 0.941 | 3.324 | 0.950 | 3.073 | 0.504 | 1.240 | 17.787 | 0.403 |
| AR-1 | 5 | IPW | -2.408 | -2.409 | 0.147 | -0.003 | 1.018 | -0.002 | 0.945 | 3.230 | 0.947 | 3.174 | 0.951 | 3.041 | 0.568 | 0.921 | 21.522 | 0.435 |
| | 5 | WIPW | -2.408 | -2.411 | 0.142 | -0.016 | 1.015 | -0.004 | 0.947 | 3.157 | 0.948 | 3.129 | 0.947 | 3.157 | 0.550 | 0.946 | 20.235 | 0.414 |
| | 5 | AIPW | -2.408 | -2.408 | 0.120 | 0.007 | 1.018 | -0.001 | 0.942 | 3.295 | 0.947 | 3.163 | 0.948 | 3.146 | 0.464 | 0.602 | 14.513 | 0.291 |
| | 5 | WAIPW | -2.408 | -2.410 | 0.119 | -0.005 | 1.022 | -0.002 | 0.943 | 3.290 | 0.947 | 3.163 | 0.948 | 3.152 | 0.456 | 0.600 | 14.106 | 0.285 |
| AR-2 | 5 | IPW | -2.408 | -2.406 | 0.152 | 0.014 | 1.024 | 0.002 | 0.942 | 3.317 | 0.950 | 3.094 | 0.943 | 3.279 | 0.580 | 1.494 | 23.096 | 0.500 |
| | 5 | WIPW | -2.408 | -2.409 | 0.150 | -0.020 | 1.028 | -0.002 | 0.940 | 3.364 | 0.951 | 3.065 | 0.938 | 3.411 | 0.564 | 1.692 | 22.395 | 0.494 |
| | 5 | AIPW | -2.408 | -2.404 | 0.122 | 0.049 | 1.015 | 0.004 | 0.947 | 3.180 | 0.943 | 3.268 | 0.950 | 3.083 | 0.472 | 0.968 | 14.921 | 0.305 |
| | 5 | WAIPW | -2.408 | -2.406 | 0.121 | 0.028 | 1.020 | 0.001 | 0.948 | 3.135 | 0.945 | 3.213 | 0.947 | 3.157 | 0.464 | 1.075 | 14.659 | 0.301 |
| IAIPW(0.25) | 5 | IPW | -2.408 | -2.411 | 0.157 | -0.033 | 1.030 | -0.003 | 0.943 | 3.268 | 0.951 | 3.065 | 0.940 | 3.364 | 0.602 | 0.829 | 24.707 | 0.515 |
| | 5 | WIPW | -2.408 | -2.409 | 0.157 | -0.020 | 1.031 | -0.001 | 0.942 | 3.295 | 0.949 | 3.112 | 0.943 | 3.290 | 0.599 | 0.827 | 24.525 | 0.513 |
| | 5 | AIPW | -2.408 | -2.411 | 0.128 | -0.038 | 1.039 | -0.003 | 0.944 | 3.241 | 0.956 | 2.907 | 0.938 | 3.411 | 0.488 | 0.544 | 16.385 | 0.333 |
| | 5 | WAIPW | -2.408 | -2.410 | 0.129 | -0.030 | 1.041 | -0.002 | 0.944 | 3.246 | 0.950 | 3.071 | 0.937 | 3.447 | 0.489 | 0.547 | 16.536 | 0.336 |
| IAIPW(0.5) | 5 | IPW | -2.408 | -2.408 | 0.155 | -0.033 | 1.004 | -0.001 | 0.953 | 2.994 | 0.957 | 2.862 | 0.949 | 3.112 | 0.607 | 1.058 | 23.955 | 0.484 |
| | 5 | WIPW | -2.408 | -2.403 | 0.152 | -0.003 | 1.003 | 0.005 | 0.954 | 2.971 | 0.953 | 3.001 | 0.951 | 3.061 | 0.595 | 1.016 | 23.060 | 0.463 |
| | 5 | AIPW | -2.408 | -2.407 | 0.127 | -0.018 | 1.025 | 0.001 | 0.943 | 3.266 | 0.947 | 3.176 | 0.945 | 3.225 | 0.489 | 0.712 | 16.230 | 0.326 |
| | 5 | WAIPW | -2.408 | -2.403 | 0.127 | 0.006 | 1.032 | 0.004 | 0.944 | 3.259 | 0.943 | 3.280 | 0.948 | 3.141 | 0.486 | 0.691 | 16.197 | 0.325 |
| IAIPW(0.75) | 5 | IPW | -2.408 | -2.406 | 0.163 | -0.029 | 1.006 | 0.002 | 0.952 | 3.035 | 0.952 | 3.035 | 0.948 | 3.146 | 0.628 | 1.702 | 26.616 | 0.613 |
| | 5 | WIPW | -2.408 | -2.400 | 0.155 | 0.007 | 1.005 | 0.008 | 0.952 | 3.011 | 0.949 | 3.123 | 0.951 | 3.041 | 0.602 | 1.303 | 24.194 | 0.523 |
| | 5 | AIPW | -2.408 | -2.407 | 0.131 | -0.031 | 1.012 | 0.001 | 0.945 | 3.230 | 0.946 | 3.186 | 0.951 | 3.053 | 0.505 | 1.182 | 17.045 | 0.383 |
| | 5 | WAIPW | -2.408 | -2.402 | 0.128 | 0.008 | 1.018 | 0.006 | 0.945 | 3.219 | 0.943 | 3.268 | 0.950 | 3.071 | 0.493 | 0.911 | 16.406 | 0.354 |
| IAIPW(1) | 5 | IPW | -2.408 | -2.402 | 0.189 | -0.025 | 1.075 | 0.005 | 0.926 | 3.699 | 0.938 | 3.398 | 0.938 | 3.407 | 0.670 | 2.941 | 35.665 | 1.015 |
| | 5 | WIPW | -2.408 | -2.395 | 0.166 | 0.021 | 1.048 | 0.012 | 0.932 | 3.569 | 0.939 | 3.377 | 0.945 | 3.225 | 0.618 | 1.640 | 27.770 | 0.660 |
| | 5 | AIPW | -2.408 | -2.402 | 0.147 | -0.013 | 1.038 | 0.006 | 0.941 | 3.328 | 0.943 | 3.270 | 0.945 | 3.220 | 0.540 | 2.496 | 21.748 | 0.612 |
| | 5 | WAIPW | -2.408 | -2.396 | 0.134 | 0.038 | 1.033 | 0.012 | 0.942 | 3.293 | 0.940 | 3.355 | 0.948 | 3.126 | 0.505 | 1.249 | 18.133 | 0.420 |
| IPW(0.25) | 5 | IPW | -2.408 | -2.405 | 0.155 | -0.002 | 1.015 | 0.002 | 0.948 | 3.146 | 0.948 | 3.135 | 0.946 | 3.186 | 0.601 | 0.805 | 24.001 | 0.488 |
| | 5 | WIPW | -2.408 | -2.405 | 0.204 | 0.005 | 1.044 | 0.003 | 0.933 | 3.536 | 0.943 | 3.274 | 0.940 | 3.364 | 0.780 | 1.763 | 41.590 | 0.853 |
| | 5 | AIPW | -2.408 | -2.407 | 0.126 | -0.001 | 1.027 | 0.001 | 0.945 | 3.213 | 0.945 | 3.219 | 0.947 | 3.163 | 0.487 | 0.545 | 15.981 | 0.318 |
| | 5 | WAIPW | -2.408 | -2.406 | 0.127 | 0.003 | 1.026 | 0.002 | 0.944 | 3.252 | 0.944 | 3.246 | 0.948 | 3.135 | 0.488 | 0.546 | 16.054 | 0.319 |
| IPW(0.5) | 5 | IPW | -2.408 | -2.402 | 0.154 | 0.003 | 1.008 | 0.006 | 0.948 | 3.126 | 0.944 | 3.259 | 0.952 | 3.016 | 0.600 | 1.020 | 23.866 | 0.492 |
| | 5 | WIPW | -2.408 | -2.403 | 0.206 | 0.000 | 1.064 | 0.005 | 0.931 | 3.573 | 0.940 | 3.359 | 0.939 | 3.372 | 0.764 | 2.436 | 42.413 | 0.929 |
| | 5 | AIPW | -2.408 | -2.404 | 0.125 | 0.014 | 1.008 | 0.004 | 0.945 | 3.218 | 0.947 | 3.176 | 0.950 | 3.083 | 0.487 | 0.693 | 15.586 | 0.314 |
| | 5 | WAIPW | -2.408 | -2.402 | 0.125 | 0.026 | 1.016 | 0.005 | 0.944 | 3.259 | 0.942 | 3.293 | 0.946 | 3.204 | 0.483 | 0.670 | 15.598 | 0.312 |
| IPW(0.75) | 5 | IPW | -2.408 | -2.409 | 0.163 | -0.064 | 1.028 | -0.001 | 0.946 | 3.208 | 0.953 | 2.999 | 0.942 | 3.301 | 0.619 | 1.628 | 26.649 | 0.589 |
| | 5 | WIPW | -2.408 | -2.401 | 0.220 | -0.004 | 1.083 | 0.007 | 0.930 | 3.613 | 0.940 | 3.359 | 0.936 | 3.457 | 0.785 | 3.702 | 48.272 | 1.247 |
| | 5 | AIPW | -2.408 | -2.409 | 0.131 | -0.039 | 1.033 | -0.001 | 0.943 | 3.268 | 0.953 | 2.999 | 0.944 | 3.252 | 0.498 | 1.150 | 17.061 | 0.357 |
| | 5 | WAIPW | -2.408 | -2.406 | 0.128 | -0.011 | 1.035 | 0.002 | 0.943 | 3.290 | 0.952 | 3.029 | 0.945 | 3.235 | 0.486 | 0.892 | 16.266 | 0.335 |
| IPW(1) | 5 | IPW | -2.408 | -2.406 | 0.180 | -0.056 | 1.050 | 0.002 | 0.937 | 3.441 | 0.943 | 3.270 | 0.940 | 3.346 | 0.653 | 2.851 | 32.360 | 0.938 |

| Randomization Method | Regime | Estimator | True Value | Mean Estimate | SD Estimates | Mean Normalized | SD Normalized | Mean Bias | CI Coverage | SE CI Coverage x 10^3 | LB Coverage | SE LB Coverage x 10^3 | UB Coverage | SE UB Coverage x 10^3 | Mean CI Length | SE CI Length x 10^3 | MSE x 10^3 | SE MSE x 10^3 |
|---|---|---|---|---|---|---|---|---|---|---|---|---|---|---|---|---|---|---|
| | 5 | WIPW | -2.408 | -2.390 | 0.246 | 0.047 | 1.142 | 0.017 | 0.914 | 3.966 | 0.920 | 3.845 | 0.941 | 3.320 | 0.817 | 4.960 | 60.589 | 1.913 |
| | 5 | AIPW | -2.408 | -2.406 | 0.144 | -0.022 | 1.031 | 0.001 | 0.942 | 3.293 | 0.945 | 3.215 | 0.947 | 3.174 | 0.526 | 2.337 | 20.756 | 0.585 |
| | 5 | WAIPW | -2.408 | -2.401 | 0.131 | 0.016 | 1.027 | 0.006 | 0.946 | 3.188 | 0.942 | 3.306 | 0.949 | 3.121 | 0.496 | 1.199 | 17.223 | 0.376 |
| SR | 5 | IPW | -2.408 | -2.403 | 0.158 | 0.028 | 1.006 | 0.004 | 0.948 | 3.135 | 0.944 | 3.258 | 0.952 | 3.018 | 0.619 | 0.822 | 24.885 | 0.496 |
| | 5 | WIPW | -2.408 | -2.403 | 0.220 | 0.023 | 1.051 | 0.005 | 0.936 | 3.452 | 0.942 | 3.296 | 0.942 | 3.312 | 0.844 | 1.797 | 48.307 | 0.972 |
| | 5 | AIPW | -2.408 | -2.406 | 0.127 | 0.013 | 1.011 | 0.002 | 0.946 | 3.191 | 0.944 | 3.258 | 0.948 | 3.135 | 0.500 | 0.553 | 16.197 | 0.311 |
| | 5 | WAIPW | -2.408 | -2.406 | 0.128 | 0.014 | 1.009 | 0.002 | 0.945 | 3.214 | 0.945 | 3.236 | 0.949 | 3.118 | 0.504 | 0.557 | 16.388 | 0.313 |
| TS(0.25) | 5 | IPW | -2.408 | -2.406 | 0.156 | -0.007 | 1.028 | 0.002 | 0.946 | 3.208 | 0.948 | 3.152 | 0.941 | 3.322 | 0.595 | 1.377 | 24.369 | 0.567 |
| | 5 | WIPW | -2.408 | -2.403 | 0.152 | 0.011 | 1.028 | 0.005 | 0.944 | 3.246 | 0.946 | 3.197 | 0.943 | 3.268 | 0.581 | 0.943 | 23.064 | 0.486 |
| | 5 | AIPW | -2.408 | -2.404 | 0.129 | 0.015 | 1.037 | 0.004 | 0.938 | 3.421 | 0.940 | 3.364 | 0.950 | 3.088 | 0.485 | 1.461 | 16.745 | 0.528 |
| | 5 | WAIPW | -2.408 | -2.402 | 0.126 | 0.026 | 1.037 | 0.005 | 0.940 | 3.369 | 0.940 | 3.348 | 0.950 | 3.094 | 0.477 | 0.686 | 15.906 | 0.361 |
| TS(0.50) | 5 | IPW | -2.408 | -2.407 | 0.167 | -0.033 | 1.025 | 0.001 | 0.942 | 3.306 | 0.949 | 3.123 | 0.951 | 3.047 | 0.626 | 1.903 | 27.754 | 0.722 |
| | 5 | WIPW | -2.408 | -2.401 | 0.156 | 0.001 | 1.016 | 0.007 | 0.944 | 3.252 | 0.948 | 3.135 | 0.953 | 2.999 | 0.602 | 1.337 | 24.523 | 0.557 |
| | 5 | AIPW | -2.408 | -2.405 | 0.137 | -0.012 | 1.039 | 0.003 | 0.940 | 3.359 | 0.945 | 3.224 | 0.947 | 3.169 | 0.507 | 1.826 | 18.766 | 0.538 |
| | 5 | WAIPW | -2.408 | -2.400 | 0.132 | 0.019 | 1.039 | 0.008 | 0.939 | 3.390 | 0.940 | 3.359 | 0.950 | 3.088 | 0.493 | 0.974 | 17.370 | 0.400 |
| TS(0.75) | 5 | IPW | -2.408 | -2.408 | 0.185 | -0.049 | 1.048 | 0.000 | 0.937 | 3.436 | 0.946 | 3.186 | 0.937 | 3.431 | 0.673 | 3.051 | 34.048 | 1.060 |
| | 5 | WIPW | -2.408 | -2.398 | 0.164 | 0.003 | 1.019 | 0.010 | 0.944 | 3.246 | 0.948 | 3.152 | 0.945 | 3.230 | 0.628 | 1.790 | 26.934 | 0.636 |
| | 5 | AIPW | -2.408 | -2.407 | 0.152 | -0.031 | 1.037 | 0.001 | 0.943 | 3.274 | 0.944 | 3.241 | 0.947 | 3.180 | 0.549 | 3.426 | 23.194 | 1.172 |
| | 5 | WAIPW | -2.408 | -2.398 | 0.135 | 0.019 | 1.021 | 0.010 | 0.946 | 3.197 | 0.948 | 3.152 | 0.949 | 3.117 | 0.514 | 1.461 | 18.358 | 0.439 |
| TS(1) | 5 | IPW | -2.408 | -2.407 | 0.224 | -0.045 | 1.103 | 0.001 | 0.929 | 3.637 | 0.933 | 3.526 | 0.936 | 3.452 | 0.721 | 4.308 | 49.990 | 1.979 |
| | 5 | WIPW | -2.408 | -2.396 | 0.176 | 0.000 | 1.033 | 0.012 | 0.942 | 3.306 | 0.939 | 3.390 | 0.944 | 3.241 | 0.651 | 2.264 | 31.061 | 0.787 |
| | 5 | AIPW | -2.408 | -2.406 | 0.180 | -0.024 | 1.060 | 0.001 | 0.938 | 3.416 | 0.934 | 3.512 | 0.949 | 3.123 | 0.602 | 5.267 | 32.504 | 1.648 |
| | 5 | WAIPW | -2.408 | -2.396 | 0.143 | 0.026 | 1.028 | 0.012 | 0.940 | 3.348 | 0.938 | 3.421 | 0.953 | 2.981 | 0.534 | 1.941 | 20.464 | 0.498 |
| WAIPW(0.25) | 5 | IPW | -2.408 | -2.405 | 0.158 | 0.003 | 1.034 | 0.003 | 0.943 | 3.268 | 0.944 | 3.252 | 0.945 | 3.213 | 0.603 | 0.834 | 24.940 | 0.514 |
| | 5 | WIPW | -2.408 | -2.403 | 0.157 | 0.016 | 1.035 | 0.005 | 0.944 | 3.246 | 0.941 | 3.322 | 0.949 | 3.123 | 0.600 | 0.830 | 24.742 | 0.510 |
| | 5 | AIPW | -2.408 | -2.406 | 0.131 | 0.003 | 1.056 | 0.002 | 0.937 | 3.431 | 0.943 | 3.284 | 0.938 | 3.406 | 0.488 | 0.555 | 17.061 | 0.343 |
| | 5 | WAIPW | -2.408 | -2.404 | 0.131 | 0.013 | 1.060 | 0.004 | 0.935 | 3.482 | 0.944 | 3.263 | 0.939 | 3.375 | 0.490 | 0.557 | 17.283 | 0.348 |
| WAIPW(0.5) | 5 | IPW | -2.408 | -2.409 | 0.155 | -0.032 | 1.013 | -0.001 | 0.944 | 3.252 | 0.949 | 3.105 | 0.943 | 3.286 | 0.604 | 1.043 | 24.155 | 0.502 |
| | 5 | WIPW | -2.408 | -2.404 | 0.153 | -0.007 | 1.015 | 0.003 | 0.944 | 3.252 | 0.948 | 3.141 | 0.948 | 3.141 | 0.593 | 0.997 | 23.311 | 0.482 |
| | 5 | AIPW | -2.408 | -2.409 | 0.127 | -0.031 | 1.019 | -0.001 | 0.947 | 3.176 | 0.947 | 3.176 | 0.948 | 3.148 | 0.489 | 0.709 | 16.030 | 0.337 |
| | 5 | WAIPW | -2.408 | -2.406 | 0.126 | -0.010 | 1.024 | 0.002 | 0.944 | 3.259 | 0.945 | 3.218 | 0.951 | 3.039 | 0.485 | 0.683 | 15.972 | 0.334 |
| WAIPW(0.75) | 5 | IPW | -2.408 | -2.409 | 0.165 | -0.052 | 1.024 | -0.002 | 0.943 | 3.268 | 0.951 | 3.053 | 0.938 | 3.406 | 0.628 | 1.669 | 27.202 | 0.579 |
| | 5 | WIPW | -2.408 | -2.402 | 0.158 | -0.011 | 1.022 | 0.005 | 0.945 | 3.219 | 0.947 | 3.174 | 0.944 | 3.257 | 0.601 | 1.303 | 24.872 | 0.511 |
| | 5 | AIPW | -2.408 | -2.407 | 0.131 | -0.030 | 1.013 | 0.001 | 0.950 | 3.083 | 0.952 | 3.035 | 0.946 | 3.191 | 0.505 | 1.210 | 17.046 | 0.363 |
| | 5 | WAIPW | -2.408 | -2.401 | 0.128 | 0.012 | 1.019 | 0.007 | 0.948 | 3.152 | 0.948 | 3.152 | 0.952 | 3.017 | 0.492 | 0.921 | 16.492 | 0.346 |
| WAIPW(1) | 5 | IPW | -2.408 | -2.409 | 0.183 | -0.059 | 1.047 | -0.002 | 0.938 | 3.398 | 0.946 | 3.192 | 0.940 | 3.363 | 0.663 | 2.890 | 33.576 | 0.896 |
| | 5 | WIPW | -2.408 | -2.401 | 0.162 | -0.013 | 1.024 | 0.006 | 0.943 | 3.266 | 0.948 | 3.126 | 0.947 | 3.155 | 0.614 | 1.628 | 26.243 | 0.599 |
| | 5 | AIPW | -2.408 | -2.411 | 0.142 | -0.063 | 1.025 | -0.003 | 0.941 | 3.320 | 0.948 | 3.145 | 0.945 | 3.211 | 0.535 | 2.400 | 20.275 | 0.512 |
| | 5 | WAIPW | -2.408 | -2.403 | 0.131 | -0.016 | 1.018 | 0.004 | 0.944 | 3.243 | 0.947 | 3.174 | 0.944 | 3.257 | 0.503 | 1.233 | 17.196 | 0.381 |
| WIPW(0.25) | 5 | IPW | -2.408 | -2.406 | 0.155 | -0.006 | 1.019 | 0.002 | 0.944 | 3.241 | 0.949 | 3.112 | 0.949 | 3.100 | 0.599 | 0.809 | 24.086 | 0.498 |
| | 5 | WIPW | -2.408 | -2.403 | 0.155 | 0.010 | 1.023 | 0.005 | 0.944 | 3.252 | 0.947 | 3.174 | 0.949 | 3.117 | 0.595 | 0.805 | 24.015 | 0.496 |
| | 5 | AIPW | -2.408 | -2.406 | 0.126 | 0.004 | 1.026 | 0.002 | 0.940 | 3.359 | 0.947 | 3.157 | 0.943 | 3.274 | 0.486 | 0.537 | 15.943 | 0.318 |
| | 5 | WAIPW | -2.408 | -2.405 | 0.127 | 0.009 | 1.028 | 0.002 | 0.941 | 3.322 | 0.945 | 3.213 | 0.942 | 3.311 | 0.487 | 0.540 | 16.073 | 0.321 |
| WIPW(0.5) | 5 | IPW | -2.408 | -2.409 | 0.158 | -0.044 | 1.034 | -0.001 | 0.942 | 3.313 | 0.951 | 3.039 | 0.941 | 3.326 | 0.600 | 1.017 | 24.984 | 0.530 |
| | 5 | WIPW | -2.408 | -2.402 | 0.156 | -0.006 | 1.040 | 0.005 | 0.943 | 3.280 | 0.946 | 3.204 | 0.945 | 3.218 | 0.588 | 0.981 | 24.248 | 0.512 |

| Randomization Method | Regime | Estimator | True Value | Mean Estimate | SD Estimates | Mean Normalized | SD Normalized | Mean Bias | CI Coverage | SE CI Coverage x 10^3 | LB Coverage | SE LB Coverage x 10^3 | UB Coverage | SE UB Coverage x 10^3 | Mean CI Length | SE CI Length x 10^3 | MSE x 10^3 | SE MSE x 10^3 |
|---|---|---|---|---|---|---|---|---|---|---|---|---|---|---|---|---|---|---|
| | 5 | AIPW | -2.408 | -2.410 | 0.127 | -0.035 | 1.027 | -0.002 | 0.943 | 3.266 | 0.952 | 3.016 | 0.944 | 3.252 | 0.487 | 0.703 | 16.014 | 0.315 |
| | 5 | WAIPW | -2.408 | -2.407 | 0.126 | -0.018 | 1.032 | 0.000 | 0.941 | 3.333 | 0.948 | 3.148 | 0.944 | 3.246 | 0.483 | 0.685 | 15.915 | 0.314 |
| WIPW(0.75) | 5 | IPW | -2.408 | -2.408 | 0.163 | -0.057 | 1.027 | -0.001 | 0.943 | 3.284 | 0.948 | 3.135 | 0.941 | 3.327 | 0.620 | 1.622 | 26.593 | 0.618 |
| | 5 | WIPW | -2.408 | -2.399 | 0.155 | -0.003 | 1.022 | 0.009 | 0.945 | 3.235 | 0.944 | 3.241 | 0.946 | 3.208 | 0.594 | 1.281 | 24.242 | 0.533 |
| | 5 | AIPW | -2.408 | -2.407 | 0.128 | -0.021 | 1.019 | 0.000 | 0.940 | 3.369 | 0.946 | 3.197 | 0.946 | 3.202 | 0.499 | 1.142 | 16.461 | 0.341 |
| | 5 | WAIPW | -2.408 | -2.404 | 0.126 | 0.006 | 1.024 | 0.004 | 0.941 | 3.322 | 0.943 | 3.290 | 0.947 | 3.157 | 0.487 | 0.886 | 15.850 | 0.322 |
| WIPW(1) | 5 | IPW | -2.408 | -2.409 | 0.181 | -0.081 | 1.047 | -0.001 | 0.938 | 3.402 | 0.946 | 3.188 | 0.939 | 3.394 | 0.650 | 2.570 | 32.652 | 0.919 |
| | 5 | WIPW | -2.408 | -2.398 | 0.161 | -0.015 | 1.037 | 0.010 | 0.942 | 3.315 | 0.942 | 3.306 | 0.943 | 3.284 | 0.604 | 1.547 | 26.158 | 0.597 |
| | 5 | AIPW | -2.408 | -2.408 | 0.143 | -0.039 | 1.041 | -0.001 | 0.939 | 3.385 | 0.944 | 3.243 | 0.942 | 3.315 | 0.525 | 2.258 | 20.574 | 0.610 |
| | 5 | WAIPW | -2.408 | -2.404 | 0.132 | -0.007 | 1.040 | 0.004 | 0.937 | 3.437 | 0.945 | 3.234 | 0.942 | 3.302 | 0.496 | 1.174 | 17.495 | 0.399 |
| AIPW(0.25) | 6 | IPW | -2.401 | -2.400 | 0.155 | -0.010 | 1.013 | 0.001 | 0.944 | 3.257 | 0.949 | 3.100 | 0.945 | 3.224 | 0.604 | 0.819 | 23.928 | 0.489 |
| | 6 | WIPW | -2.401 | -2.402 | 0.207 | -0.015 | 1.039 | -0.001 | 0.939 | 3.390 | 0.945 | 3.213 | 0.945 | 3.219 | 0.792 | 1.827 | 42.701 | 0.897 |
| | 6 | AIPW | -2.401 | -2.400 | 0.127 | -0.005 | 1.025 | 0.001 | 0.942 | 3.295 | 0.950 | 3.083 | 0.944 | 3.263 | 0.489 | 0.545 | 16.101 | 0.325 |
| | 6 | WAIPW | -2.401 | -2.400 | 0.128 | -0.007 | 1.026 | 0.001 | 0.942 | 3.295 | 0.949 | 3.117 | 0.943 | 3.274 | 0.492 | 0.553 | 16.281 | 0.329 |
| AIPW(0.5) | 6 | IPW | -2.401 | -2.401 | 0.158 | -0.028 | 1.018 | 0.000 | 0.944 | 3.252 | 0.951 | 3.054 | 0.944 | 3.252 | 0.610 | 1.038 | 25.001 | 0.535 |
| | 6 | WIPW | -2.401 | -2.397 | 0.215 | -0.002 | 1.070 | 0.004 | 0.930 | 3.609 | 0.938 | 3.405 | 0.941 | 3.333 | 0.790 | 2.538 | 46.413 | 1.013 |
| | 6 | AIPW | -2.401 | -2.399 | 0.128 | -0.011 | 1.025 | 0.002 | 0.942 | 3.306 | 0.949 | 3.112 | 0.947 | 3.169 | 0.491 | 0.700 | 16.313 | 0.354 |
| | 6 | WAIPW | -2.401 | -2.398 | 0.128 | -0.003 | 1.033 | 0.003 | 0.940 | 3.353 | 0.946 | 3.183 | 0.947 | 3.176 | 0.490 | 0.693 | 16.395 | 0.351 |
| AIPW(0.75) | 6 | IPW | -2.401 | -2.399 | 0.169 | -0.027 | 1.040 | 0.002 | 0.941 | 3.322 | 0.945 | 3.213 | 0.944 | 3.263 | 0.635 | 1.742 | 28.710 | 0.645 |
| | 6 | WIPW | -2.401 | -2.390 | 0.233 | 0.027 | 1.097 | 0.011 | 0.926 | 3.702 | 0.931 | 3.580 | 0.942 | 3.301 | 0.818 | 3.882 | 54.531 | 1.391 |
| | 6 | AIPW | -2.401 | -2.398 | 0.135 | -0.016 | 1.040 | 0.003 | 0.940 | 3.348 | 0.946 | 3.191 | 0.945 | 3.230 | 0.507 | 1.216 | 18.202 | 0.409 |
| | 6 | WAIPW | -2.401 | -2.394 | 0.132 | 0.013 | 1.045 | 0.007 | 0.939 | 3.395 | 0.941 | 3.327 | 0.943 | 3.268 | 0.496 | 0.940 | 17.566 | 0.376 |
| AIPW(1) | 6 | IPW | -2.401 | -2.396 | 0.187 | -0.022 | 1.050 | 0.005 | 0.938 | 3.398 | 0.941 | 3.333 | 0.947 | 3.159 | 0.676 | 3.056 | 35.126 | 0.974 |
| | 6 | WIPW | -2.401 | -2.386 | 0.258 | 0.035 | 1.125 | 0.015 | 0.918 | 3.884 | 0.928 | 3.652 | 0.939 | 3.381 | 0.861 | 5.308 | 66.827 | 1.957 |
| | 6 | AIPW | -2.401 | -2.396 | 0.147 | -0.007 | 1.036 | 0.005 | 0.942 | 3.311 | 0.940 | 3.363 | 0.951 | 3.039 | 0.541 | 2.509 | 21.762 | 0.580 |
| | 6 | WAIPW | -2.401 | -2.391 | 0.136 | 0.029 | 1.031 | 0.010 | 0.942 | 3.315 | 0.939 | 3.381 | 0.954 | 2.973 | 0.511 | 1.501 | 18.679 | 0.438 |
| AR-1 | 6 | IPW | -2.401 | -2.398 | 0.147 | 0.035 | 1.014 | 0.003 | 0.948 | 3.135 | 0.943 | 3.268 | 0.953 | 3.005 | 0.570 | 0.944 | 21.476 | 0.427 |
| | 6 | WIPW | -2.401 | -2.399 | 0.143 | 0.025 | 1.015 | 0.002 | 0.946 | 3.197 | 0.944 | 3.257 | 0.949 | 3.106 | 0.554 | 0.982 | 20.372 | 0.406 |
| | 6 | AIPW | -2.401 | -2.402 | 0.121 | -0.002 | 1.027 | -0.001 | 0.943 | 3.279 | 0.945 | 3.224 | 0.948 | 3.129 | 0.465 | 0.625 | 14.667 | 0.294 |
| | 6 | WAIPW | -2.401 | -2.402 | 0.120 | -0.003 | 1.031 | -0.001 | 0.944 | 3.257 | 0.946 | 3.197 | 0.950 | 3.083 | 0.460 | 0.823 | 14.486 | 0.294 |
| AR-2 | 6 | IPW | -2.401 | -2.396 | 0.148 | 0.052 | 1.009 | 0.005 | 0.945 | 3.219 | 0.940 | 3.359 | 0.952 | 3.035 | 0.577 | 1.375 | 21.811 | 0.465 |
| | 6 | WIPW | -2.401 | -2.398 | 0.145 | 0.031 | 1.012 | 0.003 | 0.946 | 3.191 | 0.944 | 3.252 | 0.948 | 3.129 | 0.563 | 1.408 | 20.974 | 0.446 |
| | 6 | AIPW | -2.401 | -2.398 | 0.121 | 0.041 | 1.020 | 0.003 | 0.944 | 3.252 | 0.942 | 3.306 | 0.948 | 3.146 | 0.469 | 0.854 | 14.722 | 0.306 |
| | 6 | WAIPW | -2.401 | -2.399 | 0.121 | 0.033 | 1.024 | 0.002 | 0.942 | 3.311 | 0.942 | 3.311 | 0.946 | 3.191 | 0.465 | 1.128 | 14.612 | 0.310 |
| IAIPW(0.25) | 6 | IPW | -2.401 | -2.406 | 0.156 | -0.045 | 1.015 | -0.005 | 0.944 | 3.241 | 0.953 | 2.999 | 0.942 | 3.306 | 0.605 | 0.839 | 24.229 | 0.500 |
| | 6 | WIPW | -2.401 | -2.404 | 0.155 | -0.033 | 1.017 | -0.003 | 0.944 | 3.241 | 0.951 | 3.047 | 0.942 | 3.301 | 0.602 | 0.838 | 24.050 | 0.497 |
| | 6 | AIPW | -2.401 | -2.405 | 0.129 | -0.042 | 1.043 | -0.004 | 0.940 | 3.348 | 0.952 | 3.017 | 0.933 | 3.531 | 0.488 | 0.547 | 16.568 | 0.333 |
| | 6 | WAIPW | -2.401 | -2.405 | 0.130 | -0.047 | 1.045 | -0.004 | 0.943 | 3.284 | 0.955 | 2.938 | 0.932 | 3.561 | 0.491 | 0.557 | 16.798 | 0.338 |
| IAIPW(0.5) | 6 | IPW | -2.401 | -2.404 | 0.155 | -0.048 | 0.999 | -0.003 | 0.954 | 2.971 | 0.956 | 2.893 | 0.949 | 3.105 | 0.609 | 1.060 | 23.948 | 0.481 |
| | 6 | WIPW | -2.401 | -2.399 | 0.152 | -0.019 | 1.001 | 0.002 | 0.954 | 2.948 | 0.954 | 2.971 | 0.953 | 3.001 | 0.598 | 1.016 | 23.175 | 0.467 |
| | 6 | AIPW | -2.401 | -2.401 | 0.128 | -0.030 | 1.030 | 0.000 | 0.949 | 3.119 | 0.948 | 3.126 | 0.943 | 3.273 | 0.490 | 0.707 | 16.371 | 0.327 |
| | 6 | WAIPW | -2.401 | -2.400 | 0.128 | -0.021 | 1.033 | 0.001 | 0.947 | 3.155 | 0.947 | 3.169 | 0.944 | 3.259 | 0.488 | 0.700 | 16.340 | 0.326 |
| IAIPW(0.75) | 6 | IPW | -2.401 | -2.400 | 0.167 | -0.038 | 1.024 | 0.001 | 0.944 | 3.241 | 0.949 | 3.112 | 0.945 | 3.213 | 0.633 | 1.732 | 27.906 | 0.643 |
| | 6 | WIPW | -2.401 | -2.394 | 0.159 | 0.000 | 1.021 | 0.007 | 0.944 | 3.252 | 0.945 | 3.224 | 0.949 | 3.100 | 0.607 | 1.333 | 25.292 | 0.552 |
| | 6 | AIPW | -2.401 | -2.399 | 0.132 | -0.025 | 1.023 | 0.002 | 0.945 | 3.235 | 0.941 | 3.322 | 0.948 | 3.135 | 0.506 | 1.188 | 17.516 | 0.396 |

| Randomization Method | Regime | Estimator | True Value | Mean Estimate | SD Estimates | Mean Normalized | SD Normalized | Mean Bias | CI Coverage | SE CI Coverage x 10^3 | LB Coverage | SE LB Coverage x 10^3 | UB Coverage | SE UB Coverage x 10^3 | Mean CI Length | SE CI Length x 10^3 | MSE x 10^3 | SE MSE x 10^3 |
|---|---|---|---|---|---|---|---|---|---|---|---|---|---|---|---|---|---|---|
| | 6 | WAIPW | -2.401 | -2.396 | 0.130 | -0.001 | 1.030 | 0.005 | 0.942 | 3.317 | 0.942 | 3.306 | 0.947 | 3.157 | 0.497 | 0.967 | 17.030 | 0.370 |
| IAIPW(1) | 6 | IPW | -2.401 | -2.394 | 0.191 | -0.016 | 1.076 | 0.007 | 0.928 | 3.656 | 0.936 | 3.462 | 0.936 | 3.462 | 0.675 | 2.959 | 36.540 | 1.024 |
| | 6 | WIPW | -2.401 | -2.388 | 0.169 | 0.030 | 1.052 | 0.013 | 0.929 | 3.633 | 0.937 | 3.445 | 0.941 | 3.324 | 0.625 | 1.664 | 28.578 | 0.661 |
| | 6 | AIPW | -2.401 | -2.392 | 0.148 | 0.013 | 1.043 | 0.009 | 0.940 | 3.350 | 0.939 | 3.372 | 0.946 | 3.202 | 0.541 | 2.488 | 22.091 | 0.618 |
| | 6 | WAIPW | -2.401 | -2.388 | 0.136 | 0.049 | 1.038 | 0.013 | 0.939 | 3.377 | 0.939 | 3.390 | 0.947 | 3.174 | 0.509 | 1.341 | 18.687 | 0.427 |
| IPW(0.25) | 6 | IPW | -2.401 | -2.399 | 0.154 | -0.005 | 1.008 | 0.002 | 0.945 | 3.219 | 0.949 | 3.117 | 0.947 | 3.163 | 0.604 | 0.814 | 23.827 | 0.482 |
| | 6 | WIPW | -2.401 | -2.400 | 0.204 | -0.005 | 1.037 | 0.001 | 0.941 | 3.343 | 0.945 | 3.219 | 0.946 | 3.186 | 0.785 | 1.768 | 41.767 | 0.845 |
| | 6 | AIPW | -2.401 | -2.400 | 0.127 | -0.005 | 1.029 | 0.001 | 0.946 | 3.202 | 0.945 | 3.213 | 0.947 | 3.180 | 0.488 | 0.541 | 16.048 | 0.314 |
| | 6 | WAIPW | -2.401 | -2.401 | 0.127 | -0.014 | 1.028 | 0.000 | 0.946 | 3.208 | 0.948 | 3.152 | 0.943 | 3.268 | 0.490 | 0.547 | 16.200 | 0.316 |
| IPW(0.5) | 6 | IPW | -2.401 | -2.395 | 0.155 | 0.001 | 1.006 | 0.006 | 0.947 | 3.155 | 0.947 | 3.169 | 0.949 | 3.098 | 0.604 | 1.021 | 24.137 | 0.508 |
| | 6 | WIPW | -2.401 | -2.398 | 0.205 | -0.010 | 1.051 | 0.003 | 0.940 | 3.359 | 0.942 | 3.293 | 0.936 | 3.456 | 0.770 | 2.478 | 41.904 | 0.922 |
| | 6 | AIPW | -2.401 | -2.396 | 0.126 | 0.027 | 1.018 | 0.005 | 0.944 | 3.246 | 0.942 | 3.306 | 0.951 | 3.039 | 0.487 | 0.691 | 15.891 | 0.320 |
| | 6 | WAIPW | -2.401 | -2.395 | 0.127 | 0.025 | 1.029 | 0.006 | 0.940 | 3.346 | 0.939 | 3.372 | 0.946 | 3.183 | 0.485 | 0.672 | 16.090 | 0.322 |
| IPW(0.75) | 6 | IPW | -2.401 | -2.404 | 0.164 | -0.071 | 1.024 | -0.003 | 0.948 | 3.146 | 0.958 | 2.831 | 0.942 | 3.301 | 0.622 | 1.616 | 26.813 | 0.601 |
| | 6 | WIPW | -2.401 | -2.395 | 0.221 | -0.009 | 1.077 | 0.006 | 0.933 | 3.531 | 0.943 | 3.279 | 0.937 | 3.436 | 0.790 | 3.678 | 48.795 | 1.305 |
| | 6 | AIPW | -2.401 | -2.403 | 0.130 | -0.040 | 1.032 | -0.002 | 0.943 | 3.290 | 0.950 | 3.088 | 0.944 | 3.252 | 0.499 | 1.143 | 16.962 | 0.361 |
| | 6 | WAIPW | -2.401 | -2.400 | 0.128 | -0.023 | 1.035 | 0.001 | 0.943 | 3.284 | 0.948 | 3.146 | 0.943 | 3.274 | 0.488 | 0.887 | 16.341 | 0.341 |
| IPW(1) | 6 | IPW | -2.401 | -2.398 | 0.180 | -0.054 | 1.049 | 0.003 | 0.933 | 3.540 | 0.945 | 3.211 | 0.939 | 3.385 | 0.657 | 2.842 | 32.346 | 0.926 |
| | 6 | WIPW | -2.401 | -2.384 | 0.245 | 0.042 | 1.148 | 0.017 | 0.914 | 3.955 | 0.925 | 3.726 | 0.938 | 3.398 | 0.822 | 4.955 | 60.353 | 1.859 |
| | 6 | AIPW | -2.401 | -2.399 | 0.144 | -0.018 | 1.035 | 0.002 | 0.944 | 3.261 | 0.945 | 3.234 | 0.944 | 3.248 | 0.527 | 2.318 | 20.632 | 0.570 |
| | 6 | WAIPW | -2.401 | -2.395 | 0.133 | 0.007 | 1.041 | 0.006 | 0.940 | 3.368 | 0.942 | 3.315 | 0.945 | 3.234 | 0.499 | 1.193 | 17.677 | 0.386 |
| SR | 6 | IPW | -2.401 | -2.395 | 0.158 | 0.035 | 1.005 | 0.006 | 0.950 | 3.083 | 0.943 | 3.279 | 0.957 | 2.876 | 0.621 | 0.822 | 25.094 | 0.509 |
| | 6 | WIPW | -2.401 | -2.395 | 0.222 | 0.028 | 1.057 | 0.006 | 0.934 | 3.507 | 0.937 | 3.432 | 0.942 | 3.317 | 0.848 | 1.795 | 49.435 | 0.998 |
| | 6 | AIPW | -2.401 | -2.399 | 0.129 | 0.012 | 1.018 | 0.002 | 0.946 | 3.197 | 0.944 | 3.252 | 0.946 | 3.208 | 0.501 | 0.552 | 16.513 | 0.322 |
| | 6 | WAIPW | -2.401 | -2.400 | 0.130 | 0.005 | 1.015 | 0.001 | 0.945 | 3.236 | 0.946 | 3.186 | 0.944 | 3.252 | 0.506 | 0.561 | 16.798 | 0.324 |
| TS(0.25) | 6 | IPW | -2.401 | -2.394 | 0.158 | 0.025 | 1.031 | 0.007 | 0.942 | 3.301 | 0.943 | 3.284 | 0.949 | 3.123 | 0.599 | 1.425 | 24.960 | 0.568 |
| | 6 | WIPW | -2.401 | -2.391 | 0.154 | 0.045 | 1.033 | 0.010 | 0.940 | 3.348 | 0.943 | 3.268 | 0.949 | 3.100 | 0.585 | 1.031 | 23.788 | 0.503 |
| | 6 | AIPW | -2.401 | -2.396 | 0.130 | 0.023 | 1.038 | 0.005 | 0.941 | 3.343 | 0.941 | 3.327 | 0.950 | 3.083 | 0.487 | 1.484 | 16.915 | 0.521 |
| | 6 | WAIPW | -2.401 | -2.394 | 0.127 | 0.032 | 1.040 | 0.007 | 0.942 | 3.295 | 0.940 | 3.354 | 0.949 | 3.106 | 0.480 | 0.729 | 16.228 | 0.366 |
| TS(0.50) | 6 | IPW | -2.401 | -2.396 | 0.169 | -0.013 | 1.032 | 0.005 | 0.939 | 3.395 | 0.945 | 3.230 | 0.948 | 3.135 | 0.631 | 1.958 | 28.584 | 0.735 |
| | 6 | WIPW | -2.401 | -2.390 | 0.160 | 0.024 | 1.028 | 0.011 | 0.937 | 3.426 | 0.942 | 3.306 | 0.950 | 3.094 | 0.606 | 1.419 | 25.714 | 0.576 |
| | 6 | AIPW | -2.401 | -2.397 | 0.139 | -0.006 | 1.044 | 0.004 | 0.936 | 3.457 | 0.940 | 3.354 | 0.946 | 3.191 | 0.511 | 1.970 | 19.367 | 0.589 |
| | 6 | WAIPW | -2.401 | -2.392 | 0.135 | 0.024 | 1.046 | 0.009 | 0.937 | 3.447 | 0.938 | 3.406 | 0.950 | 3.088 | 0.499 | 1.425 | 18.381 | 0.585 |
| TS(0.75) | 6 | IPW | -2.401 | -2.396 | 0.187 | -0.025 | 1.041 | 0.005 | 0.940 | 3.348 | 0.944 | 3.241 | 0.945 | 3.235 | 0.682 | 3.166 | 34.871 | 1.076 |
| | 6 | WIPW | -2.401 | -2.385 | 0.166 | 0.029 | 1.013 | 0.016 | 0.945 | 3.224 | 0.947 | 3.157 | 0.949 | 3.117 | 0.636 | 1.968 | 27.678 | 0.676 |
| | 6 | AIPW | -2.401 | -2.398 | 0.155 | -0.016 | 1.040 | 0.003 | 0.938 | 3.406 | 0.947 | 3.180 | 0.950 | 3.083 | 0.554 | 3.534 | 24.018 | 1.214 |
| | 6 | WAIPW | -2.401 | -2.390 | 0.140 | 0.030 | 1.025 | 0.011 | 0.942 | 3.295 | 0.944 | 3.246 | 0.952 | 3.017 | 0.522 | 1.977 | 19.726 | 0.693 |
| TS(1) | 6 | IPW | -2.401 | -2.397 | 0.227 | -0.031 | 1.099 | 0.004 | 0.930 | 3.618 | 0.932 | 3.551 | 0.944 | 3.263 | 0.731 | 4.424 | 51.754 | 2.081 |
| | 6 | WIPW | -2.401 | -2.384 | 0.181 | 0.021 | 1.035 | 0.017 | 0.941 | 3.322 | 0.939 | 3.385 | 0.953 | 2.993 | 0.660 | 2.511 | 33.018 | 0.906 |
| | 6 | AIPW | -2.401 | -2.396 | 0.185 | -0.006 | 1.073 | 0.005 | 0.937 | 3.447 | 0.930 | 3.609 | 0.951 | 3.059 | 0.607 | 5.343 | 34.255 | 1.774 |
| | 6 | WAIPW | -2.401 | -2.386 | 0.156 | 0.045 | 1.042 | 0.015 | 0.941 | 3.338 | 0.934 | 3.512 | 0.955 | 2.944 | 0.544 | 2.757 | 24.397 | 2.372 |
| WAIPW(0.25) | 6 | IPW | -2.401 | -2.399 | 0.160 | -0.001 | 1.038 | 0.002 | 0.942 | 3.295 | 0.944 | 3.241 | 0.945 | 3.219 | 0.606 | 0.844 | 25.444 | 0.531 |
| | 6 | WIPW | -2.401 | -2.397 | 0.159 | 0.010 | 1.039 | 0.004 | 0.944 | 3.263 | 0.944 | 3.252 | 0.946 | 3.208 | 0.603 | 0.839 | 25.287 | 0.529 |
| | 6 | AIPW | -2.401 | -2.398 | 0.131 | 0.008 | 1.054 | 0.003 | 0.938 | 3.416 | 0.941 | 3.338 | 0.938 | 3.406 | 0.489 | 0.556 | 17.052 | 0.343 |
| | 6 | WAIPW | -2.401 | -2.398 | 0.132 | 0.007 | 1.056 | 0.003 | 0.936 | 3.462 | 0.941 | 3.338 | 0.938 | 3.411 | 0.492 | 0.565 | 17.332 | 0.352 |

| Randomization Method | Regime | Estimator | True Value | Mean Estimate | SD Estimates | Mean Normalized | SD Normalized | Mean Bias | CI Coverage | SE CI Coverage x 10^3 | LB Coverage | SE LB Coverage x 10^3 | UB Coverage | SE UB Coverage x 10^3 | Mean CI Length | SE CI Length x 10^3 | MSE x 10^3 | SE MSE x 10^3 |
|---|---|---|---|---|---|---|---|---|---|---|---|---|---|---|---|---|---|---|
| WAIPW(0.5) | 6 | IPW | -2.401 | -2.401 | 0.157 | -0.023 | 1.017 | 0.000 | 0.945 | 3.218 | 0.948 | 3.141 | 0.948 | 3.141 | 0.608 | 1.055 | 24.588 | 0.515 |
| | 6 | WIPW | -2.401 | -2.396 | 0.154 | 0.000 | 1.017 | 0.005 | 0.946 | 3.204 | 0.944 | 3.239 | 0.950 | 3.076 | 0.597 | 1.014 | 23.731 | 0.496 |
| | 6 | AIPW | -2.401 | -2.400 | 0.128 | -0.020 | 1.030 | 0.001 | 0.941 | 3.326 | 0.945 | 3.225 | 0.946 | 3.183 | 0.490 | 0.708 | 16.309 | 0.332 |
| | 6 | WAIPW | -2.401 | -2.399 | 0.128 | -0.012 | 1.037 | 0.002 | 0.940 | 3.353 | 0.944 | 3.252 | 0.944 | 3.239 | 0.488 | 0.690 | 16.396 | 0.333 |
| WAIPW(0.75) | 6 | IPW | -2.401 | -2.402 | 0.166 | -0.044 | 1.028 | -0.001 | 0.941 | 3.333 | 0.952 | 3.011 | 0.938 | 3.401 | 0.632 | 1.702 | 27.682 | 0.598 |
| | 6 | WIPW | -2.401 | -2.395 | 0.159 | -0.006 | 1.026 | 0.006 | 0.941 | 3.327 | 0.947 | 3.169 | 0.944 | 3.252 | 0.607 | 1.321 | 25.355 | 0.529 |
| | 6 | AIPW | -2.401 | -2.399 | 0.131 | -0.018 | 1.016 | 0.002 | 0.946 | 3.208 | 0.951 | 3.065 | 0.946 | 3.208 | 0.507 | 1.207 | 17.241 | 0.370 |
| | 6 | WAIPW | -2.401 | -2.395 | 0.130 | 0.011 | 1.023 | 0.006 | 0.944 | 3.246 | 0.946 | 3.186 | 0.949 | 3.123 | 0.496 | 0.950 | 16.806 | 0.355 |
| WAIPW(1) | 6 | IPW | -2.401 | -2.402 | 0.184 | -0.054 | 1.045 | -0.001 | 0.939 | 3.377 | 0.945 | 3.215 | 0.943 | 3.275 | 0.668 | 2.932 | 33.902 | 0.903 |
| | 6 | WIPW | -2.401 | -2.394 | 0.163 | -0.010 | 1.025 | 0.007 | 0.942 | 3.311 | 0.945 | 3.215 | 0.947 | 3.159 | 0.620 | 1.652 | 26.624 | 0.599 |
| | 6 | AIPW | -2.401 | -2.402 | 0.143 | -0.046 | 1.034 | -0.001 | 0.941 | 3.333 | 0.944 | 3.257 | 0.946 | 3.202 | 0.536 | 2.395 | 20.566 | 0.523 |
| | 6 | WAIPW | -2.401 | -2.397 | 0.133 | -0.016 | 1.031 | 0.004 | 0.939 | 3.372 | 0.945 | 3.229 | 0.945 | 3.234 | 0.507 | 1.316 | 17.776 | 0.392 |
| WIPW(0.25) | 6 | IPW | -2.401 | -2.399 | 0.155 | -0.001 | 1.014 | 0.002 | 0.946 | 3.191 | 0.950 | 3.071 | 0.946 | 3.186 | 0.602 | 0.819 | 24.013 | 0.496 |
| | 6 | WIPW | -2.401 | -2.396 | 0.155 | 0.015 | 1.017 | 0.005 | 0.946 | 3.197 | 0.949 | 3.117 | 0.948 | 3.146 | 0.598 | 0.815 | 23.905 | 0.491 |
| | 6 | AIPW | -2.401 | -2.399 | 0.126 | 0.006 | 1.024 | 0.002 | 0.940 | 3.364 | 0.943 | 3.284 | 0.947 | 3.180 | 0.487 | 0.540 | 15.902 | 0.314 |
| | 6 | WAIPW | -2.401 | -2.399 | 0.127 | 0.002 | 1.026 | 0.002 | 0.942 | 3.317 | 0.949 | 3.100 | 0.944 | 3.257 | 0.489 | 0.545 | 16.132 | 0.319 |
| WIPW(0.5) | 6 | IPW | -2.401 | -2.403 | 0.157 | -0.045 | 1.025 | -0.002 | 0.942 | 3.313 | 0.952 | 3.024 | 0.940 | 3.346 | 0.603 | 1.026 | 24.694 | 0.535 |
| | 6 | WIPW | -2.401 | -2.396 | 0.154 | -0.009 | 1.028 | 0.005 | 0.942 | 3.300 | 0.947 | 3.169 | 0.945 | 3.218 | 0.592 | 0.989 | 23.879 | 0.514 |
| | 6 | AIPW | -2.401 | -2.403 | 0.127 | -0.032 | 1.031 | -0.002 | 0.943 | 3.273 | 0.947 | 3.155 | 0.942 | 3.313 | 0.488 | 0.698 | 16.161 | 0.325 |
| | 6 | WAIPW | -2.401 | -2.402 | 0.127 | -0.028 | 1.037 | -0.001 | 0.939 | 3.372 | 0.948 | 3.148 | 0.939 | 3.379 | 0.485 | 0.683 | 16.159 | 0.323 |
| WIPW(0.75) | 6 | IPW | -2.401 | -2.401 | 0.165 | -0.051 | 1.033 | 0.000 | 0.939 | 3.385 | 0.946 | 3.202 | 0.940 | 3.359 | 0.624 | 1.625 | 27.322 | 0.646 |
| | 6 | WIPW | -2.401 | -2.391 | 0.158 | 0.001 | 1.030 | 0.010 | 0.942 | 3.317 | 0.944 | 3.252 | 0.942 | 3.311 | 0.599 | 1.285 | 25.097 | 0.566 |
| | 6 | AIPW | -2.401 | -2.400 | 0.130 | -0.018 | 1.032 | 0.001 | 0.939 | 3.375 | 0.946 | 3.208 | 0.948 | 3.146 | 0.500 | 1.146 | 16.846 | 0.351 |
| | 6 | WAIPW | -2.401 | -2.398 | 0.128 | -0.006 | 1.042 | 0.003 | 0.937 | 3.436 | 0.945 | 3.213 | 0.947 | 3.174 | 0.489 | 0.895 | 16.499 | 0.337 |
| WIPW(1) | 6 | IPW | -2.401 | -2.401 | 0.182 | -0.076 | 1.047 | 0.000 | 0.939 | 3.372 | 0.947 | 3.178 | 0.941 | 3.342 | 0.653 | 2.587 | 32.946 | 0.936 |
| | 6 | WIPW | -2.401 | -2.391 | 0.162 | -0.011 | 1.033 | 0.010 | 0.941 | 3.328 | 0.941 | 3.320 | 0.948 | 3.140 | 0.608 | 1.549 | 26.368 | 0.615 |
| | 6 | AIPW | -2.401 | -2.401 | 0.144 | -0.031 | 1.046 | 0.000 | 0.938 | 3.420 | 0.944 | 3.252 | 0.941 | 3.342 | 0.525 | 2.253 | 20.749 | 0.624 |
| | 6 | WAIPW | -2.401 | -2.398 | 0.134 | -0.013 | 1.051 | 0.003 | 0.936 | 3.453 | 0.944 | 3.248 | 0.941 | 3.324 | 0.498 | 1.181 | 17.987 | 0.416 |
| AIPW(0.25) | 7 | IPW | -2.494 | -2.497 | 0.154 | -0.030 | 1.022 | -0.003 | 0.942 | 3.317 | 0.950 | 3.094 | 0.943 | 3.284 | 0.593 | 0.783 | 23.577 | 0.486 |
| | 7 | WIPW | -2.494 | -2.495 | 0.194 | -0.012 | 1.035 | -0.001 | 0.938 | 3.421 | 0.944 | 3.257 | 0.942 | 3.301 | 0.747 | 1.553 | 37.578 | 0.755 |
| | 7 | AIPW | -2.494 | -2.496 | 0.124 | -0.021 | 1.025 | -0.002 | 0.943 | 3.274 | 0.949 | 3.123 | 0.944 | 3.263 | 0.481 | 0.523 | 15.479 | 0.313 |
| | 7 | WAIPW | -2.494 | -2.496 | 0.125 | -0.026 | 1.027 | -0.002 | 0.942 | 3.295 | 0.949 | 3.106 | 0.943 | 3.284 | 0.482 | 0.522 | 15.622 | 0.317 |
| AIPW(0.5) | 7 | IPW | -2.494 | -2.494 | 0.153 | -0.019 | 1.032 | 0.000 | 0.943 | 3.286 | 0.949 | 3.105 | 0.944 | 3.239 | 0.581 | 0.857 | 23.258 | 0.499 |
| | 7 | WIPW | -2.494 | -2.495 | 0.182 | -0.033 | 1.039 | -0.001 | 0.942 | 3.293 | 0.953 | 2.994 | 0.936 | 3.450 | 0.689 | 1.938 | 33.227 | 0.744 |
| | 7 | AIPW | -2.494 | -2.495 | 0.122 | -0.019 | 1.018 | -0.001 | 0.946 | 3.183 | 0.949 | 3.098 | 0.945 | 3.218 | 0.472 | 0.563 | 14.809 | 0.295 |
| | 7 | WAIPW | -2.494 | -2.494 | 0.122 | -0.017 | 1.028 | 0.000 | 0.947 | 3.169 | 0.949 | 3.119 | 0.940 | 3.346 | 0.467 | 0.578 | 14.804 | 0.300 |
| AIPW(0.75) | 7 | IPW | -2.494 | -2.495 | 0.150 | -0.032 | 1.012 | -0.001 | 0.943 | 3.268 | 0.954 | 2.957 | 0.942 | 3.311 | 0.581 | 1.129 | 22.552 | 0.491 |
| | 7 | WIPW | -2.494 | -2.491 | 0.181 | -0.026 | 1.043 | 0.003 | 0.937 | 3.426 | 0.948 | 3.140 | 0.938 | 3.421 | 0.671 | 2.596 | 32.605 | 0.759 |
| | 7 | AIPW | -2.494 | -2.492 | 0.124 | -0.005 | 1.028 | 0.002 | 0.941 | 3.333 | 0.948 | 3.135 | 0.945 | 3.213 | 0.472 | 0.719 | 15.264 | 0.328 |
| | 7 | WAIPW | -2.494 | -2.490 | 0.122 | 0.007 | 1.039 | 0.004 | 0.940 | 3.348 | 0.944 | 3.246 | 0.945 | 3.213 | 0.462 | 0.705 | 15.003 | 0.322 |
| AIPW(1) | 7 | IPW | -2.494 | -2.493 | 0.159 | -0.033 | 1.036 | 0.001 | 0.945 | 3.215 | 0.947 | 3.174 | 0.946 | 3.206 | 0.594 | 1.840 | 25.142 | 0.606 |
| | 7 | WIPW | -2.494 | -2.487 | 0.196 | -0.029 | 1.084 | 0.007 | 0.929 | 3.625 | 0.945 | 3.220 | 0.930 | 3.597 | 0.678 | 3.696 | 38.477 | 1.107 |
| | 7 | AIPW | -2.494 | -2.489 | 0.127 | 0.015 | 1.031 | 0.005 | 0.944 | 3.261 | 0.942 | 3.315 | 0.952 | 3.024 | 0.479 | 1.272 | 16.116 | 0.379 |
| | 7 | WAIPW | -2.494 | -2.487 | 0.125 | 0.029 | 1.037 | 0.007 | 0.941 | 3.320 | 0.936 | 3.462 | 0.953 | 2.978 | 0.466 | 1.157 | 15.574 | 0.359 |
| AR-1 | 7 | IPW | -2.494 | -2.489 | 0.151 | 0.043 | 1.002 | 0.005 | 0.947 | 3.169 | 0.946 | 3.186 | 0.953 | 3.005 | 0.595 | 1.043 | 22.915 | 0.487 |

| Randomization Method | Regime | Estimator | True Value | Mean Estimate | SD Estimates | Mean Normalized | SD Normalized | Mean Bias | CI Coverage | SE CI Coverage x 10^3 | LB Coverage | SE LB Coverage x 10^3 | UB Coverage | SE UB Coverage x 10^3 | Mean CI Length | SE CI Length x 10^3 | MSE x 10^3 | SE MSE x 10^3 |
|---|---|---|---|---|---|---|---|---|---|---|---|---|---|---|---|---|---|---|
| | 7 | WIPW | -2.494 | -2.492 | 0.148 | 0.023 | 1.002 | 0.002 | 0.947 | 3.169 | 0.950 | 3.094 | 0.952 | 3.035 | 0.580 | 1.027 | 21.873 | 0.469 |
| | 7 | AIPW | -2.494 | -2.493 | 0.122 | 0.020 | 1.004 | 0.001 | 0.951 | 3.059 | 0.948 | 3.152 | 0.951 | 3.059 | 0.481 | 0.680 | 14.930 | 0.303 |
| | 7 | WAIPW | -2.494 | -2.494 | 0.123 | 0.012 | 1.011 | 0.000 | 0.950 | 3.094 | 0.949 | 3.106 | 0.950 | 3.077 | 0.478 | 0.837 | 15.042 | 0.310 |
| AR-2 | 7 | IPW | -2.494 | -2.488 | 0.169 | 0.066 | 1.032 | 0.006 | 0.939 | 3.395 | 0.936 | 3.472 | 0.953 | 3.005 | 0.637 | 1.790 | 28.488 | 0.639 |
| | 7 | WIPW | -2.494 | -2.494 | 0.162 | 0.025 | 1.027 | 0.000 | 0.945 | 3.219 | 0.944 | 3.263 | 0.954 | 2.957 | 0.614 | 1.512 | 26.128 | 0.574 |
| | 7 | AIPW | -2.494 | -2.493 | 0.135 | 0.038 | 1.039 | 0.001 | 0.945 | 3.230 | 0.941 | 3.338 | 0.944 | 3.263 | 0.509 | 1.278 | 18.219 | 0.403 |
| | 7 | WAIPW | -2.494 | -2.495 | 0.134 | 0.018 | 1.041 | -0.001 | 0.939 | 3.385 | 0.944 | 3.252 | 0.940 | 3.354 | 0.502 | 1.210 | 17.840 | 0.389 |
| IAIPW(0.25) | 7 | IPW | -2.494 | -2.498 | 0.152 | -0.036 | 1.015 | -0.004 | 0.946 | 3.186 | 0.950 | 3.077 | 0.948 | 3.140 | 0.592 | 0.785 | 23.091 | 0.465 |
| | 7 | WIPW | -2.494 | -2.496 | 0.151 | -0.029 | 1.015 | -0.002 | 0.947 | 3.174 | 0.949 | 3.106 | 0.949 | 3.123 | 0.586 | 0.780 | 22.673 | 0.458 |
| | 7 | AIPW | -2.494 | -2.496 | 0.125 | -0.028 | 1.031 | -0.002 | 0.941 | 3.327 | 0.945 | 3.224 | 0.941 | 3.322 | 0.481 | 0.523 | 15.750 | 0.319 |
| | 7 | WAIPW | -2.494 | -2.497 | 0.126 | -0.032 | 1.035 | -0.003 | 0.939 | 3.390 | 0.945 | 3.224 | 0.941 | 3.327 | 0.482 | 0.526 | 15.936 | 0.323 |
| IAIPW(0.5) | 7 | IPW | -2.494 | -2.491 | 0.150 | 0.001 | 1.022 | 0.003 | 0.947 | 3.176 | 0.944 | 3.259 | 0.948 | 3.134 | 0.580 | 0.872 | 22.612 | 0.469 |
| | 7 | WIPW | -2.494 | -2.488 | 0.147 | 0.014 | 1.024 | 0.006 | 0.947 | 3.169 | 0.941 | 3.326 | 0.949 | 3.112 | 0.565 | 0.891 | 21.531 | 0.451 |
| | 7 | AIPW | -2.494 | -2.490 | 0.123 | 0.022 | 1.028 | 0.004 | 0.945 | 3.218 | 0.945 | 3.232 | 0.949 | 3.105 | 0.472 | 0.585 | 15.259 | 0.316 |
| | 7 | WAIPW | -2.494 | -2.489 | 0.123 | 0.018 | 1.034 | 0.005 | 0.941 | 3.333 | 0.946 | 3.204 | 0.947 | 3.176 | 0.467 | 0.608 | 15.123 | 0.311 |
| IAIPW(0.75) | 7 | IPW | -2.494 | -2.496 | 0.153 | -0.039 | 1.025 | -0.002 | 0.943 | 3.279 | 0.947 | 3.157 | 0.944 | 3.263 | 0.582 | 1.169 | 23.316 | 0.516 |
| | 7 | WIPW | -2.494 | -2.491 | 0.147 | -0.017 | 1.026 | 0.003 | 0.941 | 3.327 | 0.948 | 3.140 | 0.944 | 3.252 | 0.559 | 1.156 | 21.680 | 0.479 |
| | 7 | AIPW | -2.494 | -2.493 | 0.122 | -0.014 | 1.017 | 0.001 | 0.944 | 3.241 | 0.947 | 3.180 | 0.946 | 3.202 | 0.471 | 0.767 | 14.965 | 0.318 |
| | 7 | WAIPW | -2.494 | -2.492 | 0.122 | -0.007 | 1.029 | 0.002 | 0.939 | 3.390 | 0.945 | 3.219 | 0.945 | 3.219 | 0.463 | 0.786 | 14.822 | 0.314 |
| IAIPW(1) | 7 | IPW | -2.494 | -2.494 | 0.155 | -0.040 | 1.007 | 0.000 | 0.947 | 3.155 | 0.954 | 2.973 | 0.945 | 3.225 | 0.590 | 1.720 | 24.103 | 0.613 |
| | 7 | WIPW | -2.494 | -2.488 | 0.146 | -0.005 | 1.010 | 0.006 | 0.948 | 3.145 | 0.951 | 3.063 | 0.945 | 3.220 | 0.560 | 1.393 | 21.295 | 0.469 |
| | 7 | AIPW | -2.494 | -2.491 | 0.125 | 0.002 | 1.014 | 0.003 | 0.945 | 3.211 | 0.945 | 3.211 | 0.953 | 2.994 | 0.478 | 1.170 | 15.633 | 0.373 |
| | 7 | WAIPW | -2.494 | -2.488 | 0.123 | 0.019 | 1.028 | 0.006 | 0.945 | 3.220 | 0.941 | 3.337 | 0.951 | 3.058 | 0.464 | 1.082 | 15.067 | 0.339 |
| IPW(0.25) | 7 | IPW | -2.494 | -2.494 | 0.154 | -0.012 | 1.022 | 0.001 | 0.945 | 3.230 | 0.951 | 3.065 | 0.946 | 3.186 | 0.595 | 0.779 | 23.661 | 0.479 |
| | 7 | WIPW | -2.494 | -2.492 | 0.195 | -0.003 | 1.033 | 0.002 | 0.941 | 3.338 | 0.946 | 3.191 | 0.943 | 3.290 | 0.751 | 1.553 | 38.006 | 0.786 |
| | 7 | AIPW | -2.494 | -2.493 | 0.125 | -0.002 | 1.022 | 0.001 | 0.949 | 3.117 | 0.945 | 3.224 | 0.948 | 3.129 | 0.482 | 0.523 | 15.600 | 0.314 |
| | 7 | WAIPW | -2.494 | -2.494 | 0.125 | -0.007 | 1.023 | 0.000 | 0.949 | 3.100 | 0.946 | 3.208 | 0.946 | 3.208 | 0.483 | 0.526 | 15.660 | 0.316 |
| IPW(0.5) | 7 | IPW | -2.494 | -2.488 | 0.150 | 0.012 | 1.007 | 0.006 | 0.952 | 3.031 | 0.943 | 3.286 | 0.953 | 2.986 | 0.583 | 0.869 | 22.580 | 0.466 |
| | 7 | WIPW | -2.494 | -2.489 | 0.183 | 0.002 | 1.023 | 0.005 | 0.945 | 3.211 | 0.950 | 3.083 | 0.946 | 3.204 | 0.699 | 1.982 | 33.485 | 0.734 |
| | 7 | AIPW | -2.494 | -2.487 | 0.122 | 0.048 | 1.013 | 0.007 | 0.948 | 3.126 | 0.942 | 3.300 | 0.955 | 2.925 | 0.474 | 0.586 | 14.977 | 0.304 |
| | 7 | WAIPW | -2.494 | -2.487 | 0.122 | 0.043 | 1.021 | 0.007 | 0.946 | 3.183 | 0.943 | 3.266 | 0.955 | 2.917 | 0.469 | 0.585 | 14.859 | 0.304 |
| IPW(0.75) | 7 | IPW | -2.494 | -2.494 | 0.155 | -0.044 | 1.027 | 0.000 | 0.943 | 3.274 | 0.950 | 3.077 | 0.941 | 3.322 | 0.585 | 1.184 | 24.092 | 0.549 |
| | 7 | WIPW | -2.494 | -2.488 | 0.192 | -0.015 | 1.065 | 0.006 | 0.937 | 3.426 | 0.946 | 3.202 | 0.939 | 3.380 | 0.689 | 2.793 | 36.814 | 0.917 |
| | 7 | AIPW | -2.494 | -2.495 | 0.125 | -0.019 | 1.041 | -0.001 | 0.943 | 3.274 | 0.946 | 3.197 | 0.942 | 3.306 | 0.474 | 0.769 | 15.698 | 0.330 |
| | 7 | WAIPW | -2.494 | -2.492 | 0.124 | -0.007 | 1.052 | 0.002 | 0.939 | 3.385 | 0.942 | 3.306 | 0.942 | 3.317 | 0.465 | 0.710 | 15.368 | 0.319 |
| IPW(1) | 7 | IPW | -2.494 | -2.496 | 0.159 | -0.069 | 0.994 | -0.002 | 0.949 | 3.107 | 0.956 | 2.911 | 0.941 | 3.320 | 0.597 | 1.729 | 25.197 | 1.004 |
| | 7 | WIPW | -2.494 | -2.488 | 0.200 | -0.036 | 1.066 | 0.006 | 0.931 | 3.577 | 0.946 | 3.188 | 0.933 | 3.545 | 0.688 | 3.491 | 39.986 | 1.701 |
| | 7 | AIPW | -2.494 | -2.495 | 0.127 | -0.030 | 1.001 | -0.001 | 0.950 | 3.078 | 0.957 | 2.880 | 0.950 | 3.088 | 0.484 | 1.347 | 16.033 | 0.604 |
| | 7 | WAIPW | -2.494 | -2.493 | 0.122 | -0.018 | 1.014 | 0.001 | 0.949 | 3.097 | 0.952 | 3.019 | 0.947 | 3.169 | 0.468 | 0.967 | 14.927 | 0.352 |
| SR | 7 | IPW | -2.494 | -2.492 | 0.162 | 0.010 | 1.028 | 0.002 | 0.940 | 3.349 | 0.944 | 3.258 | 0.946 | 3.191 | 0.624 | 0.827 | 26.250 | 0.545 |
| | 7 | WIPW | -2.494 | -2.494 | 0.225 | 0.001 | 1.060 | 0.000 | 0.934 | 3.507 | 0.940 | 3.359 | 0.941 | 3.322 | 0.851 | 1.770 | 50.734 | 1.016 |
| | 7 | AIPW | -2.494 | -2.493 | 0.131 | 0.004 | 1.032 | 0.001 | 0.940 | 3.364 | 0.944 | 3.252 | 0.944 | 3.258 | 0.502 | 0.545 | 17.107 | 0.353 |
| | 7 | WAIPW | -2.494 | -2.494 | 0.132 | -0.005 | 1.033 | 0.000 | 0.935 | 3.482 | 0.945 | 3.236 | 0.943 | 3.290 | 0.507 | 0.553 | 17.501 | 0.361 |
| TS(0.25) | 7 | IPW | -2.494 | -2.485 | 0.145 | 0.044 | 0.986 | 0.009 | 0.950 | 3.071 | 0.947 | 3.163 | 0.959 | 2.811 | 0.577 | 0.925 | 21.141 | 0.445 |
| | 7 | WIPW | -2.494 | -2.483 | 0.141 | 0.056 | 0.985 | 0.011 | 0.952 | 3.035 | 0.946 | 3.191 | 0.961 | 2.752 | 0.561 | 0.892 | 19.963 | 0.417 |

| Randomization Method | Regime | Estimator | True Value | Mean Estimate | SD Estimates | Mean Normalized | SD Normalized | Mean Bias | CI Coverage | SE CI Coverage x 10^3 | LB Coverage | SE LB Coverage x 10^3 | UB Coverage | SE UB Coverage x 10^3 | Mean CI Length | SE CI Length x 10^3 | MSE x 10^3 | SE MSE x 10^3 |
|---|---|---|---|---|---|---|---|---|---|---|---|---|---|---|---|---|---|---|
| | 7 | AIPW | -2.494 | -2.487 | 0.119 | 0.046 | 0.996 | 0.007 | 0.948 | 3.146 | 0.940 | 3.354 | 0.956 | 2.888 | 0.471 | 0.737 | 14.321 | 0.362 |
| | 7 | WAIPW | -2.494 | -2.486 | 0.118 | 0.051 | 1.002 | 0.008 | 0.947 | 3.180 | 0.939 | 3.375 | 0.957 | 2.863 | 0.465 | 0.603 | 14.045 | 0.303 |
| TS(0.50) | 7 | IPW | -2.494 | -2.489 | 0.149 | 0.008 | 1.013 | 0.005 | 0.950 | 3.088 | 0.947 | 3.157 | 0.950 | 3.077 | 0.578 | 1.119 | 22.322 | 0.486 |
| | 7 | WIPW | -2.494 | -2.485 | 0.144 | 0.030 | 1.015 | 0.009 | 0.947 | 3.169 | 0.945 | 3.213 | 0.951 | 3.059 | 0.556 | 1.094 | 20.952 | 0.456 |
| | 7 | AIPW | -2.494 | -2.490 | 0.123 | 0.014 | 1.015 | 0.004 | 0.943 | 3.284 | 0.947 | 3.169 | 0.953 | 2.981 | 0.473 | 1.051 | 15.166 | 0.408 |
| | 7 | WAIPW | -2.494 | -2.488 | 0.122 | 0.025 | 1.023 | 0.006 | 0.940 | 3.354 | 0.944 | 3.246 | 0.952 | 3.017 | 0.465 | 1.166 | 14.996 | 0.474 |
| TS(0.75) | 7 | IPW | -2.494 | -2.490 | 0.156 | -0.015 | 1.021 | 0.004 | 0.944 | 3.263 | 0.948 | 3.152 | 0.949 | 3.106 | 0.589 | 1.719 | 24.429 | 0.600 |
| | 7 | WIPW | -2.494 | -2.484 | 0.148 | 0.021 | 1.023 | 0.010 | 0.947 | 3.180 | 0.943 | 3.268 | 0.952 | 3.029 | 0.562 | 1.461 | 22.117 | 0.486 |
| | 7 | AIPW | -2.494 | -2.491 | 0.127 | -0.005 | 1.028 | 0.003 | 0.947 | 3.163 | 0.944 | 3.246 | 0.947 | 3.163 | 0.479 | 1.582 | 16.226 | 0.417 |
| | 7 | WAIPW | -2.494 | -2.489 | 0.133 | 0.014 | 1.043 | 0.005 | 0.940 | 3.348 | 0.938 | 3.401 | 0.948 | 3.140 | 0.469 | 2.657 | 17.740 | 1.796 |
| TS(1) | 7 | IPW | -2.494 | -2.489 | 0.170 | -0.024 | 1.051 | 0.005 | 0.941 | 3.338 | 0.945 | 3.235 | 0.944 | 3.241 | 0.606 | 2.483 | 28.798 | 0.868 |
| | 7 | WIPW | -2.494 | -2.481 | 0.156 | 0.021 | 1.041 | 0.013 | 0.940 | 3.369 | 0.941 | 3.322 | 0.947 | 3.180 | 0.574 | 1.873 | 24.577 | 0.596 |
| | 7 | AIPW | -2.494 | -2.487 | 0.136 | 0.029 | 1.047 | 0.007 | 0.941 | 3.333 | 0.938 | 3.406 | 0.948 | 3.135 | 0.493 | 2.449 | 18.527 | 0.690 |
| | 7 | WAIPW | -2.494 | -2.484 | 0.136 | 0.055 | 1.049 | 0.010 | 0.937 | 3.431 | 0.936 | 3.462 | 0.951 | 3.065 | 0.476 | 2.177 | 18.708 | 1.637 |
| WAIPW(0.25) | 7 | IPW | -2.494 | -2.499 | 0.153 | -0.045 | 1.016 | -0.005 | 0.947 | 3.163 | 0.951 | 3.059 | 0.945 | 3.235 | 0.593 | 0.778 | 23.405 | 0.461 |
| | 7 | WIPW | -2.494 | -2.498 | 0.151 | -0.038 | 1.016 | -0.004 | 0.946 | 3.191 | 0.951 | 3.065 | 0.944 | 3.246 | 0.587 | 0.773 | 22.908 | 0.452 |
| | 7 | AIPW | -2.494 | -2.498 | 0.124 | -0.039 | 1.021 | -0.004 | 0.949 | 3.123 | 0.951 | 3.059 | 0.941 | 3.327 | 0.481 | 0.529 | 15.422 | 0.301 |
| | 7 | WAIPW | -2.494 | -2.498 | 0.125 | -0.045 | 1.028 | -0.004 | 0.944 | 3.246 | 0.951 | 3.053 | 0.941 | 3.343 | 0.481 | 0.532 | 15.680 | 0.307 |
| WAIPW(0.5) | 7 | IPW | -2.494 | -2.491 | 0.150 | 0.004 | 1.018 | 0.003 | 0.948 | 3.126 | 0.950 | 3.090 | 0.946 | 3.197 | 0.578 | 0.847 | 22.421 | 0.476 |
| | 7 | WIPW | -2.494 | -2.488 | 0.147 | 0.022 | 1.025 | 0.006 | 0.945 | 3.225 | 0.945 | 3.232 | 0.947 | 3.155 | 0.562 | 0.864 | 21.586 | 0.465 |
| | 7 | AIPW | -2.494 | -2.491 | 0.123 | 0.008 | 1.022 | 0.003 | 0.945 | 3.225 | 0.944 | 3.252 | 0.949 | 3.105 | 0.471 | 0.559 | 15.091 | 0.313 |
| | 7 | WAIPW | -2.494 | -2.491 | 0.123 | 0.009 | 1.031 | 0.003 | 0.940 | 3.353 | 0.940 | 3.353 | 0.949 | 3.098 | 0.467 | 0.566 | 15.020 | 0.316 |
| WAIPW(0.75) | 7 | IPW | -2.494 | -2.489 | 0.151 | 0.006 | 1.015 | 0.005 | 0.949 | 3.112 | 0.947 | 3.157 | 0.945 | 3.219 | 0.584 | 1.190 | 22.891 | 0.502 |
| | 7 | WIPW | -2.494 | -2.485 | 0.146 | 0.026 | 1.021 | 0.009 | 0.947 | 3.174 | 0.941 | 3.322 | 0.949 | 3.112 | 0.559 | 1.135 | 21.474 | 0.468 |
| | 7 | AIPW | -2.494 | -2.490 | 0.123 | 0.012 | 1.023 | 0.004 | 0.944 | 3.263 | 0.948 | 3.146 | 0.945 | 3.230 | 0.473 | 0.753 | 15.231 | 0.336 |
| | 7 | WAIPW | -2.494 | -2.488 | 0.123 | 0.021 | 1.034 | 0.006 | 0.942 | 3.301 | 0.948 | 3.146 | 0.946 | 3.197 | 0.464 | 0.768 | 15.063 | 0.327 |
| WAIPW(1) | 7 | IPW | -2.494 | -2.492 | 0.156 | -0.026 | 1.026 | 0.002 | 0.940 | 3.355 | 0.951 | 3.063 | 0.942 | 3.306 | 0.591 | 1.617 | 24.387 | 0.585 |
| | 7 | WIPW | -2.494 | -2.487 | 0.147 | 0.001 | 1.023 | 0.007 | 0.940 | 3.350 | 0.947 | 3.164 | 0.946 | 3.206 | 0.560 | 1.351 | 21.682 | 0.473 |
| | 7 | AIPW | -2.494 | -2.490 | 0.127 | 0.007 | 1.026 | 0.004 | 0.947 | 3.174 | 0.949 | 3.121 | 0.949 | 3.097 | 0.478 | 1.109 | 16.037 | 0.356 |
| | 7 | WAIPW | -2.494 | -2.489 | 0.126 | 0.017 | 1.039 | 0.005 | 0.942 | 3.311 | 0.945 | 3.229 | 0.946 | 3.202 | 0.466 | 1.223 | 15.851 | 0.399 |
| WIPW(0.25) | 7 | IPW | -2.494 | -2.490 | 0.155 | 0.009 | 1.030 | 0.004 | 0.944 | 3.252 | 0.945 | 3.235 | 0.944 | 3.263 | 0.594 | 0.800 | 23.950 | 0.479 |
| | 7 | WIPW | -2.494 | -2.488 | 0.154 | 0.021 | 1.033 | 0.006 | 0.945 | 3.235 | 0.945 | 3.230 | 0.944 | 3.263 | 0.589 | 0.793 | 23.712 | 0.475 |
| | 7 | AIPW | -2.494 | -2.491 | 0.125 | 0.016 | 1.028 | 0.003 | 0.943 | 3.290 | 0.940 | 3.364 | 0.948 | 3.135 | 0.482 | 0.515 | 15.618 | 0.319 |
| | 7 | WAIPW | -2.494 | -2.492 | 0.126 | 0.010 | 1.032 | 0.002 | 0.943 | 3.284 | 0.943 | 3.290 | 0.946 | 3.208 | 0.483 | 0.515 | 15.781 | 0.323 |
| WIPW(0.5) | 7 | IPW | -2.494 | -2.497 | 0.149 | -0.045 | 1.005 | -0.003 | 0.951 | 3.039 | 0.956 | 2.901 | 0.945 | 3.232 | 0.583 | 0.866 | 22.324 | 0.451 |
| | 7 | WIPW | -2.494 | -2.492 | 0.147 | -0.021 | 1.012 | 0.002 | 0.949 | 3.119 | 0.950 | 3.068 | 0.944 | 3.246 | 0.568 | 0.876 | 21.490 | 0.434 |
| | 7 | AIPW | -2.494 | -2.496 | 0.124 | -0.023 | 1.032 | -0.002 | 0.946 | 3.183 | 0.948 | 3.141 | 0.943 | 3.266 | 0.473 | 0.577 | 15.358 | 0.305 |
| | 7 | WAIPW | -2.494 | -2.495 | 0.123 | -0.024 | 1.037 | -0.001 | 0.943 | 3.280 | 0.946 | 3.204 | 0.946 | 3.190 | 0.469 | 0.577 | 15.162 | 0.303 |
| WIPW(0.75) | 7 | IPW | -2.494 | -2.493 | 0.155 | -0.037 | 1.030 | 0.001 | 0.945 | 3.235 | 0.951 | 3.065 | 0.943 | 3.268 | 0.586 | 1.164 | 24.078 | 0.526 |
| | 7 | WIPW | -2.494 | -2.486 | 0.151 | 0.002 | 1.040 | 0.008 | 0.942 | 3.311 | 0.945 | 3.230 | 0.948 | 3.129 | 0.562 | 1.078 | 22.742 | 0.503 |
| | 7 | AIPW | -2.494 | -2.495 | 0.125 | -0.023 | 1.036 | -0.001 | 0.944 | 3.246 | 0.946 | 3.197 | 0.943 | 3.274 | 0.475 | 0.799 | 15.667 | 0.320 |
| | 7 | WAIPW | -2.494 | -2.493 | 0.124 | -0.016 | 1.043 | 0.001 | 0.939 | 3.390 | 0.945 | 3.219 | 0.938 | 3.411 | 0.465 | 0.725 | 15.272 | 0.316 |
| WIPW(1) | 7 | IPW | -2.494 | -2.490 | 0.159 | -0.031 | 1.016 | 0.004 | 0.945 | 3.225 | 0.949 | 3.102 | 0.944 | 3.243 | 0.597 | 1.715 | 25.229 | 0.638 |
| | 7 | WIPW | -2.494 | -2.481 | 0.151 | 0.018 | 1.022 | 0.013 | 0.944 | 3.238 | 0.945 | 3.225 | 0.948 | 3.126 | 0.566 | 1.348 | 22.859 | 0.532 |
| | 7 | AIPW | -2.494 | -2.490 | 0.128 | 0.010 | 1.027 | 0.004 | 0.941 | 3.337 | 0.942 | 3.315 | 0.949 | 3.112 | 0.484 | 1.343 | 16.514 | 0.398 |

| Randomization Method | Regime | Estimator | True Value | Mean Estimate | SD Estimates | Mean Normalized | SD Normalized | Mean Bias | CI Coverage | SE CI Coverage x 10^3 | LB Coverage | SE LB Coverage x 10^3 | UB Coverage | SE UB Coverage x 10^3 | Mean CI Length | SE CI Length x 10^3 | MSE x 10^3 | SE MSE x 10^3 |
|---|---|---|---|---|---|---|---|---|---|---|---|---|---|---|---|---|---|---|
| | 7 | WAIPW | -2.494 | -2.488 | 0.126 | 0.020 | 1.042 | 0.006 | 0.939 | 3.390 | 0.938 | 3.407 | 0.948 | 3.150 | 0.468 | 0.949 | 15.881 | 0.358 |
| AIPW(0.25) | 8 | IPW | -2.501 | -2.504 | 0.150 | -0.031 | 1.007 | -0.003 | 0.946 | 3.197 | 0.949 | 3.117 | 0.946 | 3.202 | 0.589 | 0.774 | 22.614 | 0.458 |
| | 8 | WIPW | -2.501 | -2.502 | 0.191 | -0.011 | 1.028 | -0.001 | 0.944 | 3.246 | 0.944 | 3.257 | 0.947 | 3.180 | 0.740 | 1.529 | 36.447 | 0.725 |
| | 8 | AIPW | -2.501 | -2.504 | 0.123 | -0.035 | 1.015 | -0.003 | 0.946 | 3.202 | 0.954 | 2.975 | 0.945 | 3.213 | 0.479 | 0.517 | 15.123 | 0.301 |
| | 8 | WAIPW | -2.501 | -2.503 | 0.123 | -0.030 | 1.018 | -0.003 | 0.948 | 3.140 | 0.953 | 3.005 | 0.946 | 3.191 | 0.479 | 0.509 | 15.186 | 0.304 |
| AIPW(0.5) | 8 | IPW | -2.501 | -2.500 | 0.148 | -0.012 | 1.012 | 0.001 | 0.948 | 3.134 | 0.949 | 3.098 | 0.944 | 3.252 | 0.576 | 0.841 | 21.907 | 0.461 |
| | 8 | WIPW | -2.501 | -2.500 | 0.178 | -0.015 | 1.028 | 0.001 | 0.944 | 3.239 | 0.947 | 3.169 | 0.945 | 3.218 | 0.682 | 1.873 | 31.762 | 0.692 |
| | 8 | AIPW | -2.501 | -2.502 | 0.120 | -0.028 | 1.004 | -0.002 | 0.951 | 3.061 | 0.956 | 2.901 | 0.945 | 3.211 | 0.470 | 0.564 | 14.301 | 0.283 |
| | 8 | WAIPW | -2.501 | -2.500 | 0.119 | -0.015 | 1.014 | 0.000 | 0.951 | 3.054 | 0.951 | 3.054 | 0.947 | 3.176 | 0.463 | 0.556 | 14.186 | 0.285 |
| AIPW(0.75) | 8 | IPW | -2.501 | -2.502 | 0.150 | -0.037 | 1.012 | -0.001 | 0.946 | 3.208 | 0.954 | 2.975 | 0.939 | 3.375 | 0.578 | 1.130 | 22.358 | 0.480 |
| | 8 | WIPW | -2.501 | -2.498 | 0.180 | -0.016 | 1.046 | 0.003 | 0.934 | 3.507 | 0.943 | 3.284 | 0.941 | 3.338 | 0.664 | 2.577 | 32.332 | 0.785 |
| | 8 | AIPW | -2.501 | -2.499 | 0.123 | -0.008 | 1.021 | 0.002 | 0.947 | 3.174 | 0.949 | 3.117 | 0.945 | 3.230 | 0.471 | 0.718 | 15.071 | 0.323 |
| | 8 | WAIPW | -2.501 | -2.495 | 0.121 | 0.019 | 1.029 | 0.006 | 0.945 | 3.235 | 0.945 | 3.219 | 0.946 | 3.191 | 0.459 | 0.668 | 14.604 | 0.312 |
| AIPW(1) | 8 | IPW | -2.501 | -2.499 | 0.156 | -0.029 | 1.036 | 0.001 | 0.943 | 3.284 | 0.944 | 3.248 | 0.946 | 3.188 | 0.586 | 1.708 | 24.467 | 0.583 |
| | 8 | WIPW | -2.501 | -2.494 | 0.189 | -0.011 | 1.072 | 0.007 | 0.936 | 3.453 | 0.942 | 3.311 | 0.945 | 3.225 | 0.664 | 3.431 | 35.704 | 1.006 |
| | 8 | AIPW | -2.501 | -2.498 | 0.126 | -0.002 | 1.031 | 0.003 | 0.944 | 3.257 | 0.945 | 3.234 | 0.948 | 3.140 | 0.477 | 1.214 | 15.981 | 0.374 |
| | 8 | WAIPW | -2.501 | -2.493 | 0.123 | 0.028 | 1.037 | 0.008 | 0.942 | 3.315 | 0.939 | 3.377 | 0.949 | 3.107 | 0.459 | 0.889 | 15.155 | 0.347 |
| AR-1 | 8 | IPW | -2.501 | -2.501 | 0.153 | 0.010 | 1.017 | -0.001 | 0.943 | 3.290 | 0.949 | 3.100 | 0.948 | 3.129 | 0.593 | 1.066 | 23.555 | 0.485 |
| | 8 | WIPW | -2.501 | -2.505 | 0.150 | -0.009 | 1.018 | -0.004 | 0.945 | 3.230 | 0.949 | 3.100 | 0.947 | 3.169 | 0.578 | 0.997 | 22.504 | 0.466 |
| | 8 | AIPW | -2.501 | -2.499 | 0.122 | 0.031 | 1.006 | 0.002 | 0.950 | 3.071 | 0.944 | 3.263 | 0.952 | 3.011 | 0.480 | 0.660 | 14.974 | 0.302 |
| | 8 | WAIPW | -2.501 | -2.502 | 0.122 | 0.012 | 1.014 | -0.001 | 0.946 | 3.197 | 0.944 | 3.241 | 0.949 | 3.106 | 0.474 | 0.631 | 14.832 | 0.300 |
| AR-2 | 8 | IPW | -2.501 | -2.498 | 0.172 | 0.044 | 1.046 | 0.002 | 0.940 | 3.369 | 0.939 | 3.395 | 0.944 | 3.257 | 0.641 | 1.942 | 29.626 | 0.680 |
| | 8 | WIPW | -2.501 | -2.505 | 0.164 | 0.007 | 1.041 | -0.004 | 0.939 | 3.385 | 0.943 | 3.279 | 0.944 | 3.252 | 0.616 | 1.558 | 26.897 | 0.596 |
| | 8 | AIPW | -2.501 | -2.499 | 0.136 | 0.056 | 1.041 | 0.002 | 0.939 | 3.375 | 0.939 | 3.385 | 0.948 | 3.140 | 0.511 | 1.367 | 18.487 | 0.414 |
| | 8 | WAIPW | -2.501 | -2.503 | 0.133 | 0.023 | 1.048 | -0.003 | 0.935 | 3.492 | 0.941 | 3.338 | 0.942 | 3.306 | 0.499 | 1.081 | 17.759 | 0.389 |
| IAIPW(0.25) | 8 | IPW | -2.501 | -2.503 | 0.153 | -0.023 | 1.023 | -0.002 | 0.944 | 3.263 | 0.950 | 3.088 | 0.945 | 3.230 | 0.589 | 0.768 | 23.312 | 0.468 |
| | 8 | WIPW | -2.501 | -2.501 | 0.151 | -0.014 | 1.022 | 0.000 | 0.944 | 3.263 | 0.949 | 3.123 | 0.946 | 3.202 | 0.583 | 0.762 | 22.833 | 0.460 |
| | 8 | AIPW | -2.501 | -2.503 | 0.125 | -0.024 | 1.031 | -0.002 | 0.938 | 3.401 | 0.949 | 3.106 | 0.937 | 3.426 | 0.480 | 0.523 | 15.671 | 0.322 |
| | 8 | WAIPW | -2.501 | -2.501 | 0.126 | -0.015 | 1.035 | -0.001 | 0.938 | 3.411 | 0.946 | 3.186 | 0.939 | 3.395 | 0.480 | 0.519 | 15.785 | 0.326 |
| IAIPW(0.5) | 8 | IPW | -2.501 | -2.496 | 0.149 | 0.017 | 1.014 | 0.005 | 0.950 | 3.090 | 0.942 | 3.300 | 0.951 | 3.054 | 0.578 | 0.872 | 22.204 | 0.471 |
| | 8 | WIPW | -2.501 | -2.493 | 0.145 | 0.032 | 1.016 | 0.008 | 0.950 | 3.083 | 0.939 | 3.379 | 0.952 | 3.024 | 0.562 | 0.888 | 21.201 | 0.449 |
| | 8 | AIPW | -2.501 | -2.495 | 0.123 | 0.034 | 1.022 | 0.006 | 0.949 | 3.112 | 0.943 | 3.273 | 0.950 | 3.090 | 0.471 | 0.590 | 15.053 | 0.308 |
| | 8 | WAIPW | -2.501 | -2.493 | 0.122 | 0.045 | 1.028 | 0.008 | 0.946 | 3.204 | 0.943 | 3.266 | 0.948 | 3.148 | 0.464 | 0.586 | 14.843 | 0.305 |
| IAIPW(0.75) | 8 | IPW | -2.501 | -2.502 | 0.150 | -0.033 | 1.026 | -0.001 | 0.946 | 3.191 | 0.947 | 3.157 | 0.944 | 3.252 | 0.575 | 1.084 | 22.631 | 0.478 |
| | 8 | WIPW | -2.501 | -2.497 | 0.145 | -0.007 | 1.029 | 0.004 | 0.943 | 3.290 | 0.943 | 3.268 | 0.947 | 3.174 | 0.550 | 1.055 | 20.937 | 0.441 |
| | 8 | AIPW | -2.501 | -2.501 | 0.121 | -0.020 | 1.010 | 0.000 | 0.945 | 3.224 | 0.948 | 3.129 | 0.944 | 3.246 | 0.470 | 0.757 | 14.615 | 0.301 |
| | 8 | WAIPW | -2.501 | -2.497 | 0.120 | 0.001 | 1.023 | 0.003 | 0.942 | 3.311 | 0.944 | 3.241 | 0.946 | 3.191 | 0.458 | 0.695 | 14.323 | 0.295 |
| IAIPW(1) | 8 | IPW | -2.501 | -2.502 | 0.154 | -0.047 | 1.006 | -0.001 | 0.948 | 3.150 | 0.950 | 3.092 | 0.944 | 3.257 | 0.584 | 1.653 | 23.739 | 0.592 |
| | 8 | WIPW | -2.501 | -2.496 | 0.144 | -0.018 | 1.001 | 0.005 | 0.947 | 3.155 | 0.948 | 3.145 | 0.949 | 3.097 | 0.550 | 1.324 | 20.635 | 0.470 |
| | 8 | AIPW | -2.501 | -2.501 | 0.124 | -0.026 | 1.007 | 0.000 | 0.947 | 3.164 | 0.947 | 3.159 | 0.948 | 3.150 | 0.477 | 1.194 | 15.373 | 0.367 |
| | 8 | WAIPW | -2.501 | -2.496 | 0.120 | 0.004 | 1.016 | 0.004 | 0.945 | 3.215 | 0.942 | 3.311 | 0.952 | 3.014 | 0.459 | 0.913 | 14.432 | 0.323 |
| IPW(0.25) | 8 | IPW | -2.501 | -2.500 | 0.153 | -0.008 | 1.021 | 0.001 | 0.945 | 3.235 | 0.947 | 3.163 | 0.944 | 3.241 | 0.592 | 0.767 | 23.445 | 0.482 |
| | 8 | WIPW | -2.501 | -2.497 | 0.194 | 0.009 | 1.032 | 0.004 | 0.937 | 3.436 | 0.944 | 3.257 | 0.944 | 3.257 | 0.746 | 1.532 | 37.556 | 0.775 |
| | 8 | AIPW | -2.501 | -2.500 | 0.125 | 0.001 | 1.026 | 0.001 | 0.948 | 3.135 | 0.950 | 3.077 | 0.942 | 3.306 | 0.482 | 0.525 | 15.620 | 0.311 |
| | 8 | WAIPW | -2.501 | -2.498 | 0.125 | 0.010 | 1.025 | 0.002 | 0.950 | 3.088 | 0.947 | 3.157 | 0.946 | 3.208 | 0.481 | 0.521 | 15.586 | 0.312 |

| Randomization Method | Regime | Estimator | True Value | Mean Estimate | SD Estimates | Mean Normalized | SD Normalized | Mean Bias | CI Coverage | SE CI Coverage x 10^3 | LB Coverage | SE LB Coverage x 10^3 | UB Coverage | SE UB Coverage x 10^3 | Mean CI Length | SE CI Length x 10^3 | MSE x 10^3 | SE MSE x 10^3 |
|---|---|---|---|---|---|---|---|---|---|---|---|---|---|---|---|---|---|---|
| IPW(0.5) | 8 | IPW | -2.501 | -2.495 | 0.150 | 0.013 | 1.016 | 0.006 | 0.949 | 3.112 | 0.950 | 3.083 | 0.945 | 3.218 | 0.579 | 0.852 | 22.582 | 0.473 |
| | 8 | WIPW | -2.501 | -2.494 | 0.184 | 0.012 | 1.035 | 0.007 | 0.941 | 3.326 | 0.945 | 3.225 | 0.945 | 3.218 | 0.692 | 1.904 | 33.885 | 0.735 |
| | 8 | AIPW | -2.501 | -2.495 | 0.122 | 0.035 | 1.014 | 0.006 | 0.947 | 3.176 | 0.939 | 3.392 | 0.954 | 2.963 | 0.473 | 0.586 | 14.912 | 0.310 |
| | 8 | WAIPW | -2.501 | -2.494 | 0.121 | 0.043 | 1.019 | 0.007 | 0.945 | 3.218 | 0.942 | 3.300 | 0.954 | 2.963 | 0.467 | 0.580 | 14.651 | 0.306 |
| IPW(0.75) | 8 | IPW | -2.501 | -2.500 | 0.155 | -0.035 | 1.033 | 0.001 | 0.938 | 3.421 | 0.947 | 3.157 | 0.943 | 3.290 | 0.582 | 1.190 | 23.895 | 0.535 |
| | 8 | WIPW | -2.501 | -2.493 | 0.191 | -0.006 | 1.070 | 0.007 | 0.934 | 3.521 | 0.942 | 3.306 | 0.935 | 3.487 | 0.683 | 2.779 | 36.482 | 0.905 |
| | 8 | AIPW | -2.501 | -2.501 | 0.125 | -0.017 | 1.037 | 0.000 | 0.938 | 3.416 | 0.948 | 3.135 | 0.941 | 3.327 | 0.473 | 0.775 | 15.560 | 0.331 |
| | 8 | WAIPW | -2.501 | -2.498 | 0.123 | 0.004 | 1.048 | 0.003 | 0.938 | 3.401 | 0.945 | 3.224 | 0.941 | 3.322 | 0.462 | 0.705 | 15.140 | 0.318 |
| IPW(1) | 8 | IPW | -2.501 | -2.504 | 0.157 | -0.076 | 1.000 | -0.003 | 0.946 | 3.206 | 0.958 | 2.837 | 0.938 | 3.402 | 0.593 | 1.710 | 24.601 | 0.925 |
| | 8 | WIPW | -2.501 | -2.494 | 0.194 | -0.029 | 1.051 | 0.006 | 0.940 | 3.359 | 0.945 | 3.225 | 0.938 | 3.407 | 0.680 | 3.446 | 37.715 | 1.598 |
| | 8 | AIPW | -2.501 | -2.502 | 0.127 | -0.036 | 1.007 | -0.002 | 0.951 | 3.063 | 0.952 | 3.024 | 0.949 | 3.107 | 0.482 | 1.393 | 16.051 | 0.562 |
| | 8 | WAIPW | -2.501 | -2.499 | 0.121 | -0.011 | 1.011 | 0.002 | 0.951 | 3.058 | 0.952 | 3.034 | 0.949 | 3.102 | 0.464 | 0.952 | 14.563 | 0.336 |
| SR | 8 | IPW | -2.501 | -2.500 | 0.161 | 0.001 | 1.029 | 0.000 | 0.942 | 3.301 | 0.944 | 3.263 | 0.945 | 3.236 | 0.621 | 0.824 | 25.995 | 0.527 |
| | 8 | WIPW | -2.501 | -2.502 | 0.222 | -0.004 | 1.051 | -0.001 | 0.939 | 3.380 | 0.944 | 3.263 | 0.943 | 3.274 | 0.847 | 1.764 | 49.424 | 1.002 |
| | 8 | AIPW | -2.501 | -2.500 | 0.131 | 0.004 | 1.033 | 0.001 | 0.941 | 3.338 | 0.942 | 3.317 | 0.946 | 3.191 | 0.501 | 0.547 | 17.044 | 0.350 |
| | 8 | WAIPW | -2.501 | -2.500 | 0.132 | 0.004 | 1.035 | 0.001 | 0.941 | 3.322 | 0.943 | 3.290 | 0.946 | 3.191 | 0.504 | 0.549 | 17.355 | 0.358 |
| TS(0.25) | 8 | IPW | -2.501 | -2.498 | 0.145 | 0.004 | 0.995 | 0.003 | 0.949 | 3.106 | 0.947 | 3.163 | 0.953 | 2.999 | 0.572 | 0.855 | 21.063 | 0.447 |
| | 8 | WIPW | -2.501 | -2.495 | 0.140 | 0.018 | 0.993 | 0.005 | 0.948 | 3.135 | 0.945 | 3.213 | 0.955 | 2.926 | 0.555 | 0.800 | 19.750 | 0.409 |
| | 8 | AIPW | -2.501 | -2.495 | 0.118 | 0.036 | 0.988 | 0.006 | 0.950 | 3.088 | 0.944 | 3.246 | 0.958 | 2.824 | 0.469 | 0.807 | 13.944 | 0.327 |
| | 8 | WAIPW | -2.501 | -2.494 | 0.117 | 0.044 | 0.997 | 0.007 | 0.948 | 3.146 | 0.944 | 3.246 | 0.957 | 2.869 | 0.461 | 0.568 | 13.674 | 0.286 |
| TS(0.50) | 8 | IPW | -2.501 | -2.500 | 0.148 | -0.025 | 1.019 | 0.000 | 0.941 | 3.322 | 0.951 | 3.047 | 0.942 | 3.317 | 0.572 | 1.047 | 21.975 | 0.479 |
| | 8 | WIPW | -2.501 | -2.496 | 0.142 | 0.002 | 1.018 | 0.005 | 0.942 | 3.306 | 0.948 | 3.140 | 0.946 | 3.202 | 0.547 | 0.981 | 20.223 | 0.444 |
| | 8 | AIPW | -2.501 | -2.498 | 0.121 | 0.002 | 1.014 | 0.003 | 0.944 | 3.263 | 0.947 | 3.174 | 0.949 | 3.106 | 0.469 | 0.759 | 14.641 | 0.316 |
| | 8 | WAIPW | -2.501 | -2.495 | 0.120 | 0.019 | 1.024 | 0.006 | 0.943 | 3.284 | 0.942 | 3.311 | 0.951 | 3.065 | 0.457 | 0.663 | 14.326 | 0.312 |
| TS(0.75) | 8 | IPW | -2.501 | -2.502 | 0.154 | -0.051 | 1.029 | -0.002 | 0.945 | 3.219 | 0.947 | 3.163 | 0.941 | 3.338 | 0.578 | 1.546 | 23.712 | 0.572 |
| | 8 | WIPW | -2.501 | -2.495 | 0.146 | -0.012 | 1.032 | 0.005 | 0.944 | 3.263 | 0.942 | 3.317 | 0.945 | 3.213 | 0.545 | 1.252 | 21.267 | 0.471 |
| | 8 | AIPW | -2.501 | -2.500 | 0.125 | -0.028 | 1.026 | 0.000 | 0.946 | 3.186 | 0.947 | 3.174 | 0.949 | 3.112 | 0.474 | 1.320 | 15.653 | 0.369 |
| | 8 | WAIPW | -2.501 | -2.496 | 0.123 | 0.001 | 1.042 | 0.005 | 0.942 | 3.311 | 0.942 | 3.317 | 0.946 | 3.191 | 0.457 | 0.871 | 15.192 | 0.338 |
| TS(1) | 8 | IPW | -2.501 | -2.500 | 0.165 | -0.047 | 1.049 | 0.000 | 0.939 | 3.380 | 0.948 | 3.135 | 0.941 | 3.322 | 0.593 | 2.263 | 27.351 | 0.848 |
| | 8 | WIPW | -2.501 | -2.491 | 0.151 | 0.001 | 1.039 | 0.009 | 0.940 | 3.354 | 0.944 | 3.246 | 0.943 | 3.274 | 0.552 | 1.606 | 22.933 | 0.634 |
| | 8 | AIPW | -2.501 | -2.497 | 0.132 | -0.004 | 1.045 | 0.004 | 0.942 | 3.317 | 0.948 | 3.146 | 0.944 | 3.252 | 0.486 | 2.285 | 17.421 | 0.495 |
| | 8 | WAIPW | -2.501 | -2.491 | 0.128 | 0.031 | 1.051 | 0.009 | 0.938 | 3.416 | 0.939 | 3.380 | 0.944 | 3.252 | 0.462 | 1.519 | 16.468 | 0.609 |
| WAIPW(0.25) | 8 | IPW | -2.501 | -2.505 | 0.152 | -0.041 | 1.019 | -0.005 | 0.947 | 3.169 | 0.951 | 3.041 | 0.943 | 3.274 | 0.590 | 0.771 | 23.213 | 0.454 |
| | 8 | WIPW | -2.501 | -2.504 | 0.151 | -0.034 | 1.020 | -0.003 | 0.946 | 3.197 | 0.951 | 3.047 | 0.943 | 3.279 | 0.583 | 0.766 | 22.776 | 0.447 |
| | 8 | AIPW | -2.501 | -2.505 | 0.124 | -0.043 | 1.019 | -0.005 | 0.947 | 3.180 | 0.950 | 3.071 | 0.942 | 3.317 | 0.480 | 0.525 | 15.327 | 0.298 |
| | 8 | WAIPW | -2.501 | -2.504 | 0.124 | -0.039 | 1.023 | -0.004 | 0.941 | 3.322 | 0.950 | 3.071 | 0.943 | 3.284 | 0.479 | 0.518 | 15.396 | 0.302 |
| WAIPW(0.5) | 8 | IPW | -2.501 | -2.499 | 0.151 | -0.007 | 1.036 | 0.002 | 0.943 | 3.266 | 0.949 | 3.119 | 0.946 | 3.190 | 0.573 | 0.850 | 22.795 | 0.464 |
| | 8 | WIPW | -2.501 | -2.496 | 0.148 | 0.010 | 1.043 | 0.005 | 0.940 | 3.359 | 0.943 | 3.266 | 0.942 | 3.313 | 0.557 | 0.861 | 21.868 | 0.451 |
| | 8 | AIPW | -2.501 | -2.500 | 0.123 | -0.005 | 1.026 | 0.001 | 0.944 | 3.246 | 0.947 | 3.155 | 0.946 | 3.204 | 0.470 | 0.562 | 15.093 | 0.307 |
| | 8 | WAIPW | -2.501 | -2.498 | 0.122 | 0.006 | 1.034 | 0.003 | 0.941 | 3.326 | 0.941 | 3.320 | 0.945 | 3.211 | 0.463 | 0.558 | 14.902 | 0.308 |
| WAIPW(0.75) | 8 | IPW | -2.501 | -2.496 | 0.149 | 0.001 | 1.012 | 0.004 | 0.946 | 3.191 | 0.948 | 3.146 | 0.948 | 3.152 | 0.578 | 1.168 | 22.342 | 0.472 |
| | 8 | WIPW | -2.501 | -2.491 | 0.144 | 0.027 | 1.017 | 0.009 | 0.947 | 3.174 | 0.945 | 3.213 | 0.950 | 3.094 | 0.552 | 1.090 | 20.712 | 0.434 |
| | 8 | AIPW | -2.501 | -2.498 | 0.121 | 0.002 | 1.011 | 0.003 | 0.946 | 3.202 | 0.948 | 3.146 | 0.950 | 3.094 | 0.471 | 0.746 | 14.726 | 0.323 |
| | 8 | WAIPW | -2.501 | -2.495 | 0.120 | 0.024 | 1.021 | 0.006 | 0.944 | 3.252 | 0.945 | 3.219 | 0.950 | 3.088 | 0.459 | 0.686 | 14.354 | 0.313 |
| WAIPW(1) | 8 | IPW | -2.501 | -2.500 | 0.154 | -0.029 | 1.022 | 0.001 | 0.945 | 3.234 | 0.952 | 3.024 | 0.943 | 3.284 | 0.584 | 1.545 | 23.744 | 0.548 |

| Randomization Method | Regime | Estimator | True Value | Mean Estimate | SD Estimates | Mean Normalized | SD Normalized | Mean Bias | CI Coverage | SE CI Coverage x 10^3 | LB Coverage | SE LB Coverage x 10^3 | UB Coverage | SE UB Coverage x 10^3 | Mean CI Length | SE CI Length x 10^3 | MSE x 10^3 | SE MSE x 10^3 |
|---|---|---|---|---|---|---|---|---|---|---|---|---|---|---|---|---|---|---|
| | 8 | WIPW | -2.501 | -2.494 | 0.145 | -0.002 | 1.025 | 0.006 | 0.941 | 3.328 | 0.948 | 3.136 | 0.945 | 3.234 | 0.552 | 1.283 | 21.098 | 0.457 |
| | 8 | AIPW | -2.501 | -2.499 | 0.126 | -0.014 | 1.026 | 0.001 | 0.947 | 3.178 | 0.948 | 3.145 | 0.944 | 3.257 | 0.477 | 1.124 | 15.959 | 0.359 |
| | 8 | WAIPW | -2.501 | -2.495 | 0.123 | 0.012 | 1.038 | 0.005 | 0.945 | 3.220 | 0.943 | 3.275 | 0.944 | 3.261 | 0.460 | 0.919 | 15.158 | 0.322 |
| WIPW(0.25) | 8 | IPW | -2.501 | -2.498 | 0.152 | 0.007 | 1.016 | 0.003 | 0.944 | 3.263 | 0.945 | 3.230 | 0.951 | 3.059 | 0.591 | 0.787 | 23.159 | 0.480 |
| | 8 | WIPW | -2.501 | -2.496 | 0.151 | 0.019 | 1.018 | 0.005 | 0.942 | 3.301 | 0.943 | 3.274 | 0.949 | 3.106 | 0.586 | 0.778 | 22.845 | 0.475 |
| | 8 | AIPW | -2.501 | -2.498 | 0.124 | 0.014 | 1.022 | 0.002 | 0.942 | 3.311 | 0.944 | 3.252 | 0.948 | 3.135 | 0.481 | 0.514 | 15.379 | 0.310 |
| | 8 | WAIPW | -2.501 | -2.498 | 0.124 | 0.016 | 1.026 | 0.003 | 0.942 | 3.306 | 0.944 | 3.263 | 0.947 | 3.157 | 0.480 | 0.511 | 15.478 | 0.317 |
| WIPW(0.5) | 8 | IPW | -2.501 | -2.503 | 0.148 | -0.039 | 1.003 | -0.002 | 0.950 | 3.090 | 0.959 | 2.797 | 0.944 | 3.239 | 0.580 | 0.858 | 21.793 | 0.447 |
| | 8 | WIPW | -2.501 | -2.498 | 0.145 | -0.014 | 1.010 | 0.003 | 0.947 | 3.169 | 0.954 | 2.956 | 0.944 | 3.239 | 0.564 | 0.867 | 21.012 | 0.429 |
| | 8 | AIPW | -2.501 | -2.503 | 0.123 | -0.025 | 1.032 | -0.002 | 0.945 | 3.225 | 0.950 | 3.083 | 0.939 | 3.372 | 0.472 | 0.573 | 15.215 | 0.307 |
| | 8 | WAIPW | -2.501 | -2.501 | 0.122 | -0.014 | 1.037 | 0.000 | 0.943 | 3.266 | 0.949 | 3.112 | 0.942 | 3.293 | 0.466 | 0.571 | 14.936 | 0.303 |
| WIPW(0.75) | 8 | IPW | -2.501 | -2.501 | 0.154 | -0.043 | 1.030 | 0.000 | 0.947 | 3.163 | 0.951 | 3.065 | 0.943 | 3.274 | 0.582 | 1.158 | 23.667 | 0.516 |
| | 8 | WIPW | -2.501 | -2.493 | 0.148 | -0.002 | 1.035 | 0.008 | 0.945 | 3.224 | 0.944 | 3.241 | 0.945 | 3.235 | 0.556 | 1.071 | 22.098 | 0.483 |
| | 8 | AIPW | -2.501 | -2.502 | 0.125 | -0.028 | 1.034 | -0.001 | 0.946 | 3.202 | 0.948 | 3.146 | 0.940 | 3.354 | 0.474 | 0.800 | 15.524 | 0.320 |
| | 8 | WAIPW | -2.501 | -2.499 | 0.122 | -0.008 | 1.038 | 0.002 | 0.942 | 3.317 | 0.947 | 3.180 | 0.941 | 3.333 | 0.463 | 0.713 | 14.939 | 0.310 |
| WIPW(1) | 8 | IPW | -2.501 | -2.497 | 0.156 | -0.031 | 1.004 | 0.004 | 0.945 | 3.215 | 0.953 | 2.999 | 0.947 | 3.169 | 0.594 | 1.701 | 24.250 | 0.623 |
| | 8 | WIPW | -2.501 | -2.489 | 0.147 | 0.017 | 1.008 | 0.012 | 0.945 | 3.215 | 0.949 | 3.117 | 0.950 | 3.068 | 0.561 | 1.337 | 21.786 | 0.514 |
| | 8 | AIPW | -2.501 | -2.498 | 0.127 | 0.003 | 1.021 | 0.003 | 0.942 | 3.311 | 0.946 | 3.202 | 0.946 | 3.183 | 0.483 | 1.347 | 16.172 | 0.393 |
| | 8 | WAIPW | -2.501 | -2.495 | 0.123 | 0.025 | 1.032 | 0.006 | 0.939 | 3.381 | 0.940 | 3.346 | 0.945 | 3.211 | 0.465 | 0.922 | 15.139 | 0.337 |

Identifying the Optimal Regime Results for the N=325 Continuous Outcome Scenario

| Randomization Method | Estimator | Proportion Estimating d1 Optimal | SE Proprtion d1 x 10^3 | Proportion Estimating d2 Optimal | SE Proprtion d2 x 10^3 | Proportion Estimating d3 Optimal | SE Proprtion d3 x 10^3 | Proportion Estimating d4 Optimal | SE Proprtion d4 x 10^3 | Proportion Estimating d5 Optimal | SE Proprtion d5 x 10^3 | Proportion Estimating d6 Optimal | SE Proprtion d6 x 10^3 | Proportion Estimating d7 Optimal | SE Proprtion d7 x 10^3 | Proportion Estimating d8 Optimal | SE Proprtion d8 x 10^3 |
|---|---|---|---|---|---|---|---|---|---|---|---|---|---|---|---|---|---|
| AIPW(0.25) | IPW | 0.000 | 0.000 | 0.000 | 0.000 | 0.000 | 0.000 | 0.000 | 0.000 | 0.167 | 5.280 | 0.143 | 4.951 | 0.316 | 6.576 | 0.374 | 6.842 |
| | WIPW | 0.000 | 0.000 | 0.000 | 0.000 | 0.000 | 0.000 | 0.000 | 0.000 | 0.188 | 5.524 | 0.172 | 5.333 | 0.307 | 6.523 | 0.334 | 6.670 |
| | AIPW | 0.000 | 0.000 | 0.000 | 0.000 | 0.000 | 0.000 | 0.000 | 0.000 | 0.144 | 4.966 | 0.108 | 4.393 | 0.310 | 6.541 | 0.438 | 7.017 |
| | WAIPW | 0.000 | 0.000 | 0.000 | 0.000 | 0.000 | 0.000 | 0.000 | 0.000 | 0.144 | 4.966 | 0.111 | 4.443 | 0.315 | 6.569 | 0.430 | 7.003 |
| AIPW(0.5) | IPW | 0.000 | 0.000 | 0.000 | 0.000 | 0.000 | 0.000 | 0.000 | 0.000 | 0.174 | 5.363 | 0.151 | 5.068 | 0.322 | 6.608 | 0.353 | 6.759 |
| | WIPW | 0.000 | 0.000 | 0.000 | 0.000 | 0.000 | 0.000 | 0.000 | 0.000 | 0.188 | 5.530 | 0.170 | 5.313 | 0.323 | 6.613 | 0.319 | 6.591 |
| | AIPW | 0.000 | 0.000 | 0.000 | 0.000 | 0.000 | 0.000 | 0.000 | 0.000 | 0.150 | 5.047 | 0.106 | 4.355 | 0.317 | 6.583 | 0.427 | 6.996 |
| | WAIPW | 0.000 | 0.000 | 0.000 | 0.000 | 0.000 | 0.000 | 0.000 | 0.000 | 0.143 | 4.952 | 0.111 | 4.435 | 0.330 | 6.650 | 0.417 | 6.973 |
| AIPW(0.75) | IPW | 0.000 | 0.000 | 0.000 | 0.000 | 0.000 | 0.000 | 0.000 | 0.000 | 0.170 | 5.315 | 0.155 | 5.121 | 0.307 | 6.526 | 0.367 | 6.818 |
| | WIPW | 0.000 | 0.000 | 0.000 | 0.000 | 0.000 | 0.000 | 0.000 | 0.000 | 0.180 | 5.434 | 0.171 | 5.328 | 0.312 | 6.554 | 0.337 | 6.683 |
| | AIPW | 0.000 | 0.000 | 0.000 | 0.000 | 0.000 | 0.000 | 0.000 | 0.000 | 0.142 | 4.943 | 0.114 | 4.502 | 0.327 | 6.635 | 0.416 | 6.972 |
| | WAIPW | 0.000 | 0.000 | 0.000 | 0.000 | 0.000 | 0.000 | 0.000 | 0.000 | 0.138 | 4.881 | 0.119 | 4.583 | 0.335 | 6.678 | 0.407 | 6.949 |
| AIPW(1) | IPW | 0.000 | 0.000 | 0.000 | 0.000 | 0.000 | 0.000 | 0.000 | 0.000 | 0.183 | 5.469 | 0.154 | 5.106 | 0.313 | 6.557 | 0.350 | 6.747 |
| | WIPW | 0.000 | 0.000 | 0.000 | 0.000 | 0.000 | 0.000 | 0.000 | 0.000 | 0.183 | 5.469 | 0.177 | 5.396 | 0.314 | 6.567 | 0.326 | 6.628 |
| | AIPW | 0.000 | 0.000 | 0.000 | 0.000 | 0.000 | 0.000 | 0.000 | 0.000 | 0.155 | 5.114 | 0.118 | 4.563 | 0.308 | 6.532 | 0.419 | 6.978 |
| | WAIPW | 0.000 | 0.000 | 0.000 | 0.000 | 0.000 | 0.000 | 0.000 | 0.000 | 0.146 | 4.992 | 0.114 | 4.502 | 0.333 | 6.668 | 0.406 | 6.946 |
| AR-1 | IPW | 0.000 | 0.000 | 0.000 | 0.000 | 0.000 | 0.000 | 0.000 | 0.000 | 0.174 | 5.357 | 0.143 | 4.954 | 0.299 | 6.474 | 0.384 | 6.880 |
| | WIPW | 0.000 | 0.000 | 0.000 | 0.000 | 0.000 | 0.000 | 0.000 | 0.000 | 0.170 | 5.315 | 0.138 | 4.884 | 0.304 | 6.505 | 0.388 | 6.891 |
| | AIPW | 0.000 | 0.000 | 0.000 | 0.000 | 0.000 | 0.000 | 0.000 | 0.000 | 0.145 | 4.983 | 0.110 | 4.429 | 0.316 | 6.578 | 0.428 | 6.998 |
| | WAIPW | 0.000 | 0.000 | 0.000 | 0.000 | 0.000 | 0.000 | 0.000 | 0.000 | 0.147 | 5.014 | 0.108 | 4.390 | 0.308 | 6.528 | 0.437 | 7.015 |
| AR-2 | IPW | 0.000 | 0.000 | 0.000 | 0.000 | 0.000 | 0.000 | 0.000 | 0.000 | 0.184 | 5.478 | 0.146 | 4.997 | 0.297 | 6.463 | 0.373 | 6.840 |

| Randomization Method | Estimator | Proportion Estimating d1 Optimal | SE Proprtion d1 x 10^3 | Proportion Estimating d2 Optimal | SE Proprtion d2 x 10^3 | Proportion Estimating d3 Optimal | SE Proprtion d3 x 10^3 | Proportion Estimating d4 Optimal | SE Proprtion d4 x 10^3 | Proportion Estimating d5 Optimal | SE Proprtion d5 x 10^3 | Proportion Estimating d6 Optimal | SE Proprtion d6 x 10^3 | Proportion Estimating d7 Optimal | SE Proprtion d7 x 10^3 | Proportion Estimating d8 Optimal | SE Proprtion d8 x 10^3 |
|---|---|---|---|---|---|---|---|---|---|---|---|---|---|---|---|---|---|
| | WIPW | 0.000 | 0.000 | 0.000 | 0.000 | 0.000 | 0.000 | 0.000 | 0.000 | 0.186 | 5.501 | 0.135 | 4.836 | 0.304 | 6.505 | 0.375 | 6.848 |
| | AIPW | 0.000 | 0.000 | 0.000 | 0.000 | 0.003 | 0.747 | 0.000 | 0.000 | 0.141 | 4.925 | 0.110 | 4.432 | 0.324 | 6.617 | 0.422 | 6.985 |
| | WAIPW | 0.000 | 0.000 | 0.000 | 0.000 | 0.000 | 0.000 | 0.000 | 0.000 | 0.140 | 4.913 | 0.108 | 4.397 | 0.319 | 6.591 | 0.432 | 7.007 |
| IAIPW(0.25) | IPW | 0.000 | 0.000 | 0.000 | 0.000 | 0.000 | 0.000 | 0.000 | 0.000 | 0.167 | 5.278 | 0.154 | 5.105 | 0.329 | 6.647 | 0.349 | 6.743 |
| | WIPW | 0.000 | 0.000 | 0.000 | 0.000 | 0.000 | 0.000 | 0.000 | 0.000 | 0.164 | 5.234 | 0.153 | 5.097 | 0.330 | 6.651 | 0.353 | 6.757 |
| | AIPW | 0.000 | 0.000 | 0.000 | 0.000 | 0.000 | 0.000 | 0.000 | 0.000 | 0.147 | 5.014 | 0.120 | 4.593 | 0.320 | 6.600 | 0.412 | 6.962 |
| | WAIPW | 0.000 | 0.000 | 0.000 | 0.000 | 0.000 | 0.000 | 0.000 | 0.000 | 0.141 | 4.928 | 0.125 | 4.678 | 0.339 | 6.695 | 0.395 | 6.913 |
| IAIPW(0.5) | IPW | 0.000 | 0.000 | 0.000 | 0.000 | 0.000 | 0.000 | 0.000 | 0.000 | 0.175 | 5.378 | 0.160 | 5.182 | 0.318 | 6.584 | 0.347 | 6.734 |
| | WIPW | 0.000 | 0.000 | 0.000 | 0.000 | 0.000 | 0.000 | 0.000 | 0.000 | 0.171 | 5.320 | 0.154 | 5.106 | 0.320 | 6.600 | 0.355 | 6.768 |
| | AIPW | 0.000 | 0.000 | 0.000 | 0.000 | 0.000 | 0.000 | 0.000 | 0.000 | 0.149 | 5.037 | 0.120 | 4.601 | 0.326 | 6.630 | 0.405 | 6.942 |
| | WAIPW | 0.000 | 0.000 | 0.000 | 0.000 | 0.000 | 0.000 | 0.000 | 0.000 | 0.141 | 4.916 | 0.128 | 4.726 | 0.333 | 6.666 | 0.398 | 6.924 |
| IAIPW(0.75) | IPW | 0.000 | 0.000 | 0.000 | 0.000 | 0.000 | 0.000 | 0.000 | 0.000 | 0.172 | 5.340 | 0.156 | 5.135 | 0.314 | 6.565 | 0.357 | 6.778 |
| | WIPW | 0.000 | 0.000 | 0.000 | 0.000 | 0.000 | 0.000 | 0.000 | 0.000 | 0.170 | 5.310 | 0.150 | 5.045 | 0.324 | 6.617 | 0.357 | 6.776 |
| | AIPW | 0.000 | 0.000 | 0.000 | 0.000 | 0.000 | 0.000 | 0.000 | 0.000 | 0.142 | 4.937 | 0.107 | 4.365 | 0.331 | 6.656 | 0.420 | 6.982 |
| | WAIPW | 0.000 | 0.000 | 0.000 | 0.000 | 0.000 | 0.000 | 0.000 | 0.000 | 0.134 | 4.824 | 0.113 | 4.478 | 0.339 | 6.696 | 0.413 | 6.965 |
| IAIPW(1) | IPW | 0.000 | 0.000 | 0.000 | 0.000 | 0.000 | 0.000 | 0.000 | 0.000 | 0.177 | 5.403 | 0.154 | 5.108 | 0.304 | 6.505 | 0.364 | 6.807 |
| | WIPW | 0.000 | 0.000 | 0.000 | 0.000 | 0.000 | 0.000 | 0.000 | 0.000 | 0.165 | 5.248 | 0.148 | 5.028 | 0.309 | 6.537 | 0.377 | 6.856 |
| | AIPW | 0.000 | 0.000 | 0.000 | 0.000 | 0.000 | 0.000 | 0.000 | 0.000 | 0.144 | 4.960 | 0.105 | 4.329 | 0.316 | 6.578 | 0.435 | 7.012 |
| | WAIPW | 0.000 | 0.000 | 0.000 | 0.000 | 0.000 | 0.000 | 0.000 | 0.000 | 0.136 | 4.846 | 0.108 | 4.394 | 0.330 | 6.653 | 0.425 | 6.993 |
| IPW(0.25) | IPW | 0.000 | 0.000 | 0.000 | 0.000 | 0.000 | 0.000 | 0.000 | 0.000 | 0.172 | 5.335 | 0.150 | 5.056 | 0.320 | 6.599 | 0.358 | 6.779 |
| | WIPW | 0.000 | 0.000 | 0.000 | 0.000 | 0.000 | 0.000 | 0.000 | 0.000 | 0.189 | 5.540 | 0.169 | 5.298 | 0.311 | 6.549 | 0.331 | 6.654 |
| | AIPW | 0.000 | 0.000 | 0.000 | 0.000 | 0.000 | 0.000 | 0.000 | 0.000 | 0.147 | 5.003 | 0.116 | 4.536 | 0.325 | 6.627 | 0.412 | 6.960 |
| | WAIPW | 0.000 | 0.000 | 0.000 | 0.000 | 0.000 | 0.000 | 0.000 | 0.000 | 0.144 | 4.963 | 0.125 | 4.684 | 0.332 | 6.661 | 0.399 | 6.925 |
| IPW(0.5) | IPW | 0.000 | 0.000 | 0.000 | 0.000 | 0.000 | 0.000 | 0.000 | 0.000 | 0.165 | 5.244 | 0.148 | 5.027 | 0.322 | 6.608 | 0.365 | 6.811 |
| | WIPW | 0.000 | 0.000 | 0.000 | 0.000 | 0.000 | 0.000 | 0.000 | 0.000 | 0.187 | 5.518 | 0.170 | 5.307 | 0.310 | 6.539 | 0.334 | 6.669 |
| | AIPW | 0.000 | 0.000 | 0.000 | 0.000 | 0.000 | 0.000 | 0.000 | 0.000 | 0.151 | 5.061 | 0.117 | 4.547 | 0.301 | 6.487 | 0.431 | 7.005 |
| | WAIPW | 0.000 | 0.000 | 0.000 | 0.000 | 0.000 | 0.000 | 0.000 | 0.000 | 0.146 | 4.991 | 0.123 | 4.650 | 0.315 | 6.569 | 0.416 | 6.972 |
| IPW(0.75) | IPW | 0.000 | 0.000 | 0.000 | 0.000 | 0.000 | 0.000 | 0.000 | 0.000 | 0.169 | 5.295 | 0.156 | 5.129 | 0.313 | 6.561 | 0.362 | 6.798 |
| | WIPW | 0.000 | 0.000 | 0.000 | 0.000 | 0.000 | 0.000 | 0.000 | 0.000 | 0.189 | 5.533 | 0.170 | 5.313 | 0.313 | 6.556 | 0.329 | 6.644 |
| | AIPW | 0.000 | 0.000 | 0.000 | 0.000 | 0.000 | 0.000 | 0.000 | 0.000 | 0.151 | 5.064 | 0.112 | 4.460 | 0.328 | 6.642 | 0.409 | 6.953 |
| | WAIPW | 0.000 | 0.000 | 0.000 | 0.000 | 0.000 | 0.000 | 0.000 | 0.000 | 0.142 | 4.943 | 0.118 | 4.563 | 0.335 | 6.676 | 0.405 | 6.942 |
| IPW(1) | IPW | 0.000 | 0.000 | 0.000 | 0.000 | 0.000 | 0.000 | 0.000 | 0.000 | 0.172 | 5.343 | 0.145 | 4.980 | 0.316 | 6.578 | 0.366 | 6.813 |
| | WIPW | 0.000 | 0.000 | 0.000 | 0.000 | 0.000 | 0.000 | 0.000 | 0.000 | 0.195 | 5.606 | 0.168 | 5.291 | 0.308 | 6.531 | 0.328 | 6.642 |
| | AIPW | 0.000 | 0.000 | 0.000 | 0.000 | 0.000 | 0.000 | 0.000 | 0.000 | 0.146 | 4.997 | 0.115 | 4.519 | 0.317 | 6.580 | 0.421 | 6.984 |
| | WAIPW | 0.000 | 0.000 | 0.000 | 0.000 | 0.000 | 0.000 | 0.000 | 0.000 | 0.134 | 4.812 | 0.118 | 4.557 | 0.339 | 6.695 | 0.410 | 6.955 |
| SR | IPW | 0.000 | 0.000 | 0.000 | 0.000 | 0.000 | 0.000 | 0.000 | 0.000 | 0.175 | 5.372 | 0.147 | 5.012 | 0.311 | 6.550 | 0.366 | 6.815 |
| | WIPW | 0.000 | 0.000 | 0.000 | 0.000 | 0.000 | 0.000 | 0.000 | 0.000 | 0.193 | 5.582 | 0.177 | 5.401 | 0.291 | 6.427 | 0.338 | 6.692 |
| | AIPW | 0.000 | 0.000 | 0.000 | 0.000 | 0.000 | 0.000 | 0.000 | 0.000 | 0.150 | 5.053 | 0.125 | 4.672 | 0.315 | 6.571 | 0.410 | 6.956 |
| | WAIPW | 0.000 | 0.000 | 0.000 | 0.000 | 0.000 | 0.000 | 0.000 | 0.000 | 0.149 | 5.031 | 0.130 | 4.760 | 0.321 | 6.606 | 0.400 | 6.928 |
| TS(0.25) | IPW | 0.000 | 0.000 | 0.000 | 0.283 | 0.000 | 0.283 | 0.000 | 0.000 | 0.186 | 5.499 | 0.147 | 5.008 | 0.292 | 6.428 | 0.375 | 6.847 |
| | WIPW | 0.000 | 0.000 | 0.000 | 0.000 | 0.000 | 0.000 | 0.000 | 0.000 | 0.181 | 5.441 | 0.138 | 4.881 | 0.299 | 6.475 | 0.382 | 6.873 |
| | AIPW | 0.002 | 0.565 | 0.002 | 0.565 | 0.001 | 0.346 | 0.002 | 0.600 | 0.148 | 5.028 | 0.112 | 4.467 | 0.319 | 6.590 | 0.415 | 6.969 |
| | WAIPW | 0.000 | 0.000 | 0.000 | 0.000 | 0.000 | 0.000 | 0.001 | 0.447 | 0.150 | 5.047 | 0.112 | 4.457 | 0.327 | 6.633 | 0.411 | 6.958 |
| TS(0.50) | IPW | 0.000 | 0.000 | 0.000 | 0.000 | 0.000 | 0.000 | 0.000 | 0.200 | 0.176 | 5.391 | 0.149 | 5.034 | 0.293 | 6.437 | 0.382 | 6.871 |

| Randomization Method | Estimator | Proportion Estimating d1 Optimal | SE Proprtion d1 x 10^3 | Proportion Estimating d2 Optimal | SE Proprtion d2 x 10^3 | Proportion Estimating d3 Optimal | SE Proprtion d3 x 10^3 | Proportion Estimating d4 Optimal | SE Proprtion d4 x 10^3 | Proportion Estimating d5 Optimal | SE Proprtion d5 x 10^3 | Proportion Estimating d6 Optimal | SE Proprtion d6 x 10^3 | Proportion Estimating d7 Optimal | SE Proprtion d7 x 10^3 | Proportion Estimating d8 Optimal | SE Proprtion d8 x 10^3 |
|---|---|---|---|---|---|---|---|---|---|---|---|---|---|---|---|---|---|
| | WIPW | 0.000 | 0.000 | 0.000 | 0.000 | 0.000 | 0.000 | 0.000 | 0.000 | 0.170 | 5.318 | 0.139 | 4.899 | 0.300 | 6.484 | 0.390 | 6.898 |
| | AIPW | 0.003 | 0.799 | 0.002 | 0.692 | 0.000 | 0.000 | 0.003 | 0.799 | 0.142 | 4.943 | 0.113 | 4.478 | 0.313 | 6.556 | 0.423 | 6.988 |
| | WAIPW | 0.000 | 0.000 | 0.000 | 0.000 | 0.000 | 0.000 | 0.003 | 0.774 | 0.140 | 4.913 | 0.113 | 4.485 | 0.322 | 6.610 | 0.421 | 6.983 |
| TS(0.75) | IPW | 0.001 | 0.346 | 0.000 | 0.200 | 0.000 | 0.000 | 0.000 | 0.200 | 0.182 | 5.462 | 0.147 | 5.005 | 0.302 | 6.495 | 0.368 | 6.819 |
| | WIPW | 0.000 | 0.000 | 0.000 | 0.000 | 0.000 | 0.000 | 0.000 | 0.000 | 0.175 | 5.374 | 0.133 | 4.803 | 0.317 | 6.581 | 0.375 | 6.847 |
| | AIPW | 0.002 | 0.565 | 0.005 | 0.957 | 0.000 | 0.000 | 0.004 | 0.936 | 0.149 | 5.034 | 0.108 | 4.397 | 0.326 | 6.628 | 0.407 | 6.947 |
| | WAIPW | 0.000 | 0.000 | 0.000 | 0.000 | 0.000 | 0.000 | 0.004 | 0.893 | 0.139 | 4.887 | 0.107 | 4.368 | 0.338 | 6.688 | 0.413 | 6.964 |
| TS(1) | IPW | 0.000 | 0.200 | 0.000 | 0.283 | 0.000 | 0.000 | 0.000 | 0.283 | 0.199 | 5.647 | 0.157 | 5.151 | 0.295 | 6.450 | 0.348 | 6.735 |
| | WIPW | 0.000 | 0.000 | 0.000 | 0.000 | 0.000 | 0.000 | 0.000 | 0.000 | 0.180 | 5.436 | 0.144 | 4.960 | 0.314 | 6.564 | 0.362 | 6.798 |
| | AIPW | 0.003 | 0.747 | 0.005 | 1.037 | 0.001 | 0.400 | 0.005 | 0.998 | 0.169 | 5.295 | 0.121 | 4.616 | 0.296 | 6.459 | 0.400 | 6.928 |
| | WAIPW | 0.000 | 0.000 | 0.000 | 0.200 | 0.000 | 0.000 | 0.005 | 1.037 | 0.147 | 5.011 | 0.119 | 4.583 | 0.311 | 6.547 | 0.417 | 6.974 |
| WAIPW(0.25) | IPW | 0.000 | 0.000 | 0.000 | 0.000 | 0.000 | 0.000 | 0.000 | 0.000 | 0.168 | 5.293 | 0.146 | 5.000 | 0.318 | 6.589 | 0.367 | 6.816 |
| | WIPW | 0.000 | 0.000 | 0.000 | 0.000 | 0.000 | 0.000 | 0.000 | 0.000 | 0.166 | 5.263 | 0.147 | 5.008 | 0.321 | 6.603 | 0.366 | 6.813 |
| | AIPW | 0.000 | 0.000 | 0.000 | 0.000 | 0.000 | 0.000 | 0.000 | 0.000 | 0.143 | 4.951 | 0.105 | 4.328 | 0.323 | 6.613 | 0.430 | 7.001 |
| | WAIPW | 0.000 | 0.000 | 0.000 | 0.000 | 0.000 | 0.000 | 0.000 | 0.000 | 0.145 | 4.977 | 0.106 | 4.361 | 0.337 | 6.683 | 0.412 | 6.962 |
| WAIPW(0.5) | IPW | 0.000 | 0.000 | 0.000 | 0.000 | 0.000 | 0.000 | 0.000 | 0.000 | 0.172 | 5.338 | 0.154 | 5.102 | 0.301 | 6.485 | 0.374 | 6.842 |
| | WIPW | 0.000 | 0.000 | 0.000 | 0.000 | 0.000 | 0.000 | 0.000 | 0.000 | 0.168 | 5.282 | 0.153 | 5.096 | 0.308 | 6.527 | 0.372 | 6.835 |
| | AIPW | 0.000 | 0.000 | 0.000 | 0.000 | 0.000 | 0.000 | 0.000 | 0.000 | 0.147 | 5.012 | 0.113 | 4.483 | 0.301 | 6.490 | 0.438 | 7.018 |
| | WAIPW | 0.000 | 0.000 | 0.000 | 0.000 | 0.000 | 0.000 | 0.000 | 0.000 | 0.140 | 4.908 | 0.116 | 4.534 | 0.311 | 6.549 | 0.432 | 7.007 |
| WAIPW(0.75) | IPW | 0.000 | 0.000 | 0.000 | 0.000 | 0.000 | 0.000 | 0.000 | 0.000 | 0.183 | 5.467 | 0.156 | 5.137 | 0.307 | 6.524 | 0.354 | 6.763 |
| | WIPW | 0.000 | 0.000 | 0.000 | 0.000 | 0.000 | 0.000 | 0.000 | 0.000 | 0.179 | 5.424 | 0.152 | 5.078 | 0.315 | 6.570 | 0.354 | 6.763 |
| | AIPW | 0.000 | 0.000 | 0.000 | 0.000 | 0.000 | 0.000 | 0.000 | 0.000 | 0.154 | 5.100 | 0.110 | 4.429 | 0.321 | 6.604 | 0.415 | 6.969 |
| | WAIPW | 0.000 | 0.000 | 0.000 | 0.000 | 0.000 | 0.000 | 0.000 | 0.000 | 0.151 | 5.067 | 0.107 | 4.365 | 0.344 | 6.720 | 0.398 | 6.923 |
| WAIPW(1) | IPW | 0.000 | 0.000 | 0.000 | 0.000 | 0.000 | 0.000 | 0.000 | 0.000 | 0.178 | 5.413 | 0.165 | 5.255 | 0.307 | 6.524 | 0.349 | 6.743 |
| | WIPW | 0.000 | 0.000 | 0.000 | 0.000 | 0.000 | 0.000 | 0.000 | 0.000 | 0.168 | 5.291 | 0.155 | 5.119 | 0.313 | 6.557 | 0.364 | 6.805 |
| | AIPW | 0.000 | 0.000 | 0.000 | 0.000 | 0.000 | 0.000 | 0.000 | 0.000 | 0.151 | 5.067 | 0.121 | 4.610 | 0.307 | 6.526 | 0.420 | 6.982 |
| | WAIPW | 0.000 | 0.000 | 0.000 | 0.000 | 0.000 | 0.000 | 0.000 | 0.000 | 0.146 | 4.989 | 0.117 | 4.546 | 0.330 | 6.649 | 0.408 | 6.950 |
| WIPW(0.25) | IPW | 0.000 | 0.000 | 0.000 | 0.000 | 0.000 | 0.000 | 0.000 | 0.000 | 0.176 | 5.386 | 0.150 | 5.053 | 0.318 | 6.589 | 0.355 | 6.770 |
| | WIPW | 0.000 | 0.000 | 0.000 | 0.000 | 0.000 | 0.000 | 0.000 | 0.000 | 0.175 | 5.379 | 0.148 | 5.022 | 0.319 | 6.594 | 0.357 | 6.777 |
| | AIPW | 0.000 | 0.000 | 0.000 | 0.000 | 0.000 | 0.000 | 0.000 | 0.000 | 0.152 | 5.083 | 0.110 | 4.425 | 0.320 | 6.597 | 0.418 | 6.976 |
| | WAIPW | 0.000 | 0.000 | 0.000 | 0.000 | 0.000 | 0.000 | 0.000 | 0.000 | 0.145 | 4.986 | 0.117 | 4.543 | 0.326 | 6.631 | 0.412 | 6.960 |
| WIPW(0.5) | IPW | 0.000 | 0.000 | 0.000 | 0.000 | 0.000 | 0.000 | 0.000 | 0.000 | 0.174 | 5.357 | 0.143 | 4.952 | 0.331 | 6.655 | 0.353 | 6.758 |
| | WIPW | 0.000 | 0.000 | 0.000 | 0.000 | 0.000 | 0.000 | 0.000 | 0.000 | 0.174 | 5.357 | 0.140 | 4.912 | 0.330 | 6.651 | 0.356 | 6.773 |
| | AIPW | 0.000 | 0.000 | 0.000 | 0.000 | 0.000 | 0.000 | 0.000 | 0.000 | 0.152 | 5.075 | 0.109 | 4.399 | 0.319 | 6.594 | 0.420 | 6.982 |
| | WAIPW | 0.000 | 0.000 | 0.000 | 0.000 | 0.000 | 0.000 | 0.000 | 0.000 | 0.144 | 4.963 | 0.113 | 4.470 | 0.331 | 6.655 | 0.413 | 6.964 |
| WIPW(0.75) | IPW | 0.000 | 0.000 | 0.000 | 0.000 | 0.000 | 0.000 | 0.000 | 0.000 | 0.180 | 5.436 | 0.146 | 4.988 | 0.304 | 6.507 | 0.370 | 6.829 |
| | WIPW | 0.000 | 0.000 | 0.000 | 0.000 | 0.000 | 0.000 | 0.000 | 0.000 | 0.177 | 5.403 | 0.144 | 4.960 | 0.315 | 6.570 | 0.364 | 6.805 |
| | AIPW | 0.000 | 0.000 | 0.000 | 0.000 | 0.000 | 0.000 | 0.000 | 0.000 | 0.147 | 5.003 | 0.115 | 4.505 | 0.323 | 6.614 | 0.416 | 6.971 |
| | WAIPW | 0.000 | 0.000 | 0.000 | 0.000 | 0.000 | 0.000 | 0.000 | 0.000 | 0.136 | 4.851 | 0.125 | 4.678 | 0.330 | 6.651 | 0.409 | 6.953 |
| WIPW(1) | IPW | 0.000 | 0.000 | 0.000 | 0.000 | 0.000 | 0.000 | 0.000 | 0.000 | 0.179 | 5.422 | 0.156 | 5.138 | 0.309 | 6.535 | 0.356 | 6.771 |
| | WIPW | 0.000 | 0.000 | 0.000 | 0.000 | 0.000 | 0.000 | 0.000 | 0.000 | 0.175 | 5.377 | 0.147 | 5.012 | 0.320 | 6.600 | 0.357 | 6.777 |
| | AIPW | 0.000 | 0.000 | 0.000 | 0.000 | 0.000 | 0.000 | 0.000 | 0.000 | 0.153 | 5.089 | 0.121 | 4.616 | 0.313 | 6.560 | 0.413 | 6.963 |
| | WAIPW | 0.000 | 0.000 | 0.000 | 0.000 | 0.000 | 0.000 | 0.000 | 0.000 | 0.146 | 4.989 | 0.122 | 4.633 | 0.326 | 6.630 | 0.406 | 6.946 |

Regime 1 Normalized Estimates for WAIPW(0.5) Randomization

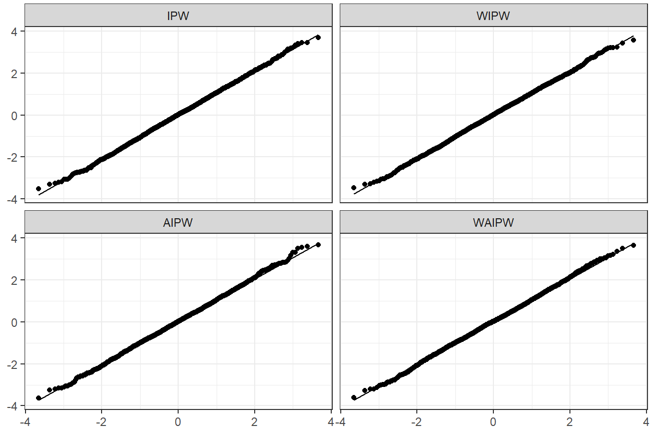


Regime 1 Normalized Estimates for TS(0.50) Randomization

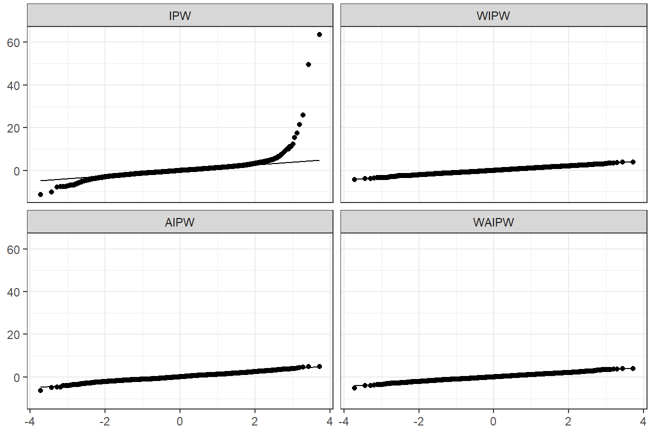


Regime 8 Normalized Estimates for WAIPW(0.5) Randomization

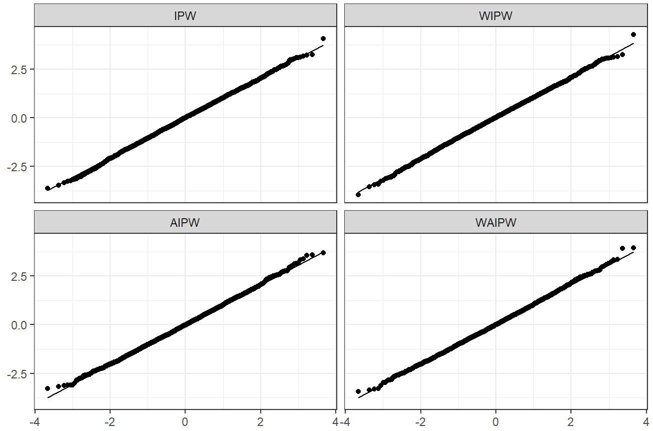

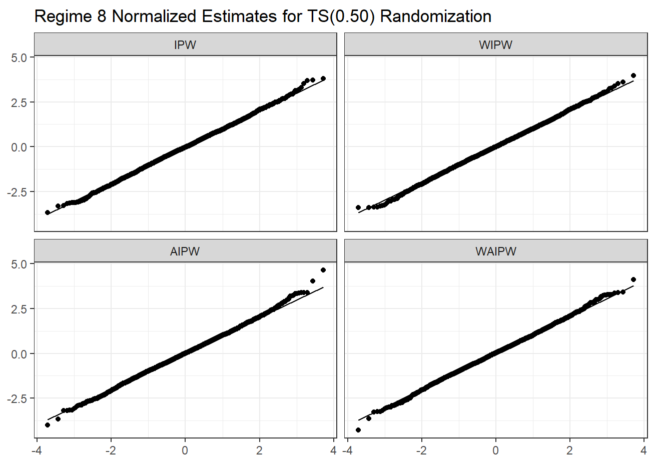
Regime 8 Normalized Estimates for TS(0.50) Randomization
IPW
WIPW
AIPW
WAIPW
5.0
2.5
0.0
-2.5
-4
-2
0
2
4
